# Taming the radical cation intermediate enabled one-step access to structurally diverse lignans

**DOI:** 10.1038/s41467-022-31000-4

**Published:** 2022-06-16

**Authors:** Jia-Chen Xiang, Cédric Fung, Qian Wang, Jieping Zhu

**Affiliations:** 1 Laboratory of Synthesis and Natural Products (LSPN), Institute of Chemical Sciences and Engineering, Ecole Polytechnique Fédérale de Lausanne, EPFL-SB-ISIC-LSPN, BCH5304, 1015 Lausanne, Switzerland; 2 School of Chemistry and Chemical Engineering, Southeast University, 211189 Nanjing, P. R. China

**Keywords:** Photocatalysis, Natural product synthesis

## Abstract

Lignans, in spite of their structural diversity, are all biosynthetically derived from coniferyl alcohol. We report herein a divergent synthesis of lignans from biomass-derived monolignols in a short synthetic sequence. Blue LED irradiation of a dichloromethane solution of dicinnamyl ether derivatives in the presence of Cu(TFA)_2_, an alcohol (2.0 equiv) and a catalytic amount of Fukuzumi’s salt affords the C7-alkoxylated aryltetralin cyclic ethers. Increasing the amount of alcohol under otherwise identical conditions diverts the reaction course to furnish the C7,C7’-dialkoxylated dibenzyltetrahydrofurans, while replacing Cu(TFA)_2_ with diphenyl disulfide (PhSSPh) provides selectively the C7-monoalkoxylated dibenzyltetrahydrofurans. Aza-, thia- and carba-analogues of lignans are equally accessible by simply changing the tethering atom of the allylic alcohols. Concise total syntheses of aglacins A, E, F, brassilignan, and dehydrodimethylconidendrin are documented featuring these transformations.

## Abstract (web summary)

Lignans, in spite of their structural diversity, are all biosynthetically derived from coniferyl alcohol. Here, the authors describe a divergent and stereoselective chemical synthesis of three types of lignans from biomass-derived monolignols, exploiting the different reaction manifolds of highly reactive radical cation intermediate.

## Introduction

**Fig. 1:**
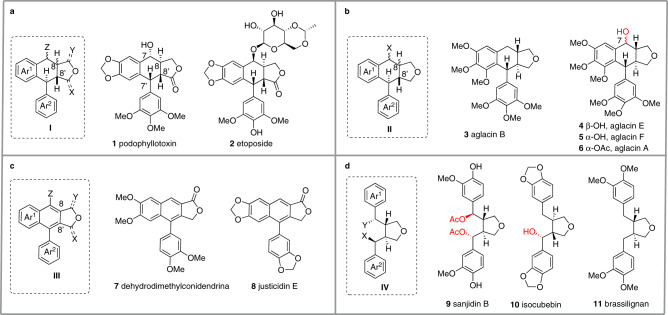
Representative lignan structures. **a** Aryltetralin lactones **I** and its representative natural products: podophyllotoxin (**1**) and its derivative etoposide (**2**), a market anticancer drug; **b** Aryltetralin cyclic ethers **II** and its representative natural products: aglacin B (**3**), aglacin E (**4**), aglacin F (**5**), and aglacin A (**6)**; **c** Arylnaphthalene **III** and its representative natural products: dehydrodimethylconidendrin (**7**) and justicidin E (**8**); **d** 3,4-Dibenzyl tetrahydrofurans **IV** and its representative natural products: sanjidin B (**9**), isocubebin (**10**), and brassilignan (**11**).

Lignans, secondary plant metabolites generated biosynthetically by oxidative dimerization of two phenylpropanoids, display broad structural diversity and oxidation levels^1^. They are widely distributed in the plant kingdom forming a front-line chemical defense against various pathogens and pests as well as stimulating the plant growth and development. The classic lignans (CLs) are defined as those dimers bearing a C8-C8’ bond. Aryltetralin lactones (**I**) such as podophyllotoxin (**1**) and its derivative etoposide (**2**)^2^, aryltetralin cyclic ethers (**II**) such as aglacins (**3**–**6**)^3,4^, arylnaphthalenes (**III**, **7**–**8**)^5^, and 3,4-dibenzyl tetrahydrofurans (**IV**, **9**–**11**)^6–8^ are representative families of CLs. These lignans possess significant pharmacological properties including anticancer, antimicrobial, anti-inflammatory, antiviral, immunosuppressive, cardiovascular and antioxidant activities^9^. For example, etoposide (**2**), a derivative of podophyllotoxin (**1**), is a potent topoisomerase inhibitor that has been used as an anticancer drug for about 40 years (Fig. 1)^10^.

**Fig. 2:**
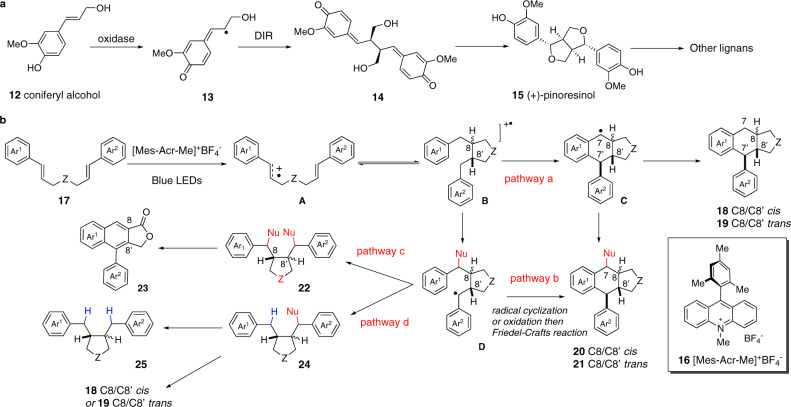
Lignan synthesis. **a** Biosynthesis of lignans; **b** proposed divergent synthesis of lignans.

In spite of enormous structural diversity, all lignans are biosynthesized from coniferyl alcohol (**12**) (Fig. 2a)^11–13^. The regio-, diastereo-, and enantio-selective dimerization of radical **13** generates the *para*-quinone methide **14**, which undergoes the diastereoselective double cyclization to afford (+)-pinoresinol (**15**). The latter is then further converted to structurally diversified lignans via a sequence of enzymatic transformations. A synergistic effect between oxidase and dirigent protein (DIR) has been discovered in the conversion of **12** to **14**. Oxidase is responsible for the generation of the phenoxy radical, depicted as its resonance structure **13**, while DIR ensures the stereoselective dimerization of this reactive radical. In vitro studies indicated that in the absence of DIR, laccases (oxidase) catalyzed dimerization of **13** are neither regio- nor stereo-selective. On the other hand, DIR has no catalytically active oxidative center. It can, however, bind and preorganize radical **13** to ensure the subsequent regio- and stereoselective radical recombination process.

Notwithstanding the great progress recorded in the synthesis of lignans^14^, a concise and divergent synthetic approach allowing access to diverse subsets of lignan scaffolds from simple and cheap starting materials is still highly demanded in order to fully exploit the biological potential of these natural products^15–21^. While the structures of lignans are relatively simple, the control of stereochemistry in the cyclization step remains challenging^22–26^. Inspired by the pioneering work of Yoon^27–29^ and Nicewicz^30,31^ on the visible-light photoredox-catalyzed [2 + 2] and [4 + 2] cycloadditions of styrene derivatives, we recently reported an acridinium salt (Fukuzumi’s salt, **16**)^32–35^ catalyzed intramolecular formal [4 + 2] cycloaddition of functionalized dicinnamyl ethers **17** to aryltetralin cyclic ethers **18** or **19** under blue LED irradiation (Fig. 2b, path a)^36^. We hypothesized that the reaction is initiated by single electron transfer (SET) of the substrate **17** to **16** leading to radical cation **A**. Depending on the electronic nature of the aromatic ring, **A** was further transformed to the distonic radical cation^37^ with either an 8,8’-*cis* or an 8,8’-*trans* disubstituted furan scaffold **B** selectively which, upon cyclization and hydrogen abstraction, was converted to **18** or **19** in high yields and excellent diastereoselectivities. Aiming at developing a concise and divergent synthesis of lignans from biomass-derived monoligonols and related phenylpropanoids, we became interested in diverting this synthetic route to reach C7-functionalized aryltetralin cyclic ethers as well as other lignan scaffolds. We surmised that it might be possible to trap radical cation **B** with an exogenous nucleophile to afford radical intermediate **D** which upon further cyclization and re-aromatization would be converted to the C-7 functionalized aryltetralin cyclic ethers **20** or **21** (Fig. 2b, pathway b). A 6-*exo*-trig-cyclization of the radical **D** followed by oxidation and rearomatization or alternatively, a sequence of oxidation of radical **D** followed by intramolecular Friedel–Crafts reaction of the resulting benzylic cation could account for the formation of **20** or **21**. In addition, we hypothesized that by fine-tuning the reaction conditions, it should be possible to divert the reaction pathways of the radical intermediate **D**. Thus, further SET oxidation of radical **D** followed by nucleophilic addition to the resulting benzylic cation would provide C7,C7’-difunctionalized dibenzyl furans **22** (Fig. 2b, pathway c)^38–40^. One-step conversion of **22** to arylnaphthalene lactones **23** is known. It is also conceivable that the same radical intermediate **D** could undergo hydrogen atom abstraction from a suitable hydrogen atom donor to afford C7-substituted dibenzyl furans **24** which upon further reduction could be converted to **25**. If the incoming nucleophile is also a good nucleofuge, then it should also be possible to convert **24** into aryltetralin cyclic ethers **18** or **19** by an intramolecular Friedel–Crafts reaction. Since the pathway leading to **24** is different from the one-step synthesis of **18**/**19**, the C8/C8’ relative stereochemistry of the tetrahydrofuran obtained by these two routes could also be different, providing therefore an example of stereochemistry divergency. We note that the reactions featuring a key nucleophilic addition to in situ generated radical cation intermediates have recently been developed into powerful tools for performing hydrofunctionalization^41–46^ and difunctionalization^47–53^ of alkenes with unique *anti*-Markovnikov selectivity^54,55^. It is therefore interesting to stress that the regioselectivity of the alkene difunctionalization depicted in Fig. 2b would have to follow the Markovnikov rule to ensure the successful execution of the synthetic pathways leading to lignan type structures. Since access to all the targeted lignans proceeds through the same intermediate **B**, we assume that we might be able to drive the reaction towards one specific manifold by slightly modifying the reaction conditions. The presence of an external nucleophile and its stoichiometry, an additional oxidant capable of oxidizing benzylic radical to carbocation or including a hydrogen atom donor able to undergo the hydrogen atom transfer (HAT) selectively with the benzylic radical are some of the reaction parameters that could change the reaction outcome. We report herein the realization of this endeavor by developing divergent and highly diastereoselective synthesis of three structurally different lignans. Syntheses of aglacins E (**4**), F (**5**) and A (**6**), dehydrodimethylconidendrin (**7**), and brassilignan (**11**) in two to four steps are also documented.

## Results

### Synthesis of C-7 functionalized aryltetralin cyclic ethers from dicinnamyl ether derivatives

**Fig. 3:**
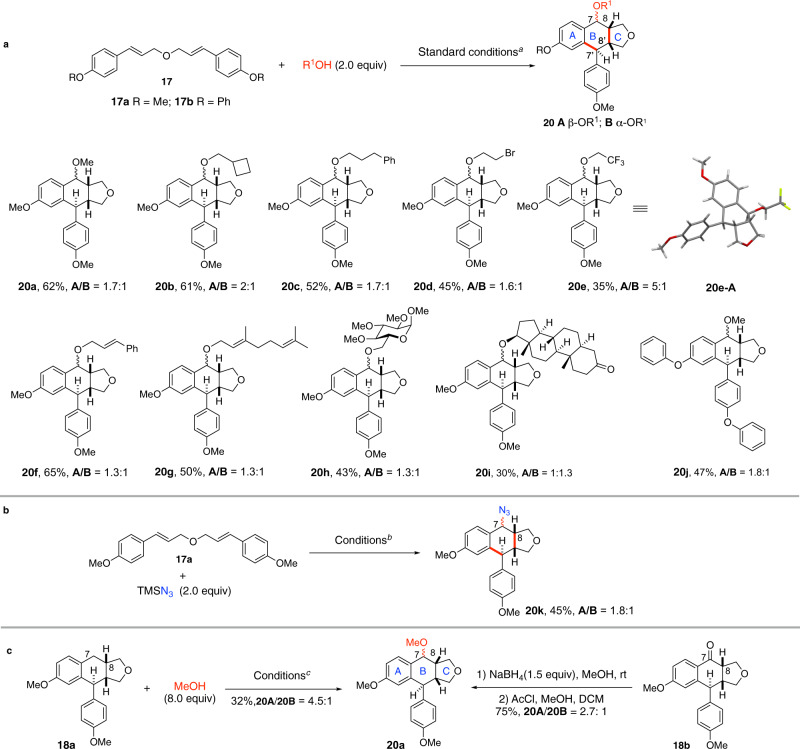
Synthesis of C7-functionalized aryltetraline cyclic ethers. **a** Synthesis of C7-alkoxylated aryltetralin cyclic ethers: Standard conditions: **17**(0.1 mmol), ROH (0.2 mmol, 2.0 equiv), [Mes-Acr-Me]^+^BF_4_^−^ (**16**) (5 mol%), Cu(TFA)_2_•*x*H_2_O (0.12 mmol, 1.2 equiv), DCM (4.0 mL, *c* 0.025 M), 24 W blue LED, 23 °C, isolated yields. CF_3_CH_2_OH (4 equiv) was used in the case of **20e**; **b** Synthesis of C7-azido aryltetralin cyclic ether. TMSN_3_ (0.2 mmol, 2.0 equiv) was used as nucleophile instead of alcohol under otherwise standard conditions; **c** Conditions: **18a** (0.1 mmol), [Mes-Acr-Me]^+^BF_4_^−^ (**16**) (5 mol%), Cu(TFA)_2_•*x*H_2_O (0.12 mmol, 1.2 equiv), DCM (4.0 mL, *c* 0.025 M), 24 W blue LED, N_2_, 23 °C. Yields refer to isolated pure product.

To begin our investigation, conditions were surveyed using di(*p*-methoxy)cinnamyl ether **17a** (R = Me, *E* = 1.05 V) as a test substrate and methanol as a nucleophile (2.0 equiv, Fig. 3). Following our working hypothesis outlined in Fig. 2b, different oxidants and photoredox catalysts were screened. The combination of Cu(TFA)_2_ with Fukuzumi’s salt (**16**, E* = 2.06 V vs SCE) was found to be the most competent in our hands to provide **20a** in 62% isolated yield as a mixture of two diastereomers (**20a-A**:**20a-B** = 1.7/1). We note that (a) using Ir[dF(CF_3_)ppy]_2_dtbpy^+^PF_6_^−^ (E = 1.21 V vs SCE) or 1,2,3,5-tetrakis(carbazole-9-yl)-4,6-dicyanobenzene (4CzlPN) (*E* = 1.35 V vs SCE) as photoredox catalyst afforded cyclobutane derivative as a major product. On the other hand, triphenylpyrylium (E* = 2.30 V vs SCE) was as effective as acridinium salt **16** in accord with the oxidation power of these catalysts; (b) using AgTFA instead of Cu(TFA)_2_ under otherwise identical conditions afforded **20a** in 54% yield (**20a-A**:**20a-B** = 1.7/1), while no desired product was formed using K_2_S_2_O_8_ as oxidant; (c) performing the reaction under air or oxygen atmosphere reduced the yield of **20a** due to the competitive formation of C7-hydroxy and C7-oxo products^56^; (d) the amount of MeOH is of high importance with 2 equivalent being optimum. Increasing the amount of MeOH led to the competitive formation of C7,C7’-dimethoxy-dibenzyltetrahydrofuran (vide infra); (e) the diastereomeric ratio of **20a** remained constant at different time intervals indicating the kinetic selectivity of the reaction. Finally, both photoredox catalyst **16** and blue LED irradiation were needed to ensure the occurrence of the reaction.

The generality of this reaction is shown in Fig. 3a. Cyclobutylmethanol, 3-phenylpropan-1-ol, 2-bromoethan-1-ol, and trifluoroethanol participated in the reaction to afford the corresponding tricyclic lignans **20b**–**20e** in moderate to good yields. Cinnamic alcohol (**20f**) and geraniol (**20g**) containing oxidizable alkene functionalities were also effective nucleophiles indicating the chemoselectivity of the process. Biological molecules such as protected glucose (**20h**) and androstanolone (**20i**) can also be introduced into the C7 position of the tricyclic products illustrating the potential of the present methodology in the synthesis of etoposide analogs as well as late-stage functionalization of bioactive compounds. The presence of a biaryl ether function (**20j**), an important structural motif found in many natural products and pharmaceuticals, was also tolerated. Finally, azide can act as nucleophile to provide the C7-azidated product **20k** under standard conditions (Fig. 3b).

Three chemical bonds are created with concurrent generation of four stereocenters. Therefore, it is interesting to note that only two out of eight diastereoisomers are isolated. Detailed NMR studies indicated that the three stereocenters C7’, C8, and C8’ are generated stereoselectively during the cyclization with a *cis*-fused B/C ring system, while the formation of the C7-OR and the C7-azide bond is non-stereoselective. To confirm this assignment, a direct benzylic C7-methoxylation of aryltetralin cyclic ether **18a** was performed (Fig. 3c). Treatment of the known tricyclic compound **18a** whose relative stereochemistry has been determined by X-ray crystallographic analysis^36^ under standard conditions with an excess of MeOH (8.0 equiv) afforded the same products **20a** as a mixture of two diastereomers^38^. The reaction proceeded much slower presumably due to the sluggish oxidation of the benzylic carbon of compound **18a**, but the diastereoselectivity of this reaction (dr = 4.5:1) was higher than the one-pot process from **17a** and MeOH. This result indicated that the sequence of events leading to **20a** from **17a** and from **18a** might be different and we hypothesized that the methoxylation occurred preceding ring B formation in the conversion of **17a** to **20a** (*cf* Fig. 2b, pathway b, **B** → **D** → **20**/**21**). In addition, reduction of C7-oxo derivative **18b** with sodium borohydride (1.5 equiv) followed by treatment of the resulting C7-hydroxyl compound with methanol in the presence of acetyl chloride provided also a mixture of **20a-A** and **20a-B** (dr = 2.7:1, Fig. 3c). Overall, the results of these two control experiments indicate clearly that **20a-A** and **20a-B** differ only in the C7-stereochemistry. Finally, the structure of **20e-A** was confirmed by X-ray crystallographic analysis. As expected, the ^3^*J* coupling constant between H7 and H8 of the C7-C8 *trans* diastereomer (*J*_H7-H8_ = 9.3 Hz, *pseudo* axial–*pseudo* axial) is larger than that of the *cis* isomer (*J*_H7-H8_ = 4.0 Hz, *pseudo* axial–*pseudo* equatorial). This diagnostic trend is observed in all compounds of this series and is used to differentiate the C7/C8 relative stereochemistry. We note that both diastereomers of C7-hydroxylated aryltetralin cyclic ethers exist in nature.

### Total synthesis of aglacins E, F, and A

**Fig. 4:**
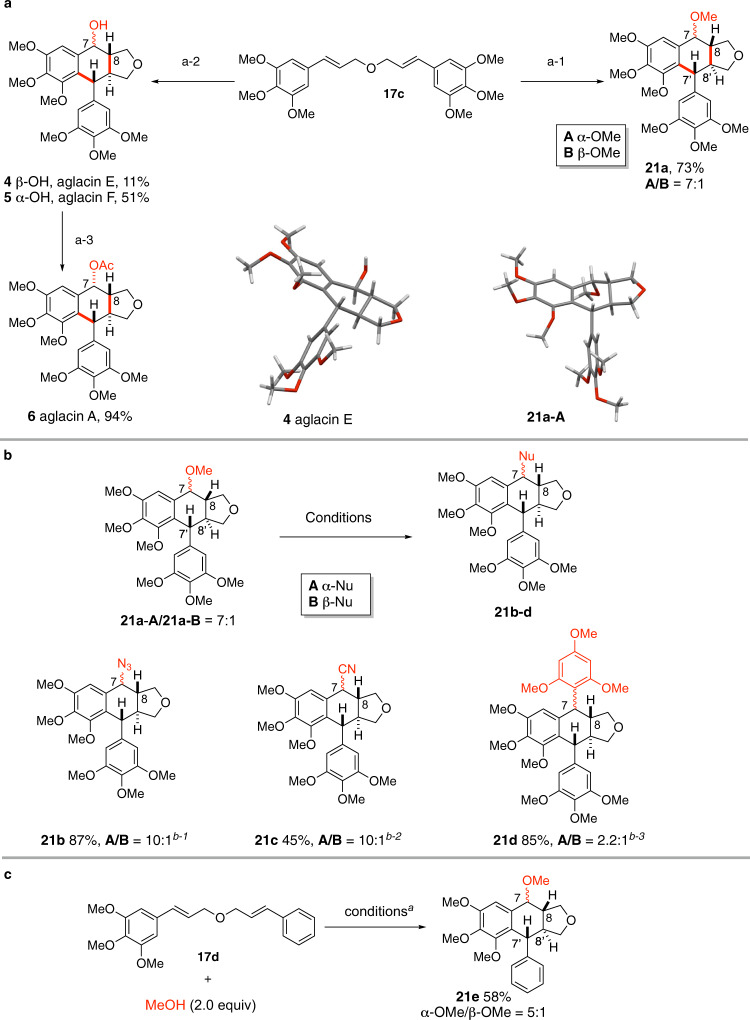
Synthesis of C7-functionalized aryltetralin cyclic ethers. **a** Total synthesis of aglacin E (**4**), F (**5**) and A (**6**). Reagents and conditions: a-1) MeOH (2.0 equiv), [Mes-Acr-Me]^+^BF_4_^−^ (5 mol%), Cu(TFA)_2_•*x*H_2_O (1.2 equiv), DCM, N_2_, RT, Blue LED; a-2) AcOH (2.0 equiv), [Mes-Acr-Me]^+^BF_4_^−^ (5 mol%), Cu(TFA)_2_ (1.2 equiv), DCM, N_2_, RT, Blue LED, aqueous work-up; a-3) Ac_2_O, 1*H*-imidazole (1.2 equiv), DMAP (0.4 equiv), DCM, RT; **b** Synthesis of natural product analogs. Reagents and conditions: b-1) TMSN_3_ (1.5 equiv), TBSOTf (0.2 equiv), DCM, RT, 1 h; b-2) TMSCN (1.5 equiv), Sc(OTf)_3_ (0.2 equiv), DCM, RT, 12 h; b-3) 1,3,5-trimethoxybenzene (1.5 equiv), TBSOTf (0.2 equiv), DCM, RT, 1 h; **c** Cyclization of unsymmetric diallyl ether **17d**. Reagents and conditions: **17d** (0.1 mmol), [Mes-Acr-Me]^+^BF_4_^−^ (**16**) (5 mol%), Cu(TFA)_2_ (0.12 mmol, 1.2 equiv), DCM (4.0 mL, *c* 0.025 M), 24 W blue LED, 23 °C. Yields refer to isolated pure product.

Most of the aryltetralin cyclic ether lignans bear multiple alkoxyl or hydroxyl substituents on the aromatic rings with a *trans*-fused B/C ring system. We have previously shown that the stereochemical outcome of the formal [4 + 2] cycloaddition of dicinnamyl ether derivatives under photoredox catalytic conditions depends on the number of hydroxyl/alkoxyl groups on the aromatic rings^36^. The reaction of di(*p*-methoxy)cinnamyl ether **17a** afforded the tricyclic lignan with a *cis*-fused B/C ring **18**, while those derived from polymethoxylated cinnamic alcohols provided the *trans*-fused B/C ring system **19** (*cf* Fig. 2b). We therefore set out to examine the double cyclization of **17c**, easily prepared from sinapyl alcohol, in the presence of methanol or other related nucleophiles as a means to access rapidly the C7 functionalized aglacin family natural products. Gratefully, reaction of **17c** with MeOH under standard conditions afforded **21a** in 73% overall yield with high diastereoselectivity (dr = 7:1) in favor of the 7,8-*cis*-7’,8’-*trans-*8,8’-*trans* stereoisomer (Fig. 4a). It is interesting to note that the diastereoselectivity in the formation of C7-OMe bond is apparently higher in the case of 3,4-*trans*-substituted furan than the 3,4-*cis* counterpart (*cf* step **B** to **D**, Fig. 2b). This tendency is confirmed in our subsequent synthesis of C7,C7’-difunctionalized tetrahydrofuran lignans (vide infra). Subsequently, we found that acetic acid was also a competent nucleophile to provide, after basic work-up, the separable C7-hydroxylated diastereomers aglacin E (**4**) and aglacin F (**5**) in isolated yields of 11 and 51%, respectively. Acetylation of aglacin F (**5**) furnished aglacin A (**6**) in 94% yield. The relative stereochemistry of compounds **21a-A** and aglacin E (**4**) was further confirmed by X-ray crystallographic analysis. Overall, this represents a two-step total synthesis of aglacins E (**4**), F (**5**), and a three-step synthesis of aglacin A (**6**) from 3,4,5-trimethoxyphenyl cinnamic alcohol. We note that Gao’s group has recently communicated an elegant asymmetric total synthesis of aglacins E (**4**), A (**6**), and B (**3**) in 13- and 14-steps, respectively^21^.

In the presence of a suitable Lewis acid, it is reasonable to expect that compound **21a** can be easily converted to the *para*-quinomethide intermediate which could subsequently be in situ trapped by a nucleophile. Indeed, treatment of a mixture of diastereomers **21a-A**/**21a-B** with TMSN_3_ in the presence of a substoichiometric amount of TBSOTf afforded C7-azidated derivative **21b** in 87% yield as a mixture of two diastereomers with high diastereoselectivity (dr = 10:1, Fig. 4b). The C7-cyano substituted derivative **21c** was similarly prepared using TMSCN as a nucleophile and Sc(OTf)_3_ as a catalyst. Finally, in the presence of TMSOTf (0.2 equiv), electron-rich 1,3,5-trimethoxybenzene can also participate in the reaction with a mixture of **21a-A** and **21a-B** to provide C7-arylated product **21d** in 55% yield (dr = 2.2:1).

The unsymmetric diallyl ether **17d** was transformed chemo- and regio-selectively to the C7 functionalized tricyclic lignan analog **21e** under the standard conditions with an excellent C8/C8’ *trans* and C8’/C7’ *trans* selectivity (Fig. 4c). This result indicated that the reaction was most probably initiated by the oxidation of the more electron-rich double bond.

### Synthesis of C7,C7’-dialkoxylated dibenzyltetrahydrofuran lignans

To divert the reaction from the formation of aryltetralin cyclic ethers to that of furans, the intramolecular trapping of the hypothetic distonic radical cation **B** or radical **D** (*cf* Fig. 2b) would have to be avoided. To render the intermolecular trapping of **B** or **D** more competitive, we speculate that using an excess of nucleophile is a simple yet obvious solution if the kinetic difference between these two processes was not so significant. Indeed, cerium ammonium nitrate (CAN)-mediated oxidative cyclization of cinnamyl ethers in alcohol solvent has been developed for the synthesis of functionalized tetrahydrofurans^57,58^.

**Fig. 5:**
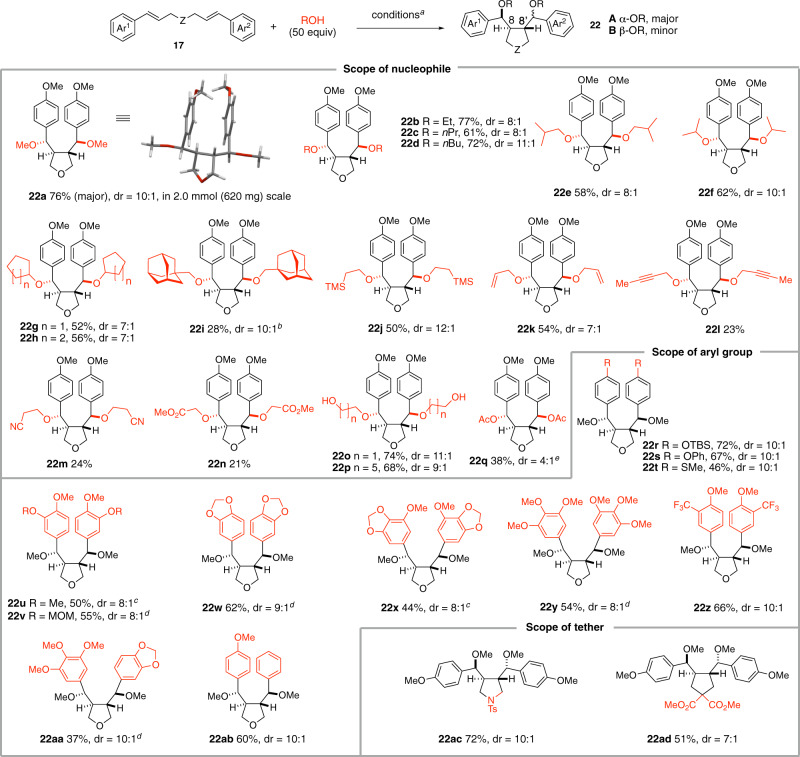
Synthesis of C7,C7’-dialkoxy dibenzyl tetrahydrofurans. Reagents and conditions: (a) **17** (0.1 mmol), [Mes-Acr-Me]^+^BF_4_^−^ (5 mol%), Cu(TFA)_2_•*x*H_2_O (0.12 mmol, 1.2 equiv), alcohol (5.0 mmol, 50.0 equiv), DCM (1 mL, *c* 0.1 M), 24 W blue LED, 23 °C. (b) 20 equiv of alcohol was used; c) Cu(TFA)_2_^.^*x*H_2_O (0.2 mmol) was used; d) AgTFA (0.2 mmol) was used as oxidant instead of Cu(TFA)_2_^.^*x*H_2_O; e) [Mes-Acr-Me]^+^BF_4_^−^ (5 mol%), Cu(TFA)_2_•xH_2_O (1.2 equiv), acetic acid (20 equiv), K_2_HPO_4_ (2.0 equiv), DCM (*c* 0.1 M), 24 W blue LED, 23 °C. Yields refer to isolated pure product.

During the survey of the reaction conditions for the synthesis of C7-methoxylated aryltetralin cyclic ether **20a**, we observed the concurrent formation of C7,C7’-dimethoxylated dibenzyltetrahydrofuran lignan **22a** and found that the yield of **22a** varied depending on the number of equivalent of MeOH used. A brief survey of the stoichiometry of MeOH indicated that 50 equivalent was optimum for the formation of **22a**. However, a lower yield of **22a** was isolated when MeOH was used as a solvent (see SI). In addition to Cu(TFA)_2_, Cu(TFA)_2_•MeCN and Cu(OTf)_2_ were also effective catalysts affording the product **22a** in yields of 62 and 65%, respectively, whereas Cu(OAc)_2_, Cu(ClO_4_)_2_ and CuSO_4_ were far less effective. Overall, under optimized conditions (Fig. 5), reaction of **17a** (Z = O, Ar^1^ = Ar^2^ = 4-methoxyphenyl) with MeOH (50 equiv) afforded **22a**-**A** in 76% isolated yield together with its diastereomer **22a**-**B** (7%, dr = 10:1). The relative stereochemistry of **22a**-**A** was determined by X-ray crystallographic analysis. The C8/C8’ *trans* stereochemistry found in both **22a**-**A** and **22a**-**B** corresponds well to that of the natural dibenzyl furan lignans. Notwithstanding this much wanted stereochemical outcome, it is important to note that it is different from that of the aryltetralin cyclic ether **20a**. The stereochemical divergency in the formation of **20a** and **22a** has important mechanistic implications as it might indicate that the reaction pathways leading to these two products would be different in spite of the similarity of the reaction conditions. Indeed, the amount of nucleophile (2 vs 50 equivalent of MeOH) is the only difference between these two conditions. It is also important to note that the diastereomeric ratio remained the same at a different level of conversion indicating that the observed high diastereoselectivity reflects the kinetic selectivity.

With the optimum conditions in hand, the scope of nucleophiles was first examined. As shown in Fig. 5, primary alcohols (EtOH, *n*PrOH, *n*BuOH, *i*BuOH) and secondary alcohols (*i*PrOH, cyclopentanol, cyclohexanol) participated in the reaction smoothly to afford the corresponding tetrahydrofurans **22b**-**22h** in good to high yields and high diastereoselectivities. Reaction of **17a** (Z = O) with 1-adamantanemethanol gave the desired product **22i** in low yield (28%, dr = 10:1) due presumably to the steric hindrance of the nucleophile. Alcohols bearing diverse functional groups such as trimethylsilyl (TMS), double bond, triple bond, cyano and methoxycabonyl groups were competent nucleophiles in this transformation to afford the desired products (**22j**-**22n**), albeit with reduced yields. Diols such as glycol and hexanediol reacted with **17a** (Z = O) to afford the dialkoxylated products **22o** and **22p**, respectively. In these latter cases, bicyclic compounds resulting from the sequential inter- and intra-molecular trapping of the cationic intermediates were not observed. Acetic acid (AcOH), in the presence of K_2_HPO_4_ (2.0 equiv), can also act as nucleophile to provide the bis-acetoxylated product **22q** in moderate yield. However, primary amines, secondary amines, and thiols failed to take part in this transformation.

Using MeOH as nucleophile, the impact of the electronic nature of the arene on the reaction outcome was next probed. Substrates bearing different substituents on the *para* position of aromatic rings such as silyl ether (OTBS, **22r**), diaryl ether (**22s**), and thioether (SMe, **22t**) reacted smoothly with MeOH to afford the expected products. The dicinnamyl ether derivatives bearing multiple alkoxyl substituents on the phenyl ring participated in the reaction to afford the furans (**22u**-**22y**) in good yields and diastereoselectivities. The presence of an electron-withdrawing group (CF_3_) at the *meta* position of the dicinnamyl ether was tolerated (**22z**, 67% yield, dr = 10:1). Unsymmetric dicinnamyl ethers were similarly converted to the furan derivatives (**22aa**, **22ab**). Finally, 3,4-disubstituted pyrrolidine (**22ac**) and 3,4-disubstituted cyclopentane **22ad** were readily prepared in good to high yields and diastereoselectivities from the corresponding diallylamine (Z = NTs) and 1,6-dienes [Z = C(COOMe)_2_].

**Fig. 6:**
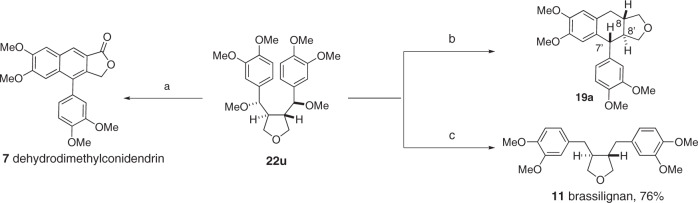
Lignan interconversion. Reagents and conditions: (a) DDQ, TFA, DCM, RT, 24 h, 73% yield; (b) Et_3_SiH, BF_3_•Et_2_O, 94% yield, dr = 11:1; (c) PdCl_2_ (10 mol%), PMHS, MeOH, 40 °C. Yields refer to isolated pure product.

With its nucleophilic arene and a latent electrophilic benzylic carbon, compound **22** is an ideal precursor to other types of lignans. This is illustrated using **22u** as starting material (Fig. 6). Oxidative cyclization of **22u** in the presence of TFA and DDQ afforded dehydrodimethylconidendrin (**7**), an arylnaphthalene lactone retrolignan^59^. Alternatively, reductive cyclization of **22u** in the presence of Et_3_SiH and boron trifluoride-diethyl etherate furnished **19a** in excellent yield and diastereoselectivity. The relative stereochemistry of the C7’-C8’-C8 triad in **19a** is the same as it is found in the aryltetralin cyclic ether lignans. Finally, palladium-catalyzed C-O bond hydrogenolysis of **22u** afforded brassilignan **11** in 76% yield^60^.

### Synthesis of C7-monoalkoxylated dibenzyltetrahydrofuran lignans

The success encountered in the double nucleophilic trapping of the hypothetic distonic radical cation **B** (Fig. 2b) under oxidative conditions prompted us to examine yet another mechanistic possibility. Instead of incorporating an oxidant into the reaction mixture, we suspected that adding an efficient hydrogen atom donor might quench the radical intermediate leading to C-7 mono-functionalized dibenzyl tetrahydrofuran **24**, another sub-class of lignans. While conceptually simple, it is not obvious to accomplish this transformation since the formation of **24** would have to outcompete with that of aryltetralin cyclic ethers **18/19** whose formation involved a very similar reaction sequence^36^. Gratefully, we found that the presence of MeOH changed completely the reaction outcome. The reaction of **17a** (Z = O) with MeOH (50 equiv) in the presence of a catalytic amount of Fukuzumi’s salt **16** (5 mol%) and PhSSPh (0.2 equiv)^30^ under blue LED irradiation afforded **24a** in 80% yield. The amount of MeOH determined the product ratio of **24a** vs aryltetralin cyclic ether **18a**. When only 2 equiv of MeOH was used, **24a** and **18a** were formed in yields of 30 and 27%, respectively. With 50 equiv of MeOH, **18a** was formed in less than 5**%** yield. However, further increasing the amount of MeOH reduced the yield of the desired product **24a**. Finally, thiophenol (PhSH) was also an effective hydrogen donor affording **24a** in slightly reduced yield (72%). We stress that only two diastereomers were isolated with excellent diastereoselectivity (dr > 20:1) although three stereocenters were generated in this transformation. As the diastereomeric ratio remained the same during the course of the reaction, we assumed that the observed diastereoselectivity reflects the kinetic selectivity of the reaction. The relative stereochemistry of **24a** was tentatively assigned on the basis of NMR spectroscopic analysis as well as by analogy to that of compound **22a**.

**Fig. 7:**
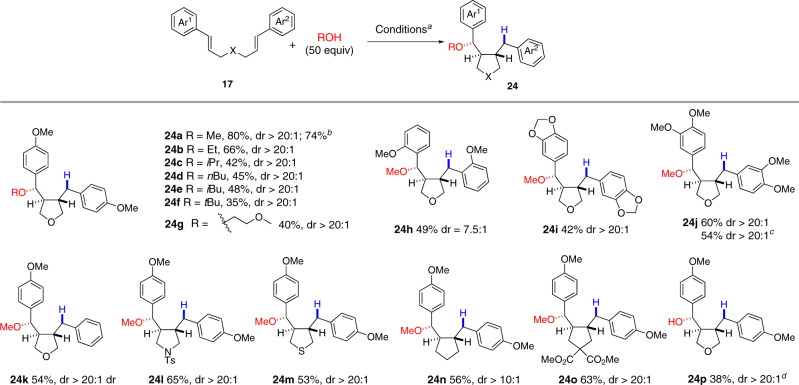
Synthesis of C7-monoalkoxy dibenzyl tetrahydrofuran. Reagents and conditions: (a) **17** (0.1 mmol), [Mes-Acr-Me]^+^BF_4_^−^ (5 mol%), PhSSPh (20 mol%), alcohol (5.0 mmol), DCM (1.0 mL), 24 W blue LED, 23 °C, 36–48 h; (b) 2.5 g scale; (c) 1.0 g scale; (d) THF:H_2_O (v/v = 1:1, 1 mL) was used as solvent. Yields refer to isolated pure product.

The reaction turns out to be applicable to a wide arrange of alcohols and dicinnamyl ethers (Fig. 7). Primary alcohols (MeOH, EtOH, *n*BuOH, *i*BuOH, 2-methoxyethanol), secondary alcohols (*i*PrOH) and even tertiary alcohols participated in the reaction as nucleophiles to afford **24a**–**24g** with excellent diastereoselectivities (dr > 20:1). However, the yields decreased as the alcohols became bulkier. Di-[2-methoxy-(*E*)-cinnamyl]ether and other polymethoxylated derivatives were also competent substrates (**24h**–**24j**). For the unsymmetric diallyl ether, the methoxylation took place regioselectively on the benzylic carbon attached to the more electron-rich phenyl ring (**24k**) indicating that the reaction was initiated by single-electron oxidation of the more electron-rich double bond. In addition to 3,4-disubstituted tetrahydrofurans, pyrrolidine (**24l**), tetrahydrothiophene (**24m**), and pentanes (**24n**, **24o)** were readily accessed by changing the tether of the two cinnamic alcohol derivatives. Finally, using a solvent mixture of THF and H_2_O instead of DCM, hydroxylated tetrahydrofuran **24p** was isolated in 42% yield with excellent stereoselectivity. Reactions leading to **24a** and **24j** were performed a gram scale and the desired products were isolated only in slightly reduced yield.

Under our previously established conditions, the diallyl ether derived from *p*-coumaryl alcohol underwent double cyclization leading to the aryltetralin cyclic ether with a *cis*-fused B/C ring system^36^. Taking advantage of the structural elements in compounds **24**, we deduced that it might be possible to synthesize the tricyclic lignans with a *trans*-fused B/C ring, complementing therefore the direct cyclization strategy. In practice, treatment of a solution of **24a** in dichloromethane with BF_3_•Et_2_O at room temperature afforded the aryltetralin cyclic ether **19b** in 92% yield with excellent diastereoselectivity (dr > 20:1, Fig. 8). Lignan analogs containing a pyrrolidine (**19c**), tetrahydrothiophene (**19d**) and cyclopentane units (**19e**) were similarly prepared from the corresponding mono-methoxylated dibenzyl furans **24l**, **24m**, **24o**, respectively. This two-step process allows us therefore to synthesize the aryltetralin cyclic ether of type **19** bearing one single methoxyl group on the aromatic ring, inaccessible by our previous one-step process^36^.

**Fig. 8:**
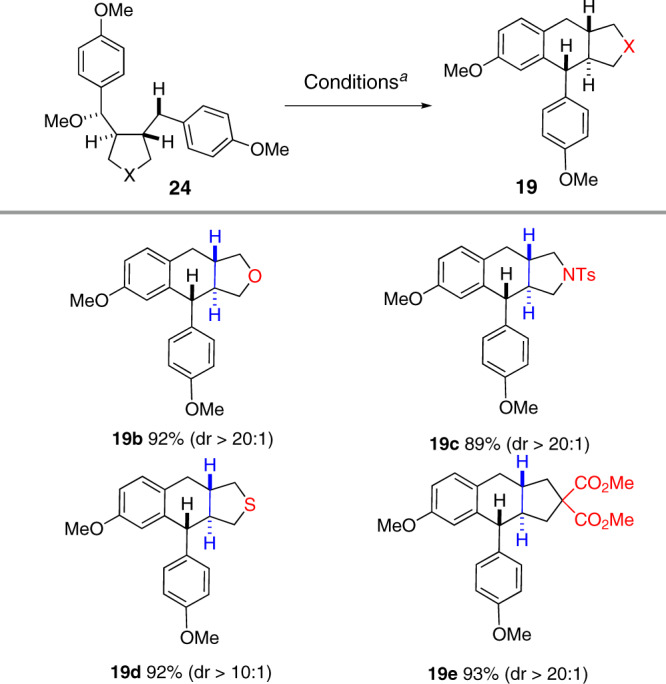
Synthesis of aza-, thia- and carba-analogs of lignans. Reagents and conditions: (a) BF_3_•Et_2_O (2.0 equiv), DCM (*c* 0.01 M), 0 °C to RT.

### Mechanistic consideration

**Fig. 9:**
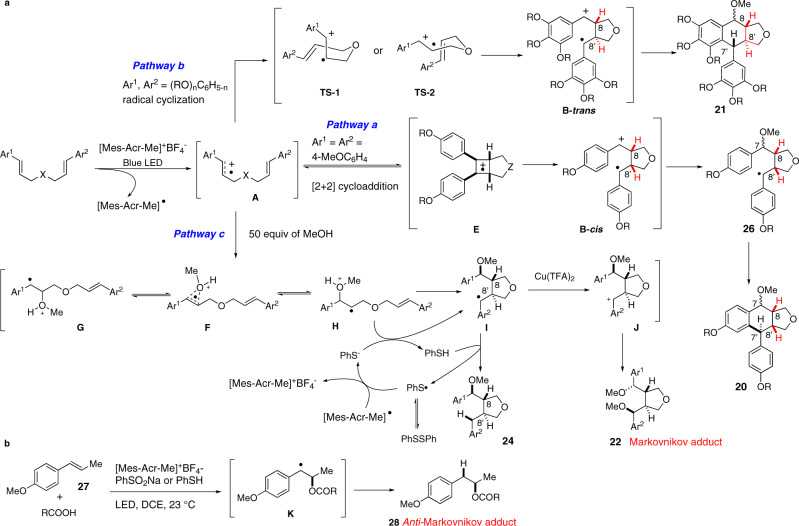
Reaction pathways. **a** Divergent reaction pathways leading to different scaffolds of lignans; **b** *anti*-Markovnikov addition of carboxylic acid to anethole.

The stereodivergency in the formation of aryltetralin cyclic ethers between diallyl ethers derived from *p*-coumaryl alcohol **17a** and sinapyl alcohol **17c** has been observed in our previous study^36^. The fact that the same trend is followed in the synthesis of C7 functionalized derivatives indicated that the present reaction is initiated by the same C8-C8’ bond-forming process (Fig. 9a). In the case of **17a** (Ar^1^ = Ar^2^ = 4-methoxyphenyl), the [2 + 2] cycloaddition of the in situ generated radical cation **A** is known to be very fast (*k* ≃ 10^9^ s^−1^)^61,62^ leading to cyclobutane radical cation **E** (pathway a, Fig. 9a). The cleavage of the benzylic C-C bond, the weakest C-C bond of cyclobutane would afford the distonic 1,4-radical cation **B-*cis***^63^. Nucleophilic addition of methanol to **B-*cis*** would afford intermediate **26** which upon cyclization and rearomatization would furnish **20**. On the other hand, [2 + 2] cycloaddition of the radical cation derived from dicinnamyl ethers with a multiple alkoxyl group on the phenyl ring would be less efficient due to the delocalization of the positive charge to the aromatic ring. Radical cyclization via transition state **TS-1** or **TS-2** according to Beckwith–Houk model^64,65^ would instead take place to provide selectively *trans*-disubstituted tetrahydrofuran radical cation intermediate **B-*trans***, which would then be converted to product **21** with a *trans*-fused B/C ring system (pathway b, Fig. 9a). Whereas both radical cyclization/oxidation/aromatization and oxidation of radical to benzylic carbocation followed by intramolecular Friedel-Crafts reaction could account for the formation of **20** and **21** from **B-*cis*** and **B-*trans***, respectively, the fact that intermediate **I** can be converted either to **22** in the presence of a large excess of external nucleophile or to **24** in the presence of a hydrogen atom donor indicated that the 6-*exo*-trig cyclization of radical **26** and **I** is a relatively slow process. Therefore, oxidation of benzylic radical to carbocation followed by Friedel-Crafts reaction might be a predominant pathway in the formation of compounds **20** and **21**.

The stereoselective formation of tetrahydrofuran is more surprising. In contrast to the formation of aryltetralin cyclic ether, the relative stereochemistry of C8/C8’ is *trans* in all cases irrespective of the substitution pattern on the aromatic rings. For example, tetrahydrofurans **22a** and **22y** of the same relative stereochemistry are produced from **17a** (Ar^1^ = Ar^2^ = 4-methoxyphenyl) and **17c** (Ar^1^ = Ar^2^ = 3,4,5-trimethoxyphenyl), respectively. Direct nucleophilic addition to radical cation affords in general the *anti*-Markovnikov adduct. For example, the reaction of anethole (**27**) with carboxylic acid in the presence of Fukuzumi’s catalyst and a hydrogen donor (PhSO_2_H formed in situ from PhSO_2_Na or PhSH) affords product **28**, via thermodynamically more stable benzylic radical **K**, in excellent yields and regioselectivities (Fig. 9b)^41,42^. Since the Markovnikov rule is respected in all our transformations, an alternative pathway other than the direct addition of nucleophile might be involved. A simplified view would be that, in the presence of a large excess of methanol, the reaction went through a unified mechanism regardless of the electronic nature of the aromatic ring. On the basis of Arnold’s seminal work^66^, it would be reasonable to hypothesize that methanol will react with radical cation **A** to form firstly a three-membered adduct **F** (pathway c, Fig. 9a). The cleavage of benzylic C-O bond from **F** would afford the thermodynamically more stable benzylic radical **G**, while the rupture of the homobenzylic C-O bond would provide the secondary radical **H** which is less stable than **G**. However, radical **H** is pre-disposed for the subsequent kinetically fast 5-*exo*-trig radical cyclization to furnish the new benzylic radical with concurrent formation of the *trans*-disubstituted tetrahydrofuran radical **I** according to Beckwith-Houk model. Oxidation of the latter by Cu(II) would afford carbocation **J** which, upon diastereoselective addition of MeOH, would be converted to product **22**. Radical cation **G** could also undergo a 6-*exo*-trig radical cyclization, but it would be slower than the cyclization of **H**. Overall, under dynamic kinetic conditions, the Markovnikov adduct **22** was formed at the expense of the *anti*-Markovnikov adduct. The involvement of the three-membered adduct **F** and the subsequent Curtin-Hammett scenario could therefore account for the observed regioselectivity in our case.

As expected, in the presence of a suitable hydrogen donor, intermediate **I** is transformed to the C-7 mono-methoxylated tetrahydrofuran **24** via intermolecular hydrogen atom transfer process (HAT). In our case, the in situ generated thiophenol is thought to act as a hydrogen atom donor. Thus, SET between [Mes-Acr-Me]^•^ and thiyl radical, generated in situ via homolysis of diphenyl disulfide^67^ would produce thiophenolate PhS^-^ with concurrent regeneration of the Fukuzumi’s salt. Deprotonation of intermediate **H** by PhS^-^ would generate the thiophenol which upon an intermolecular HAT with radical **I** would afford product **24**. The regioselective formation of **24k** from unsymmetrically substituted diallyl ether is also in line with the proposed radical cyclization on the way to the tetrahydrofuran core. The excellent stereoselectivity observed in the formation of acyclic benzylic stereocenters remains nevertheless puzzling at the present stage of development.

## Discussion

We report in this paper a unified strategy to access diverse subclasses of lignans from biomass-derived monolignols^68^. Under photoredox catalytic conditions, C-7 functionalized aryltetralin cyclic ethers, dibenzyl tetrahydrofurans with different oxidation levels at benzylic positions are readily prepared in one operation by varying the stoichiometry of nucleophiles and additives (oxidant or hydrogen donor). Up to four stereocenters are generated with excellent diastereoselectivities. Applying these transformations, natural products such as aglacins A, E, F, brassilignan, and dehydrodimethylconidendrin are synthesized in only two to four steps. Aza-, thia-, and carba-analogs of lignans are equally accessible by simply changing the tethering atom of the allylic alcohols.

## Methods

Unless otherwise stated, starting materials were purchased from Aldrich and/or Fluka. Solvents were purchased in high-performance liquid chromatography quality, degassed by purging thoroughly with nitrogen, and dried over activated molecular sieves of appropriate size. Alternatively, they were purged with argon and passed through alumina columns in a solvent purification system (Innovative Technology). The conversion was monitored by thin-layer chromatography (TLC) using Merck TLC silica gel 60 F254. Compounds were visualized by ultraviolet light at 254 nm and by dipping the plates in an ethanolic vanillin/sulfuric acid solution or an aqueous potassium permanganate solution followed by heating. Flash column chromatography was performed over silica gel (230−400 mesh).

NMR spectra were recorded on a Brüker AvanceIII-400, Brüker Avance-400, or Brüker DPX-400 spectrometer at room temperature, ^1^H frequency is at 400.13 MHz, and ^13^C frequency is at 100.62 MHz. Chemical shifts (*δ*) were reported in parts per million (ppm) relative to residual solvent peaks rounded to the nearest 0.01 for proton and 0.1 for carbon (*ref: CHCl*_*3*_ *[*^*1*^*H: 7.26*, ^*13*^*C: 77.16]*. Coupling constants (*J*) were reported in Hz to the nearest 0.1 Hz. Peak multiplicity was indicated as follows s (singlet), d (doublet), t (triplet), q (quartet), m (multiplet), and br (broad). Attribution of peaks was done using the multiplicities and integrals of the peaks.

IR spectra were recorded in a Jasco FT/IR-4100 spectrometer outfitted with a PIKE technology MIRacleTM ATR accessory as neat films compressed onto a Zinc Selenide window. The spectra were reported in cm^−1^.

The accurate masses were measured by the mass spectrometry service of the EPFL by ESI-TOF using a QTOF Ultima from Waters or APPI-FT-ICR using a linear ion trap Fourier transform ion cyclotron resonance mass spectrometer from Thermo Scientific.

Melting points were measured using a Stuart SMP30.

## Supplementary information

Supplementary Information

## Data Availability

Crystallographic data for the structure reported in this article have been deposited at the Cambridge Crystallographic Data Centre, under deposition numbers CCDC 2141449 (**20e-A**), CCDC 2141448 (**21a-A**), CCDC 2141447 (**22a**), and CCDC 1968555 (**4**). Copies of the data can be obtained free of charge via https://www.ccdc.cam.ac.uk/structures/. The data supporting the findings of this study are available within the article and its Supplementary Information files.
