# Supplementary material for: Taming the radical cation intermediate enabled one-step access to structurally diverse lignans
Source: Nat Commun. 2022 Jun 16;13:3481. doi: 10.1038/s41467-022-31000-4 (PMC9203495; doi:10.1038/s41467-022-31000-4)
Supplement: Supplementary file 1 — Supplementary Information [file 41467_2022_31000_MOESM1_ESM.pdf]

Supplementary Information

Taming the Radical Cation Intermediate Enabled One-Step Access to Structurally Diverse Lignans

Content of supplementary information

1. Supplementary methods 2
1.1 General information 2
1.2. Optimization of the reaction conditions 3
1.3. General procedures 7
1.4. Control experiments 9
1.5. Synthesis and characterization data 11
1.6. Crystallographic data 41
1.7. Copies of the ^{1}H, ^{13}C and 2D NMR spectra 72
2. Supplementary references 192

1. Supplementary methods

1.1 General information

NMR spectra were recorded on AV2 400 or AV2 500 MHz Bruker spectrometers. Chemical shifts (δ) were reported in parts per million (ppm) relative to residual solvent peaks rounded to the nearest 0.01 for proton and 0.1 for carbon (*ref: $CHCl_3$ [^1H: 7.26, ^{13}C: 77.16]*). Coupling constants (*J*) were reported in Hz to the nearest 0.1 Hz. Multiplicities are abbreviated as follows: singlet (s), doublet (d), triplet (t), quartet (q), doublet-doublet (dd), quintet (quint), sextet (sext), septet (sept), multiplet (m), and broad (b). Infrared spectra were recorded on a JASCO FT/IR-4100 spectrometer. Mass spectra were determined with a Waters ACQUITY H-class UPLC/MS ACQ-SQD by electron ionization (EI positive and negative) or a Finnigan TSQ7000 by electrospray ionization (ESI^+). The accurate masses were measured by the mass spectrometry service of the EPFL by ESI-TOF using a QTOF Ultima from Waters or APPI-FT-ICR using a linear ion trap Fourier transform ion cyclotron resonance mass spectrometer from Thermo Scientific. Melting points were measured using a Stuart SMP30.

Materials and Methods: Unless otherwise stated, starting materials were purchased from commercial sources (Aldrich, Acros, Merck, Fluka, TCI and VWR international). Solvents were purchased in HPLC quality, degassed by purging thoroughly with nitrogen and dried over activated molecular sieves of appropriate size. Alternatively, they were purged with argon and passed through alumina columns in a solvent purification system (Innovative Technology). Reactions were monitored by thin layer chromatography (TLC) using Merck TLC silica gel 60 F254. Compounds were visualized by UV-light at 254 nm and by dipping the plates in an aqueous potassium permanganate solution followed by heating. Flash column chromatography was performed over silica gel (230-400 mesh). Preparative TLC were performed using Merck TLC silica gel 60 F254. The $CDCl_3$ used in the NMR experiments was stored over anhydrous K_2CO_3 before use.

1.2. Optimization of the reaction conditions

1.2.1. Supplementary Table 1: MeOH loading screening in the presence of the oxidant

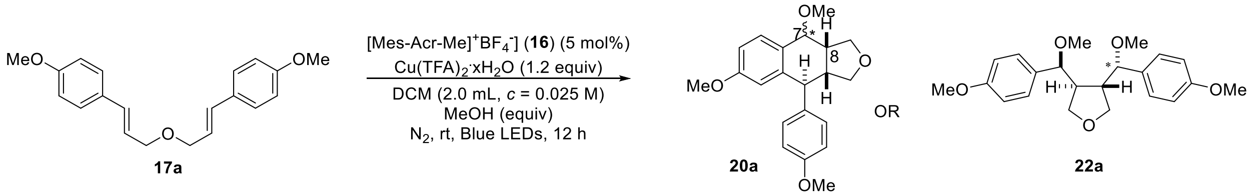

entry	MeOH (equiv)	yield of **20a**	yield of **22a**
1	2	62% (1.7:1 dr) (7,8-*trans* : 7,8-*cis*)	<10%
2	5	49% (2:1 dr)	13%
3	10	41% (3:1 dr)	28%
4	20	27% (5:1 dr)	44%
5	50	10%	65% major (10:1 dr)
6	80	10%	65% major (10:1 dr)
7	as solvent	<10%	42%

17a (0.05 mmol, 1.0 equiv), 9-mesityl-10-methylacridinium tetrafluoroborate (5 mol%), $Cu(TFA)_2{\cdot}xH_2O$ (1.2 equiv), MeOH (50 equiv) and DCM (c = 0.025 M) were irradiated with 24W blue LEDs at 23 ºC. Yield was determined by ^{1}H NMR spectroscopy with CH_2Br_2 as an internal standard.

Results: Using 2.0 equiv of MeOH, **20a** was the major product and **22a** could only be detected less than 10% yield (entry 1). As the dose of MeOH increased, **22a** became the major product (entries 2-6), the optimal equiv of MeOH was determined as 50 equiv (entry 5). However, using MeOH as the solvent reduced the yield of **22a** (entry 7).

1.2.2. Supplementary Table 2: Oxidant screening

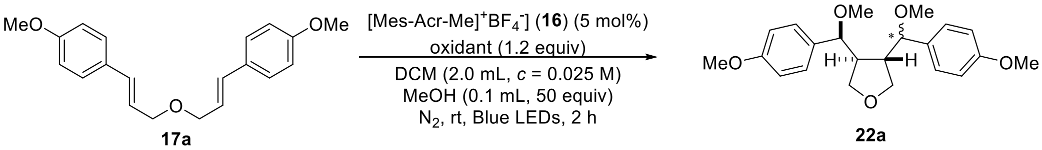

entry	[Cu]	conv.	yield
1	$Cu(TFA)_2{\cdot}xH_2O$	full conv.	69% major (10:1 dr)
2	$Cu(TFA)_2{\cdot}MeCN$	~85%	62%
3	$Cu(OAc)_2$	>95%	31%
4	$Cu(ClO_4)_2{\cdot}xH_2O$	>95%	19%
5	$Cu(OTf)_2$	full conv.	65%
6	$CuSO_4$	~85%	~10% (messy)

17a (0.05 mmol, 1.0 equiv), 9-mesityl-10-methylacridinium tetrafluoroborate (5 mol%), copper source (1.2 equiv), MeOH (50 equiv) and DCM (c = 0.025 M) were irradiated with 24W blue LEDs at 23 ºC for 2 h. Yield was determined by ^{1}H NMR spectroscopy with CH_2Br_2 as an internal standard.

Results: Several copper sources were used as oxidant. $Cu(TFA)_2{\cdot}xH_2O$ still gave the best result.

1.2.3. Supplementary Table 3: Photocatalyst screening

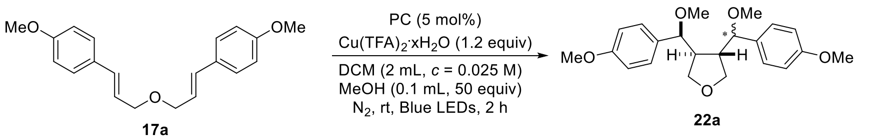

entry	Photocat.		conv.	yield
1	**P1**	E = +1.21 V	>50%	<10%
2	**P2**	E = +1.35 V	35%	14% (26% after 18 h)
3	**P3**	E = +1.65 V	42%	20% (33% after 12 h)
4	**P4**	E = +2.06 V	full conv.	69% major (10:1 dr)
5	**P5**	E = +2.30 V	full conv.	66% major (10:1 dr)

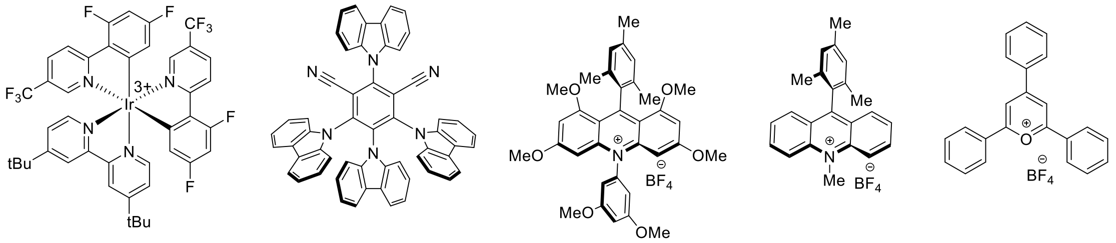

P1 **P2** **P3** **P4** **P5**

17a (0.05 mmol, 1.0 equiv), photocatalyst (for **P1**, 1 mol%, for **P2-5**, 5% mol%), Cu(TFA)$_2$·xH$_2$O (1.2 equiv), MeOH (50 equiv) and DCM (c = 0.025 M) were irradiated with 24W blue LEDs at 23 °C for 2 h. Yield was determined by ^{1}H NMR spectroscopy with CH_2Br_2 as an internal standard.

Results: Using [Mes-Acr-Me]$^+$BF$_4^-$ and TPP provided efficient conversion and better yields (entries 4 and 5).

1.2.4. Supplementary Table 4: Concentration screening in the presence of the oxidant

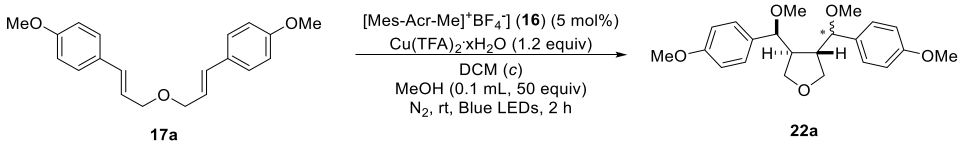

entry	concentration	conv.	yield
1	0.2 M (0.25 mL)	78%	48%[a]
2	0.1 M (0.5 mL)	full conv.	80%[b] (major), 10:1 dr
3	0.05 M (1.0 mL)	full conv.	77%[b] (major), 10:1 dr
4	0.025 M (2.0 mL)	full conv.	71%[b] (major), 10:1 dr

17a (0.05 mmol, 1.0 equiv), 9-mesityl-10-methylacridinium tetrafluoroborate (5 mol%), Cu(TFA)$_2$·xH$_2$O (1.2 equiv), MeOH (50 equiv) and DCM were irradiated with 24W blue LEDs at 23 °C for 2 h. [a] Yield was determined by ^{1}H NMR spectroscopy with CH_2Br_2 as an internal standard. [b] Isolated yield.

Results: The best concentration was found to be 0.1 M.

1.2.5. Supplementary Table 5: MeOH loading screening in the presence of HAT reagent[a]

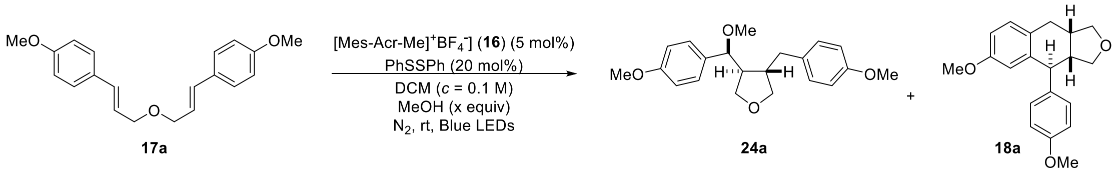

entry	MeOH (equiv)	conv.	**24a** (dr)	**18a**
1	0	full conv.	-	71%
2	2	full conv.	30% (>20:1)	27%
3	10	full conv.	42% (>20:1)	10%
4	20	full conv.	64% (>20:1)	10%
5	50	full conv.	82% (>20:1)	~4%
6	100	full conv.	50% (>20:1)	~2%
7	as solvent	94%	71% (>20:1)	~5%

17a (0.05 mmol, 1.0 equiv), 9-mesityl-10-methylacridinium tetrafluoroborate (5 mol%), PhSSPh (20 mol%), MeOH and DCM (0.5 mL) were irradiated with 24W blue LEDs at 23 ºC for 36 h. [a] Yield was determined by ^{1}H NMR spectroscopy with CH_2Br_2 as an internal standard.

Results: Without adding MeOH, the aryltetralin cyclic ether product **18a** was detected as the major product (entry 1). In the presence of MeOH, compound **24a** was generated as a competitive product (entries 2-7). MeOH loading affected the reaction outcome. The best conditions to afford **24a** were obtained using 50 equiv of MeOH (entry 5) and only less than 5% of **18a** was detected. However, using MeOH as solvent reduced the yield of **24a** (entry 7).

1.2.6. Supplementary Table 6: Concentration screening in the presence of HAT reagent

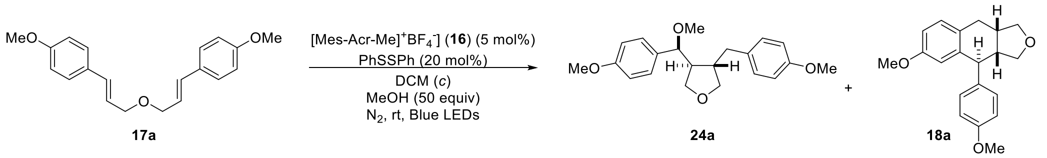

entry	DCM (*c*)	conv.	**24a** (dr)	**18a**
1	0.2 M	full conv.	31% (>20:1)	~3%
2	0.1 M	full conv.	82% (>20:1)	~4%
3	0.05 M	full conv.	77% (>20:1)	7%
4	0.025 M	full conv.	56% (>20:1)	10%

17a (0.05 mmol, 1.0 equiv), 9-mesityl-10-methylacridinium tetrafluoroborate (5 mol%), PhSSPh (20 mol%), MeOH (50 equiv) and DCM were irradiated with 24W blue LEDs at 23 ºC for 36 h. Yield was determined by ^{1}H NMR spectroscopy with CH_2Br_2 as an internal standard.

Results: The best concentration was determined as 0.1 M. Higher or lower concentrations resulted in reduced yields of **24a**.

1.2.7. Supplementary Table 7: HAT reagent screening

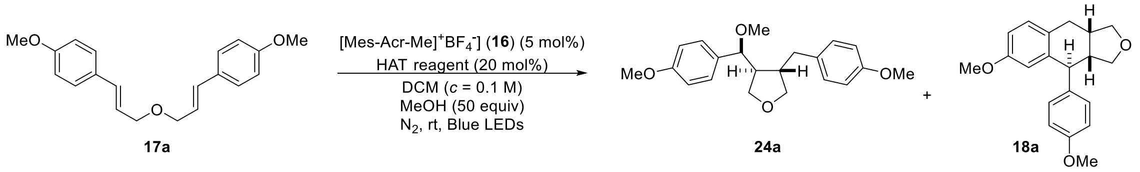

entry	HAT	conv.	**24a**	**18a**
1	PhSSPh	full conv.	80%[b] (> 20 : 1 dr)	~4%
2	PhSH	full conv.	72%[a] (> 20 : 1 dr)	7%

17a (0.05 mmol, 1.0 equiv), 9-mesityl-10-methylacridinium tetrafluoroborate (5 mol%), HAT reagent (20 mol%), MeOH (50 equiv) and DCM (*c* = 0.1 M) were irradiated with 24W blue LEDs at 23 °C for 36 h. [a] Yield was determined by ^{1}H NMR spectroscopy with CH_2Br_2 as an internal standard. [b] Isolated yield.

Results: Using PhSSPh gave a better yield of **24a** compared to PhSH.

1.3. General procedures

1.3.1. General procedures for the synthesis of the substrates

All the substrates are synthesized according to our previous reported methods[1] and Yoon's publications[2,3].

1.3.2. General procedure for the synthesis of products 22 (using 22a as an example)

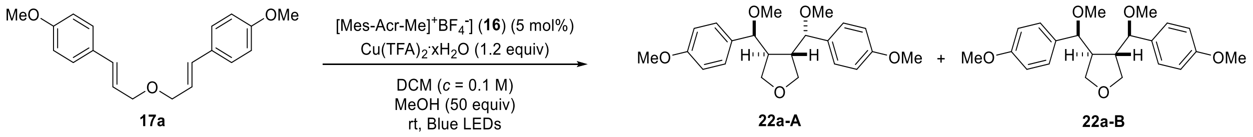

A screw cap tube was charged with cinnamyl ether **17a** (31.0 mg, 0.1 mmol, 1.0 equiv), 9-mesityl-10-methylacridinium tetrafluoroborate (2.0 mg, 0.005 mmol, 0.05 equiv), $Cu(TFA)_2{\cdot}xH_2O$ (34.7 mg, 0.12 mmol, 1.2 equiv), MeOH (0.2 mL, 50 equiv) and DCM (1.0 mL, *c* = 0.1 M) under N_2. The solution was degassed by three Freeze-pump-thaw cycles under N_2 and then irradiated with 24 W blue LEDs strip for 2 h under N_2 atmosphere. The solvent was removed by rotary evaporation, and purification of the residue by preparative TLC (SiO_2, eluent: Petroleum ether/EtOAc = 6:1, Rf = 0.4) afforded the desired product **22a** (29.6 mg, 80% yield) as a colorless solid.

1.3.3. General procedure for the synthesis of products 20 (using 20a as an example)

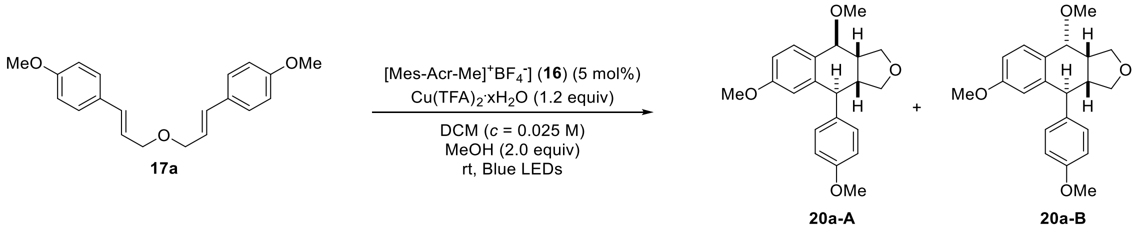

A screw cap tube was charged with cinnamyl ether **17a** (31.0 mg, 0.1 mmol, 1.0 equiv), 9-mesityl-10-methylacridinium tetrafluoroborate (2.0 mg, 0.005 mmol, 0.05 equiv), $Cu(TFA)_2{\cdot}xH_2O$ (34.7 mg, 0.12 mmol, 1.2 equiv), MeOH (8.1 μL, 2 equiv) and DCM (4.0 mL, *c* = 0.025 M) under N_2. The solution was degassed by three Freeze-pump-thaw cycles under N_2 and then irradiated with 24 W blue LEDs strip for 48 h under N_2 atmosphere. The solvent was removed by rotary evaporation, and purification of the residue by preparative TLC (SiO_2, eluent: DCM, Rf = 0.7 for **20a-A** and 0.65 for **20a-B**) afforded the desired product **20a-A** (13.3 mg, 39% yield) and **20a-B** (7.7 mg, 23% yield) as a colorless oil.

1.3.4. General procedure for the synthesis of products 24 (using 24a as an example)

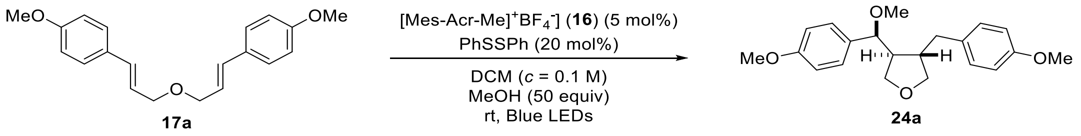

A screw cap tube was charged with cinnamyl ether **17a** (31.0 mg, 0.1 mmol, 1.0 equiv), 9-mesityl-10-methylacridinium tetrafluoroborate (2.0 mg, 0.005 mmol, 0.05 equiv), PhSSPh (4.4 mg, 0.2 mmol, 0.2 equiv), MeOH

(0.2 mL, 50 equiv) and DCM (1.0 mL, c = 0.1 M) under N_2. The solution was degassed by three pump-thaw cycles under N_2 and then irradiated with 24 W blue LEDs strip for 36 h under N_2 atmosphere. The solvent was removed by rotary evaporation, and purification of the residue by preparative TLC (SiO_2, eluent: Petroleum ether/EtOAc = 6:1, Rf = 0.65) afforded the desired product **24a** (27. 5 mg, 80% yield) as a colorless oil.

1.3.5. General procedure for the synthesis of products 19 (using 19b as an example)

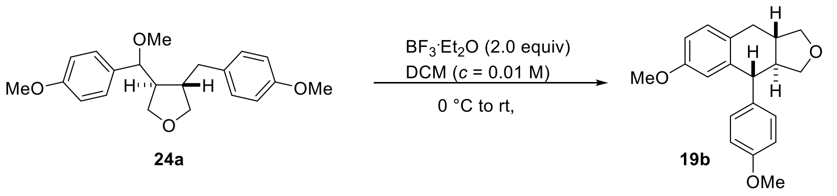

A screw cap tube was charged with **17a** (10.0 mg, 0.032 mmol, 1.0 equiv) and DCM (3.0 mL, c = 0.01 M) under N_2. The solution was degassed by three Freeze-pump-thaw cycles under N_2 and then cooled down to 0 ºC with an ice-bath. Boron trifluoride-diethyl etherate complex (8.1 μL, 2 equiv) was added at this temperature. The solution was stirred for 3 hours and the reaction temperature was slowly raised to room temperature. The reaction mixture was poured into an aqueous saturated $NaHCO_3$. solution and extracted three times with ethyl acetate. The combined organic layers were washed with brine and dried over anhydrous Na_2SO_4. After filtration, the solvent was removed by rotary evaporation and purification of the residue by preparative TLC (SiO_2, eluent: Petroleum ether/EtOAc = 8:1, Rf = 0.5) afforded the desired product **19b** (9.1 mg, 92% yield) as a colorless oil.

1.3.6. General procedure for the synthesis of products 21b-d

Compounds **21b-d** were synthesized according to a reported method[4].

1.4. Control experiments

1.4.1. Transformation from 22a

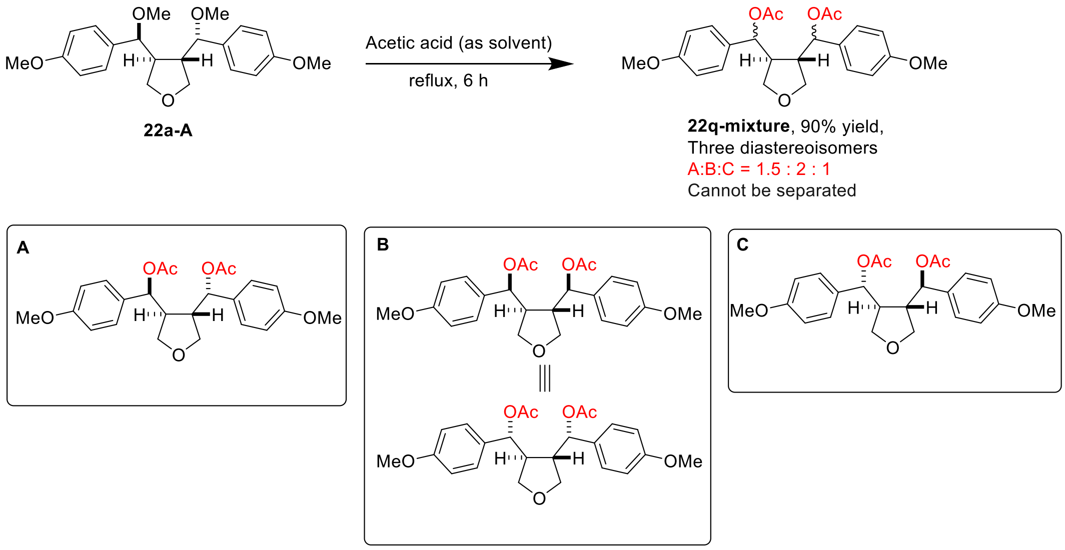

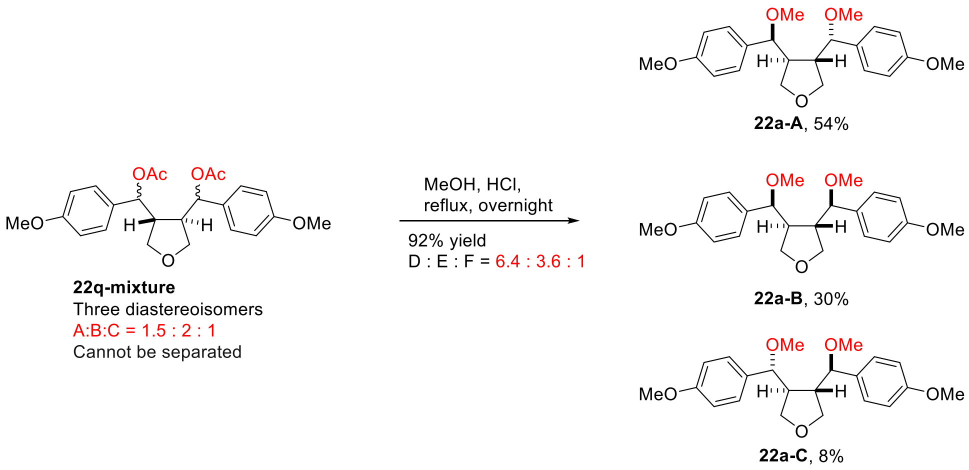

Results: Treating **22a** with acetic acid at reflux temperature for 6 h, the desired acetylation products **22q** could be generated as three inseparable diastereomers. Then, treating **22q** with acid using MeOH as solvent, **22a** could be obtained as three separable diastereomers. **22a-A** (major) and **22a-B** (minor) were detected in our photoredox reaction while **22a-C** was not formed in our photoredox reaction.

1.4.2. Transformation from 24a

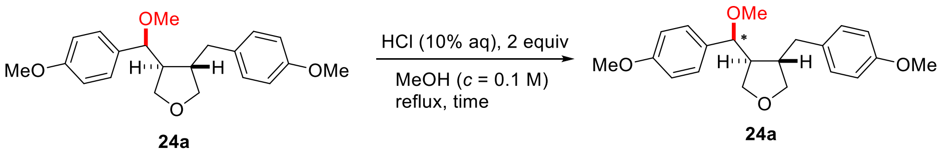

24a pure diastereomer

24a

+

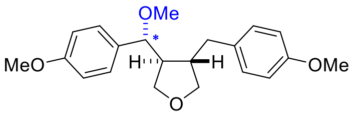

24a-B

entry	time	**24a** : **24a-B**
1	0.5 h	2.7 : 1
2	4h	1.9 : 1
3	8 h	1.9 : 1
4	24 h	2 : 1

Results: Similarly, treating **24a** with acid in MeOH at reflux temperature, another diastereomer was generated. The final ratio is **24a**: **24a-B** = 2 : 1 after 24 h. This new diastereomer **24a-B** was not detected in our photoredox reaction.

1.4.3. Transformation from 18a to 20a and from ketone 18b to 20a.

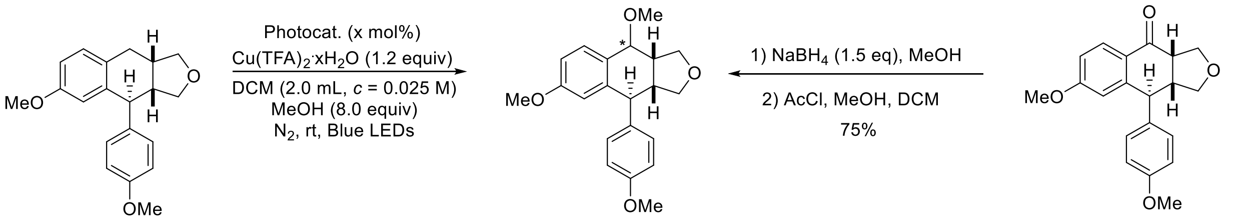

18a (confirmed by x-ray)

20a

18b, from unpublished results

entry	photocat.	conv.	yield of **20a**
1	Ir(dF-CF$_3$-ppy)$_2$(dtbpy)$_3^+$	low conv.	trace
2	Mes-Acr-Me	65%	32% (4.8:1 dr)

Note: dr of **20a** is 2.7:1 from **18b**.

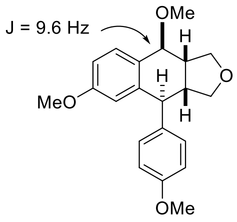

20a-A

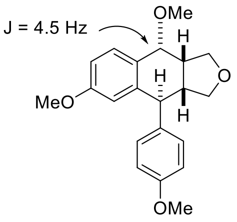

20a-B

Results: **20a** could also be obtained from **18a** based on Yoon's method[5] to confirm the configuration of 7',8'-*trans*-8,8'-*cis*.

1.5. Synthesis and characterization data

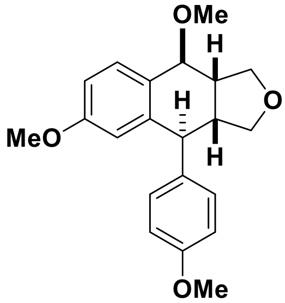

(3a*R*,4*S*,9*S*,9a*S*)-6,9-dimethoxy-4-(4-methoxyphenyl)-1,3,3a,4,9,9a-hexahydronaphtho[2,3-c]furan (**20a-A**)
39% yield, R_f = 0.4 (PE/EtOAc = 10:1), white solid. M.p = 139–140 ºC.
^{1}H NMR (400 MHz, $CDCl_3$) δ 7.36 (d, *J* = 8.3 Hz, 1H), 7.17 – 7.14 (m, 2H), 6.95 – 6.91 (m, 2H), 6.78 (dd, *J* = 8.3, 2.5 Hz, 1H), 6.21 (d, *J* = 2.5 Hz, 1H), 4.19 (d, *J* = 9.6 Hz, 1H), 4.03 (dd, *J* = 9.2, 4.1 Hz, 1H), 3.96 (dd, *J* = 9.2, 6.9 Hz, 1H), 3.84 (s, 3H), 3.71 – 3.61 (m, 3H), 3.67 (s, 3H), 3.66 (s, 3H), 2.73 (tdd, *J* = 10.7, 6.8, 3.8 Hz, 1H), 2.45 (tdd, *J* = 10.4, 6.8, 4.1 Hz, 1H).
^{13}C NMR (101 MHz, $CDCl_3$) δ 158.8 (2C), 142.5, 132.8, 130.7, 130.5, 123.9, 114.4, 113.5, 110.5, 80.8, 73.7, 73.2, 59.8, 55.4, 55.3, 47.1, 47.0, 45.4.
IR (ν_{max}, cm^{-1}) 2933 (m), 2359 (m), 1612 (s), 1581 (m), 1512 (s), 1487 (s), 1462 (s), 1277 (s), 1248 (s), 1180 (s), 1111 (s), 1034 (s), 926 (s), 831 (s), 804 (s).
HRMS (ESI/QTOF) m/z: $[M + Na]^+$ Calcd for $C_{21}H_{24}NaO_4^+$ 363.1567; Found 363.1567.

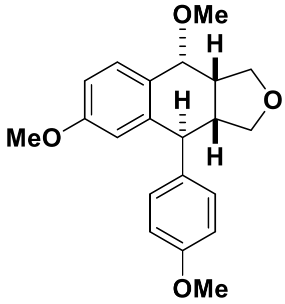

(3a*R*,4*S*,9*R*,9a*S*)-6,9-dimethoxy-4-(4-methoxyphenyl)-1,3,3a,4,9,9a-hexahydronaphtho[2,3-c]furan (**20a-B**)
23% yield, R_f = 0.45 (PE/EtOAc = 10:1), colorless oil.
^{1}H NMR (400 MHz, $CDCl_3$) δ 7.20 (d, *J* = 8.2 Hz, 1H), 7.18 – 7.13 (m, 2H), 6.93 – 6.88 (m, 2H), 6.71 (dd, *J* = 8.1, 2.4 Hz, 1H), 6.34 (d, *J* = 2.1 Hz, 1H), 4.28 (d, *J* = 4.5 Hz, 1H), 4.10 – 4.04 (m, 2H), 3.96 (t, *J* = 8.3 Hz, 1H), 3.85 – 3.81 (m 1H), 3.83 (s, 3H), 3.69 (s, 3H), 3.47 (dd, *J* = 8.8, 6.3 Hz, 1H), 3.22 (s, 3H), 2.90 – 2.77 (m, 2H).
^{13}C NMR (101 MHz, $CDCl_3$) δ 159.7, 158.5, 143.5, 133.7, 130.2, 129.3, 128.9, 114.5, 114.2, 109.8, 78.8, 74.2, 69.8, 56.4, 55.4, 55.3, 46.2, 45.5, 43.8.
IR (ν_{max}, cm^{-1}) 2931 (m), 2360 (s), 2337 (s), 2156 (m), 1799 (m), 1610 (s), 1512 (s), 1250 (s), 1034 (s), 810 (s), 717 (s).
HRMS (ESI/QTOF) m/z: $[M + Na]^+$ Calcd for $C_{21}H_{24}NaO_4^+$ 363.1567; Found 363.1571.

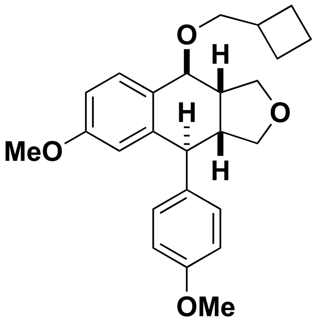

(3a*R*,4*S*,9*S*,9a*S*)-9-(cyclobutylmethoxy)-6-methoxy-4-(4-methoxyphenyl)-1,3,3a,4,9,9a-hexahydronaphtho[2,3-c]furan (**20b-A**)
41% yield, R_f = 0.7 (DCM), colorless oil.
^{1}H NMR (400 MHz, $CDCl_3$) δ 7.36 (d, *J* = 8.3 Hz, 1H), 7.16 (d, *J* = 8.6 Hz, 2H), 6.93 (d, *J* = 8.7 Hz, 2H), 6.77 (dd, *J* = 8.3, 2.5 Hz, 1H), 6.19 (d, *J* = 1.8 Hz, 1H), 4.27 (d, *J* = 9.9 Hz, 1H), 4.04 (dd, *J* = 9.2, 3.8 Hz, 1H), 3.94 (dd, *J* = 9.2, 6.9 Hz, 1H), 3.87-3.81 (m, 1H), 3.84 (s, 3H), 3.71 – 3.60 (m, 4H), 3.66 (s, 3H), 2.79 – 2.67 (m, 2H), 2.45 (tdd, *J* = 10.4, 6.8, 3.9 Hz, 1H), 2.16 (ddt, *J* = 11.1, 7.3, 3.0 Hz, 2H), 2.06 – 1.75 (m, 4H).

^{13}C NMR (101 MHz, $CDCl_3$) δ 158.8, 158.7, 142.5, 132.7, 131.6, 130.5, 123.8, 114.4, 113.3, 110.4, 79.0, 76.8, 73.8, 73.1, 55.4, 55.3, 47.3, 47.0, 45.4, 35.6, 25.4, 25.4, 18.9.
IR (ν_{max}, cm^{-1}) 2933 (m), 2850 (m), 2360 (m), 1610 (s), 1581 (m), 1512 (s), 1306 (m), 1275 (s), 1248 (s), 1178 (m), 1113 (s), 1038 (s), 928 (m), 829 (s).
HRMS (ESI/QTOF) m/z: $[M + Na]^+$ Calcd for $C_{25}H_{30}NaO_4^+$ 417.2036; Found 417.2025.

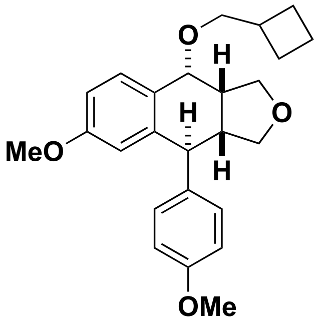

(3a*R*,4*S*,9*R*,9a*S*)-9-(cyclobutylmethoxy)-6-methoxy-4-(4-methoxyphenyl)-1,3,3a,4,9,9a-hexahydronaphtho[2,3-c]furan (**20b-B**)
21% yield, R_f = 0.75 (DCM), colorless oil.
^{1}H NMR (400 MHz, $CDCl_3$) δ 7.21 (d, *J* = 8.2 Hz, 1H), 7.15 – 7.12 (m, 2H), 6.91 – 6.88 (m, 2H), 6.70 (dd, *J* = 8.1, 2.6 Hz, 1H), 6.35 (d, *J* = 2.2 Hz, 1H), 4.36 (d, *J* = 4.4 Hz, 1H), 4.10 (d, *J* = 8.1 Hz, 1H), 4.02 (d, *J* = 7.1 Hz, 2H), 3.92 – 3.88 (m, 1H), 3.82 (s, 3H), 3.70 (s, 3H), 3.39 (t, *J* = 8.1 Hz, 1H), 3.30 (dd, *J* = 9.3, 6.1 Hz, 1H), 3.21 (dd, *J* = 9.3, 6.5 Hz, 1H), 2.91-2.76 (m, 2H), 2.58-2.47 (m, 1H), 2.04 – 1.96 (m, 2H), 1.90 – 1.79 (m, 2H), 1.76 – 1.67 (m, 2H).
^{13}C NMR (101 MHz, $CDCl_3$) δ 159.6, 158.5, 143.3, 133.9, 130.2, 130.0, 128.5, 114.4, 114.2, 109.8, 76.6, 74.6, 72.6, 69.6, 55.4, 55.3, 46.0, 45.4, 43.9, 35.4, 25.1, 25.1, 18.8.
IR (ν_{max}, cm^{-1}) 2933 (m), 2852 (m), 2359 (m), 2274 (m), 2154 (m), 1610 (s), 1581 (m), 1512 (s), 1462 (s), 1250 (s), 1178 (s), 1115 (s), 1038 (s), 933 (s), 823 (s), 758 (s), 733 (s).
HRMS (ESI/QTOF) m/z: $[M + Na]^+$ Calcd for $C_{25}H_{30}NaO_4^+$ 417.2036; Found 417.2041.

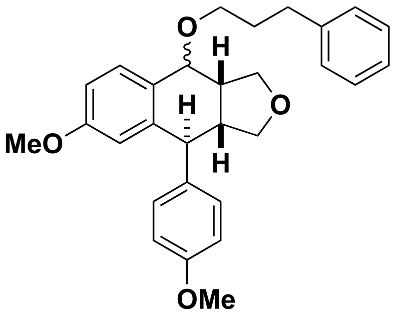

(3a*R*,4*S*,9a*S*)-6-methoxy-4-(4-methoxyphenyl)-9-(3-phenylpropoxy)-1,3,3a,4,9,9a-hexahydronaphtho[2,3-c]furan (**20c, A+B**)
52% yield, 1.7:1 dr, R_f = 0.45 (DCM/EtOAc = 50:1), colorless oil.
^{1}H NMR (400 MHz, $CDCl_3$) (2 isomers in a ratio of about 1.5 : 1) 7.38-7.11 (m, 20H), 6.94 (d, *J* = 8.6 Hz, 3H), 6.91 (d, *J* = 8.7 Hz, 2H), 6.78 (dd, *J* = 8.3, 2.5 Hz, 1.5H), 6.71 (dd, *J* = 8.2, 2.6 Hz, 1H), 6.38 (d, *J* = 2.3 Hz, 1H), 6.21 (d, *J* = 2.1 Hz, 1.5H), 4.37 (d, *J* = 4.5 Hz, 1H), 4.28 (d, *J* = 9.7 Hz, 1.5H), 4.14 (d, *J* = 8.3 Hz, 1H), 4.06-3.80 (m, 7.5H), 3.73-3.61 (m, 6H), 3.84 (s, 4.5H, OMe, major), 3.82 (s, 3H, OMe, minor), 3.71 (s, 3H, OMe, minor), 3.67 (s, 4.5 H, OMe, major), 3.46-3.42 (m, 1H), 3.38-3.27 (m, 2H), 2.91-2.61 (m, 8.5H), 2.54-2.45 (m, 1.5H), 2.13-2.04 (m, 3H), 1.93-1.77 (m, 2H).
^{13}C NMR (101 MHz, $CDCl_3$) δ 159.6, 158.8, 158.7, 158.5, 143.3, 142.5, 142.1, 141.9, 133.8, 132.7, 131.3, 130.5, 130.1, 130.0, 128.6, 128.6, 128.5 (2C), 128.4, 126.0, 125.8, 123.9, 114.4, 114.4, 114.2, 113.4, 110.4, 109.9, 79.2, 76.8, 74.5, 73.7, 73.2, 71.2, 69.6, 67.4, 55.4, 55.4, 55.3, 55.3, 47.2, 47.0, 46.1, 45.4, 45.4, 43.8, 32.6 (2C), 31.9, 31.6.
IR (ν_{max}, cm^{-1}) 2937 (s), 2854 (m), 2359 (s), 1610 (s), 1581 (m), 1512 (s), 1491 (s), 1460 (s), 1275 (s), 1248 (s), 1180 (s), 1109 (s), 1038 (s), 930 (m), 827 (s), 744 (m), 698 (m).
HRMS (ESI/QTOF) m/z: $[M + Na]^+$ Calcd for $C_{29}H_{32}NaO_4^+$ 467.2193; Found 467.2193.

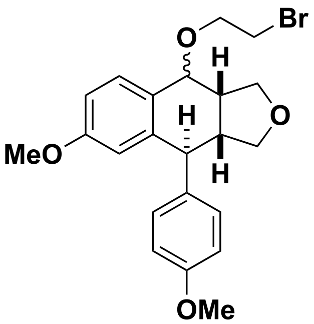

(3a*R*,4*S*,9ai)-9-(2-bromoethoxy)-6-methoxy-4-(4-methoxyphenyl)-1,3,3a,4,9,9a-hexahydronaphtho[2,3-c]furan (**20d, A+B**)

45% yield, 1.6:1 dr, R_f = 0.5 (toluene/EtOAc = 15:1), colorless oil.

^{1}H NMR (400 MHz, $CDCl_3$) (2 isomers in a ratio of about 1.5 : 1) δ 7.41 (d, *J* = 8.3 Hz, 1.5H), 7.19 (d, *J* = 8.2 Hz, 1H), 7.17 – 7.12 (m, 5H), 6.96 – 6.88 (m, 5H), 6.78 (dd, *J* = 8.3, 2.5 Hz, 1.5H), 6.70 (dd, *J* = 8.1, 2.5 Hz, 1H), 6.33 (d, *J* = 2.1 Hz, 1H), 6.21 (d, *J* = 1.9 Hz, 1.5H), 4.48 (d, *J* = 4.3 Hz, 1H), 4.36 (d, *J* = 9.6 Hz, 1.5H), 4.22 – 3.94 (m, 9H), 3.88-3.80 (m, 1H), 3.84 (s, 4.5H, OMe, major), 3.83 (s, 3H, OMe, minor), 3.69 (s, 3H, OMe, minor), 3.67 (s, 4.5H, OMe, major), 3.72-3.55 (m, 9.5H), 3.48-3.44 (m, 1H), 3.43-3.39 (m, 2H), 2.89-2.71 (m, 3.5H), 2.56-2.48 (m, 1.5H).
^{13}C NMR (101 MHz, $CDCl_3$) δ 159.9, 158.9, 158.8, 158.6, 143.9, 142.5, 133.6, 132.7, 130.6, 130.5, 130.2, 129.1, 128.6, 124.0, 114.5, 114.4, 114.3, 113.6, 110.5, 109.9, 79.7, 77.5, 74.4, 73.76, 72.94, 71.6, 69.5, 68.2, 55.4, 55.4, 55.36, 55.3, 47.1, 47.0, 46.0, 45.5, 45.3, 44.0, 31.2, 30.8.
IR (ν_{max}, cm^{-1}) 2927 (m), 2852 (m), 1610 (m), 1581 (m), 1512 (s), 1491 (m), 1462 (m), 1275 (s), 1248 (s), 1178 (m), 1111 (s), 1036 (s), 928 (m), 827 (m), 733 (m)
HRMS (ESI/QTOF) m/z: $[M + Na]^+$ Calcd for $C_{22}H_{25}BrNaO_4^+$ 455.0828; Found 455.0831.

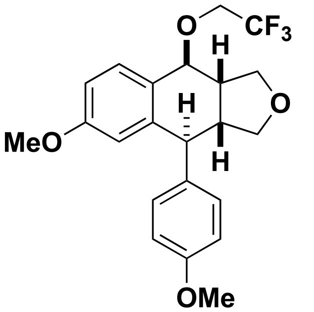

(3aR,4S,9S,9aS)-6-methoxy-4-(4-methoxyphenyl)-9-(2,2,2-trifluoroethoxy)-1,3,3a,4,9,9a-hexahydronaphtho[2,3-c]furan (**20e-A**)

29% yield, R_f = 0.85 (DCM), white solid. M.p = 84–86 °C.
^{1}H NMR (400 MHz, $CDCl_3$) δ 7.29 (d, *J* = 8.3 Hz, 1H), 7.15 (d, *J* = 8.6 Hz, 2H), 6.93 (d, *J* = 8.7 Hz, 2H), 6.79 (dd, *J* = 8.3, 2.5 Hz, 1H), 6.24 (d, *J* = 1.8 Hz, 1H), 4.48 (d, *J* = 9.3 Hz, 1H), 4.21 (dq, *J* = 12.2, 8.7 Hz, 1H), 4.06 – 3.93 (m, 3H), 3.84 (s, 3H), 3.73 – 3.60 (m, 3H), 3.67 (s, 3H), 2.77 (tdd, *J* = 10.6, 6.9, 3.8 Hz, 1H), 2.57 (dtd, *J* = 14.6, 6.8, 4.4 Hz, 1H).
^{19}F NMR (377 MHz, $CDCl_3$) δ -74.19 (t, *J* = 8.5 Hz).
^{13}C NMR (101 MHz, $CDCl_3$) δ 159.2, 158.8, 142.5, 132.6, 130.4, 129.2, 124.0, 124.0 (q, *J* = 279.0 Hz), 114.4, 113.9, 110.7, 81.2, 73.7, 72.5, 68.6 (q, *J* = 34.1 Hz), 55.4, 55.4, 47.0, 46.9, 45.2.
IR (ν_{max}, cm^{-1}) 2933 (m), 2841 (m), 2100 (m), 1610 (m), 1581 (m), 1512 (s), 1462 (m), 1273 (s), 1250 (s), 1159 (s), 1113 (s), 1036 (s), 968 (m), 931 (m), 825 (m), 737 (s).
HRMS (ESI/QTOF) m/z: $[M + Na]^+$ Calcd for $C_{22}H_{23}F_3NaO_4^+$ 431.1441; Found 431.1451.

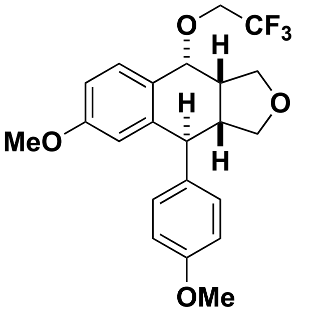

(3a*R*,4*S*,9*R*,9a*S*)-6-methoxy-4-(4-methoxyphenyl)-9-(2,2,2-trifluoroethoxy)-1,3,3a,4,9,9a-hexahydronaphtho[2,3-

c]furan (**20e-B**)
6% yield, R_f = 0.8 (DCM), colorless oil.
^{1}H NMR (400 MHz, $CDCl_3$) δ 7.18 (d, *J* = 8.2 Hz, 1H), 7.15 (d, *J* = 8.6 Hz, 2H), 6.93 – 6.89 (m, 2H), 6.71 (dd, *J* = 8.2, 2.5 Hz, 1H), 6.34 (d, *J* = 2.0 Hz, 1H), 4.60 (d, *J* = 4.0 Hz, 1H), 4.13 (dd, *J* = 8.8, 6.2 Hz, 1H), 4.04 (q, *J* = 8.4 Hz, 2H), 3.87-3.83 (m, 1H), 3.83 (s, 3H), 3.72-3.60 (m, 2H), 3.69 (s, 3H), 3.45 (dd, *J* = 8.6, 7.1 Hz, 1H).
^{19}F NMR (377 MHz, $CDCl_3$) δ -73.52 (t, *J* = 8.7 Hz).
^{13}C NMR (101 MHz, $CDCl_3$) δ 160.2, 158.5, 144.0, 133.1, 130.1, 129.0, 127.5, 124.2 (q, *J* = 280.0 Hz), 114.7, 114.2, 109.8, 78.3, 74.1, 69.1, 64.9 (q, *J* = 33.9 Hz), 55.3, 55.2, 45.9, 45.3, 43.8.
IR (ν_{max}, cm^{-1}) 2360 (m), 2274 (m), 2162 (m), 1612 (s), 1510 (s), 1275 (s), 1252 (s), 1161 (s), 1115 (s), 1036 (s), 1012 (s), 816 (s), 756 (s), 719 (s).
HRMS (ESI/QTOF) m/z: $[M + Na]^+$ Calcd for $C_{22}H_{23}F_3NaO_4^+$ 431.1441; Found 431.1453.

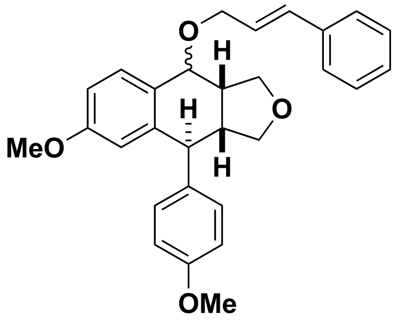

(3a*R*,4*S*,9a*S*)-9-(cinnamyloxy)-6-methoxy-4-(4-methoxyphenyl)-1,3,3a,4,9,9a-hexahydronaphtho[2,3-c]furan (**20f, A+B**)
65% yield, 1.3:1 dr, R_f = 0.45 (toluene/EtOAc = 7:1), colorless oil.
^{1}H NMR (400 MHz, $CDCl_3$) (2 isomers in a ratio of about 2 : 1) 7.45-7.23 (m, 9H), 7.18-7.15 (m, 3H), 6.93 (d, *J* = 8.7 Hz, 2H), 6.89 (d, *J* = 8.7 Hz, 1H), 6.80 (dd, *J* = 8.3, 2.5 Hz, 1H), 6.74-6.69 (m, 1.5H), 6.57 (d, *J* = 16.0 Hz, 0.5H), 6.46 – 6.38 (m, 1.5H), 6.29 – 6.22 (m, 1.5H), 4.58 – 4.50 (m, 1.5H), 4.42 (d, *J* = 9.6 Hz, 1H), 4.37 (ddd, *J* = 12.6, 6.4, 1.1 Hz, 1H), 4.16 – 3.96 (m, 4.5H), 3.91-3.86 (m, 0.5H), 3.84 (s, 3H), 3.82 (s, 1.5H), 3.73-3.61 (m, 3H), 3.71 (s, 1.5H), 3.67 (s, 3H), 3.47 (dd, *J* = 8.6, 7.2 Hz, 0.5H), 2.94– 2.81 (m, 1H), 2.76 (tdd, *J* = 10.8, 6.9, 3.8 Hz, 1H), 2.57-2.50 (m, 1H).
^{13}C NMR (101 MHz, $CDCl_3$) δ 159.7 , 158.8, 158.8, 158.5, 143.5, 142.6, 136.9, 136.7, 133.7, 132.9, 132.8, 132.1, 130.9, 130.5, 130.1, 129.6, 128.7, 128.7, 128.6, 128.0, 127.8, 126.8, 126.7, 126.6, 125.9, 124.1, 114.5, 114.4, 114.2, 113.5, 110.5, 110.0, 78.4, 75.8, 74.3, 73.7, 73.2, 72.2, 69.7, 68.8, 55.4, 55.39, 55.35, 55.3, 47.1, 47.0, 46.10, 45.43, 45.4, 43.8.
IR (ν_{max}, cm^{-1}) 2933 (m), 2854 (m), 2362 (m), 2339 (m), 1610 (s), 1512 (s), 1491 (s), 1460 (m), 1304 (m), 1275 (s), 1248 (s), 1178 (m), 1115 (s), 1036 (s), 966 (m), 831 (m), 729 (m), 692 (m)
HRMS (ESI/QTOF) m/z: $[M + Na]^+$ Calcd for $C_{29}H_{30}NaO_4^+$ 465.2036; Found 465.2049.

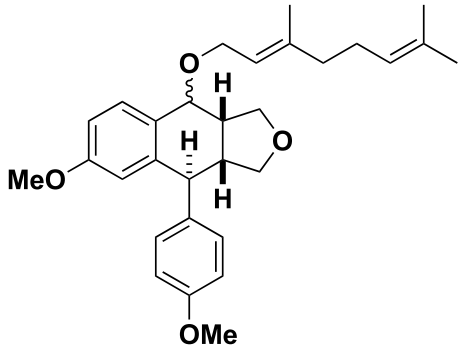

(3a*R*,4*S*,9a*S*)-9-(((E)-3,7-dimethylocta-2,6-dien-1-yl)oxy)-6-methoxy-4-(4-methoxyphenyl)-1,3,3a,4,9,9a-hexahydronaphtho[2,3-c]furan (**20g, A+B**)
50% yield, 1.3:1 dr, R_f = 0.5 (DCM/EtOAc = 20:1), colorless oil.
^{1}H NMR (400 MHz, $CDCl_3$) δ 7.38 (d, *J* = 8.2 Hz, 1H), 7.19 (d, *J* = 8.2 Hz, 1H), 7.18 – 7.13 (m, 4H), 6.95 – 6.91 (m, 2H), 6.91 – 6.86 (m, 2H), 6.78 (dd, *J* = 8.3, 2.5 Hz, 1H), 6.71 (dd, *J* = 8.1, 2.6 Hz, 1H), 6.37 (d, *J* = 2.2 Hz, 1H), 6.20 (d, *J* = 1.8 Hz, 1H), 5.54 (t, *J* = 6.2 Hz, 1H), 5.31 (t, *J* = 6.0 Hz, 1H), 5.16-5.06 (m, 2H), 4.43 (d, *J* = 4.7 Hz, 1H), 4.37 (dd, *J* = 11.6, 6.7 Hz, 1H), 4.33 (d, *J* = 9.9 Hz, 1H), 4.24 (dd, *J* = 11.5, 7.1 Hz, 1H), 4.11 (d, *J* = 8.0 Hz, 1H), 4.05 (dd, *J* = 9.3, 4.0 Hz, 1H), 4.02 – 3.93 (m, 3H), 3.90-3.82 (m, 3H), 3.71-3.61 (m, 3H), 3.84 (s, 3H), 3.82 (s, 3H), 3.70 (s, 3H), 3.66 (s, 3H), 3.42 (dd, *J* = 8.6, 7.2 Hz, 1H), 2.92 – 2.78 (m, 2H), 2.72 (tdd, *J* = 10.8, 6.9, 3.9 Hz, 1H), 2.47 (ddt, *J* = 10.4, 6.8, 3.9 Hz, 1H), 2.20-2.10 (m, 8H), 1.71 (s, 3H), 1.70 (s, 3H), 1.69 (s, 3H), 1.63 (s, 3H), 1.62 (s, 3H), 1.58 (s, 3H).
^{13}C NMR (101 MHz, $CDCl_3$) δ 159.4, 158.6, 158.6, 158.3, 143.2, 142.5, 140.6, 139.5, 133.7, 132.6, 131.8, 131.6, 131.2, 130.4, 129.9, 129.8, 128.3, 124.0, 124.0, 123.8, 121.5, 120.7, 114.3, 114.2, 114.0, 113.3, 110.30, 109.81, 78.22,

75.1, 74.2, 73.6, 73.1, 69.7, 68.2, 64.7, 55.3, 55.3, 55.2, 55.2, 47.0, 46.9, 45.9, 45.3, 45.2, 43.7, 39.7, 39.6, 26.4, 26.4, 25.7, 25.7, 17.7 (2C), 16.7, 16.5.
IR (ν_{max}, cm^{-1}) 2954 (s), 2854 (s), 2360 (s), 1610 (s), 1581 (m), 1512 (s), 1491 (s), 1460 (s), 1248 (s), 1178 (m), 1111 (s), 1066 (s), 1038 (s), 827 (m)
HRMS (ESI/QTOF) m/z: [M + Na]$^+$ Calcd for $C_{30}H_{38}NaO_4^+$ 485.2662; Found 485.2672.

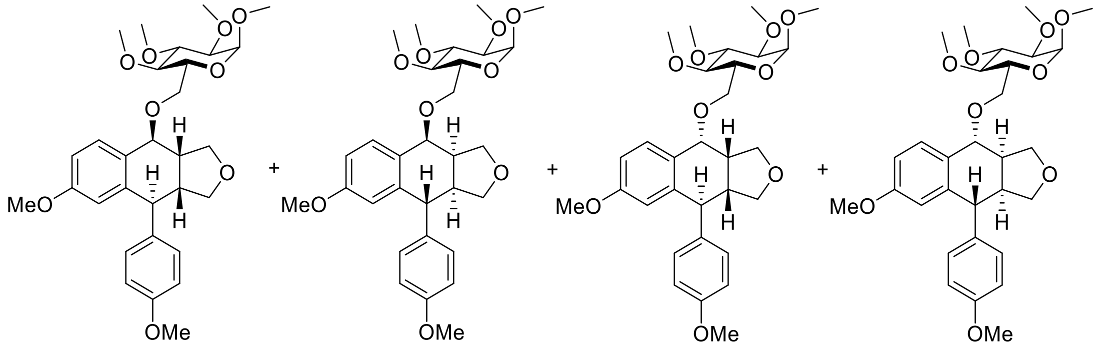

(3a*R*,4*S*,9a*S*)-6-methoxy-4-(4-methoxyphenyl)-9-(((2R,3R,4S,5R,6S)-3,4,5,6-tetramethoxytetrahydro-2H-pyran-2-yl)methoxy)-1,3,3a,4,9,9a-hexahydronaphtho[2,3-c]furan (**20h, A+B**)
43% yield, 1.3:1 dr, R_f = 0.3 (PE/EtOAc = 2:1), colorless oil.
^{1}H NMR (400 MHz, $CDCl_3$) (4 isomers in a ratio of about 1 : 1 : 0.7 : 0.7) δ 7.49 (d, *J* = 8.3 Hz, 1H, major), 7.45 (d, *J* = 8.3 Hz, 1H, major), 7.27 – 7.23 (m, 1.4H, minor), 7.15 (d, *J* = 8.6 Hz, 4H, major), 7.122 (d, *J* = 8.7 Hz, 1.4H, minor), 7.117 (d, *J* = 8.7 Hz, 1.4H, minor), 6.92 (d, *J* = 8.6 Hz, 4H, major), 6.89 (d, *J* = 8.7 Hz, 1.4H, minor), 6.88 (d, *J* = 8.7 Hz, 1.4H, minor), 6.78 (dd, *J* = 8.3, 2.2 Hz, 1H, major), 6.77 (dd, *J* = 8.3, 2.2 Hz, 1H, major), 6.72 (dd, *J* = 8.2, 2.6 Hz, 0.7H, minor), 6.70 (dd, *J* = 8.2, 2.6 Hz, 0.7H, minor), 6.40 (d, *J* = 2.3 Hz, 0.7H, minor), 6.35 (d, *J* = 2.2 Hz, 0.7H, minor), 6.19-6.18 (m, 2H, major), 4.90 (d, *J* = 3.6 Hz, 1H, major), 4.88 (d, *J* = 3.6 Hz, 1H, major), 4.79 (d, *J* = 3.6 Hz, 0.7H, minor), 4.76 (d, *J* = 3.6 Hz, 0.7H, minor), 4.48 (d, *J* = 4.2 Hz, 0.7H, minor), 4.47 (d, *J* = 4.2 Hz, 0.7H, minor), 4.40 (d, *J* = 9.6 Hz, 1H, major)), 4.34 (d, *J* = 9.7 Hz, 1H, major), 4.12 – 3.32 (m, 30.6H, 3CH + 3CH_2), 3.83, 3.82, 3.81, 3.71, 3.69, 3.65, 3.65, 3.62, 3.61, 3.61, 3.58, 3.54, 3.54, 3.52, 3.52, 3.50, 3.46, 3.44, 3.41, 3.41, 3.36 (21 s, OMe, 61.2 H), 3.32 – 3.08 (m, 6.8H, 2CH), 2.93-2.80 (m, 2.8H, minor), 2.76-2.68 (m, 2H, major), 2.56-2.47 (m, 2H, major).
^{13}C NMR (101 MHz, $CDCl_3$) δ 159.7, 159.6, 158.8, 158.7, 158.68, 158.5, 158.4, 143.3, 142.9, 142.6, 142.5, 133.8, 133.6, 132.64, 132.63, 131.3, 131.0, 130.5, 129.93, 129.85, 129.64, 129.55, 128.6, 128.4, 124.2, 124.1, 114.50, 114.47, 114.4, 114.2, 114.1, 113.4, 113.3, 110.5, 110.4, 110.1, 109.9, 97.6, 97.53, 97.48, 97.4, 83.9, 83.82, 83.78, 83.76, 82.1, 82.02, 81.98, 81.9, 80.5, 80.0, 79.9, 79.8, 79.7, 79.5, 77.4, 74.54, 74.51, 73.7, 73.6, 73.2, 70.9, 70.6, 70.5, 70.42, 70.39, 70.3, 69.6, 69.5, 67.5, 67.1, 61.03, 60.99, 60.97, 60.7, 60.6, 60.5, 59.2, 59.1, 55.38, 55.36, 55.30, 55.27, 55.2, 55.1, 47.1, 47.0, 46.9, 46.0, 45.8, 45.5, 45.3, 45.1, 43.7, 43.6.
IR (ν_{max}, cm^{-1}) 2360 (m), 2274 (m), 2162 (m), 1612 (s), 1510 (s), 1275 (s), 1252 (s), 1161 (s), 1115 (s), 1036 (s), 1012 (s), 816 (s), 756 (s), 719 (s).
HRMS (ESI/QTOF) m/z: [M + Na]$^+$ Calcd for $C_{30}H_{40}NaO_9^+$ 567.2565; Found 567.2577.

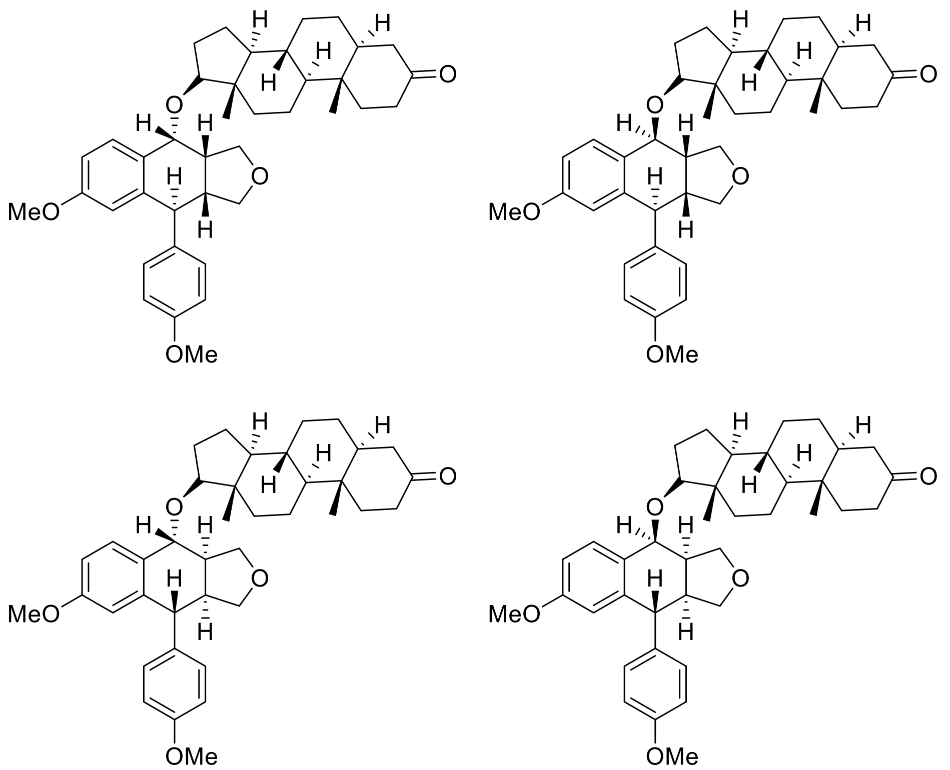

(5*S*,8*R*,9*S*,10*S*,13*S*,14*S*,17*R*)-17-(((3*aS*,9*S*,9*aR*)-7-methoxy-9-(4-methoxyphenyl)-1,3,3a,4,9,9a-hexahydronaphtho[2,3-c]furan-4-yl)oxy)-10,13-dimethylhexadecahydro-3*H*-cyclopenta[a]phenanthren-3-one (**20i, A+B, two isomers, major**)

17% yield, R_f = 0.5 (DCM/EtOAc = 30:1), colorless oil.

(2 isomers in a ratio of approximately 1.2 : 1)

^{1}H NMR (400 MHz, $CDCl_3$) δ 7.44 (d, *J* = 8.3 Hz, 1H), 7.18 – 7.10 (m, 5H), 6.93-6.89 (m, 4H), 6.75 (dd, *J* = 8.3, 2.1 Hz, 1H), 6.67 (dd, *J* = 8.1, 2.3 Hz, 1H), 6.29 (d, *J* = 1.5 Hz, 1H), 6.17 (d, *J* = 1.5 Hz, 1H), 4.42 (d, *J* = 4.0 Hz, 1H), 4.33 (d, *J* = 9.8 Hz, 1H), 4.15 (d, *J* = 8.9 Hz, 1H), 4.09 – 4.02 (m, 3H), 3.99 – 3.95 (m, 1H), 3.89 (t, *J* = 7.8 Hz, 1H), 3.83 (s, 3H), 3.82 (s, 3H), 3.75-3.68 (m, 2H), 3.68 (s, 3H), 3.65 (s, 3H), 3.62-3.56 (m, 2H), 3.39 (t, *J* = 8.4 Hz, 1H), 3.10 (t, *J* = 8.1 Hz, 1H), 2.81 – 2.65 (m, 3H), 2.43 – 1.95 (m, 11H), 1.73 – 0.62 (m, 34 H, overlapped with H_2O and silicone grease), 1.05 (s, 3H), 0.99 (s, 3H), 0.93 (s, 3H), 0.81 (s, 3H).

^{13}C NMR (101 MHz, $CDCl_3$) δ 212.0, 212.0, 159.5, 158.6, 158.4, 143.6, 142.7, 133.7, 132.5, 131.8, 130.4, 130.1, 130.0, 128.3, 123.6, 114.2, 114.1, 113.1, 110.1, 109.4, 87.8, 84.7, 76.4, 74.7, 74.6, 73.6, 73.0, 69.5, 55.3 (2C), 55.2, 55.1, 54.1, 54.0, 50.9, 50.6, 47.2, 46.8, 46.8 (2C), 46.0, 45.4, 45.4, 44.7 (2C), 44.2, 43.4, 42.9, 38.6 (2C), 38.2, 38.2, 37.7, 37.0, 35.8, 35.7, 35.2, 35.1, 31.3, 31.3, 28.8 (2C), 28.3, 27.2, 23.7, 23.5, 21.2, 21.0, 12.2, 11.9, 11.6, 11.5.

IR (ν_{max}, cm^{-1}) 2939 (s), 2916 (s), 2360 (s), 2339 (s), 1712 (s), 1514 (s), 1248 (s), 1115 (s), 1036 (s), 725 (s).

HRMS (ESI/QTOF) m/z: [M + Na]$^+$ Calcd for $C_{39}H_{50}NaO_5^+$ 621.3550; Found 621.3561.

(**20i, A+B, two isomers, minor**)

13% yield, R_f = 0.4 (DCM/EtOAc = 30:1), colorless oil.

^{1}H NMR (400 MHz, $CDCl_3$) δ 7.41 (d, *J* = 8.4 Hz, 1H), 7.21 (d, *J* = 8.2 Hz, 1H), 7.16-7.11 (m, 4H), 6.92 (d, *J* = 8.5 Hz, 2H), 6.88 (d, *J* = 8.5 Hz, 2H), 6.76 (dd, *J* = 8.3, 2.3 Hz, 1H), 6.70 (dd, *J* = 8.1, 2.4 Hz, 1H), 6.35 (d, *J* = 2.0 Hz, 1H), 6.17 (d, *J* = 1.9 Hz, 1H), 4.44-4.42 (m, 2H), 4.14 (dd, *J* = 9.1, 4.8 Hz, 1H), 4.09 – 3.98 (m, 4H), 3.92 (t, *J* = 7.9 Hz, 1H), 3.84 (s, 3H), 3.82 (s, 3H), 3.70 (s, 3H), 3.65 (s, 3H), 3.73 – 3.55 (m, 4H), 3.39-3.31 (m, 2H), 2.85 – 2.67 (m, 3H), 2.50 – 1.91 (m, 11H), 1.85 – 0.68 (m, 34H), 1.04 (s, 3H), 1.02 (s, 3H), 0.95 (s, 3H), 0.82 (s, 3H).

^{13}C NMR (101 MHz, $CDCl_3$) δ 212.1, 212.0, 159.3, 158.6, 158.5, 158.3, 143.0, 142.5, 133.8, 132.4, 132.2, 131.1, 130.3, 129.8, 128.0, 124.0, 114.2, 114.1, 114.0, 113.0, 110.1, 109.9, 89.6, 87.3, 78.4, 76.3, 74.5, 73.6, 73.0, 69.6, 55.3 (2C), 55.2, 55.1, 54.0, 53.9, 50.9, 50.6, 47.7, 46.8, 46.8, 46.7, 45.8, 45.4, 45.1, 44.7, 44.7, 43.9, 43.5, 43.5, 38.6, 38.6, 38.2, 38.2, 37.9, 37.9, 35.8, 35.8, 35.3, 35.2, 31.3, 31.2, 29.7, 28.8 (2C), 28.4, 23.5, 23.2, 21.1 (2C), 12.2, 12.1, 11.5 (2C).

IR (ν_{max}, cm^{-1}) 2929 (s), 2848 (s), 1711 (s), 1610 (s), 1581 (m), 1512 (s), 1250 (s), 1111 (s), 1036 (s), 914 (m), 758 (s), 729 (s).

HRMS (ESI/QTOF) m/z: [M + Na]$^+$ Calcd for $C_{39}H_{50}NaO_5^+$ 621.3550; Found 621.3561.

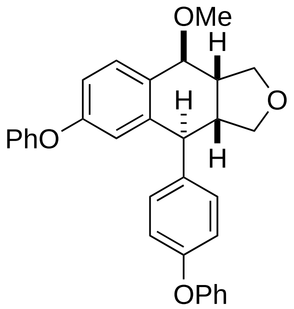

(3a*R*,4*S*,9*S*,9a*S*)-9-methoxy-6-phenoxy-4-(4-phenoxyphenyl)-1,3,3a,4,9,9a-hexahydronaphtho[2,3-c]furan (**20j-A**)
30% yield, R_f = 0.6 (PE/EtOAc = 5:1), colorless oil.
^{1}H NMR (400 MHz, $CDCl_3$) δ 7.40 – 7.33 (m, 3H), 7.31 – 7.27 (m, 2H), 7.19 – 7.15 (m, 2H), 7.14 – 7.10 (m, 1H), 7.08 – 6.98 (m, 5H), 6.95 – 6.91 (m, 2H), 6.85 (dd, *J* = 8.3, 2.4 Hz, 1H), 6.40 – 6.39 (m, 1H), 4.21 (d, *J* = 9.7 Hz, 1H), 4.06 (dd, *J* = 9.3, 3.9 Hz, 1H), 3.98 (dd, *J* = 9.3, 6.8 Hz, 1H), 3.71 (dd, *J* = 9.3, 6.8 Hz, 1H), 3.68 (s, 3H), 3.68 – 3.64 (m, 2H), 2.75 (tdd, *J* = 10.6, 6.7, 3.7 Hz, 1H), 2.48 (tdd, *J* = 10.3, 6.8, 3.9 Hz, 1H).
^{13}C NMR (101 MHz, $CDCl_3$) δ 157.3, 157.1, 156.6, 156.2, 142.6, 135.2, 133.4, 130.7, 129.9, 129.8, 124.2, 123.6, 123.2, 119.3, 119.1, 118.7, 117.7, 116.2, 80.8, 73.7, 73.2, 59.9, 47.1, 47.0, 45.5.
IR (ν_{max}, cm^{-1}) 2931 (w), 2850 (w), 1589 (m), 1504 (m), 1487 (s), 1263 (m), 1234 (s), 1109 (m), 1068 (m), 964 (m), 754 (m), 692 (m).
HRMS (ESI/QTOF) m/z: $[M + Na]^+$ Calcd for $C_{31}H_{28}NaO_4^+$ 487.1880; Found 487.1885.

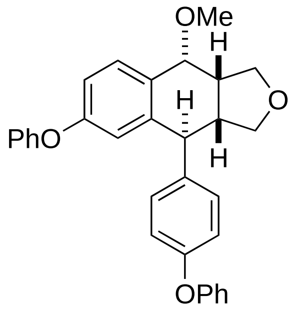

(3a*R*,4*S*,9*R*,9a*S*)-9-methoxy-6-phenoxy-4-(4-phenoxyphenyl)-1,3,3a,4,9,9a-hexahydronaphtho[2,3-c]furan (**20j-B**)
17% yield, R_f = 0.75 (PE/EtOAc = 5:1), colorless oil.
^{1}H NMR (400 MHz, $CDCl_3$) δ 7.37 – 7.29 (m, 4H), 7.23 (d, *J* = 8.1 Hz, 1H), 7.17 (d, *J* = 8.6 Hz, 2H), 7.13-7.06 (m, 2H), 7.04 – 7.00 (m, 2H), 6.97 (d, *J* = 8.6 Hz, 4H), 6.78 (dd, *J* = 8.1, 2.4 Hz, 1H), 6.53 (d, *J* = 2.0 Hz, 1H), 4.31 (d, *J* = 4.3 Hz, 1H), 4.11 (d, *J* = 8.1 Hz, 1H), 4.06 (dd, *J* = 8.8, 5.7 Hz, 1H), 3.99 (t, *J* = 8.2 Hz, 1H), 3.90 – 3.83 (m, 1H), 3.48 (dd, *J* = 8.8, 6.2 Hz, 1H), 3.25 (s, 3H), 2.95 – 2.80 (m, 2H).
^{13}C NMR (101 MHz, $CDCl_3$) δ 157.4, 157.2, 157.0, 156.3, 143.4, 136.2, 131.9, 130.4, 129.9, 129.8, 129.0, 123.5, 119.2, 119.1, 119.0, 118.5, 115.4, 78.7, 74.2, 69.7, 56.6, 46.2, 45.4, 43.6.
IR (ν_{max}, cm^{-1}) 2920 (m), 2852 (m), 2528 (m), 2359 (m), 2158 (m), 1589 (s), 1489 (s), 1240 (s), 1012 (s), 756 (s).
HRMS (ESI/QTOF) m/z: $[M + Na]^+$ Calcd for $C_{31}H_{28}NaO_4^+$ 487.1880; Found 487.1884.

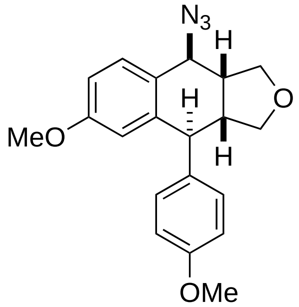

(3a*R*,4*S*,9*S*,9a*R*)-9-azido-6-methoxy-4-(4-methoxyphenyl)-1,3,3a,4,9,9a-hexahydronaphtho[2,3-c]furan (**20k-A**)
29% yield, R_f = 0.6 (DCM/PE = 5:1), white solid.
^{1}H NMR (400 MHz, $CDCl_3$) δ 7.40 (dd, *J* = 8.4, 0.9 Hz, 1H), 7.18 – 7.13 (m, 2H), 6.96 – 6.91 (m, 2H), 6.80 (dd, *J* = 8.4, 2.5 Hz, 1H), 6.23 (d, *J* = 1.7 Hz, 1H), 4.41 (d, *J* = 9.8 Hz, 1H), 4.08 – 3.99 (m, 2H), 3.84 (s, 3H), 3.73 (dd, *J* = 9.3, 6.8 Hz, 1H), 3.67 (s, 3H), 3.67-3.62 (m, 2H), 2.77 (tdd, *J* = 10.8, 6.8, 3.4 Hz, 1H), 2.58-2.47 (m, 1H).
^{13}C NMR (101 MHz, $CDCl_3$) δ 159.3, 158.9, 142.3, 132.5, 130.5, 128.3, 125.1, 114.5, 114.0, 110.8, 73.8, 73.0, 62.8, 55.4, 55.4, 47.1, 46.4, 45.4.
IR (ν_{max}, cm^{-1}) 2927 (m), 2854 (m), 2359 (m), 2104 (s), 1612 (m), 1577 (m), 1512 (s), 1487 (s), 1460 (m), 1423 (m), 1308 (s), 1246 (s), 1182 (s), 1107 (m), 1032 (s), 926 (s), 806 (s).

HRMS (ESI/QTOF) m/z: $[M + Na]^+$ Calcd for $C_{20}H_{21}N_3NaO_3^+$ 374.1475; Found 374.1474.

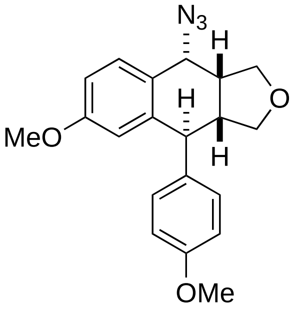

(3a*R*,4*S*,9*R*,9a*R*)-9-azido-6-methoxy-4-(4-methoxyphenyl)-1,3,3a,4,9,9a-hexahydronaphtho[2,3-c]furan (**20k-B**)
16% yield, R_f = 0.55 (DCM/PE = 5:1), colorless oil.
^{1}H NMR (400 MHz, $CDCl_3$) δ 7.24 (d, *J* = 8.2 Hz, 1H), 7.16-7.13 (m, 2H), 6.94 – 6.88 (m, 2H), 6.75 (dd, *J* = 8.2, 2.6 Hz, 1H), 6.39 (d, *J* = 2.2 Hz, 1H), 4.76 (d, *J* = 5.2 Hz, 1H), 4.02 – 3.96 (m, 2H), 3.92 (dd, *J* = 9.2, 5.6 Hz, 1H), 3.87 – 3.80 (m, 2H), 3.83 (s, 3H), 3.71 (s, 3H), 3.47 (dd, *J* = 9.0, 6.2 Hz, 1H), 2.96 – 2.81 (m, 2H).
^{13}C NMR (101 MHz, $CDCl_3$) δ 160.1, 158.7, 142.8, 133.0, 130.1, 128.5, 127.3, 115.1, 114.4, 110.6, 74.0, 70.4, 62.2, 55.4, 55.4, 46.1, 45.2, 42.9.
IR (ν_{max}, cm^{-1}) 2931 (w), 2848 (w), 2360 (w), 2096 (s), 1608 (m), 1581 (m), 1512 (s), 1462 (m), 1248 (s), 1178 (m), 1155 (m), 1111 (m), 1034 (s), 931 (m), 822 (m).
HRMS (APPI/LTQ-Orbitrap) m/z: $[M + H]^+$ Calcd for $C_{20}H_{22}NO_3^+$ 324.1594; Found 324.1598 ($-N_3$).

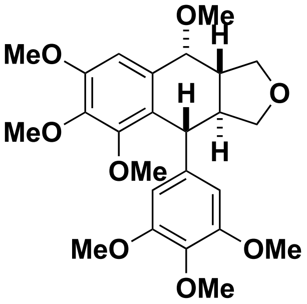

(3a*S*,4*R*,9*R*,9a*S*)-5,6,7,9-tetramethoxy-4-(3,4,5-trimethoxyphenyl)-1,3,3a,4,9,9a-hexahydronaphtho[2,3-c]furan (**21a-A**)
64% yield, 7:1 dr, R_f = 0.45 (PE/EtOAc = 2:1), white solid. M.p = 144– 145 °C.
^{1}H NMR (400 MHz, $CDCl_3$) δ 6.60 (s, 1H), 6.31 (s, 2H), 4.26 (d, *J* = 2.4 Hz, 1H), 4.04 (t, *J* = 7.5 Hz, 1H), 3.96 (t, *J* = 7.4 Hz, 1H), 3.90 (s, 3H), 3.83 (dd, *J* = 6.0, 4.5 Hz, 1H), 3.80 (s, 3H), 3.77 (s, 6H), 3.76 (s, 3H), 3.75 (d, *J* = 8.1 Hz, 1H), 3.56 (dd, *J* = 10.6, 7.5 Hz, 1H), 3.49 (s, 3H), 3.14 (s, 3H), 2.80 – 2.66 (m, 1H), 2.16 (dddd, *J* = 12.9, 10.3, 7.6, 2.4 Hz, 1H).
^{13}C NMR (101 MHz, $CDCl_3$) δ 153.2, 153.0, 152.0, 144.1, 142.9, 136.2, 132.6, 126.9, 109.3, 104.3, 76.5, 72.5, 68.2, 61.1, 60.5, 59.5, 57.6, 56.3, 56.1, 46.5, 46.4, 44.4.
IR (ν_{max}, cm^{-1}) 2935 (w), 1591 (m), 1491 (m), 1456 (m), 1419 (m), 1329 (m), 1236 (m), 1109 (s), 1020 (s), 924 (m), 833 (m).
HRMS (ESI/QTOF) m/z: $[M + Na]^+$ Calcd for $C_{25}H_{32}NaO_8^+$ 483.1989; Found 483.1997.

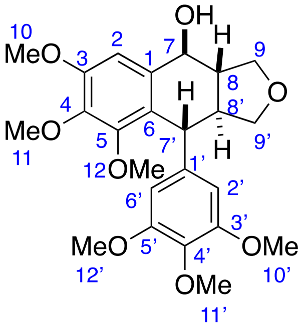

Aglacin E (**4**), 11% yield. Colorless solid.
^{1}H NMR (400 MHz, Chloroform-d) δ 7.04 (s, 1H), 6.23 (s, 2H), 4.75 (d, *J* = 8.7 Hz, 1H), 4.31 (t, *J* = 7.5 Hz, 1H), 3.90 (m, 1H), 3.90 (s, 3H), 3.86 (d, *J* = 10.6 Hz, 1H, partially overlapping. *Measured in C_6D_6*: d, *J* = 10.7 Hz) 3.80 (s, 3H), 3.77 (s, 6H), 3.74 (s, 3H), 3.74 (m, overlapping 1H), 3.66 (dd, *J* = 9.5, 8.0 Hz 1H), 3.14 (s, 3H), 2.21 – 2.08 (m, 2H), 1.87 (brs, 1H).
^{13}C NMR (101 MHz, $CDCl_3$) δ 153.3, 153.0, 151.9, 143.6, 141.8, 137.5, 136.4, 126.0, 104.1, 103.8, 73.4, 72.6, 72.3, 61.1, 60.5, 59.6, 56.4, 56.0, 50.2, 49.9, 47.2.

IR (ν_{max}, cm^{-1}) 1591 (m), 1487 (m), 1456 (m), 1421 (m), 1331 (m), 1236 (m), 1120 (s), 1032 (m), 1009 (m), 916 (w), 758 (m), 731 (w)
HRMS (ESI/QTOF) m/z: $[M + Na]^+$ Calcd for $C_{24}H_{30}NaO_8^+$ 469.1833; Found 469.1838.

	(±)-aglacin E (synthetic)	aglacin E (reported)[7]	
	δ_H (ppm, *J* in Hz)	δ_H (ppm, *J* in Hz)	$\triangle\delta_H$
1			
2	7.04 s	7.05 s	-0.01
3			
4			
5			
6			
7	4.76 d (8.7)	4.76 t (9.0)	0.00
8	2.23 - 2.06 m	2.16 m	-0.01
9α	4.31 t (7.5)	4.32 t (7.4)	-0.01
9β	3.74 m (overlapped)	3.74 t (7.9)	0.00
10	3.90 s	3.91 s	-0.01
11	3.74 s	3.75 s	-0.01
12	3.14 s	3.15 s	-0.01
1'			
2'/6'	6.23 s	6.23 s	0.00
3'/5'			
4'			
7'	3.86 d (10.7)	3.86 d (9.2)	0.00
8'	2.23 - 2.06, m	2.16 m	-0.01
9'α	3.90 m (overlapped)	3.92 t (7.1)	0.00
9'β	3.66 dd (9.5, 8.0)	3.66 dd (9.8, 7.9)	0.00
10'/12'	3.77 s	3.77 s	0.00
11'	3.80 s	3.81 s	-0.01

In our ^1H NMR, $CDCl_3$ was calibrated at 7.26 ppm according to the literature[6].

	(±)-aglacin E (synthetic)	aglacin E (reported)[7]	
	δ_C (ppm)	δ_C (ppm)	$\triangle\delta_C$
1	137.5	137.4	0.1
2	104.1	104.0	0.1
3	153.0	152.9	0.1
4	141.8	141.7	0.1
5	151.9	151.8	0.1
6	126.0	125.9	0.1
7	73.4	73.3	0.1
8	50.2	50.1	0.1
9	72.3	72.1	0.2
10	56.0	55.9	0.1
11	59.6	59.5	0.1
12	61.1	60.9	0.2
1'	143.6	143.4	0.2
2'/6'	103.8	103.7	0.1
3'/5'	153.3	153.2	0.1
4'	136.4	136.3	0.1
7'	47.2	47.1	0.1
8'	49.9	49.8	0.1
9'	72.6	72.4	0.2
10'/12'	56.4	56.2	0.2
11'	60.5	60.4	0.1

In our ^{13}C NMR, $CDCl_3$ was calibrated at 77.16 ppm according to the literature[6].

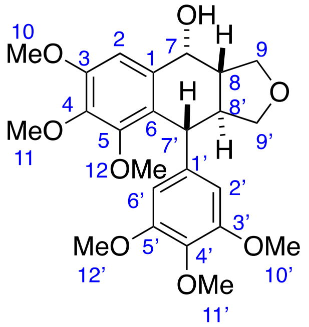

Aglacin F (**5**), 51% yield. Colorless oil.
^{1}H NMR (400 MHz, $CDCl_3$) δ 6.71 (s, 1H), 6.30 (s, 2H), 4.83 (d, *J* = 2.6 Hz, 1H), 4.09 (t, *J* = 7.8 Hz, 1H), 3.94 (t, *J* = 7.5 Hz, 1H), 3.91 (s, 3H), 3.87 (dd, *J* = 10.5, 7.8 Hz, 1H), 3.81 (s, 3H), 3.78 (s, 6H), 3.76 (s, 3H), 3.73 (d, *J* = 8.0 Hz, 1H, partially overlapping. *Measured in C_6D_6*: d, *J* = 10.9 Hz), 3.61 (dd, *J* = 10.4, 7.7 Hz, 1H), 3.15 (s, 3H), 2.66 – 2.56 (m, 1H), 2.21 – 2.13 (m, 1H), 1.85 (brs, 1H).
^{13}C NMR (101 MHz, $CDCl_3$) δ 153.3, 153.0, 152.8, 143.7, 142.9, 136.4, 135.3, 126.2, 108.5, 104.0, 72.6, 68.3, 67.4, 61.1, 60.6, 59.7, 56.4, 56.1, 47.1, 46.3, 43.9.
IR (ν_{max}, cm^{-1}) 2360 (s), 2337 (s), 1593 (m), 1491 (m), 1456 (m), 1421 (m), 1334 (m), 1236 (m), 1117 (s), 1030 (m), 1012 (m), 922 (w), 737 (m), 721 (m).
HRMS (ESI/QTOF) m/z: $[M + Na]^+$ Calcd for $C_{24}H_{30}NaO_8^+$ 469.1833; Found 469.1835.

	(±)-aglacin F (synthetic)	aglacin F (reported)[7]	
	δ_H (ppm, *J* in Hz)	δ_H (ppm, *J* in Hz)	$\Delta\delta_H$
1			
2	6.71 s	6.71 s	0.00
3			
4			
5			
6			
7	4.83 d (2.6)	4.83 d (3.2)	0.00
8	2.21 - 2.13 (m)	2.18 m	-0.01
9α	4.09 t (7.8)	4.09 t (7.7)	0.00
9β	3.87 dd (10.5, 7.8)	3.87 dd (10.5, 7.7)	0.00
10	3.91 s	3.91 s	0.00
11	3.76 s	3.76 s	0.00
12	3.15 s	3.15 s	0.00
1'			
2'/6'	6.30 s	6.30 s	0.00
3'/5'			
4'			
7'	3.73 d (10.9)	3.74 d (9.5)	0.00
8'	2.66 - 2.56 m	2.61 m	0.00
9'α	3.94 t (7.5)	3.94 t (7.6)	0.00
9'β	3.61 dd (10.4, 7.7)	3.61 dd (10.4, 7.6)	0.00
10'/12'	3.78 s	3.78 s	0.00
11'	3.81 s	3.82 s	-0.01

In our ^{1}H NMR, $CDCl_3$ was calibrated at 7.26 ppm according to the literature[6].

	(±)-aglacin F (synthetic)	aglacin F (reported)[7]	
	δ_C (ppm)	δC (ppm)	$\Delta\delta_C$
1	135.3	135.1	0.02
2	108.5	108.3	0.02
3	153.0	152.8	0.02
4	142.9	142.7	0.02
5	152.8	152.7	0.01
6	126.2	126.1	0.01
7	67.4	67.3	0.01
8	46.3	46.1	0.02
9	68.3	68.2	0.01

10	56.1	55.9	0.02
11	60.6	60.4	0.02
12	59.7	59.5	0.02
1'	143.7	143.5	0.02
2'/6'	104.0	103.9	0.01
3'/5'	153.3	153.2	0.01
4'	136.4	136.2	0.02
7'	47.1	46.9	0.02
8'	43.9	43.8	0.01
9'	72.6	72.5	0.01
10'/12'	56.4	56.2	0.02
11'	61.1	60.9	0.02

In our ^{13}C NMR, $CDCl_3$ was calibrated at 77.16 ppm according to the literature[6].

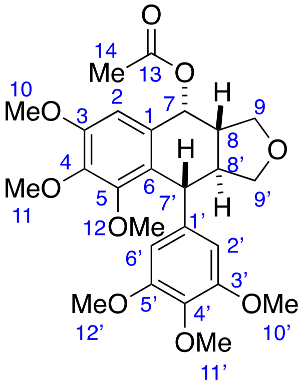

Aglacin A (6)
94% yield from **5**. Colorless oil.
^{1}H NMR (400 MHz, $CDCl_3$) δ 6.77 (s, 1H), 6.32 (s, 2H), 6.11 (d, *J* = 2.6 Hz, 1H), 4.07 (t, *J* = 7.8 Hz, 1H), 3.94 (t, *J* = 7.5 Hz, 1H), 3.87 (s, 3H), 3.82 (s, 3H), 3.79 (s, 6H), 3.77 (d, *J* = 8.0 Hz, 1H, partially overlapping. *Measured in C_6D_6*: d, *J* = 10.7 Hz) 3.76 (s, 3H), 3.59 (dd, *J* = 10.4, 7.8 Hz, 1H), 3.49 (dd, *J* = 10.3, 8.2 Hz, 1H), 3.15 (s, 3H), 2.65 – 2.55 (m, 1H), 2.31 – 2.24 (m, 1H), 2.12 (s, 3H).
^{13}C NMR (101 MHz, $CDCl_3$) δ 171.0, 153.4, 152.8, 152.6, 143.6, 143.2, 136.5, 131.4, 127.0, 109.2, 104.2, 72.4, 68.5, 68.3, 61.1, 60.6, 59.7, 56.4, 56.1, 46.6, 45.0, 44.8, 21.4.
IR (ν_{max}, cm^{-1}) 1734 (m), 1593 (m), 1491 (m), 1458 (m), 1421 (m), 1336 (m), 1234 (s), 1120 (s), 1022 (m), 839 (w).
HRMS (ESI/QTOF) m/z: $[M + Na]^+$ Calcd for $C_{26}H_{32}NaO_9^+$ 511.1939; Found 511.1941.

	(±)-aglacin A (synthetic)	(+)-aglacin A (reported)[8]	
	δ_H (ppm, *J* in Hz)	δ_H (ppm, *J* in Hz)	$\Delta\delta_H$
1			
2	6.77 s	6.77 s	0.00
3			
4			
5			
6			
7α			
7β	6.11 d (2.6)	6.11 d (2.5)	0.00
8	2.31 - 2.24 m	2.25 m	0.02
9α	4.07 t (7.8)	4.06 br t (7.9)	0.01
9β	3.49 dd (10.3, 8.2)	3.48 dd (10.7, 7.9)	0.01
10	3.87 s	3.86 s	0.01
11	3.76 s	3.76 s	0.00
12	3.15 s	3.15 s	0.00
13			
14	2.12 s	2.12 s	0.00
1'			
2'	6.32 s	6.32 s	0.00
3'			
4'			
5'			

6'	6.32 s	6.32 s	0.00
7'	3.77 d (10.7)	3.77 d (8.0)	0.00
8'	2.65 - 2.55 m	2.60 m	0.00
9'α	39.4 t (7.5)	3.94 br t (7.6)	0.00
9'β	3.59 dd (10.4, 7.8)	3.59 dd (10.4, 7.6)	0.00
10'	3.79 s	3.79 s	0.00
11'	3.82 s	3.81 s	0.01
12'	3.79 s	3.79 s	0.00

In our ^{1}H NMR, $CDCl_3$ was calibrated at 7.26 ppm according to the literature[6].

	(±)-aglacin A (synthetic)	(+)-aglacin A (reported)[8]	
	δC (ppm)	δC (ppm)	△δC
1	131.4	131.2	0.02
2	109.2	109.1	0.01
3	152.8	152.6	0.02
4	143.2	143.1	0.01
5	152.6	152.5	0.01
6	127.0	126.8	0.02
7α	68.5	68.3	0.02
7β			
8	44.8	44.6	0.02
9α	68.3	68.1	0.02
9β			
10	56.1	55.9	0.02
11	60.6	60.4	0.02
12	59.7	59.5	0.02
13	171.0	170.8	0.02
14	21.4	21.2	0.02
1'	143.6	143.5	0.01
2'	104.2	104.0	0.02
3'	153.4	153.2	0.02
4'	136.5	136.3	0.02
5'	153.4	153.2	0.02
6'	104.2	104.0	0.02
7'	46.6	46.4	0.02
8'	45	44.8	0.02
9'α	72.4	72.2	0.02
9'β			
10'	56.4	56.2	0.02
11'	61.1	60.9	0.02
12'	56.4	56.2	0.02

In our ^{13}C NMR, $CDCl_3$ was calibrated at 77.16 ppm according to the literature[6].

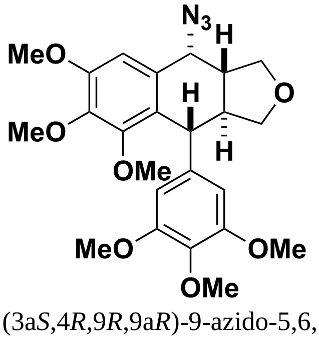

(3a*S*,4*R*,9*R*,9a*R*)-9-azido-5,6,7-trimethoxy-4-(3,4,5-trimethoxyphenyl)-1,3,3a,4,9,9a-hexahydronaphtho[2,3-c]furan (**21b-A**)

87% yield, > 10:1 dr, R_f = 0.4 (PE/EtOAc = 2:1), white solid. M.p = 132– 134 °C.

^{1}H NMR (400 MHz, $CDCl_3$) δ 6.62 (s, 1H), 6.30 (s, 2H), 4.64 (d, *J* = 3.1 Hz, 1H), 4.04 (t, *J* = 7.8 Hz, 1H), 3.97 (t, *J* = 7.5 Hz, 1H), 3.92 (s, 3H), 3.82 – 3.75 (m, 2H), 3.81 (s, 3H), 3.78 (s, 3H), 3.77 (s, 6H), 3.60 (dd, *J* = 10.2, 7.7 Hz, 1H), 3.15 (s, 3H), 2.46 (dtd, *J* = 12.6, 10.4, 7.3 Hz, 1H), 2.27 (dddd, *J* = 12.8, 10.5, 7.6, 3.2 Hz, 1H).

^{13}C NMR (101 MHz, $CDCl_3$) δ 153.4, 153.0, 153.0, 143.3, 143.3, 136.3, 129.7, 126.8, 108.6, 103.7, 72.4, 68.5, 61.1, 60.6, 59.9, 59.7, 56.3, 56.2, 46.4, 45.3 (2C).

IR (ν_{max}, cm^{-1}) 2937 (m), 2362 (m), 2098 (m), 1591 (m), 1491 (m), 1456 (s), 1421 (m), 1334 (s), 1236 (s), 1120 (s), 1026 (m).
HRMS (ESI/QTOF) m/z: $[M + Na]^+$ Calcd for $C_{24}H_{29}N_3NaO_7^+$ 494.1898; Found 494.1908.

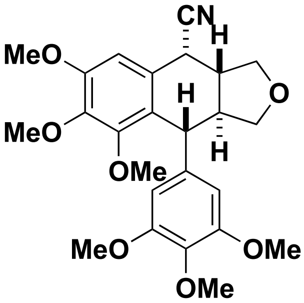

(3a*S*,4*R*,9*R*,9a*S*)-6,7,8-trimethoxy-9-(3,4,5-trimethoxyphenyl)-1,3,3a,4,9,9a-hexahydronaphtho[2,3-c]furan-4-carbonitrile (**21c-A**)
45% yield, > 10:1 dr, R_f = 0.2 (PE/EtOAc = 2:1), white solid. M.p = 202– 203 °C.
^{1}H NMR (400 MHz, $CDCl_3$) δ 6.61 (s, 1H), 6.31 (s, 2H), 4.19 (t, *J* = 7.8 Hz, 1H), 4.06 (d, *J* = 4.7 Hz, 1H), 4.05 (t, *J* = 7.6 Hz, 1H), 3.91 (s, 3H), 3.85 (d, *J* = 10.5 Hz, 1H), 3.83 (dd, *J* = 10.4 Hz, 1H), 3.82 (s, 3H), 3.79 (s, 6H), 3.76 (s, 3H), 3.65 (dd, *J* = 10.0, 7.9 Hz, 1H), 3.15 (s, 3H), 2.60 – 2.46 (m, 1H), 2.31 (dddd, *J* = 12.2, 10.3, 7.5, 4.5 Hz, 1H).
^{13}C NMR (101 MHz, $CDCl_3$) δ 153.5, 153.3, 153.2, 143.0, 142.8, 136.6, 127.2, 126.2, 118.7, 107.7, 104.0, 72.4, 69.8, 61.1, 60.6, 59.8, 56.4, 56.2, 48.4, 46.3, 42.3, 33.62.
IR (ν_{max}, cm^{-1}) 2021 (m), 1593 (m), 1493 (m), 1456 (m), 1336 (m), 1236 (m), 1122 (s), 1028 (m), 908 (m), 719 (s).
HRMS (APPI/LTQ-Orbitrap) m/z: $[M]^+$ Calcd for $C_{25}H_{29}NO_7^+$ 455.1939; Found 455.1951

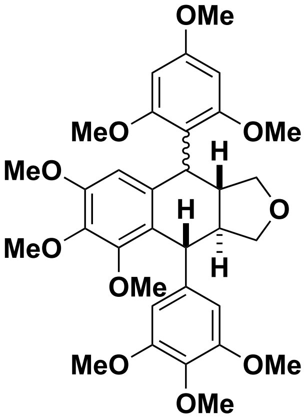

(3a*S*,4*R*,9a*S*)-5,6,7-trimethoxy-9-(2,4,6-trimethoxyphenyl)-4-(3,4,5-trimethoxyphenyl)-1,3,3a,4,9,9a-hexahydronaphtho[2,3-c]furan (**21d (A+B)**)
85% yield, 2.2 : 1 dr, R_f = 0.45 (PE/EtOAc = 2:1), white solid. M.p = 203– 205 °C.
^{1}H NMR (400 MHz, $CDCl_3$) (2 isomers in a ratio of about 2 : 1) 6.54 (s, 2H, minor), 6.35 (s, 1H, minor), 6.33 (s, 4H, major), 6.20 (d, *J* = 2.2 Hz, 3H, major + minor), 6.13 (d, *J* = 2.1 Hz, 1H, minor), 6.11 (s, 2H, major), 6.07 (d, *J* = 2.0 Hz, 2H, major), 4.64 (d, *J* = 6.4 Hz, 1H), 4.59 (d, *J* = 11.1 Hz, 2H), 4.09 (t, *J* = 7.7 Hz, 1H), 3.96 (d, *J* = 10.6 Hz, 2H), 3.92-3.57 (m, 9H), 3.85, 3.83, 3.82, 3.80, 3.79 (five s, 45H, OMe), 3.74 (s, 3H, OMe), 3.72 (s, 3H, OMe), 3.71 (s, 6H, OMe), 3.58 (s, 6H, OMe), 3.53-3.47 (m, 2H), 3.40 (s, 6H, OMe), 3.30 (s, 3H, OMe), 3.17 (s, 3H, OMe), 3.15 (s, 6H, OMe), 3.02-2.95 (m, 1H), 2.86-2.75 (m, 2H), 2.49-2.34 (m, 2H), 2.27-2.30 (m, 2H).
^{13}C NMR (101 MHz, $CDCl_3$) δ 160.6, 160.0, 159.6, 159.2, 153.1, 153.0, 151.9, 151.8, 144.7, 144.1, 140.2, 140.1, 138.7, 138.3, 136.1, 136.0, 126.3, 125.5, 112.2, 111.8, 107.1, 105.3, 104.7, 103.8, 94.8, 92.1, 91.7, 90.5, 73.2, 72.8, 72.7, 71.4, 61.1, 60.6, 59.6, 57.9, 56.5, 56.4, 56.16, 56.12, 55.9, 55.6, 55.43, 55.39, 52.8, 49.1, 47.5, 46.0, 45.3, 44.8, 39.3, 37.0.
IR (ν_{max}, cm^{-1}) 2360 (s), 2156 (s), 2023 (m), 1770 (m), 1516 (s), 966 (s), 918 (s), 845 (s), 717 (s).
HRMS (ESI/QTOF) m/z: $[M + Na]^+$ Calcd for $C_{33}H_{40}NaO_{10}^+$ 619.2514; Found 619.2522.

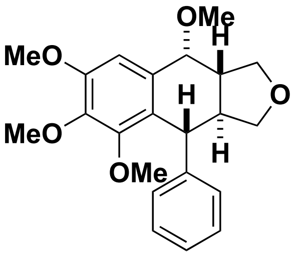

(3a*S*,4*R*,9*R*,9a*S*)-5,6,7,9-tetramethoxy-4-phenyl-1,3,3a,4,9,9a-hexahydronaphtho[2,3-c]furan (**21e-A**)
48% yield, 5 : 1 dr, R_f = 0.25 (DCM/EtOAc = 20:1), colorless oil.
^{1}H NMR (400 MHz, $CDCl_3$) δ 7.26 – 7.22 (m, 2H), 7.16 – 7.12 (m, 1H), 7.10 – 7.06 (m, 2H), 6.62 (s, 1H), 4.27 (d, *J* = 2.5 Hz, 1H), 4.05 (t, *J* = 7.5 Hz, 1H), 3.91 (t, *J* = 7.4 Hz, 1H), 3.90 (s, 3H), 3.83 (dd, *J* = 10.6, 7.6 Hz, 1H), 3.82 (d, *J* = 10.4 Hz, 1H), 3.76 (s, 3H), 3.57 (dd, *J* = 10.6, 7.5 Hz, 1H), 3.52 (s, 3H), 3.00 (s, 3H), 2.80 – 2.67 (m, 1H), 2.19 (dddd, *J* = 12.9, 10.3, 7.6, 2.5 Hz, 1H).
^{13}C NMR (101 MHz, $CDCl_3$) δ 152.9, 152.0, 148.1, 143.0, 132.7, 128.4, 127.26, 127.25, 125.9, 109.4, 76.6, 72.5, 68.3, 60.5, 59.3, 57.9, 56.2, 46.5, 46.4, 44.6.
IR (ν_{max}, cm^{-1}) 2937 (m), 2389 (w), 1599 (m), 1489 (s), 1454 (m), 1406 (m), 1331 (s), 1113 (s), 1028 (s), 922 (m), 704 (m).
HRMS (ESI/QTOF) m/z: $[M + Na]^+$ Calcd for $C_{22}H_{26}NaO_5^+$ 393.1672; Found 393.1680.

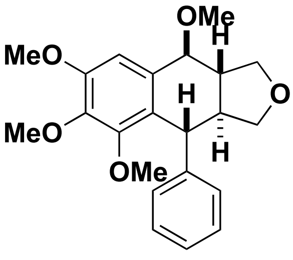

(3a*S*,4*R*,9*S*,9a*S*)-5,6,7,9-tetramethoxy-4-phenyl-1,3,3a,4,9,9a-hexahydronaphtho[2,3-c]furan (**21e-B**)
10% yield, 5 : 1 dr, R_f = 0.2 (DCM/EtOAc = 20:1), colorless oil.
^{1}H NMR (400 MHz, $CDCl_3$) δ 7.27 – 7.23 (m, 2H), 7.18 – 7.12 (m, 1H), 7.04 (d, *J* = 7.1 Hz, 2H), 6.96 (s, 1H), 4.55 (d, *J* = 9.6 Hz, 1H), 4.40 – 4.32 (m, 1H), 3.95 – 3.91 (m, 1H), 3.90 (s, 3H), 3.82 (t, *J* = 7.4 Hz, 1H), 3.78 – 3.74 (m, 1H), 3.73 (s, 3H), 3.63 (dd, *J* = 10.0, 7.8 Hz, 1H), 3.41 (s, 3H), 3.00 (s, 3H), 2.32 – 2.15 (m, 2H).
^{13}C NMR (101 MHz, $CDCl_3$) δ 152.9, 151.9, 147.8, 141.9, 134.9, 128.6, 126.9, 126.1, 104.5, 81.4, 72.3, 71.8, 60.5, 59.3, 56.0, 55.9, 50.4, 46.8, 46.8.
IR (ν_{max}, cm^{-1}) 2362 (s), 2158 (s), 1770 (s), 1518 (s), 1334 (s), 1275 (s), 993 (s), 750 (s), 719 (s).
HRMS (ESI/QTOF) m/z: $[M + Na]^+$ Calcd for $C_{22}H_{26}NaO_5^+$ 393.1672; Found 393.1675.

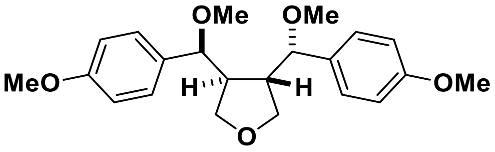

(3*R*,4*R*)-3,4-bis((*S*)-methoxy(4-methoxyphenyl)methyl)tetrahydrofuran (**22a-A**)
76% yield, 10:1 dr, R_f = 0.4 (Petroleum ether/EtOAc = 6:1), white solid, M.p. = 126-127 °C.
^{1}H NMR (400 MHz, $CDCl_3$) δ 6.92 – 6.80 (m, 4H), 6.81 – 6.67 (m, 4H), 3.92 (dd, *J* = 9.0, 4.6 Hz, 2H), 3.82 (dd, *J* = 8.8, 6.6 Hz, 2H), 3.81 (s, 6H), 3.58 (d, *J* = 7.9 Hz, 2H), 3.05 (s, 6H), 2.20 – 2.08 (m, 2H).
^{13}C NMR (101 MHz, $CDCl_3$) δ 159.2, 132.3, 128.3, 113.8, 84.6, 70.6, 56.7, 55.4, 48.9.
IR (ν_{max}, cm^{-1}) 2360 (m), 2158 (m), 2023 (m), 1610 (m), 1512 (s), 1462 (m), 1246 (s), 1174 (m), 1084 (s), 1034 (s), 829 (s).
HRMS (ESI/QTOF) m/z: $[M + Na]^+$ Calcd for $C_{22}H_{28}NaO_5^+$ 395.1829; Found 395.1833.

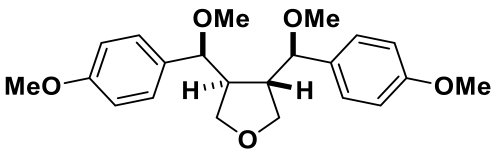

(3*R*,4*R*)-3-((*R*)-methoxy(4-methoxyphenyl)methyl)-4-((*S*)-methoxy(4-methoxyphenyl)methyl)tetrahydrofuran (**22a-B**)

R_f = 0.4 (Petroleum ether/EtOAc = 6:1), colorless oil.
^{1}H NMR (400 MHz, $CDCl_3$) δ 7.15 (d, *J* = 8.6 Hz, 2H), 7.08 (d, *J* = 8.6 Hz, 2H), 6.90 – 6.80 (m, 4H), 4.07 (d, *J* = 6.7 Hz, 1H), 3.93 (dd, *J* = 8.8, 5.3 Hz, 1H), 3.82 (s, 3H), 3.80 (s, 3H), 3.76 (d, *J* = 7.8 Hz, 1H), 3.70 – 3.64 (m, 2H), 3.47 (dd, *J* = 9.0, 5.2 Hz, 1H), 3.18 (s, 3H), 3.08 (s, 3H), 2.55 – 2.50 (m, 1H), 2.36 (tt, *J* = 7.7, 4.9 Hz, 1H).
^{13}C NMR (101 MHz, $CDCl_3$) δ 159.3, 159.1, 133.0, 132.2, 128.7, 128.3, 113.8, 113.8, 85.1, 84.6, 70.2, 70.1, 57.1, 56.7, 55.4, 49.4, 48.6.
IR (ν_{max}, cm^{-1}) 2929 (m), 2160 (m), 2023 (m), 1610 (m), 1510 (s), 1464 (m), 1248 (s), 1176 (m), 1084 (s), 1036 (s), 935 (m), 835 (m), 816 (m), 723 (m), 690 (m)
HRMS (ESI/QTOF) m/z: $[M + Na]^+$ Calcd for $C_{22}H_{28}NaO_5^+$ 395.1829; Found 395.1839.

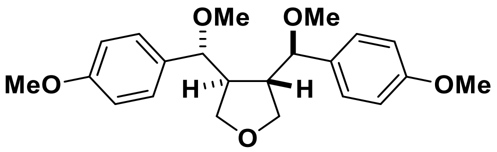

(3*R*,4*R*)-3,4-bis((*R*)-methoxy(4-methoxyphenyl)methyl)tetrahydrofuran (**22a-C, another diastereomer which has not been detected in photocyclization reaction**)
R_f = 0.55 (DCM/EtOAc = 20:1), colorless oil.
^{1}H NMR (400 MHz, $CDCl_3$) δ 7.25 – 7.20 (m, 4H), 6.92 – 6.85 (m, 4H), 4.05 (d, *J* = 8.1 Hz, 2H), 3.81 (s, 6H), 3.56 (dd, *J* = 9.1, 6.9 Hz, 2H), 3.45 (dd, *J* = 9.1, 4.8 Hz, 2H), 3.18 (s, 6H), 2.72 – 2.66 (m, 2H).
^{13}C NMR (101 MHz, $CDCl_3$) δ 159.3, 132.3, 129.1, 113.8, 84.9, 69.6, 56.8, 55.4, 48.7.
IR (ν_{max}, cm^{-1}) 2362 (s), 1612 (s), 1512 (s), 1248 (s), 1082 (s), 1036 (s), 829 (s), 681 (s).
HRMS (ESI/QTOF) m/z: $[M + Na]^+$ Calcd for $C_{22}H_{28}NaO_5^+$ 395.1829; Found 395.1839.

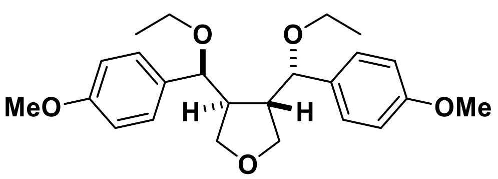

(3*R*,4*R*)-3,4-bis((*S*)-ethoxy(4-methoxyphenyl)methyl)tetrahydrofuran (**22b-A**)
77% yield, 8:1 dr, R_f = 0.6 (DCM/EtOAc = 20:1), pale yellow oil.
^{1}H NMR (400 MHz, $CDCl_3$) δ 6.87 – 6.84 (m, 4H), 6.74 – 6.70 (m, 4H), 3.95 (dd, *J* = 9.0, 4.4 Hz, 2H), 3.82 (dd, *J* = 8.9, 6.5 Hz, 2H), 3.80 (s, 6H), 3.70 (d, *J* = 8.2 Hz, 2H), 3.22 (dq, *J* = 9.3, 7.0 Hz, 2H), 3.12 (dq, *J* = 9.3, 7.0 Hz, 2H), 2.18 – 2.10 (m, 2H), 1.07 (t, *J* = 7.0 Hz, 6H).
^{13}C NMR (101 MHz, $CDCl_3$) δ 159.0, 133.0, 128.3, 113.7, 82.6, 70.7, 64.2, 55.4, 48.7, 15.3.
IR (ν_{max}, cm^{-1}) 2362 (m), 2158 (s), 2021 (m), 1612 (m), 1512 (s), 1489 (m), 1248 (s), 1088 (s), 1036 (s), 829 (s), 717 (s).
HRMS (ESI/QTOF) m/z: $[M + Na]^+$ Calcd for $C_{24}H_{32}NaO_5^+$ 423.2142; Found 423.2146.

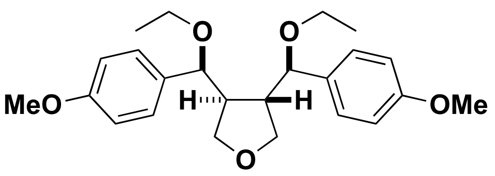

(3*S*,4*S*)-3-((*R*)-ethoxy(4-methoxyphenyl)methyl)-4-((*S*)-ethoxy(4-methoxyphenyl)methyl)tetrahydrofuran (**22b-B**)
R_f = 0.62 (DCM/EtOAc = 20:1), colorless oil.
^{1}H NMR (400 MHz, $CDCl_3$) δ 7.16 (d, *J* = 8.6 Hz, 2H), 7.05 (d, *J* = 8.6 Hz, 2H), 6.84 (d, *J* = 8.7 Hz, 2H), 6.82 (d, *J* = 8.7 Hz, 2H), 4.20 (d, *J* = 7.2 Hz, 1H), 3.95 (dd, *J* = 8.8, 5.2 Hz, 1H), 3.85 (d, *J* = 8.0 Hz, 1H), 3.82 (s, 3H), 3.80 (s, 3H), 3.75 – 3.62 (m, 2H), 3.46 (dd, *J* = 9.0, 5.1 Hz, 1H), 3.41 – 3.32 (m, 1H), 3.32 – 3.18 (m, 2H), 3.18 – 3.07 (m, 1H), 2.62 – 2.52 (m, 1H), 2.30 (ddd, *J* = 12.3, 7.9, 4.8 Hz, 1H), 1.14 (t, *J* = 7.0 Hz, 3H), 1.09 (t, *J* = 7.0 Hz, 3H).
^{13}C NMR (101 MHz, $CDCl_3$) δ 159.1, 159.0, 133.8, 133.2, 128.5, 128.3, 113.7, 113.7, 83.0, 82.5, 70.4, 70.2, 64.4, 64.0, 55.4, 55.4, 49.4, 48.5, 15.4, 15.4.
IR (ν_{max}, cm^{-1}) 2360 (s), 2021 (s), 1612 (m), 1510 (s), 1248 (s), 1082 (s), 1038 (s), 829 (m).
HRMS (ESI/QTOF) m/z: $[M + Na]^+$ Calcd for $C_{24}H_{32}NaO_5^+$ 423.2142; Found 423.2139.

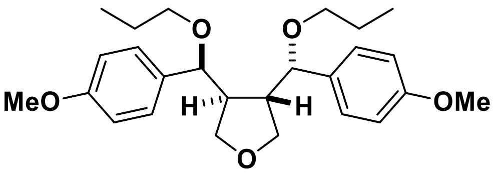

(3*R*,4*R*)-3,4-bis((*S*)-(4-methoxyphenyl)(propoxy)methyl)tetrahydrofuran (**22c-A**)
61% yield, 8:1 dr, R_f = 0.6 (DCM/EtOAc = 20:1), colorless oil.
^{1}H NMR (400 MHz, $CDCl_3$) δ 6.88 – 6.85 (m, 4H), 6.74 – 6.70 (m, 4H), 3.97 (dd, *J* = 9.0, 4.3 Hz, 2H), 3.82 (dd, *J* = 8.9, 6.4 Hz, 2H), 3.81 (s, 6H), 3.68 (d, *J* = 8.1 Hz, 2H), 3.12 (dt, *J* = 9.2, 6.6 Hz, 2H), 3.00 (dt, *J* = 9.1, 6.6 Hz, 2H), 2.18 – 2.12 (m, 2H), 1.53 – 1.40 (m, 4H), 0.84 (t, *J* = 7.4 Hz, 6H).
^{13}C NMR (101 MHz, $CDCl_3$) δ 159.0, 133.1, 128.3, 113.7, 82.8, 70.7, 70.6, 55.3, 48.9, 23.1, 10.9.
IR (ν_{max}, cm^{-1}) 2360 (s), 2021 (s), 1612 (m), 1510 (s), 1248 (s), 1082 (s), 1038 (s), 829 (m).
HRMS (ESI/QTOF) m/z: $[M + Na]^+$ Calcd for $C_{26}H_{36}NaO_5^+$ 451.2455; Found 451.2456.

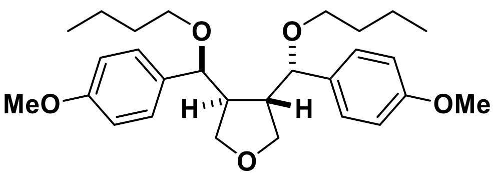

(3*R*,4*R*)-3,4-bis((*S*)-butoxy(4-methoxyphenyl)methyl)tetrahydrofuran (**22d-A**)
72% yield, 11:1 dr, R_f = 0.6 (Petroleum ether/EtOAc = 7:1), pale yellow oil.
^{1}H NMR (400 MHz, $CDCl_3$) δ 6.87 – 6.84 (m, 4H), 6.74 – 6.70 (m, 4H), 3.95 (dd, *J* = 9.0, 4.5 Hz, 2H), 3.82 (dd, *J* = 8.8, 6.5 Hz, 2H), 3.80 (s, 6H), 3.67 (d, *J* = 8.1 Hz, 2H), 3.15 (dt, *J* = 9.3, 6.4 Hz, 2H), 3.04 (dt, *J* = 9.3, 6.5 Hz, 2H), 2.17 – 2.11 (m, 2H), 1.46 – 1.39 (m, 4H), 1.33 – 1.24 (m, 4H), 0.84 (t, *J* = 7.3 Hz, 6H).
^{13}C NMR (101 MHz, $CDCl_3$) δ 159.0, 133.1, 128.3, 113.7, 82.8, 70.7, 68.6, 55.3, 48.9, 32.0, 19.6, 14.0.
IR (ν_{max}, cm^{-1}) 2956 (m), 2868 (m), 2360 (m), 1610 (m), 1512 (s), 1462 (m), 1246 (s), 1173 (m), 1088 (s), 1036 (s), 829 (m).
HRMS (ESI/QTOF) m/z: $[M + Na]^+$ Calcd for $C_{28}H_{40}NaO_5^+$ 479.2768; Found 479.2767.

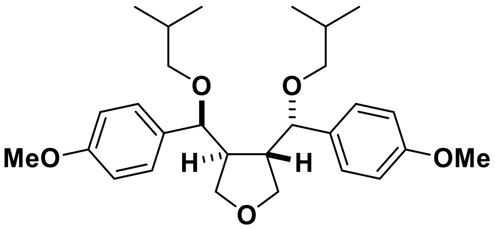

(3*R*,4*R*)-3,4-bis((*S*)-isobutoxy(4-methoxyphenyl)methyl)tetrahydrofuran (**22e-A**)
58% yield, 8:1 dr, R_f = 0.45 (PE/EtOAc = 9:1), colorless oil.
^{1}H NMR (400 MHz, $CDCl_3$) δ 6.89 – 6.83 (m, 4H), 6.75 – 6.70 (m, 4H), 3.98 (dd, *J* = 8.9, 4.6 Hz, 2H), 3.82 (dd, *J* = 9.0, 6.6 Hz, 2H), 3.81 (s, 6H), 3.66 (d, *J* = 7.9 Hz, 2H), 2.93 (dd, *J* = 8.9, 6.4 Hz, 2H), 2.79 (dd, *J* = 8.9, 6.7 Hz, 2H), 2.19 – 2.13 (m, 2H), 1.79 – 1.69 (m, 2H), 0.85 (d, *J* = 6.7 Hz, 6H), 0.80 (d, *J* = 6.7 Hz, 6H).
^{13}C NMR (101 MHz, $CDCl_3$) δ 159.0, 133.1, 128.3, 113.7, 82.9, 75.8, 70.6, 55.3, 49.0, 28.7, 19.7, 19.6.
IR (ν_{max}, cm^{-1}) 2954 (m), 2360 (m), 1612 (m), 1512 (s), 1464 (m), 1246 (s), 1173 (m), 1080 (s), 1036 (s), 827 (s).
HRMS (ESI/QTOF) m/z: $[M + Na]^+$ Calcd for $C_{28}H_{40}NaO_5^+$ 479.2768; Found 479.2775

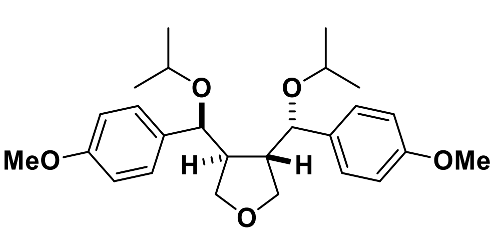

(3*R*,4*R*)-3,4-bis((*S*)-isopropoxy(4-methoxyphenyl)methyl)tetrahydrofuran (**22f-A**)
62% yield, 10:1 dr, R_f = 0.65 (Petroleum ether/EtOAc = 7:1), white solid, M.p. = 117-118 ^oC.
^{1}H NMR (400 MHz, $CDCl_3$) δ 6.89 – 6.84 (m, 4H), 6.74 – 6.69 (m, 4H), 3.93 (dd, *J* = 8.9, 4.4 Hz, 2H), 3.82 – 3.78 (m, 4H), 3.81 (s, 6H), 3.29 (sept, *J* = 6.1 Hz, 2H), 2.12 – 2.06 (m, 2H), 1.06 (d, *J* = 6.0 Hz, 6H), 0.94 (d, *J* = 6.2 Hz, 6H).
^{13}C NMR (101 MHz, $CDCl_3$) δ 158.9, 133.8, 128.3, 113.6, 79.9, 70.7, 68.6, 55.4, 48.9, 23.6, 21.2.
IR (ν_{max}, cm^{-1}) 2970 (m), 2360 (m), 1612 (m), 1510 (s), 1462 (m), 1375 (m), 1246 (s), 1174 (m), 1120 (m), 1038 (s), 939 (m), 831 (m).

HRMS (ESI/QTOF) m/z: [M + Na]$^+$ Calcd for $C_{26}H_{36}NaO_5^+$ 451.2455; Found 451.2456.

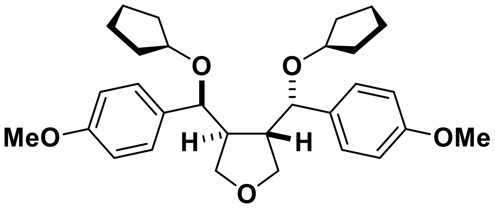

(3*R*,4*R*)-3,4-bis((*S*)-(cyclopentyloxy)(4-methoxyphenyl)methyl)tetrahydrofuran (**22g-A**)
52% yield, 7:1 dr, R_f = 0.45 (DCM), yellow oil.
^{1}H NMR (400 MHz, $CDCl_3$) δ 6.90 – 6.83 (m, 4H), 6.77 – 6.67 (m, 4H), 3.90 (dd, *J* = 8.9, 4.5 Hz, 2H), 3.81 (s, 6H), 3.77 (dd, *J* = 8.9, 6.7 Hz, 2H), 3.76 (d, *J* = 7.8 Hz, 2H), 3.63 – 3.55 (m, 2H), 2.12 – 2.05 (m, 2H), 1.69 – 1.58 (m, 6H), 1.54 – 1.34 (m, 10H).
^{13}C NMR (101 MHz, $CDCl_3$) δ 158.9, 133.6, 128.4, 113.6, 80.3, 78.4, 70.6, 55.3, 48.9, 33.2, 31.3, 23.5.
IR (ν_{max}, cm^{-1}) 2952 (m), 2868 (m), 2362 (m), 1612 (m), 1585 (m), 1510 (s), 1246 (s), 1173 (m), 1036 (s), 829 (s).
HRMS (ESI/QTOF) m/z: [M + Na]$^+$ Calcd for $C_{30}H_{40}NaO_5^+$ 503.2768; Found 503.2765.

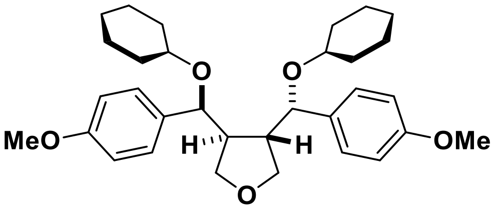

(3*R*,4*R*)-3,4-bis((*S*)-(cyclohexyloxy)(4-methoxyphenyl)methyl)tetrahydrofuran (**22h-A**)
56% yield, 7:1 dr, R_f = 0.5 (DCM), yellow oil.
^{1}H NMR (400 MHz, $CDCl_3$) δ 6.87 (d, *J* = 8.6 Hz, 4H), 6.71 (d, *J* = 8.6 Hz, 4H), 3.95 (dd, *J* = 8.9, 4.4 Hz, 2H), 3.86 (d, *J* = 7.9 Hz, 2H), 3.81 (s, 6H), 3.79 (dd, *J* = 9.4, 6.6 Hz, 2H), 3.00 – 2.94 (m, 2H), 2.13 – 2.07 (m, 2H), 1.87 – 1.78 (m, 2H), 1.69 – 1.56 (m, 4H), 1.53 – 1.40 (m, 4H), 1.28 – 1.04 (m, 10H).
^{13}C NMR (101 MHz, $CDCl_3$) δ 158.9, 134.0, 128.3, 113.6, 79.6, 74.6, 70.8, 55.3, 49.0, 33.7, 31.3, 25.9, 24.3, 24.1.
IR (ν_{max}, cm^{-1}) 2929 (m), 2856 (m), 2362 (m), 1610 (m), 1510 (s), 1450 (m), 1246 (s), 1173 (m), 1072 (s), 1036 (s), 827 (m).
HRMS (ESI/QTOF) m/z: [M + Na]$^+$ Calcd for $C_{32}H_{44}NaO_5^+$ 531.3081; Found 531.3085.

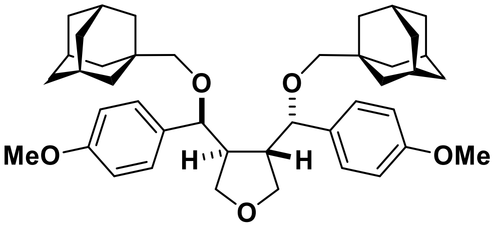

(3*R*,4*R*)-3-((1*S*)-(((1*R*,3*R*,5*S*)-adamantan-1-yl)methoxy)(4-methoxyphenyl)methyl)-4-((*S*)-(((3*S*,5*S*,7*S*)-adamantan-1-yl)methoxy)(4-methoxyphenyl)methyl)tetrahydrofuran (**22i-A**)
28% yield, 10:1 dr, R_f = 0.55 (PE/EtOAc = 20:1), white solid. M.p. = 236-237 °C.
^{1}H NMR (400 MHz, $CDCl_3$) δ 6.86 (d, *J* = 8.6 Hz, 4H), 6.73 (d, *J* = 8.6 Hz, 4H), 3.97 (dd, *J* = 8.8, 4.9 Hz, 2H), 3.81 (s, 6H), 3.79 (dd, *J* = 8.8, 6.7 Hz, 2H), 3.59 (d, *J* = 7.4 Hz, 2H), 2.70 (d, *J* = 8.7 Hz, 2H), 2.59 (d, *J* = 8.7 Hz, 2H), 2.19 – 2.13 (m, 2H), 1.97 – 1.88 (m, 6H), 1.69 (d, *J* = 12.0 Hz, 6H), 1.61 (d, *J* = 11.7 Hz, 6H), 1.50 – 1.43 (m, 12H).
^{13}C NMR (101 MHz, $CDCl_3$) δ 158.9, 133.5, 128.2, 113.6, 82.7, 79.7, 70.6, 55.3, 49.2, 39.9, 37.4, 34.2, 28.4.
IR (ν_{max}, cm^{-1}) 2900 (s), 2360 (s), 1512 (m), 1246 (s), 1080 (s), 677 (m).
HRMS (ESI/QTOF) m/z: [M + Na]$^+$ Calcd for $C_{42}H_{56}NaO_5^+$ 663.4020; Found 663.4023.

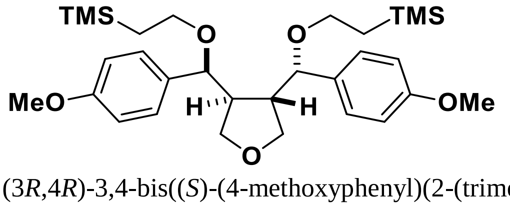

(3*R*,4*R*)-3,4-bis((*S*)-(4-methoxyphenyl)(2-(trimethylsilyl)ethoxy)methyl)tetrahydrofuran (**22j-A**)
50% yield, 12:1 dr, R_f = 0.8 (toluene/EtOAc = 30:1), yellow oil.

^{1}H NMR (400 MHz, $CDCl_3$) δ 6.89 – 6.84 (m, 4H), 6.77 – 6.70 (m, 4H), 3.94 (dd, *J* = 8.9, 4.7 Hz, 2H), 3.82 – 3.79 (m, 2H), 3.81 (s, 6H), 3.67 (d, *J* = 7.8 Hz, 2H), 3.26 (ddd, *J* = 10.4, 9.4, 5.9 Hz, 2H), 3.11 (ddd, *J* = 10.3, 9.4, 5.9 Hz, 2H), 2.18 – 2.09 (m, 2H), 0.87 (ddd, *J* = 13.9, 10.4, 5.9 Hz , 2H), 0.77 (ddd, *J* = 13.9, 10.4, 5.9 Hz, 2H), -0.07 (s, 18H).
^{13}C NMR (101 MHz, $CDCl_3$) δ 159.0, 133.3, 128.2, 113.7, 82.3, 70.8, 66.0, 55.3, 48.9, 18.4, -1.2.
IR (ν_{max}, cm^{-1}) 2360 (w), 1610 (m), 1510 (m), 1246 (s), 1173 (m), 1072 (s), 1038 (m), 831 (s).
HRMS (ESI/QTOF) m/z: $[M + Na]^+$ Calcd for $C_{30}H_{48}NaO_5Si_2^+$ 567.2932; Found 567.2948.

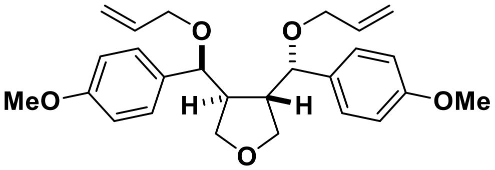

(3*R*,4*R*)-3,4-bis((*S*)-(allyloxy)(4-methoxyphenyl)methyl)tetrahydrofuran (**22k-A**)
54% yield, 7:1 dr, R_f = 0.6 (PE/EtOAc = 9:1), colorless oil.
^{1}H NMR (400 MHz, $CDCl_3$) δ 6.90 – 6.83 (m, 4H), 6.76 – 6.70 (m, 4H), 5.80 (dddd, *J* = 16.7, 10.5, 6.1, 5.3 Hz, 2H), 5.16 – 5.08 (m, 4H), 3.97 (dd, *J* = 9.0, 4.5 Hz, 2H), 3.85 – 3.71 (m, 2H), 3.81 (s, 6H), 3.78 (d, *J* = 8.1 Hz, 2H), 3.74 (ddt, *J* = 12.6, 5.2, 1.4 Hz, 2H), 3.56 (ddt, *J* = 12.6, 6.2, 1.2 Hz, 2H), 2.22 – 2.15 (m, 2H).
^{13}C NMR (101 MHz, $CDCl_3$) δ 159.2, 134.9, 132.5, 128.4, 117.0, 113.8, 82.1, 70.7, 69.5, 55.4, 48.8.
IR (ν_{max}, cm^{-1}) 1610 (m), 1512 (s), 1462 (m), 1246 (s), 1173 (m), 1066 (s), 1034 (s), 926 (m), 829 (s).
HRMS (ESI/QTOF) m/z: $[M + Na]^+$ Calcd for $C_{26}H_{32}NaO_5^+$ 447.2142; Found 447.2148.

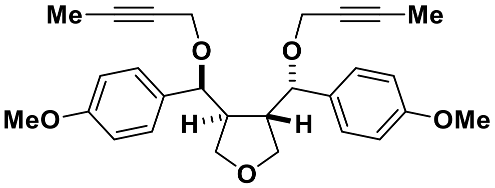

(3*R*,4*R*)-3,4-bis((*S*)-(but-2-yn-1-yloxy)(4-methoxyphenyl)methyl)tetrahydrofuran (**22l-A**)
23% yield, R_f = 0.5 (toluene/EtOAc = 50:1), colorless oil.
^{1}H NMR (400 MHz, $CDCl_3$) δ 6.85 (d, *J* = 8.6 Hz, 4H), 6.72 (d, *J* = 8.6 Hz, 4H), 4.01 – 3.94 (m, 4H), 3.93 – 3.83 (m, 4H), 3.81 (s, 6H), 3.68 – 3.61 (m, 2H), 2.24 – 2.13 (m, 2H), 1.84 (t, *J* = 2.3 Hz, 6H).
^{13}C NMR (101 MHz, $CDCl_3$) δ 159.3, 131.6, 128.6, 113.8, 82.1, 81.5, 75.4, 70.7, 56.3, 55.4, 48.5, 3.8.
IR (ν_{max}, cm^{-1}) 1610 (s), 1512 (s), 1344 (s), 1248 (s), 1163 (s), 1099 (s), 1034 (s), 825 (s), 758 (s), 717 (s), 663 (s).
HRMS (ESI/QTOF) m/z: $[M + Na]^+$ Calcd for $C_{28}H_{32}NaO_5^+$ 471.2142; Found 471.2152.

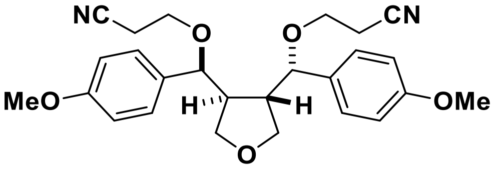

3,3'-(((1*S*,1'*S*)-((3*R*,4*R*)-tetrahydrofuran-3,4-diyl)bis((4-methoxyphenyl)methylene))bis(oxy))dipropanenitrile (**22m**)
24% yield, R_f = 0.5 (toluene/EtOAc = 2:1), colorless oil.
^{1}H NMR (400 MHz, $CDCl_3$) δ 6.86 (d, *J* = 8.7 Hz, 4H), 6.73 (d, *J* = 8.7 Hz, 4H), 3.97 (dd, *J* = 9.2, 4.5 Hz, 2H), 3.84 (dd, *J* = 9.2, 6.4 Hz, 2H), 3.81 (s, 6H), 3.77 (d, *J* = 8.2 Hz, 2H), 3.37 – 3.26 (m, 4H), 2.51 – 2.39 (m, 4H), 2.20 – 2.14 (m, 2H).
^{13}C NMR (101 MHz, $CDCl_3$) δ 159.5, 131.3, 128.3, 117.9, 114.1, 83.5, 70.5, 63.2, 55.4, 48.6, 19.0.
IR (ν_{max}, cm^{-1}) 2360 (s), 2158 (s), 2021 (s), 1612 (s), 1510 (s), 1246 (s), 1032 (s), 831 (s), 760 (s), 717 (s).
HRMS (APPI/LTQ-Orbitrap) m/z: $[M + Na]^+$ Calcd for $C_{26}H_{30}N_2NaO_5^+$ 473.2047; Found 473.2040.

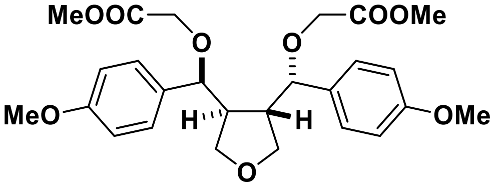

Dimethyl 2,2'-(((1*S*,1'*S*)-((3*R*,4*R*)-tetrahydrofuran-3,4-diyl)bis((4-methoxyphenyl)methylene))bis(oxy))diacetate (**22n**)
21% yield, R_f = 0.35 (toluene/EtOAc = 3:1), colorless oil.

^{1}H NMR (400 MHz, $CDCl_3$) δ 6.86 – 6.80 (m, 4H), 6.71 – 6.67 (m, 4H), 4.15 (dd, *J* = 9.1, 4.2 Hz, 2H), 3.96 (d, *J* = 8.4 Hz, 2H), 3.87 (dd, *J* = 9.0, 6.4 Hz, 2H), 3.83 (d, *J* = 16.3 Hz, 2H), 3.81 (s, 6H), 3.69 (d, *J* = 16.3 Hz, 2H), 3.67 (s, 6H), 2.31 – 2.22 (m, 2H).
^{13}C NMR (101 MHz, $CDCl_3$) δ 170.9, 159.4, 131.0, 128.7, 113.9, 83.4, 70.4, 65.6, 55.4, 51.8, 48.4.
IR (ν_{max}, cm^{-1}) 2360 (s), 2156 (m), 1753 (s), 1612 (s), 1512 (s), 1248 (s), 1211 (s), 1120 (s), 1034 (s), 833 (s), 700 (s).
HRMS (ESI/QTOF) m/z: $[M + Na]^+$ Calcd for $C_{26}H_{32}NaO_9^+$ 511.1939; Found 511.1954.

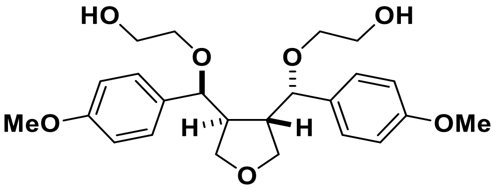

2,2'-(((1*S*,1'*S*)-((3*R*,4*R*)-tetrahydrofuran-3,4-diyl)bis((4-methoxyphenyl)methylene))bis(oxy))bis(ethan-1-ol) (**22o-A**)
74% yield, 11:1 dr, R_f = 0.15 (DCM/MeOH = 50:1), yellow oil.
^{1}H NMR (400 MHz, $CDCl_3$) δ 6.91 – 6.85 (m, 4H), 6.78 – 6.71 (m, 4H), 3.94 (dd, *J* = 9.0, 4.7 Hz, 2H), 3.86 – 3.78 (m, 4H), 3.81 (s, 6H), 3.66 – 3.56 (m, 4H), 3.29 (ddd, *J* = 9.0, 5.6, 3.3 Hz, 2H), 3.25 – 3.19 (m, 2H), 2.25 – 2.18 (m, 2H), 2.01 (brs, 2H).
^{13}C NMR (101 MHz, $CDCl_3$) δ 159.3, 132.2, 128.2, 114.0, 83.2, 70.6, 70.1, 62.1, 55.4, 48.7.
IR (ν_{max}, cm^{-1}) 2360 (s), 2158 (m), 1612 (s), 1512 (s), 1248 (s), 1107 (s), 1032 (s), 825 (s).
HRMS (ESI/QTOF) m/z: $[M + Na]^+$ Calcd for $C_{24}H_{32}NaO_7^+$ 455.2040; Found 455.2046.

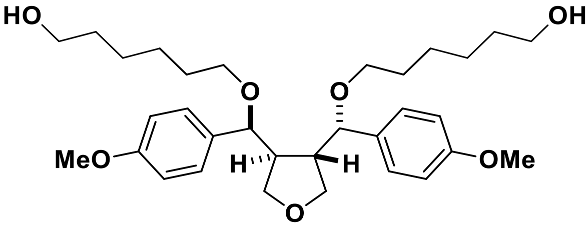

6,6'-(((1*S*,1'*S*)-((3*R*,4*R*)-tetrahydrofuran-3,4-diyl)bis((4-methoxyphenyl)methylene))bis(oxy))bis(hexan-1-ol) (**22p-A**)
68% yield, 9:1 dr, R_f = 0.15 (DCM/MeOH = 50:1), yellow oil.
^{1}H NMR (400 MHz, $CDCl_3$) δ 6.86 – 6.84 (m, 4H), 6.74 – 6.71 (m, 4H), 3.95 (dd, *J* = 9.0, 4.4 Hz, 2H), 3.83 – 3.79 (m, 2H), 3.80 (s, 6H), 3.67 (d, *J* = 8.0 Hz, 2H), 3.61 (t, *J* = 6.6 Hz, 4H), 3.16 (dt, *J* = 9.2, 6.3 Hz, 2H), 3.05 (dt, *J* = 9.3, 6.5 Hz, 2H), 2.19 – 2.10 (m, 2H), 1.57 – 1.42 (m, 10H), 1.33 – 1.27 (m, 8H).
^{13}C NMR (101 MHz, $CDCl_3$) δ 159.0, 133.0, 128.3, 113.7, 82.8, 70.6, 68.7, 63.0, 55.4, 48.9, 32.8, 29.8, 26.1, 25.6.
IR (ν_{max}, cm^{-1}) 2933 (m), 2856 (m), 2360 (s), 2158 (s), 1610 (s), 1512 (s), 1460 (s), 1246 (s), 1174 (s), 1034 (s), 829 (s), 719 (s).
HRMS (APPI/LTQ-Orbitrap) m/z: $[M + Na]^+$ Calcd for $C_{32}H_{48}NaO_7^+$ 567.3292; Found 567.3285.

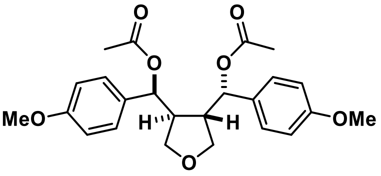

22q-A

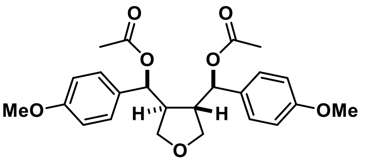

22q-B

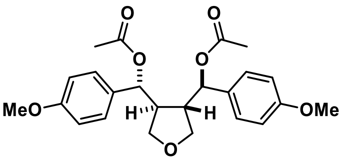

22q-C

((3*S*,4*S*)-tetrahydrofuran-3,4-diyl)bis((4-methoxyphenyl)methylene) diacetate (**22q**)
38% yield, **22q-A**: **22q-B** = 4:1 dr mixture, R_f = 0.3 (PE/EtOAc = 7:1), colorless oil. The diastereomer **22q-C** was not detected in our photoredox reaction.

^{1}H NMR (400 MHz, $CDCl_3$) (for 3 diastereoisomers in a ratio of approximately, **22q-A**: **22q-B** : **22q-C** = 1.5 : 2 : 1, prepared from S4.1.) δ 7.25 (d, *J* = 8.6 Hz, 2H), 7.19 (d, *J* = 8.6 Hz, 2H), 7.09 (d, *J* = 8.6 Hz, 2H), 6.94 (d, *J* = 8.6 Hz, 3H), 6.88 – 6.84 (m, 4H), 6.80 (d, *J* = 8.6 Hz, 2H), 6.74 (d, *J* = 8.6 Hz, 3H), 5.71 (d, *J* = 8.5 Hz, 1H), 5.64 (d, *J* = 8.1 Hz, 1H), 5.52 (d, *J* = 9.2 Hz, 1H), 5.38 (d, *J* = 8.1 Hz, 1.5H), 3.90 – 3.75 (m, 4H), 3.80 (s, 3H), 3.79 (s, 3H), 3.79 (s, 4.5H), 3.77 (s, 3H), 3.70 – 3.64 (m, 2H), 3.47 – 3.40 (m, 2H), 2.71 – 2.63 (m, 2H), 2.47 – 2.39 (m, 1H), 2.39 - 2.32 (m, 1.5H), 2.06 (s, 3H), 2.06 (s, 3H), 1.99 (s, 4.5), 1.96 (s, 3H).
^{13}C NMR (101 MHz, $CDCl_3$) (for 3 diastereoisomers) δ 170.3, 170.2, 170.1, 170.0, 159.6, 159.5, 159.4, 159.4, 131.4, 131.0, 130.9, 130.8, 128.7, 128.4, 128.2, 128.2, 114.0, 114.0, 113.9, 76.7, 76.7, 76.4, 76.3, 70.2, 70.1, 70.0, 69.9, 55.3, 55.3, 48.0, 47.5, 47.2, 47.0, 21.4, 21.3, 21.3, 21.2.
IR (ν_{max}, cm^{-1}) 1736 (m), 1612 (w), 1514 (m), 1371 (w), 1236 (s), 1176 (m), 1028 (m), 829 (w), 717 (w).
HRMS (ESI/QTOF) m/z: $[M + Na]^+$ Calcd for $C_{24}H_{28}NaO_7^+$ 451.1727; Found 451.1731.

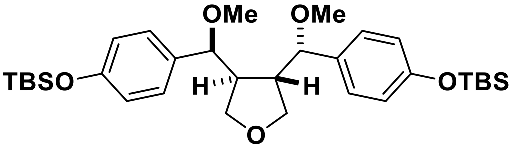

(3*R*,4*R*)-3,4-bis((*S*)-(4-((tert-butyldimethylsilyl)oxy)phenyl)(methoxy)methyl)tetrahydrofuran (**22r-A**)
72% yield, 10:1 dr, R_f = 0.35 (PE/EtOAc = 10:1), yellow oil.
^{1}H NMR (400 MHz, $CDCl_3$) δ 6.86 – 6.83 (m, 4H), 6.76 – 6.62 (m, 4H), 3.90 (dd, *J* = 8.9, 4.8 Hz, 2H), 3.79 (dd, *J* = 8.9, 6.6 Hz, 2H), 3.55 (d, *J* = 7.4 Hz, 2H), 3.06 (s, 6H), 2.18 – 2.12 (m, 2H), 0.99 (s, 18H), 0.20 (s, 12H).
^{13}C NMR (101 MHz, $CDCl_3$) δ 155.3, 133.2, 128.3, 120.1, 84.5, 70.5, 56.8, 49.0, 25.8, 18.4, -4.2, -4.3.
IR (ν_{max}, cm^{-1}) 2360 (s), 1608 (m), 1510 (s), 1257 (s), 914 (s), 841 (s), 779 (s), 717 (s).
HRMS (ESI/QTOF) m/z: $[M + Na]^+$ Calcd for $C_{32}H_{52}NaO_5Si_2^+$ 595.3245; Found 595.3273.

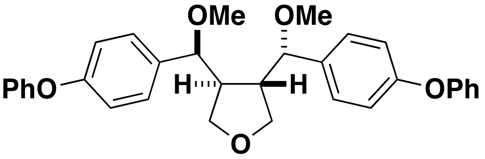

(3*S*,4*S*)-3,4-bis((*S*)-methoxy(4-phenoxyphenyl)methyl)tetrahydrofuran (**22s-A**)
67% yield, 10:1 dr, R_f = 0.35 (PE/EtOAc = 10:1), colorless oil.
^{1}H NMR (400 MHz, $CDCl_3$) δ 7.35 – 7.29 (m, 4H), 7.13 – 7.07 (m, 2H), 7.02 – 6.98 (m, 4H), 6.97 – 6.93 (m, 4H), 6.91 – 6.87 (m, 4H), 3.94 (dd, *J* = 9.0, 4.6 Hz, 2H), 3.84 (dd, *J* = 9.0, 6.5 Hz, 2H), 3.63 (d, *J* = 7.7 Hz, 2H), 3.11 (s, 6H), 2.19 – 2.14 (m, 2H).
^{13}C NMR (101 MHz, $CDCl_3$) δ 157.1, 157.1, 135.1, 129.9, 128.6, 123.6, 119.1, 118.8, 84.56, 70.5, 57.0, 48.9.
IR (ν_{max}, cm^{-1}) 2924 (w), 2360 (m), 1589 (m), 1502 (m), 1487 (s), 1234 (s), 1074 (m), 872 (m), 750 (m), 690 (m).
HRMS (ESI/QTOF) m/z: $[M + Na]^+$ Calcd for $C_{32}H_{32}NaO_5^+$ 519.2142; Found 519.2150.

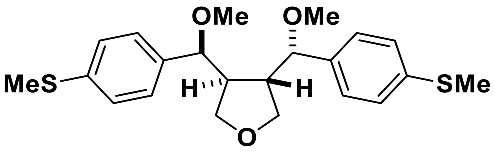

(3*R*,4*R*)-3,4-bis((*S*)-methoxy(4-(methylthio)phenyl)methyl)tetrahydrofuran (**22t-A**)
46% yield, 10:1 dr, R_f = 0.4 (PE/EtOAc = 10:1), white solid. M.p. = 92-94 ^{o}C.
^{1}H NMR (400 MHz, $CDCl_3$) δ 7.15 – 6.97 (m, 4H), 6.83 – 6.81 (m, 4H), 3.93 (dd, *J* = 9.1, 4.4 Hz, 2H), 3.84 (dd, *J* = 9.1, 6.5 Hz, 2H), 3.60 (d, *J* = 8.4 Hz, 2H), 3.04 (s, 6H), 2.51 (s, 6H), 2.15 – 2.06 (m, 2H).
^{13}C NMR (101 MHz, $CDCl_3$) δ 137.9, 136.9, 127.7, 126.3, 84.8, 70.7, 56.9, 48.6, 15.8.
IR (ν_{max}, cm^{-1}) 2925 (m), 2360 (m), 1599 (w), 1493 (m), 1439 (w), 1088 (s), 960 (m), 931 (m), 816 (m), 756 (w).
HRMS (APPI/LTQ-Orbitrap) m/z: $[M]^+$ Calcd for $C_{22}H_{28}O_3S_2^+$ 404.1474; Found 404.1469.

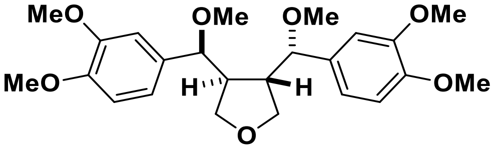

(3*R*,4*R*)-3,4-bis((*S*)-(3,4-dimethoxyphenyl)(methoxy)methyl)tetrahydrofuran (**22u-A**)
50% yield, 8:1 dr, R_f = 0.3 (DCM/EtOAc = 4:1), colorless oil.
^{1}H NMR (400 MHz, $CDCl_3$) δ 6.68 (d, *J* = 8.1 Hz, 2H), 6.48 (dd, *J* = 8.1, 1.8 Hz, 2H), 6.41 (d, *J* = 1.8 Hz, 2H), 3.95 (dd, *J* = 9.1, 4.2 Hz, 2H), 3.88 (s, 6H), 3.88 – 3.83 (m, 2H), 3.73 (s, 6H), 3.59 (d, *J* = 8.5 Hz, 2H), 3.05 (s, 6H), 2.16 – 2.09 (m, 2H).
^{13}C NMR (101 MHz, $CDCl_3$) δ 149.1, 148.5, 132.7, 119.8, 110.5, 109.4, 85.1, 70.6, 56.8, 55.9, 55.7, 48.9.
IR (ν_{max}, cm^{-1}) 2360 (s), 1680 (m), 1595 (m), 1514 (s), 1460 (m), 1267 (s), 1157 (m), 1080 (m), 1026 (s).
HRMS (ESI/QTOF) m/z: $[M + Na]^+$ Calcd for $C_{24}H_{32}NaO_7^+$ 455.2040; Found 455.2048.

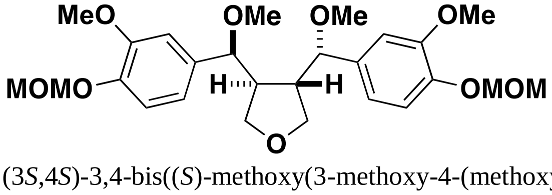

(3*S*,4*S*)-3,4-bis((*S*)-methoxy(3-methoxy-4-(methoxymethoxy)phenyl)methyl)tetrahydrofuran(**22v-A**)
55% yield, 8:1 dr, R_f = 0.3 (DCM/EtOAc = 5:1), colorless oil.

^{1}H NMR (400 MHz, $CDCl_3$) δ 6.98 (d, *J* = 8.6 Hz, 2H), 6.50-6.47 (m, 4H), 5.24 (d, *J* = 6.8 Hz, 2H), 5.20 (d, *J* = 6.8 Hz, 2H), 3.93 (dd, *J* = 9.1, 4.4 Hz, 2H), 3.84 (dd, *J* = 9.0, 6.5 Hz, 2H), 3.75 (s, 6H), 3.57 (d, *J* = 8.0 Hz, 2H), 3.51 (s, 6H), 3.07 (s, 6H), 2.16 – 2.10 (m, 2H).
^{13}C NMR (101 MHz, $CDCl_3$) δ 149.8, 146.1, 134.5, 119.9, 115.7, 109.8, 95.6, 85.0, 70.6, 56.9, 56.4, 55.8, 48.9.
IR (ν_{max}, cm^{-1}) 2360 (m), 1259 (s), 1080 (s), 1011 (s), 864 (m), 789 (s), 748 (m), 702 (m).
HRMS (ESI/QTOF) m/z: $[M + Na]^+$ Calcd for $C_{26}H_{36}NaO_9^+$ 515.2252; Found 515.2261.

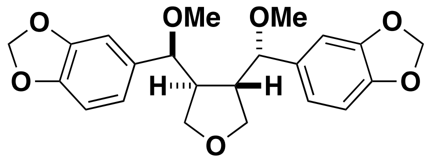

(3*S*,4*S*)-3,4-bis((*S*)-benzo[d][1,3]dioxol-5-yl(methoxy)methyl)tetrahydrofuran (**22w-A**)
62% yield, 9:1 dr, R_f = 0.55 (DCM), colorless oil.
^{1}H NMR (400 MHz, $CDCl_3$) δ 6.64 (d, *J* = 7.8 Hz, 2H), 6.44 (dd, *J* = 7.9, 1.5 Hz, 2H), 6.36 (d, *J* = 1.5 Hz, 2H), 5.97 (d *J* = 1.5 Hz, 2H), 5.93 (d *J* = 1.5 Hz, 2H), 3.92 (dd, *J* = 9.2, 4.2 Hz, 2H), 3.84 (dd, *J* = 9.1, 6.4 Hz, 2H), 3.57 (d, *J* = 8.8 Hz, 2H), 3.04 (s, 6H), 2.10 – 2.04 (m, 2H).
^{13}C NMR (101 MHz, $CDCl_3$) δ 147.8, 147.1, 134.1, 121.1, 107.7, 107.0, 101.1, 85.0, 70.8, 56.6, 48.7.
IR (ν_{max}, cm^{-1}) 1502 (m), 1486 (s), 1441 (m), 1379 (w), 1242 (s), 1183 (m), 1114 (m), 1078 (s), 1038 (s), 930 (s), 810 (m), 737 (m).
HRMS (ESI/QTOF) m/z: $[M + Na]^+$ Calcd for $C_{22}H_{24}NaO_7^+$ 423.1414; Found 423.1419.

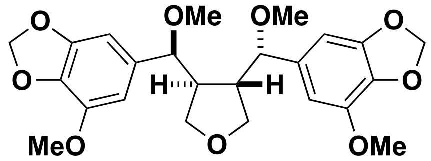

(3*S*,4*S*)-3,4-bis((*S*)-methoxy(7-methoxybenzo[d][1,3]dioxol-5-yl)methyl)tetrahydrofuran (**22x-A**)
44% yield, 8:1 dr, R_f = 0.35 (PE/EtOAc = 10:1), colorless oil.
^{1}H NMR (400 MHz, $CDCl_3$) δ 6.12 (s, 4H), 6.01 (d, *J* = 1.7 Hz, 2H), 5.96 (d, *J* = 1.6 Hz, 2H), 3.93 (dd, *J* = 9.3, 4.2 Hz, 2H), 3.86 (dd, *J* = 9.2, 6.3 Hz, 2H), 3.81 (s, 6H), 3.55 (d, *J* = 9.1 Hz, 2H), 3.06 (s, 6H), 2.07 (ddq, *J* = 9.9, 7.4, 3.2 Hz, 2H).
^{13}C NMR (101 MHz, $CDCl_3$) δ 148.8, 143.4, 134.8, 134.7, 106.2, 101.8, 101.3, 85.4, 70.9, 56.8, 56.4, 48.8.
IR (ν_{max}, cm^{-1}) 2362 (m), 2154 (m), 2023 (m), 1633 (s), 1506 (s), 1452 (s), 1429 (s), 1192 (s), 1132 (s), 1086 (s), 1041 (s), 937 (s), 843 (s), 719 (s).
HRMS (ESI/QTOF) m/z: $[M + Na]^+$ Calcd for $C_{24}H_{28}NaO_9^+$ 483.1626; Found 483.1634.

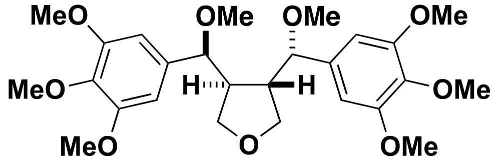

(3*S*,4*S*)-3,4-bis((*S*)-methoxy(3,4,5-trimethoxyphenyl)methyl)tetrahydrofuran (**22y-A**)
54% yield, 8:1 dr, R_f = 0.4 (PE/EtOAc = 2:1), pale yellow oil.
^{1}H NMR (400 MHz, $CDCl_3$) δ 6.24 (s, 4H), 3.94 (dd, *J* = 9.0, 4.7 Hz, 2H), 3.85 – 3.80 (m, 2H), 3.83 (s, 6H), 3.78 (s, 12H), 3.53 (d, *J* = 7.4 Hz, 2H), 3.11 (s, 6H), 2.22 – 2.11 (m, 2H).
^{13}C NMR (101 MHz, $CDCl_3$) δ 153.4, 137.4, 136.2, 103.7, 85.4, 70.5, 60.9, 57.1, 56.1, 49.0.
IR (ν_{max}, cm^{-1}) 2362 (w), 2156 (m), 2021 (m), 1591 (m), 1504 (m), 1460 (m), 1419 (m), 1327 (m), 1234 (m), 1126 (s), 1007 (m), 717 (m).
HRMS (ESI/QTOF) m/z: $[M + Na]^+$ Calcd for $C_{26}H_{36}NaO_9^+$ 515.2252; Found 515.2251.

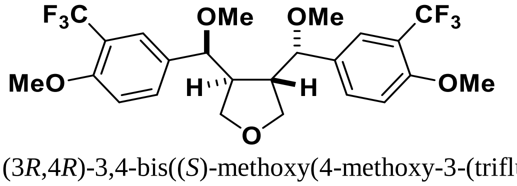

(3*R*,4*R*)-3,4-bis((*S*)-methoxy(4-methoxy-3-(trifluoromethyl)phenyl)methyl)tetrahydrofuran (**22z-A**)
66% yield, 10:1 dr, R_f = 0.4 (PE/EtOAc = 10:1), white solid. M.p. = 121-122 °C.
^{1}H NMR (400 MHz, $CDCl_3$) δ 7.15 (d, *J* = 2.0 Hz, 2H), 7.06 (dd, *J* = 8.5, 2.0 Hz, 2H), 6.81 (d, *J* = 8.5 Hz, 2H), 3.99 (dd, *J* = 9.2, 4.2 Hz, 2H), 3.90 – 3.86 (m, 2H), 3.90 (s, 6H), 3.63 (d, *J* = 8.6 Hz, 2H), 3.04 (s, 6H), 2.12 – 2.05 (m, 2H).
^{13}C NMR (101 MHz, $CDCl_3$) δ 157.1, 131.81, 131.79, 126.0 (q, *J* = 5.4 Hz), 123.6 (q, *J* = 272.4 Hz), 118.4 (q, *J* = 30.9 Hz), 112.2, 84.2, 70.6, 56.9, 55.9, 48.8.

IR (ν_{max}, cm^{-1}) 1620 (m), 1506 (m), 1323 (s), 1275 (s), 1188 (m), 1120 (s), 1084 (m), 1057 (s), 1026 (m), 823 (m), 669 (m).
^{19}F NMR (377 MHz, $CDCl_3$) δ -62.48.
HRMS (ESI/QTOF) m/z: [M + Na]$^+$ Calcd for $C_{24}H_{26}F_6NaO_5^+$ 531.1577; Found 531.1572.

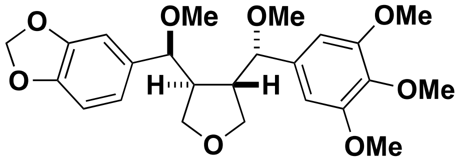

5-((*S*)-methoxy((3*S*,4*S*)-4-((*S*)-methoxy(3,4,5-trimethoxyphenyl)methyl)tetrahydrofuran-3-yl)methyl)benzo[d][1,3]dioxole (**22aa-A**)
37% yield, 10:1 dr, R_f = 0.4 (PE/EtOAc = 20:1), colorless oil.
^{1}H NMR (400 MHz, $CDCl_3$) δ 6.63 (d, *J* = 7.8 Hz, 1H), 6.45 (dd, *J* = 7.8, 1.5 Hz, 1H), 6.28 (d, *J* = 1.5 Hz, 1H), 6.16 (s, 2H), 6.02 (d, *J* = 1.6 Hz, 1H), 5.94 (d, *J* = 1.6 Hz, 1H), 3.97 – 3.92 (m, 2H), 3.89 – 3.82 (m, 5H), 3.77 (s, 6H), 3.57 (dd, *J* = 9.0, 2.1 Hz, 2H), 3.09 (s, 3H), 3.04 (s, 3H), 2.14 – 2.03 (m, 2H).
^{13}C NMR (101 MHz, $CDCl_3$) δ 153.2, 148.2, 147.3, 137.4, 135.9, 134.0, 121.4, 107.3, 106.5, 103.8, 101.5, 85.5, 85.2, 70.8, 70.6, 61.0, 57.0, 56.7, 56.0, 48.9, 48.7.
IR (ν_{max}, cm^{-1}) 2360 (w), 1712 (w), 1591 (m), 1504 (m), 1487 (m), 1444 (m), 1325 (m), 1238 (s), 1184 (m), 1122 (s), 1078 (s), 1036 (s), 1009 (m), 930 (m), 816 (m), 694 (m).
HRMS (ESI/QTOF) m/z: [M + Na]$^+$ Calcd for $C_{24}H_{30}NaO_8^+$ 469.1833; Found 469.1843.

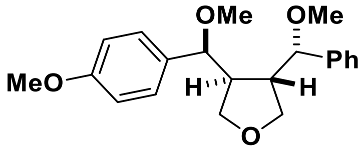

(3*R*,4*R*)-3-((*S*)-methoxy(4-methoxyphenyl)methyl)-4-((*S*)-methoxy(phenyl)methyl)tetrahydrofuran (**22ab-A**)
60% yield, 10:1 dr, R_f = 0.45 (PE/EtOAc = 10:1), white solid. M.p. = 109-110 °C.
^{1}H NMR (400 MHz, $CDCl_3$) δ 7.25 - 7.23 (m, 3H), 6.98 – 6.95 (m, 2H), 6.90 – 6.85 (m, 2H), 6.78 – 6.75 (m, 2H), 3.93 (dd, *J* = 8.9, 4.9 Hz, 2H), 3.88 – 3.76 (m, 2H), 3.81 (s, 3H), 3.60 (d, *J* = 7.5 Hz, 1H), 3.08 (s, 3H), 3.05 (s, 3H), 3.05 (d, *J* = 7.8 Hz, 1H), 2.22 – 2.13 (m, 2H).
^{13}C NMR (101 MHz, $CDCl_3$) δ 159.2, 140.5, 132.4, 128.5, 128.3, 127.8, 127.2, 113.9, 84.9, 84.7, 70.7, 70.4, 57.0, 56.7, 55.4, 48.9, 48.8.
IR (ν_{max}, cm^{-1}) 1610 (s), 1512 (s), 1344 (s), 1248 (s), 1163 (s), 1034 (s), 825 (s), 758 (s), 717 (s), 663 (s).
HRMS (ESI/QTOF) m/z: [M + Na]$^+$ Calcd for $C_{21}H_{26}NaO_4^+$ 365.1723; Found 365.1720.

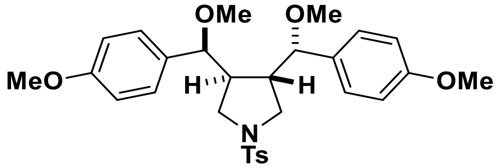

(3*R*,4*R*)-3,4-bis((*S*)-methoxy(4-methoxyphenyl)methyl)-1-tosylpyrrolidine (**22ac-A**)
72% yield, 10:1 dr, R_f = 0.3 (PE/EtOAc = 10:1), white solid. M.p. = 112-113 °C.
^{1}H NMR (400 MHz, $CDCl_3$) δ 7.75 (d, *J* = 8.2 Hz, 2H), 7.36 (d, *J* = 8.0 Hz, 2H), 6.80 – 6.75 (m, 8H), 3.79 (s, 6H), 3.42 (dd, *J* = 10.2, 4.3 Hz, 2H), 3.31 (d, *J* = 7.8 Hz, 2H), 3.18 (dd, *J* = 10.1, 6.7 Hz, 2H), 2.92 (s, 6H), 2.45 (s, 3H), 2.06 – 1.98 (m, 2H).
^{13}C NMR (101 MHz, $CDCl_3$) δ 159.4, 143.4, 133.3, 131.6, 129.7, 128.3, 128.0, 113.9, 83.6, 56.6, 55.4, 49.6, 47.1, 21.7.
IR (ν_{max}, cm^{-1}) 2365 (s), 2023 (s), 1520 (m), 1344 (m), 1248 (m), 1162 (s), 1011 (m), 757 (s), 746 (m), 652 (s).
HRMS (ESI/QTOF) m/z: [M + Na]$^+$ Calcd for $C_{29}H_{35}NNaO_6S^+$ 548.2077; Found 548.2080.

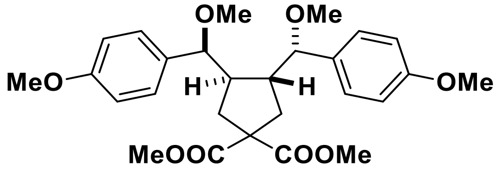

dimethyl (3*S*,4*S*)-3,4-bis((*S*)-methoxy(4-methoxyphenyl)methyl)cyclopentane-1,1-dicarboxylate (**22ad-A**)
51% yield, 7:1 dr, R_f = 0.25 (PE/EtOAc = 10:1), colorless oil.
^{1}H NMR (400 MHz, $CDCl_3$) δ 7.01 – 6.99 (m, 4H), 6.85 – 6.83 (m, 4H), 3.80 (s, 6H), 3.69 (s, 6H), 3.46 (d, *J* = 5.5 Hz, 2H), 3.06 (s, 6H), 2.39 – 2.24 (m, 4H), 2.22 – 2.13 (m, 2H).
^{13}C NMR (101 MHz, $CDCl_3$) δ 173.0, 159.1, 133.3, 128.2, 113.8, 84.5, 59.3, 56.9, 55.4, 52.7, 47.9, 35.9.
IR (ν_{max}, cm^{-1}) 2360 (s), 2156 (m), 1610 (m), 1512 (s), 1454 (s), 1246 (s), 1176 (s), 1088 (s), 1034 (s), 833 (s), 715

(s).
HRMS (ESI/QTOF) m/z: $[M + Na]^+$ Calcd for $C_{27}H_{34}NaO_8^+$ 509.2146; Found 509.2153.

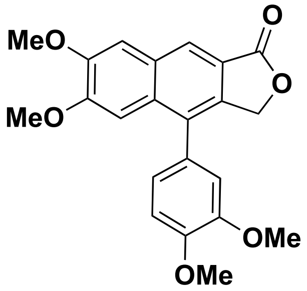

4-(3,4-dimethoxyphenyl)-6,7-dimethoxynaphtho[2,3-c]furan-1(3H)-one (**7**, known compound: *J. Org. Chem.* **2011**, *76*, 9919−9933)
73% yield, R_f = 0.5 (PE/EtOAc = 1:1), yellow solid. M.p. = 87-88 ºC.
^{1}H NMR (400 MHz, $CDCl_3$) δ 8.31 (s, 1H), 7.31 (s, 1H), 7.12 (s, 1H), 7.05 (d, *J* = 8.2 Hz, 1H), 6.96 (dd, *J* = 8.1, 1.9 Hz, 1H), 6.90 (d, *J* = 1.8 Hz, 1H), 5.27 (d, *J* = 14.9 Hz, 1H), 5.20 (d, *J* = 14.9 Hz, 1H), 4.06 (s, 3H), 3.99 (s, 3H), 3.89 (s, 3H), 3.83 (s, 3H).
^{13}C NMR (101 MHz, $CDCl_3$) δ 171.6, 152.0, 150.1, 149.3, 149.0, 137.9, 132.2, 131.7, 129.9, 128.7, 124.1, 121.6, 121.4, 112.2, 111.7, 107.7, 104.2, 69.6, 56.1, 56.1, 56.0, 55.9.
IR (ν_{max}, cm^{-1}) 2360 (m), 1753 (s), 1620 (m), 1508 (s), 1485 (s), 1458 (s), 1431 (s), 1342 (m), 1257 (s), 1228 (s), 1157 (s), 1005 (s), 735 (s).
HRMS (ESI/QTOF) m/z: $[M + H]^+$ Calcd for $C_{22}H_{21}O_6^+$ 381.1333; Found 381.1337.

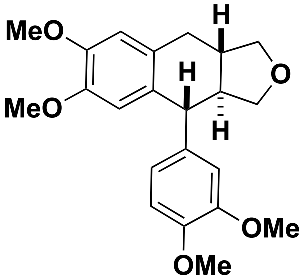

(3a*S*,4*R*,9a*S*)-4-(3,4-dimethoxyphenyl)-6,7-dimethoxy-1,3,3a,4,9,9a-hexahydronaphtho[2,3-c]furan (**19a**)
94% yield (11:1 dr). White solid. M.p = 132– 133ºC.
^{1}H NMR (400 MHz, $CDCl_3$) (major) δ 6.81 (d, *J* = 8.2 Hz, 1H), 6.72 (dd, *J* = 8.2, 1.9 Hz, 1H), 6.64 (s, 1H), 6.59 (d, *J* = 1.8 Hz, 1H), 6.31 (s, 1H), 4.21 (t, *J* = 7.4 Hz, 1H), 3.89 (s, 3H), 3.87 (s, 3H), 3.84 – 3.72 (m, 2H), 3.80 (s, 3H), 3.60 (s, 3H), 3.54 (ddd, *J* = 10.7, 7.9, 3.2 Hz, 2H), 3.00 (dd, *J* = 15.4, 4.5 Hz, 1H), 2.79 – 2.72 (m, 1H), 2.33 – 2.15 (m, 2H).
^{13}C NMR (101 MHz, $CDCl_3$) (major) δ 149.2, 147.9, 147.6, 147.4, 137.4, 132.0, 128.6, 120.9, 112.5, 111.8, 111.2, 111.1, 73.4, 72.5, 56.1, 56.0, 51.0, 49.9, 42.4, 32.6.
IR (ν_{max}, cm^{-1}) 1512 (s), 1462 (m), 1263 (s), 1246 (s), 1219 (s), 1155 (m), 1142 (m), 1093 (m), 1026 (m), 993 (m), 901 (m), 868 (w), 760 (w), 735 (w), 688 (w), 669 (w).
HRMS (APPI/LTQ-Orbitrap) m/z: $[M]^+$ Calcd for $C_{22}H_{26}O_5^+$ 370.1775; Found 370.1770.

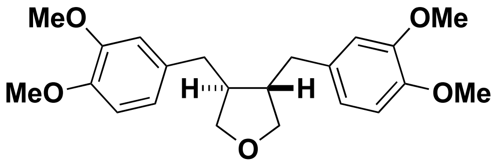

(3*S*,4*S*)-3,4-bis(3,4-dimethoxybenzyl)tetrahydrofuran (**11**, brassilignan)
76% yield, R_f = 0.5 (PE/EtOAc = 8:1), White solid. M.p = 87– 88 ºC.
^{1}H NMR (400 MHz, $CDCl_3$) δ 6.76 (d, *J* = 8.1 Hz, 2H), 6.63 (dd, *J* = 8.1, 1.9 Hz, 2H), 6.59 (d, *J* = 1.9 Hz, 2H), 3.91 (dd, *J* = 8.7, 6.7 Hz, 2H), 3.85 (s, 6H), 3.84 (s, 6H), 3.53 (dd, *J* = 8.7, 6.0 Hz, 2H), 2.64 (dd, *J* = 13.7, 6.1 Hz, 2H), 2.53 (dd, *J* = 13.7, 8.3 Hz, 2H), 2.24 – 2.15 (m, 2H).
^{13}C NMR (101 MHz, $CDCl_3$) δ 149.0, 147.5, 133.1, 120.7, 112.1, 111.3, 73.4, 56.0, 56.0, 46.8, 39.2.
IR (ν_{max}, cm^{-1}) 2360 (s), 2156 (m), 1514 (s), 1460 (s), 1261 (s), 1236 (s), 1155 (s), 1028 (s), 717 (s).
HRMS (ESI/QTOF) m/z: $[M + Na]^+$ Calcd for $C_{22}H_{28}NaO_5^+$ 395.1829; Found 395.1830.

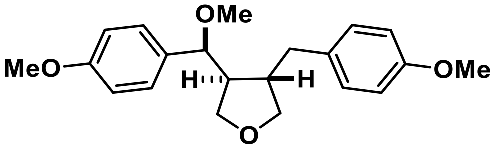

(3*S*,4*S*)-3-(methoxy(4-methoxyphenyl)methyl)-4-(4-methoxybenzyl)tetrahydrofuran (**24a-A**)
80% yield, > 20:1 dr, R_f = 0.65 (PE/EtOAc = 6:1), colorless oil.

Major isomer:

^{1}H NMR (400 MHz, $CDCl_3$) δ 7.16 (d, *J* = 8.6 Hz, 2H), 6.87 (d, *J* = 8.7 Hz, 2H), 6.76 – 6.69 (m, 4H), 4.00 – 3.89 (m, 2H), 3.86 – 3.75 (m, 2H), 3.82 (s, 3H), 3.76 (s, 3H), 3.43 (dd, *J* = 8.8, 6.2 Hz, 1H), 3.12 (s, 3H), 2.26 (dd, *J* = 13.5, 9.5 Hz, 1H), 2.23 – 2.17 (m, 1H), 2.12 (dd, *J* = 13.6, 5.7 Hz, 1H), 2.05 – 1.96 (m, 1H).
^{13}C NMR (101 MHz, $CDCl_3$) δ 159.5, 158.0, 132.6, 132.4, 129.6, 128.7, 113.9, 113.8, 85.9, 73.5, 71.8, 56.5, 55.4, 55.4, 51.9, 44.0, 38.8.
IR (ν_{max}, cm^{-1}) 2360 (m), 2158 (m), 1610 (m), 1512 (s), 1462 (m), 1300 (m), 1246 (s), 1176 (m), 1107 (m), 1082 (m), 1036 (s), 829 (m), 754 (s).
HRMS (ESI/QTOF) m/z: $[M + Na]^+$ Calcd for $C_{21}H_{26}NaO_4^+$ 365.1723; Found 365.1724.

Minor isomer: (**24a-minor**)
R_f = 0.75 (PE/EtOAc = 7:1), colorless oil.
^{1}H NMR (400 MHz, $CDCl_3$) δ 7.22 – 7.17 (m, 2H), 7.12 – 7.06 (m, 2H), 6.91 – 6.86 (m, 2H), 6.85 – 6.80 (m, 2H), 3.95 (d, *J* = 8.7 Hz, 1H), 3.82 (s, 3H), 3.79 (s, 3H), 3.76 (dd, *J* = 8.7, 6.7 Hz, 1H), 3.62 (dd, *J* = 9.0, 7.9 Hz, 1H), 3.50 (dd, *J* = 8.8, 5.6 Hz, 1H), 3.40 (dd, *J* = 9.1, 6.1 Hz, 1H), 3.18 (s, 3H), 2.94 (dd, *J* = 12.8, 3.8 Hz, 1H), 2.58 – 2.52 (m, 1H), 2.49 (dd, *J* = 12.8, 10.5 Hz, 1H), 2.34 – 2.27 (m, 1H).
^{13}C NMR (101 MHz, $CDCl_3$) δ 159.4, 158.0, 133.1, 132.7, 129.9, 128.6, 114.0, 113.9, 85.8, 73.4, 70.3, 56.6, 55.4, 51.9, 44.8, 39.4.
IR (ν_{max}, cm^{-1}) 2341 (m), 2158 (m), 1512 (s), 1248 (s), 1036 (s), 1012 (s), 771 (s), 758 (s), 719 (s).
HRMS (ESI/QTOF) m/z: $[M + Na]^+$ Calcd for $C_{21}H_{26}NaO_4^+$ 365.1723; Found 365.1724.

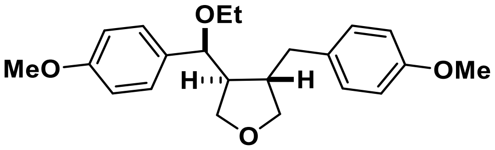

(3*S*,4*S*)-3-(ethoxy(4-methoxyphenyl)methyl)-4-(4-methoxybenzyl)tetrahydrofuran (**24b-A**)
66% yield, > 20:1 dr, R_f = 0.53 (PE/EtOAc = 5:1), colorless oil.
^{1}H NMR (400 MHz, $CDCl_3$) δ 7.16 (d, *J* = 8.8 Hz, 2H), 6.86 (d, *J* = 8.8 Hz, 2H), 6.74 (d, *J* = 8.8 Hz, 2H), 6.70 (d, *J* = 8.8 Hz, 2H), 4.01 – 3.91 (m, 3H), 3.82 (s, 3H), 3.78 (dd, *J* = 10.5, 7.1 Hz, 1H), 3.76 (s, 3H), 3.42 (dd, *J* = 8.8, 6.1 Hz, 1H), 3.30 (dq, *J* = 9.3, 7.0 Hz, 1H), 3.21 (dq, *J* = 9.4, 7.0 Hz, 1H), 2.25 (dd, *J* = 13.8, 9.6 Hz, 1H), 2.24-2.17 (m, 1H), 2.12 (dd, *J* = 13.8, 5.7 Hz, 1H), 2.05 – 1.95 (m, 1H), 1.11 (t, *J* = 7.0 Hz, 3H).
^{13}C NMR (101 MHz, $CDCl_3$) δ 159.3, 157.9, 133.2, 132.6, 129.6, 128.6, 113.9, 113.8, 83.9, 73.5, 71.8, 63.9, 55.4, 55.3, 51.8, 43.9, 38.8, 15.3.
IR (ν_{max}, cm^{-1}) 1610 (m), 1512 (s), 1462 (w), 1442 (w), 1300 (m), 1246 (s), 1176 (m), 1086 (m), 1036 (m), 829 (m).
HRMS (nanochip-ESI/LTQ-Orbitrap) m/z: $[M + Na]^+$ Calcd for $C_{22}H_{28}NaO_4^+$ 379.1880; Found 379.1880.

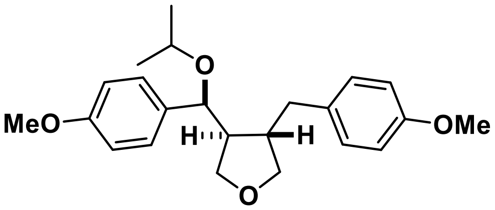

(3*S*,4*S*)-3-(isopropoxy(4-methoxyphenyl)methyl)-4-(4-methoxybenzyl)tetrahydrofuran (**24c-A**)
42% yield, > 20:1 dr, R_f = 0.56 (PE/EtOAc = 5:1), colorless oil.
^{1}H NMR (400 MHz, $CDCl_3$) δ 7.18 (d, *J* = 8.7 Hz, 2H), 6.86 (d, *J* = 8.8 Hz, 2H), 6.74 (d, *J* = 9.0 Hz, 2H), 6.70 (d, *J* = 9.0 Hz, 2H), 4.06 (d, *J* = 9.2 Hz, 1H), 3.96 (dd, *J* = 9.2, 7.3 Hz, 1H), 3.90 (dd, *J* = 9.2, 5.2 Hz, 1H), 3.82 (s, 3H), 3.77 (dd, *J* = 8.8, 7.7 Hz, 1H), 3.76 (s, 3H), 3.45 – 3.36 (m, 2H), 2.23 (dd, *J* = 13.7, 9.5 Hz, 1H), 2.19 – 2.14 (m, 1H), 2.11 (dd, *J* = 13.6, 5.7 Hz, 1H), 2.04-1.96 (m, 1H), 1.10 (d, *J* = 6.0 Hz, 3H), 0.99 (d, *J* = 6.3 Hz, 3H).
^{13}C NMR (101 MHz, $CDCl_3$) δ 159.3, 157.9, 133.9, 132.7, 129.6, 128.6, 113.8, 81.1, 73.6, 72.0, 68.5, 55.4, 55.4, 52.0, 43.9, 38.8, 23.7, 21.1.
IR (ν_{max}, cm^{-1}) 2968 (m), 1611 (m), 1512 (s), 1247 (s), 1176 (m), 1035 (m).
HRMS (nanochip-ESI/LTQ-Orbitrap) m/z: $[M + Na]^+$ Calcd for $C_{23}H_{30}NaO_4^+$ 393.2036; Found 393.2048.

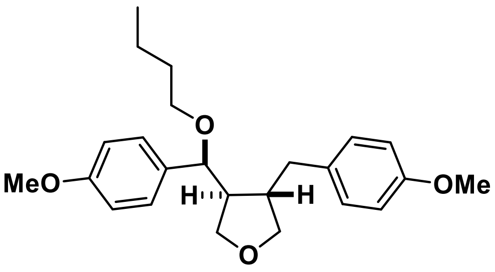

(3*S*,4*S*)-3-(butoxy(4-methoxyphenyl)methyl)-4-(4-methoxybenzyl)tetrahydrofuran (**24d-A**)
45% yield, > 20:1 dr, R_f = 0.57 (PE/EtOAc = 5:1), colorless oil.
^{1}H NMR (400 MHz, $CDCl_3$) δ 7.16 (d, *J* = 8.8 Hz, 2H), 6.86 (d, *J* = 8.8 Hz, 2H), 6.75 (d, *J* = 8.9 Hz, 2H), 6.70 (d, *J* = 8.9 Hz, 2H), 4.01 – 3.89 (m, 3H), 3.82 (s, 3H), 3.77 (dd, *J* = 9.0, 7.3 Hz, 1H), 3.76 (s, 3H), 3.43 (dd, *J* = 8.8, 6.1 Hz, 1H), 3.24 (dt, *J* = 9.3, 6.4 Hz, 1H), 3.13 (dt, *J* = 9.3, 6.5 Hz, 1H), 2.26 (dd, *J* = 13.6, 9.5 Hz, 1H), 2.24-2.17 (m, 1H), 2.12 (dd, *J* = 13.6, 5.8 Hz, 1H), 2.06 – 1.96 (m, 1H), 1.51 – 1.42 (m, 2H), 1.36 – 1.25 (m, 2H), 0.86 (t, *J* = 7.3 Hz, 3H).
^{13}C NMR (101 MHz, $CDCl_3$) δ 159.3, 157.9, 133.2, 132.7, 129.6, 128.6, 113.8, 113.8, 84.0, 73.5, 71.8, 68.5, 55.4, 55.3, 52.0, 43.9, 38.8, 32.0, 19.6, 14.0.
IR (ν_{max}, cm^{-1}) 2931 (m), 2841 (m), 1610 (m), 1512 (s), 1462 (m), 1300 (m), 1246 (s), 1176 (m), 1090 (m), 1036 (m), 823 (m).
HRMS (ESI/QTOF) m/z: $[M + Na]^+$ Calcd for $C_{24}H_{32}NaO_4^+$ 407.2193; Found 407.2190.

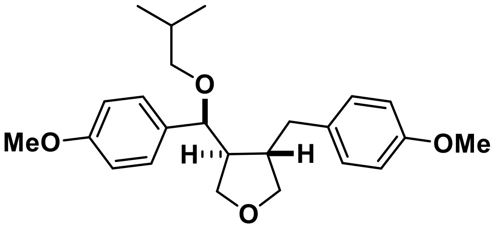

(3*S*,4*S*)-3-(isobutoxy(4-methoxyphenyl)methyl)-4-(4-methoxybenzyl)tetrahydrofuran (**24e-A**)
48% yield, > 20:1 dr, R_f = 0.56 (PE/EtOAc = 5:1), colorless oil.
^{1}H NMR (400 MHz, $CDCl_3$) δ 7.15 (d, *J* = 8.8 Hz, 2H), 6.86 (d, *J* = 8.8 Hz, 2H), 6.75 (d, *J* = 8.8 Hz, 2H), 6.70 (d, *J* = 8.8 Hz, 2H), 4.02 – 3.93 (m, 2H), 3.91 (d, *J* = 9.4 Hz, 1H), 3.82 (s, 3H), 3.77 (dd, *J* = 8.9, 6.6 Hz, 1H), 3.76 (s, 3H), 3.44 (dd, *J* = 8.9, 6.1 Hz, 1H), 3.02 (dd, *J* = 8.9, 6.4 Hz, 1H), 2.88 (dd, *J* = 8.9, 6.6 Hz, 1H), 2.25 (dd, *J* = 13.7, 9.5 Hz, 1H), 2.24 – 2.17 (m, 1H), 2.12 (dd, *J* = 13.5, 5.8 Hz, 1H), 2.07-1.98 (m, 1H), 1.82-1.72 (m, 1H), 0.86 (d, *J* = 6.6 Hz, 3H), 0.83 (d, *J* = 6.6 Hz, 3H).
^{13}C NMR (101 MHz, $CDCl_3$) δ 159.3, 157.9, 133.3, 132.7, 129.6, 128.6, 113.8, 113.8, 84.2, 75.7, 73.5, 71.8, 55.4, 55.4, 52.1, 43.9, 38.9, 28.7, 19.7, 19.6.
IR (ν_{max}, cm^{-1}) 2954 (w), 2838 (w), 1611 (m), 1512 (s), 1464 (w), 1299 (w), 1246 (s), 1176 (m), 1082 (m), 1036 (m), 830 (m).
HRMS (ESI/QTOF) m/z: $[M + Na]^+$ Calcd for $C_{24}H_{32}NaO_4^+$ 407.2193; Found 407.2194.

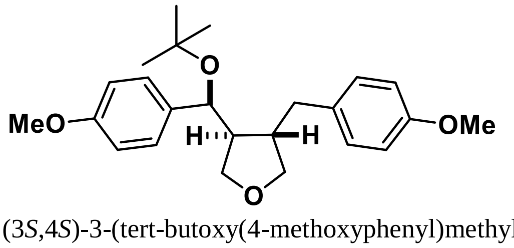

(3*S*,4*S*)-3-(tert-butoxy(4-methoxyphenyl)methyl)-4-(4-methoxybenzyl)tetrahydrofuran (**24f-A**)
35% yield, > 20:1 dr, R_f = 0.56 (PE/EtOAc = 5:1), colorless oil.
^{1}H NMR (400 MHz, $CDCl_3$) δ 7.19 (d, *J* = 8.8 Hz, 2H), 6.83 (d, *J* = 8.8 Hz, 2H), 6.76 (d, *J* = 8.8 Hz, 2H), 6.71 (d, *J* = 8.8 Hz, 2H), 4.23 (d, *J* = 8.4 Hz, 1H), 3.91 – 3.86 (m, 2H), 3.81 (s, 3H), 3.77 (dd, *J* = 8.7, 6.9 Hz, 1H), 3.76 (s, 3H), 3.43 (dd, *J* = 8.8, 5.9 Hz, 1H), 2.21 (dd, *J* = 13.4, 9.5 Hz, 1H), 2.14 – 2.02 (m, 3H), 1.06 (s, 9H).
^{13}C NMR (101 MHz, $CDCl_3$) δ 158.7, 157.8, 137.0, 132.7, 129.5, 128.0, 113.7, 113.5, 75.6, 74.3, 73.6, 71.7, 55.2, 53.0, 43.6, 38.8, 29.1.
IR (ν_{max}, cm^{-1}) 2929 (m), 2839 (m), 1612 (m), 1512 (s), 1462 (m), 1365 (m), 1300 (m), 1246 (s), 1176 (m), 1036 (m), 823 (m).
HRMS (ESI/QTOF) m/z: $[M + Na]^+$ Calcd for $C_{24}H_{32}NaO_4^+$ 407.2193; Found 407.2191.

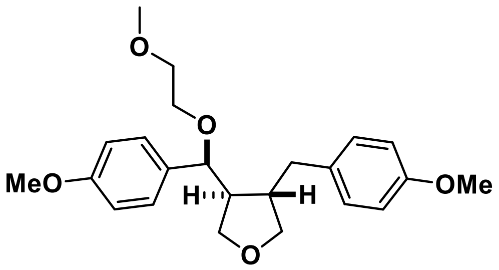

(3*S*,4*S*)-3-(4-methoxybenzyl)-4-((2-methoxyethoxy)(4-methoxyphenyl)methyl)tetrahydrofuran (**24g-A**)
40% yield, > 20:1 dr, R_f = 0.48 (PE/EtOAc = 1:1), colorless oil.
^{1}H NMR (400 MHz, $CDCl_3$) δ 7.17 (d, *J* = 8.8 Hz, 2H), 6.86 (d, *J* = 8.8 Hz, 2H), 6.73 (d, *J* = 8.9 Hz, 2H), 6.69 (d, *J* = 8.9 Hz, 2H), 4.01 – 3.96 (m, 3H), 3.82 (s, 3H), 3.77 (dd, *J* = 8.8, 7.0 Hz, 1H), 3.76 (s, 3H), 3.52 – 3.38 (m, 4H), 3.34-3.29 (m, 1H), 3.33 (s, 3H), 2.29 – 2.20 (m, 2H), 2.11 (dd, *J* = 13.6, 5.8 Hz, 1H), 2.04 – 1.95 (m, 1H).
^{13}C NMR (101 MHz, $CDCl_3$) δ 159.4, 157.9, 132.7, 132.6, 129.6, 128.8, 113.9, 113.8, 84.7, 73.5, 72.1, 71.9, 67.8, 59.1, 55.4, 55.3, 51.7, 43.9, 38.8.
IR (ν_{max}, cm^{-1}) 2929 (m), 1610 (m), 1512 (s), 1467 (m), 1248 (s), 1177 (m), 1129 (m), 1107 (m), 1032 (m), 832 (m), 810 (m), 759 (s), 719 (s).
HRMS (ESI/QTOF) m/z: $[M + Na]^+$ Calcd for $C_{23}H_{30}NaO_5^+$ 409.1985; Found 409.1994.

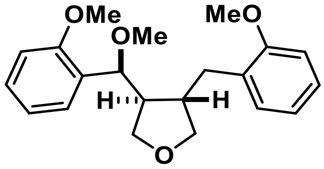

(3*S*,4*S*)-3-(methoxy(2-methoxyphenyl)methyl)-4-(2-methoxybenzyl)tetrahydrofuran (**24h-A**)
49% yield, 7.5:1 dr mixture, R_f = 0.45 (PE/EtOAc = 10:1), colorless oil.
^{1}H NMR (400 MHz, $CDCl_3$) (major) δ 7.24 – 7.19 (m, 2H), 7.12 (td, *J* = 8.1, 1.8 Hz, 1H), 6.94 – 6.84 (m, 3H), 6.79 (td, *J* = 7.4, 1.0 Hz, 1H), 6.72 (d, *J* = 8.2 Hz, 1H), 4.53 (d, *J* = 7.6 Hz, 1H), 3.93 (dd, *J* = 9.0, 5.2 Hz, 1H), 3.88 (dd, *J* = 8.9, 6.9 Hz, 1H), 3.81 (dd, *J* = 8.6, 6.5 Hz, 1H), 3.79 (s, 3H), 3.64 (s, 3H), 3.49 (dd, *J* = 8.6, 5.4 Hz, 1H), 3.15 (s, 3H), 2.51 (dd, *J* = 13.3, 8.6 Hz, 1H), 2.42 (dd, *J* = 13.3, 5.9 Hz, 1H), 2.37 – 2.27 (m, 2H).
^{13}C NMR (101 MHz, $CDCl_3$) (major) δ 157.7, 157.5, 130.5, 129.0, 129.0, 128.4, 127.5, 127.3, 120.9, 120.3, 110.3, 110.2, 78.3, 73.7, 70.8, 56.9, 55.4, 55.1, 50.9, 41.3, 34.2.
IR (ν_{max}, cm^{-1}) 2360 (m), 1599 (m), 1491 (s), 1462 (m), 1242 (s), 1103 (m), 1028 (s), 754 (s).
HRMS (ESI/QTOF) m/z: $[M + Na]^+$ Calcd for $C_{21}H_{26}NaO_4^+$ 365.1723; Found 365.1720.

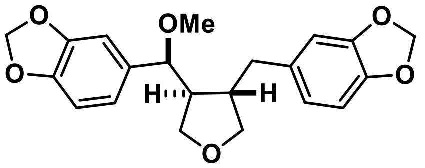

5-(((3*S*,4*S*)-4-(benzo[d][1,3]dioxol-5-yl(methoxy)methyl)tetrahydrofuran-3-yl)methyl)benzo[d][1,3]dioxole (**24i-A**)
42% yield, > 20:1 dr, R_f = 0.4 (DCM/EtOAc = 50:1), colorless oil.
^{1}H NMR (400 MHz, $CDCl_3$) δ 6.75 (d, *J* = 8.3 Hz, 1H), 6.68 – 6.64 (m, 2H), 6.61 (d, *J* = 7.8 Hz, 1H), 6.32 (dd, *J* = 7.9, 1.5 Hz, 1H), 6.29 (d, *J* = 1.5 Hz, 1H), 5.97 (d, *J* = 1.5 Hz, 1H), 5.96 (d, *J* = 1.5 Hz, 1H), 5.90 (d, *J* = 1.5 Hz, 1H), 5.89 (d, *J* = 1.5 Hz, 1H), 3.97 (dd, *J* = 9.2, 7.1 Hz, 1H), 3.92 (dd, *J* = 9.3, 5.0 Hz, 1H), 3.81 (dd, *J* = 8.8, 7.1 Hz, 1H), 3.76 (d, *J* = 9.5 Hz, 1H), 3.43 (dd, *J* = 8.8, 5.8 Hz, 1H), 3.11 (s, 3H), 2.30 (dd, *J* = 13.6, 8.6 Hz, 1H), 2.18 – 2.11 (m, 2H), 2.04 – 1.95 (m, 1H).
^{13}C NMR (101 MHz, $CDCl_3$) δ 148.1, 147.6, 147.4, 145.8, 134.4, 134.2, 121.6, 121.4, 109.0, 108.1, 107.9, 107.2, 101.2, 100.9, 86.1, 73.5, 71.7, 56.6, 51.6, 43.9, 39.7.
IR (ν_{max}, cm^{-1}) 2362 (s), 2152 (s), 1489 (s), 1441 (s), 1244 (s), 1097 (m), 1078 (m), 1038 (s), 933 (m), 806 (m), 719 (s).
HRMS (nanochip-ESI/LTQ-Orbitrap) m/z: $[M]^+$ Calcd for $C_{21}H_{22}O_6^+$ 370.1411; Found 370.1422.

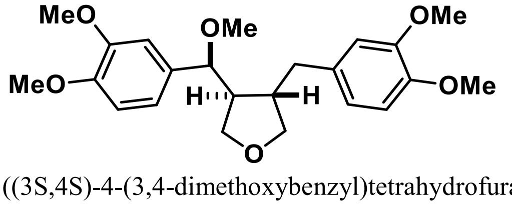

((3S,4S)-4-(3,4-dimethoxybenzyl)tetrahydrofuran-3-yl)(3,4-dimethoxyphenyl)methanol (**24j-A**)
60% yield, > 20:1 dr, R_f = 0.4 (PE/EtOAc = 1:2), colorless oil.
^{1}H NMR (400 MHz, $CDCl_3$) δ 6.82 (d, *J* = 8.0 Hz, 1H), 6.76 (dd, *J* = 8.0, 1.9 Hz, 1H), 6.72 (d, *J* = 1.9 Hz, 1H), 6.65

(d, J = 8.2 Hz, 1H), 6.37 (dd, J = 8.1, 2.1 Hz, 1H), 6.28 (d, J = 2.0 Hz, 1H), 4.00 (dd, J = 9.2, 7.2 Hz, 1H), 3.94 (dd, J = 9.3, 5.0 Hz, 1H), 3.89 (s, 3H), 3.84 (s, 3H), 3.83 (s, 3H), 3.82 – 3.79 (m, 2H), 3.76 (s, 3H), 3.47 (dd, J = 8.9, 5.6 Hz, 1H), 3.13 (s, 3H), 2.32 (dd, J = 13.6, 9.0 Hz, 1H), 2.20 (ddt, J = 9.5, 7.1, 4.9 Hz, 1H), 2.14 (dd, J = 13.6, 6.3 Hz, 1H), 2.08 – 1.96 (m, 1H).
^{13}C NMR (101 MHz, $CDCl_3$) δ 149.2, 148.7, 148.6, 147.2, 132.9, 132.8, 120.4, 120.1, 111.8, 111.0, 110.5, 109.7, 86.1, 73.4, 71.6, 56.5, 55.9, 55.86, 55.84, 55.83, 51.6, 43.9, 39.5.
IR (ν_{max}, cm^{-1}) 2871 (m), 2839 (m), 2276 (m), 2355 (m), 1591 (m), 1514 (s), 1460 (m), 1417 (m), 1261 (s), 1236 (s), 1155 (m), 1140 (m), 1028 (s), 758 (s).
HRMS (ESI/QTOF) m/z: [M + Na]$^+$ Calcd for $C_{23}H_{30}NaO_6^+$ 425.1935; Found 425.1945.

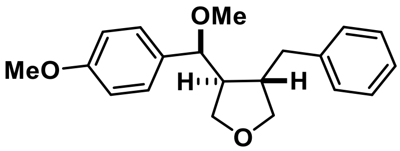

(3*S*,4*S*)-3-benzyl-4-(methoxy(4-methoxyphenyl)methyl)tetrahydrofuran (**24k-A**)
54% yield, > 20:1 dr, R_f = 0.45 (PE/EtOAc = 7:1), colorless oil
^{1}H NMR (400 MHz, $CDCl_3$) δ 7.21 – 7.09 (m, 5H), 6.91 – 6.86 (m, 2H), 6.86 – 6.81 (m, 2H), 3.99 (dd, J = 9.2, 7.3 Hz, 1H), 3.93 (dd, J = 9.2, 5.3 Hz, 1H), 3.83 (d, J = 9.0 Hz, 1H), 3.82 (s, 3H), 3.77 (dd, J = 8.8, 7.1 Hz, 1H), 3.44 (dd, J = 8.8, 6.2 Hz, 1H), 3.12 (s, 3H), 2.32 (dd, J = 13.5, 9.7 Hz, 1H), 2.25 – 2.16 (m, 2H), 2.10 – 2.00 (m, 1H).
^{13}C NMR (101 MHz, $CDCl_3$) δ 159.5, 140.5, 132.4, 128.7, 128.7, 128.4, 126.1, 114.0, 85.9, 73.5, 71.8, 56.5, 55.4, 52.0, 43.8, 39.7.
IR (ν_{max}, cm^{-1}) 2360 (m), 1610 (s), 1512 (s), 1456 (s), 1302 (s), 1248 (s), 1107 (s), 1036 (s), 928 (s), 831 (s), 756 (s).
HRMS (ESI/QTOF) m/z: [M + Na]$^+$ Calcd for $C_{20}H_{24}NaO_3^+$ 335.1618; Found 335.1623.

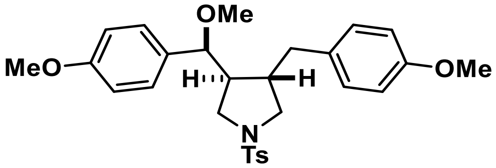

(3*S*,4*S*)-3-(methoxy(4-methoxyphenyl)methyl)-4-(4-methoxybenzyl)-1-tosylpyrrolidine (**24l-A**)
65% yield, > 20:1 dr, R_f = 0.35 (PE/EtOAc = 4:1), colorless oil.
^{1}H NMR (400 MHz, $CDCl_3$) δ 7.69 (d, J = 8.2 Hz, 2H), 7.33 (d, J = 8.0 Hz, 2H), 7.03 (d, J = 8.6 Hz, 2H), 6.84 (d, J = 8.6 Hz, 2H), 6.71 (s, 4H), 3.80 (s, 3H), 3.76 (s, 3H), 3.63 (d, J = 8.4 Hz, 1H), 3.41 (dd, J = 10.3, 5.3 Hz, 1H), 3.31 (dd, J = 10.3, 7.6 Hz, 1H), 3.20 (dd, J = 9.8, 7.0 Hz, 1H), 3.02 (s, 3H), 2.81 (dd, J = 9.8, 5.9 Hz, 1H), 2.45 (s, 3H), 2.17 – 1.95 (m, 4H).
^{13}C NMR (101 MHz, $CDCl_3$) δ 159.6, 158.1, 143.5, 133.2, 131.7, 131.7, 129.7, 129.6, 128.5, 127.9, 114.0, 113.91, 84.6, 56.5, 55.4, 55.4, 52.9, 50.5, 50.2, 42.1, 38.5, 21.7.
IR (ν_{max}, cm^{-1}) 2360 (s), 2335 (s), 1612 (m), 1512 (s), 1439 (m), 1342 (m), 1248 (s), 1163 (s), 1092 (m), 1034 (m), 833 (m), 717 (s).
HRMS (ESI/QTOF) m/z: [M + Na]$^+$ Calcd for $C_{28}H_{33}NNaO_5S^+$ 518.1972; Found 518.1974.

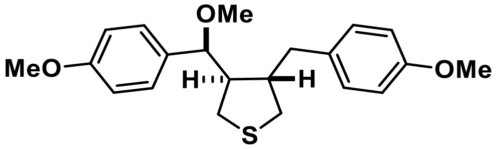

(3*R*,4*S*)-3-(methoxy(4-methoxyphenyl)methyl)-4-(4-methoxybenzyl)tetrahydrothiophene (**24m-A)**
53% yield, > 20:1 dr, R_f = 0.7 (PE/EtOAc = 8:1), White solid. M.p = 81– 83 °C.
^{1}H NMR (400 MHz, $CDCl_3$) δ 7.11 – 7.08 (m, 2H), 6.89 – 6.85 (m, 4H), 6.78 – 6.74 (m, 2H), 3.95 (d, J = 8.3 Hz, 1H), 3.83 (s, 3H), 3.77 (s, 3H), 3.18 (dd, J = 11.1, 4.1 Hz, 1H), 3.14 (s, 3H), 2.90 (dd, J = 11.1, 6.4 Hz, 1H), 2.77 (dd, J = 10.9, 6.3 Hz, 1H), 2.52 (dd, J = 11.0, 4.3 Hz, 1H), 2.48 (dd, J = 13.7, 7.0 Hz, 1H), 2.41 (dd, J = 13.7, 8.5 Hz, 1H), 2.27 (ddt, J = 8.3, 6.4, 4.1 Hz, 1H), 2.12 (dtt, J = 8.4, 6.8, 4.2 Hz, 1H).
^{13}C NMR (101 MHz, $CDCl_3$) δ 159.4, 158.1, 132.4, 132.3, 129.9, 128.6, 113.9, 113.8, 83.5, 56.9, 55.4, 55.4, 54.3, 46.3, 38.7, 35.6, 32.5.
IR (ν_{max}, cm^{-1}) 2360 (m), 2335 (m), 1612 (m), 1512 (s), 1458 (m), 1439 (m), 1246 (s), 1178 (m), 1093 (m), 1034 (m), 831 (m), 748 (w), 717 (s).
HRMS (APPI/LTQ-Orbitrap) m/z: [M + Na]$^+$ Calcd for $C_{21}H_{26}NaO_3S^+$ 381.1495; Found 381.1488.

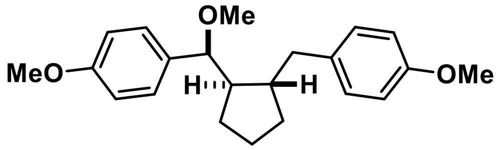

1-methoxy-4-(methoxy((1*R*,2*S*)-2-(4-methoxybenzyl)cyclopentyl)methyl)benzene (**24n-A**)
56% yield, > 20:1 dr, R_f = 0.5 (PE/EtOAc = 10:1), colorless oil.
^{1}H NMR (400 MHz, $CDCl_3$) δ 7.17 (d, *J* = 8.6 Hz, 2H), 6.88 (d, *J* = 8.7 Hz, 2H), 6.83 (d, *J* = 8.6 Hz, 2H), 6.74 (d, *J* = 8.7 Hz, 2H), 3.81 (s, 3H), 3.76 (d, *J* = 7.0 Hz, 1H), 3.76 (s, 3H), 3.14 (s, 3H), 2.18 (dd, *J* = 13.3, 5.8 Hz, 1H), 2.13 (dd, *J* = 13.3, 8.5 Hz, 1H), 1.89 – 1.66 (m, 3H), 1.66 – 1.48 (m, 3H), 1.29 – 1.19 (m, 2H).
^{13}C NMR (101 MHz, $CDCl_3$) δ 159.1, 157.7, 134.2, 133.8, 129.9, 128.6, 113.7, 113.6, 86.8, 56.8, 55.4, 55.4, 52.0, 43.8, 41.1, 32.7, 29.3, 24.7.
IR (ν_{max}, cm^{-1}) 1610 (m), 1510 (s), 1460 (m), 1298 (m), 1244 (s), 1174 (s), 1036 (s), 833 (s), 756 (s).
HRMS (APCI/QTOF) m/z: $[M + Na]^+$ Calcd for $C_{22}H_{28}NaO_3^+$ 363.1931; Found 363.1931.

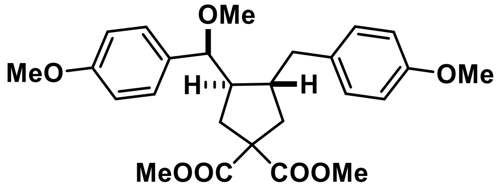

dimethyl(3*R*,4*R*)-3-(methoxy(4-methoxyphenyl)methyl)-4-(4-methoxybenzyl)cyclopentane-1,1-dicarboxylate (**24o-A**)
63% yield, > 20:1 dr, R_f = 0.4 (PE/EtOAc = 5:1), colorless oil.
^{1}H NMR (400 MHz, $CDCl_3$) δ 7.18 (d, *J* = 8.6 Hz, 2H), 6.89 (d, *J* = 8.1 Hz, 4H), 6.76 (d, *J* = 8.5 Hz, 2H), 3.87 (d, *J* = 6.5 Hz, 1H), 3.80 (s, 3H), 3.76 (s, 3H), 3.69 (s, 3H), 3.69 (s, 3H), 3.14 (s, 3H), 2.41 (dd, *J* = 16.9, 8.5 Hz, 1H), 2.37 (dd, *J* = 16.9, 8.7 Hz, 1H), 2.26 (dd, *J* = 13.2, 7.5 Hz, 1H), 2.19 – 2.15 (m, 2H), 2.12 – 1.98 (m, 2H), 1.91 (dd, *J* = 13.2, 8.1 Hz, 1H).
^{13}C NMR (101 MHz, $CDCl_3$) δ 173.1, 173.0, 159.3, 157.9, 133.2, 133.1, 129.8, 128.4, 113.9, 113.8, 85.5, 58.9, 56.8, 55.4, 55.4, 52.8, 52.8, 51.5, 42.8, 40.1, 40.0, 37.0.
IR (ν_{max}, cm^{-1}) 2360 (s), 2337 (s), 2023 (m), 1734 (m), 1512 (m), 1248 (m), 1032 (w), 717 (m).
HRMS (ESI/QTOF) m/z: $[M + Na]^+$ Calcd for $C_{26}H_{32}NaO_7^+$ 479.2040; Found 479.2050.

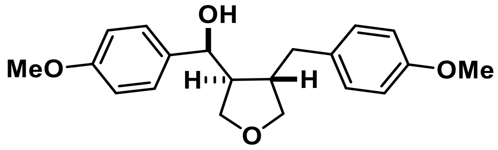

((3*S*,4*S*)-4-(4-methoxybenzyl)tetrahydrofuran-3-yl)(4-methoxyphenyl)methanol (**24p-A**)
38% yield, > 20:1 dr, R_f = 0.1 (PE/EtOAc = 5:1), colorless oil.
^{1}H NMR (400 MHz, $CDCl_3$) δ 7.23 – 7.16 (m, 2H), 6.89 – 6.83 (m, 2H), 6.82 – 6.77 (m, 2H), 6.76 – 6.69 (m, 2H), 4.42 (d, *J* = 8.4 Hz, 1H), 3.99 (dd, *J* = 9.2, 5.4 Hz, 1H), 3.95 (dd, *J* = 9.2, 7.0 Hz, 1H), 3.84 – 3.78 (m, 1H), 3.81 (s, 3H), 3.76 (s, 3H), 3.44 (dd, *J* = 8.8, 6.1 Hz, 1H), 2.34 (dd, *J* = 13.6, 9.3 Hz, 1H), 2.29 – 2.19 (m, 2H), 2.13 – 2.04 (m, 1H), 2.00 (s, 1H).
^{13}C NMR (101 MHz, $CDCl_3$) δ 159.4, 158.0, 135.5, 132.5, 129.6, 127.8, 114.0, 113.9, 76.4, 73.6, 71.1, 55.4, 55.4, 52.3, 44.0, 38.8.
IR (ν_{max}, cm^{-1}) 1611 (m), 1511 (s), 1464 (w), 1439 (w), 1302 (m), 1245 (s), 1176 (m), 1034 (m), 830 (m), 757 (m).
HRMS (ESI/QTOF) m/z: $[M + Na]^+$ Calcd for $C_{20}H_{24}NaO_4^+$ 351.1567; Found 351.1566.

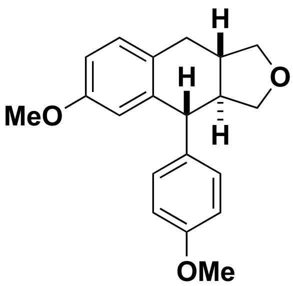

(3a*S*,4*R*,9a*S*)-6-methoxy-4-(4-methoxyphenyl)-1,3,3a,4,9,9a-hexahydronaphtho[2,3-c]furan (**19b**)
92% yield, > 20:1 dr, R_f = 0.5 (PE/EtOAc = 8:1), colorless oil.

^{1}H NMR (400 MHz, $CDCl_3$) δ 7.09 – 7.03 (m, 3H), 6.84 (d, *J* = 8.7 Hz, 2H), 6.71 (dd, *J* = 8.4, 2.6 Hz, 1H), 6.34 (d, *J* = 2.3 Hz, 1H), 4.20 (t, *J* = 7.3 Hz, 1H), 3.82-3.76 (m, 2H), 3.81 (s, 3H), 3.62 (s, 3H), 3.56 – 3.49 (m, 2H), 3.04 (dd, *J* = 15.6, 4.1 Hz, 1H), 2.73 (dd, *J* = 15.4, 10.6 Hz, 1H), 2.32 – 2.17 (m, 2H).
^{13}C NMR (101 MHz, $CDCl_3$) δ 158.5, 158.0, 141.7, 136.7, 130.2, 129.6, 128.6, 115.1, 114.2, 112.2, 73.4, 72.5, 55.4, 55.3, 50.9, 49.9, 42.5, 32.2.
IR (ν_{max}, cm^{-1}) 2360 (s), 1610 (s), 1510 (s), 1495 (s), 1462 (s), 1284 (s), 1250 (s), 1178 (s), 1032 (s), 899 (s), 717 (s).
HRMS (APPI/LTQ-Orbitrap) m/z: $[M]^+$ Calcd for $C_{20}H_{22}O_3^+$ 310.1563; Found 310.1571.

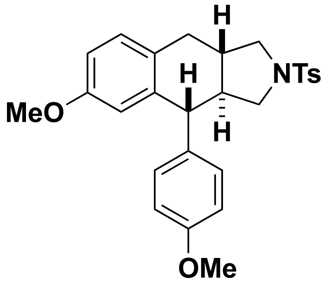

(3a*S*,4*R*,9a*S*)-6-methoxy-4-(4-methoxyphenyl)-2-tosyl-2,3,3a,4,9,9a-hexahydro-1H-benzo[f]isoindole (**19c**)
89% yield, > 20:1 dr, R_f = 0.1 (PE/EtOAc = 10:1), white solid. M.p = 181– 182 °C.
^{1}H NMR (400 MHz, $CDCl_3$) δ 7.67 (d, *J* = 8.2 Hz, 2H), 7.29 (d, *J* = 8.0 Hz, 2H), 7.00 (d, *J* = 8.5 Hz, 1H), 6.97 (d, *J* = 8.6 Hz, 2H), 6.83 (d, *J* = 8.7 Hz, 2H), 6.67 (dd, *J* = 8.4, 2.6 Hz, 1H), 6.26 (d, *J* = 2.3 Hz, 1H), 3.82 (s, 3H), 3.77 (dd, *J* = 9.6, 6.9 Hz, 1H), 3.64 (d, *J* = 9.9 Hz, 1H), 3.59 (s, 3H), 3.35 (dd, *J* = 9.7, 6.8 Hz, 1H), 3.04 – 2.93 (m, 3H), 2.62 (dd, *J* = 15.2, 11.1 Hz, 1H), 2.42 (s, 3H), 2.09 – 1.93 (m, 2H).
^{13}C NMR (101 MHz, $CDCl_3$) δ 158.7, 158.1, 143.4, 140.8, 135.7, 134.8, 130.1, 129.8, 129.6, 127.7, 127.4, 115.0, 114.3, 112.4, 55.4, 55.3, 53.8, 53.0, 50.3, 49.5, 41.1, 32.8, 21.7.
IR (ν_{max}, cm^{-1}) 1512 (m), 1265 (m), 1159 (m), 1012 (m), 810 (m), 756 (s), 737 (s), 717 (s).
HRMS (ESI/QTOF) m/z: $[M + Na]^+$ Calcd for $C_{27}H_{29}NNaO_4S^+$ 486.1710; Found 486.1722.

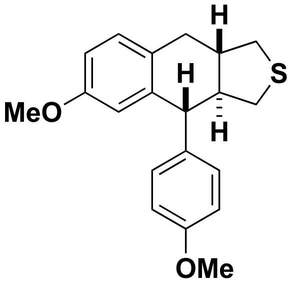

(3a*S*,4*R*,9a*S*)-6-methoxy-4-(4-methoxyphenyl)-1,3,3a,4,9,9a-hexahydronaphtho[2,3-c]thiophene (**19d**)
92% yield, 10:1 dr, R_f = 0.7 (PE/EtOAc = 8:1), colorless oil.
^{1}H NMR (400 MHz, $CDCl_3$) δ 7.07 – 7.04 (m, 3H), 6.85 (d, *J* = 8.7 Hz, 2H), 6.70 (dd, *J* = 8.4, 2.6 Hz, 1H), 6.28 (d, *J* = 2.1 Hz, 1H), 3.81 (s, 3H), 3.76 (d, *J* = 10.4 Hz, 1H), 3.61 (s, 3H), 3.22 – 3.11 (m, 2H), 2.81 – 2.72 (m, 3H), 2.67 (t, *J* = 10.3 Hz, 1H), 2.25 – 2.07 (m, 2H).
^{13}C NMR (101 MHz, $CDCl_3$) δ 158.5, 158.0, 141.2, 136.8, 129.9, 129.7, 128.3, 114.9, 114.2, 112.2, 55.4, 55.3, 53.7, 53.0, 45.3, 37.8, 37.2, 35.6.
IR (ν_{max}, cm^{-1}) 2158 (s), 1982 (m), 1610 (m), 1506 (s), 1460 (s), 1263 (s), 1012 (s), 771 (s), 758 (s), 733 (s), 717 (s).
HRMS (APCI/QTOF) m/z: $[M + H]^+$ Calcd for $C_{20}H_{23}O_2S^+$ 327.1413; Found 327.1405.

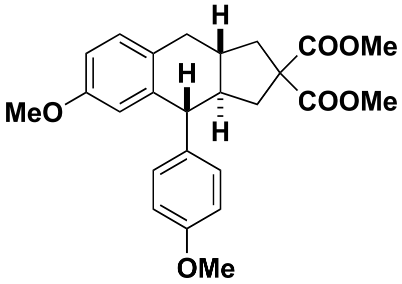

dimethyl (3a*R*,4*R*,9a*R*)-6-methoxy-4-(4-methoxyphenyl)-1,3,3a,4,9,9a-hexahydro-2H-cyclopenta[b]naphthalene-2,2-dicarboxylate (**19e**)
93% yield, > 20:1 dr, R_f = 0.3 (PE/EtOAc = 10:1), colorless oil.

^{1}H NMR (400 MHz, $CDCl_3$) δ 7.06 – 7.02 (m, 3H), 6.84 (d, *J* = 8.7 Hz, 2H), 6.68 (dd, *J* = 8.4, 2.6 Hz, 1H), 6.29 (d, *J* = 2.3 Hz, 1H), 3.81 (s, 3H), 3.73 (s, 3H), 3.67 (s, 3H), 3.67 – 3.65 (m, 1H), 3.60 (s, 3H), 3.04 (dd, *J* = 15.7, 3.2 Hz, 1H), 2.74 – 2.61 (m, 2H), 2.36 – 2.29 (m, 1H), 1.98 – 1.87 (m, 4H).
^{13}C NMR (101 MHz, $CDCl_3$) δ 173.5, 173.3, 158.3, 157.8, 141.8, 136.9, 129.9, 129.9, 129.0, 115.0, 114.1, 112.0, 58.4, 55.4, 55.3, 52.9, 52.9, 52.7, 50.4, 42.1, 41.1, 39.9, 35.3.
IR (ν_{max}, cm^{-1}) 1730 (s), 1610 (m), 1510 (m), 1437 (m), 1248 (s), 1196 (m), 1174 (s), 1153 (m), 1034 (s), 812 (m), 735 (m).
HRMS (ESI/QTOF) m/z: $[M + Na]^+$ Calcd for $C_{25}H_{28}NaO_6^+$ 447.1778; Found 447.1779.

1.6. Crystallographic data

22a-A

Submitted by: **Jia-Chen Xiang**

Solved by: **Farzaneh Fadaei Tirani**

Sample ID: **XJC-674-2a**

R_1=2.68%

Supplementary Figure 1: X-ray of compound **22a-A**

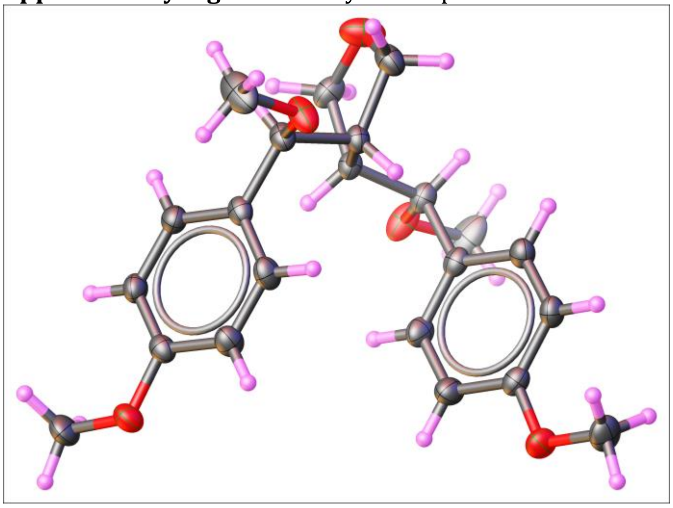

Experimental. Single clear colourless prism crystals of **xjc-674-2a** were used as supplied. A suitable crystal with dimensions of 0.61 × 0.41 × 0.30 mm³ was selected and mounted on a SuperNova, Dual, Cu at home/near, Atlas diffractometer. The crystal was kept at a steady T = 140.00(10) K during data collection. The structure was solved with the **ShelXT** 2018/2 (Sheldrick, 2018) solution program using dual methods and by using **Olex2** (Dolomanov et al., 2009) as the graphical interface. The model was refined with **ShelXL** 2018/3 (Sheldrick, 2015) using full matrix least squares minimisation on $|F|^2$.

Crystal Data. $C_{22}H_{28}O_5$, M_r = 372.44, tetragonal, $P4_12_12$ (No. 92), a = 8.25505(8) Å, b = 8.25505(8) Å, c = 29.4002(4) Å, $\alpha = \beta = \gamma$ = 90°, V = 2003.50(5) Å³, T = 140.00(10) K, Z = 4, Z' = 0.5, μ(Cu K_α) = 0.702, 13970 reflections measured, 1988 unique (R_{int} = 0.0150) which were used in all calculations. The final wR_2 was 0.0736 (all data) and R_1 was 0.0268 (I > 2(I)).

Supplementary Table 8: Crystal data for compound **22a-A**

Formula	$C_{22}H_{28}O_5$
$D_{calc.}$/ g cm^{-3}	1.235
μ/mm^{-1}	0.702
Formula Weight	372.44
Colour	clear colourless
Shape	prism
Size/mm³	0.61×0.41×0.30
T/K	140.00(10)
Crystal System	tetragonal
Flack Parameter	0.5(2)
Hooft Parameter	0.52(2)
Space Group	$P4_12_12$
a/Å	8.25505(8)
b/Å	8.25505(8)
c/Å	29.4002(4)
α/°	90
β/°	90
γ/°	90
V/Å³	2003.50(5)
Z	4
Z'	0.5
Wavelength/Å	1.54184
Radiation type	Cu $K\alpha$
Θ_{min}/°	5.566
Θ_{max}/°	72.711
Measured Refl's.	13970
Ind't Refl's	1988
Refl's with I > 2(I)	1982
R_{int}	0.0150
Parameters	127
Restraints	0
Largest Peak/e Å^{-3}	0.192
Deepest Hole/e Å^{-3}	-0.149
GooF	1.097
wR_2 (all data)	0.0736
wR_2	0.0735
R_1 (all data)	0.0268
R_1	0.0268

Supplementary Table 9: Structure Quality Indicators

**Reflections:**	d min (Cu) CIF 0.81	I/σ CIF 144.9	Rint CIF 1.50%	complete 100% (IUCr) 100%
**Refinement:**	Shift CIF 0.001	Max Peak CIF 0.2	Min Peak CIF -0.1	GooF CIF 1.097

A clear colourless prism-shaped crystal with dimensions of 0.61 × 0.41 × 0.30 mm^3 was mounted. Data were collected using a SuperNova, Dual, Cu at home/near, Atlas diffractometer operating at T = 140.00(10) K.

Data were measured using ω scans using Cu K_α radiation. The diffraction pattern was indexed and the total number of runs and images was based on the strategy calculation from the program **CrysAlisPro** (Rigaku, V1.171.40.67a, 2019). The maximum resolution achieved was Θ = 72.711° (0.81 Å).

The diffraction pattern was indexed and the total number of runs and images was based on the strategy calculation from the program **CrysAlisPro** (Rigaku, V1.171.40.67a, 2019). The unit cell was refined using **CrysAlisPro** (Rigaku, V1.171.40.67a, 2019) on 9211 reflections, 66% of the observed reflections.

Data reduction, scaling and absorption corrections were performed using **CrysAlisPro** (Rigaku, V1.171.40.67a, 2019). The final completeness is 99.90 % out to 72.711° in Θ. An analytical absorption correction was performed using CrysAlisPro 1.171.40.67a (Rigaku Oxford Diffraction, 2019). Analytical numeric absorption correction using a multifaceted crystal model based on expressions derived by R.C. Clark & J.S. Reid. (Clark, R. C. & Reid, J. S. (1995). Acta Cryst. A51, 887-897) Empirical absorption correction using spherical harmonics as implemented in SCALE3 ABSPACK scaling algorithm. The absorption coefficient μ of this material is 0.702 mm^{-1} at this wavelength (λ = 1.54184Å) and the minimum and maximum transmissions are 0.770 and 0.842.

The structure was solved and the space group $P4_12_12$ (# 92) determined by the ShelXT 2018/2 (Sheldrick, 2018) structure solution program using using dual methods and refined by full matrix least squares minimisation on $|F|^2$ using version 2018/3 of ShelXL 2018/3 (Sheldrick, 2015). All non-hydrogen atoms were refined anisotropically. Hydrogen atom positions were calculated geometrically and refined using the riding model.

This structure was refined as a 2-component inversion twin.

The value of Z' is 0.5. This means that only half of the formula unit is present in the asymmetric unit, with the other half consisting of symmetry equivalent atoms.

The Flack parameter was refined to 0.5(2). Determination of absolute structure using Bayesian statistics on Bijvoet differences using the Olex2 results in 0.52(2). Note: The Flack parameter is used to determine chirality of the crystal studied, the value should be near 0, a value of 1 means that the stereochemistry is wrong and the model should be inverted. A value of 0.5 means that the crystal consists of a racemic mixture of the two enantiomers.

Citations

CrysAlis^{Pro} Software System, Rigaku Oxford Diffraction, (2019).

Sheldrick, G.M., ShelXT-Integrated space-group and crystal-structure determination, *Acta Cryst.*, (2015), **A71**, 3-8.

Sheldrick, G.M., Crystal structure refinement with ShelXL, *Acta Cryst.*, (2015), **C71**, 3-8.

O.V. Dolomanov and L.J. Bourhis and R.J. Gildea and J.A.K. Howard and H. Puschmann, Olex2: A complete structure solution, refinement and analysis program, *J. Appl. Cryst.*, (2009), **42**, 339-341.

Supplementary Figure 2: Images of the Crystal on the Diffractometer

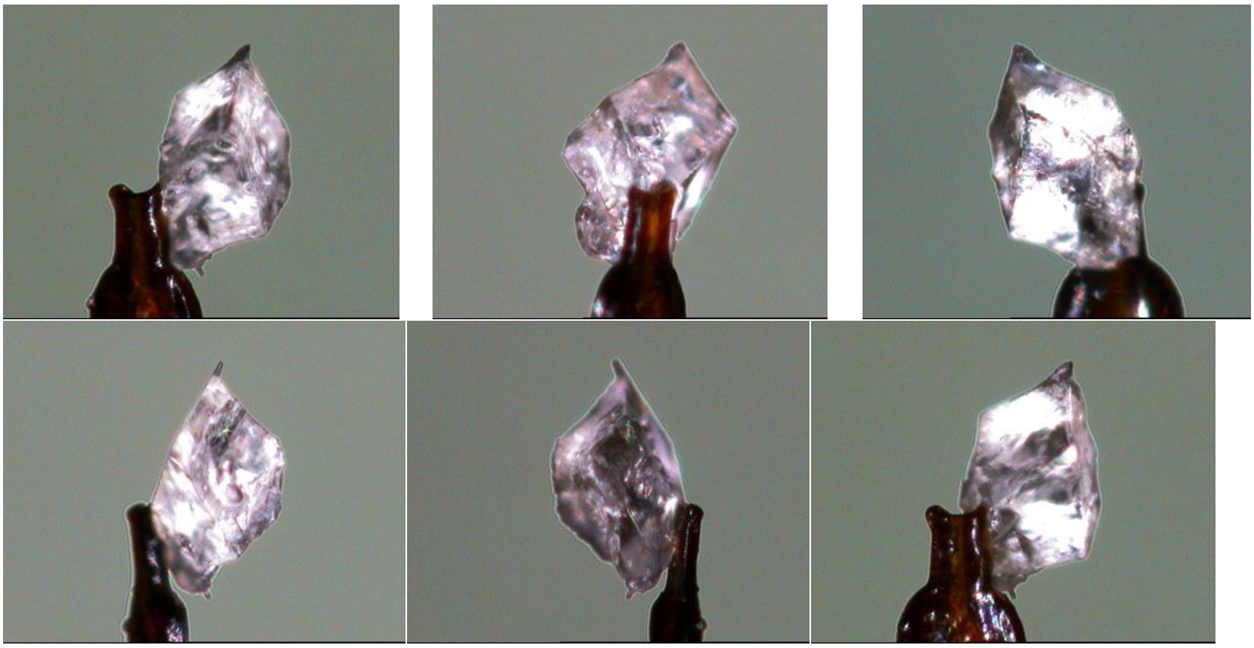

Supplementary Figure 3: Data Plots: Diffraction Data

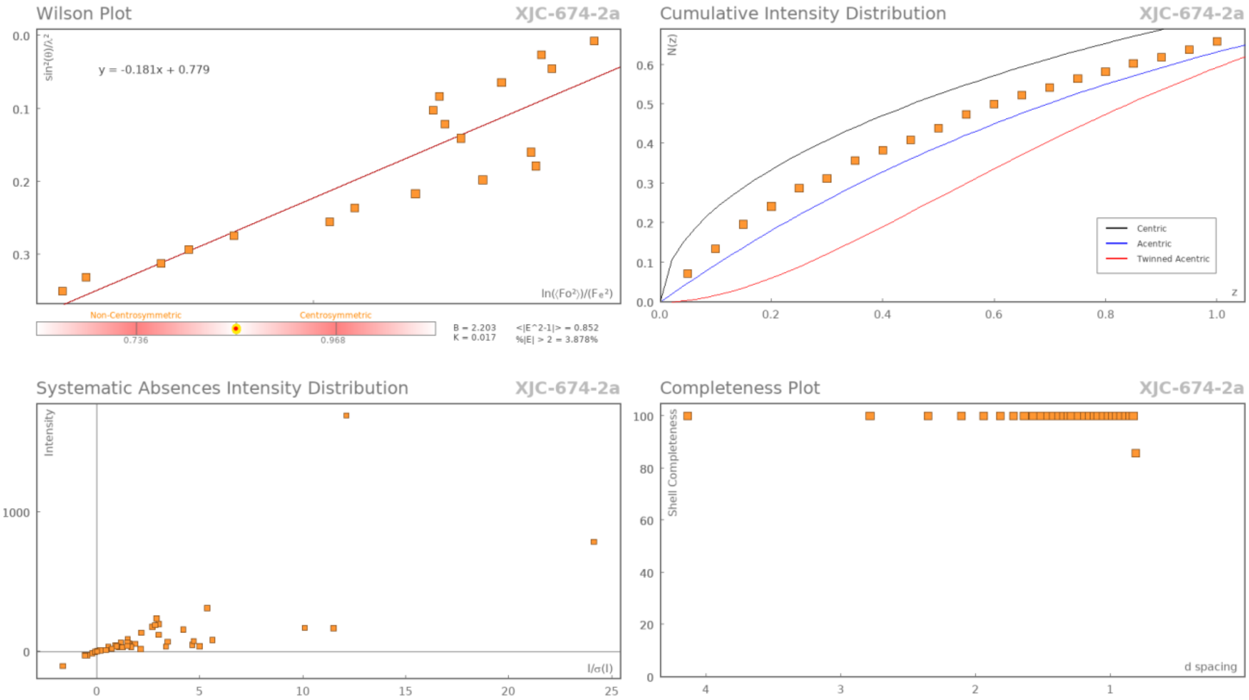

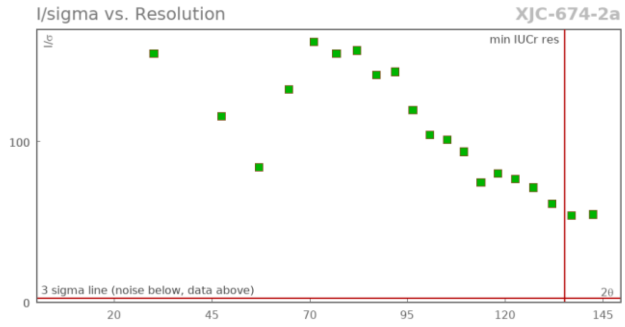

Supplementary Figure 4: Data Plots: Refinement and Data

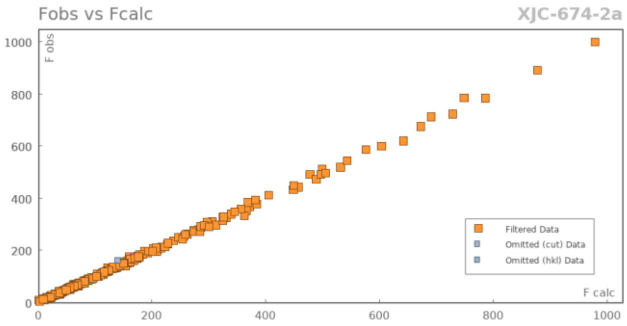

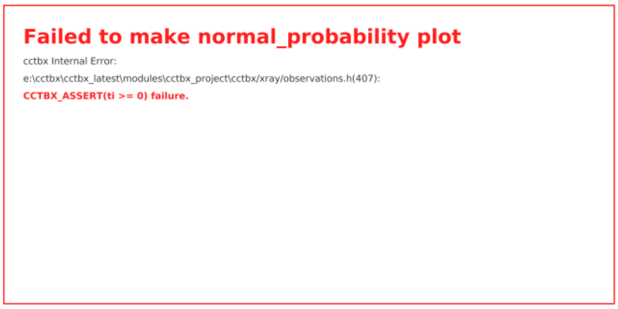

Supplementary Table 10: Reflection Statistics

Total reflections (after filtering)	14039	Unique reflections	1988
Completeness	0.996	Mean I/σ	107.3
hkl_{max} collected	(9, 9, 36)	hkl_{min} collected	(-10, -9, -26)
hkl_{max} used	(7, 10, 36)	hkl_{min} used	(-6, 0, 0)
Lim d_{max} collected	100.0	Lim d_{min} collected	0.77
d_{max} used	9.8	d_{min} used	0.81
Friedel pairs	1279	Friedel pairs merged	0
Inconsistent equivalents	10	R_{int}	0.015
R_{sigma}	0.0069	Intensity transformed	0
Omitted reflections	0	Omitted by user (OMIT hkl)	5
Multiplicity	(5422, 2321, 819, 262, 58, 30)	Maximum multiplicity	17
Removed systematic absences	64	Filtered off (Shel/OMIT)	0

Supplementary Table 11: Fractional Atomic Coordinates (×10⁴) and Equivalent Isotropic Displacement Parameters (Å²×10³) for **xjc-674-2a**. U_{eq} is defined as 1/3 of the trace of the orthogonalised U_{ij}.

**Atom**	**x**	**y**	**z**	U_{eq}
O1	4110.7(14)	14110.7(14)	5000	46.9(5)
O2	4478.9(12)	9405.3(13)	5187.7(3)	36.2(3)
O3	-1909.8(12)	7406.7(13)	6235.2(3)	32.7(3)
C1	4240.9(19)	12590.2(18)	4777.7(5)	33.0(3)
C2	2839.2(15)	11553.1(16)	4947.5(4)	23.3(3)
C3	3350.8(16)	10559.6(17)	5363.1(4)	25.8(3)
C4	5375(2)	8610(2)	5535.3(6)	50.9(5)
C5	1951.4(16)	9748.9(17)	5603.9(4)	24.6(3)
C6	1124.7(19)	8461.4(17)	5402.3(5)	30.0(3)
C7	-149.3(19)	7703.9(18)	5618.4(5)	30.7(3)
C8	-647.7(16)	8243.0(17)	6046.3(4)	25.9(3)
C9	130.5(17)	9538.2(17)	6249.5(4)	27.7(3)
C10	1439.6(16)	10264.5(18)	6029.2(4)	27.4(3)

Atom	x	y	z	U_{eq}
C11	-2557(2)	8017(2)	6651.8(5)	39.5(4)

Supplementary Table 12: Anisotropic Displacement Parameters (×10[4]) for **xjc-674-2a**. The anisotropic displacement factor exponent takes the form: $-2\pi^2[h^2a^{*2} \times U_{11} + ... + 2hka^* \times b^* \times U_{12}]$

Atom	U_{11}	U_{22}	U_{33}	U_{23}	U_{13}	U_{12}
O1	34.5(5)	34.5(5)	71.9(12)	-4.8(6)	4.8(6)	-7.1(7)
O2	29.8(5)	41.7(6)	37.3(5)	14.5(5)	9.4(4)	17.0(4)
O3	30.5(5)	36.7(6)	30.8(5)	1.7(4)	7.0(4)	-4.3(4)
C1	32.2(7)	34.0(7)	33.0(7)	8.4(6)	6.9(6)	3.5(6)
C2	24.4(6)	25.4(7)	20.2(6)	1.7(5)	1.3(5)	4.8(5)
C3	22.8(6)	29.9(7)	24.6(6)	4.3(5)	1.6(5)	5.9(5)
C4	39.9(9)	56.3(11)	56.6(10)	28.9(9)	5.8(8)	22.0(8)
C5	24.2(6)	26.7(7)	23.0(6)	5.2(5)	1.0(5)	6.4(5)
C6	37.0(8)	27.7(7)	25.3(6)	-1.5(5)	7.6(6)	3.1(6)
C7	36.3(8)	26.5(7)	29.1(6)	-2.5(5)	4.1(6)	-2.0(6)
C8	24.1(6)	27.8(7)	25.7(6)	4.9(5)	2.8(5)	3.5(5)
C9	27.5(7)	35.0(7)	20.6(5)	-0.9(5)	1.4(5)	1.9(5)
C10	26.6(7)	32.4(7)	23.1(6)	0.8(5)	-1.6(5)	0.2(5)
C11	32.6(8)	55.1(10)	30.7(7)	0.2(7)	10.6(6)	-5.7(7)

Supplementary Table 13: Bond Lengths in Å for **xjc-674-2a**.

Atom	Atom	Length/Å
O1	C1	1.4192(18)
O1	C1[1]	1.4193(18)
O2	C3	1.4285(16)
O2	C4	1.4222(18)
O3	C8	1.3677(16)
O3	C11	1.4282(18)
C1	C2	1.5235(19)
C2	C2[1]	1.533(2)
C2	C3	1.5309(17)
C3	C5	1.5112(19)
C5	C6	1.395(2)
C5	C10	1.3867(18)
C6	C7	1.379(2)
C7	C8	1.3963(19)
C8	C9	1.3831(19)
C9	C10	1.395(2)

----
[1]-1+y,1+x,1-z

Supplementary Table 14: Bond Angles in ° for **xjc-674-2a**.

Atom	Atom	Atom	Angle/°
C1	O1	C1[1]	110.24(16)
C4	O2	C3	112.81(11)
C8	O3	C11	117.07(12)
O1	C1	C2	106.77(12)
C1	C2	C2[1]	101.70(8)
C1	C2	C3	110.67(11)
C3	C2	C2[1]	113.67(12)
O2	C3	C2	104.43(10)
O2	C3	C5	111.84(11)
C5	C3	C2	113.60(11)
C6	C5	C3	120.81(11)
C10	C5	C3	121.30(12)
C10	C5	C6	117.89(13)
C7	C6	C5	121.53(12)
C6	C7	C8	119.69(13)
O3	C8	C7	115.43(12)
O3	C8	C9	124.65(12)
C9	C8	C7	119.92(13)
C8	C9	C10	119.42(12)
C5	C10	C9	121.52(13)

----
[1]-1+y,1+x,1-z

Supplementary Table 15: Torsion Angles in ° for **xjc-674-2a**.

Atom	Atom	Atom	Atom	Angle/°
O1	C1	C2	C2[1]	29.46(15)
O1	C1	C2	C3	-91.62(12)
O2	C3	C5	C6	-48.04(16)
O2	C3	C5	C10	132.46(13)

Atom	Atom	Atom	Atom	Angle/°
O3	C8	C9	C10	178.01(12)
C1^{1}	O1	C1	C2	-11.61(6)
C1	C2	C3	O2	-68.88(14)
C1	C2	C3	C5	169.01(11)
C2^{1}	C2	C3	O2	177.41(7)
C2^{1}	C2	C3	C5	55.30(12)
C2	C3	C5	C6	69.87(16)
C2	C3	C5	C10	-109.63(14)
C3	C5	C6	C7	179.69(13)
C3	C5	C10	C9	178.88(12)
C4	O2	C3	C2	166.38(13)
C4	O2	C3	C5	-70.36(16)
C5	C6	C7	C8	0.9(2)
C6	C5	C10	C9	-0.6(2)
C6	C7	C8	O3	-179.41(13)
C6	C7	C8	C9	0.4(2)
C7	C8	C9	C10	-1.8(2)
C8	C9	C10	C5	1.9(2)
C10	C5	C6	C7	-0.8(2)
C11	O3	C8	C7	-174.10(13)
C11	O3	C8	C9	6.1(2)

----

^{1}-1+y,1+x,1-z

Supplementary Table 16: Hydrogen Fractional Atomic Coordinates (×10^4) and Equivalent Isotropic Displacement Parameters (Å^2×10^3) for **xjc-674-2a**. U_{eq} is defined as 1/3 of the trace of the orthogonalised U_{ij}.

Atom	x	y	z	U_{eq}
H1A	5288.94	12070.46	4851.85	40
H1B	4172.99	12731.49	4443.86	40
H2	2451.66	10819.03	4699.77	28
H3	3923.76	11285.84	5582.67	31
H4A	4628.32	8039.98	5739	76
H4B	5992.73	9413.06	5709.22	76
H4C	6122.55	7829.7	5397.27	76
H6	1447.12	8098.37	5109.38	36
H7	-685.56	6819.19	5476.88	37
H9	-223.21	9929.93	6536.66	33
H10	1992.86	11132.14	6173.8	33
H11A	-1706.55	8027.44	6884.32	59
H11B	-3449.87	7321.63	6752.35	59
H11C	-2958.8	9121.6	6604.53	59

21a-A

Submitted by: **Jia-Chen Xiang**

Solved by: **Farzaneh Fadaei Tirani**

Sample ID: **XJC-937**

R_1=3.83%

Supplementary Figure 5: X-ray of compound **21a-A**

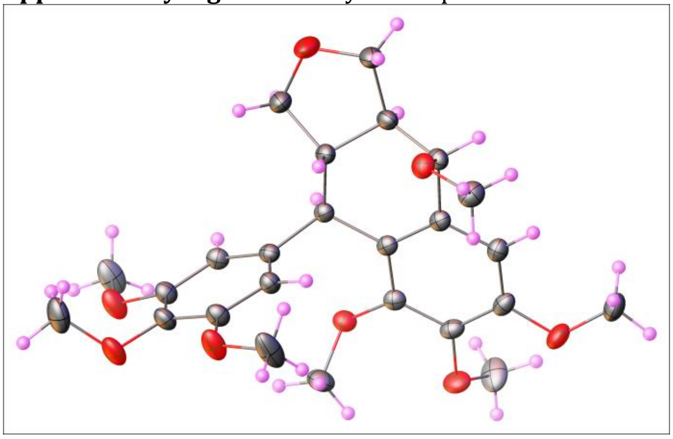

Experimental. Single colourless prism crystals of **xjc-937** were used as supplied. A suitable crystal with dimensions 0.97 × 0.79 × 0.53 mm³ was selected and mounted on a SuperNova, Dual, Cu at home/near, AtlasS2 diffractometer. The crystal was kept at a steady T = 140.01(10) K during data collection. The structure was solved with the ShelXT 2018/2 (Sheldrick, 2015) solution program using dual methods and by using Olex2 (Dolomanov et al., 2009) as the graphical interface. The model was refined with ShelXL 2018/3 (Sheldrick, 2015) using full matrix least squares minimisation on $\boldsymbol{F}^2$.

Crystal Data. $C_{25}H_{32}O_8$, M_r = 460.50, monoclinic, $P2_1/n$ (No. 14), a = 11.43471(7) Å, b = 12.70325(8) Å, c = 15.90691(11) Å, β = 91.5400(6)°, α = γ = 90°, V = 2309.77(3) Å³, T = 140.01(10) K, Z = 4, Z' = 1, μ(Cu K_α) = 0.814, 24675 reflections measured, 4790 unique (R_{int} = 0.0129) which were used in all calculations. The final wR_2 was 0.0965 (all data) and R_1 was 0.0383 (I≥2 σ(I)).

Supplementary Table 17: Crystal data for compound **21a-A**

Formula	$C_{25}H_{32}O_8$
$D_{calc.}$/ g cm^{-3}	1.324
μ/mm^{-1}	0.814
Formula Weight	460.50
Colour	colourless
Shape	prism
Size/mm³	0.97×0.79×0.53
T/K	140.01(10)
Crystal System	monoclinic
Space Group	$P2_1/n$
a/Å	11.43471(7)
b/Å	12.70325(8)
c/Å	15.90691(11)
α/°	90
β/°	91.5400(6)
γ/°	90
V/Å³	2309.77(3)
Z	4
Z'	1
Wavelength/Å	1.54184
Radiation type	CuK_α
Θ_{min}/°	4.455
Θ_{max}/°	75.954
Measured Refl's.	24675
Indep't Refl's	4790
Refl's I≥2σ(I)	4716
R_{int}	0.0129
Parameters	306
Restraints	0
Largest Peak/e Å^{-3}	0.280
Deepest Hole/e Å^{-3}	-0.165
GooF	1.070
wR_2 (all data)	0.0965
wR_2	0.0960
R_1 (all data)	0.0392
R_1	0.0383

Supplementary Table 18: Structure Quality Indicators

**Reflections:**	d min (Cu) CIF 0.79	I/σ(I) CIF 120.5	Rint CIF 1.29%	complete 100% (IUCr) 100%
**Refinement:**	Shift CIF 0.000	Max Peak CIF 0.3	Min Peak CIF -0.2	GooF CIF 1.070

A colourless prism-shaped crystal with dimensions 0.97 × 0.79 × 0.53 mm^3 was mounted. Data were collected using a SuperNova, Dual, Cu at home/near, AtlasS2 diffractometer operating at T = 140.01(10) K.

Data were measured using ω scans using Cu K_α radiation. The diffraction pattern was indexed and the total number of runs and images was based on the strategy calculation from the program CrysAlisPro (Rigaku, V1.171.41.78a, 2020). The maximum resolution achieved was Θ = 75.954° (0.79 Å).

The diffraction pattern was indexed and the total number of runs and images was based on the strategy calculation from the program CrysAlisPro (Rigaku, V1.171.41.78a, 2020). The unit cell was refined using CrysAlisPro (Rigaku, V1.171.41.78a, 2020) on 19878 reflections, 81% of the observed reflections.

Data reduction, scaling and absorption corrections were performed using CrysAlisPro (Rigaku, V1.171.41.78a, 2020). The final completeness is 100.00 % out to 75.954° in Θ. A Gaussian absorption correction was performed using CrysAlisPro 1.171.41.78a (Rigaku Oxford Diffraction, 2020) Numerical absorption correction based on Gaussian integration over a multifaceted crystal model. Empirical absorption correction using spherical harmonics as implemented in SCALE3 ABSPACK scaling algorithm. The absorption coefficient μ of this material is 0.814 mm^{-1} at this wavelength (λ = 1.54184Å) and the minimum and maximum transmissions are 0.226 and 1.000.

The structure was solved and the space group $P2_1/n$ (# 14) determined by the ShelXT 2018/2 (Sheldrick, 2015) structure solution program using using dual methods and refined by full matrix least squares minimisation on $\boldsymbol{F}^2$ using version 2018/3 of ShelXL 2018/3 (Sheldrick, 2015). All non-hydrogen atoms were refined anisotropically. Hydrogen atom positions were calculated geometrically and refined using the riding model.

There is a single molecule in the asymmetric unit, which is represented by the reported sum formula. In other words: Z is 4 and Z' is 1.

Citations

CrysAlis^{Pro} Software System, Rigaku Oxford Diffraction, (2020).

Sheldrick, G.M., ShelXT-Integrated space-group and crystal-structure determination, *Acta Cryst.*, (2015), **A71**, 3-8.

Sheldrick, G.M., Crystal structure refinement with ShelXL, *Acta Cryst.*, (2015), **C71**, 3-8.

O.V. Dolomanov and L.J. Bourhis and R.J. Gildea and J.A.K. Howard and H. Puschmann, Olex2: A complete structure solution, refinement and analysis program, *J. Appl. Cryst.*, (2009), **42**, 339-341.

Supplementary Figure 6: Images of the Crystal on the Diffractometer

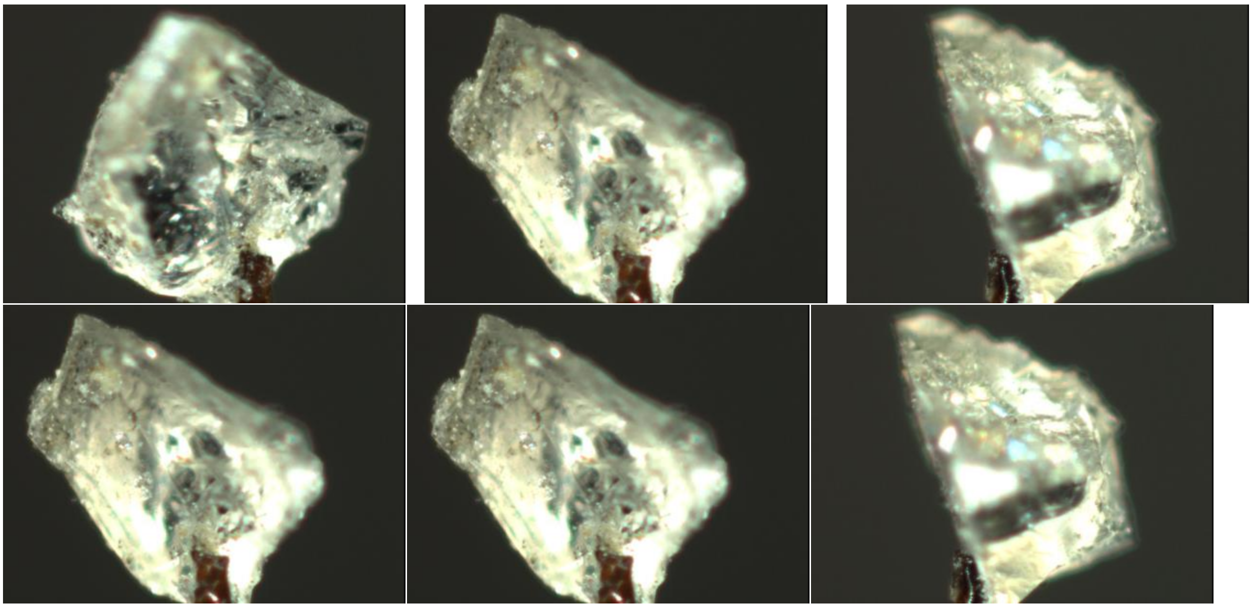

Supplementary Figure 7: Data Plots: Diffraction Data

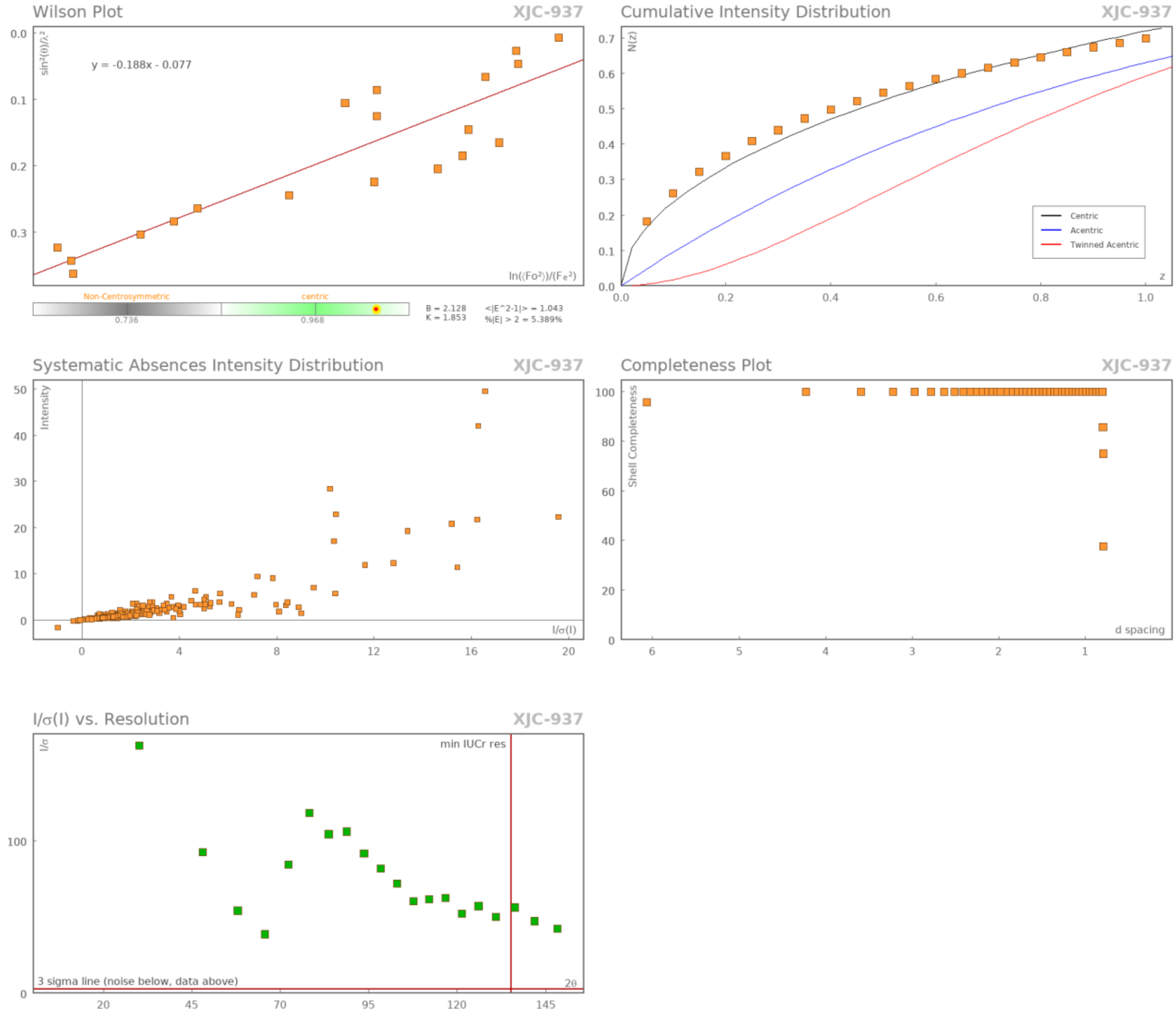

Supplementary Figure 8: Data Plots: Refinement and Data

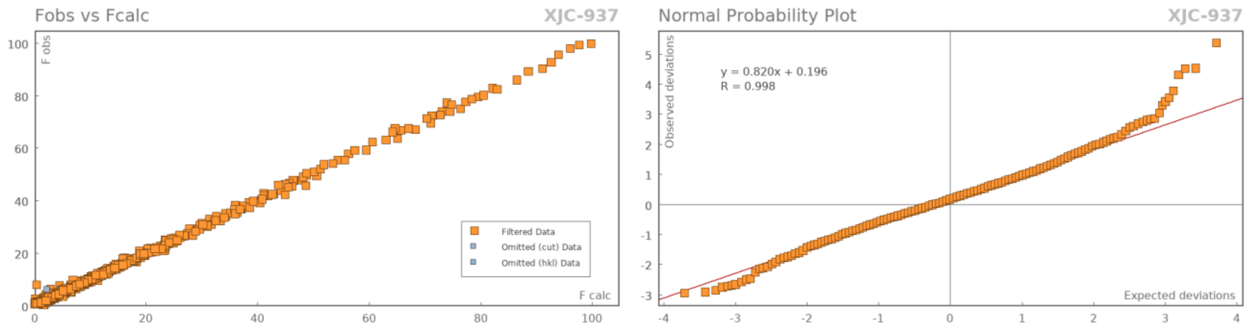

Supplementary Table 19: Reflection Statistics

Total reflections (after filtering)	25216	Unique reflections	4790
Completeness	0.995	Mean I/σ	74.62
hkl_{max} collected	(12, 15, 19)	hkl_{min} collected	(-14, -13, -19)
hkl_{max} used	(14, 15, 19)	hkl_{min} used	(-14, 0, 0)
Lim d_{max} collected	100.0	Lim d_{min} collected	0.77
d_{max} used	12.7	d_{min} used	0.79
Friedel pairs	2262	Friedel pairs merged	1
Inconsistent equivalents	20	R_{int}	0.0129
R_{sigma}	0.0083	Intensity transformed	0
Omitted reflections	0	Omitted by user (OMIT hkl)	1
Multiplicity	(5464, 3030, 1604, 718, 380, 214, 117, 47, 35, 36, 48, 20, 11, 2, 1)	Maximum multiplicity	18
Removed systematic absences	540	Filtered off (Shel/OMIT)	0

Supplementary Table 20: Fractional Atomic Coordinates (×10⁴) and Equivalent Isotropic Displacement Parameters (Å²×10³) for **xjc-937**. U_{eq} is defined as 1/3 of the trace of the orthogonalised U_{ij}.

**Atom**	**x**	**y**	**z**	U_{eq}
O1	9176.6(9)	4829.2(9)	6387.8(8)	47.6(3)
O2	4361.0(9)	3091.3(7)	8710.5(6)	35.6(2)
O3	3387.7(9)	4984.0(8)	8855.1(6)	35.8(2)
O4	3436.1(9)	6388.2(7)	7593.5(6)	33.8(2)
O5	8091.7(8)	1876.4(7)	6357.0(5)	27.4(2)
O6	4700.1(8)	502.0(7)	3956.1(6)	32.9(2)
O7	3138.4(8)	2019.6(8)	4319.5(6)	34.2(2)
O8	3809.5(7)	3736.7(7)	5275.7(5)	25.75(19)
C1	6047.3(10)	4049.8(9)	5968.1(7)	19.9(2)
C2	7328.5(10)	4022.3(9)	6275.8(7)	22.3(2)
C3	7949.4(11)	5066.5(11)	6428.0(9)	30.5(3)
C4	9336.3(11)	3835.1(11)	5982.5(9)	33.7(3)
C5	8128.1(10)	3513.7(10)	5639.7(7)	24.3(2)
C6	7836.8(10)	2356.0(10)	5556.2(7)	23.2(2)
C7	6552.1(10)	2264.2(9)	5281.9(7)	20.9(2)
C8	6231.9(11)	1408.4(9)	4770.7(7)	23.1(2)
C9	5095.7(11)	1307.1(9)	4457.1(7)	24.4(2)
C10	4268.2(10)	2081.3(10)	4638.3(7)	24.1(2)
C11	4588.0(10)	2927.2(9)	5151.4(7)	21.6(2)
C12	5731.5(10)	3035.5(9)	5488.9(7)	19.9(2)
C13	5262.1(10)	4281.3(9)	6707.1(7)	20.3(2)
C14	5136.1(10)	3530.2(9)	7337.4(7)	22.6(2)
C15	4512.9(11)	3771.3(10)	8054.0(7)	25.1(3)
C16	4000.5(11)	4760.6(10)	8142.1(7)	25.5(3)

Atom	x	y	z	U_{eq}
C17	4063.2(10)	5484.9(9)	7482.7(8)	24.2(2)
C18	4718.8(10)	5254.7(9)	6777.5(7)	22.8(2)
C19	4714.1(16)	2027.1(12)	8588.7(11)	47.5(4)
C20	3840.4(16)	5839.2(13)	9351.3(9)	45.3(4)
C21	3501.6(15)	7174.3(12)	6962.9(10)	43.1(4)
C22	8021.3(12)	759.2(11)	6338.2(8)	31.9(3)
C23	5546.0(13)	-190.1(11)	3614.1(9)	36.6(3)
C24	3046.2(14)	2129.3(14)	3422.3(9)	44.9(4)
C25	2805.2(11)	3482.3(12)	5758.0(9)	36.1(3)

Supplementary Table 21: Anisotropic Displacement Parameters (×10[4]) for **xjc-937**. The anisotropic displacement factor exponent takes the form: $-2\pi^2[h^2a^{*2} \times U_{11} + ... + 2hka^* \times b^* \times U_{12}]$

Atom	U_{11}	U_{22}	U_{33}	U_{23}	U_{13}	U_{12}
O1	24.5(5)	43.0(6)	75.1(8)	-24.8(6)	2.3(5)	-8.5(4)
O2	50.1(6)	28.2(5)	29.3(5)	10.9(4)	18.4(4)	9.9(4)
O3	42.3(5)	33.4(5)	32.7(5)	4.7(4)	21.2(4)	6.7(4)
O4	40.5(5)	30.5(5)	31.0(5)	9.9(4)	12.6(4)	14.5(4)
O5	30.2(4)	30.6(5)	21.2(4)	-0.5(3)	-5.6(3)	2.3(4)
O6	35.8(5)	26.8(5)	35.5(5)	-8.1(4)	-6.0(4)	-6.2(4)
O7	24.4(4)	45.5(6)	32.2(5)	-5.2(4)	-7.2(4)	-6.7(4)
O8	21.0(4)	28.9(4)	27.2(4)	1.1(3)	-0.7(3)	1.2(3)
C1	21.2(5)	22.3(5)	16.2(5)	1.0(4)	1.4(4)	-1.7(4)
C2	21.7(5)	26.8(6)	18.3(5)	-3.3(4)	0.9(4)	-2.3(4)
C3	25.8(6)	32.1(7)	33.7(6)	-9.1(5)	1.4(5)	-5.4(5)
C4	22.7(6)	39.8(7)	38.6(7)	-11.8(6)	3.2(5)	-4.5(5)
C5	21.0(5)	30.7(6)	21.2(5)	-3.0(5)	3.1(4)	-2.7(5)
C6	22.2(5)	29.3(6)	18.0(5)	-1.8(4)	0.5(4)	0.7(4)
C7	23.9(5)	23.9(5)	14.8(5)	2.2(4)	-0.4(4)	-1.8(4)
C8	28.5(6)	22.2(5)	18.6(5)	1.8(4)	-1.0(4)	0.1(4)
C9	31.9(6)	22.7(6)	18.5(5)	1.0(4)	-2.0(4)	-6.4(5)
C10	22.6(6)	29.3(6)	20.2(5)	2.9(4)	-3.3(4)	-6.1(5)
C11	23.0(5)	23.8(5)	18.0(5)	4.5(4)	0.6(4)	-1.4(4)
C12	22.7(5)	23.1(5)	13.8(5)	2.6(4)	0.3(4)	-2.7(4)
C13	19.1(5)	24.7(6)	17.0(5)	0.0(4)	-0.5(4)	-2.3(4)
C14	23.5(5)	22.0(5)	22.4(5)	1.7(4)	2.5(4)	2.1(4)
C15	26.8(6)	26.2(6)	22.4(6)	6.1(5)	4.8(4)	1.0(5)
C16	25.1(6)	28.4(6)	23.4(6)	3.0(5)	8.7(5)	2.7(5)
C17	23.3(5)	24.2(6)	25.2(6)	3.2(5)	2.3(4)	4.3(4)
C18	24.3(5)	24.2(6)	19.8(5)	4.6(4)	0.9(4)	-0.1(4)
C19	64.7(10)	31.5(7)	47.8(9)	19.3(6)	31.7(8)	17.2(7)
C20	65.7(10)	41.0(8)	30.1(7)	-2.3(6)	16.6(7)	11.3(7)
C21	56.3(9)	33.9(7)	39.9(8)	14.7(6)	14.8(7)	20.1(7)
C22	32.8(7)	32.0(7)	30.6(6)	3.6(5)	-5.9(5)	1.5(5)
C23	45.9(8)	29.4(7)	34.6(7)	-10.5(5)	0.1(6)	-5.1(6)
C24	44.0(8)	56.6(9)	32.9(7)	-12.7(7)	-19.7(6)	12.0(7)
C25	23.1(6)	47.1(8)	38.3(7)	-2.8(6)	5.3(5)	-1.3(6)

Supplementary Table 22: Bond Lengths in Å for **xjc-937**.

Atom	Atom	Length/Å
O1	C3	1.4384(16)
O1	C4	1.4318(17)
O2	C15	1.3699(14)
O2	C19	1.4257(16)
O3	C16	1.3787(14)

Atom	Atom	Length/Å
O3	C20	1.431(2)
O4	C17	1.3670(14)
O4	C21	1.4188(16)
O5	C6	1.4349(14)
O5	C22	1.4218(16)

Atom	Atom	Length/Å
O6	C9	1.3662(14)
O6	C23	1.4257(17)
O7	C10	1.3774(14)
O7	C24	1.4350(17)
O8	C11	1.3778(14)
O8	C25	1.4350(15)
C1	C2	1.5325(15)
C1	C12	1.5351(15)
C1	C13	1.5269(15)
C2	C3	1.5209(16)
C2	C5	1.5255(15)
C4	C5	1.5271(16)
C5	C6	1.5129(17)

Atom	Atom	Length/Å
C6	C7	1.5256(15)
C7	C8	1.4004(16)
C7	C12	1.4020(16)
C8	C9	1.3851(17)
C9	C10	1.4001(18)
C10	C11	1.3923(17)
C11	C12	1.4068(16)
C13	C14	1.3942(16)
C13	C18	1.3897(16)
C14	C15	1.3945(16)
C15	C16	1.3952(17)
C16	C17	1.3985(17)
C17	C18	1.3969(16)

Supplementary Table 23: Bond Angles in ° for **xjc-937**.

Atom	Atom	Atom	Angle/°
C4	O1	C3	109.95(10)
C15	O2	C19	116.88(10)
C16	O3	C20	115.19(11)
C17	O4	C21	117.51(10)
C22	O5	C6	113.27(9)
C9	O6	C23	117.83(10)
C10	O7	C24	113.82(11)
C11	O8	C25	115.98(10)
C2	C1	C12	110.46(9)
C13	C1	C2	109.60(9)
C13	C1	C12	114.12(9)
C3	C2	C1	117.98(10)
C3	C2	C5	100.92(9)
C5	C2	C1	112.50(9)
O1	C3	C2	105.18(10)
O1	C4	C5	105.76(10)
C2	C5	C4	101.68(9)
C6	C5	C2	109.60(10)
C6	C5	C4	119.09(11)
O5	C6	C5	107.21(9)
O5	C6	C7	113.11(9)
C5	C6	C7	107.91(10)
C8	C7	C6	117.40(10)
C8	C7	C12	120.85(10)
C12	C7	C6	121.65(10)
C9	C8	C7	120.62(11)

Atom	Atom	Atom	Angle/°
O6	C9	C8	124.92(11)
O6	C9	C10	115.53(11)
C8	C9	C10	119.55(11)
O7	C10	C9	121.10(11)
O7	C10	C11	119.26(11)
C11	C10	C9	119.64(11)
O8	C11	C10	119.98(10)
O8	C11	C12	118.01(10)
C10	C11	C12	121.73(11)
C7	C12	C1	123.59(10)
C7	C12	C11	117.58(10)
C11	C12	C1	118.38(10)
C14	C13	C1	119.73(10)
C18	C13	C1	120.45(10)
C18	C13	C14	119.74(10)
C13	C14	C15	120.15(11)
O2	C15	C14	124.24(11)
O2	C15	C16	115.42(10)
C14	C15	C16	120.34(11)
O3	C16	C15	119.48(11)
O3	C16	C17	121.26(11)
C15	C16	C17	119.16(11)
O4	C17	C16	114.71(10)
O4	C17	C18	125.03(11)
C18	C17	C16	120.26(11)
C13	C18	C17	120.11(10)

Supplementary Table 24: Torsion Angles in ° for **xjc-937**.

Atom	Atom	Atom	Atom	Angle/°
O1	C4	C5	C2	-30.37(13)
O1	C4	C5	C6	-<br>150.84(11)
O2	C15	C16	O3	-0.44(18)
O2	C15	C16	C17	-<br>176.84(11)
O3	C16	C17	O4	-2.65(18)
O3	C16	C17	C18	178.03(11)
O4	C17	C18	C13	-

Atom	Atom	Atom	Atom	Angle/°
				175.84(11)
O5	C6	C7	C8	-93.44(12)
O5	C6	C7	C12	90.26(13)
O6	C9	C10	O7	-0.43(16)
O6	C9	C10	C11	179.36(10)
O7	C10	C11	O8	-5.64(16)
O7	C10	C11	C12	-179.47(10)
O8	C11	C12	C1	-0.50(15)
O8	C11	C12	C7	-173.14(10)
C1	C2	C3	O1	-158.53(10)
C1	C2	C5	C4	166.12(10)
C1	C2	C5	C6	-66.96(12)
C1	C13	C14	C15	173.84(11)
C1	C13	C18	C17	-175.86(10)
C2	C1	C12	C7	-7.17(14)
C2	C1	C12	C11	-179.34(9)
C2	C1	C13	C14	-68.84(13)
C2	C1	C13	C18	107.94(12)
C2	C5	C6	O5	-63.81(12)
C2	C5	C6	C7	58.32(12)
C3	O1	C4	C5	8.38(16)
C3	C2	C5	C4	39.47(12)
C3	C2	C5	C6	166.38(10)
C4	O1	C3	C2	17.39(15)
C4	C5	C6	O5	52.56(14)
C4	C5	C6	C7	174.69(10)
C5	C2	C3	O1	-35.58(13)
C5	C6	C7	C8	148.14(10)
C5	C6	C7	C12	-28.16(14)
C6	C7	C8	C9	-176.22(10)
C6	C7	C12	C1	2.73(16)
C6	C7	C12	C11	174.95(10)
C7	C8	C9	O6	-179.89(11)
C7	C8	C9	C10	1.46(17)
C8	C7	C12	C1	-173.45(10)
C8	C7	C12	C11	-1.23(16)
C8	C9	C10	O7	178.34(11)
C8	C9	C10	C11	-1.88(17)
C9	C10	C11	O8	174.57(10)
C9	C10	C11	C12	0.74(17)
C10	C11	C12	C1	173.44(10)
C10	C11	C12	C7	0.80(16)
C12	C1	C2	C3	154.96(10)
C12	C1	C2	C5	38.07(13)
C12	C1	C13	C14	55.62(14)
C12	C1	C13	C18	-127.59(11)
C12	C7	C8	C9	0.11(17)
C13	C1	C2	C3	-78.47(12)
C13	C1	C2	C5	164.64(10)
C13	C1	C12	C7	-131.18(11)
C13	C1	C12	C11	56.66(13)
C13	C14	C15	O2	-

Atom	Atom	Atom	Atom	Angle/°
				178.84(11)
C13	C14	C15	C16	0.69(18)
C14	C13	C18	C17	0.93(17)
C14	C15	C16	O3	179.99(11)
C14	C15	C16	C17	3.59(19)
C15	C16	C17	O4	173.68(11)
C15	C16	C17	C18	-5.64(19)
C16	C17	C18	C13	3.40(18)
C18	C13	C14	C15	-2.97(17)
C19	O2	C15	C14	-9.8(2)
C19	O2	C15	C16	170.67(13)
C20	O3	C16	C15	119.08(14)
C20	O3	C16	C17	-64.60(16)
C21	O4	C17	C16	177.47(13)
C21	O4	C17	C18	-3.25(19)
C22	O5	C6	C5	-
				171.32(10)
C22	O5	C6	C7	69.86(13)
C23	O6	C9	C8	-10.99(18)
C23	O6	C9	C10	167.70(11)
C24	O7	C10	C9	-66.44(15)
C24	O7	C10	C11	113.77(13)
C25	O8	C11	C10	68.87(14)
C25	O8	C11	C12	-
				117.08(12)

Supplementary Table 25: Hydrogen Fractional Atomic Coordinates (×10[4]) and Equivalent Isotropic Displacement Parameters (Å[2]×10[3]) for **xjc-937**. U_{eq} is defined as 1/3 of the trace of the orthogonalised U_{ij}.

Atom	x	y	z	U_{eq}
H1	5961.04	4648.21	5562.78	24
H2	7378.06	3603.04	6807.66	27
H3A	7715.82	5585.95	5990.88	37
H3B	7760.85	5354.94	6986.41	37
H4A	9641.65	3305.97	6389.22	40
H4B	9893.57	3901.82	5519.88	40
H5	7989.71	3859.55	5081.13	29
H6	8336.76	2030.65	5120.15	28
H8	6799.27	892.55	4637.67	28
H14	5475.66	2852.53	7278.47	27
H18	4793.37	5764.38	6345.22	27
H19A	4332	1748.92	8076.54	71
H19B	4488.12	1602.69	9072.49	71
H19C	5565.04	1998.13	8533.33	71
H20A	3974.21	6447.87	8987.71	68
H20B	4580.54	5627.64	9626.53	68
H20C	3276.23	6027.66	9779.21	68
H21A	3247.65	6877.87	6419.26	65
H21B	4310.32	7422.96	6929.22	65
H21C	2992.19	7765.08	7104.58	65
H22A	8440.56	492.26	5852.93	48
H22B	7198.98	544.23	6292.02	48
H22C	8375.87	470.92	6856.53	48
H23A	5151.94	-701.25	3242.12	55
H23B	5959.59	-563.08	4072.07	55
H23C	6107.81	218.33	3293.02	55
H24A	2225.79	2056.12	3237.42	67
H24B	3514.66	1582.22	3156.76	67

Atom	x	y	z	U_{eq}
H24C	3335.28	2824.47	3259.9	67
H25A	2240.54	3089.64	5405.58	54
H25B	2441.69	4132.69	5954.81	54
H25C	3047.34	3050.86	6242.8	54

Citations

CrysAlisPro (ROD), Rigaku Oxford Diffraction, Poland (?).

CrysAlisPro Software System, Rigaku Oxford Diffraction, (2020).

O.V. Dolomanov and L.J. Bourhis and R.J. Gildea and J.A.K. Howard and H. Puschmann, Olex2: A complete structure solution, refinement and analysis program, *J. Appl. Cryst.*, (2009), **42**, 339-341.

Sheldrick, G.M., Crystal structure refinement with ShelXL, *Acta Cryst.*, (2015), **C71**, 3-8.

Sheldrick, G.M., ShelXT-Integrated space-group and crystal-structure determination, *Acta Cryst.*, (2015), **A71**, 3-8.

20e-A

Submitted by: **Jia-Chen Xiang**

Solved by: **Farzaneh Fadaei Tirani**

Sample ID: **XJC-964a**

R_1=4.46%

Supplementary Figure 9: X-ray of compound **20e-A**

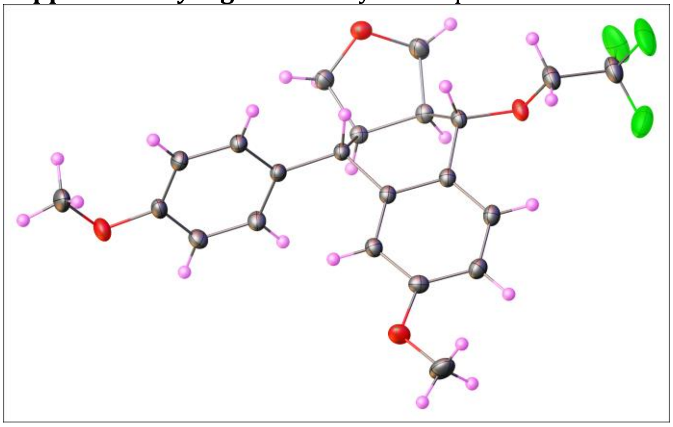

Supplementary Table 26: Crystal data for compound **20e-A**

Formula	$C_{22}H_{23}F_3O_4$
$D_{calc.}$/ g cm^{-3}	1.435
μ/mm^{-1}	0.117
Formula Weight	408.40
Colour	colourless
Shape	prism
Size/mm^3	0.64×0.38×0.16
T/K	140.00(10)
Crystal System	monoclinic
Space Group	$P2_1/n$
a/Å	10.2952(4)
b/Å	8.6405(3)
c/Å	21.7787(9)
α/°	90
β/°	102.717(4)
γ/°	90
V/Å^3	1889.82(13)
Z	4
Z'	1
Wavelength/Å	0.71073
Radiation type	MoK_α
Θ_{min}/°	3.039
Θ_{max}/°	32.795
Measured Refl's.	22768
Indep't Refl's	6525
Refl's I≥2 σ(I)	5347
R_{int}	0.0218
Parameters	265
Restraints	0
Largest Peak/e Å^{-3}	0.445
Deepest Hole/e Å^{-3}	-0.290
GooF	1.055
wR_2 (all data)	0.1241
wR_2	0.1148
R_1 (all data)	0.0568
R_1	0.0446

Experimental. Single colourless prism crystals of **xjc-964a** were used as supplied. A suitable crystal with dimensions 0.64 × 0.38 × 0.16 mm^3 was selected and mounted on a SuperNova, Dual, Cu at home/near, Atlas diffractometer. The crystal was kept at a steady T = 140.00(10) K during data collection. The structure was solved with the ShelXT 2018/2 (Sheldrick, 2015) solution program using dual methods and by using Olex2 (Dolomanov et al., 2009) as the graphical interface. The model was refined with ShelXL 2018/3 (Sheldrick, 2015) using full matrix least squares minimisation on $\boldsymbol{F^2}$.

Crystal Data. $C_{22}H_{23}F_3O_4$, M_r = 408.40, monoclinic, $P2_1/n$ (No. 14), a = 10.2952(4) Å, b = 8.6405(3) Å, c = 21.7787(9) Å, β = 102.717(4)°, α = γ = 90°, V = 1889.82(13) Å^3, T = 140.00(10) K, Z = 4, Z' = 1, μ(Mo K_α) = 0.117, 22768 reflections measured, 6525 unique (R_{int} = 0.0218) which were used in all calculations. The final wR_2 was 0.1241 (all data) and R_1 was 0.0446 (I≥2 σ(I)).

Supplementary Table 27: Structure Quality Indicators

**Reflections:**	d min (Mo) CIF 0.66	I/σ(I) CIF 42.7	Rint CIF 2.18%	complete 100% (IUCr) 100%
**Refinement:**	Shift CIF 0.001	Max Peak CIF 0.4	Min Peak CIF -0.3	GooF CIF 1.055

A colourless prism-shaped crystal with dimensions 0.64 × 0.38 × 0.16 mm^3 was mounted. Data were collected using a SuperNova, Dual, Cu at home/near, Atlas diffractometer operating at T = 140.00(10) K.

Data were measured using ω scans using Mo K_α radiation. The diffraction pattern was indexed and the total number of runs and images was based on the strategy calculation from the program CrysAlisPro (Rigaku, V1.171.41.78a, 2020). The maximum resolution achieved was Θ = 32.795° (0.66 Å).

The diffraction pattern was indexed and the total number of runs and images was based on the strategy calculation from the program CrysAlisPro (Rigaku, V1.171.41.78a, 2020). The unit cell was refined using CrysAlisPro (Rigaku, V1.171.41.78a, 2020) on 9674 reflections, 42% of the observed reflections.

Data reduction, scaling and absorption corrections were performed using CrysAlisPro (Rigaku, V1.171.41.78a, 2020). The final completeness is 99.90 % out to 32.795° in Θ. A Gaussian absorption correction was performed using CrysAlisPro 1.171.41.78a (Rigaku Oxford Diffraction, 2020) Numerical absorption correction based on Gaussian integration over a multifaceted crystal model. Empirical absorption correction using spherical harmonics as implemented in SCALE3 ABSPACK scaling algorithm. The absorption coefficient μ of this material is 0.117 mm^{-1} at this wavelength (λ = 0.71073Å) and the minimum and maximum transmissions are 0.489 and 1.000.

The structure was solved and the space group $P2_1/n$ (# 14) determined by the ShelXT 2018/2 (Sheldrick, 2015) structure solution program using using dual methods and refined by full matrix least squares minimisation on $\boldsymbol{F}^2$ using version 2018/3 of ShelXL 2018/3 (Sheldrick, 2015). All non-hydrogen atoms were refined anisotropically. Hydrogen atom positions were calculated geometrically and refined using the riding model.

There is a single molecule in the asymmetric unit, which is represented by the reported sum formula. In other words: Z is 4 and Z' is 1.

Citations

CrysAlis^{Pro} Software System, Rigaku Oxford Diffraction, (2020).

Sheldrick, G.M., ShelXT-Integrated space-group and crystal-structure determination, *Acta Cryst.*, (2015), **A71**, 3-8.

Sheldrick, G.M., Crystal structure refinement with ShelXL, *Acta Cryst.*, (2015), **C71**, 3-8.

O.V. Dolomanov and L.J. Bourhis and R.J. Gildea and J.A.K. Howard and H. Puschmann, Olex2: A complete structure solution, refinement and analysis program, *J. Appl. Cryst.*, (2009), **42**, 339-341.

Supplementary Figure 10: Images of the Crystal on the Diffractometer

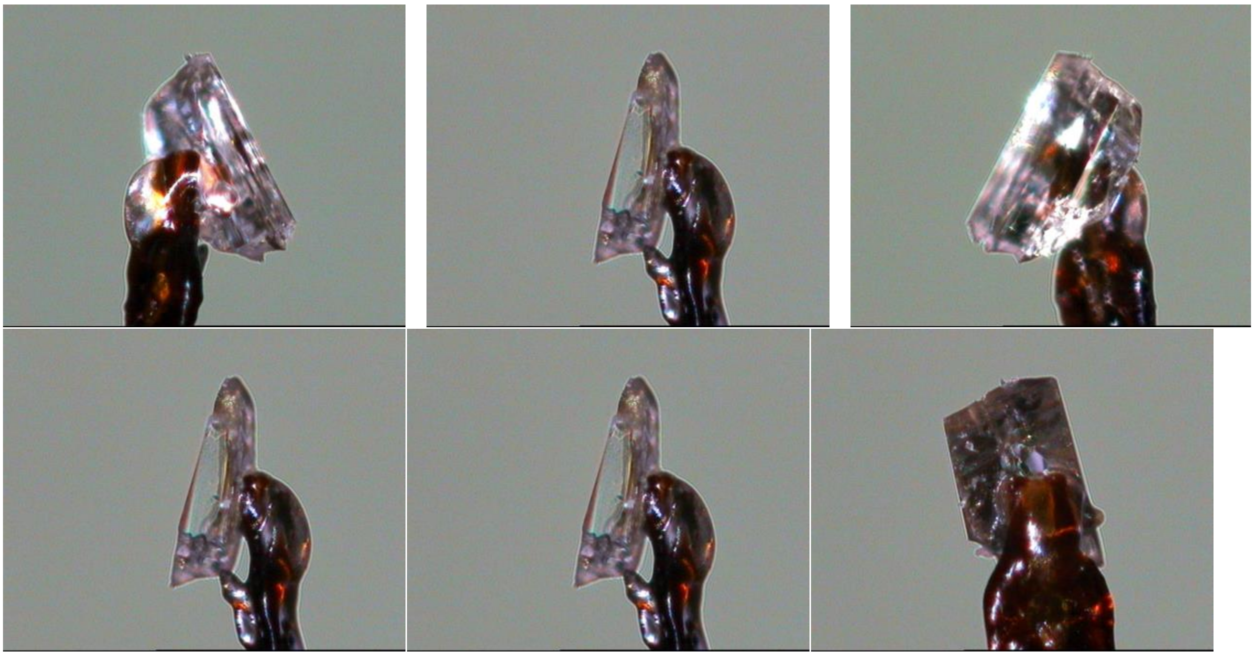

Supplementary Figure 11: Data Plots: Diffraction Data

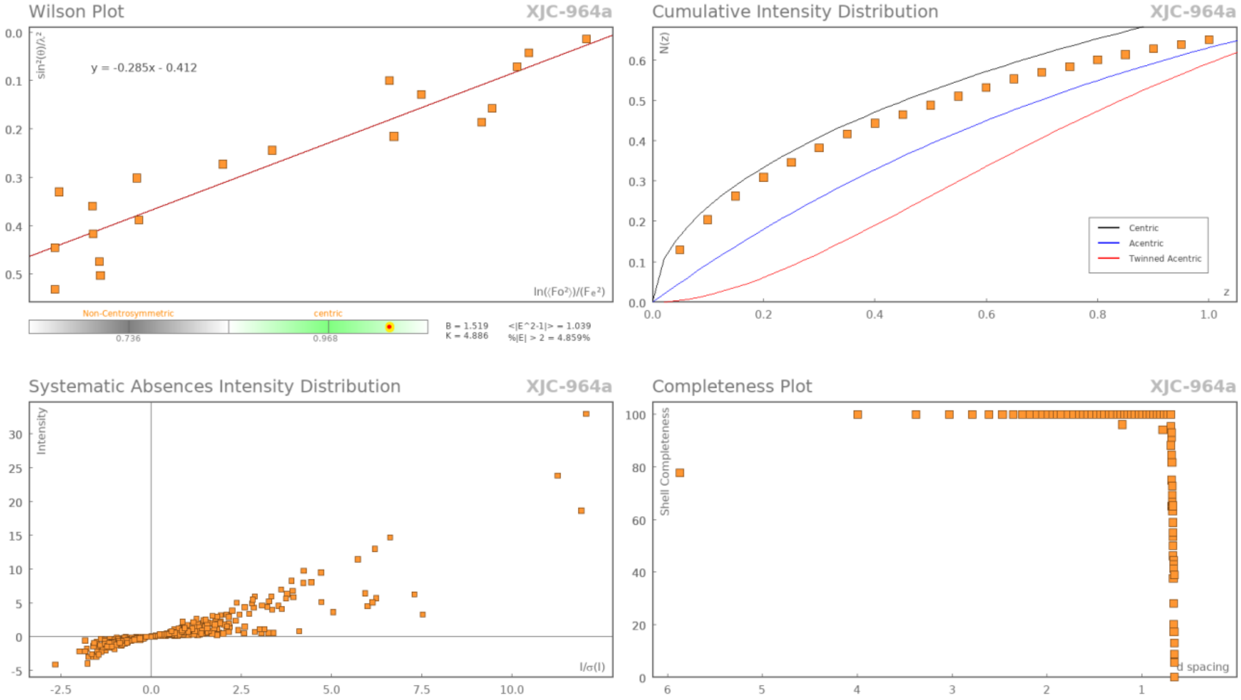

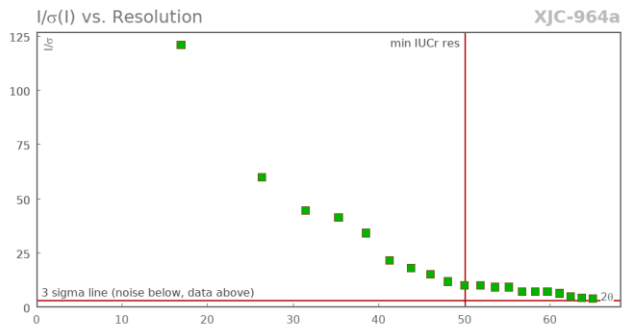

Supplementary Figure 12: Data Plots: Refinement and Data

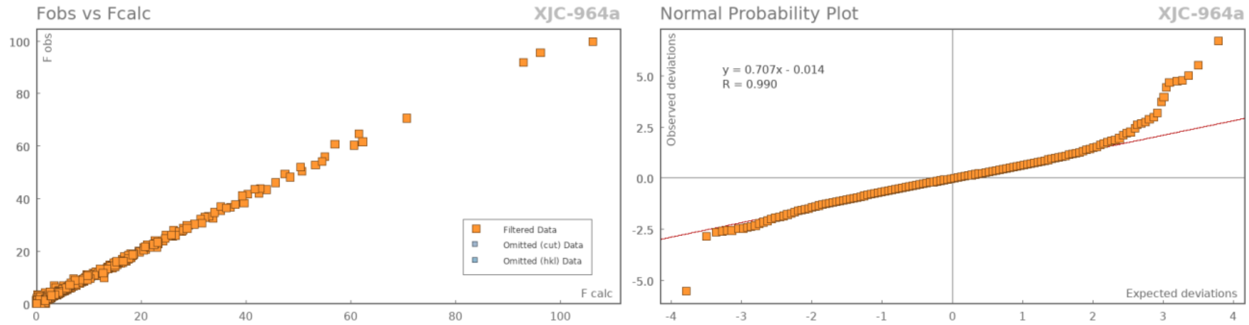

Supplementary Table 28: Reflection Statistics

Total reflections (after filtering)	23514	Unique reflections	6525
Completeness	0.93	Mean I/σ	23.67
hkl_{max} collected	(15, 9, 32)	hkl_{min} collected	(-15, -12, -32)
hkl_{max} used	(15, 12, 32)	hkl_{min} used	(-15, 0, 0)
Lim d_{max} collected	100.0	Lim d_{min} collected	0.36
d_{max} used	6.7	d_{min} used	0.66
Friedel pairs	3893	Friedel pairs merged	1
Inconsistent equivalents	0	R_{int}	0.0218
R_{sigma}	0.0234	Intensity transformed	0
Omitted reflections	0	Omitted by user (OMIT hkl)	0
Multiplicity	(10508, 4979, 810, 128, 20, 1)	Maximum multiplicity	11
Removed systematic absences	746	Filtered off (Shel/OMIT)	0

Supplementary Table 29: Fractional Atomic Coordinates (×10⁴) and Equivalent Isotropic Displacement Parameters (Å²×10³) for **xjc-964a.** U_{eq} is defined as 1/3 of the trace of the orthogonalised U_{ij}.

Atom	x	y	z	U_{eq}
F1	2873.8(12)	1891.3(13)	2344.0(4)	55.0(3)
F2	3431.3(13)	4211.7(12)	2165.3(4)	55.6(3)
F3	4919.8(11)	2540.4(12)	2552.0(5)	49.7(3)
O1	909.9(8)	1603.5(10)	4613.6(4)	25.46(18)
O2	3905.9(9)	2366.3(11)	8114.3(4)	27.98(19)
O3	3821.0(8)	2207.7(9)	3598.4(4)	20.75(16)
O4	8434.9(8)	3845.3(11)	6003.6(4)	27.63(19)
C1	3707.2(10)	2536.6(11)	5491.3(5)	15.91(18)
C2	3090.1(10)	1043.5(11)	5158.4(5)	17.39(18)
C3	1620.6(11)	889.5(14)	5177.8(5)	22.5(2)
C4	1546.4(12)	992.1(15)	4142.8(5)	25.9(2)
C5	3039.9(11)	1051.4(12)	4440.8(5)	18.54(19)
C6	3696.0(11)	2482.3(12)	4229.9(5)	17.09(18)
C7	5012.0(10)	2813.7(11)	4667.8(5)	16.64(18)

Atom	x	y	z	U_{eq}
C8	6186.3(11)	3065.3(12)	4471.9(5)	19.7(2)
C9	7366.9(11)	3403.2(13)	4902.1(5)	21.0(2)
C10	7359.3(11)	3479.9(12)	5536.8(5)	19.7(2)
C11	6191.2(11)	3177.7(12)	5741.1(5)	18.31(19)
C12	5021.4(10)	2852.9(11)	5312.9(5)	15.98(18)
C13	3769.6(10)	2502.6(12)	6189.8(5)	16.70(18)
C14	4449.7(11)	1346.6(13)	6581.5(5)	21.2(2)
C15	4482.3(11)	1342.0(13)	7219.3(5)	22.5(2)
C16	3824.4(11)	2490.4(13)	7482.3(5)	20.5(2)
C17	3145.7(11)	3648.8(13)	7104.4(5)	21.0(2)
C18	3131.4(11)	3638.9(12)	6463.5(5)	18.60(19)
C19	3270.4(13)	3546.6(18)	8397.3(6)	31.6(3)
C20	3597.4(13)	3529.7(13)	3212.5(5)	25.3(2)
C21	3700.9(15)	3034.6(15)	2566.3(6)	30.6(3)
C22	9596.7(12)	4387.9(18)	5820.5(7)	33.3(3)

Supplementary Table 30: Anisotropic Displacement Parameters (×10[4]) for **xjc-964a.** The anisotropic displacement factor exponent takes the form: $-2\pi^2[h^2a^{*2} \times U_{11} + ... +2hka^* \times b^* \times U_{12}]$

Atom	U_{11}	U_{22}	U_{33}	U_{23}	U_{13}	U_{12}
F1	79.0(8)	57.6(6)	23.2(4)	-11.6(4)	0.2(4)	-18.9(5)
F2	99.6(9)	45.7(5)	26.0(4)	17.1(4)	23.5(5)	24.3(5)
F3	59.1(6)	55.2(6)	44.1(5)	8.3(4)	31.1(5)	17.9(5)
O1	19.4(4)	31.5(4)	24.9(4)	2.9(3)	3.4(3)	1.2(3)
O2	33.6(5)	35.7(5)	14.9(4)	1.1(3)	5.8(3)	4.1(4)
O3	29.8(4)	19.1(3)	13.6(3)	-0.2(3)	5.5(3)	2.7(3)
O4	19.1(4)	37.7(5)	24.5(4)	0.4(3)	1.2(3)	-5.7(3)
C1	17.4(4)	15.4(4)	15.5(4)	0.8(3)	4.8(3)	1.3(3)
C2	18.9(4)	15.2(4)	18.2(4)	1.3(3)	4.3(4)	-0.3(3)
C3	20.6(5)	25.4(5)	22.2(5)	1.8(4)	5.9(4)	-3.5(4)
C4	23.3(5)	33.4(6)	19.7(5)	-1.7(4)	2.0(4)	-5.6(4)
C5	21.2(5)	16.1(4)	18.4(4)	-2.0(3)	4.7(4)	-1.0(3)
C6	21.2(5)	16.5(4)	13.7(4)	-0.7(3)	4.2(3)	1.0(3)
C7	19.8(4)	14.0(4)	16.8(4)	0.5(3)	5.4(4)	0.6(3)
C8	23.7(5)	17.9(4)	19.1(4)	1.0(3)	8.6(4)	-0.5(4)
C9	20.8(5)	20.0(5)	23.9(5)	1.3(4)	8.9(4)	-0.8(4)
C10	18.4(5)	17.3(4)	22.7(5)	1.2(4)	3.0(4)	-0.8(3)
C11	20.8(5)	17.2(4)	17.1(4)	0.5(3)	4.5(4)	0.1(4)
C12	18.7(4)	13.2(4)	16.8(4)	0.5(3)	5.4(3)	0.3(3)
C13	16.9(4)	17.6(4)	16.3(4)	0.6(3)	5.1(3)	-0.7(3)
C14	23.9(5)	20.8(5)	19.6(5)	2.1(4)	6.6(4)	4.5(4)
C15	24.4(5)	23.6(5)	18.9(5)	3.8(4)	3.5(4)	3.1(4)
C16	20.5(5)	26.8(5)	14.4(4)	0.0(4)	4.1(4)	-2.3(4)
C17	21.8(5)	23.5(5)	18.7(5)	-1.4(4)	6.6(4)	2.5(4)
C18	19.4(5)	19.3(4)	17.5(4)	1.1(3)	4.9(4)	2.1(4)
C19	31.1(6)	47.6(8)	17.2(5)	-3.9(5)	7.8(5)	3.6(5)
C20	37.2(6)	22.2(5)	17.0(5)	1.9(4)	6.9(4)	4.7(4)
C21	45.7(7)	29.6(6)	17.3(5)	3.4(4)	8.6(5)	5.7(5)
C22	17.4(5)	43.6(7)	38.8(7)	-6.5(6)	5.9(5)	-5.0(5)

Supplementary Table 31: Bond Lengths in Å for **xjc-964a**.

Atom	Atom	Length/Å
F1	C21	1.3244(18)
F2	C21	1.3294(15)
F3	C21	1.3324(17)

Atom	Atom	Length/Å
O1	C3	1.4247(14)
O1	C4	1.4338(14)
O2	C16	1.3646(13)

Atom	Atom	Length/Å
O2	C19	1.4233(16)
O3	C6	1.4293(12)
O3	C20	1.4067(13)
O4	C10	1.3652(14)
O4	C22	1.4211(15)
C1	C2	1.5462(14)
C1	C12	1.5125(14)
C1	C13	1.5088(14)
C2	C3	1.5285(15)
C2	C5	1.5525(14)
C4	C5	1.5325(16)
C5	C6	1.5271(14)
C6	C7	1.5028(15)

Atom	Atom	Length/Å
C7	C8	1.3850(14)
C7	C12	1.4031(13)
C8	C9	1.3926(16)
C9	C10	1.3855(15)
C10	C11	1.3957(14)
C11	C12	1.3800(15)
C13	C14	1.3974(15)
C13	C18	1.3868(14)
C14	C15	1.3820(15)
C15	C16	1.3936(16)
C16	C17	1.3835(16)
C17	C18	1.3928(14)
C20	C21	1.4970(16)

Supplementary Table 32: Bond Angles in ° for **xjc-964a**.

Atom	Atom	Atom	Angle/°
C3	O1	C4	103.20(9)
C16	O2	C19	116.64(10)
C20	O3	C6	113.73(8)
C10	O4	C22	117.53(10)
C12	C1	C2	109.20(8)
C13	C1	C2	112.23(8)
C13	C1	C12	114.87(9)
C1	C2	C5	112.80(8)
C3	C2	C1	111.52(8)
C3	C2	C5	102.38(8)
O1	C3	C2	105.19(8)
O1	C4	C5	105.00(9)
C4	C5	C2	103.54(8)
C6	C5	C2	112.79(8)
C6	C5	C4	111.63(9)
O3	C6	C5	107.12(8)
O3	C6	C7	112.50(8)
C7	C6	C5	111.10(8)
C8	C7	C6	124.10(9)
C8	C7	C12	119.38(10)
C12	C7	C6	116.52(9)
C7	C8	C9	121.16(10)
C10	C9	C8	119.04(10)
O4	C10	C9	124.90(10)

Atom	Atom	Atom	Angle/°
O4	C10	C11	114.87(10)
C9	C10	C11	120.24(10)
C12	C11	C10	120.51(10)
C7	C12	C1	116.32(9)
C11	C12	C1	124.06(9)
C11	C12	C7	119.62(9)
C14	C13	C1	122.27(9)
C18	C13	C1	120.04(9)
C18	C13	C14	117.70(9)
C15	C14	C13	120.91(10)
C14	C15	C16	120.30(10)
O2	C16	C15	115.53(10)
O2	C16	C17	124.59(10)
C17	C16	C15	119.88(10)
C16	C17	C18	118.96(10)
C13	C18	C17	122.25(10)
O3	C20	C21	107.06(9)
F1	C21	F2	107.82(12)
F1	C21	F3	106.59(12)
F1	C21	C20	112.57(11)
F2	C21	F3	106.93(11)
F2	C21	C20	110.56(11)
F3	C21	C20	112.08(11)

Supplementary Table 33: Torsion Angles in ° for **xjc-964a**.

Atom	Atom	Atom	Atom	Angle/°
O1	C4	C5	C2	23.71(11)
O1	C4	C5	C6	-97.89(10)
O2	C16	C17	C18	-179.58(10)
O3	C6	C7	C8	9.43(14)
O3	C6	C7	C12	-170.13(8)
O3	C20	C21	F1	56.10(15)
O3	C20	C21	F2	176.75(12)
O3	C20	C21	F3	-64.05(14)
O4	C10	C11	C12	177.50(9)
C1	C2	C3	O1	91.72(10)
C1	C2	C5	C4	-116.95(9)

Atom	Atom	Atom	Atom	Angle/°
C1	C2	C5	C6	3.88(12)
C1	C13	C14	C15	-179.62(10)
C1	C13	C18	C17	179.22(10)
C2	C1	C12	C7	49.75(11)
C2	C1	C12	C11	-130.66(10)
C2	C1	C13	C14	58.13(13)
C2	C1	C13	C18	-121.45(10)
C2	C5	C6	O3	168.72(8)
C2	C5	C6	C7	45.48(11)
C3	O1	C4	C5	-43.09(11)
C3	C2	C5	C4	3.03(10)
C3	C2	C5	C6	123.86(9)
C4	O1	C3	C2	45.51(11)
C4	C5	C6	O3	-75.19(10)
C4	C5	C6	C7	161.57(9)
C5	C2	C3	O1	-29.15(10)
C5	C6	C7	C8	129.53(10)
C5	C6	C7	C12	-50.03(12)
C6	O3	C20	C21	-177.38(10)
C6	C7	C8	C9	178.46(10)
C6	C7	C12	C1	0.74(13)
C6	C7	C12	C11	-178.87(9)
C7	C8	C9	C10	0.29(16)
C8	C7	C12	C1	-178.84(9)
C8	C7	C12	C11	1.55(14)
C8	C9	C10	O4	-177.91(10)
C8	C9	C10	C11	1.84(16)
C9	C10	C11	C12	-2.27(16)
C10	C11	C12	C1	-179.02(9)
C10	C11	C12	C7	0.56(15)
C12	C1	C2	C3	-165.09(8)
C12	C1	C2	C5	-50.52(11)
C12	C1	C13	C14	-67.40(13)
C12	C1	C13	C18	113.02(11)
C12	C7	C8	C9	-1.99(15)
C13	C1	C2	C3	66.34(11)
C13	C1	C2	C5	-179.10(8)
C13	C1	C12	C7	176.84(8)
C13	C1	C12	C11	-3.57(14)
C13	C14	C15	C16	0.52(17)
C14	C13	C18	C17	-0.38(16)
C14	C15	C16	O2	179.20(10)
C14	C15	C16	C17	-0.60(17)
C15	C16	C17	C18	0.20(17)
C16	C17	C18	C13	0.30(17)
C18	C13	C14	C15	-0.03(16)
C19	O2	C16	C15	178.48(11)
C19	O2	C16	C17	-1.73(17)
C20	O3	C6	C5	142.44(10)
C20	O3	C6	C7	-95.19(11)
C22	O4	C10	C9	8.90(17)
C22	O4	C10	C11	-170.86(10)

Supplementary Table 34: Hydrogen Fractional Atomic Coordinates (×10[4]) and Equivalent Isotropic Displacement Parameters ($Å^2×10^3$) for **xjc-964a.** U_{eq} is defined as 1/3 of the trace of the orthogonalised U_{ij}.

Atom	x	y	z	U_{eq}
H1	3096.94	3405.18	5314.27	19
H2	3595.6	118.59	5358.79	21
H3A	1367.08	-213.2	5191.2	27
H3B	1435.23	1421.08	5551.99	27
H4A	1334.72	1629.35	3755.8	31
H4B	1256.08	-85.25	4035.04	31
H5	3481.6	98.07	4324.54	22
H6	3095.31	3391.23	4231.37	21
H8	6186.33	3006.43	4036.32	24
H9	8166.09	3578.55	4762.49	25
H11	6201.43	3195.81	6178.27	22
H14	4895.71	552.97	6406.99	25
H15	4955.35	552.42	7479.66	27
H17	2696.51	4438.64	7279.41	25
H18	2668.59	4437.74	6204.89	22
H19A	2310.52	3527.74	8216.67	47
H19B	3632.63	4557.64	8317.92	47
H19C	3430.42	3367.3	8852.07	47
H20A	4270.55	4333.86	3374.81	30
H20B	2702.48	3961.06	3203.33	30
H22A	9378.4	5314.86	5558.84	50
H22B	9932.78	3580.88	5579.87	50
H22C	10280.19	4642.13	6196.55	50

4 (aglacin E)

Submitted by: **Jia-Chen Xiang**

Solved by: **Farzaneh Fadaei Tirani**

Sample ID: **XJC-606dt**

R_1=3.13%

Supplementary Figure 13: X-ray of compound **4 (aglacin E)**

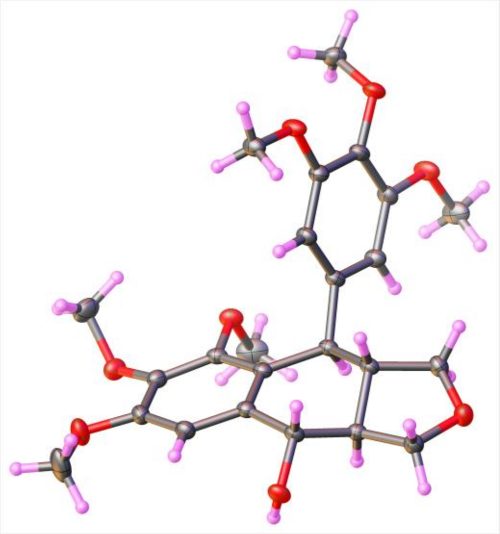

Experimental. Single clear colourless prism crystals of **xjc-606dt** were used as supplied. A suitable crystal with dimensions of 0.25 × 0.15 × 0.10 mm³ was selected and mounted on a SuperNova, Dual, Cu at home/near, AtlasS2 diffractometer. The crystal was kept at a steady T = 140.00(10) K during data collection. The structure was solved with the **ShelXT** 2018/2 (Sheldrick, 2018) solution program using dual methods and by using **Olex2** (Dolomanov et al., 2009) as the graphical interface. The model was refined with **ShelXL** 2018/3 (Sheldrick, 2015) using full matrix least squares minimisation on $|F|^2$.

Crystal Data. $C_{24}H_{30}O_8$, M_r = 446.48, triclinic, $P\bar{1}$ (No. 2), a = 8.7639(2) Å, b = 10.1274(3) Å, c = 13.6449(3) Å, α = 111.255(2)°, β = 90.0646(19)°, γ = 101.601(2)°, V = 1101.98(5) Å³, T = 140.00(10) K, Z = 2, Z' = 1, μ(Cu $K\alpha$) = 0.837, 20352 reflections measured, 4538 unique (R_{int} = 0.0192) which were used in all calculations. The final wR_2 was 0.0845 (all data) and R_1 was 0.0313 (I > 2(I)).

Supplementary Table 35: Crystal data for compound **4 (aglacin E)**

Formula	$C_{24}H_{30}O_8$
$D_{calc.}$/ g cm^{-3}	1.346
μ/mm^{-1}	0.837
Formula Weight	446.48
Colour	clear colourless
Shape	prism
Size/mm³	0.25×0.15×0.10
T/K	140.00(10)
Crystal System	triclinic
Space Group	$P\bar{1}$
a/Å	8.7639(2)
b/Å	10.1274(3)
c/Å	13.6449(3)
α/°	111.255(2)
β/°	90.0646(19)
γ/°	101.601(2)
V/Å³	1101.98(5)
Z	2
Z'	1
Wavelength/Å	1.54184
Radiation type	Cu $K\alpha$
Θ_{min}/°	3.487
Θ_{max}/°	76.187
Measured Refl's.	20352
Ind't Refl's	4538
Refl's with I > 2(I)	4258
R_{int}	0.0192
Parameters	410
Restraints	0
Largest Peak/e Å^{-3}	0.299
Deepest Hole/e Å^{-3}	-0.177
GooF	1.042
wR_2 (all data)	0.0845
wR_2	0.0830
R_1 (all data)	0.0331
R_1	0.0313

Supplementary Table 36: Structure Quality Indicators

**Reflections:**	d min (Cu) CIF 0.79	I/σ CIF 73.5	Rint CIF 1.92%	complete 100%
**Refinement:**	Shift CIF 0.000	Max Peak CIF 0.3	Min Peak CIF -0.2	GooF CIF 1.042

A clear colourless prism-shaped crystal with dimensions of 0.25 × 0.15 × 0.10 mm^3 was mounted. Data were collected using a SuperNova, Dual, Cu at home/near, AtlasS2 diffractometer operating at T = 140.00(10) K.

Data were measured using ω scans using Cu $K\alpha$ radiation. The diffraction pattern was indexed and the total number of runs and images was based on the strategy calculation from the program **CrysAlis^{Pro}** (Rigaku, V1.171.40.62a, 2019). The maximum resolution achieved was Θ = 76.187° (0.79 Å).

The diffraction pattern was indexed and the total number of runs and images was based on the strategy calculation from the program **CrysAlis^{Pro}** (Rigaku, V1.171.40.62a, 2019) The unit cell was refined using **CrysAlis^{Pro}** (Rigaku, V1.171.40.62a, 2019) on 13909 reflections, 68% of the observed reflections.

Data reduction, scaling and absorption corrections were performed using **CrysAlis^{Pro}** (Rigaku, V1.171.40.62a, 2019). The final completeness is 99.90 % out to 76.187° in Θ. A Gaussian absorption correction was performed using CrysAlis^{Pro} 1.171.40.62a (Rigaku Oxford Diffraction, 2019) Numerical absorption correction based on Gaussian integration over a multifaceted crystal model. Empirical absorption correction using spherical harmonics as implemented in SCALE3 ABSPACK scaling algorithm. The absorption coefficient μ of this material is 0.837 mm^{-1} at this wavelength (λ = 1.54184Å) and the minimum and maximum transmissions are 0.668 and 1.000.

The structure was solved and the space group $P\bar{1}$ (# 2) determined by the ShelXT 2018/2 (Sheldrick, 2018) structure solution program using using dual methods and refined by full matrix least squares minimisation on $|F|^2$ using version 2018/3 of ShelXL 2018/3 (Sheldrick, 2015). All non-hydrogen atoms were refined anisotropically. Hydrogen atom positions were found in a difference map and refined freely.

There is a single molecule in the asymmetric unit, which is represented by the reported sum formula. In other words: Z is 2 and Z' is 1.

Citations

CrysAlis^{Pro} Software System, Rigaku Oxford Diffraction, (2019).

Sheldrick, G.M., ShelXT-Integrated space-group and crystal-structure determination, *Acta Cryst.*, (2015), **A71**, 3-8.

Sheldrick, G.M., Crystal structure refinement with ShelXL, *Acta Cryst.*, (2015), **C71**, 3-8.

O.V. Dolomanov and L.J. Bourhis and R.J. Gildea and J.A.K. Howard and H. Puschmann, Olex2: A complete structure solution, refinement and analysis program, *J. Appl. Cryst.*, (2009), **42**, 339-341.

Supplementary Figure 14: Images of the crystal on the diffractometer

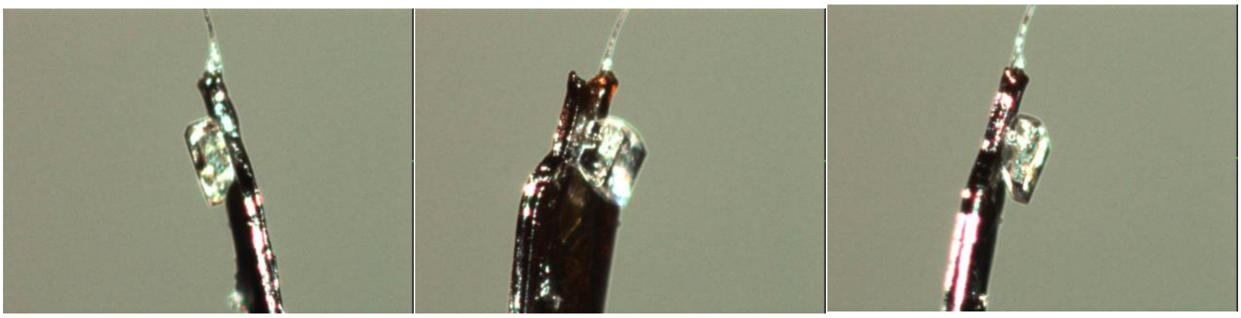

Supplementary Figure 15: Data Plots: Diffraction Data

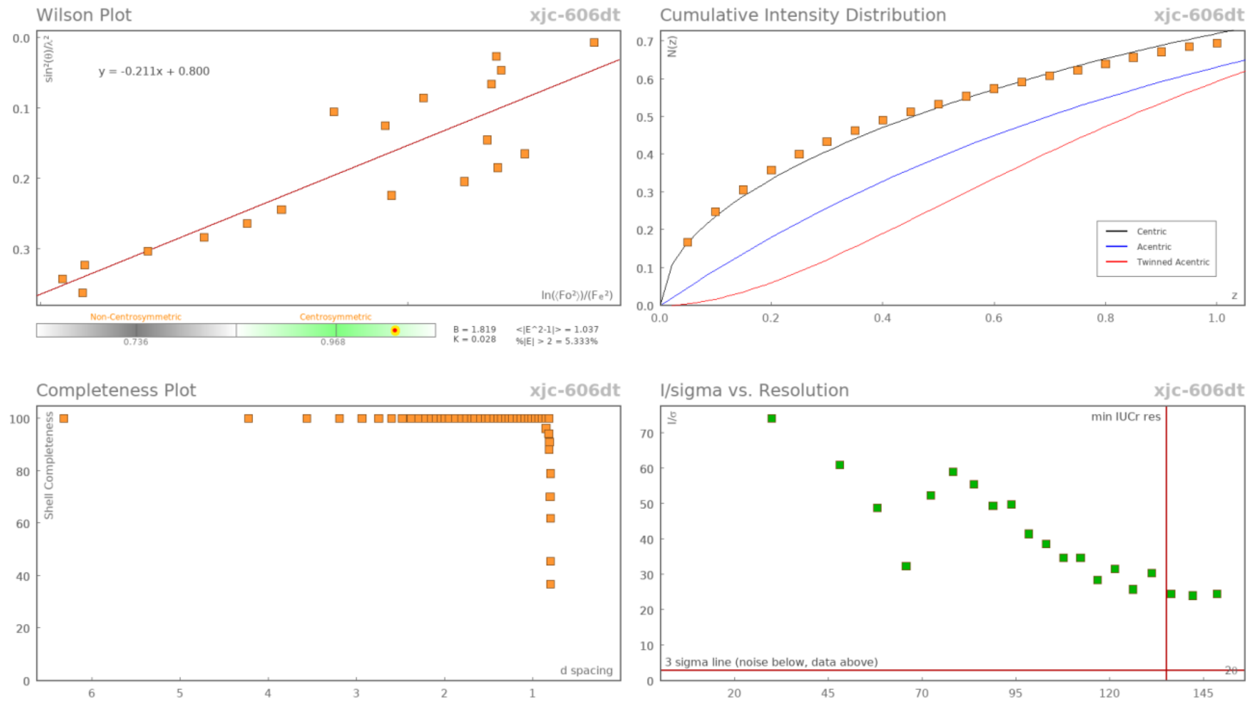

Supplementary Figure 16: Data Plots: Refinement and Data

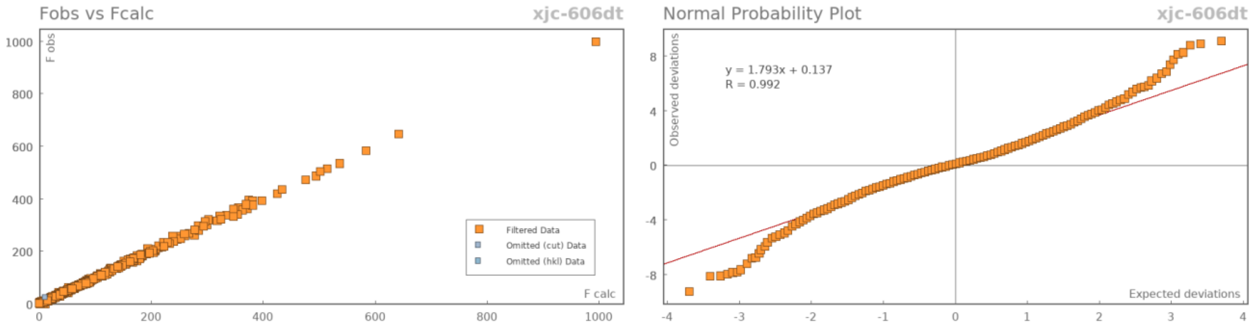

Supplementary Table 37: Reflection Statistics

Total reflections (after filtering)	20354	Unique reflections	4538

Completeness	0.987	Mean I/σ	41.36
hkl_{max} collected	(11, 11, 17)	hkl_{min} collected	(-10, -12, -17)
hkl_{max} used	(11, 11, 17)	hkl_{min} used	(-10, -12, 0)
Lim d_{max} collected	100.0	Lim d_{min} collected	0.77
d_{max} used	12.67	d_{min} used	0.79
Friedel pairs	3927	Friedel pairs merged	1
Inconsistent equivalents	7	R_{int}	0.0192
R_{sigma}	0.0136	Intensity transformed	0
Omitted reflections	0	Omitted by user (OMIT hkl)	2
Multiplicity	(1982, 2935, 2279, 868, 281, 82, 29, 7, 3, 1)	Maximum multiplicity	13
Removed systematic absences	0	Filtered off (Shel/OMIT)	0

Supplementary Table 38: Fractional Atomic Coordinates (×10[4]) and Equivalent Isotropic Displacement Parameters (Å[2]×10[3]) for **xjc-606dt.** U_{eq} is defined as 1/3 of the trace of the orthogonalised U_{ij}.

Atom	x	y	z	U_{eq}
O1	7563.5(9)	9873.8(8)	4271.2(6)	26.00(18)
O2	5480.5(9)	10361.1(8)	1471.5(6)	22.86(17)
O3	17.0(9)	7900.9(10)	-188.4(7)	31.02(19)
O4	-1404.1(9)	6919.0(8)	1225.1(6)	26.01(18)
O5	334.2(9)	6531.1(8)	2747.5(6)	22.66(17)
O6	3519.2(9)	2375.6(8)	1747.1(6)	24.41(17)
O7	3210.3(9)	2224.0(8)	3645.7(6)	23.58(17)
O8	2856.9(10)	4527.2(8)	5328.9(6)	27.76(18)
C1	3581.1(12)	7656.7(10)	3222.6(8)	17.4(2)
C2	5329.8(11)	8223.8(11)	3222.1(8)	17.9(2)
C3	6306.2(12)	8701.3(12)	4264.5(8)	22.0(2)
C4	7394.3(12)	10142.7(12)	3313.7(8)	22.4(2)
C5	5668.9(11)	9588.4(10)	2959.4(8)	18.0(2)
C6	5165.7(12)	9159.6(11)	1807.8(8)	18.2(2)
C7	3435.7(12)	8395.6(10)	1608.9(8)	18.0(2)
C8	2562.3(12)	8455.3(11)	769.9(8)	20.7(2)
C9	961.4(12)	7911.2(11)	613.2(8)	22.1(2)
C10	205.2(12)	7316.4(11)	1310.4(8)	20.9(2)
C11	1088.1(12)	7195.7(10)	2109.5(8)	19.2(2)
C12	2712.5(11)	7721.1(10)	2274.3(7)	17.5(2)
C13	753.0(17)	8418(2)	-951.2(12)	43.4(3)
C14	-2005.6(15)	5398.6(14)	711.3(12)	36.2(3)
C15	-210.3(16)	7514.7(14)	3653.3(11)	34.4(3)
C16	3320.0(11)	6167.9(11)	3302.0(8)	18.5(2)
C17	3447.5(12)	4954.1(11)	2428.4(8)	20.0(2)
C18	3378.9(12)	3634.3(11)	2546.5(8)	19.6(2)
C19	3179.1(12)	3519.2(11)	3529.9(8)	20.2(2)
C20	3041.1(12)	4739.4(11)	4399.5(8)	21.0(2)
C21	3109.1(12)	6064.1(11)	4283.3(8)	20.9(2)
C22	3784.1(14)	2439.6(12)	730.1(8)	24.1(2)
C23	1702.6(14)	1287.5(12)	3484.4(10)	27.8(2)
C24	2696.0(17)	5744.9(14)	6234.1(9)	32.9(3)

Supplementary Table 39: Anisotropic Displacement Parameters (×10[4]) for **xjc-606dt.** The anisotropic displacement factor exponent takes the form: $-2\pi^2[h^2a^{*2} \times U_{11} + ... + 2hka^* \times b^* \times U_{12}]$

Atom	U_{11}	U_{22}	U_{33}	U_{23}	U_{13}	U_{12}
O1	26.3(4)	27.5(4)	23.9(4)	11.4(3)	-3.7(3)	1.6(3)
O2	24.3(4)	23.0(4)	27.4(4)	16.5(3)	4.3(3)	4.7(3)
O3	25.6(4)	41.7(5)	30.5(4)	21.9(4)	-5.7(3)	1.9(3)
O4	19.2(4)	24.1(4)	33.2(4)	10.1(3)	-0.3(3)	2.3(3)
O5	24.9(4)	20.2(4)	25.4(4)	12.0(3)	6.9(3)	3.7(3)

Atom	U_{11}	U_{22}	U_{33}	U_{23}	U_{13}	U_{12}
O6	38.4(4)	17.8(4)	20.3(4)	8.7(3)	6.5(3)	10.1(3)
O7	28.3(4)	18.4(4)	28.9(4)	14.2(3)	1.7(3)	5.5(3)
O8	45.1(5)	23.0(4)	20.0(4)	13.1(3)	7.5(3)	8.1(3)
C1	21.3(5)	15.9(5)	17.3(4)	7.9(4)	3.7(4)	5.6(4)
C2	21.3(5)	17.6(5)	17.7(4)	8.6(4)	3.4(4)	6.9(4)
C3	22.1(5)	25.5(5)	21.1(5)	11.2(4)	1.0(4)	6.4(4)
C4	22.2(5)	22.3(5)	23.6(5)	10.3(4)	0.0(4)	3.5(4)
C5	19.9(5)	16.4(5)	19.1(5)	7.7(4)	2.8(4)	4.9(4)
C6	21.0(5)	17.7(5)	19.2(5)	10.3(4)	3.7(4)	4.7(4)
C7	21.2(5)	15.7(4)	18.1(4)	6.7(4)	2.2(4)	5.4(4)
C8	24.2(5)	19.8(5)	20.1(5)	10.2(4)	2.1(4)	4.3(4)
C9	24.8(5)	21.1(5)	21.4(5)	8.8(4)	-2.6(4)	5.4(4)
C10	19.2(5)	17.5(5)	25.2(5)	7.1(4)	0.7(4)	3.5(4)
C11	23.1(5)	14.7(4)	21.0(5)	7.7(4)	4.4(4)	4.9(4)
C12	21.5(5)	14.0(4)	18.4(4)	6.8(4)	2.7(4)	5.7(4)
C13	34.7(7)	67.8(10)	37.9(7)	36.7(7)	-6.8(6)	1.1(7)
C14	28.2(6)	26.9(6)	45.8(8)	9.4(5)	-3.9(5)	-3.2(5)
C15	36.8(7)	32.3(6)	36.3(6)	14.4(5)	19.4(5)	9.3(5)
C16	19.7(5)	17.6(5)	21.2(5)	10.1(4)	2.8(4)	5.1(4)
C17	24.6(5)	19.8(5)	19.1(5)	10.6(4)	3.5(4)	6.0(4)
C18	22.2(5)	17.0(5)	20.5(5)	7.3(4)	2.4(4)	5.6(4)
C19	22.5(5)	18.1(5)	24.3(5)	12.6(4)	2.1(4)	4.8(4)
C20	24.5(5)	22.6(5)	19.6(5)	12.3(4)	3.7(4)	4.9(4)
C21	25.5(5)	19.0(5)	19.8(5)	8.6(4)	4.6(4)	5.9(4)
C22	33.6(6)	21.4(5)	19.5(5)	8.8(4)	4.5(4)	8.2(4)
C23	33.3(6)	20.9(5)	29.7(6)	12.8(5)	0.9(5)	0.7(4)
C24	51.9(8)	28.1(6)	20.7(5)	11.3(5)	8.1(5)	8.6(6)

Supplementary Table 40: Bond Lengths in Å for **xjc-606dt**.

Atom	Atom	Length/Å
O1	C3	1.4457(13)
O1	C4	1.4412(12)
O2	C6	1.4274(12)
O3	C9	1.3666(12)
O3	C13	1.4261(15)
O4	C10	1.3791(12)
O4	C14	1.4249(15)
O5	C11	1.3764(12)
O5	C15	1.4309(14)
O6	C18	1.3730(12)
O6	C22	1.4296(12)
O7	C19	1.3825(12)
O7	C23	1.4276(14)
O8	C20	1.3654(12)
O8	C24	1.4256(14)
C1	C2	1.5247(14)
C1	C12	1.5287(13)
C1	C16	1.5222(13)

Atom	Atom	Length/Å
C2	C3	1.5236(13)
C2	C5	1.5236(13)
C4	C5	1.5151(14)
C5	C6	1.5096(13)
C6	C7	1.5317(14)
C7	C8	1.4025(14)
C7	C12	1.4033(13)
C8	C9	1.3859(15)
C9	C10	1.4013(15)
C10	C11	1.3911(14)
C11	C12	1.4030(14)
C16	C17	1.3920(14)
C16	C21	1.3903(14)
C17	C18	1.3928(14)
C18	C19	1.3967(14)
C19	C20	1.3952(15)
C20	C21	1.3967(14)

Supplementary Table 41: Bond Angles in ° for **xjc-606dt**.

Atom	Atom	Atom	Angle/°
C4	O1	C3	109.23(8)
C9	O3	C13	117.20(9)
C10	O4	C14	113.93(9)
C11	O5	C15	113.24(8)
C18	O6	C22	117.37(8)
C19	O7	C23	113.27(8)

Atom	Atom	Atom	Angle/°
C20	O8	C24	117.14(8)
C2	C1	C12	109.78(8)
C16	C1	C2	108.82(8)
C16	C1	C12	115.51(8)
C3	C2	C1	116.65(8)
C5	C2	C1	111.86(8)

Atom	Atom	Atom	Angle/°
C5	C2	C3	102.54(8)
O1	C3	C2	105.89(8)
O1	C4	C5	104.55(8)
C4	C5	C2	100.72(8)
C6	C5	C2	108.03(8)
C6	C5	C4	118.45(8)
O2	C6	C5	112.48(8)
O2	C6	C7	112.34(8)
C5	C6	C7	108.77(8)
C8	C7	C6	117.94(9)
C8	C7	C12	120.42(9)
C12	C7	C6	121.56(9)
C9	C8	C7	120.68(9)
O3	C9	C8	124.95(10)
O3	C9	C10	115.44(9)
C8	C9	C10	119.61(9)
O4	C10	C9	119.67(9)
O4	C10	C11	120.90(9)
C11	C10	C9	119.37(9)
O5	C11	C10	118.49(9)

Atom	Atom	Atom	Angle/°
O5	C11	C12	119.60(9)
C10	C11	C12	121.90(9)
C7	C12	C1	123.22(9)
C11	C12	C1	118.71(8)
C11	C12	C7	117.83(9)
C17	C16	C1	120.41(9)
C21	C16	C1	118.85(9)
C21	C16	C17	120.42(9)
C16	C17	C18	119.46(9)
O6	C18	C17	124.54(9)
O6	C18	C19	114.82(9)
C17	C18	C19	120.64(9)
O7	C19	C18	119.79(9)
O7	C19	C20	120.60(9)
C20	C19	C18	119.50(9)
O8	C20	C19	115.44(9)
O8	C20	C21	124.57(9)
C19	C20	C21	119.99(9)
C16	C21	C20	119.99(9)

Supplementary Table 42: Torsion Angles in ° for **xjc-606dt**.

Atom	Atom	Atom	Atom	Angle/°
O1	C4	C5	C2	-39.18(9)
O1	C4	C5	C6	-156.62(8)
O2	C6	C7	C8	28.09(12)
O2	C6	C7	C12	-148.48(9)
O3	C9	C10	O4	-6.99(14)
O3	C9	C10	C11	175.86(9)
O4	C10	C11	O5	6.66(14)
O4	C10	C11	C12	-173.77(9)
O5	C11	C12	C1	-5.26(13)
O5	C11	C12	C7	-179.80(8)
O6	C18	C19	O7	3.05(14)
O6	C18	C19	C20	179.32(9)
O7	C19	C20	O8	-3.46(15)
O7	C19	C20	C21	176.00(9)
O8	C20	C21	C16	179.17(10)
C1	C2	C3	O1	-147.90(8)
C1	C2	C5	C4	164.55(8)
C1	C2	C5	C6	-70.59(10)
C1	C16	C17	C18	172.81(9)
C1	C16	C21	C20	-172.89(9)
C2	C1	C12	C7	-8.05(13)
C2	C1	C12	C11	177.72(8)
C2	C1	C16	C17	-75.69(11)
C2	C1	C16	C21	97.91(10)
C2	C5	C6	O2	-178.16(8)
C2	C5	C6	C7	56.73(10)
C3	O1	C4	C5	24.44(10)
C3	C2	C5	C4	38.80(9)
C3	C2	C5	C6	163.66(8)
C4	O1	C3	C2	0.78(10)
C4	C5	C6	O2	-64.65(11)
C4	C5	C6	C7	170.24(8)
C5	C2	C3	O1	-25.33(10)
C5	C6	C7	C8	153.28(9)
C5	C6	C7	C12	-23.29(12)

Atom	Atom	Atom	Atom	Angle/˚
C6	C7	C8	C9	-173.59(9)
C6	C7	C12	C1	-1.59(14)
C6	C7	C12	C11	172.70(9)
C7	C8	C9	O3	-178.99(10)
C7	C8	C9	C10	1.01(15)
C8	C7	C12	C1	-178.08(9)
C8	C7	C12	C11	-3.80(14)
C8	C9	C10	O4	173.01(9)
C8	C9	C10	C11	-4.14(15)
C9	C10	C11	O5	-176.23(9)
C9	C10	C11	C12	3.34(15)
C10	C11	C12	C1	175.17(9)
C10	C11	C12	C7	0.62(14)
C12	C1	C2	C3	160.65(8)
C12	C1	C2	C5	43.07(10)
C12	C1	C16	C17	48.29(13)
C12	C1	C16	C21	-138.12(9)
C12	C7	C8	C9	3.03(15)
C13	O3	C9	C8	3.61(17)
C13	O3	C9	C10	-176.39(11)
C14	O4	C10	C9	101.57(12)
C14	O4	C10	C11	-81.33(13)
C15	O5	C11	C10	-89.13(12)
C15	O5	C11	C12	91.28(12)
C16	C1	C2	C3	-72.04(11)
C16	C1	C2	C5	170.38(8)
C16	C1	C12	C7	-131.52(10)
C16	C1	C12	C11	54.25(12)
C16	C17	C18	O6	-178.77(9)
C16	C17	C18	C19	0.20(15)
C17	C16	C21	C20	0.70(16)
C17	C18	C19	O7	-176.01(9)
C17	C18	C19	C20	0.26(16)
C18	C19	C20	O8	-179.70(9)
C18	C19	C20	C21	-0.24(16)
C19	C20	C21	C16	-0.24(16)
C21	C16	C17	C18	-0.68(15)
C22	O6	C18	C17	1.27(15)
C22	O6	C18	C19	-177.75(9)
C23	O7	C19	C18	-91.61(12)
C23	O7	C19	C20	92.16(12)
C24	O8	C20	C19	-179.39(10)
C24	O8	C20	C21	1.18(16)

Supplementary Table 43: Hydrogen Fractional Atomic Coordinates (×10^4) and Equivalent Isotropic Displacement Parameters (Å^2×10^3) for **xjc-606dt**. U_{eq} is defined as 1/3 of the trace of the orthogonalised U_{ij}.

Atom	x	y	z	*Ueq*
H2	4850(20)	10927(18)	1782(13)	43(4)
H1	3237(14)	8315(14)	3870(10)	18(3)
H2A	5748(14)	7475(13)	2678(10)	17(3)
H3A	5704(15)	9093(14)	4883(10)	23(3)
H3B	6747(15)	7916(14)	4345(10)	22(3)
H4A	8026(15)	9612(14)	2789(10)	20(3)

Atom	x	y	z	U_{eq}
H4B	7773(16)	11203(15)	3487(11)	26(3)
H5	5082(15)	10308(14)	3387(10)	19(3)
H6	5817(14)	8485(13)	1385(9)	15(3)
H8	3089(15)	8894(14)	302(10)	24(3)
H13A	1560(20)	7847(18)	-1263(13)	46(4)
H13B	1260(20)	9490(20)	-612(14)	53(5)
H13C	-60(20)	8220(20)	-1496(15)	59(5)
H14A	-1600(20)	4871(18)	1084(13)	46(4)
H14B	-1730(20)	5055(19)	-37(15)	51(5)
H14C	-3120(20)	5258(19)	711(14)	49(5)
H15A	-717(19)	6939(18)	4050(13)	43(4)
H15B	-900(20)	7980(20)	3433(15)	60(5)
H15C	680(20)	8290(20)	4117(15)	55(5)
H17	3609(15)	5055(14)	1752(11)	23(3)
H21	3009(15)	6917(15)	4887(11)	25(3)
H22A	4771(17)	3141(16)	763(11)	30(3)
H22B	3883(16)	1460(16)	286(11)	30(3)
H22C	2913(18)	2682(16)	448(11)	32(4)
H23A	1789(19)	501(18)	3692(13)	43(4)
H23B	1324(19)	900(18)	2758(14)	43(4)
H23C	950(20)	1781(18)	3894(13)	44(4)
H24A	3592(19)	6582(18)	6380(12)	40(4)
H24B	1707(18)	6036(16)	6157(11)	33(4)
H24C	2633(19)	5393(17)	6811(13)	41(4)

1.7. Copies of the ^{1}H, ^{13}C and 2D NMR spectra

Supplementary Figure 17. ^{1}H NMR spectrum of compound **20a-A** (400 MHz, $CDCl_3$)

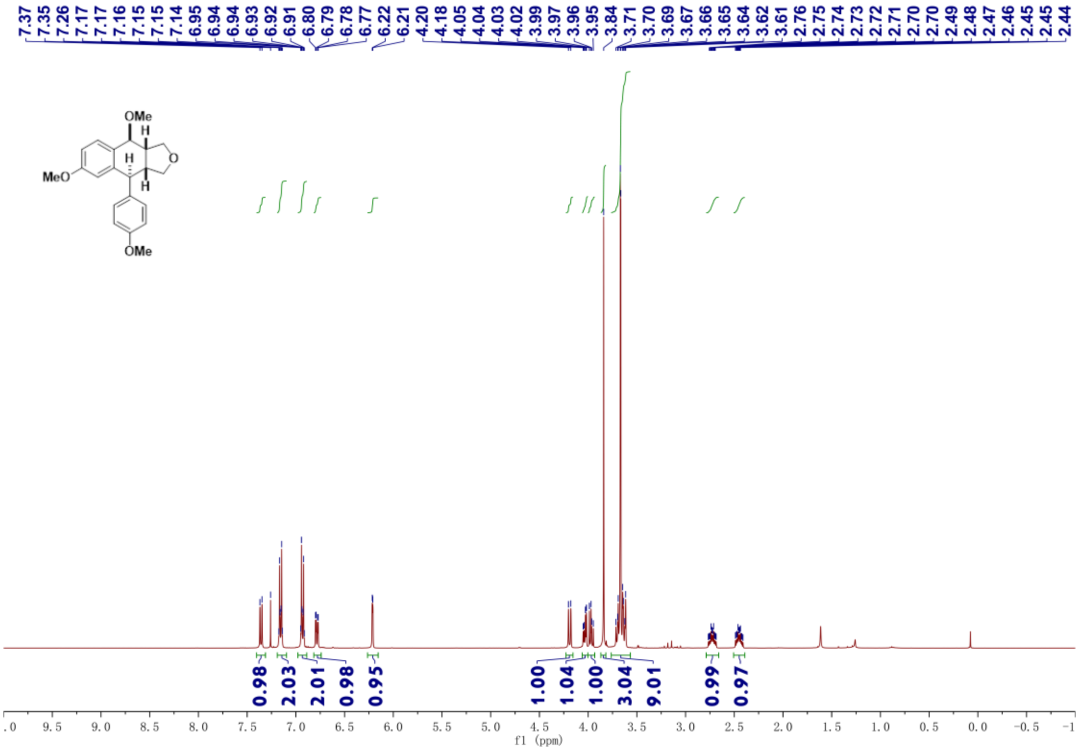

Supplementary Figure 18. ^{13}C NMR spectrum of compound **20a-A** (101 MHz, $CDCl_3$)

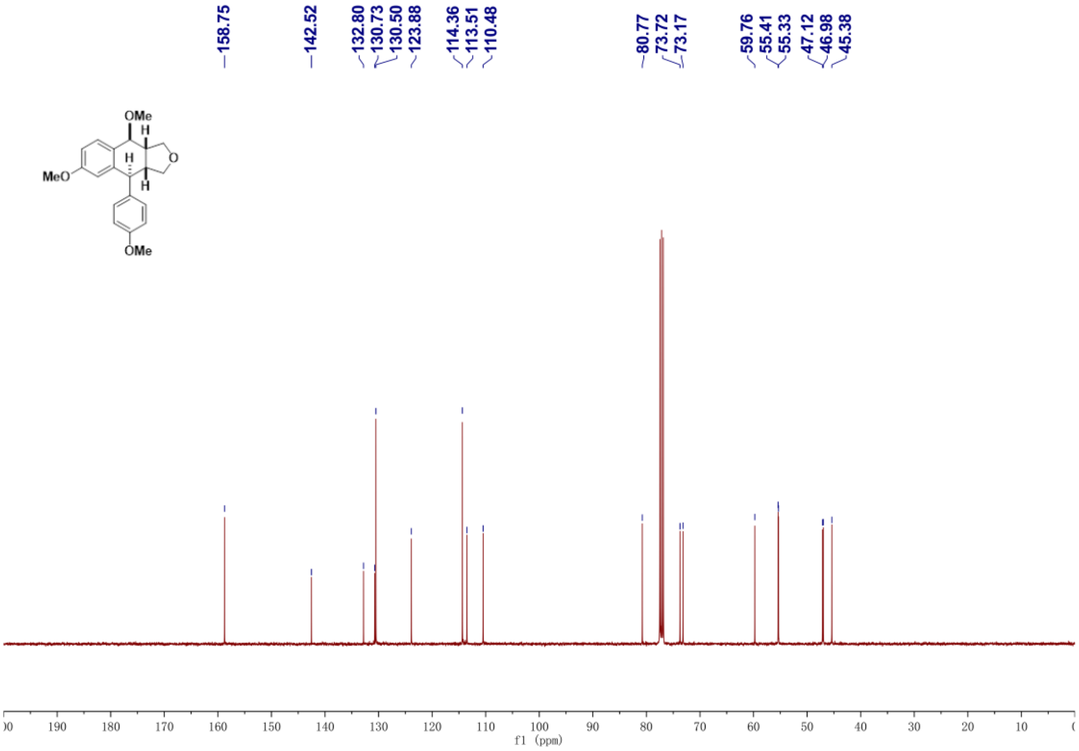

Supplementary Figure 19. 2D NMR spectra of compound **20a-A** ($CDCl_3$)

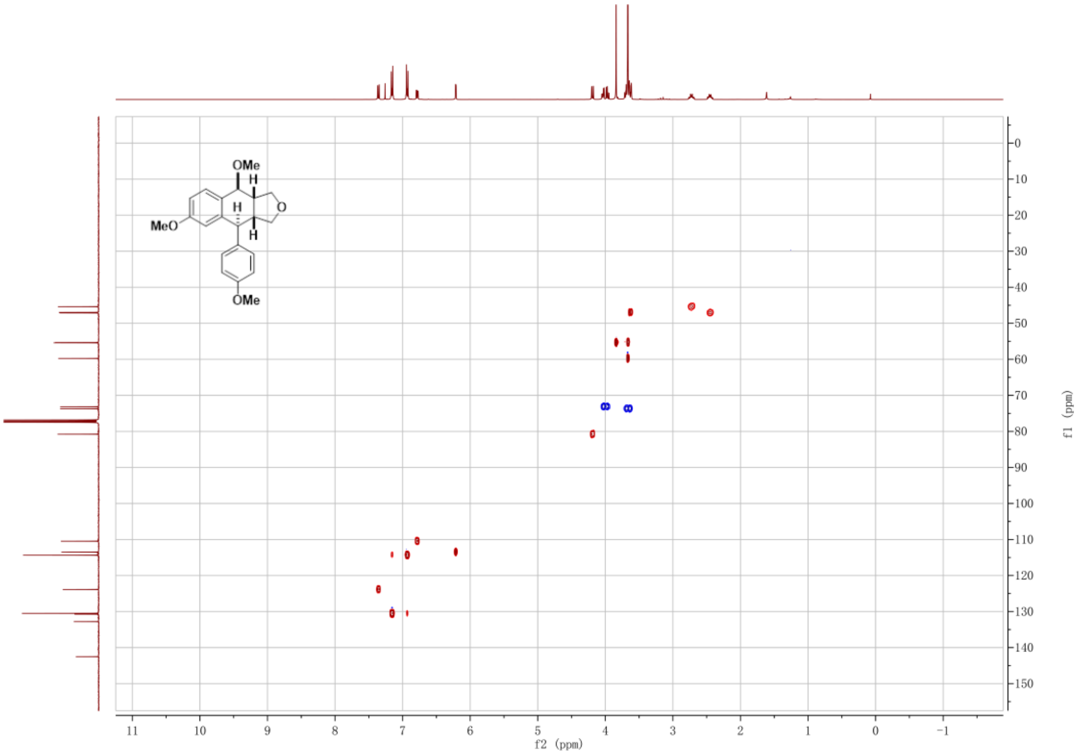

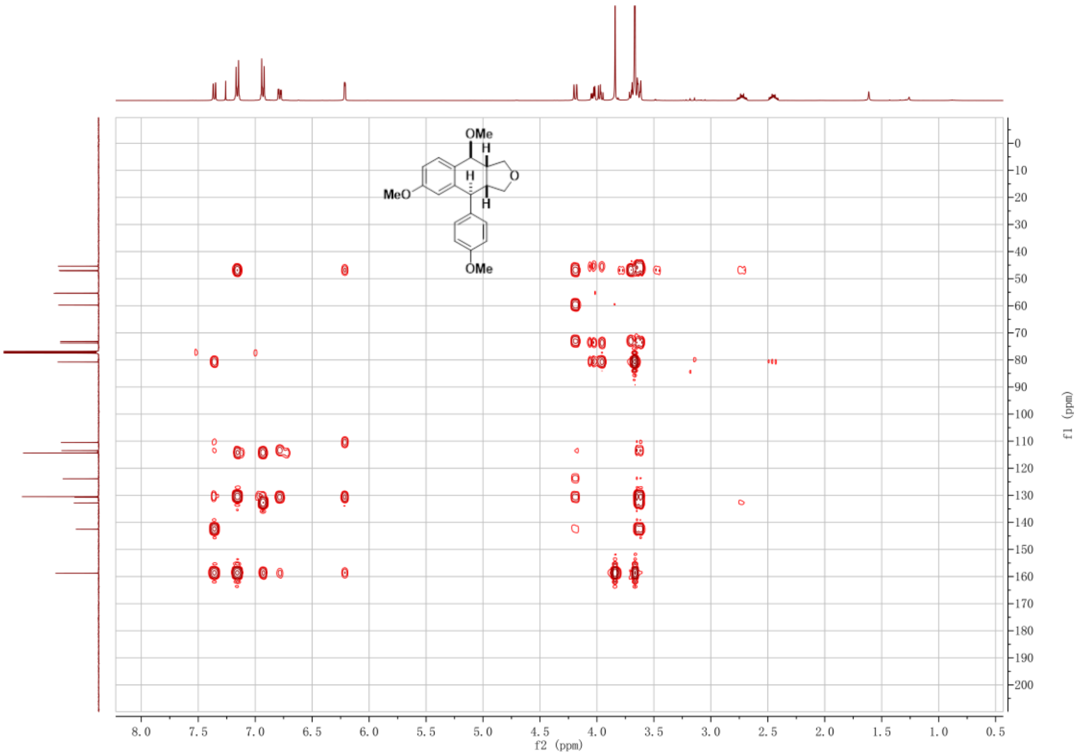

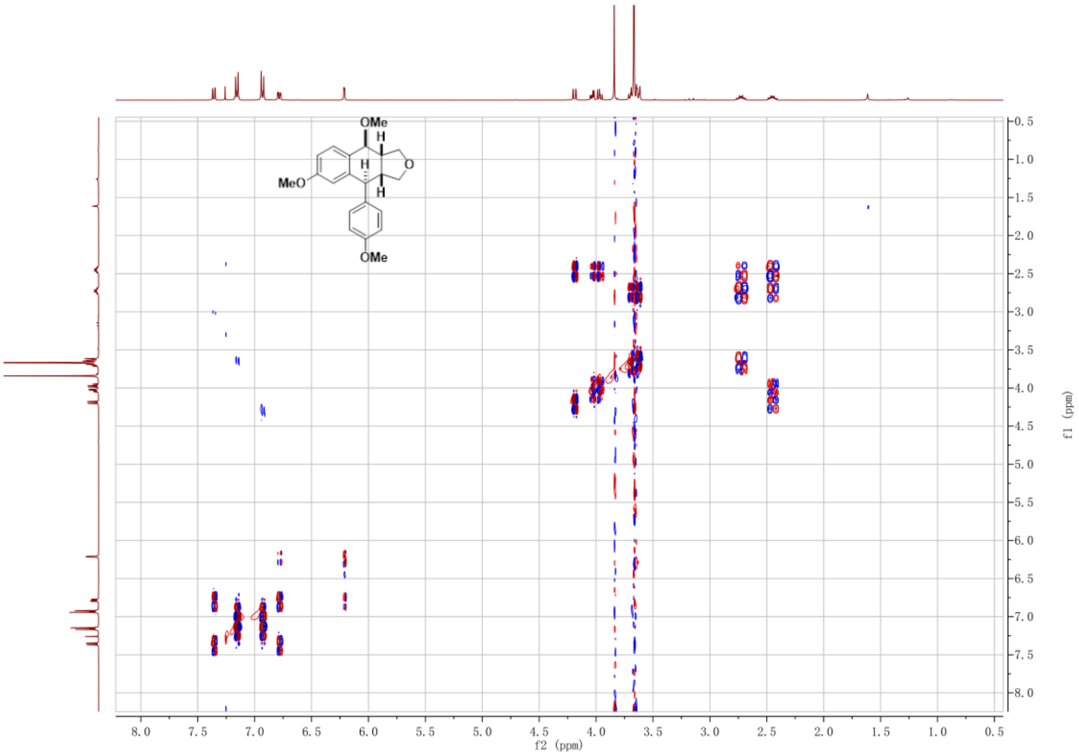
OMe
H
H
O
MeO
H
OMe
f2 (ppm)
f1 (ppm)

Supplementary Figure 20. ^{1}H NMR spectrum of compound **20a-B** (400 MHz, $CDCl_3$)

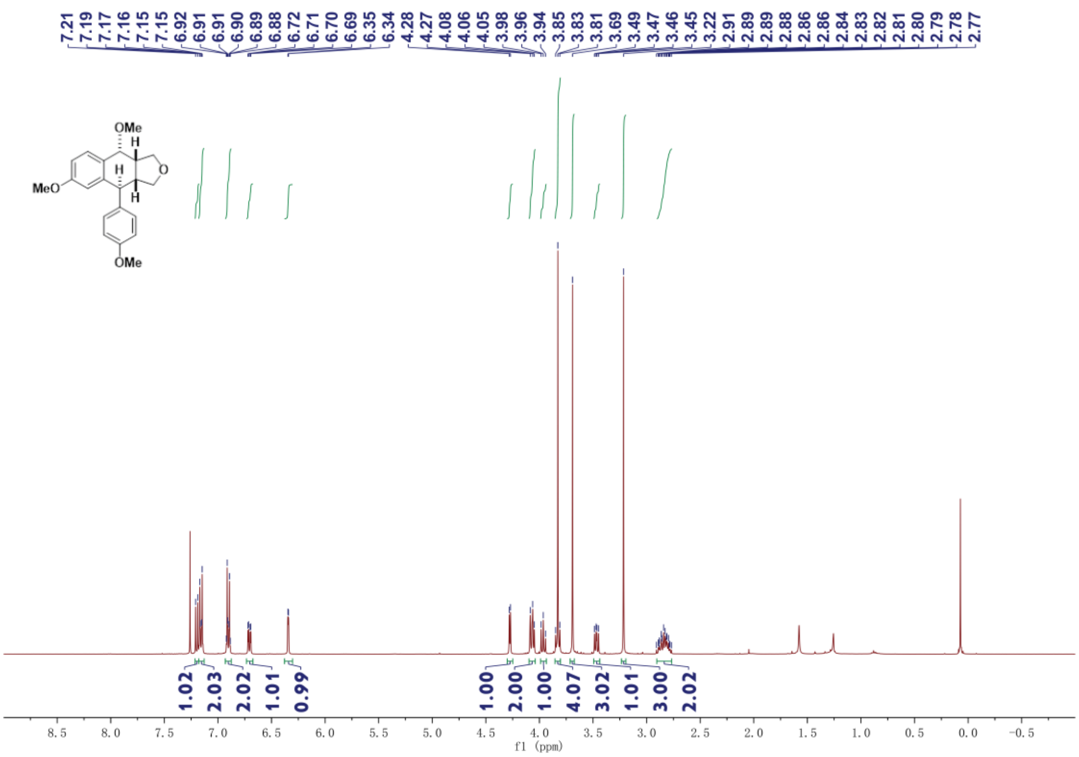

Supplementary Figure 21. ^{13}C NMR spectrum of compound **20a-B** (101 MHz, $CDCl_3$)

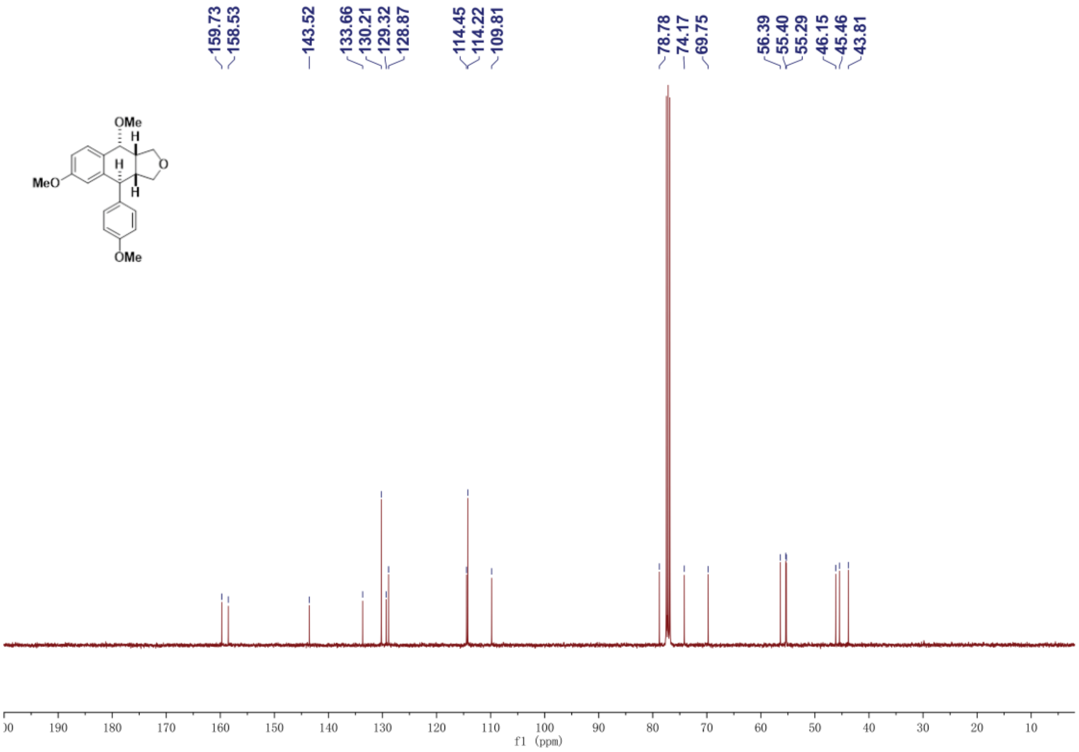

Supplementary Figure 22. 2D NMR spectra of compound **20a-B** ($CDCl_3$)

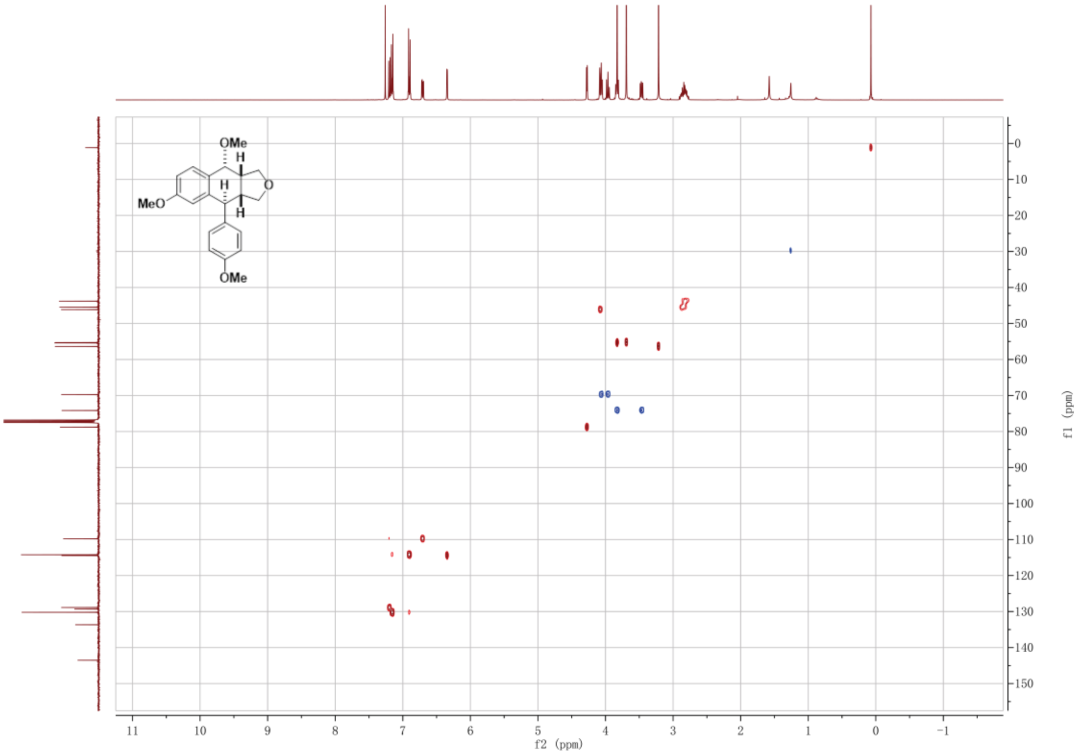

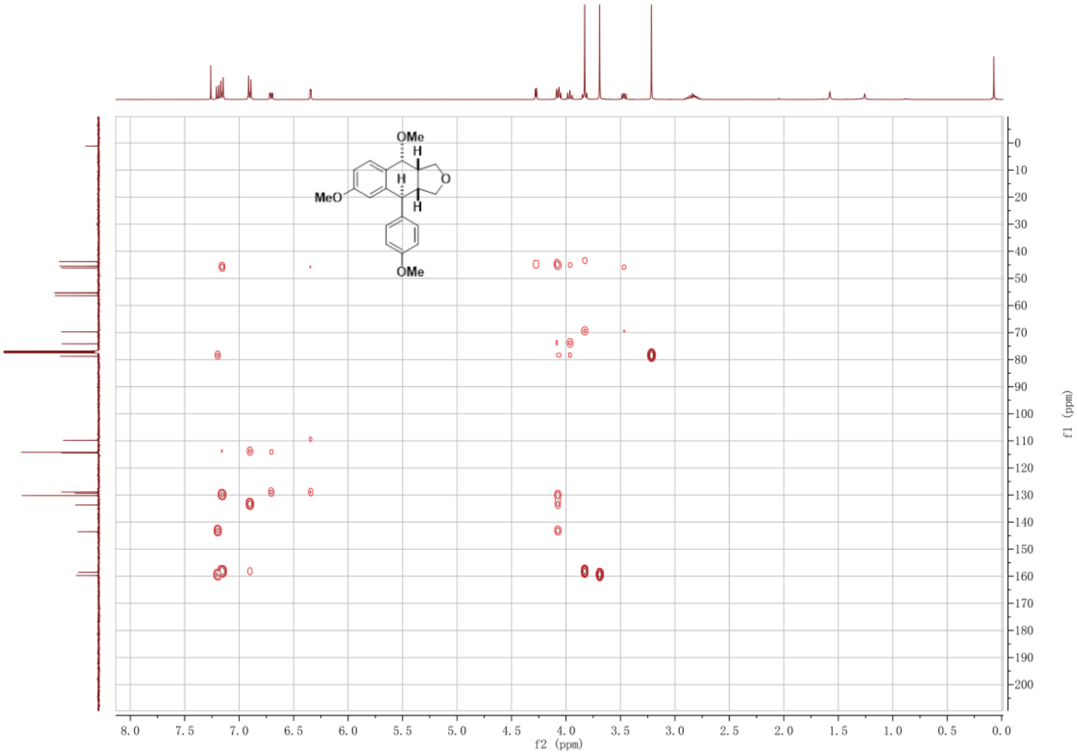

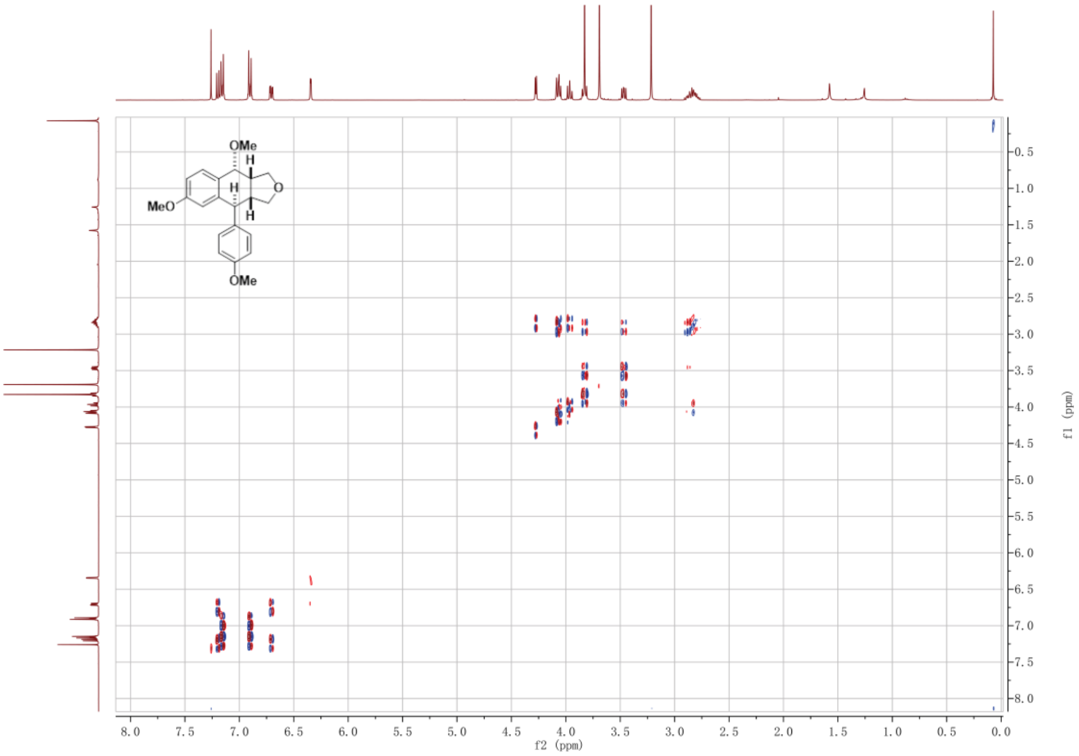
OMe
H
H
O
MeO
H
OMe
f2 (ppm)
f1 (ppm)

Supplementary Figure 23. ^{1}H NMR spectrum of compound **20b-A** (400 MHz, $CDCl_3$)

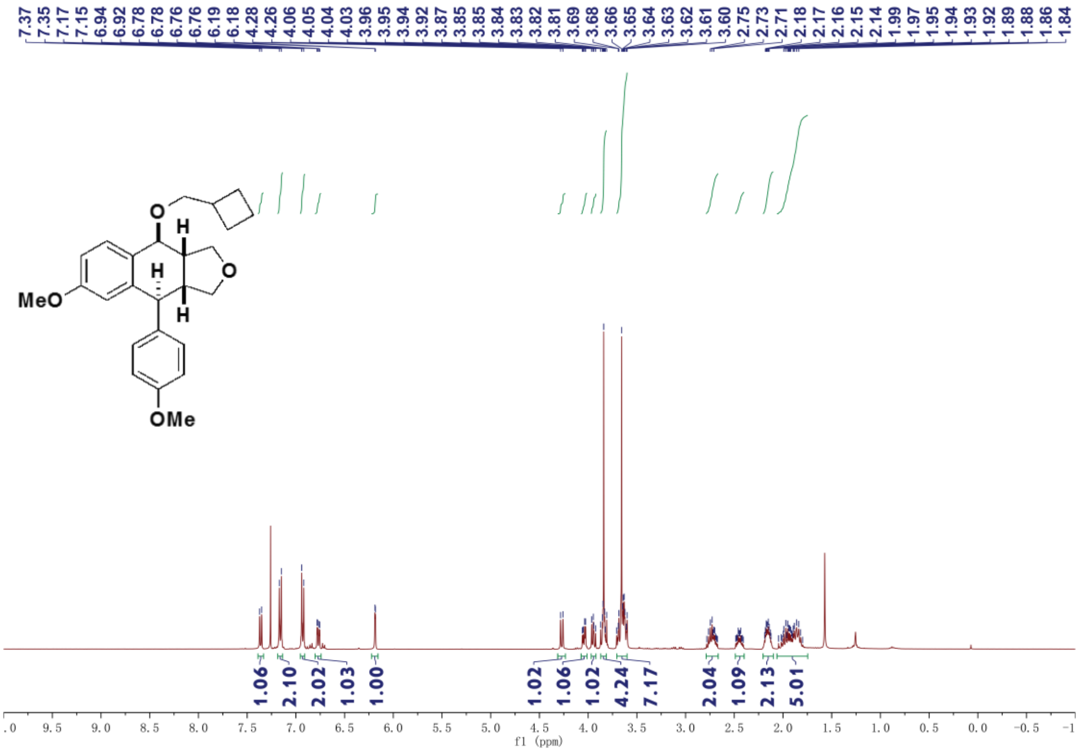

Supplementary Figure 24. ^{13}C NMR spectrum of compound **20b-A** (101 MHz, $CDCl_3$)

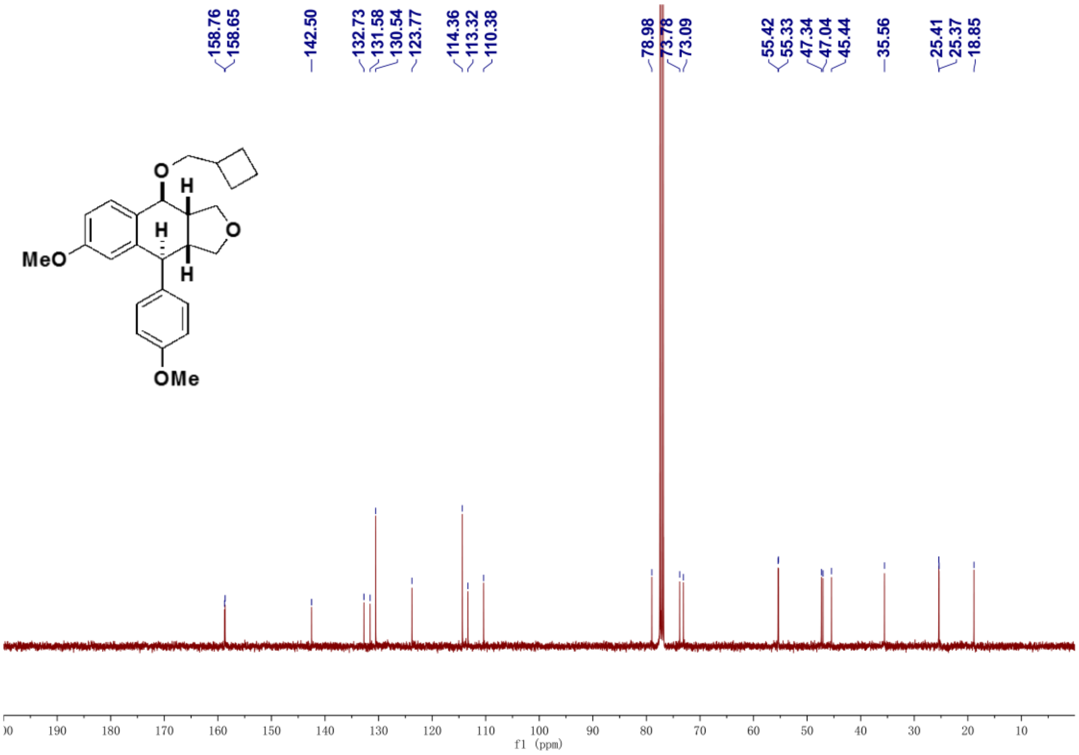

Supplementary Figure 25. ^{1}H NMR spectrum of compound **20b-B** (400 MHz, $CDCl_3$)

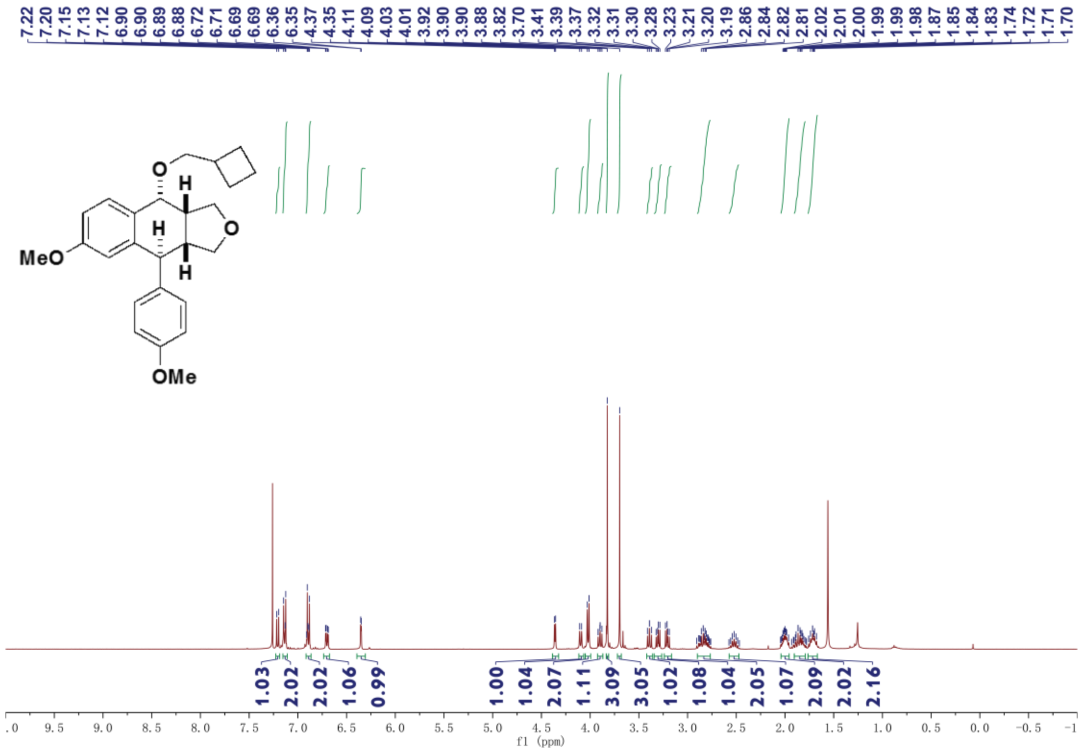

Supplementary Figure 26. ^{13}C NMR spectrum of compound **20b-B** (101 MHz, $CDCl_3$)

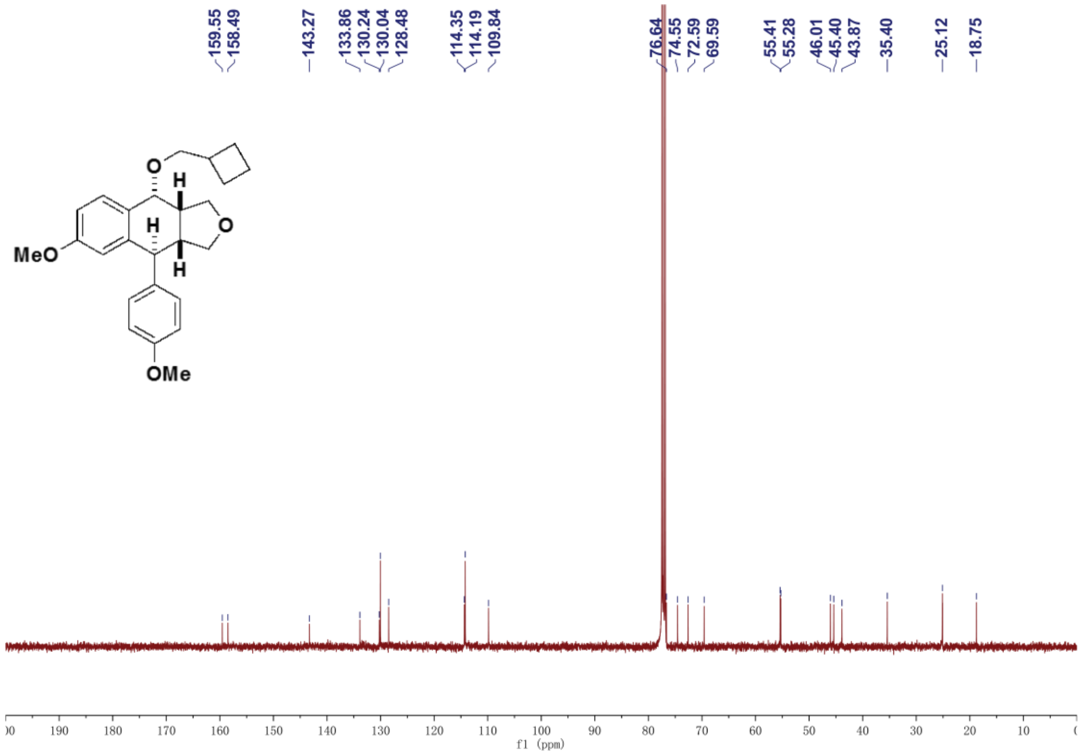

Supplementary Figure 27. ^{1}H NMR spectrum of compound **20c (A+B), major+minor** (400 MHz, $CDCl_3$)

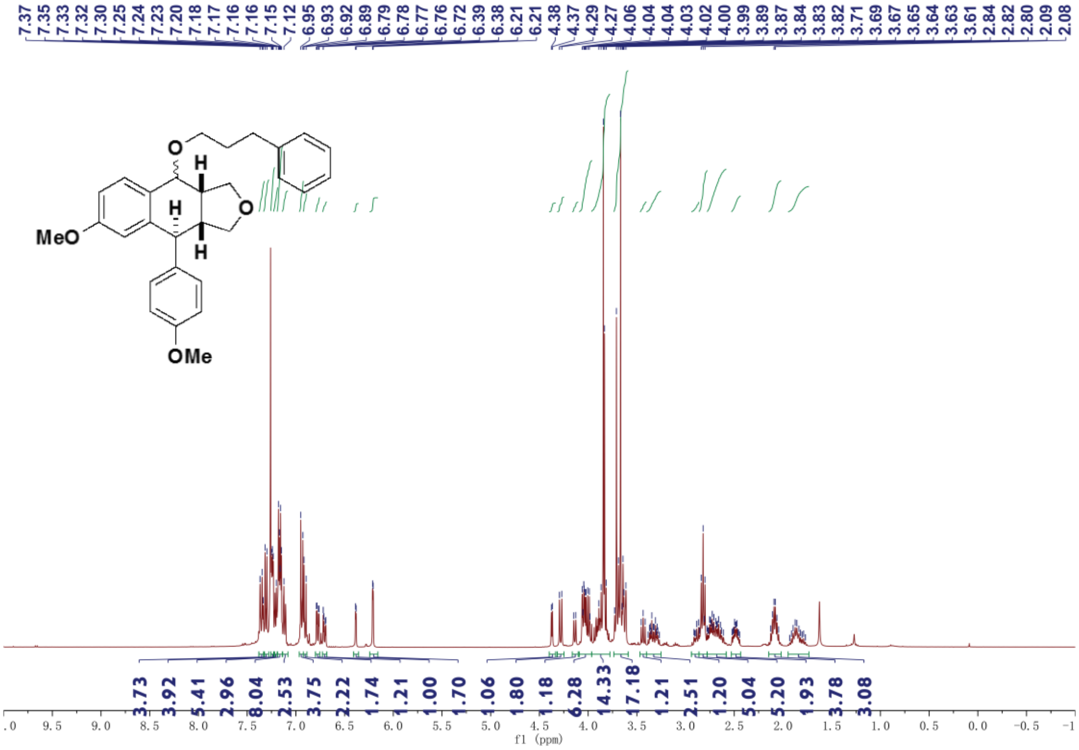

Supplementary Figure 28. ^{13}C NMR spectrum of compound **20c (A+B), major+minor** (101 MHz, $CDCl_3$)

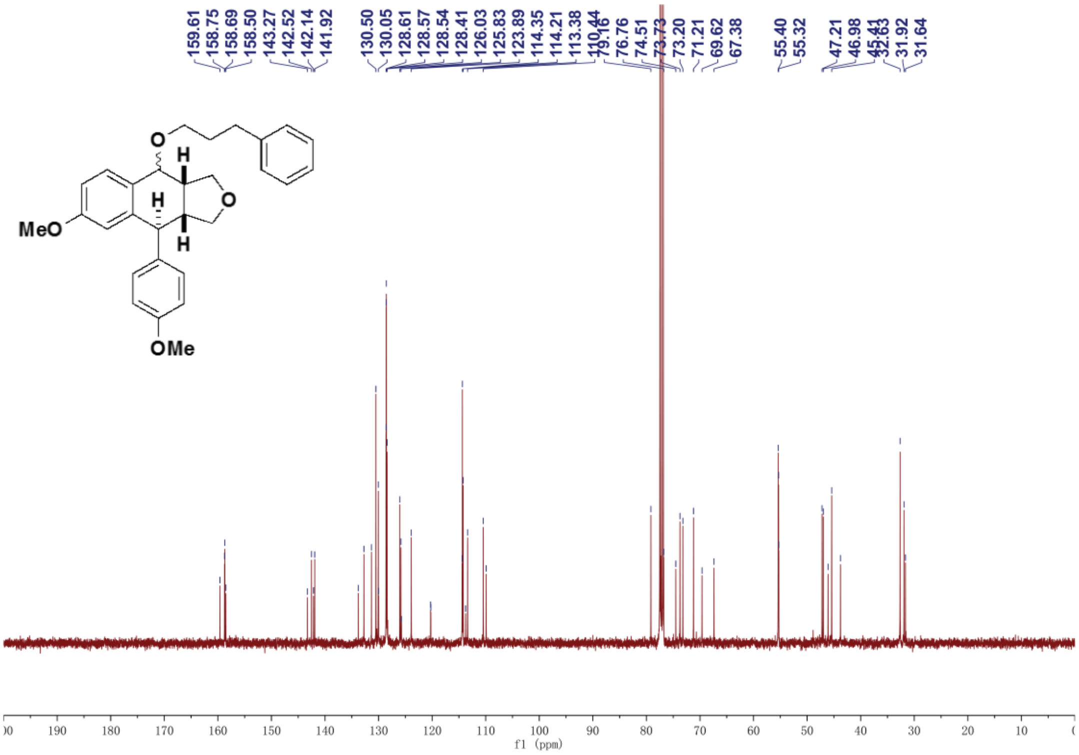

Supplementary Figure 29. ^{1}H NMR spectrum of compound **20d (A+B), major+minor** (400 MHz, $CDCl_3$)

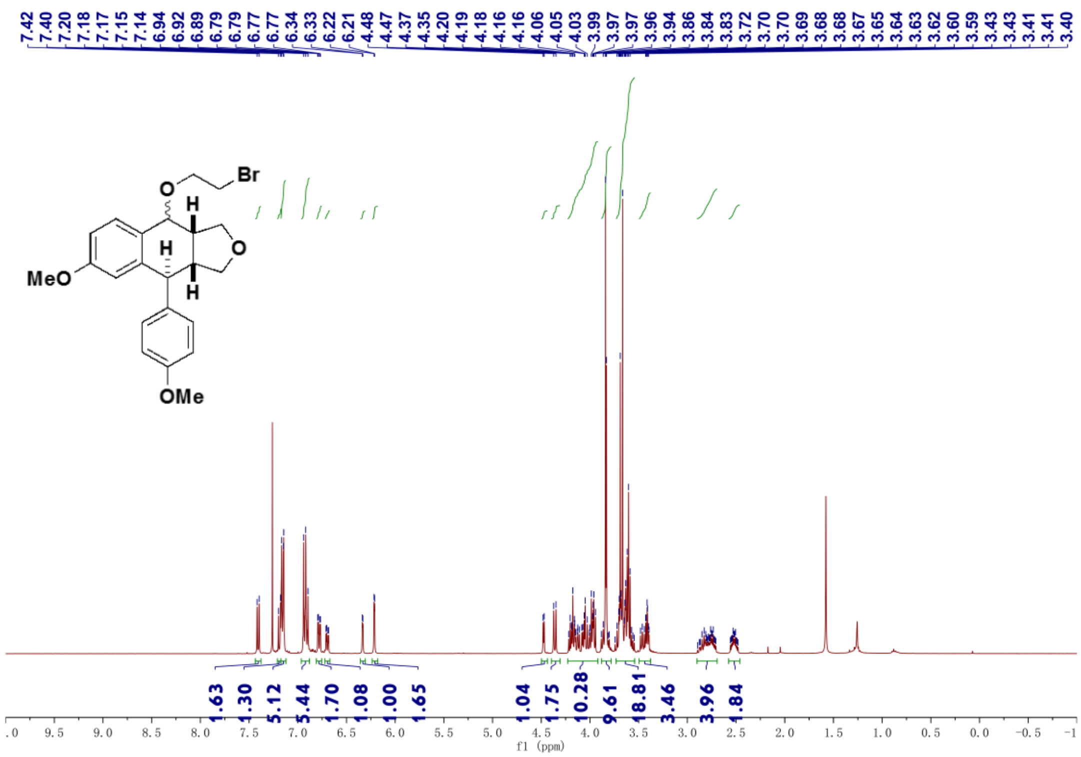

Supplementary Figure 30. ^{13}C NMR spectrum of compound **20d (A+B), major+minor** (101 MHz, $CDCl_3$)

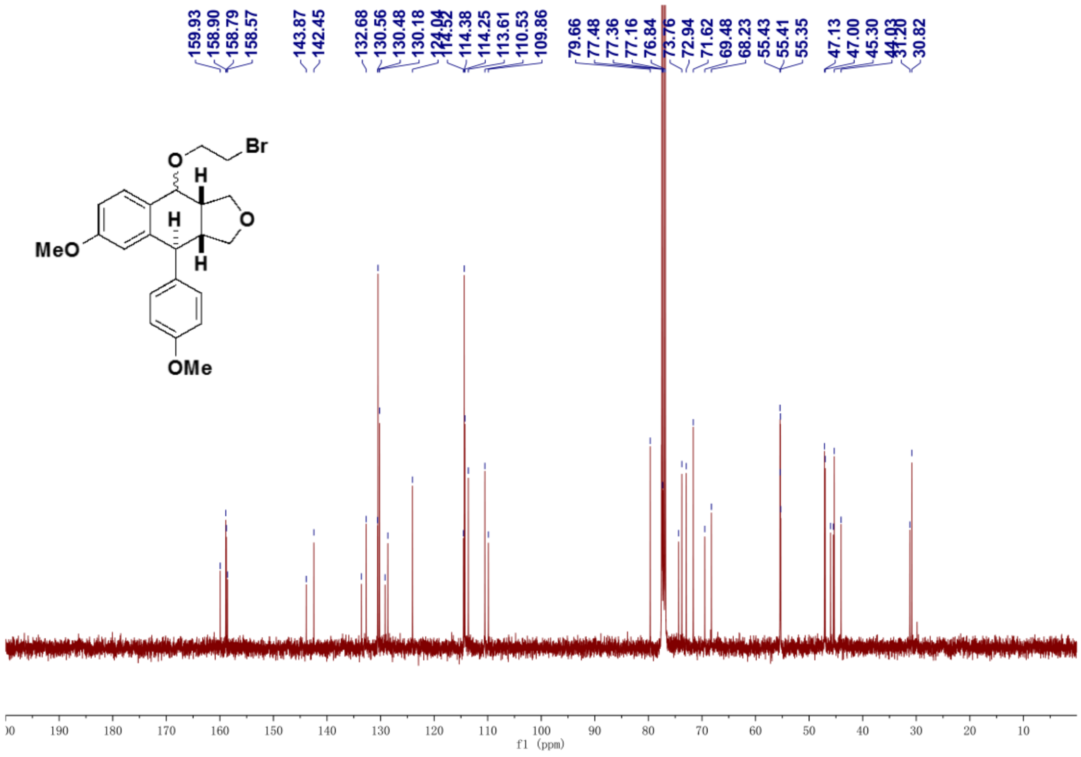

Supplementary Figure 31. ^{1}H NMR spectrum of compound **20e-A** (400 MHz, $CDCl_3$)

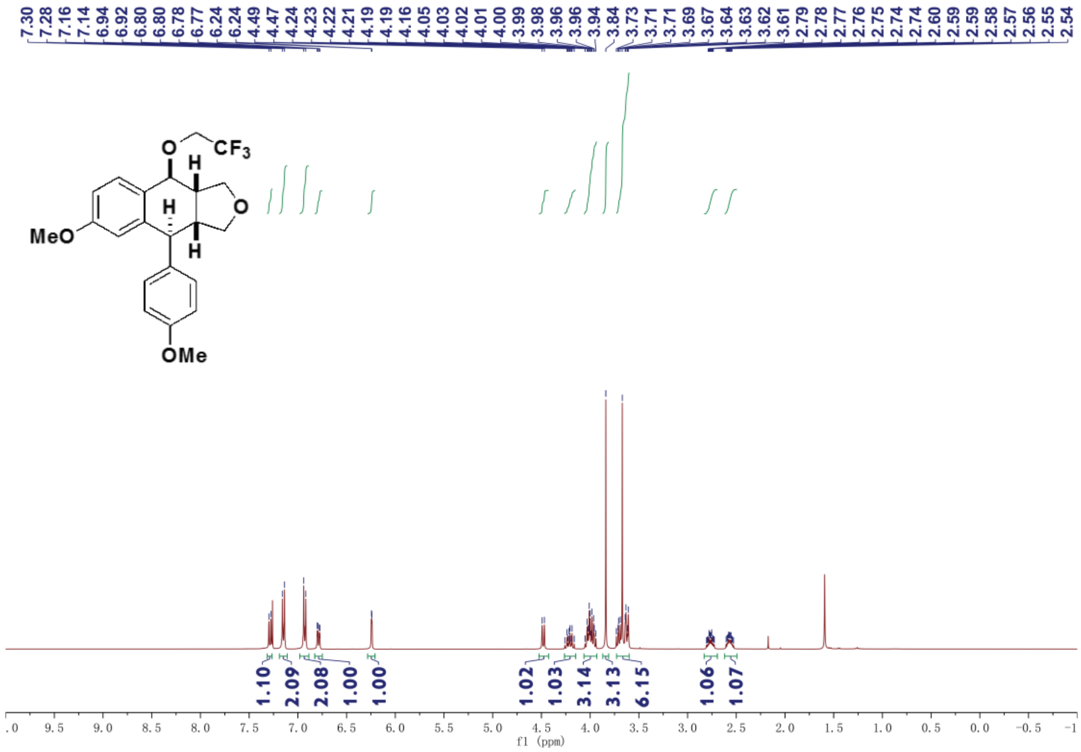

Supplementary Figure 32. ^{13}C NMR spectrum of compound **20e-A** (101 MHz, $CDCl_3$)

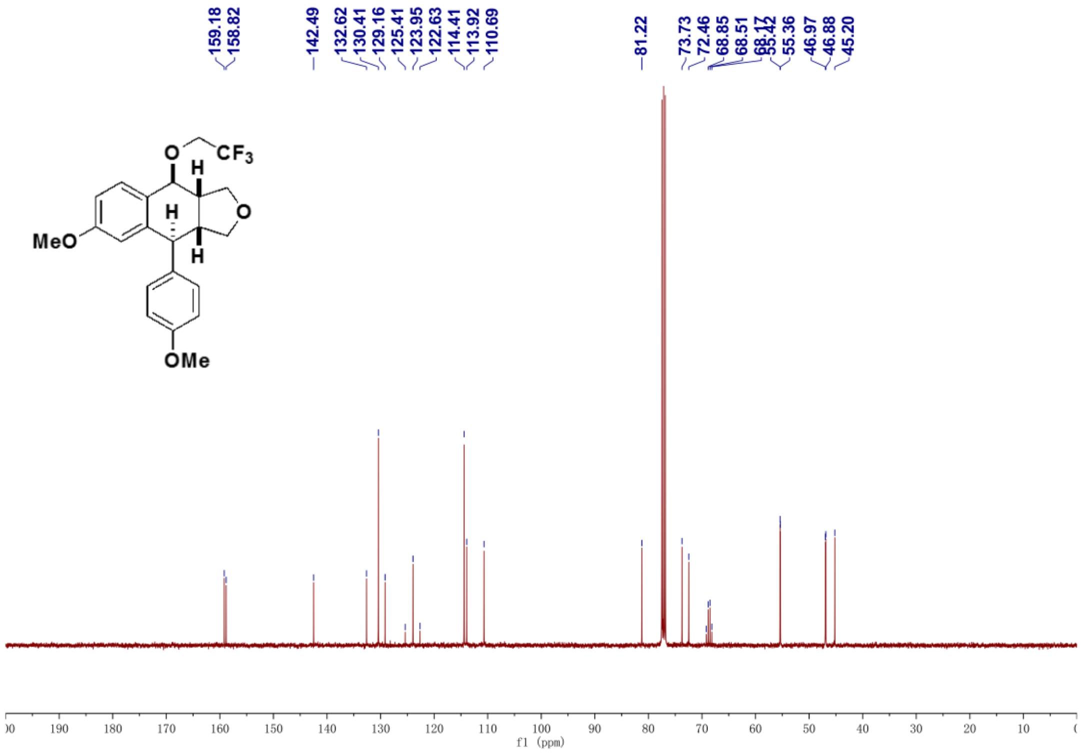

Supplementary Figure 33. ^{19}F NMR spectrum of compound **20e-A** (377 MHz, $CDCl_3$)

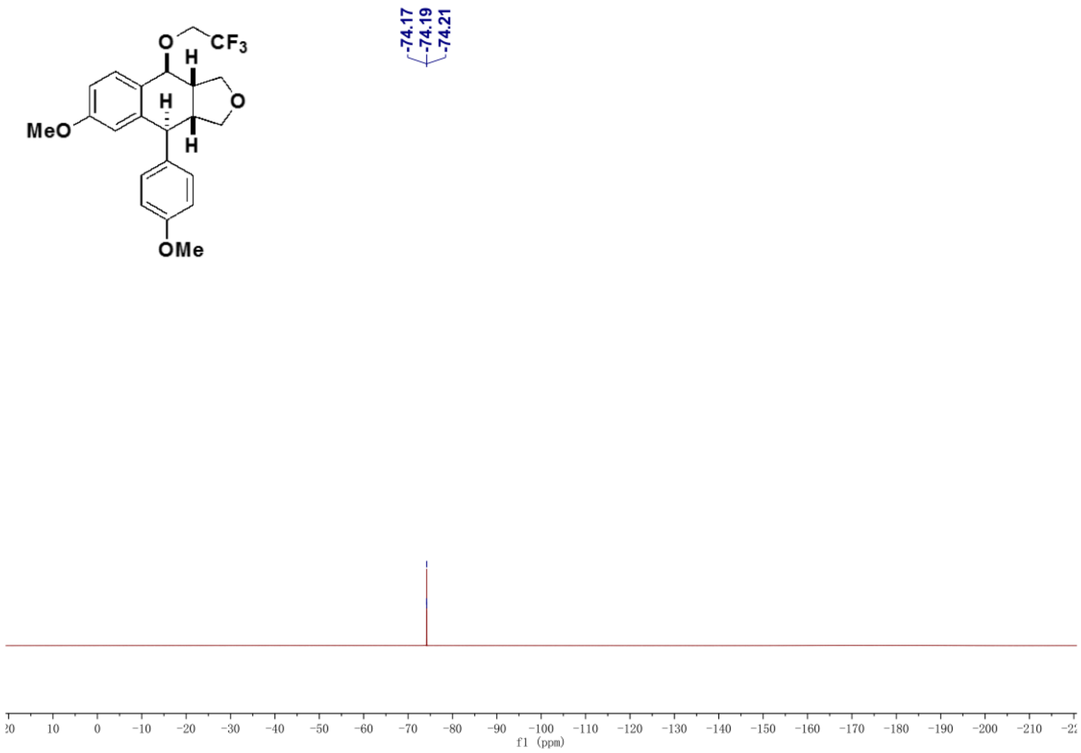

Supplementary Figure 34. ^{1}H NMR spectrum of compound **20e-B** (400 MHz, $CDCl_3$)

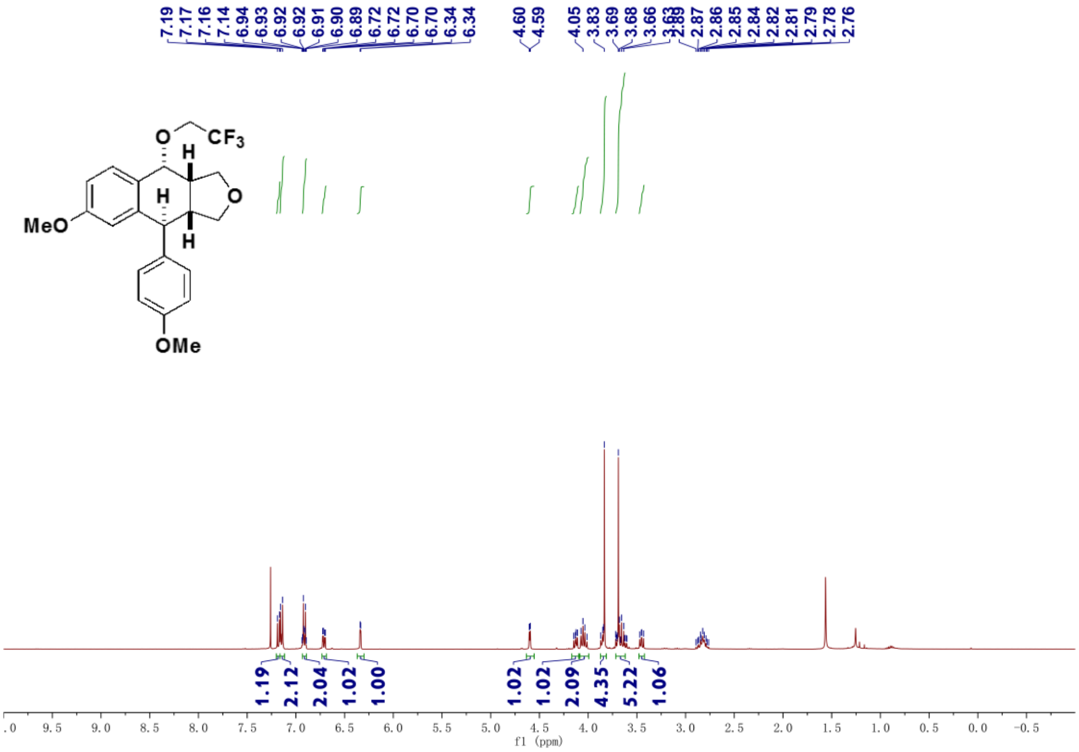

Supplementary Figure 35. ^{13}C NMR spectrum of compound **20e-B** (101 MHz, $CDCl_3$)

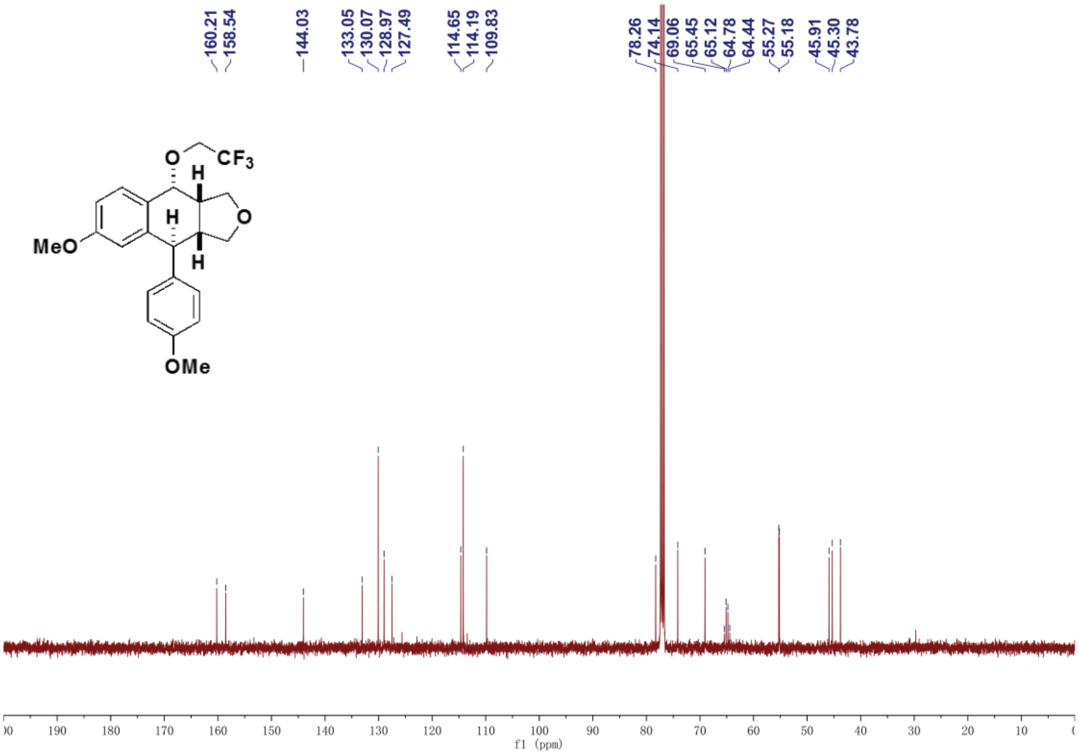

Supplementary Figure 36. ^{19}F NMR spectrum of compound **20e-B** (377 MHz, $CDCl_3$)

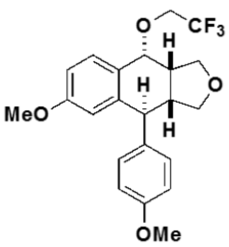

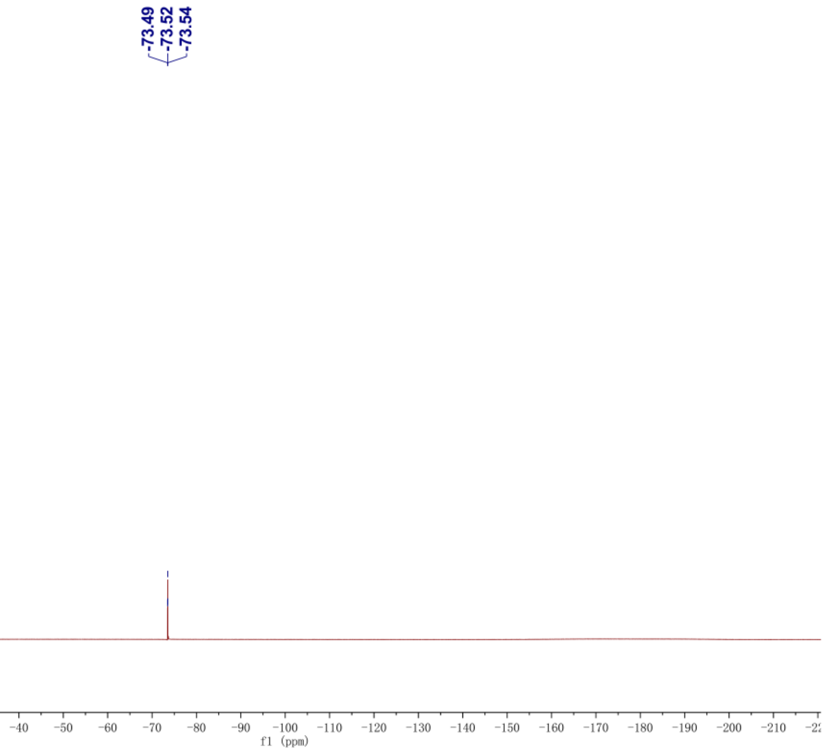

Supplementary Figure 37. ^{1}H NMR spectrum of compound **20f (A+B)** (400 MHz, $CDCl_3$)

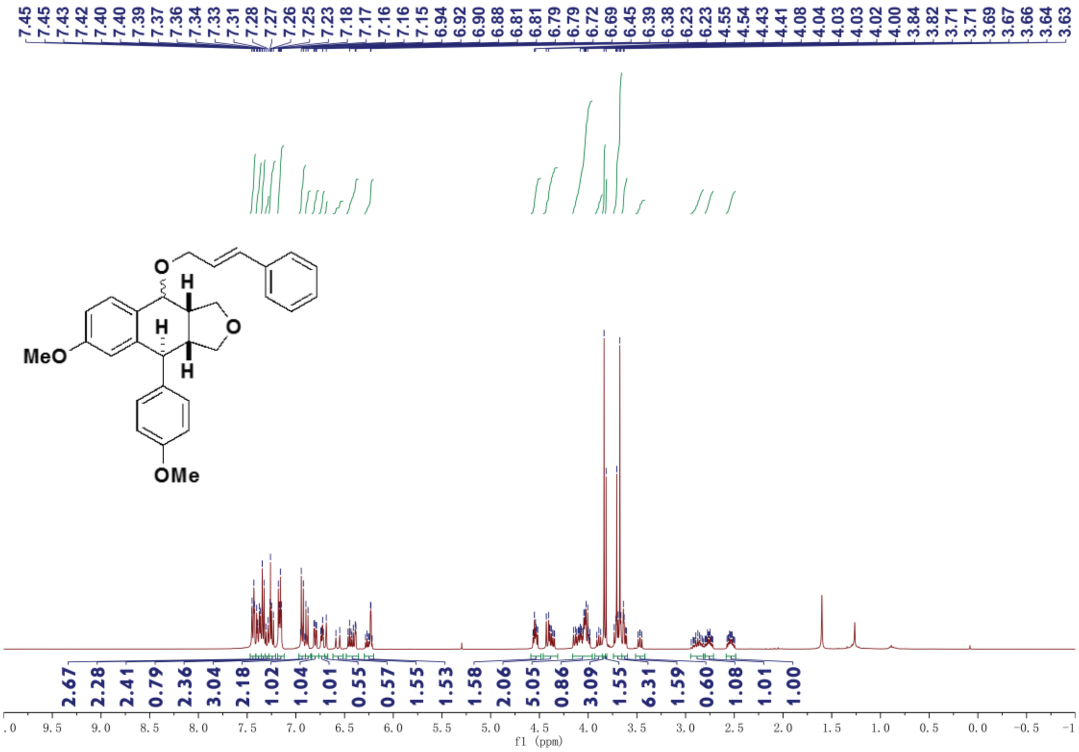

Supplementary Figure 38. ^{13}C NMR spectrum of compound **20f (A+B)** (101 MHz, $CDCl_3$)

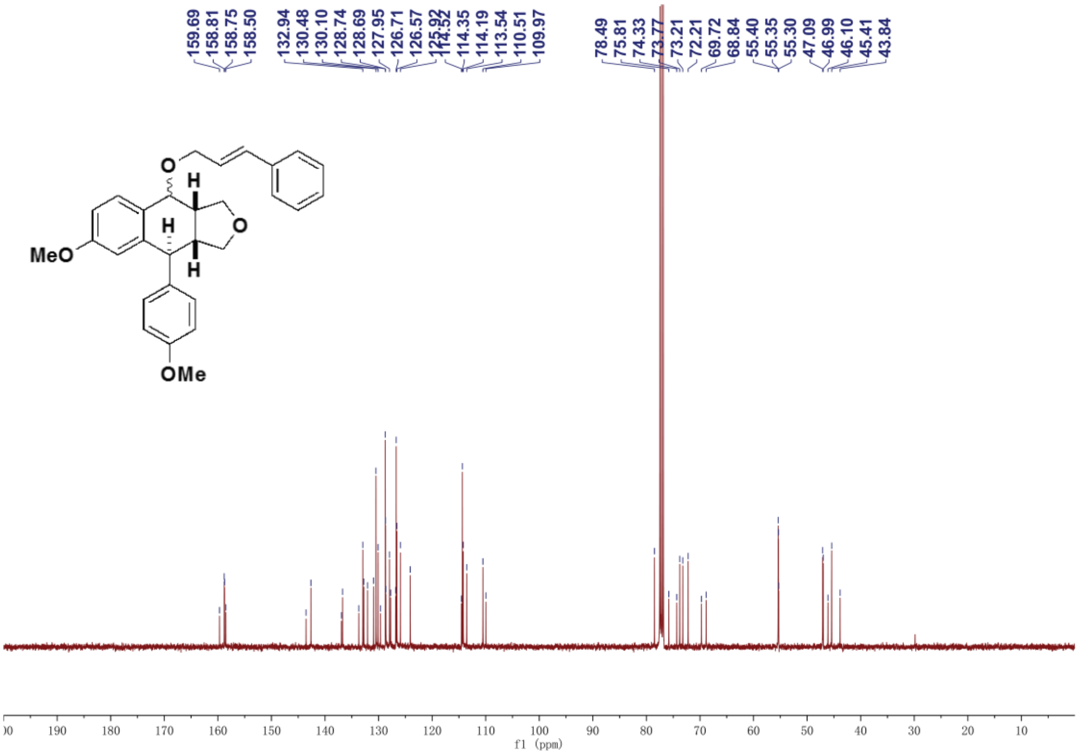

Supplementary Figure 39. ^{1}H NMR spectrum of compound **20g (A+B)** (400 MHz, $CDCl_3$)

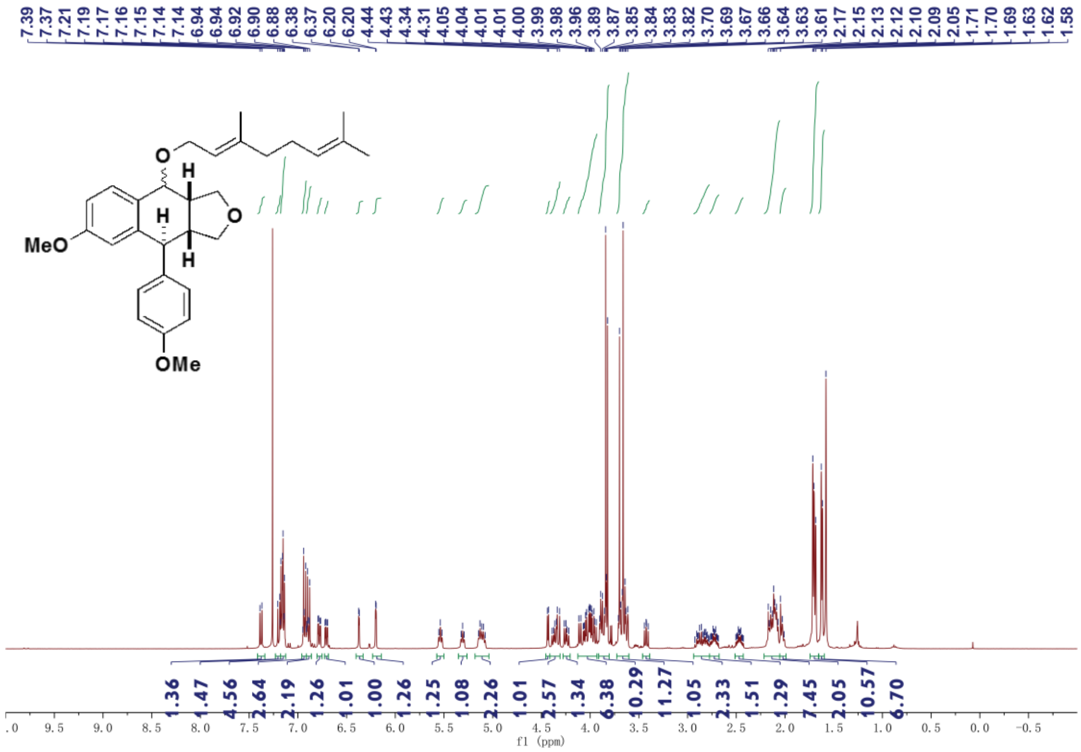

Supplementary Figure 40. ^{13}C NMR spectrum of compound **20g (A+B)** (101 MHz, $CDCl_3$)

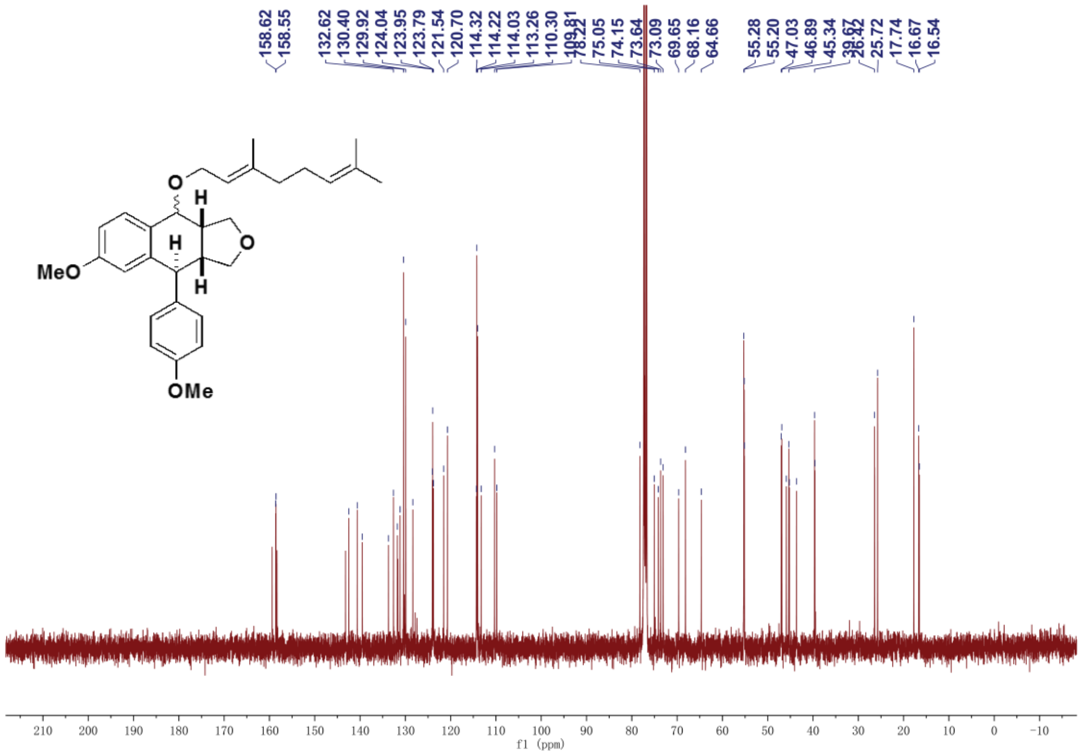

Supplementary Figure 41. ^{1}H NMR spectrum of compound **20h (A+B)** (400 MHz, $CDCl_3$)

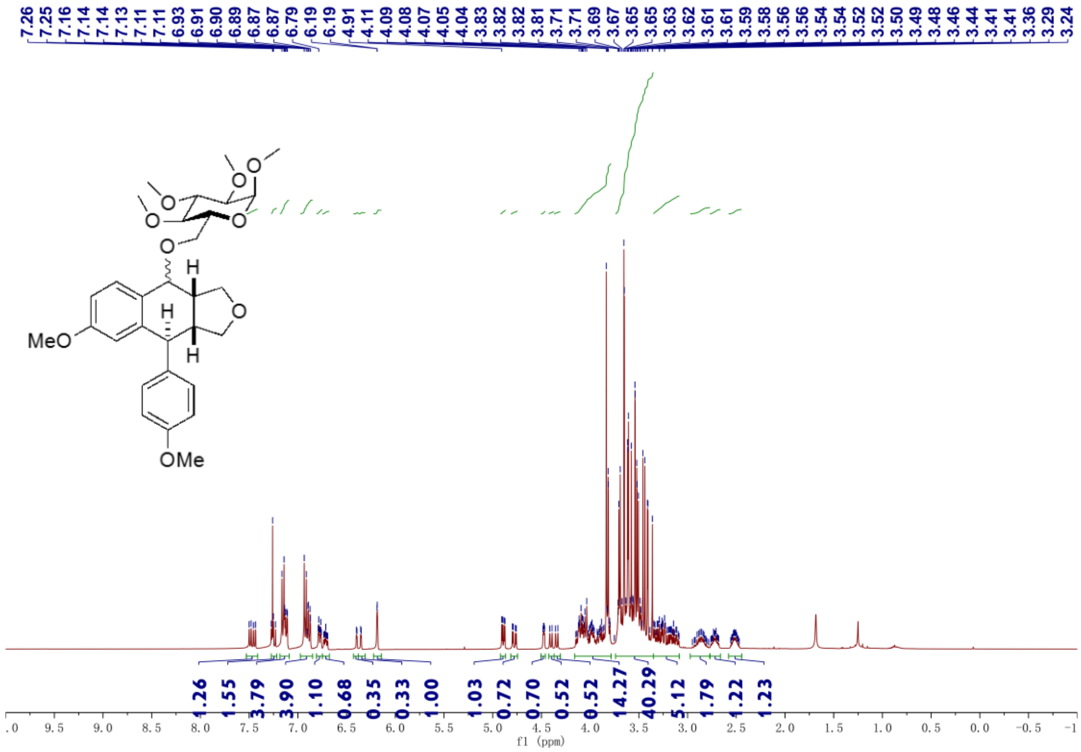

Supplementary Figure 42. ^{13}C NMR spectrum of compound **20h (A+B)** (101 MHz, $CDCl_3$)

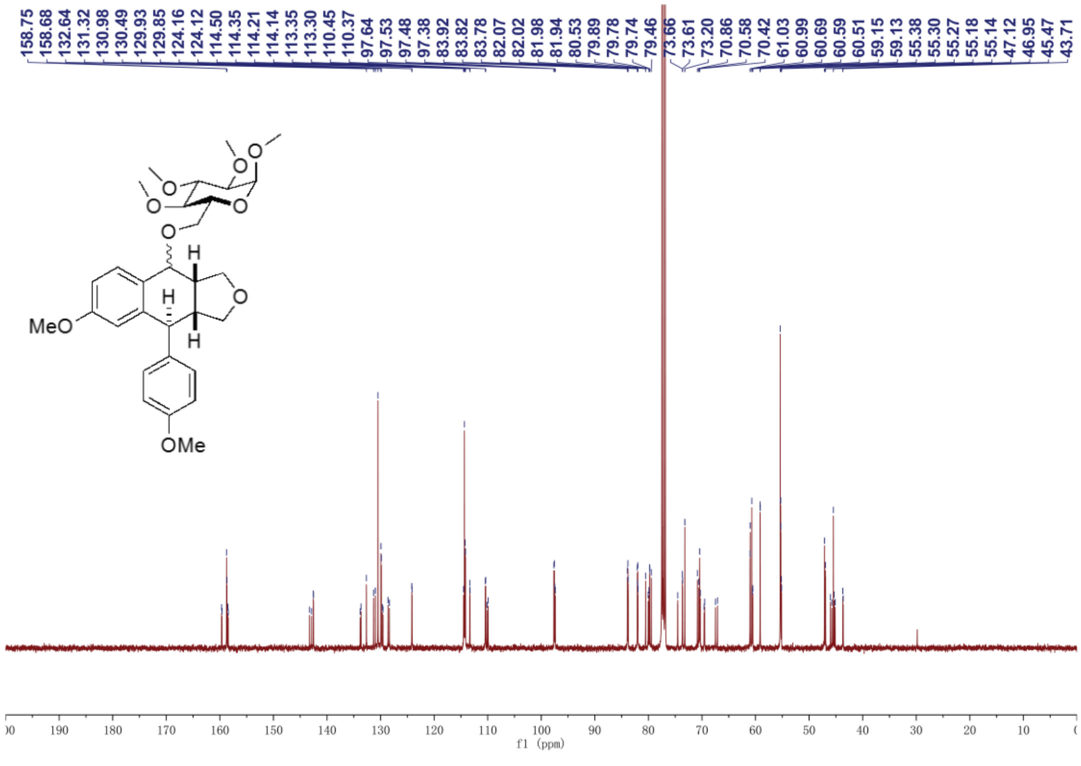

Supplementary Figure 43. 2D NMR spectra of compound **20h (A+B)** ($CDCl_3$)

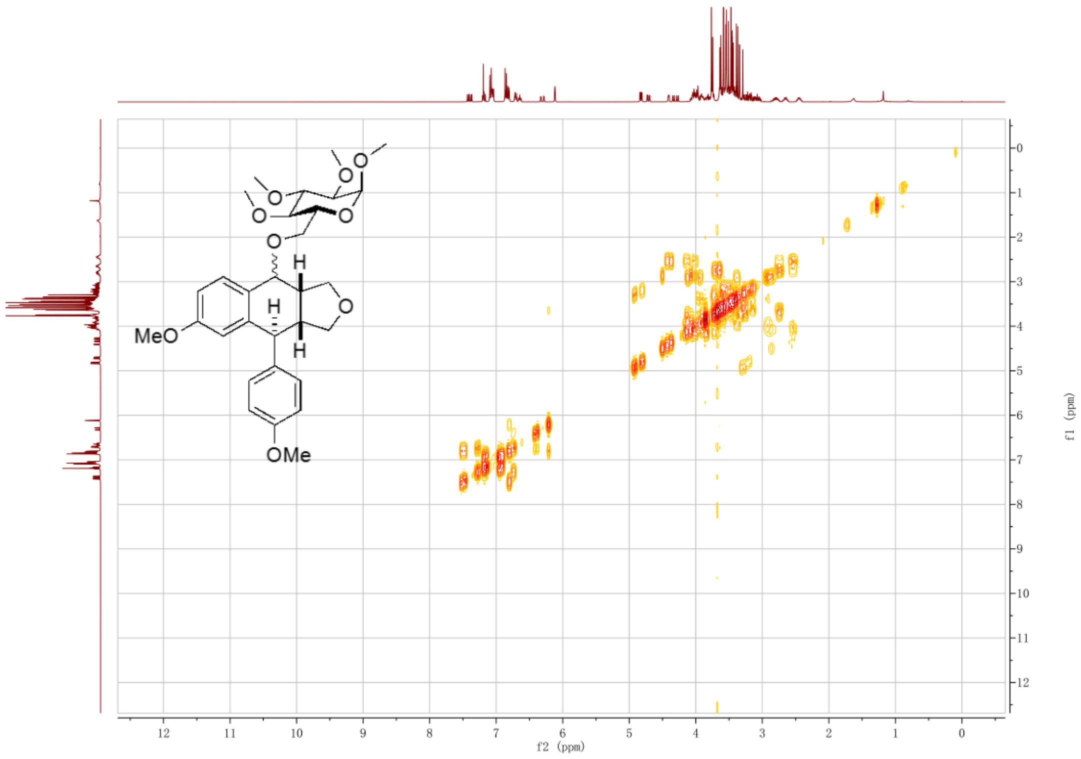

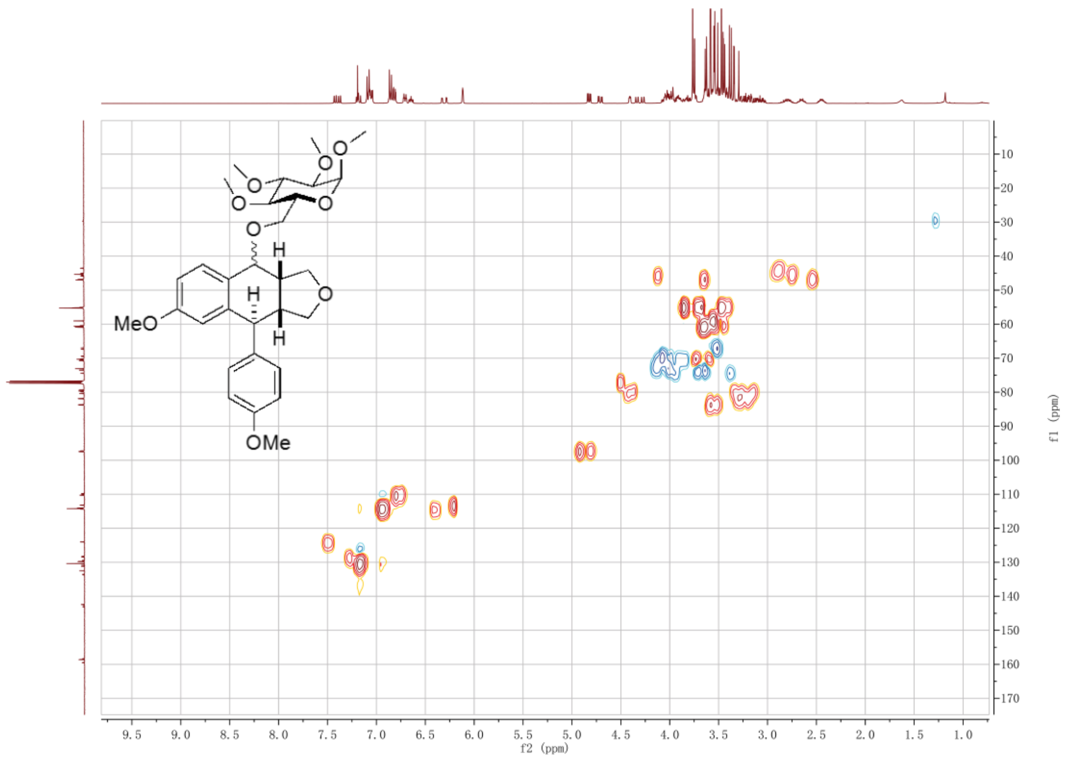

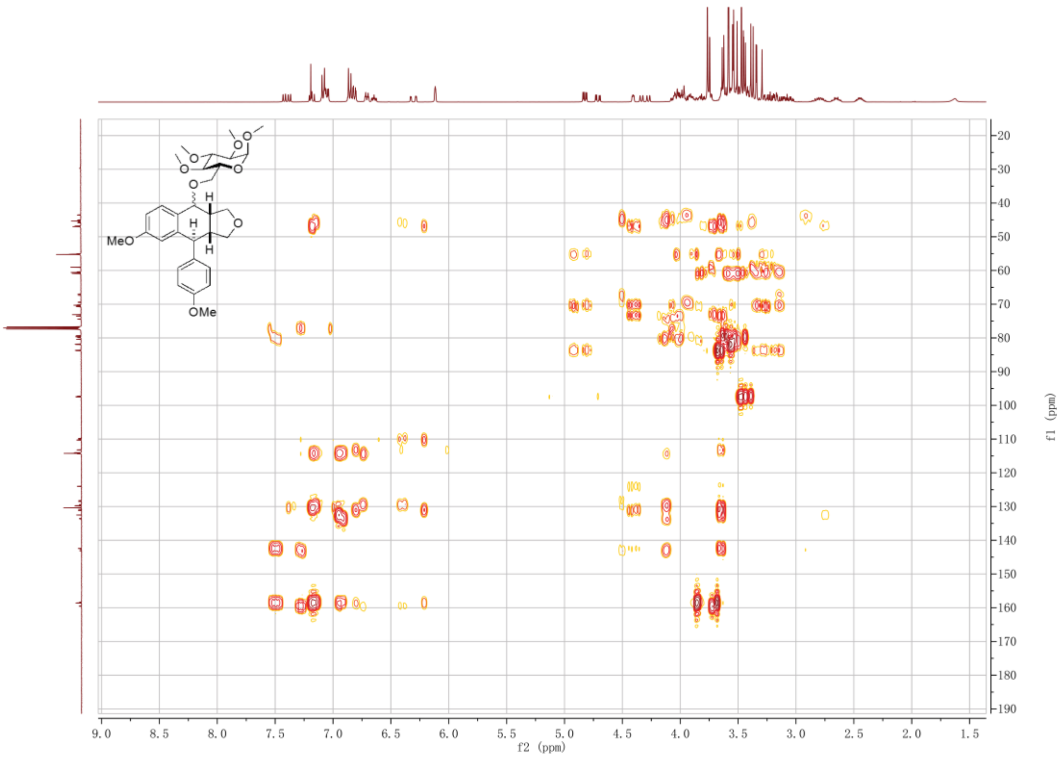
MeO
OMe
H
H
H
f2 (ppm)
f1 (ppm)

Supplementary Figure 44. ^{1}H NMR spectrum of compound **20i (two of four isomers)** (400 MHz, $CDCl_3$)

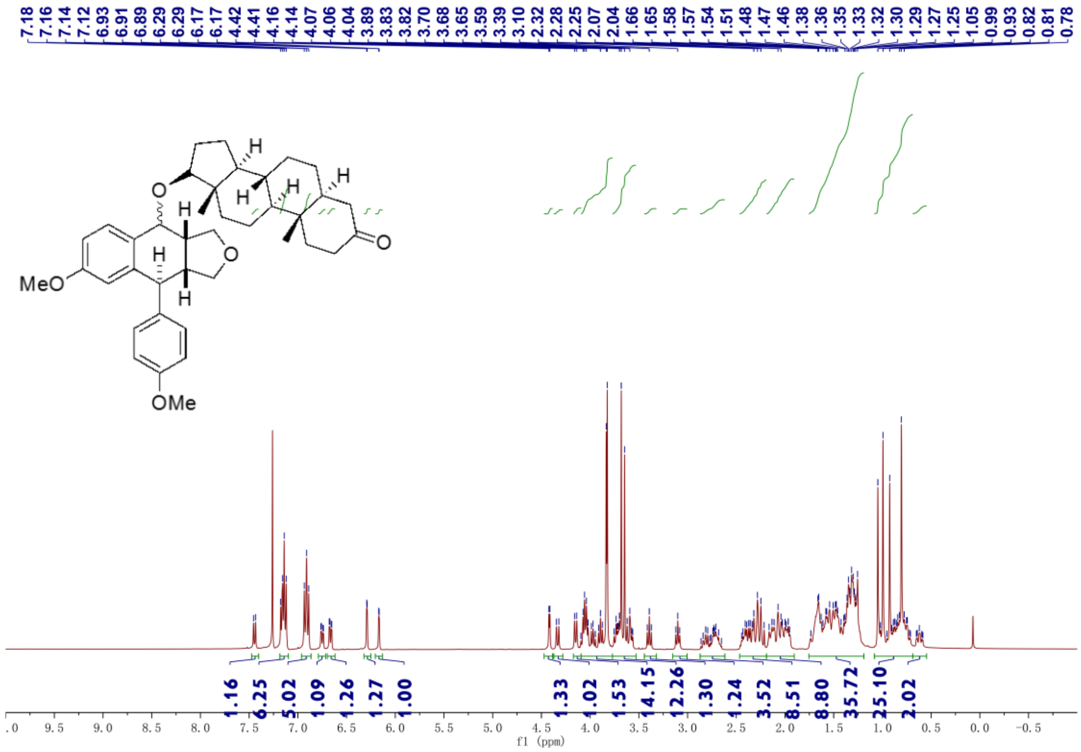

Supplementary Figure 45. ^{13}C NMR spectrum of compound **20i (two of four isomers)** (101 MHz, $CDCl_3$)

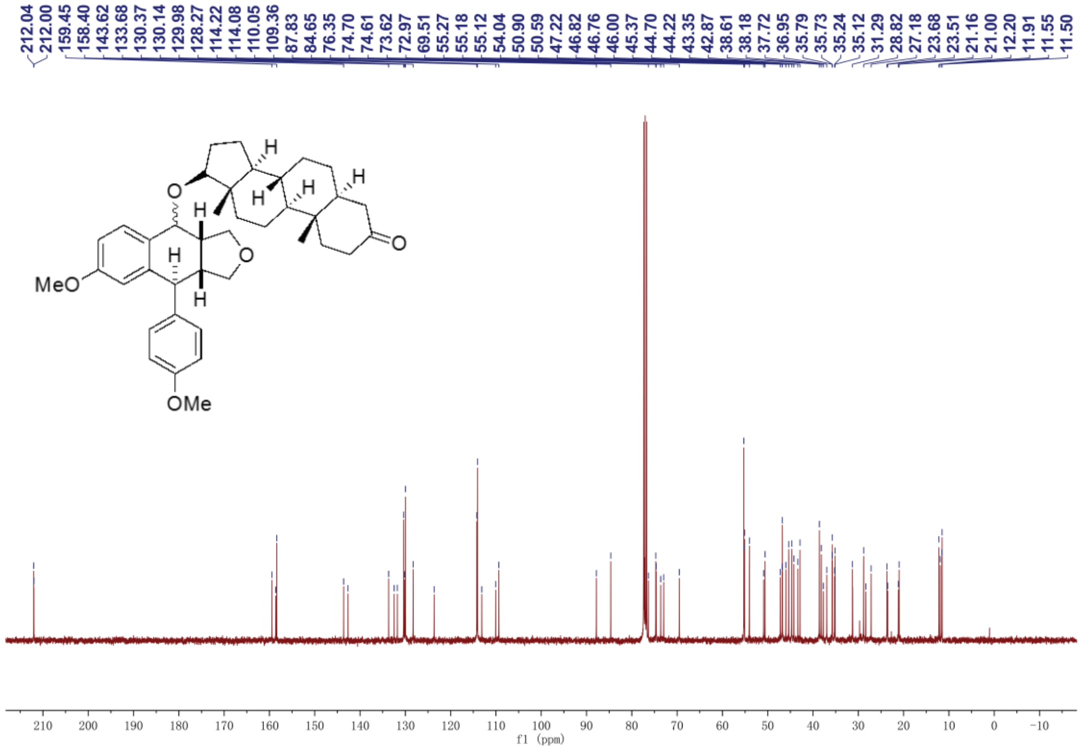

Supplementary Figure 46. 2D NMR spectra of compound **20i (two of four isomers)** ($CDCl_3$)

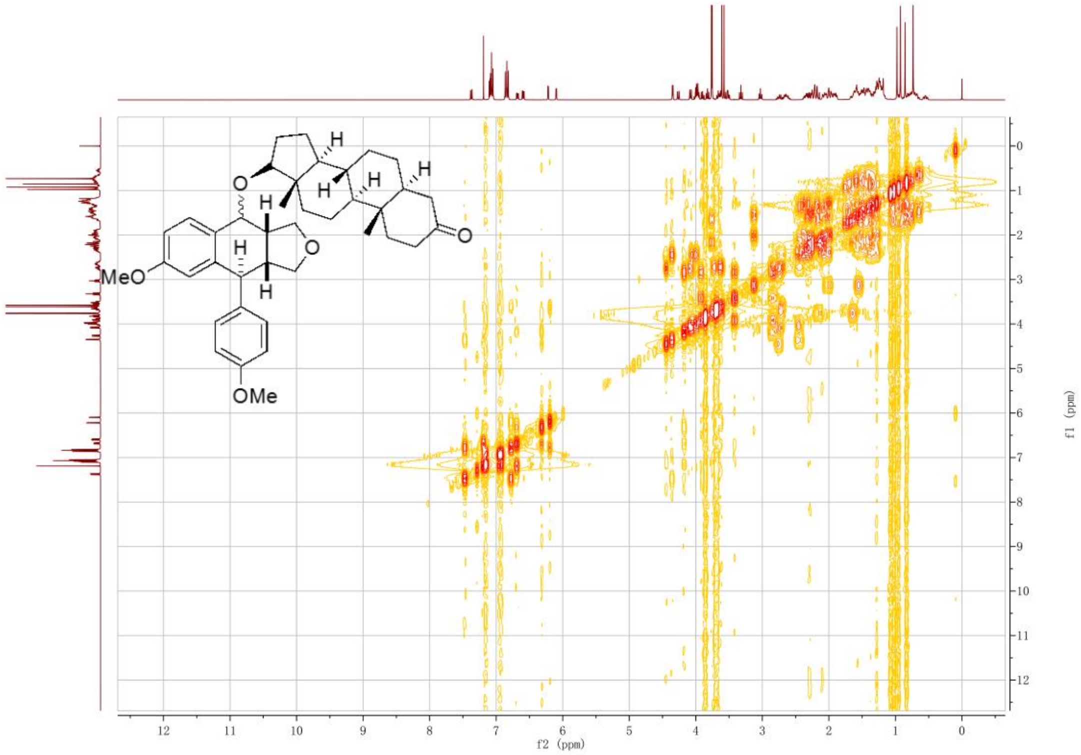

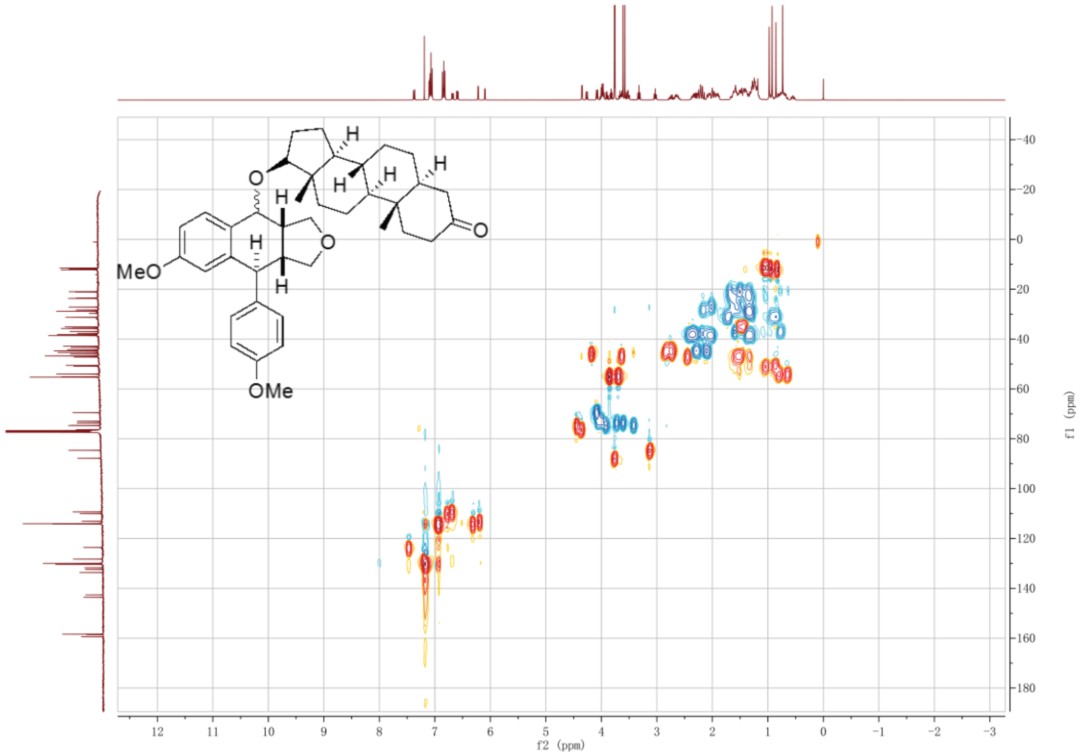

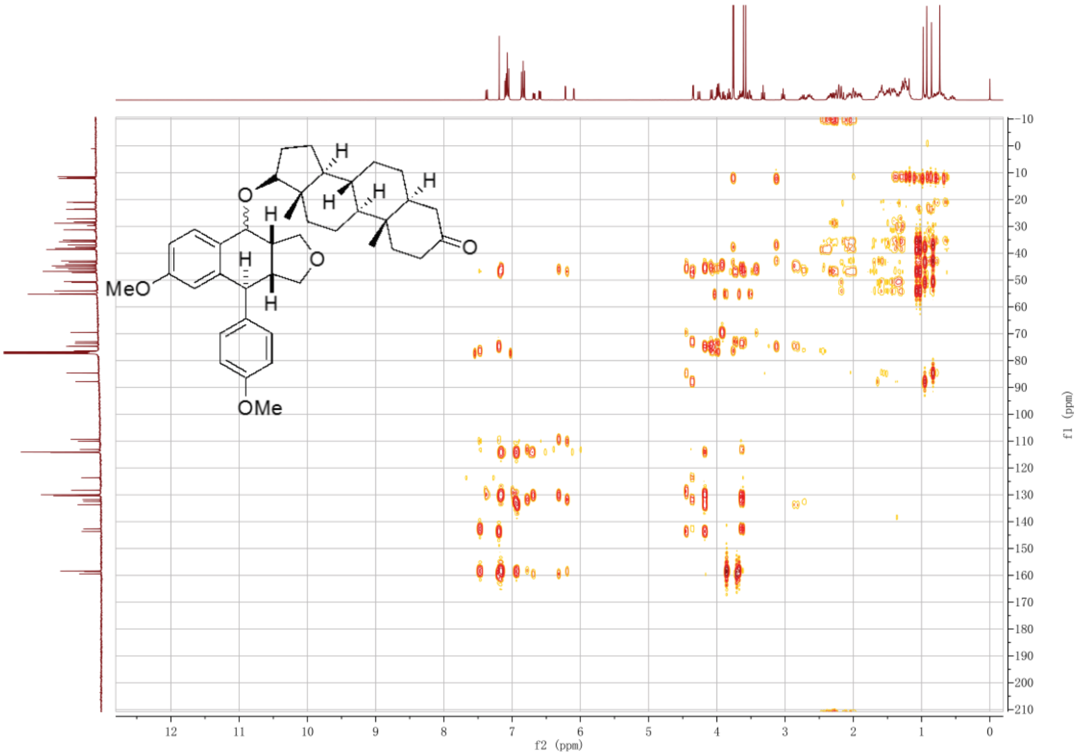

MeO
OMe
H
O
f2 (ppm)
f1 (ppm)

Supplementary Figure 47. ^{1}H NMR spectrum of compound **20i (two of four isomers)** (400 MHz, $CDCl_3$)

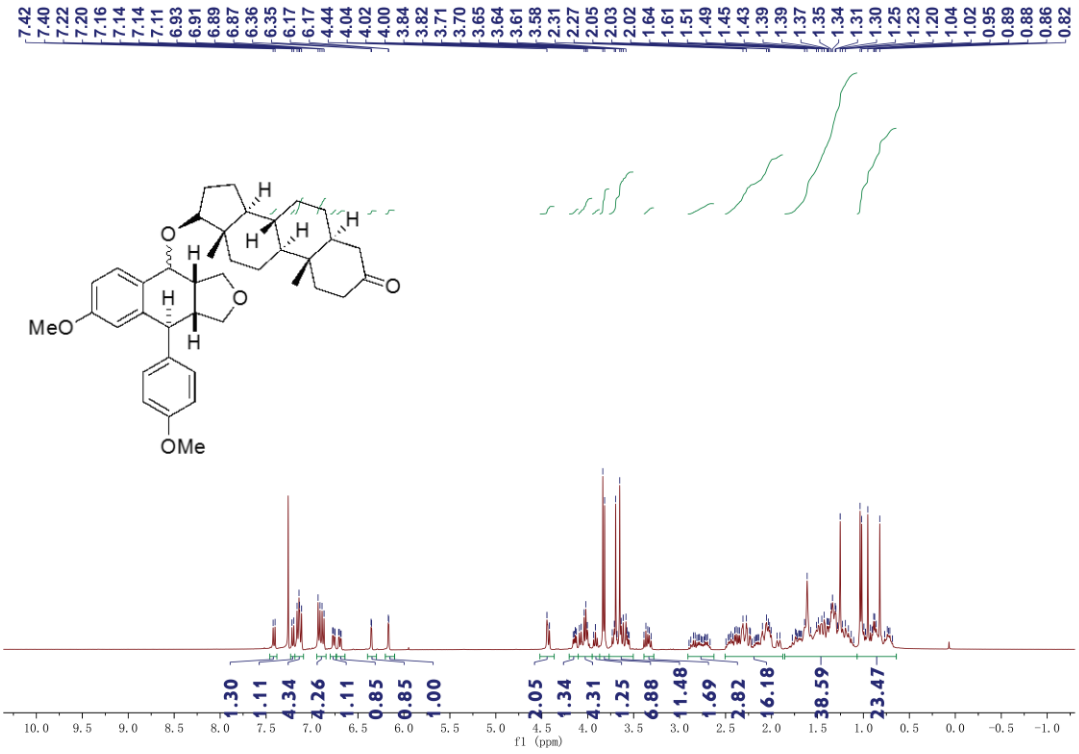

Supplementary Figure 48. ^{13}C NMR spectrum of compound **20i (two of four isomers)** (101 MHz, $CDCl_3$)

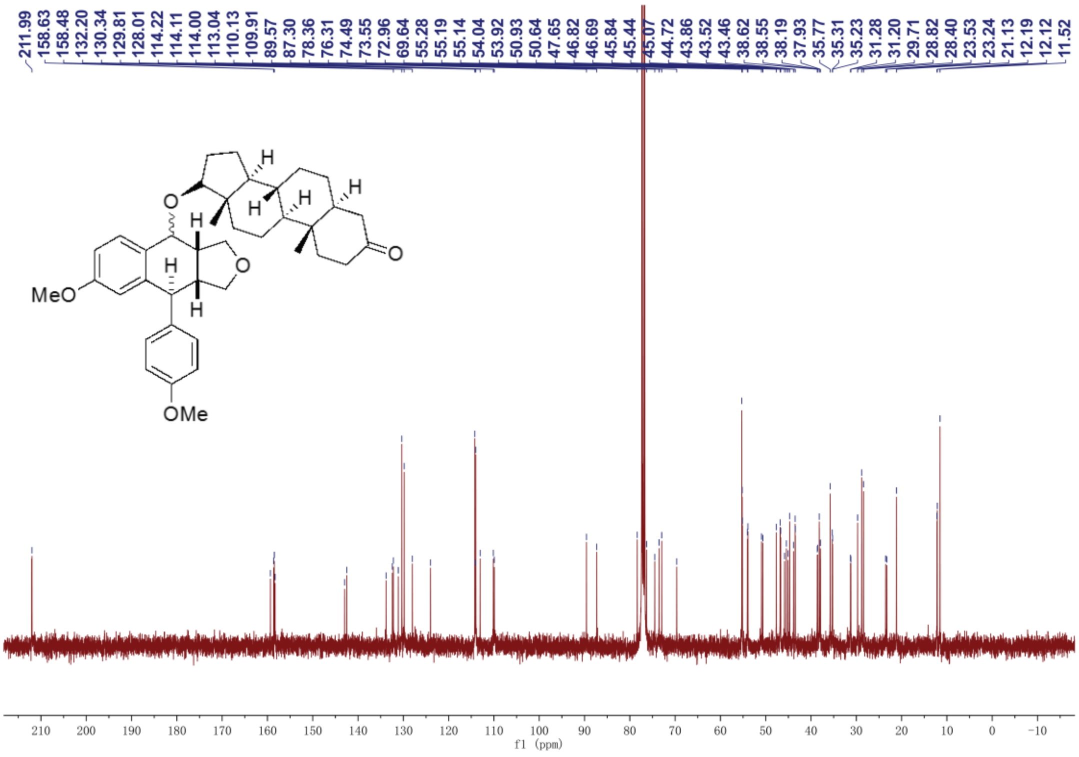

Supplementary Figure 49. ^{1}H NMR spectrum of compound **20j-A** (400 MHz, $CDCl_3$)

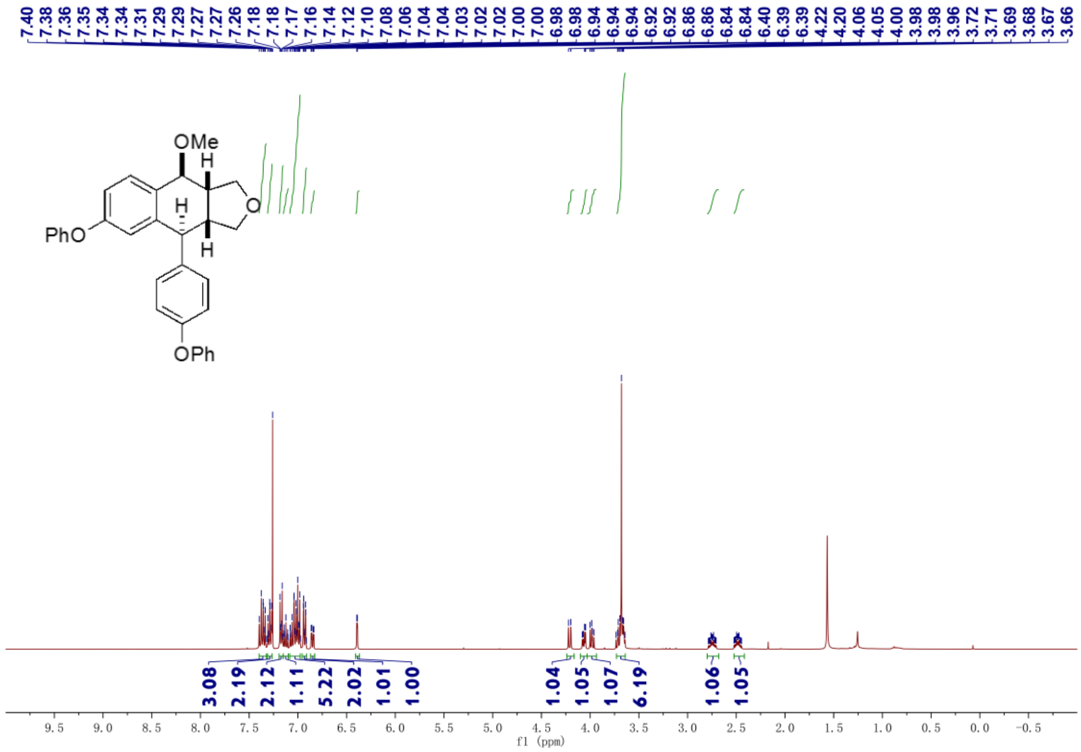

Supplementary Figure 50. ^{13}C NMR spectrum of compound **20j-A** (101 MHz, $CDCl_3$)

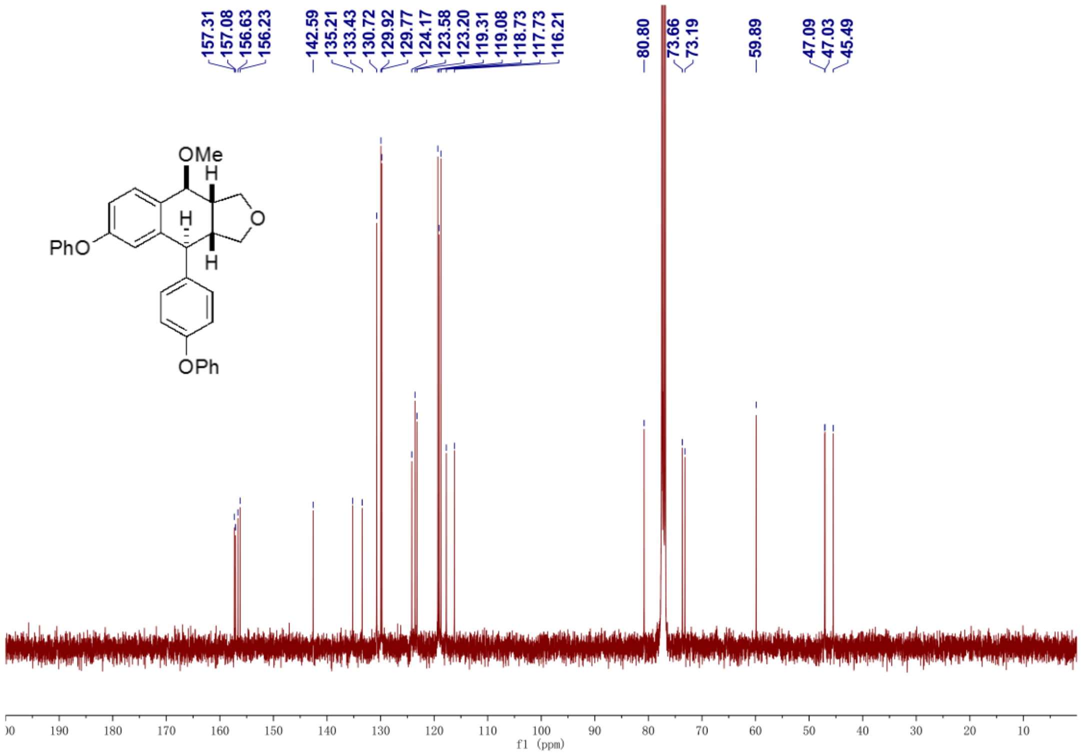

Supplementary Figure 51. ^{1}H NMR spectrum of compound **20j-B** (400 MHz, $CDCl_3$)

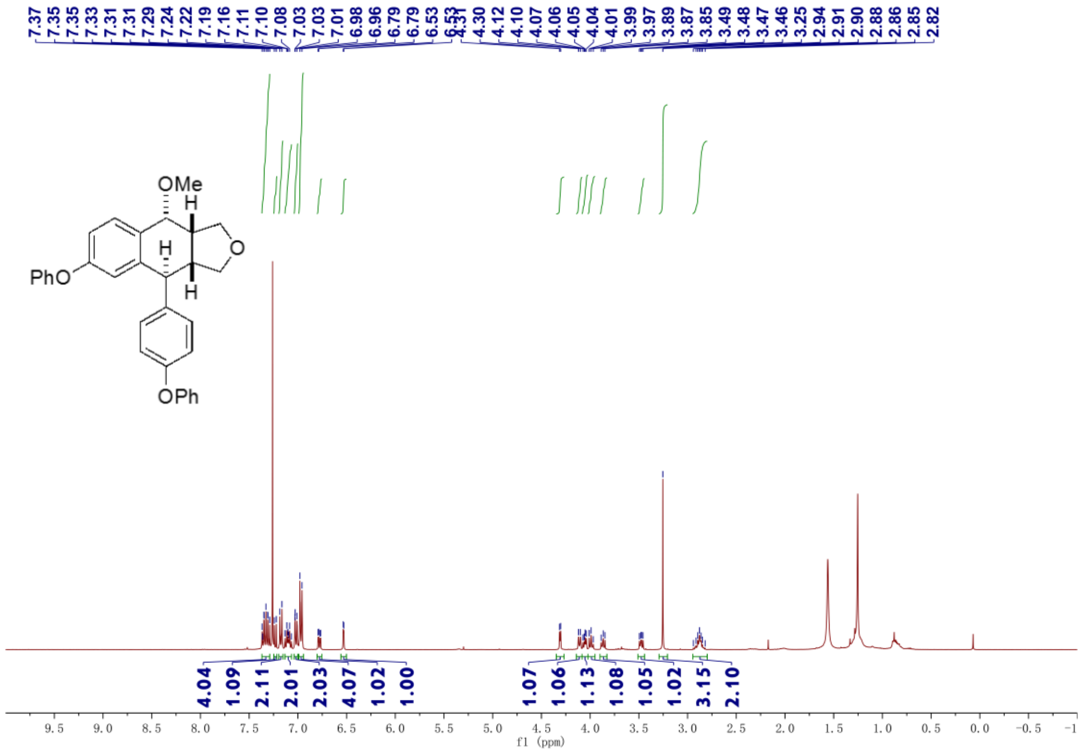

Supplementary Figure 52. ^{13}C NMR spectrum of compound **20j-B** (101 MHz, $CDCl_3$)

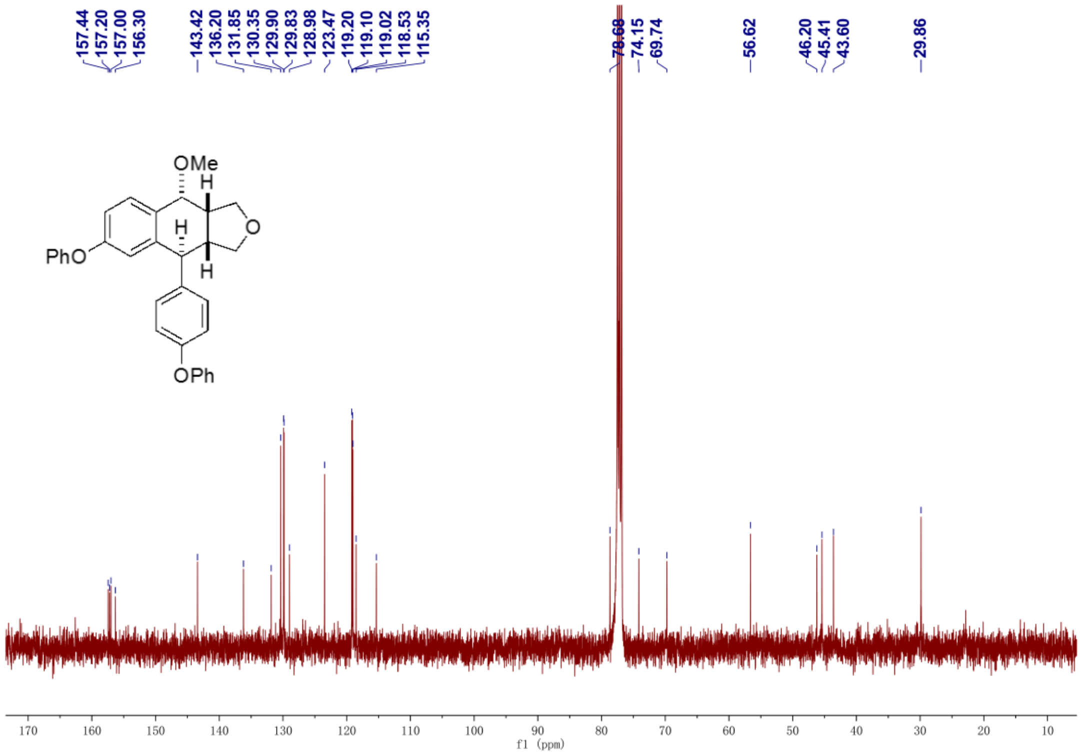

Supplementary Figure 53. ^{1}H NMR spectrum of compound **20k-A** (400 MHz, $CDCl_3$)

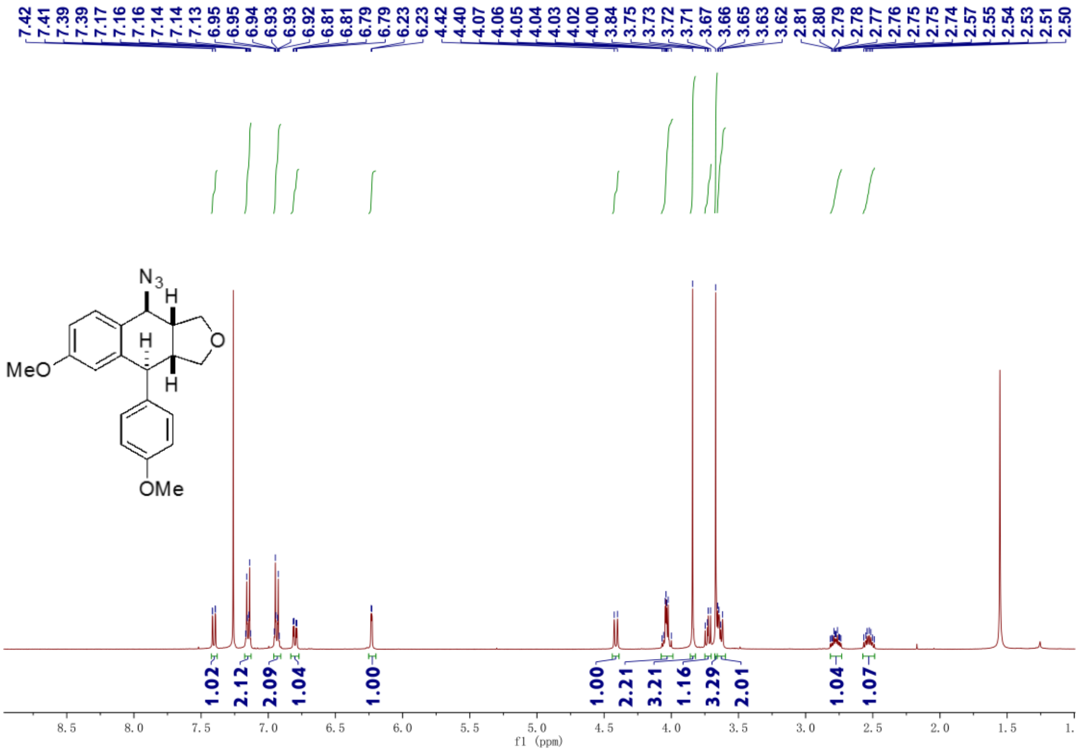

Supplementary Figure 54. ^{13}C NMR spectrum of compound **20k-A** (101 MHz, $CDCl_3$)

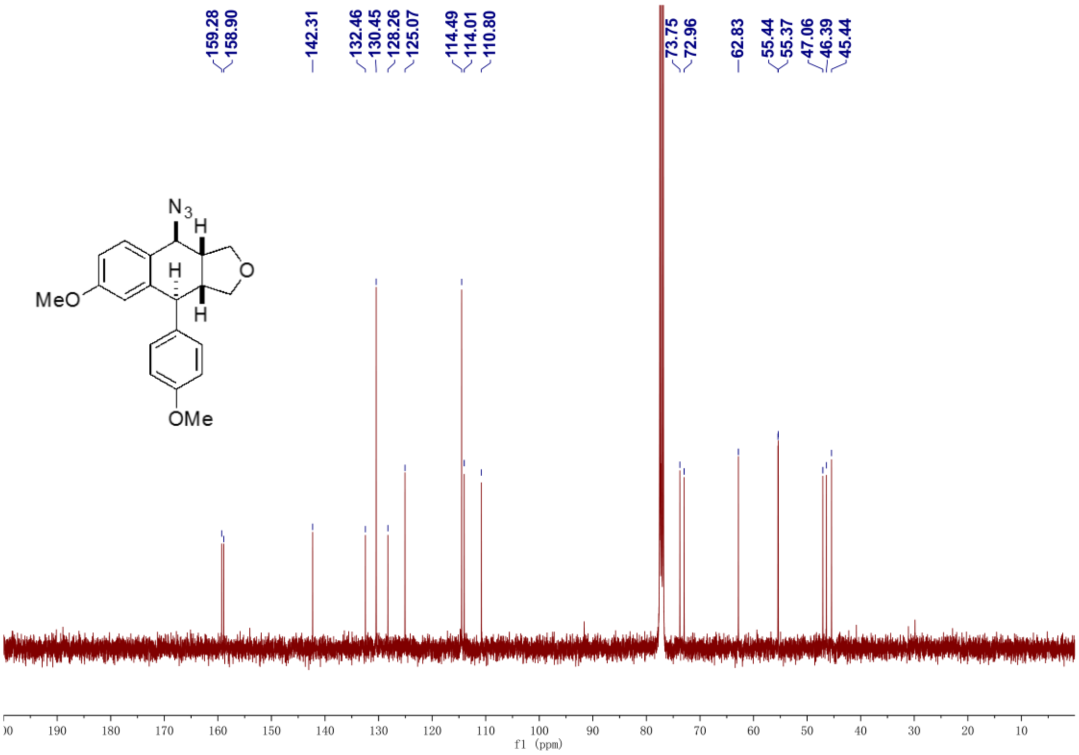

Supplementary Figure 55. ^{1}H NMR spectrum of compound **20k-B** (400 MHz, $CDCl_3$)

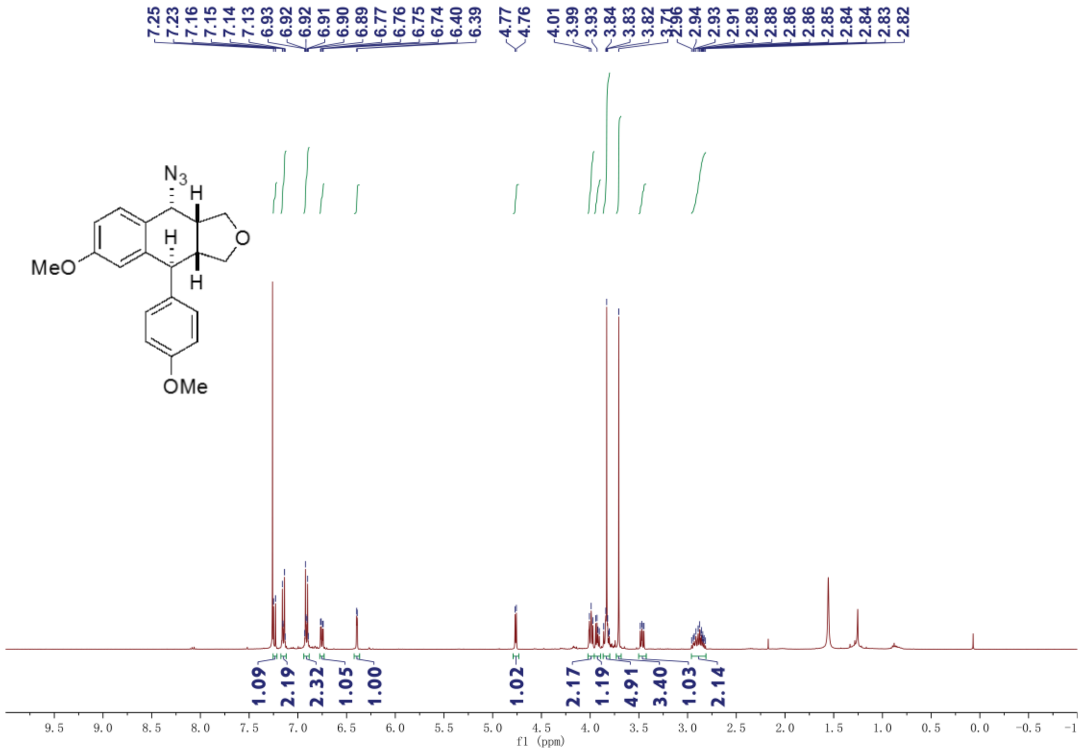

Supplementary Figure 56. ^{13}C NMR spectrum of compound **20k-B** (101 MHz, $CDCl_3$)

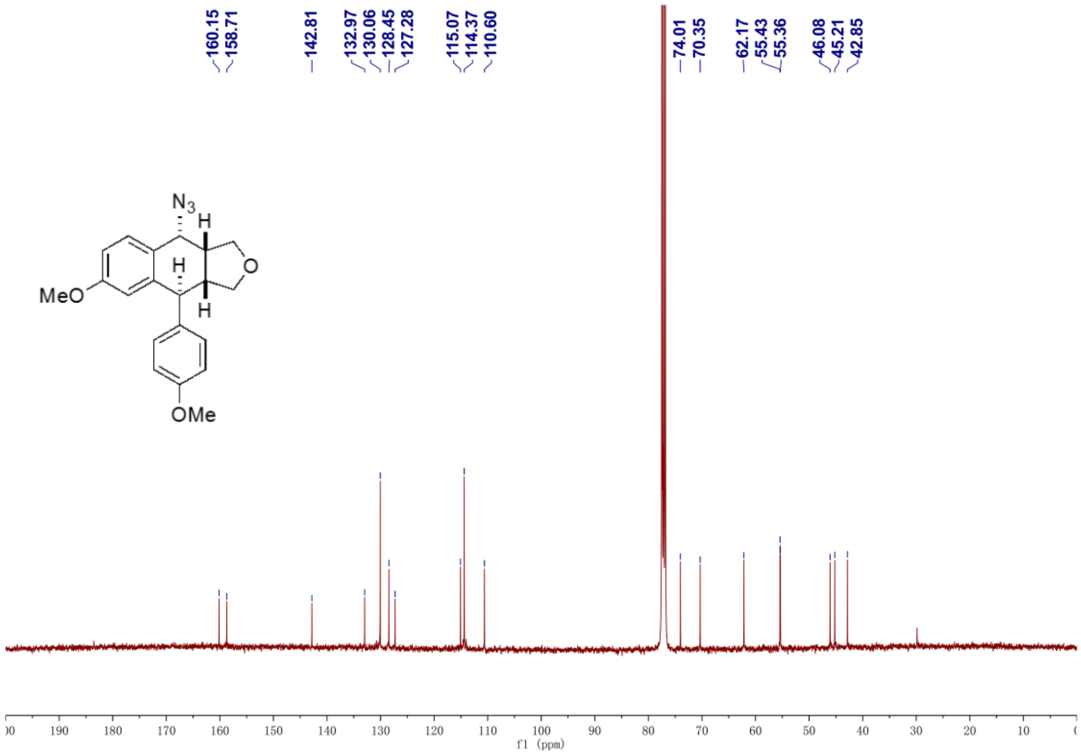

Supplementary Figure 57. ^{1}H NMR spectrum of compound **21a-A** (400 MHz, $CDCl_3$)

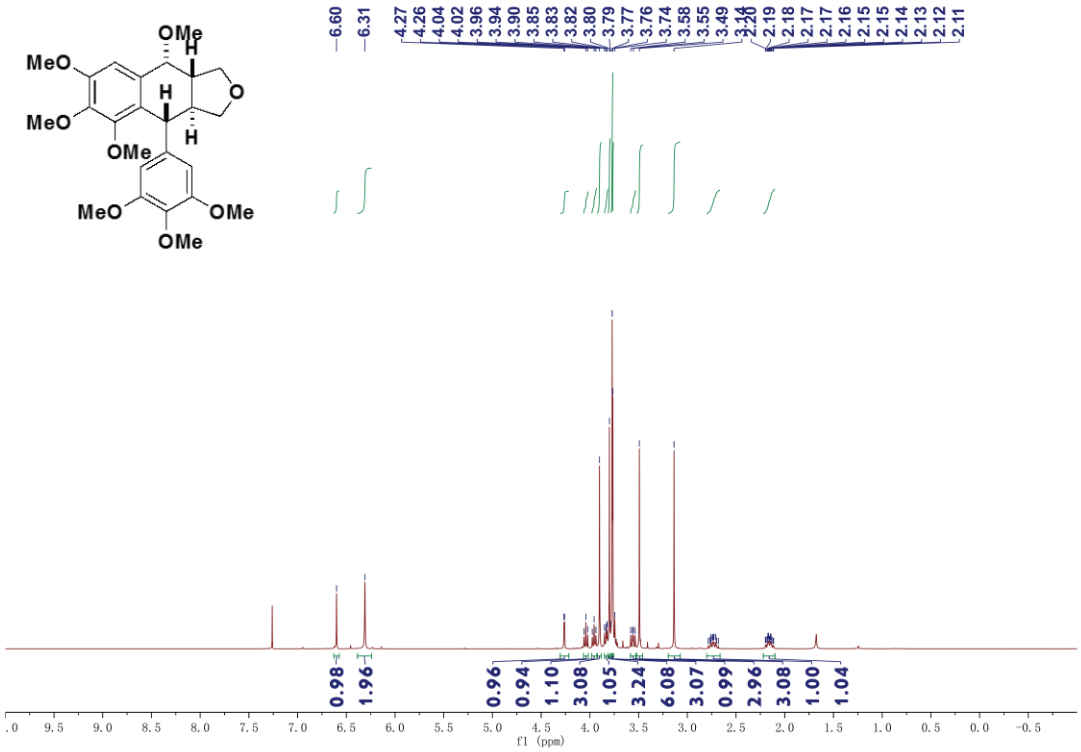

Supplementary Figure 58. ^{13}C NMR spectrum of compound **21a-A** (101 MHz, $CDCl_3$)

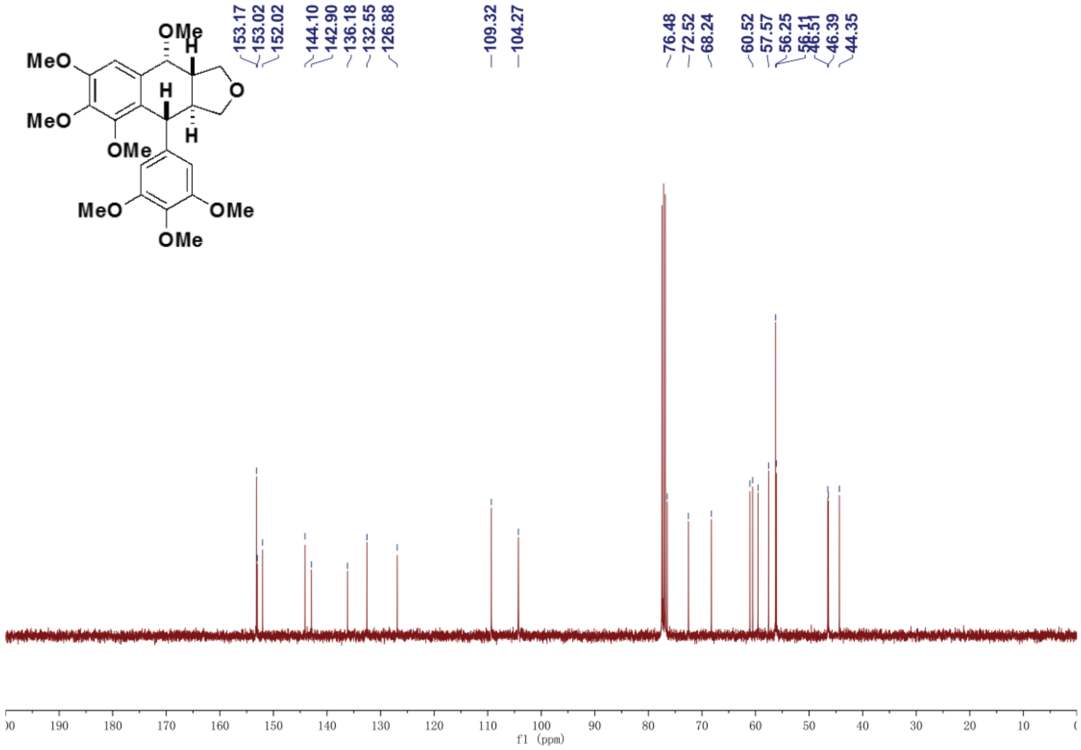

Supplementary Figure 59. ^{1}H NMR spectrum of compound **4 (Aglacin E)** (400 MHz, $CDCl_3$)

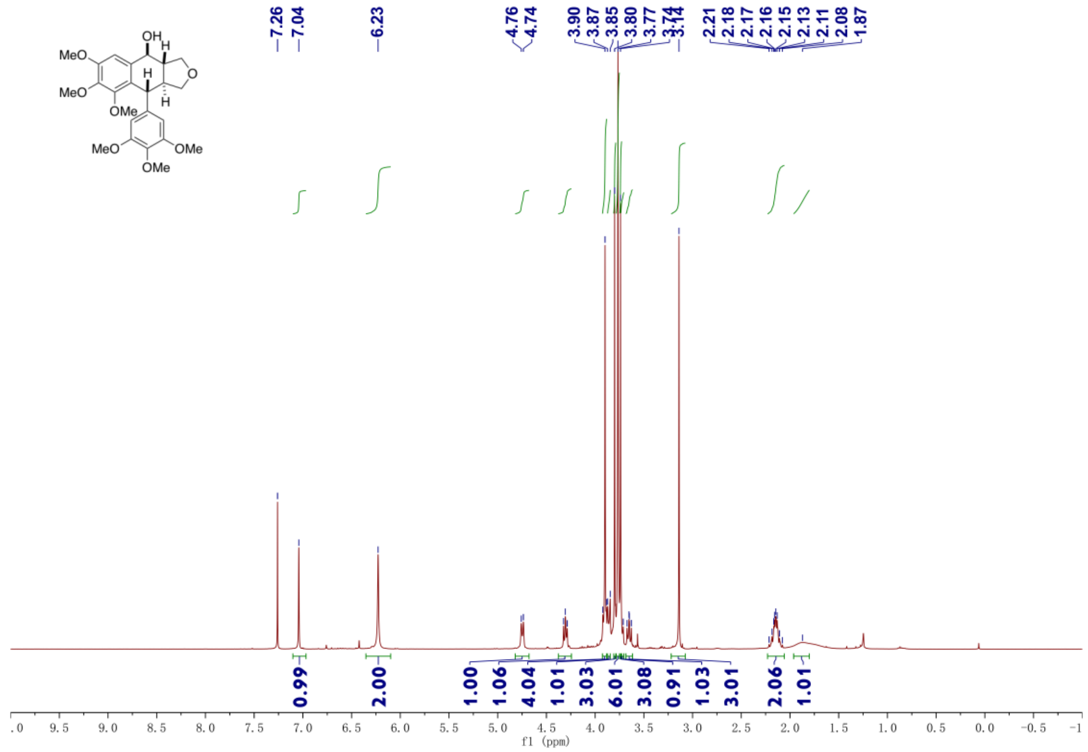

Supplementary Figure 60. ^{13}C NMR spectrum of compound **4 (Aglacin E)** (101 MHz, $CDCl_3$)

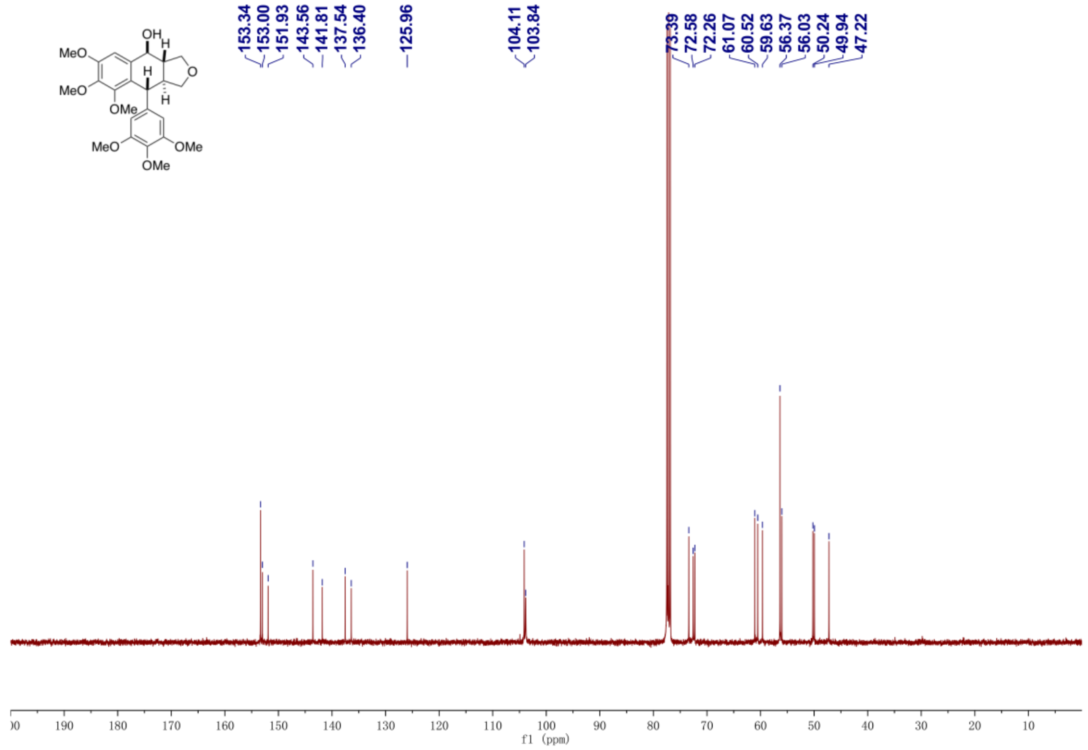

Supplementary Figure 61. 2D NMR spectra of compound **4 (Aglacin E)** ($CDCl_3$)

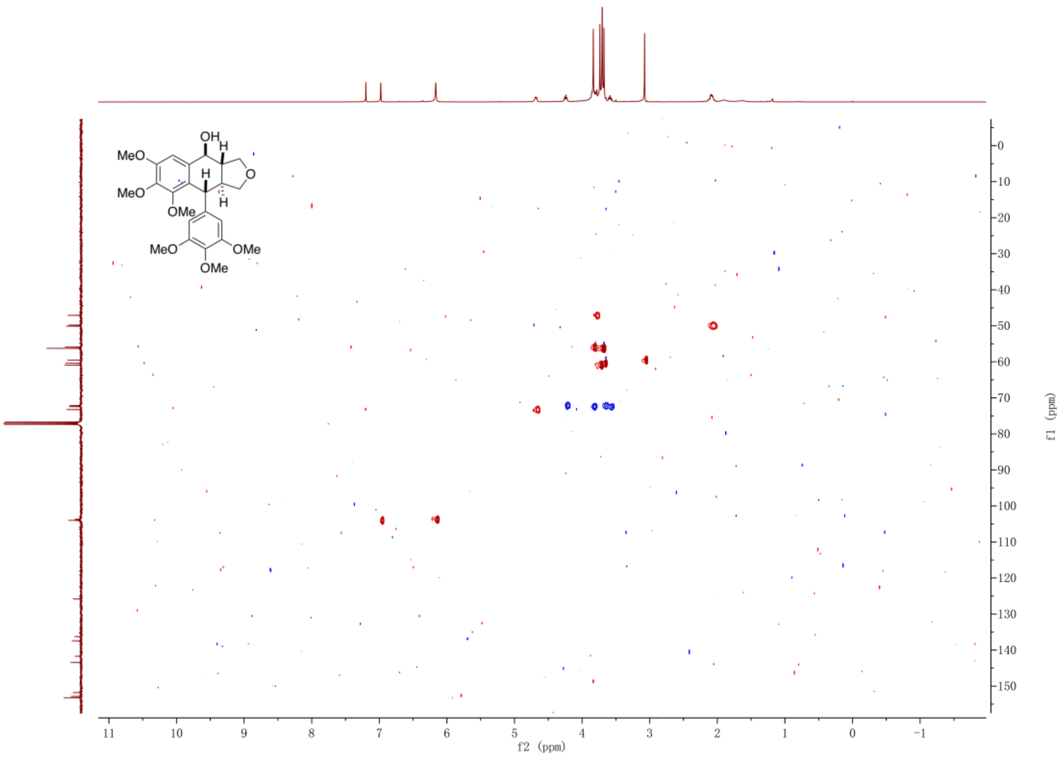

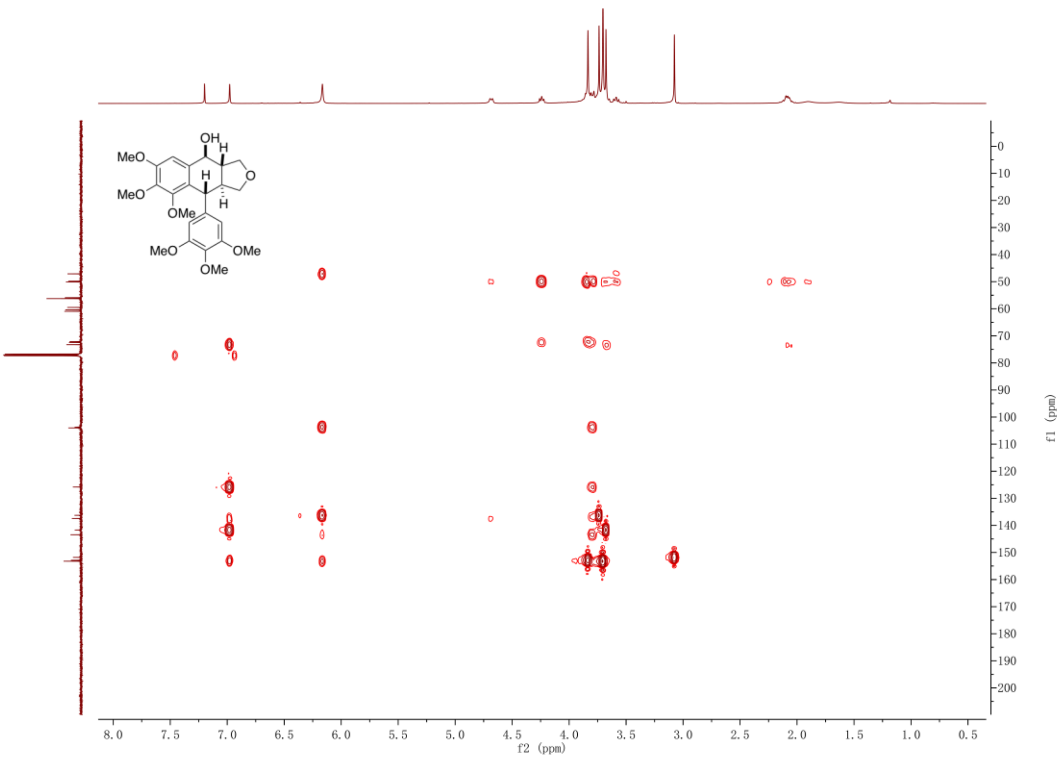

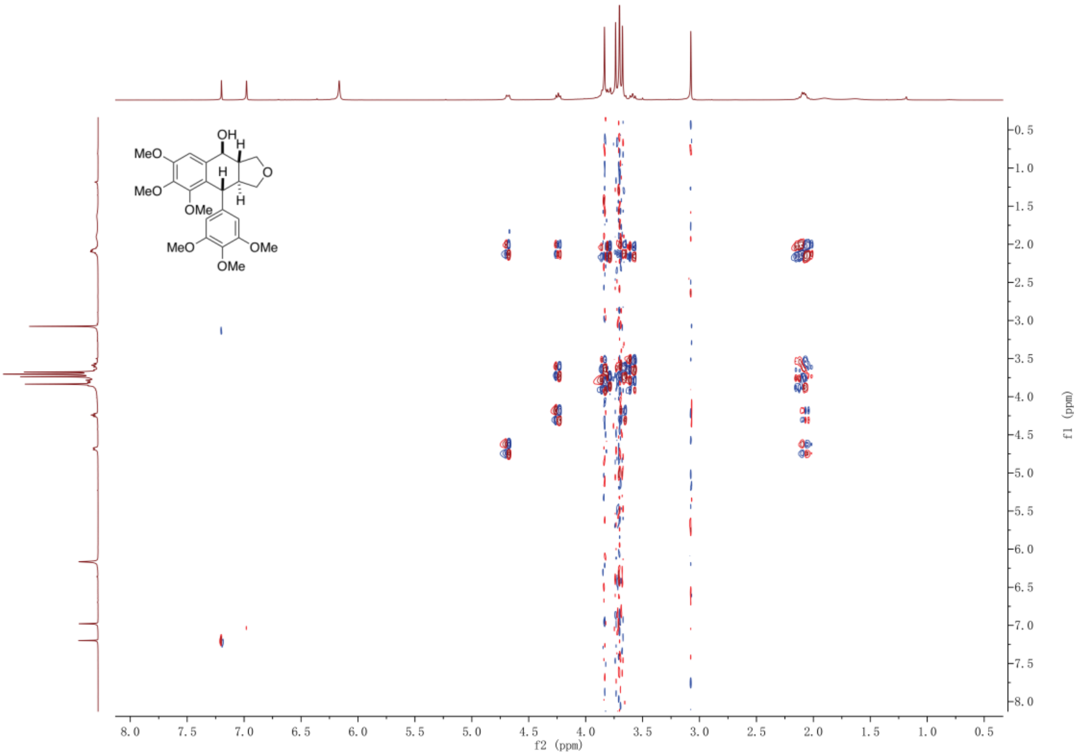
OH
H
MeO
MeO
H
O
H
OMe
MeO
OMe
OMe
8.0
7.5
7.0
6.5
6.0
5.5
5.0
4.5
4.0
3.5
3.0
2.5
2.0
1.5
1.0
0.5
f2 (ppm)
f1 (ppm)

Supplementary Figure 62. ^{1}H NMR spectrum of compound **5 (Aglacin F)** (400 MHz, $CDCl_3$)

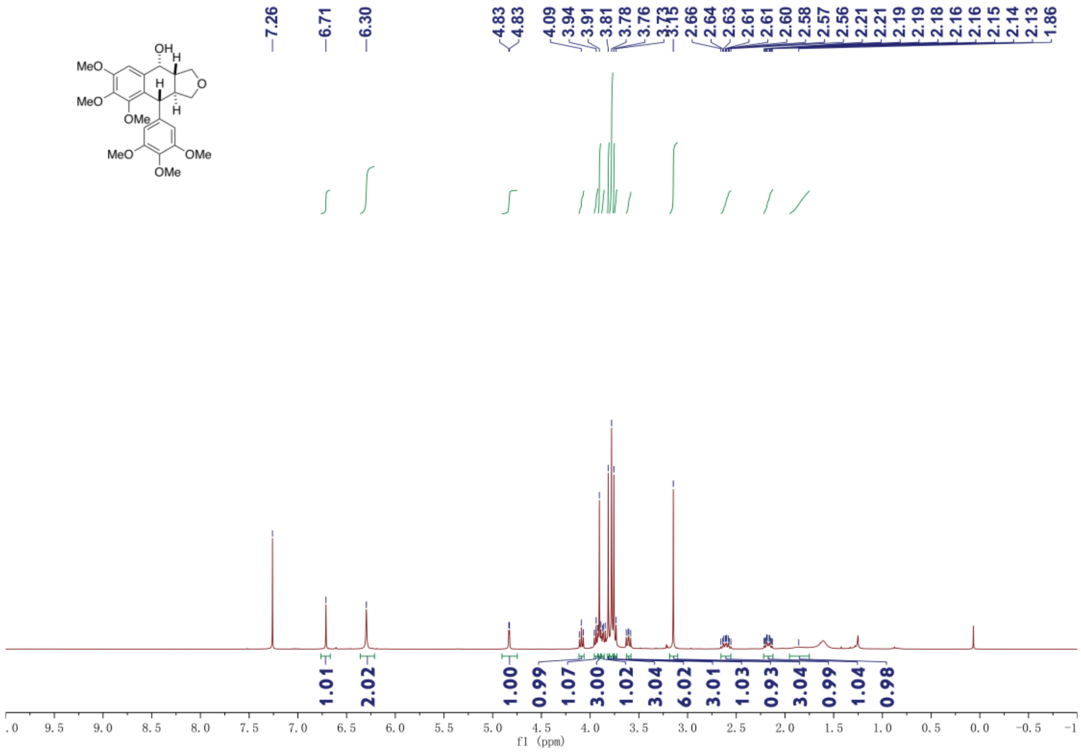

Supplementary Figure 63. ^{13}C NMR spectrum of compound **5 (Aglacin F)** (101 MHz, $CDCl_3$)

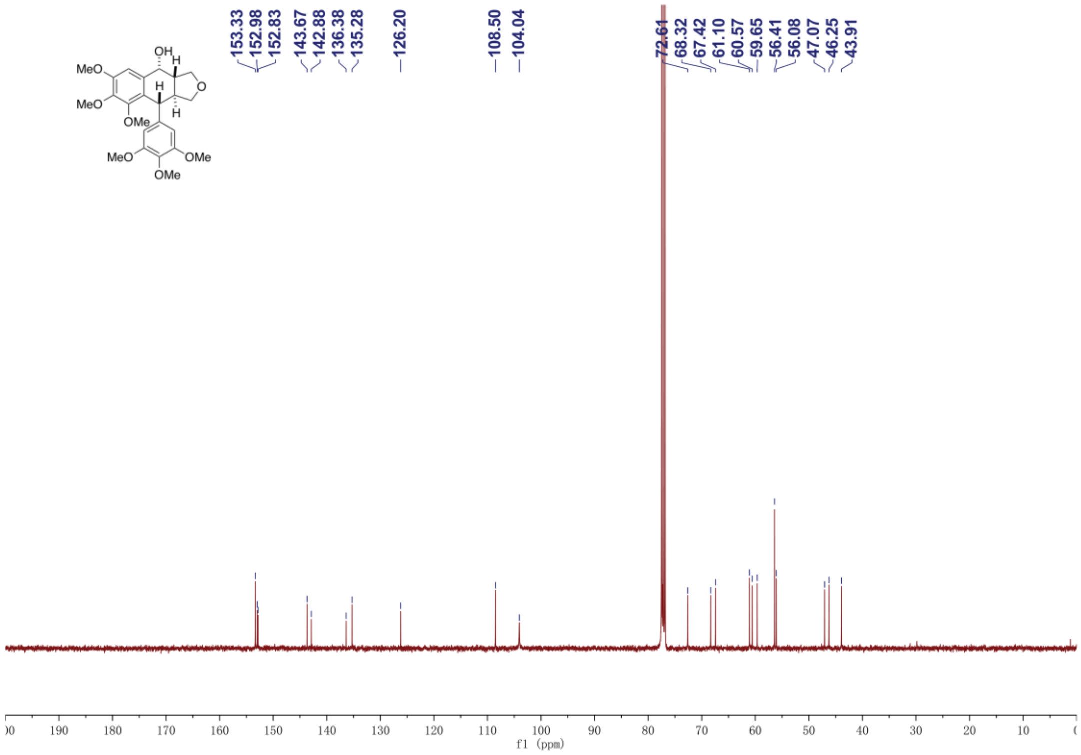

Supplementary Figure 64. 2D NMR spectra of compound **5 (Aglacin F)** ($CDCl_3$)

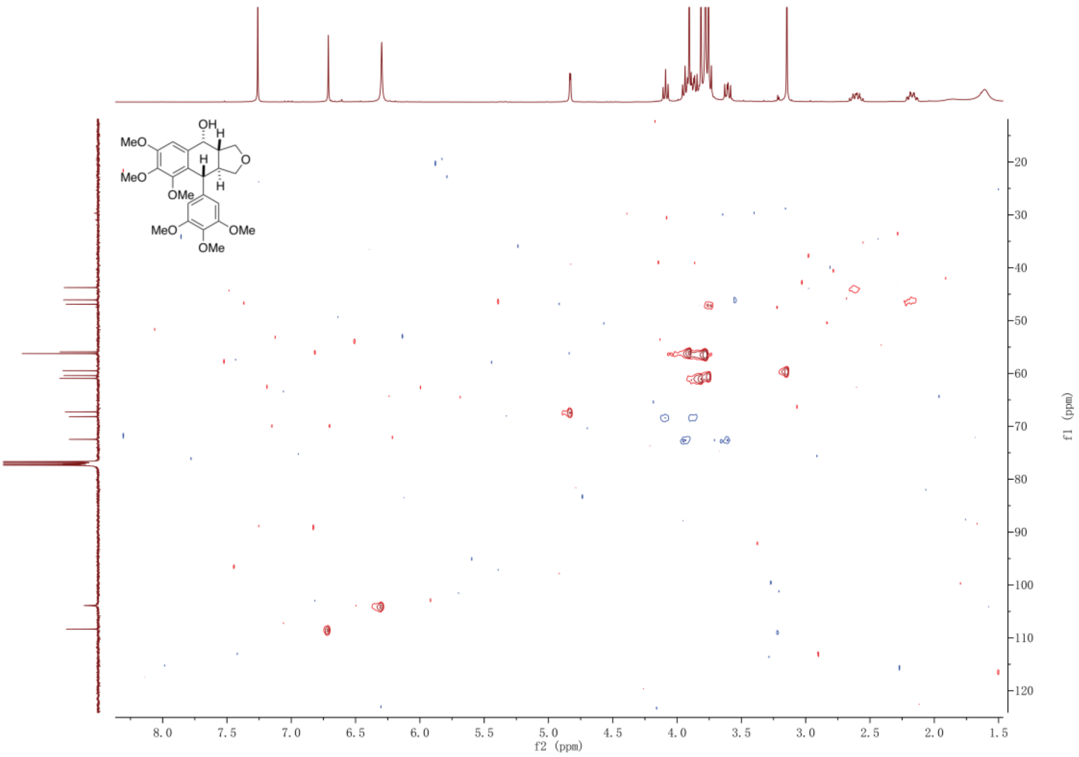

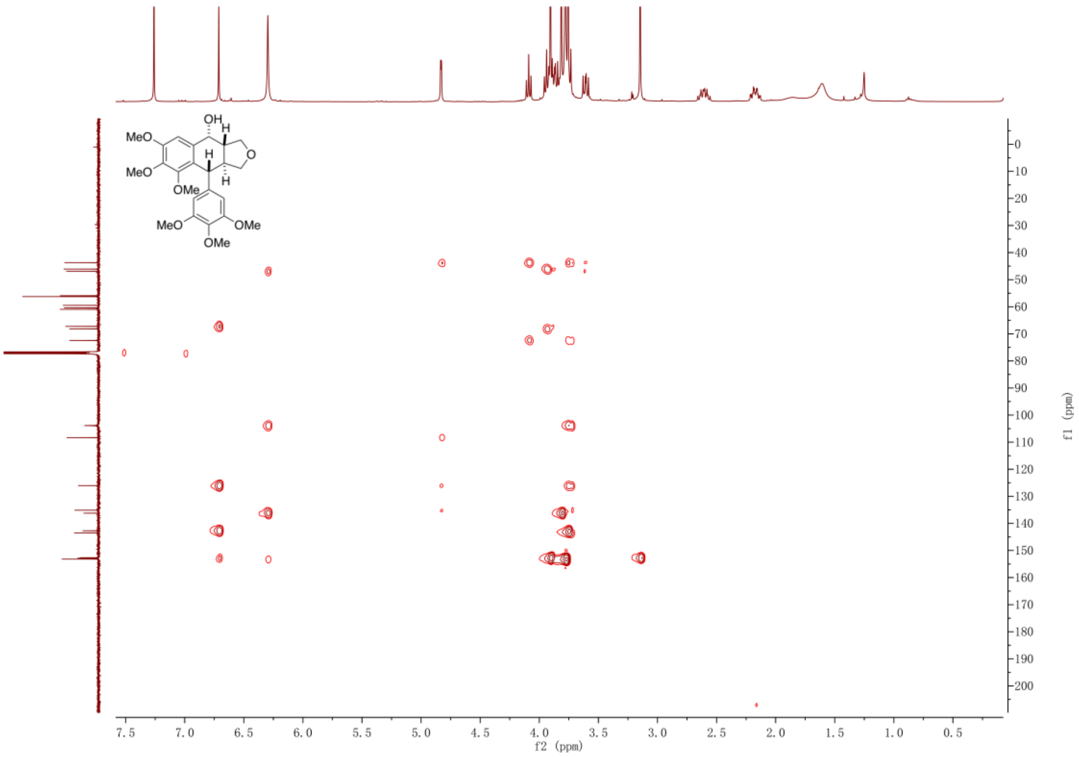

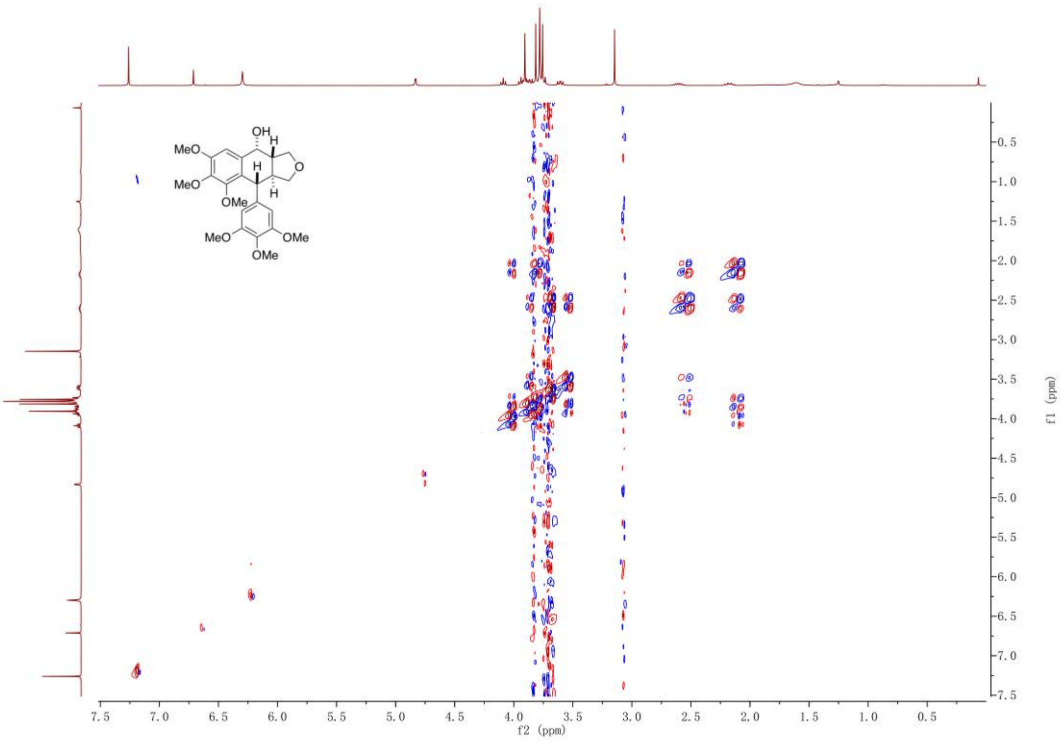
OH
H
H
H
MeO
MeO
OMe
MeO
OMe
OMe
f1 (ppm)
f2 (ppm)

Supplementary Figure 65. ^{1}H NMR spectrum of compound **6 (Aglacin A)** (400 MHz, $CDCl_3$)

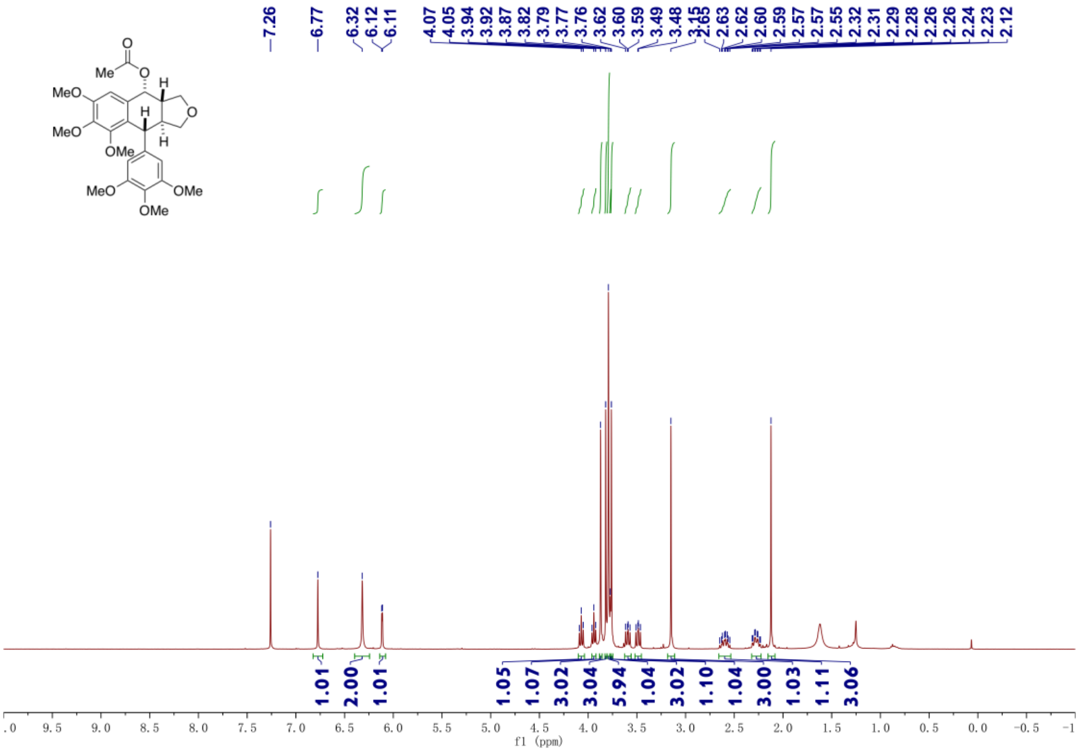

Supplementary Figure 66. ^{13}C NMR spectrum of compound **6 (Aglacin A)** (101 MHz, $CDCl_3$)

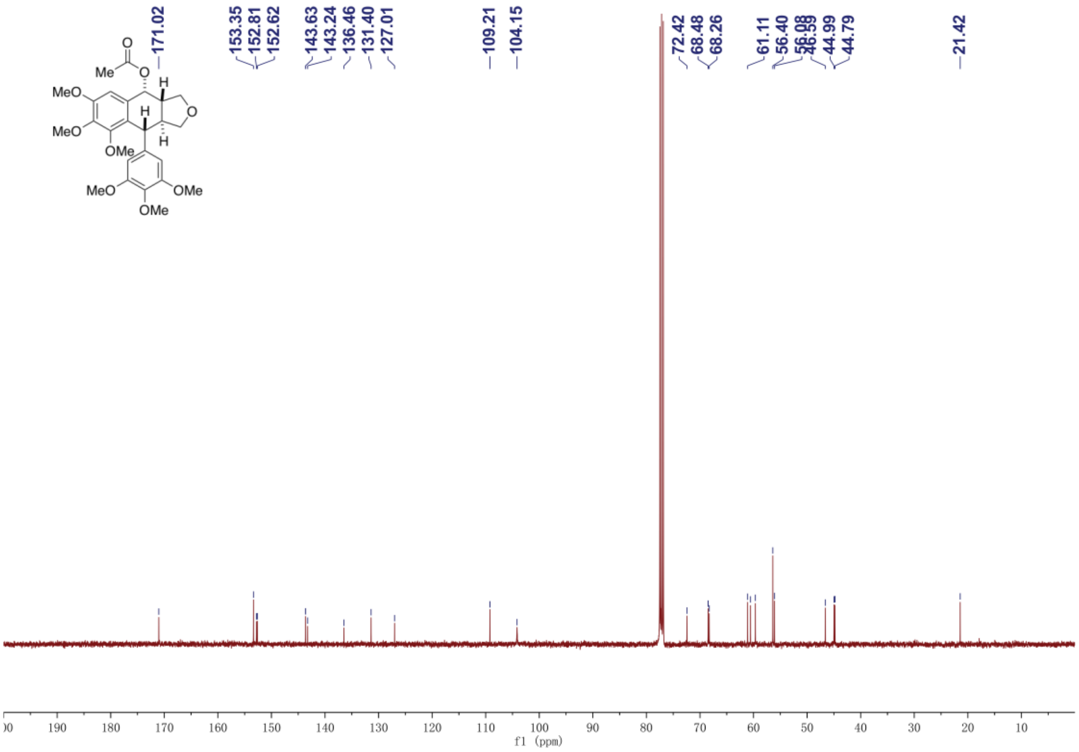

Supplementary Figure 67. 2D spectra of compound **6 (Aglacin A)** ($CDCl_3$)

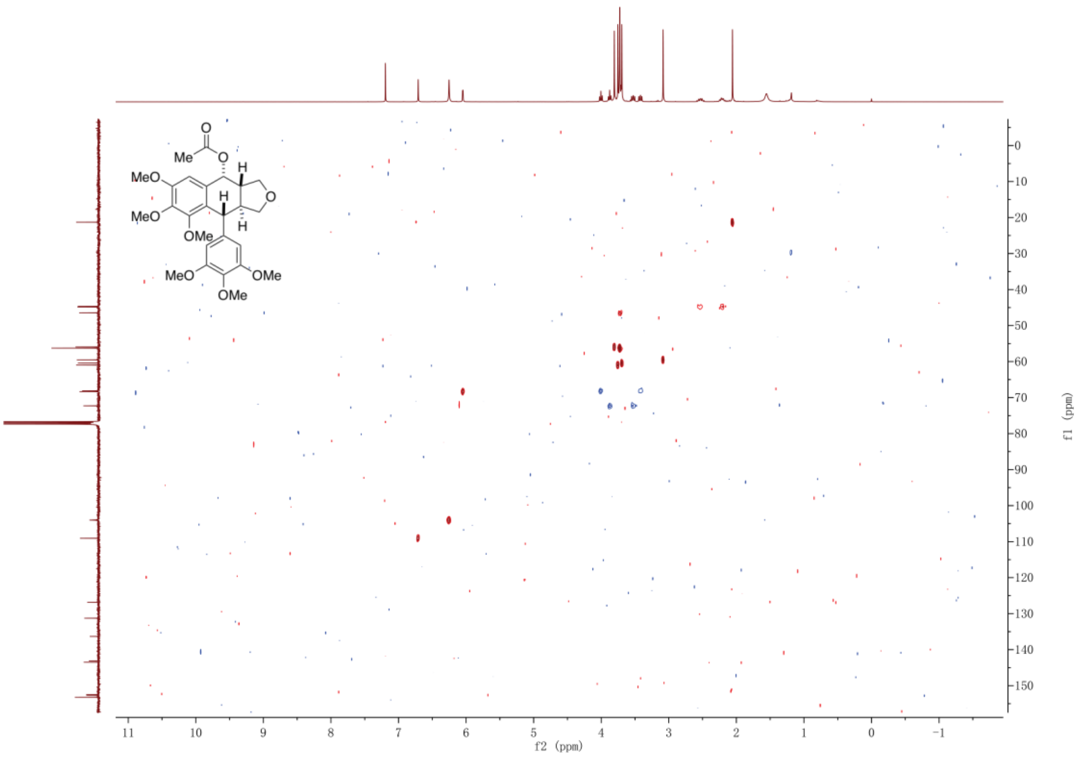

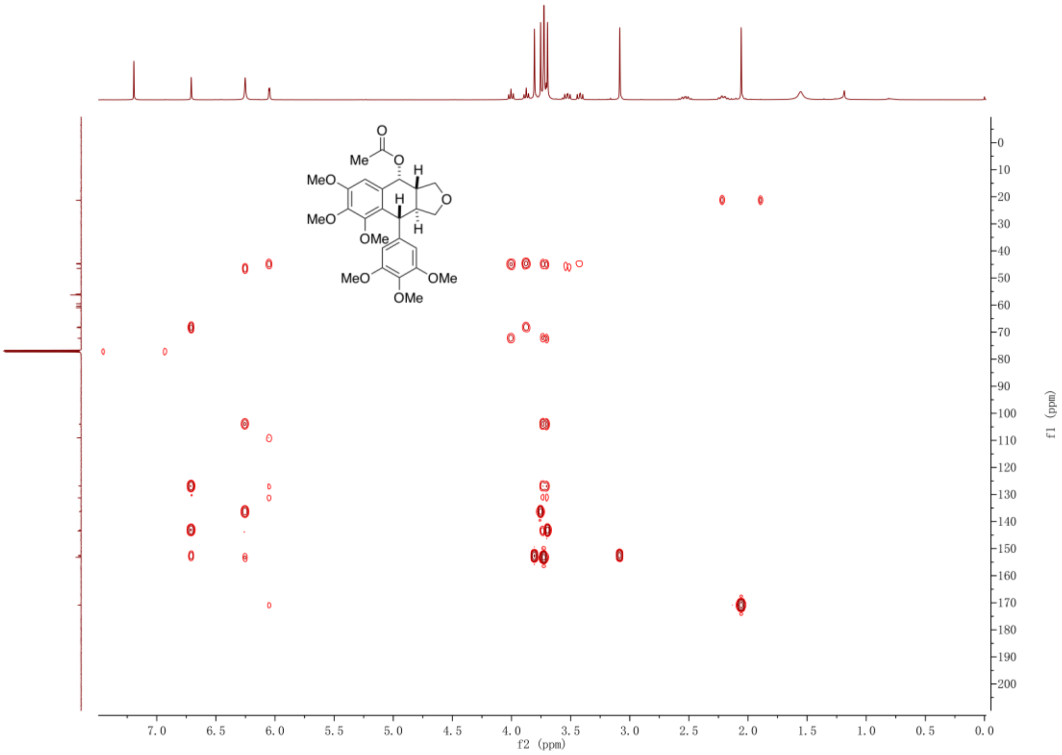

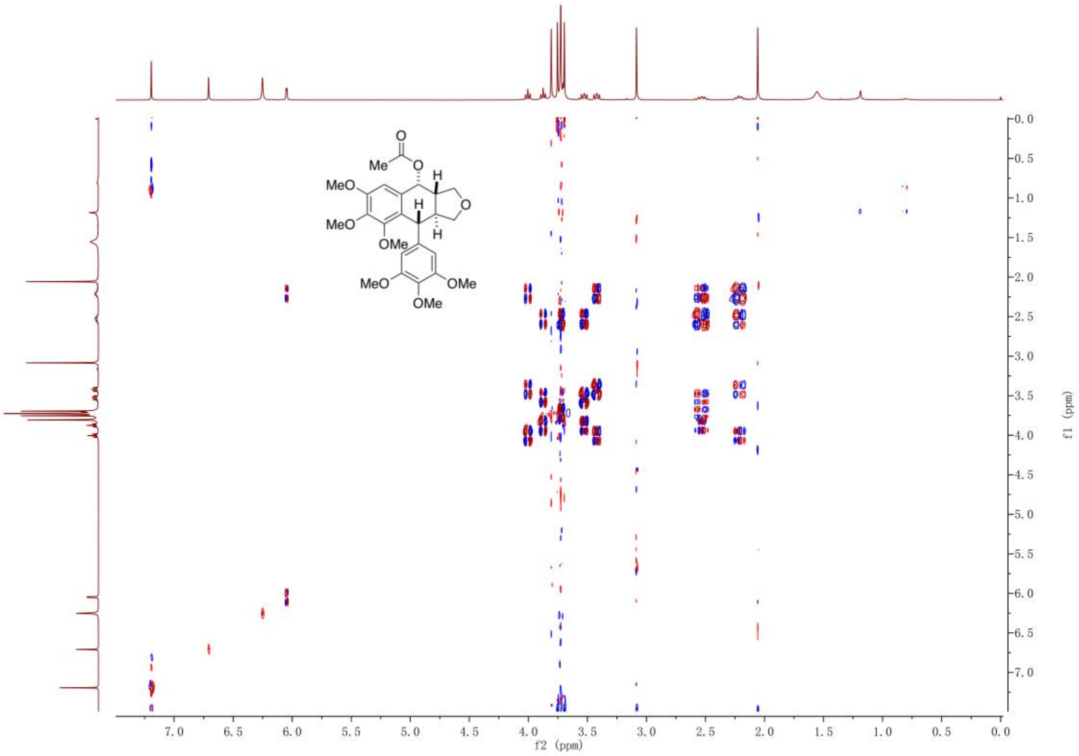
Me
O
MeO
MeO
OMe
H
H
H
O
MeO
OMe
OMe
f2 (ppm)
f1 (ppm)

Supplementary Figure 68. ^{1}H NMR spectrum of compound **21e-A** (400 MHz, $CDCl_3$)

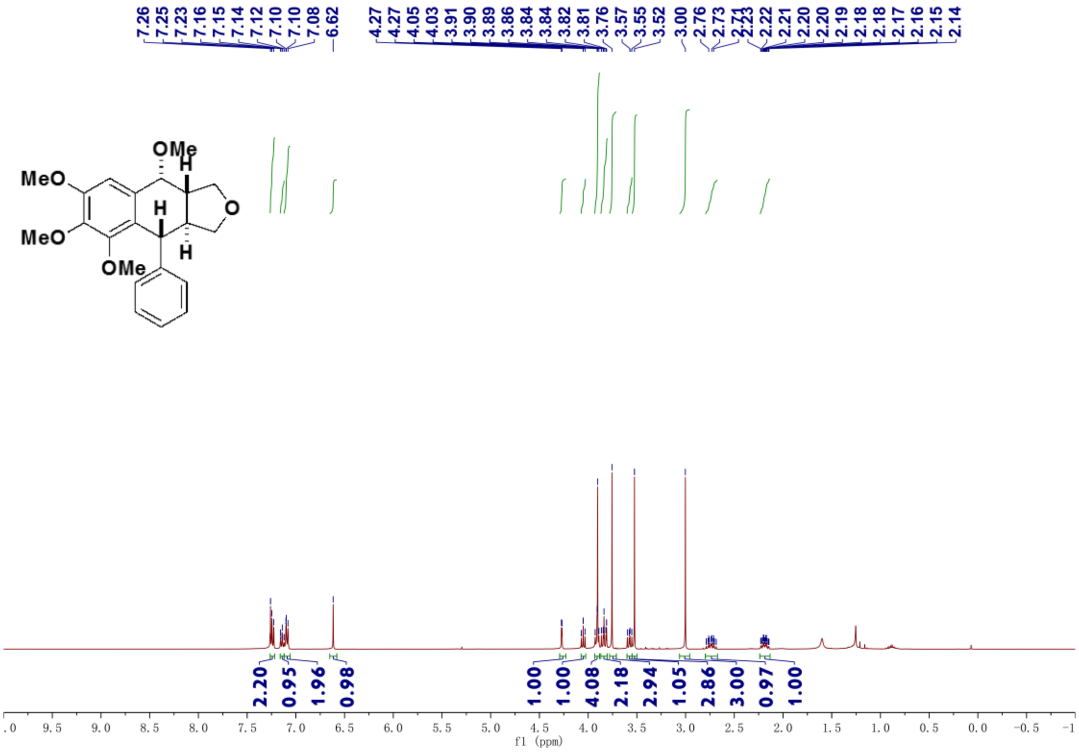

Supplementary Figure 69. ^{13}C NMR spectrum of compound **21e-A** (101 MHz, $CDCl_3$)

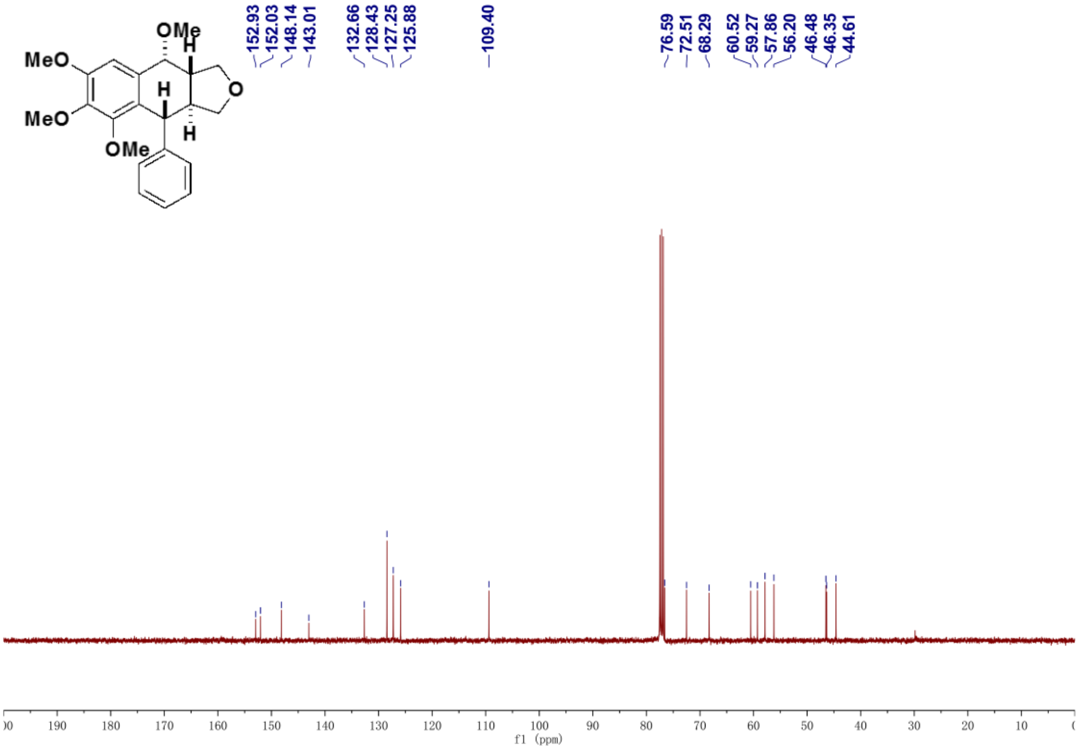

Supplementary Figure 70. ^{1}H NMR spectrum of compound **21e-B** (400 MHz, $CDCl_3$)

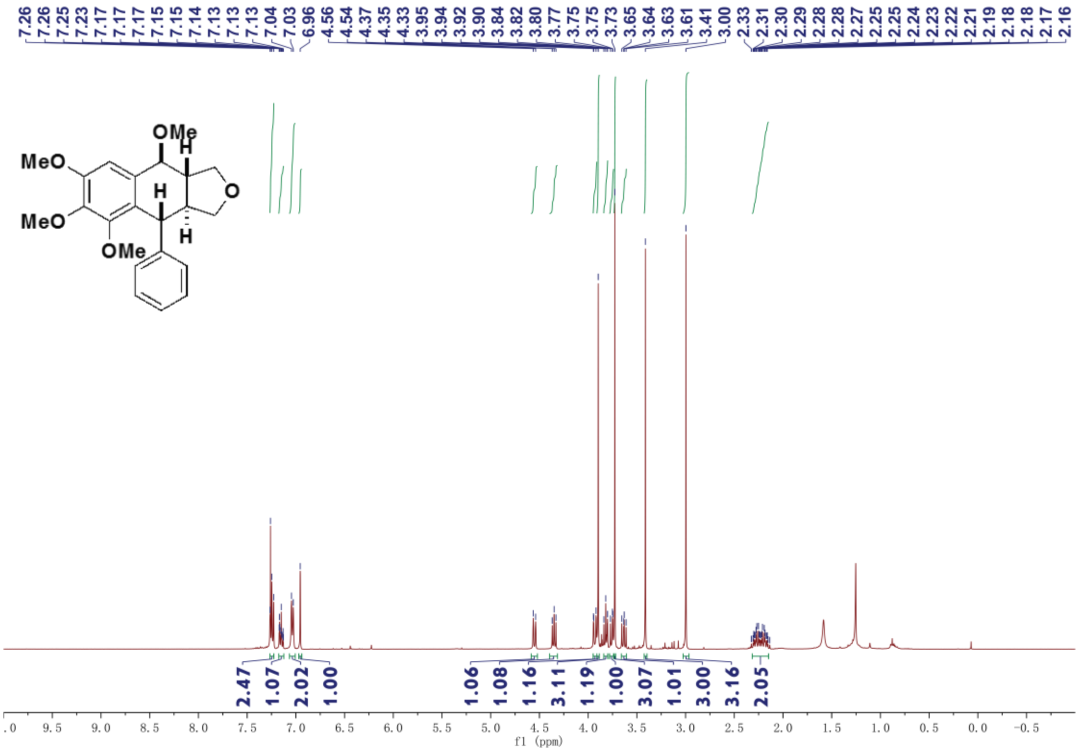

Supplementary Figure 71. ^{13}C NMR spectrum of compound **21e-B** (101 MHz, $CDCl_3$)

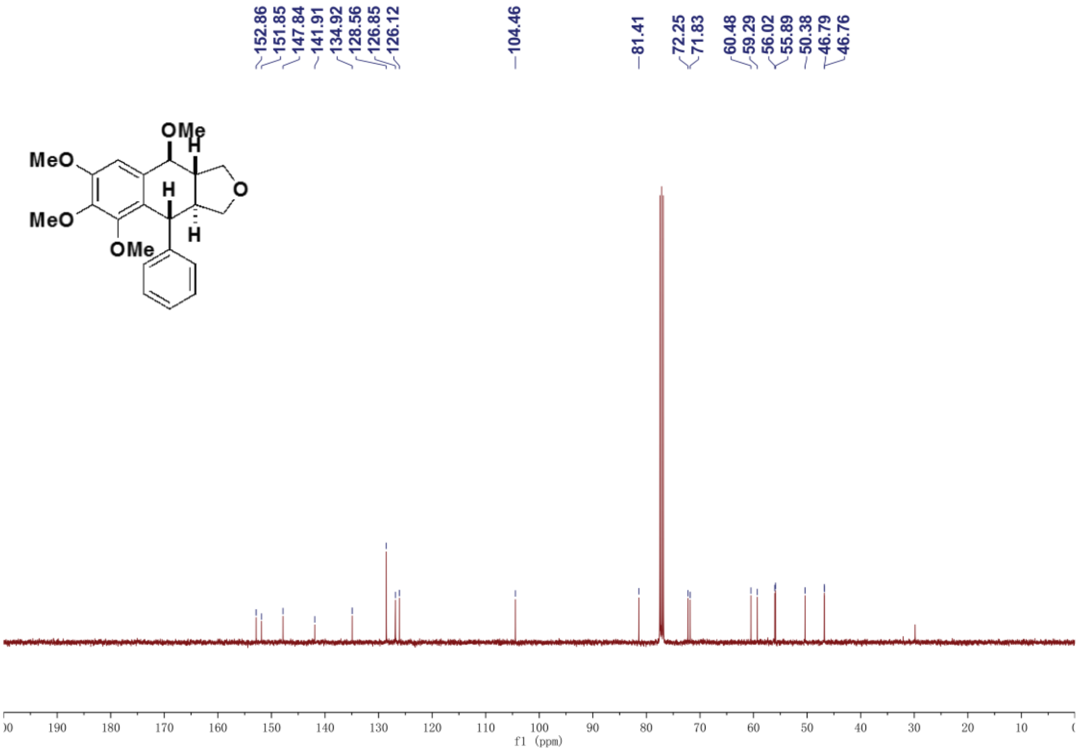

Supplementary Figure 72. ^{1}H NMR spectrum of compound **21b-A** (400 MHz, $CDCl_3$)

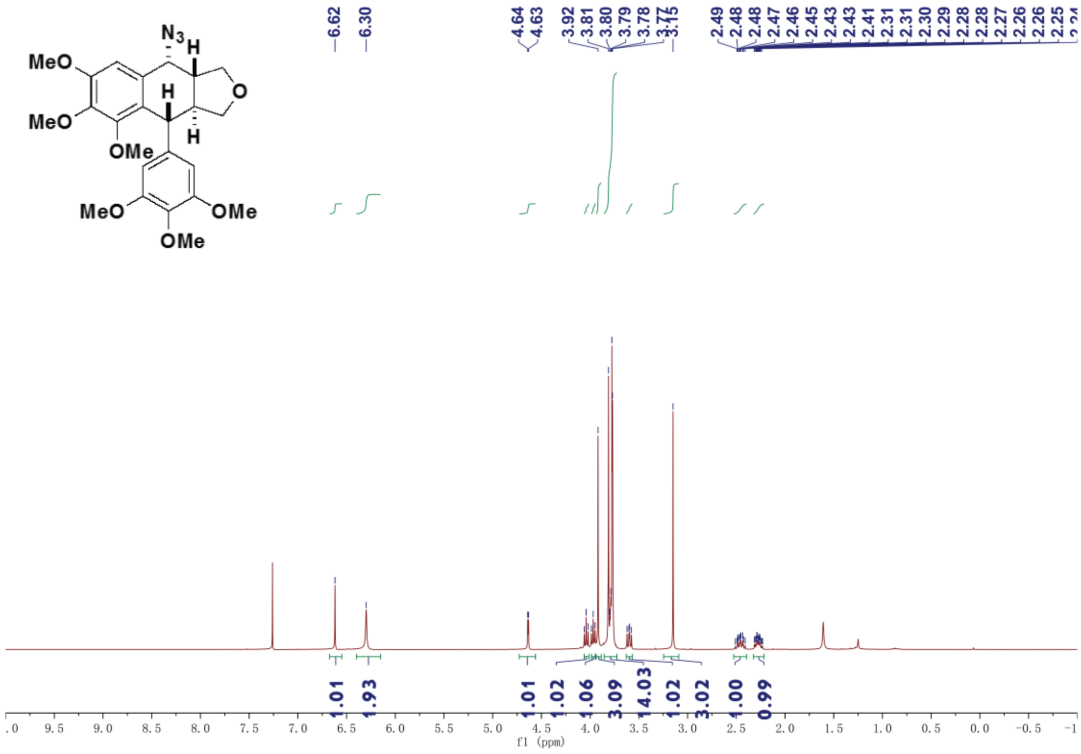

Supplementary Figure 73. ^{13}C NMR spectrum of compound **21b-A** (101 MHz, $CDCl_3$)

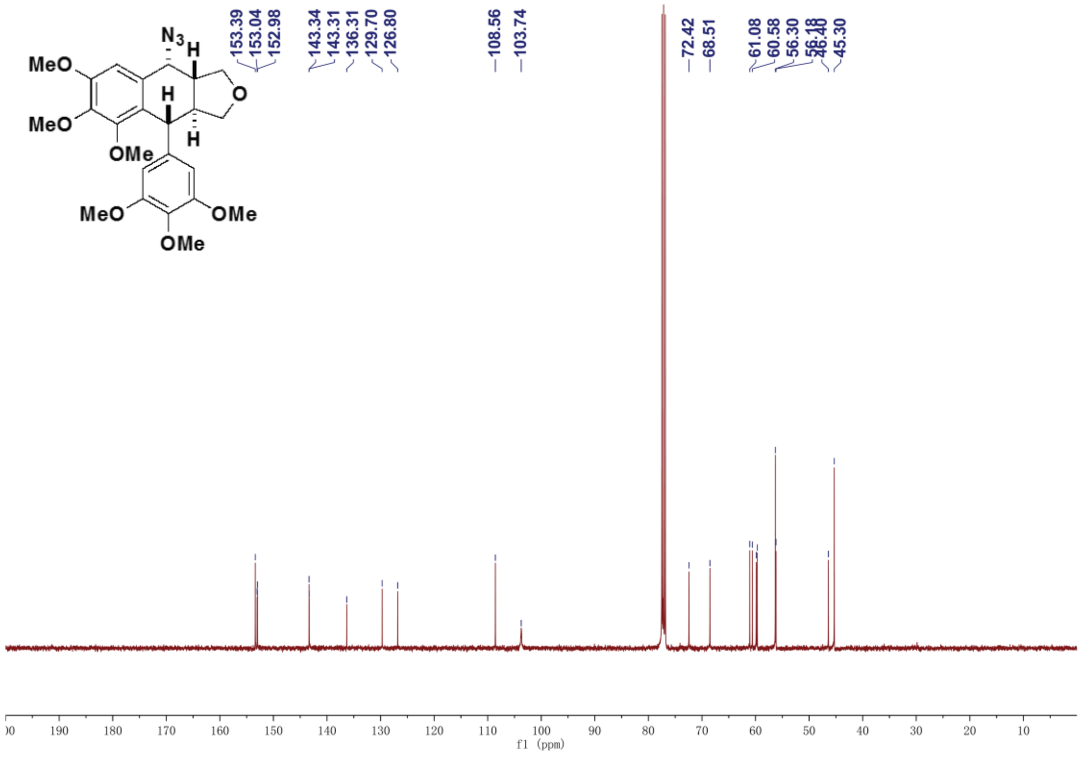

Supplementary Figure 74. ^{1}H NMR spectrum of compound **21c-A** (400 MHz, $CDCl_3$)

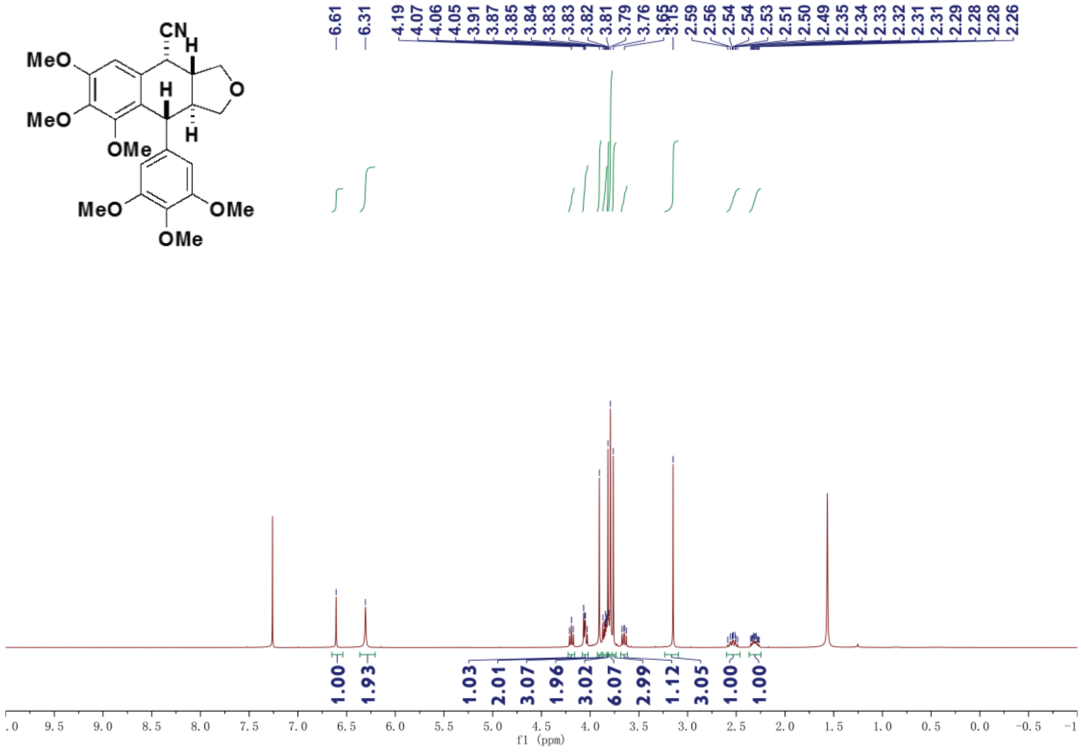

Supplementary Figure 75. ^{13}C NMR spectrum of compound **21c-A** (101 MHz, $CDCl_3$)

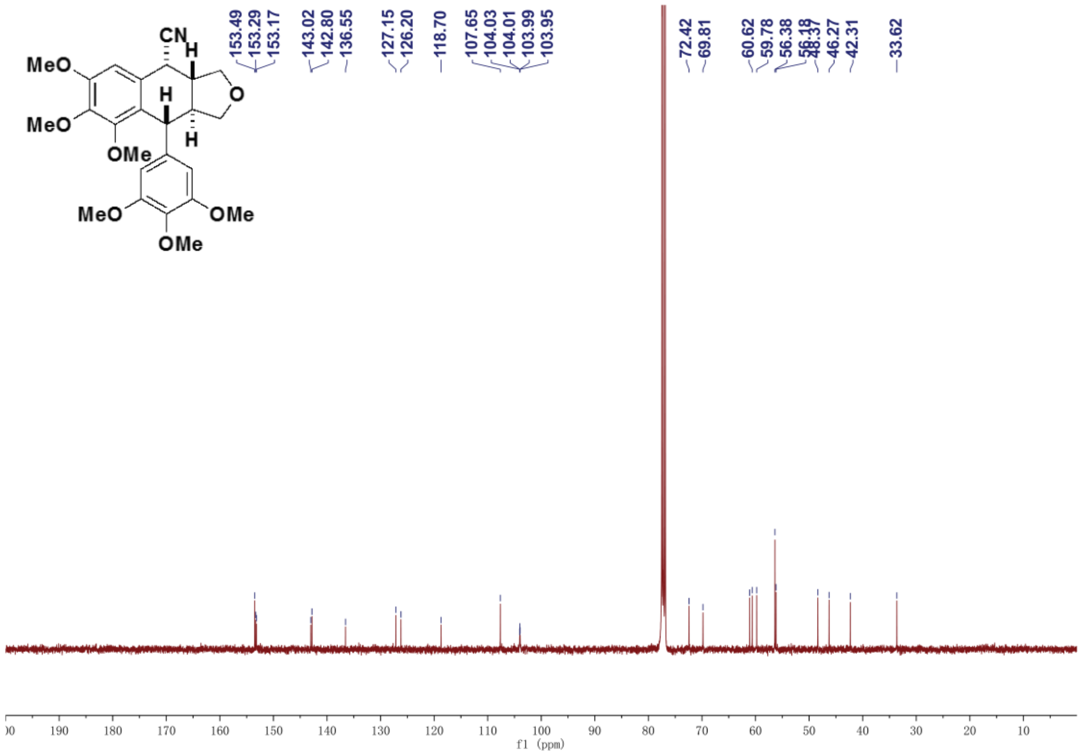

Supplementary Figure 76. 2D NMR spectra of compound **21c-A** ($CDCl_3$)

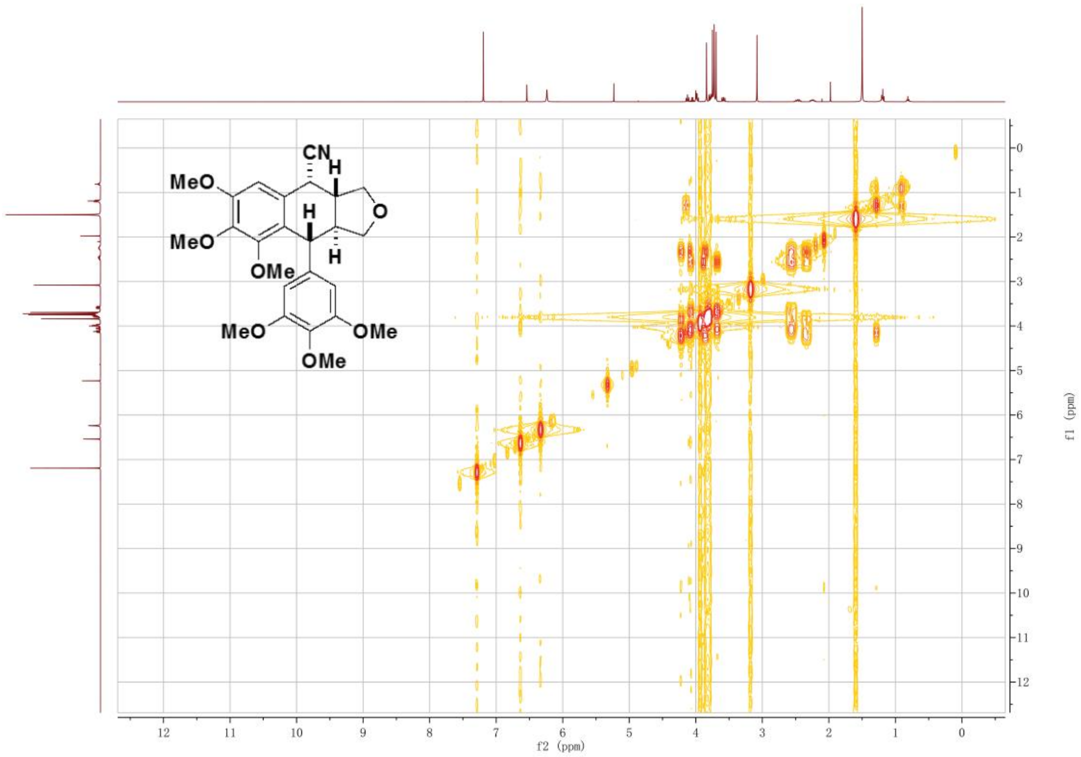

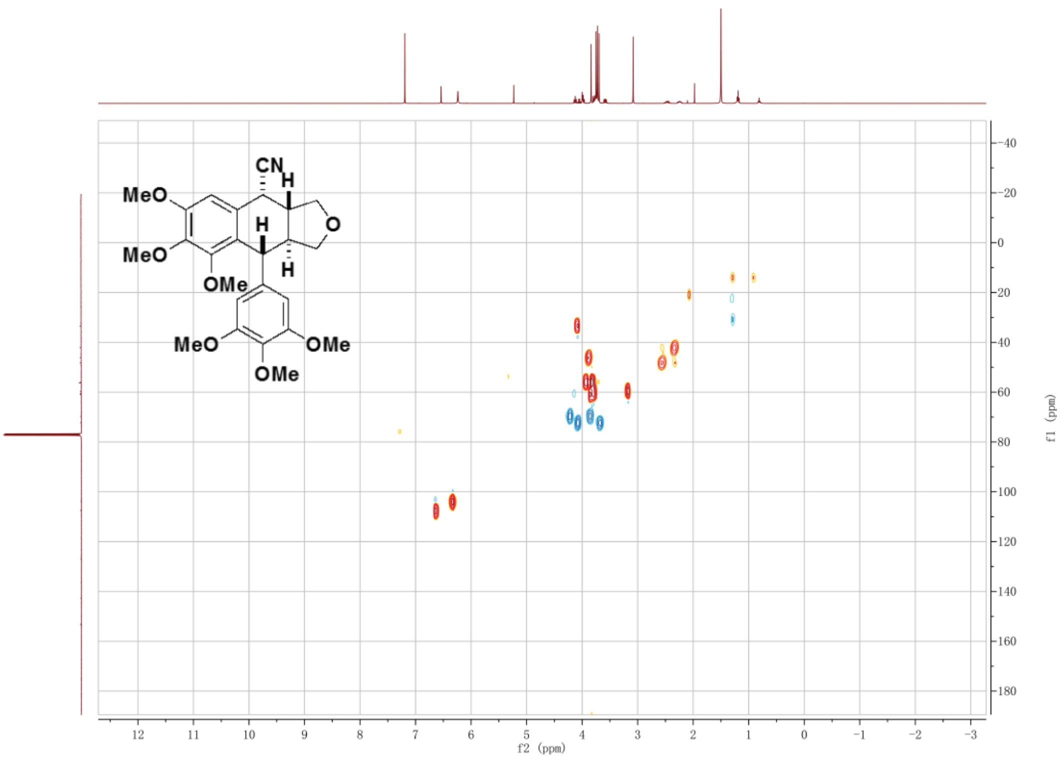

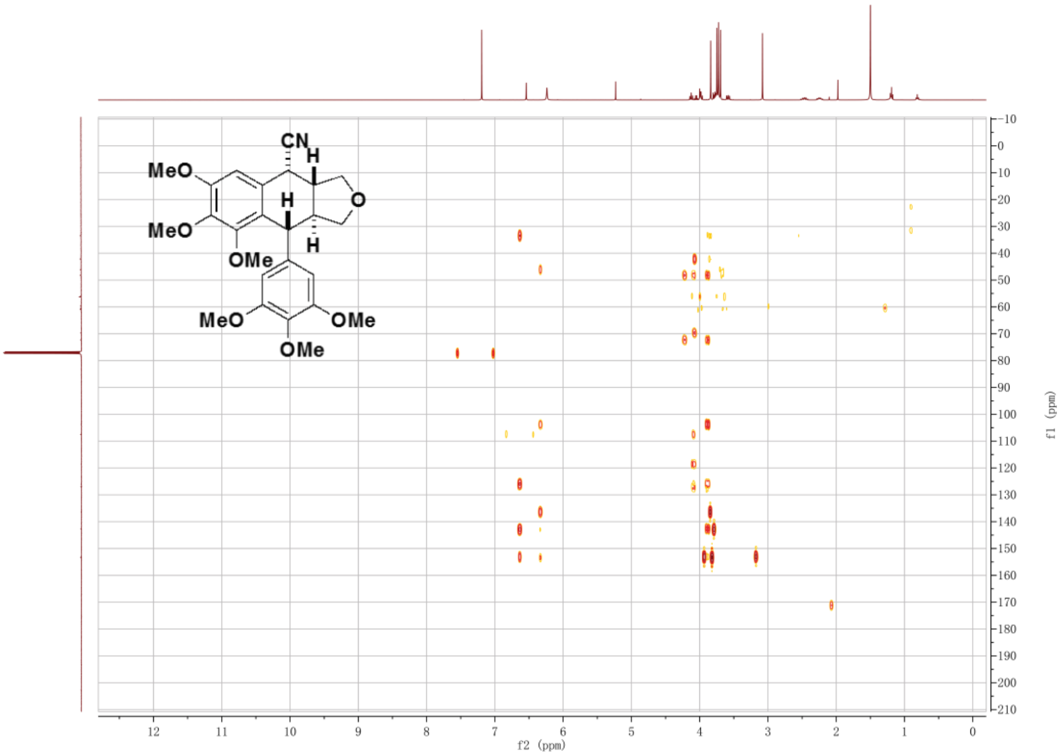
CN
H
MeO
MeO
O
H
H
OMe
MeO
OMe
OMe
f2 (ppm)
f1 (ppm)

Supplementary Figure 77. ^{1}H NMR spectrum of compound **21d (A+B)** (400 MHz, $CDCl_3$)

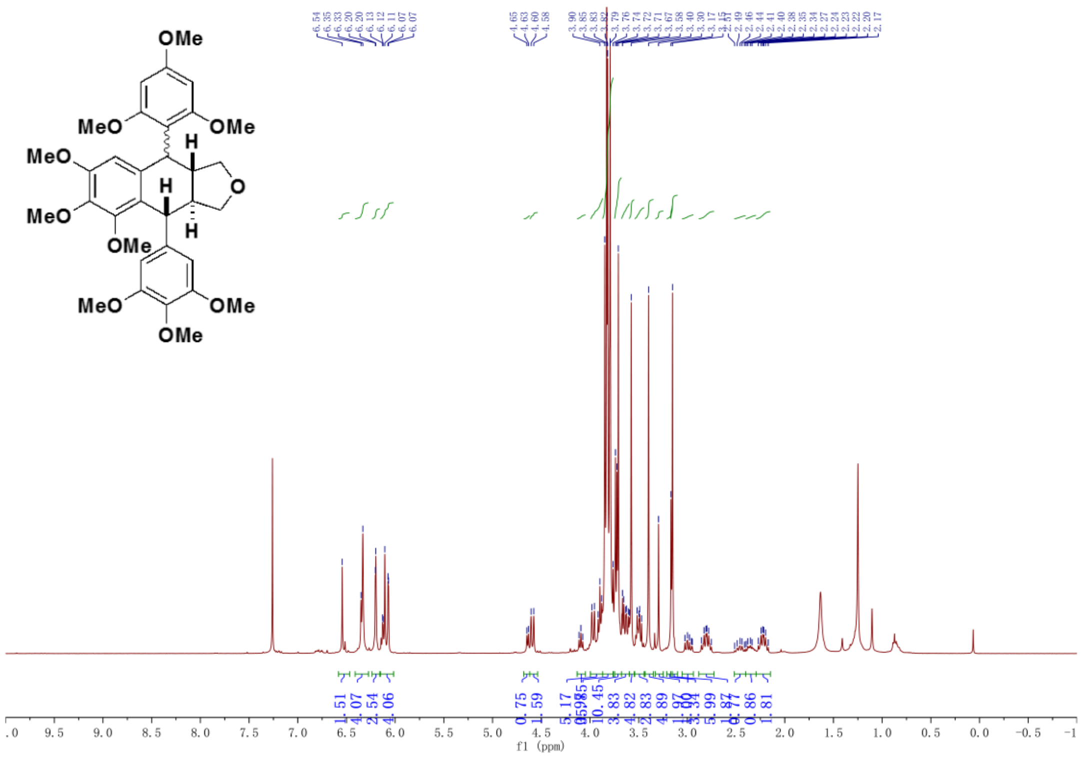

Supplementary Figure 78. ^{13}C NMR spectrum of compound **21d (A+B)** (101 MHz, $CDCl_3$)

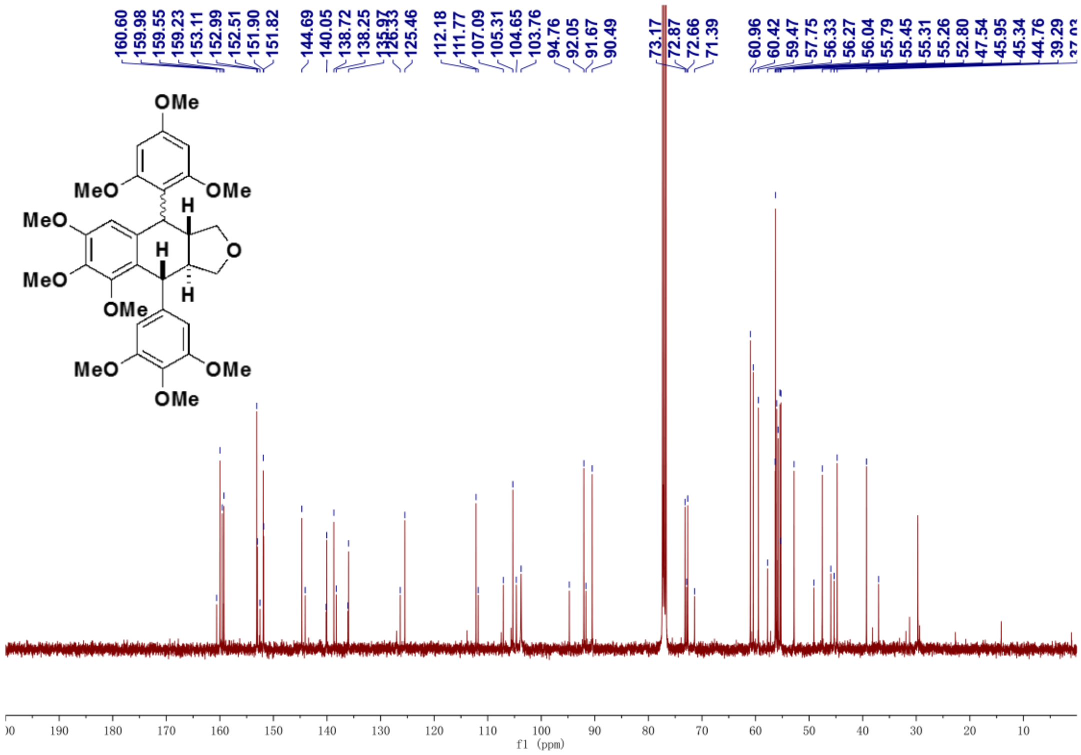

Supplementary Figure 79. ^{1}H NMR spectrum of compound **22a-A** (400 MHz, $CDCl_3$)

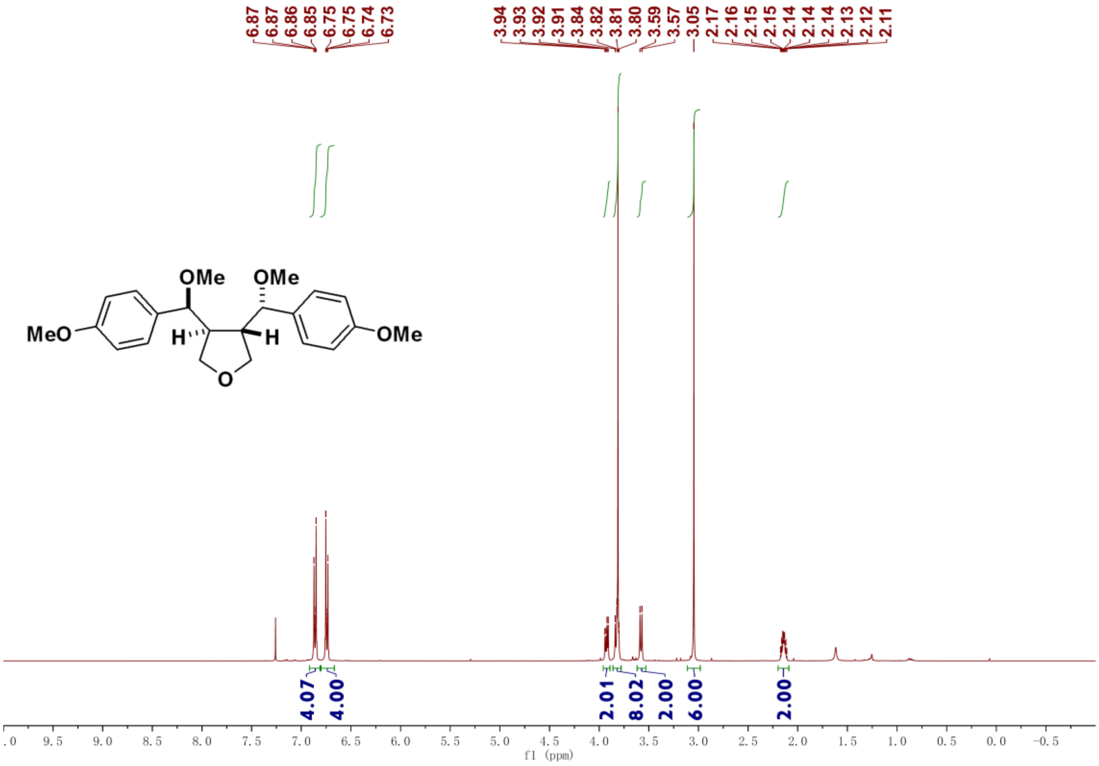

Supplementary Figure 80. ^{13}C NMR spectrum of compound **22a-A** (101 MHz, $CDCl_3$)

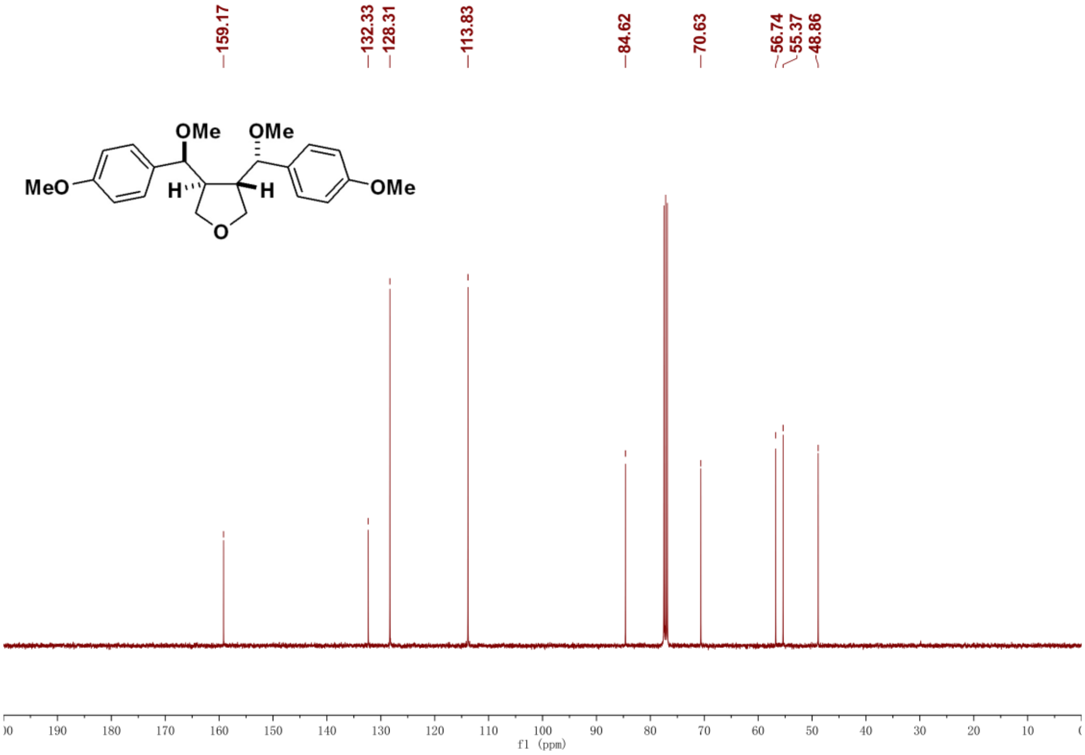

Supplementary Figure 81. 2D NMR spectra of compound **22a-A** ($CDCl_3$)

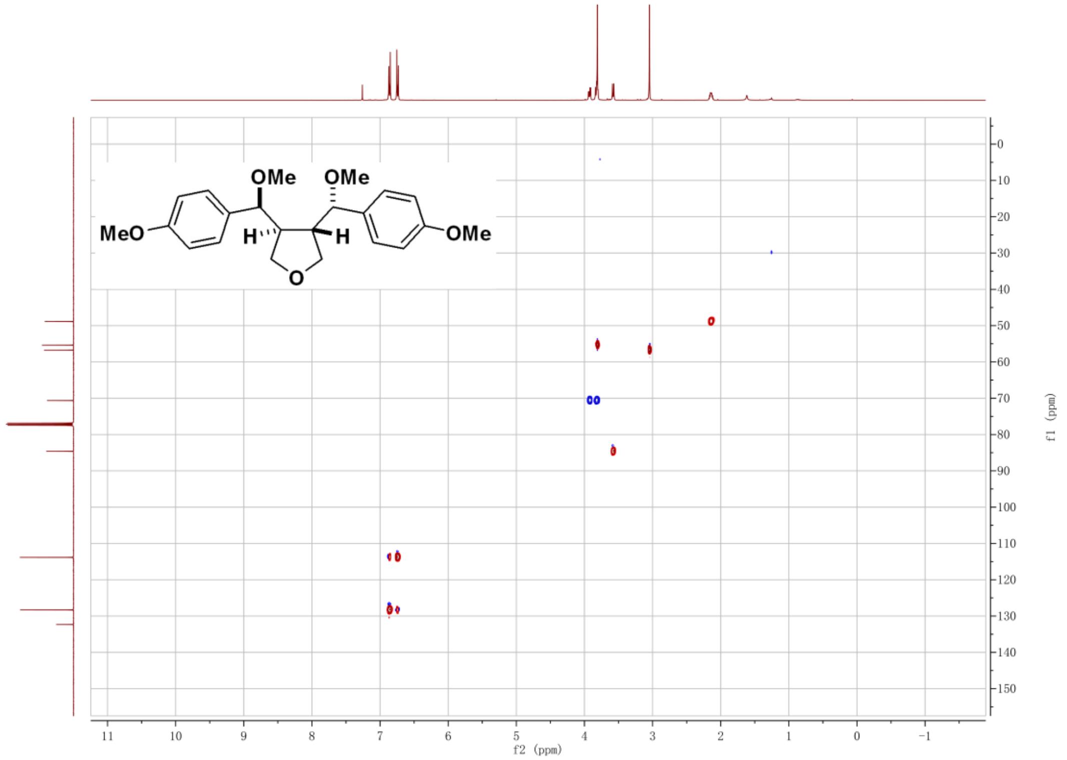

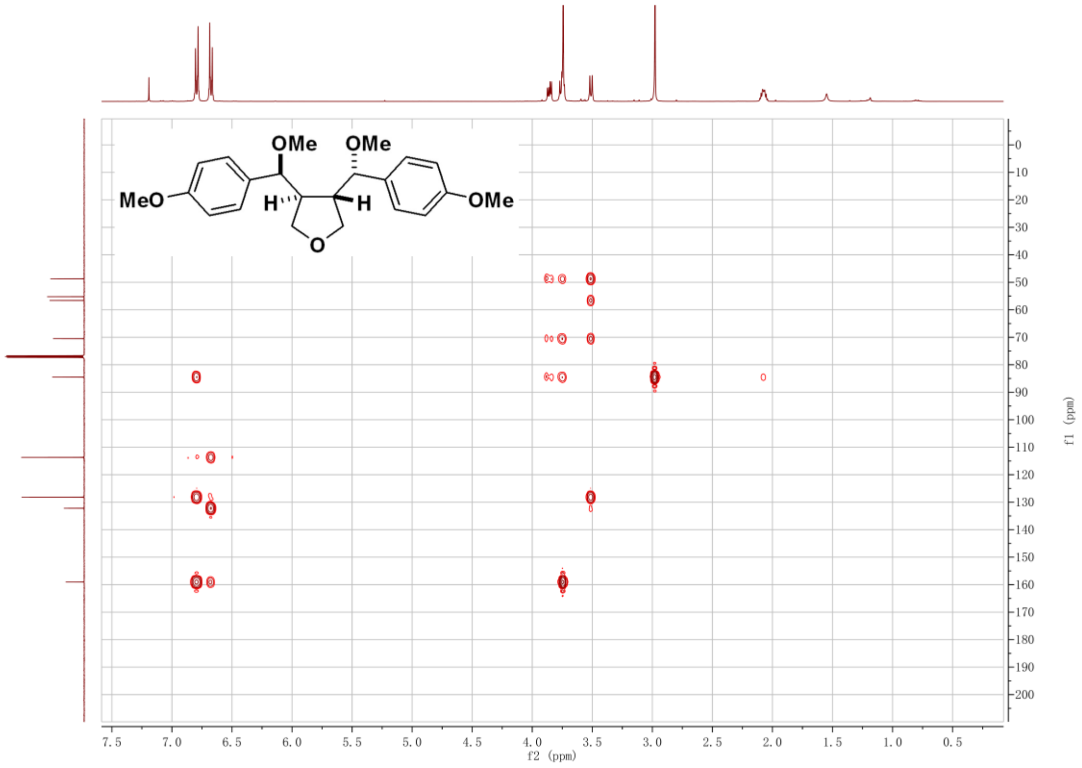

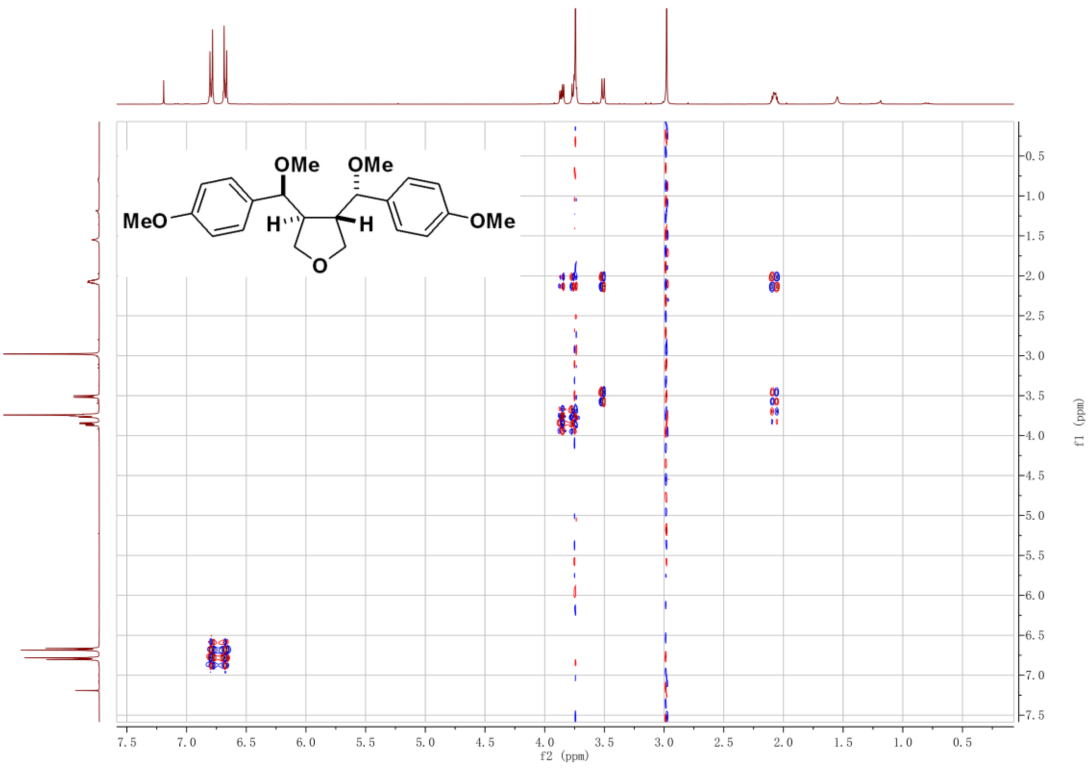
OMe
OMe
MeO
H
H
O
OMe
f2 (ppm)
f1 (ppm)

Supplementary Figure 82. ^{1}H NMR spectrum of compound **22a-B** (400 MHz, $CDCl_3$)

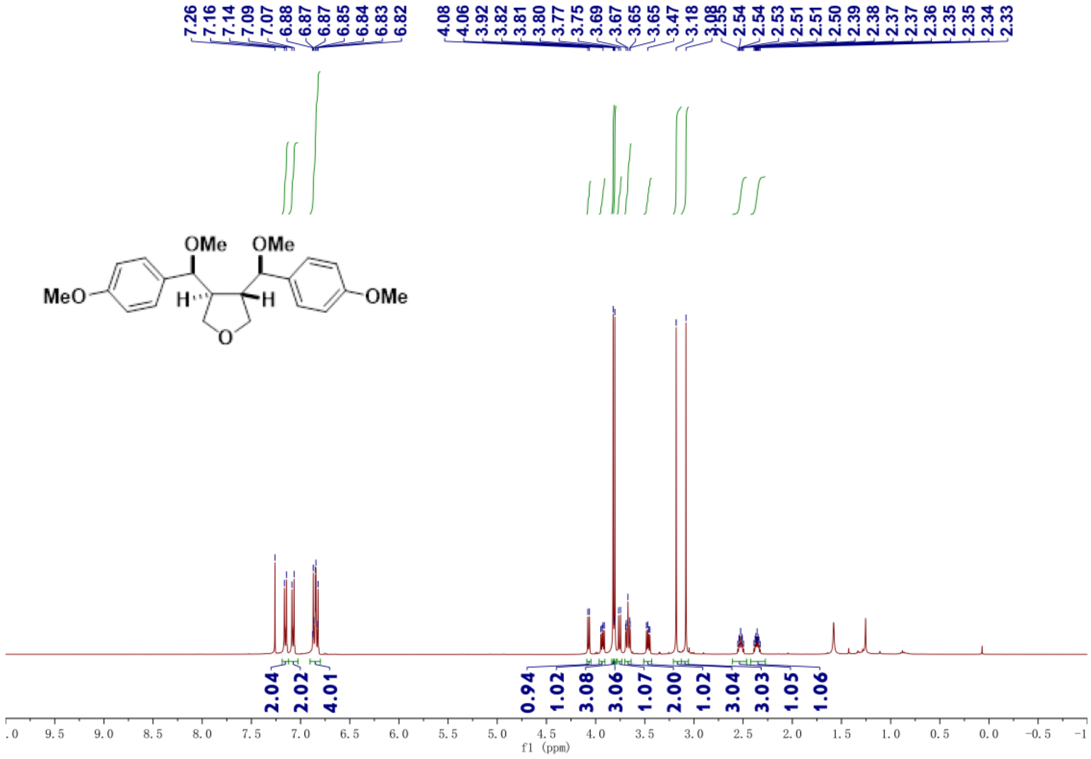

Supplementary Figure 83. ^{13}C NMR spectrum of compound **22a-B** (101 MHz, $CDCl_3$)

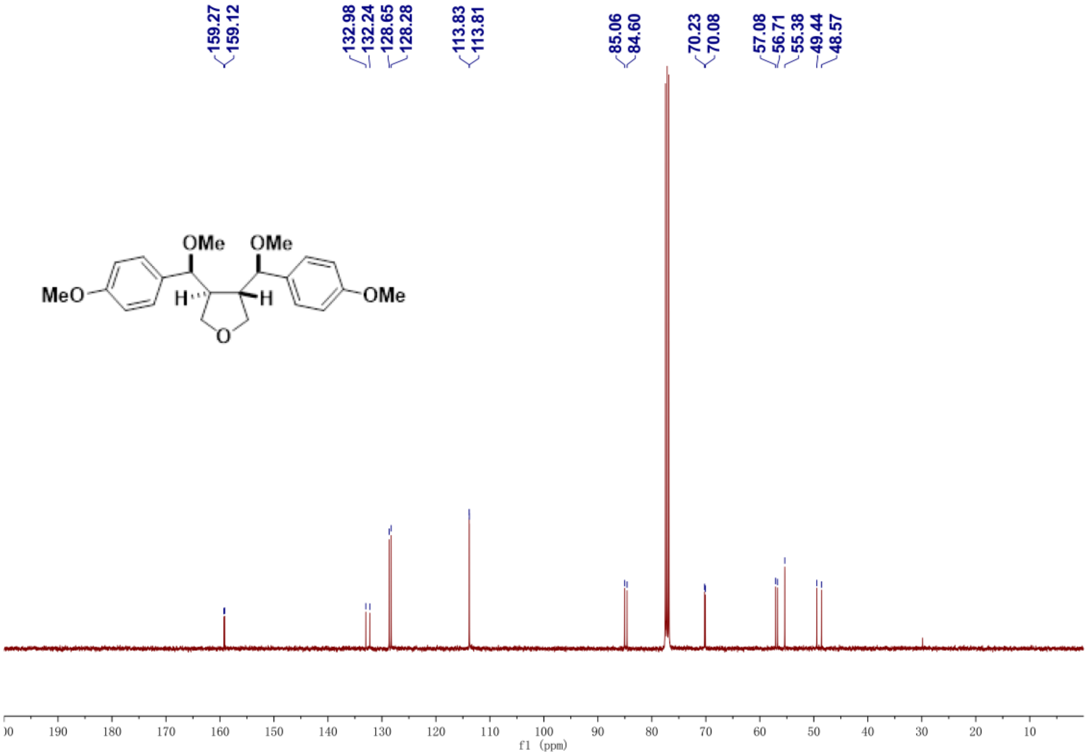

Supplementary Figure 84. 2D NMR spectra of compound **22a-B** ($CDCl_3$)

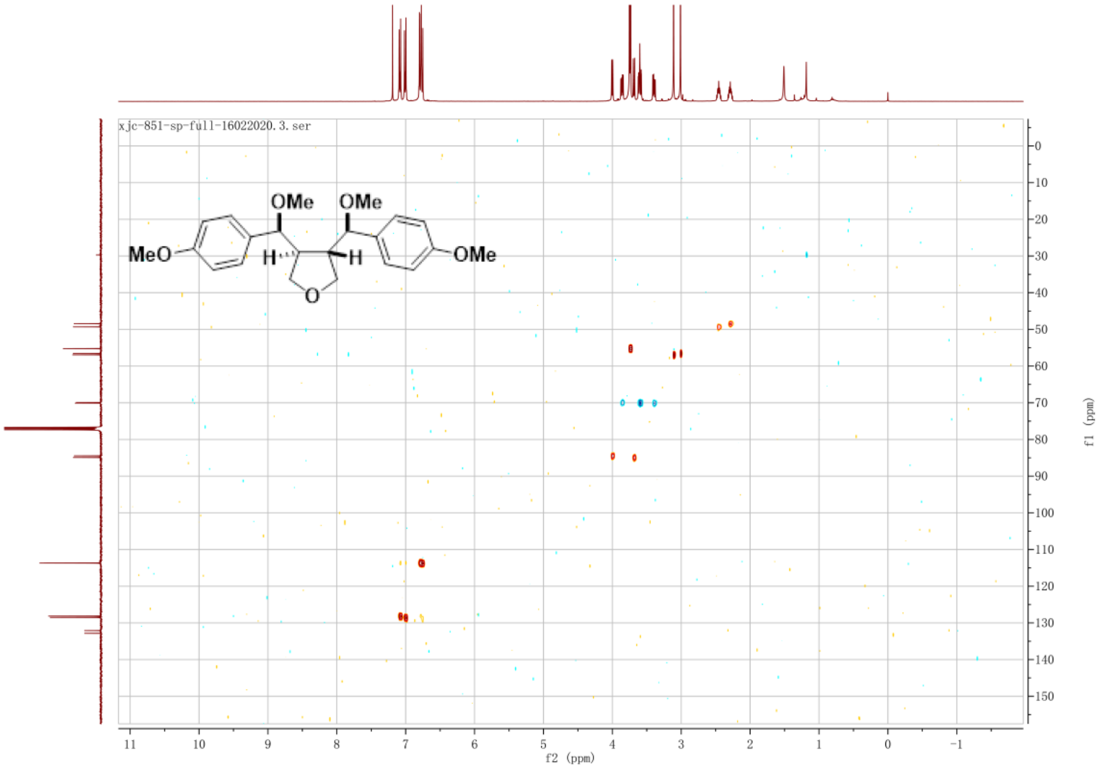

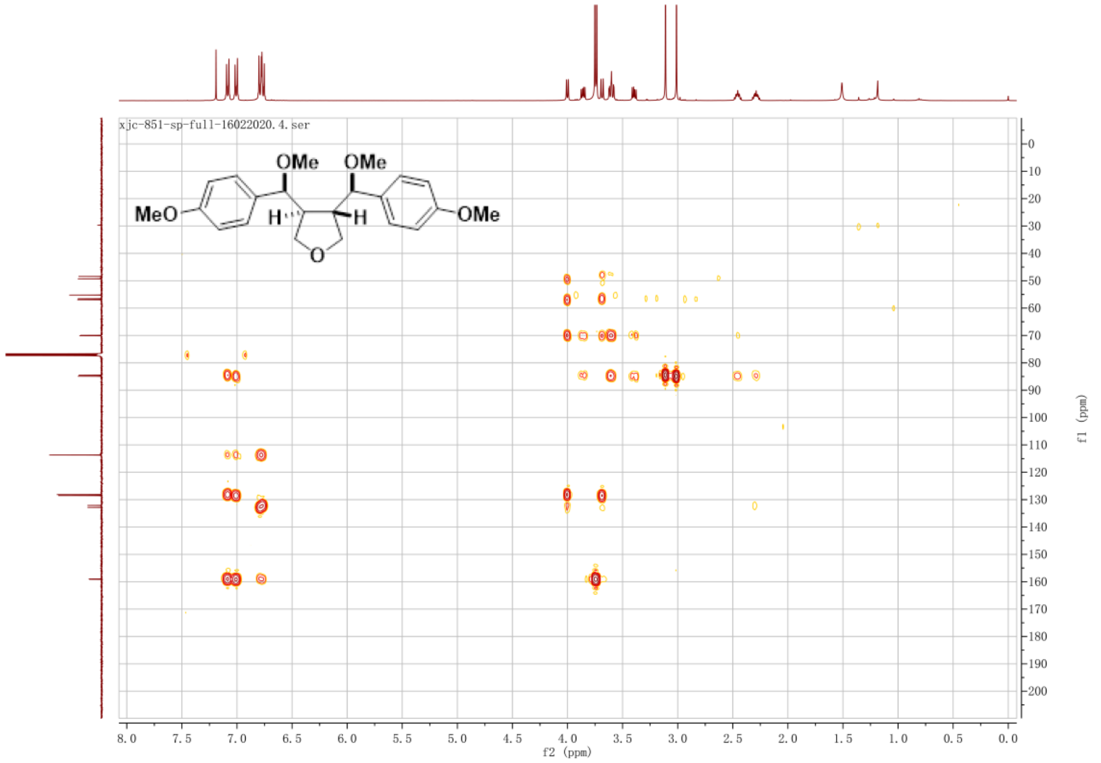

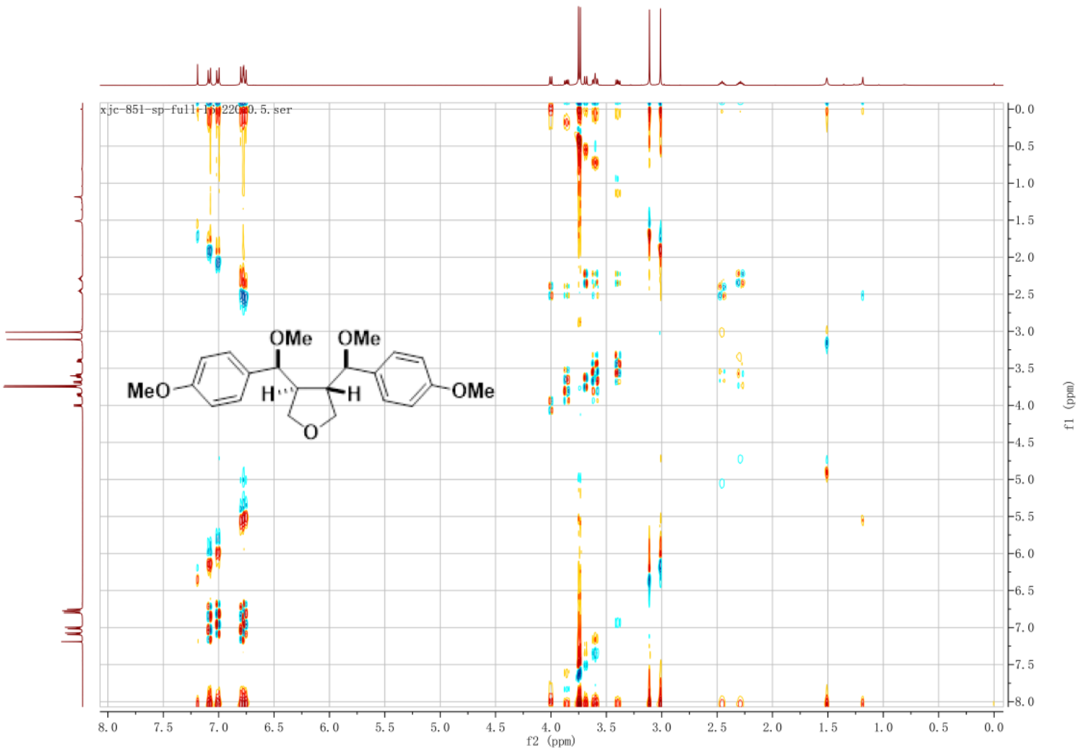

xjc-851-sp-full-[illegible]0.5.ser
OMe
OMe
MeO
H
H
O
OMe
f2 (ppm)
f1 (ppm)

Supplementary Figure 85. ^{1}H NMR spectrum of compound **22a-C (another diastereomer which had not been detected in photocyclization reaction)** (400 MHz, $CDCl_3$)

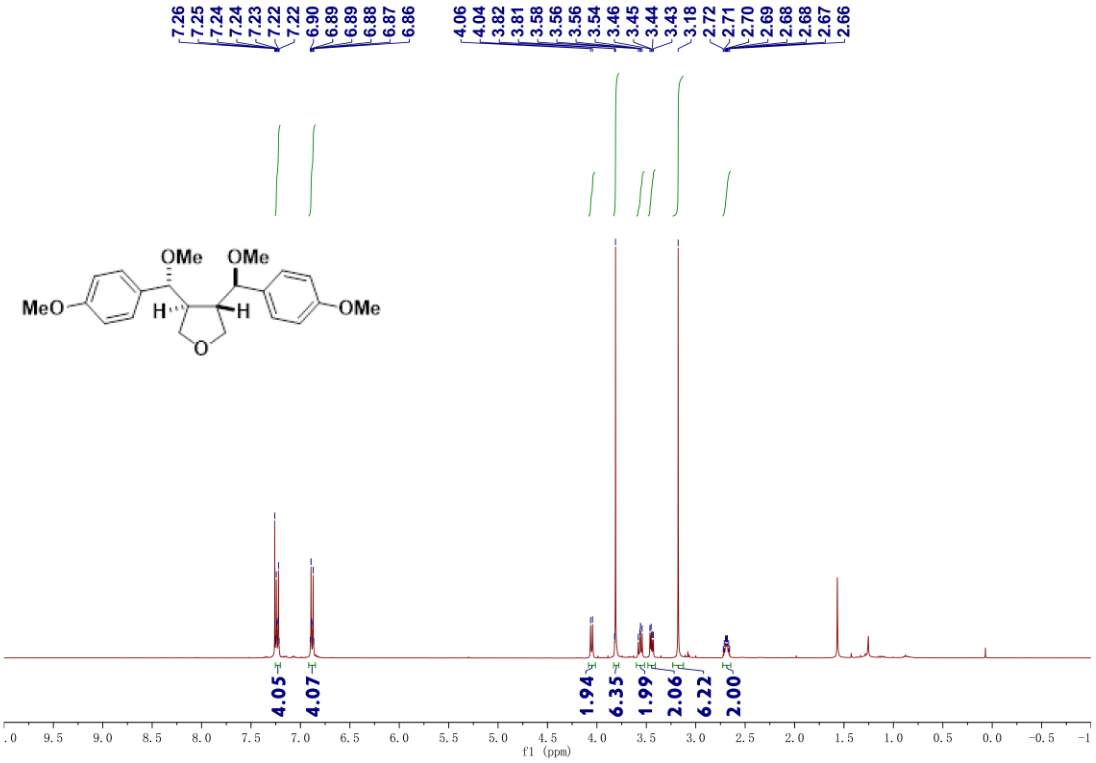

Supplementary Figure 86. ^{13}C NMR spectrum of compound **22a-C (another diastereomer which had not been detected in photocyclization reaction)** (101 MHz, $CDCl_3$)

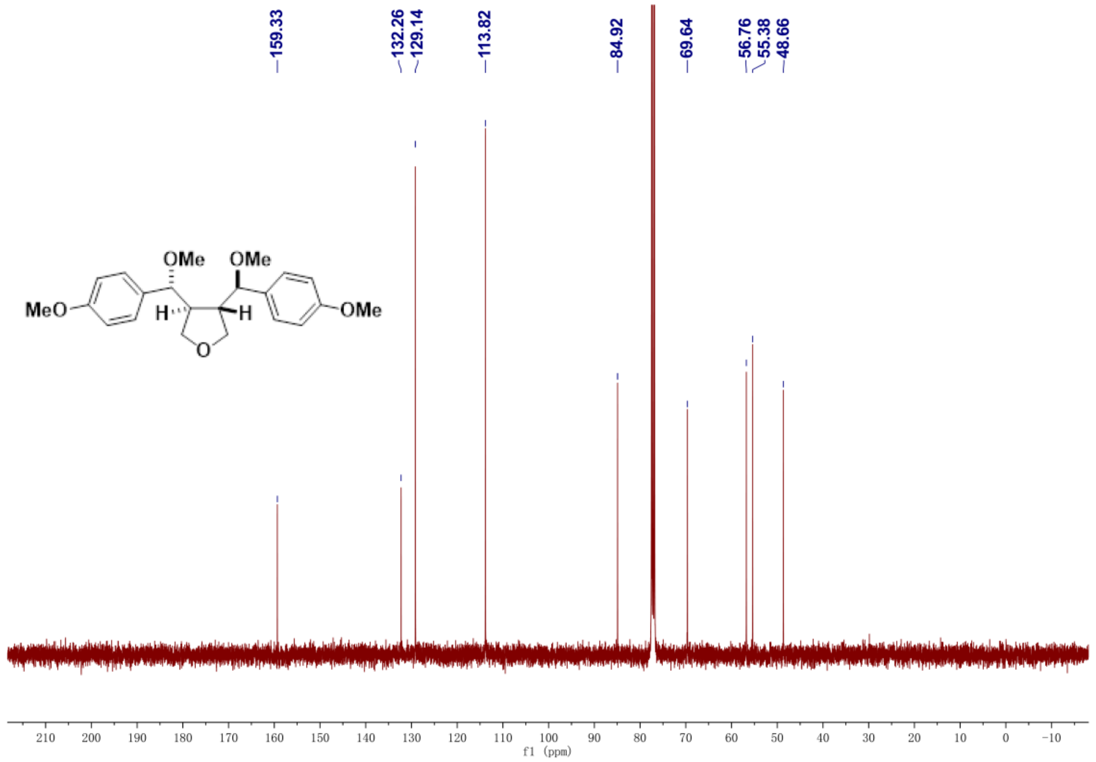

Supplementary Figure 87. 2D NMR spectra of compound **22a-C (another diastereomer which had not been detected in photocyclization reaction)** (CDCl$_3$)

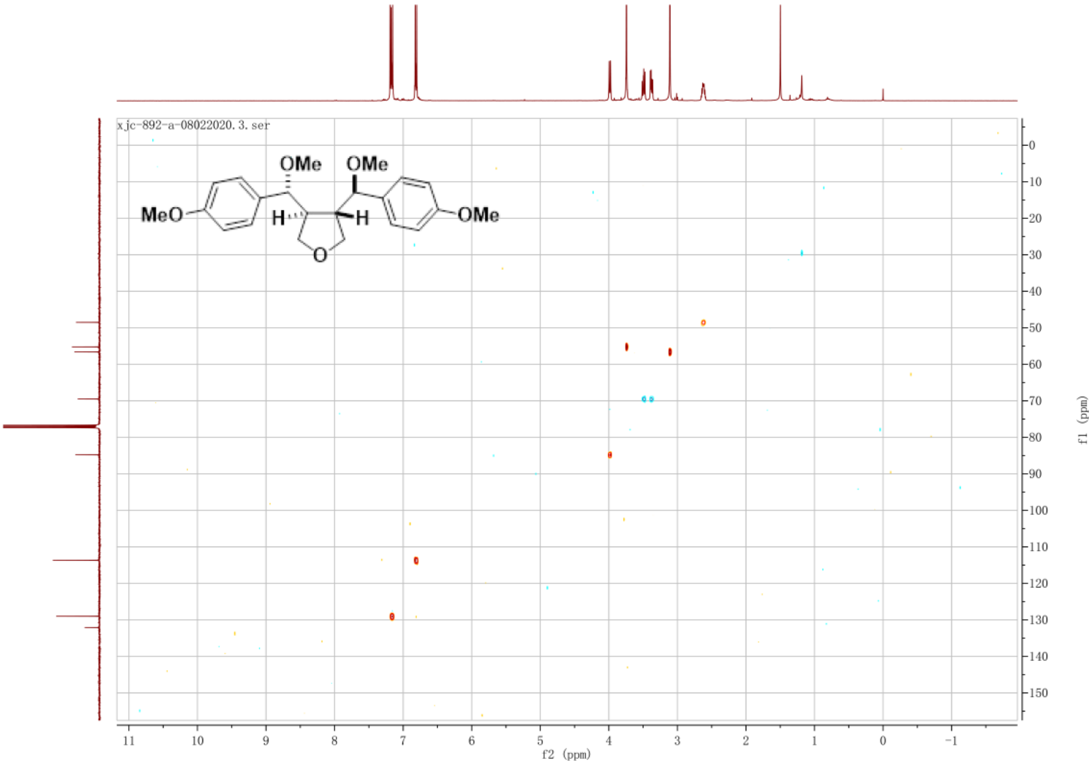

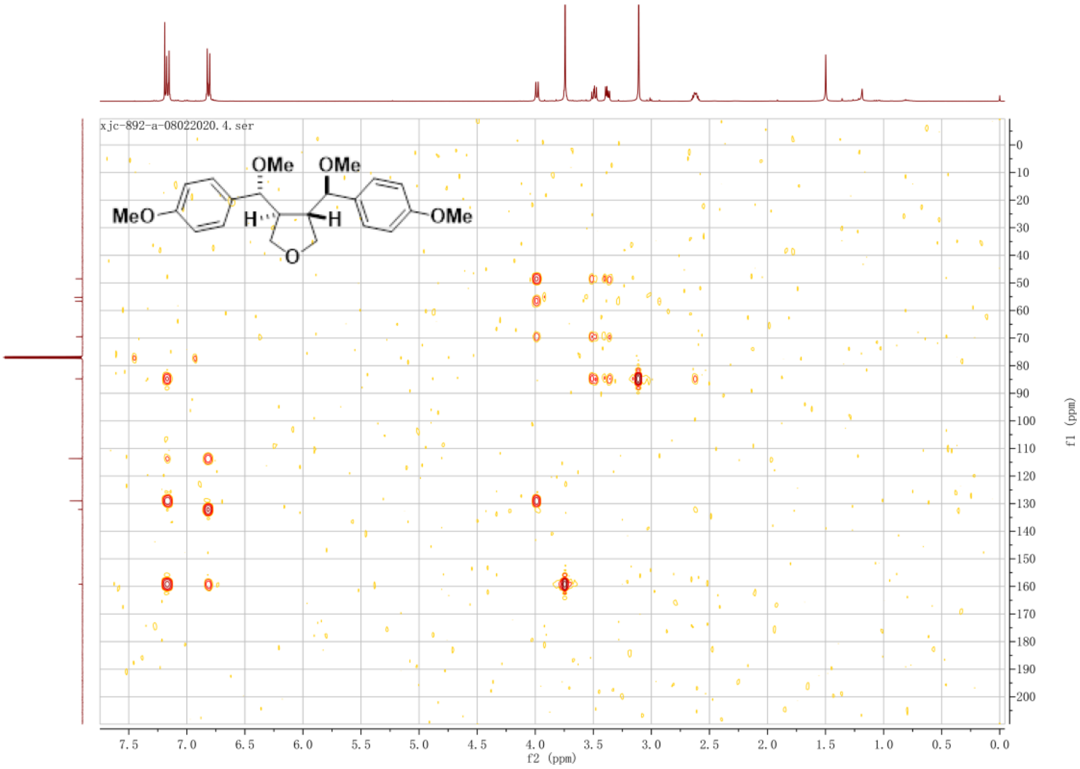

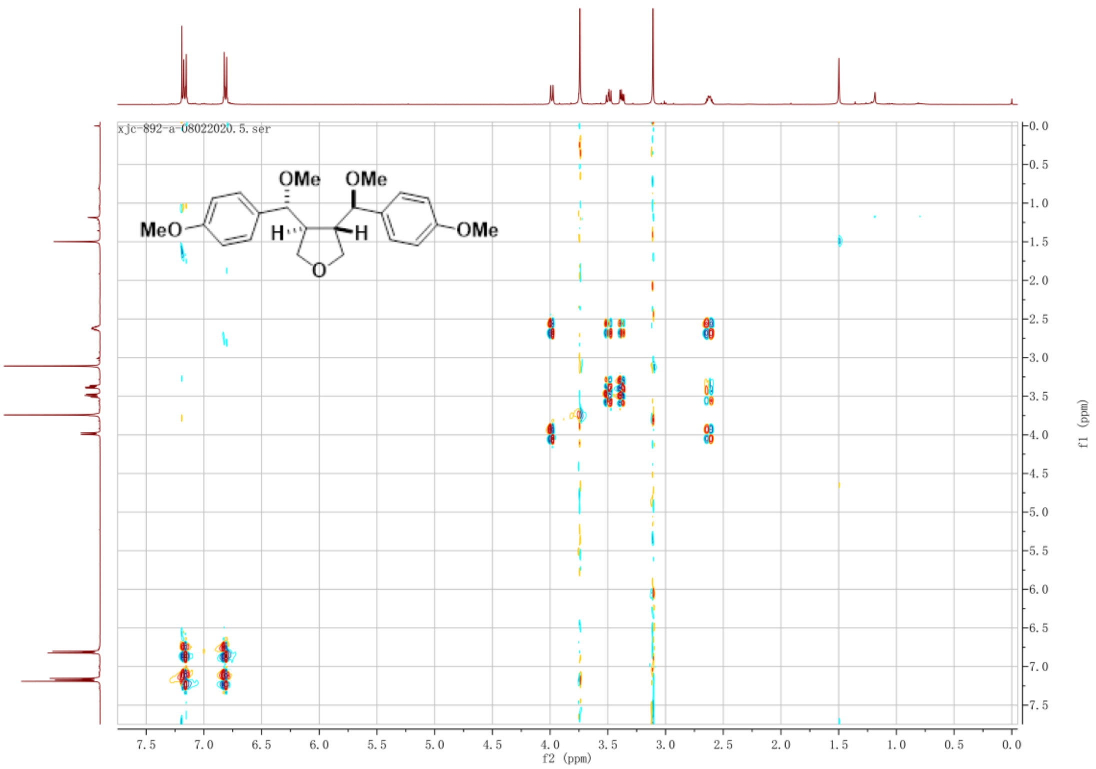
xjc-892-a-08022020.5.ser
OMe
OMe
MeO
H
H
O
OMe
f2 (ppm)
f1 (ppm)

Supplementary Figure 88. ^{1}H NMR spectrum of compound **22b-A** (400 MHz, $CDCl_3$)

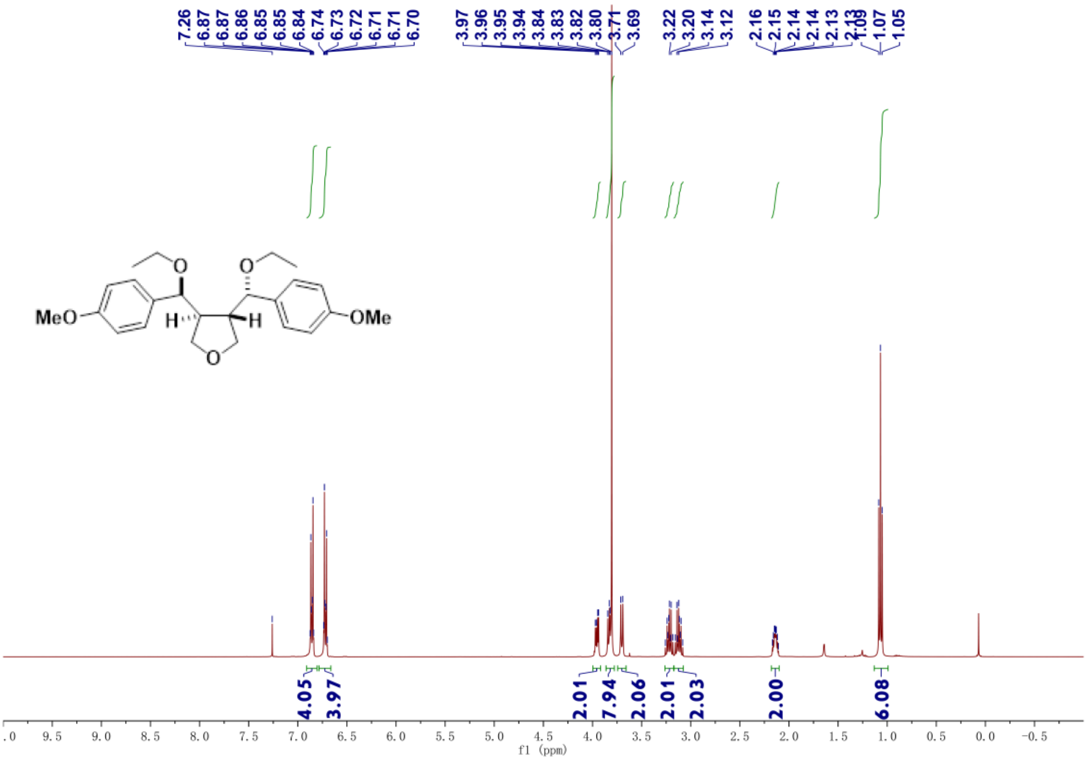

Supplementary Figure 89. ^{13}C NMR spectrum of compound **22b-A** (101 MHz, $CDCl_3$)

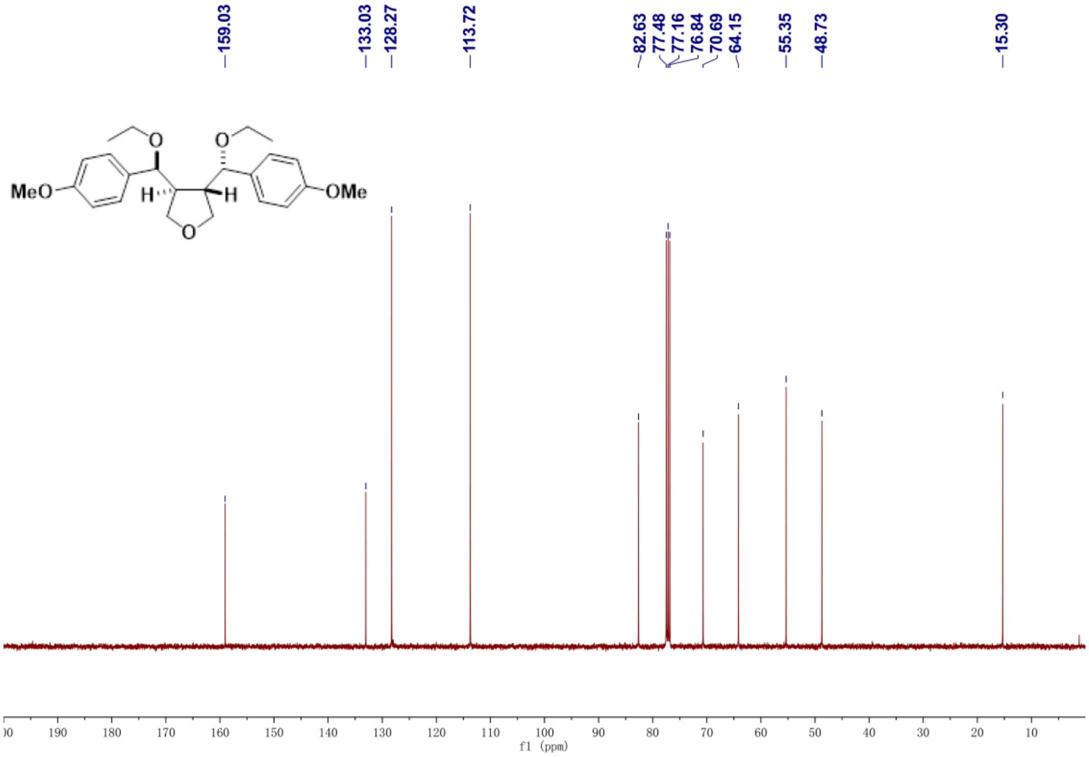

Supplementary Figure 90. ^{1}H NMR spectrum of compound **22b-B** (400 MHz, $CDCl_3$)

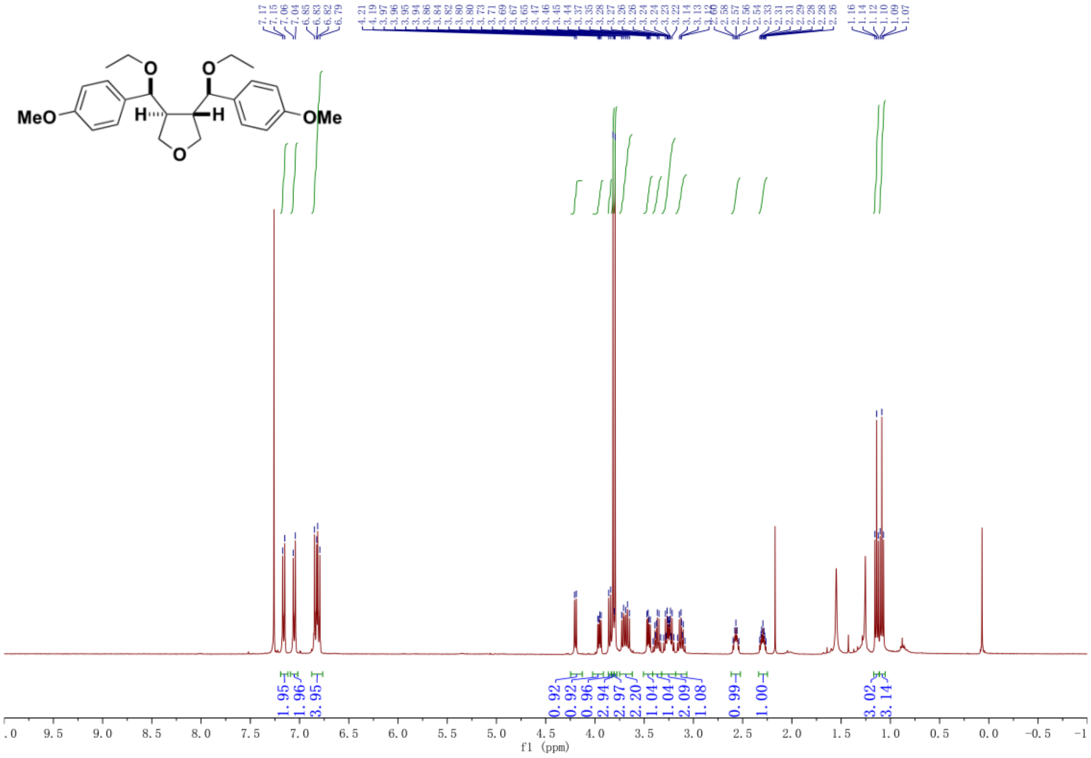

Supplementary Figure 91. ^{13}C NMR spectrum of compound **22b-B** (101 MHz, $CDCl_3$)

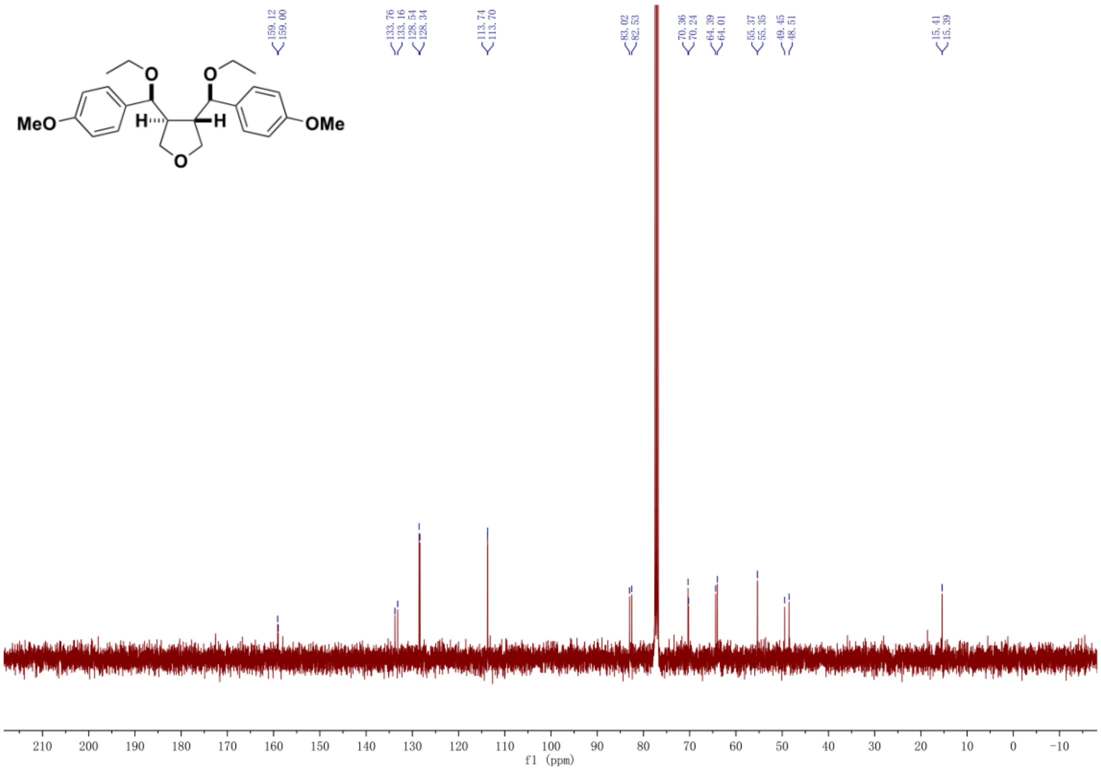

Supplementary Figure 92. ^{1}H NMR spectrum of compound **22c-A** (400 MHz, $CDCl_3$)

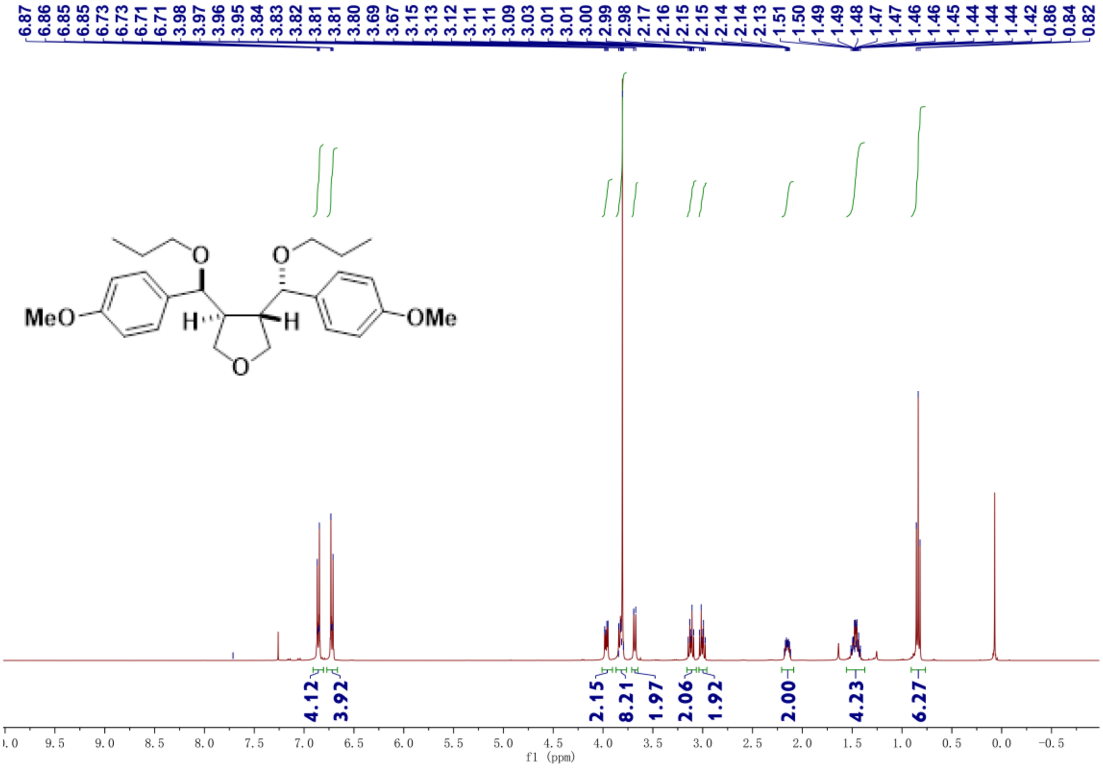

Supplementary Figure 93. ^{13}C NMR spectrum of compound **22c-A** (101 MHz, $CDCl_3$)

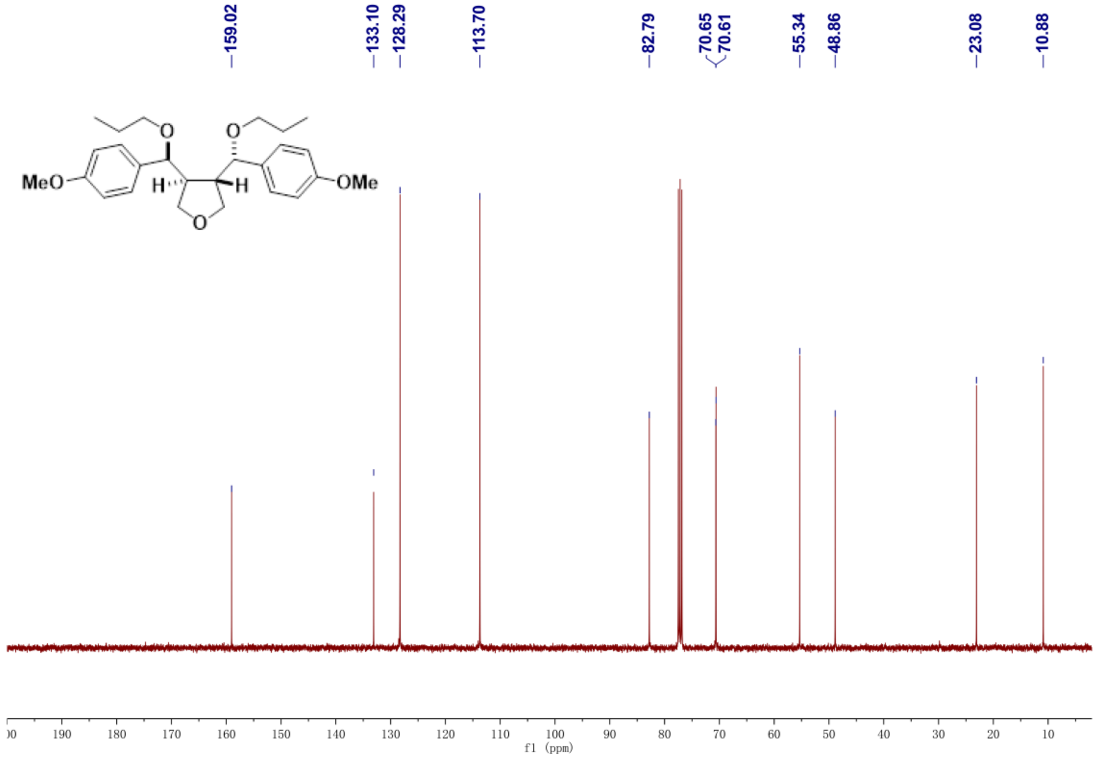

Supplementary Figure 94. ^{1}H NMR spectrum of compound **22d-A** (400 MHz, $CDCl_3$)

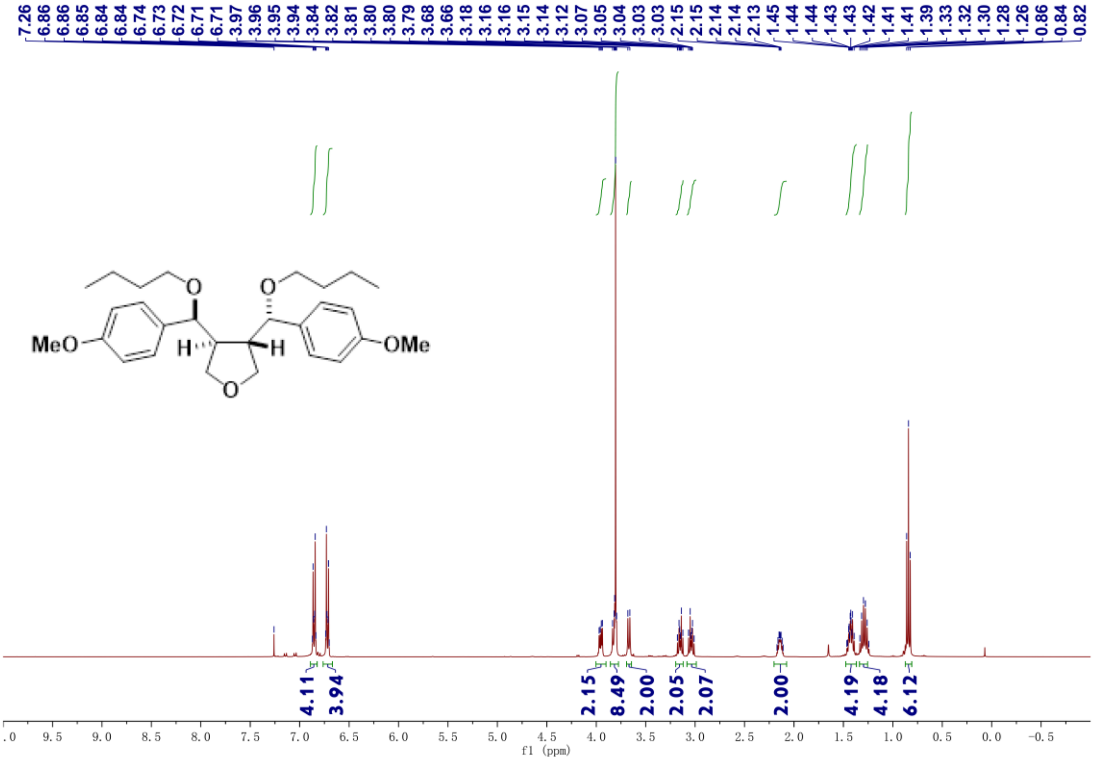

Supplementary Figure 95. ^{13}C NMR spectrum of compound **22d-A** (101 MHz, $CDCl_3$)

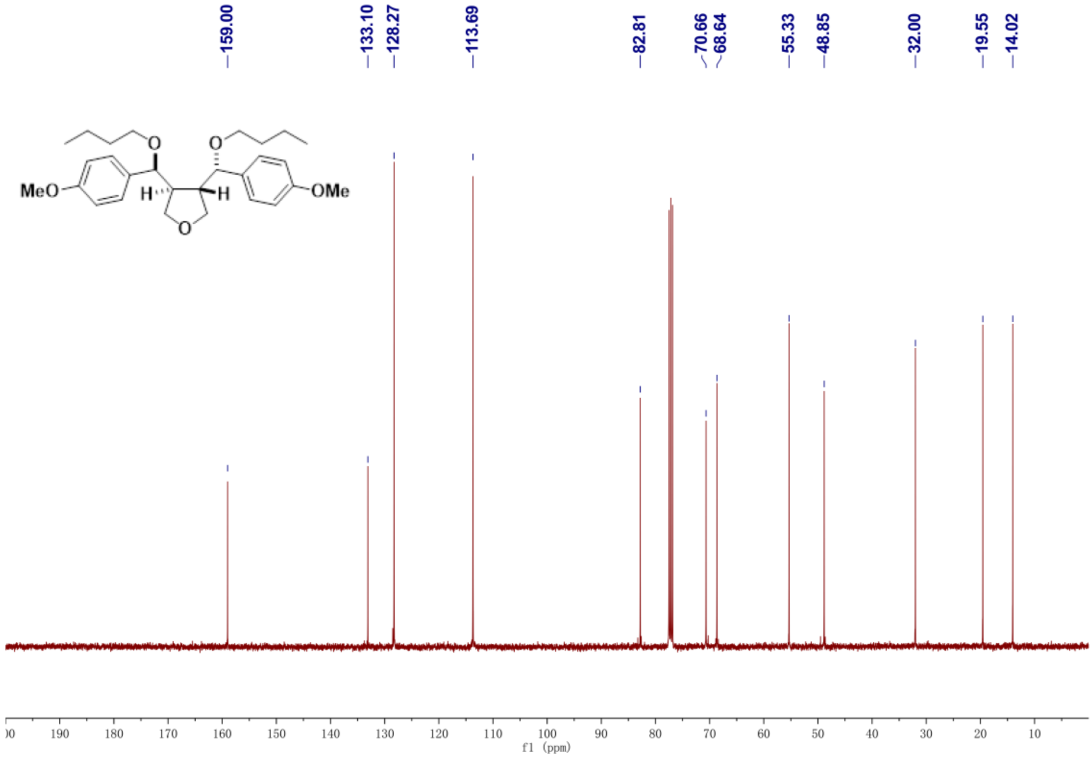

Supplementary Figure 96. ^{1}H NMR spectrum of compound **22e-A** (400 MHz, $CDCl_3$)

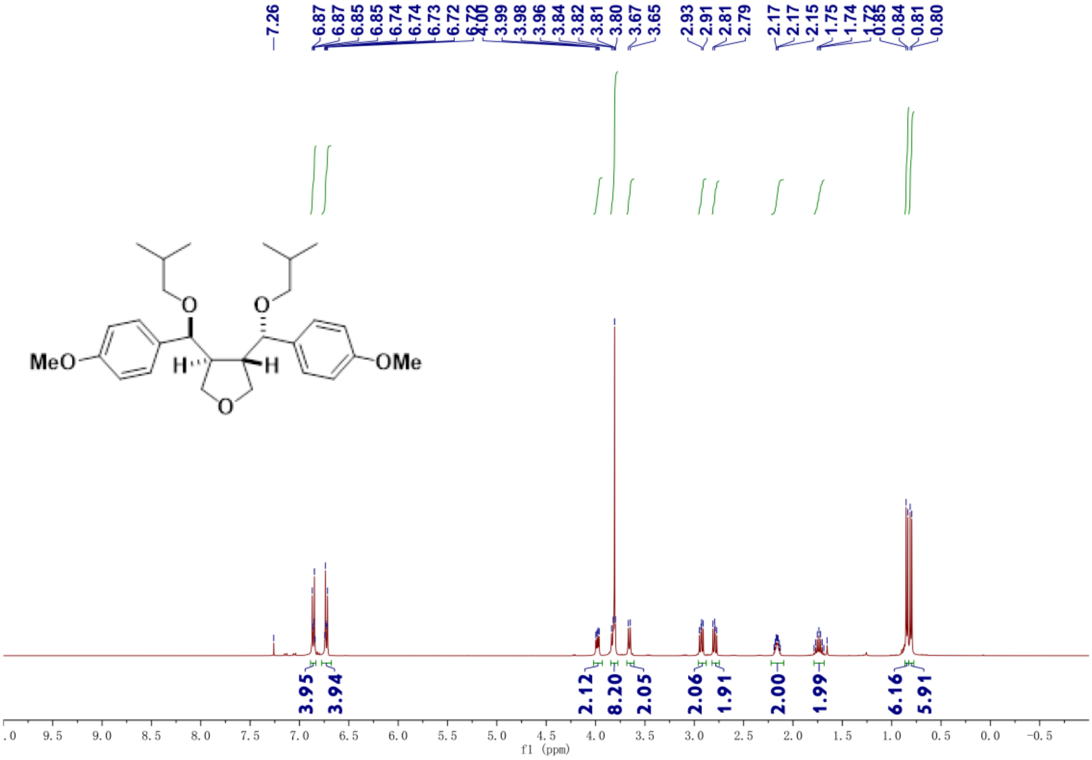

Supplementary Figure 97. ^{13}C NMR spectrum of compound **22e-A** (101 MHz, $CDCl_3$)

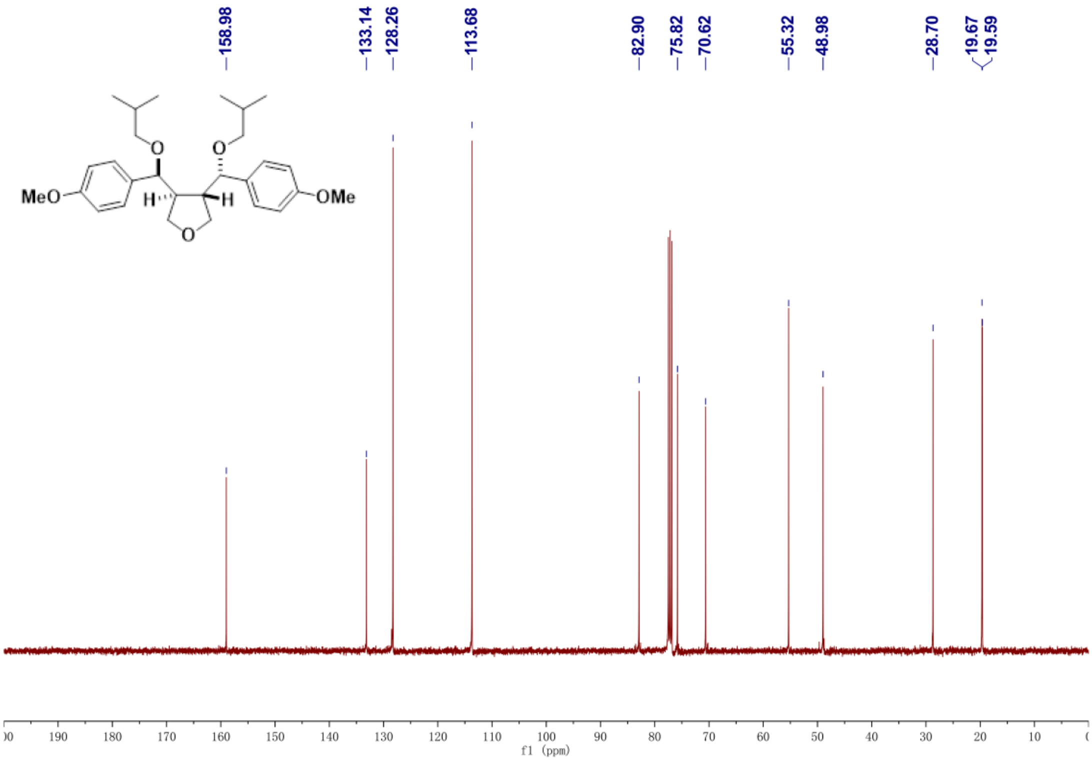

Supplementary Figure 98. ^{1}H NMR spectrum of compound **22f-A** (400 MHz, $CDCl_3$)

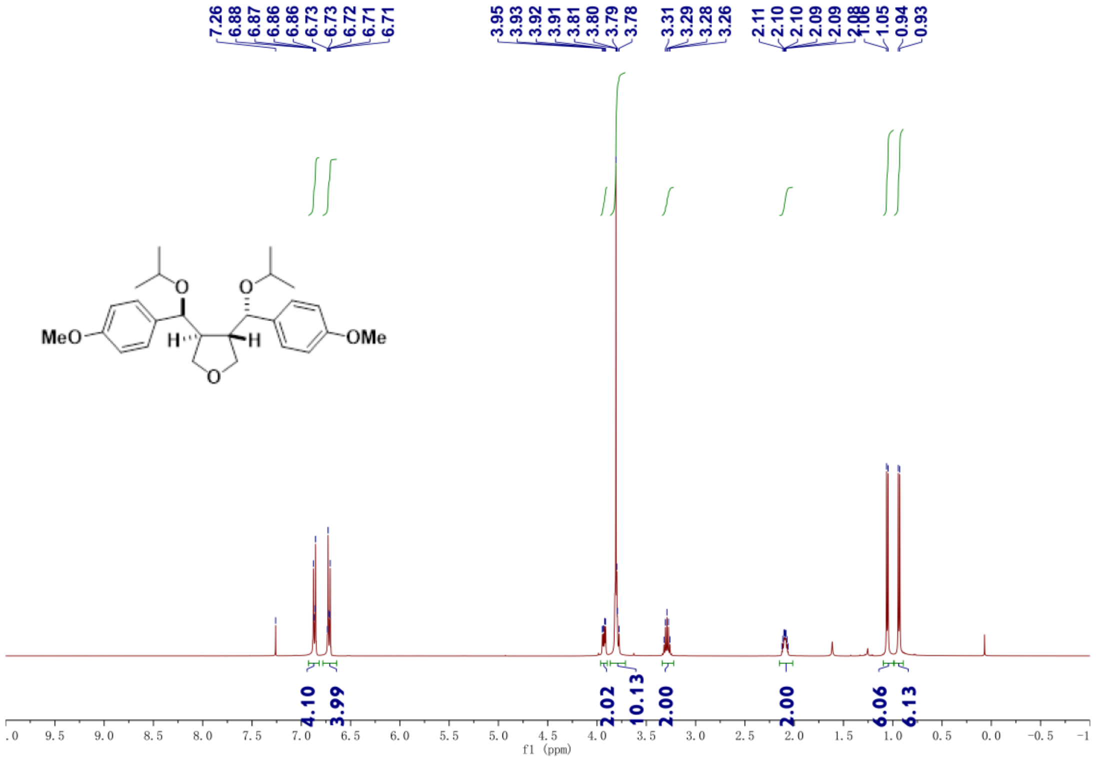

Supplementary Figure 99. ^{13}C NMR spectrum of compound **22f-A** (101 MHz, $CDCl_3$)

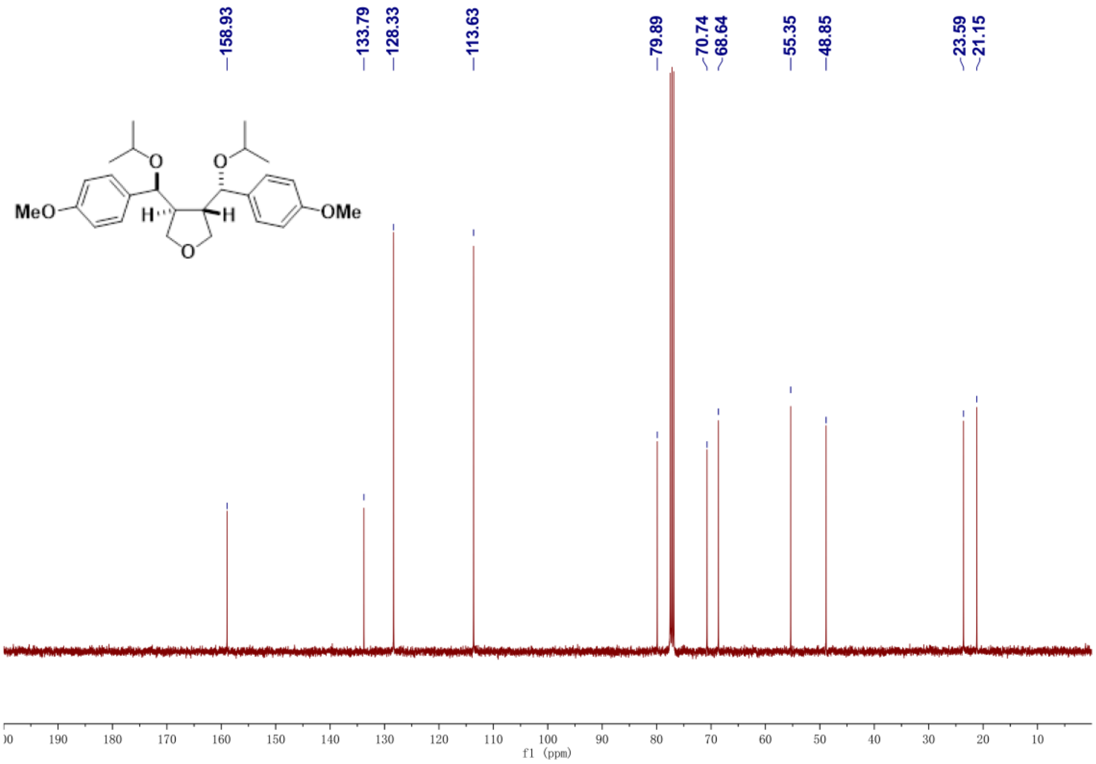

Supplementary Figure 100. ^{1}H NMR spectrum of compound **22g-A** (400 MHz, $CDCl_3$)

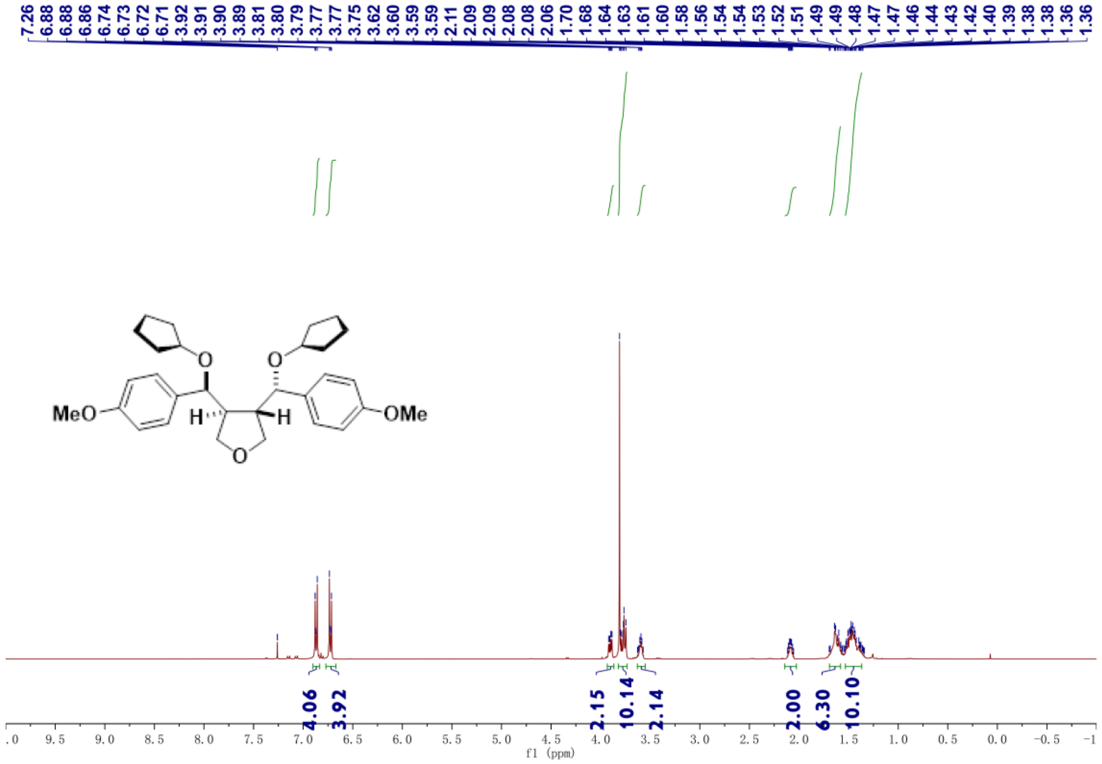

Supplementary Figure 101. ^{13}C NMR spectrum of compound **22g-A** (101 MHz, $CDCl_3$)

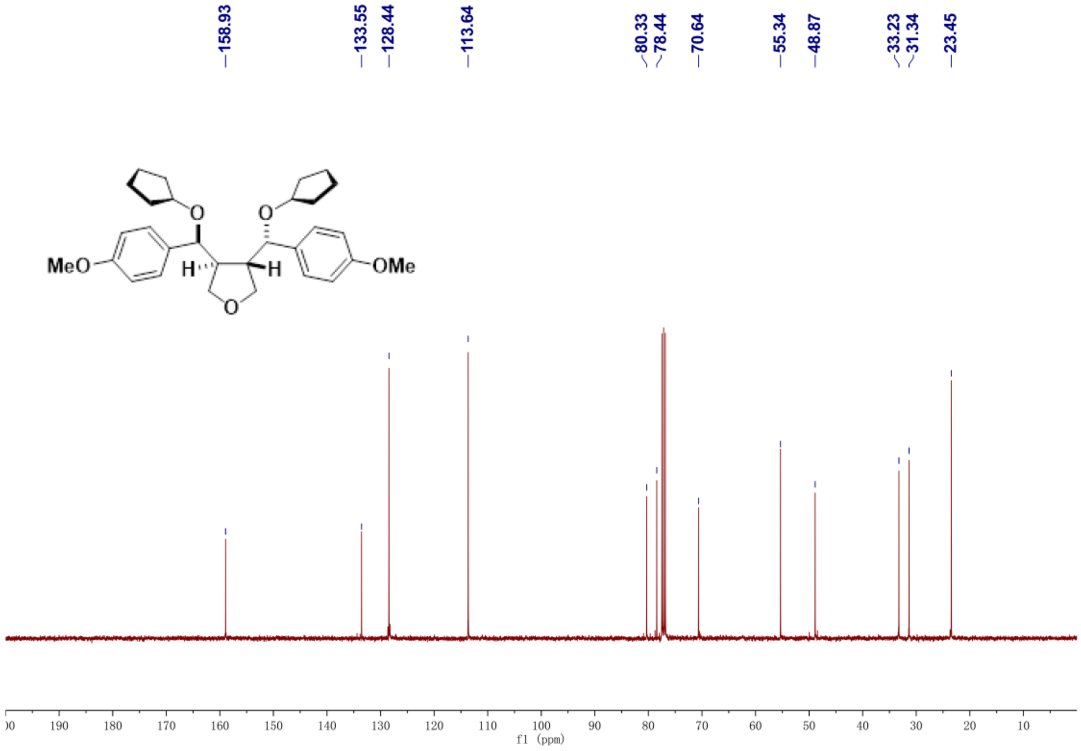

Supplementary Figure 102. ^{1}H NMR spectrum of compound **22h-A** (400 MHz, $CDCl_3$)

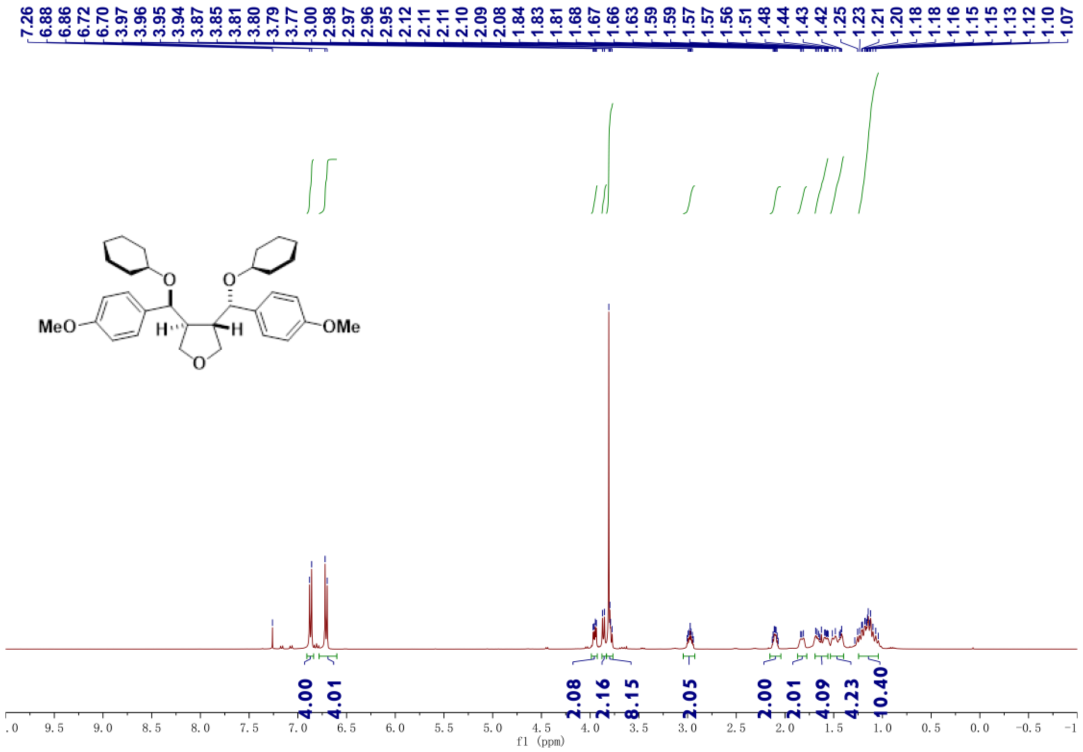

Supplementary Figure 103. ^{13}C NMR spectrum of compound **22h-A** (101 MHz, $CDCl_3$)

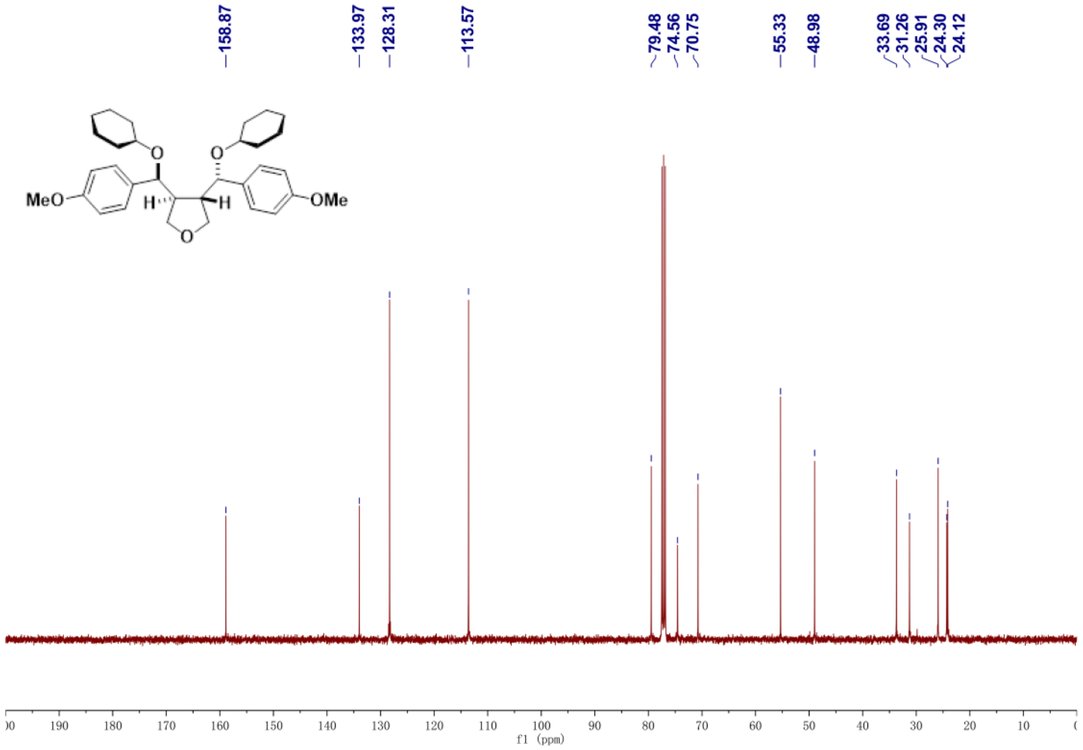

Supplementary Figure 104. ^{1}H NMR spectrum of compound **22i-A** (400 MHz, $CDCl_3$)

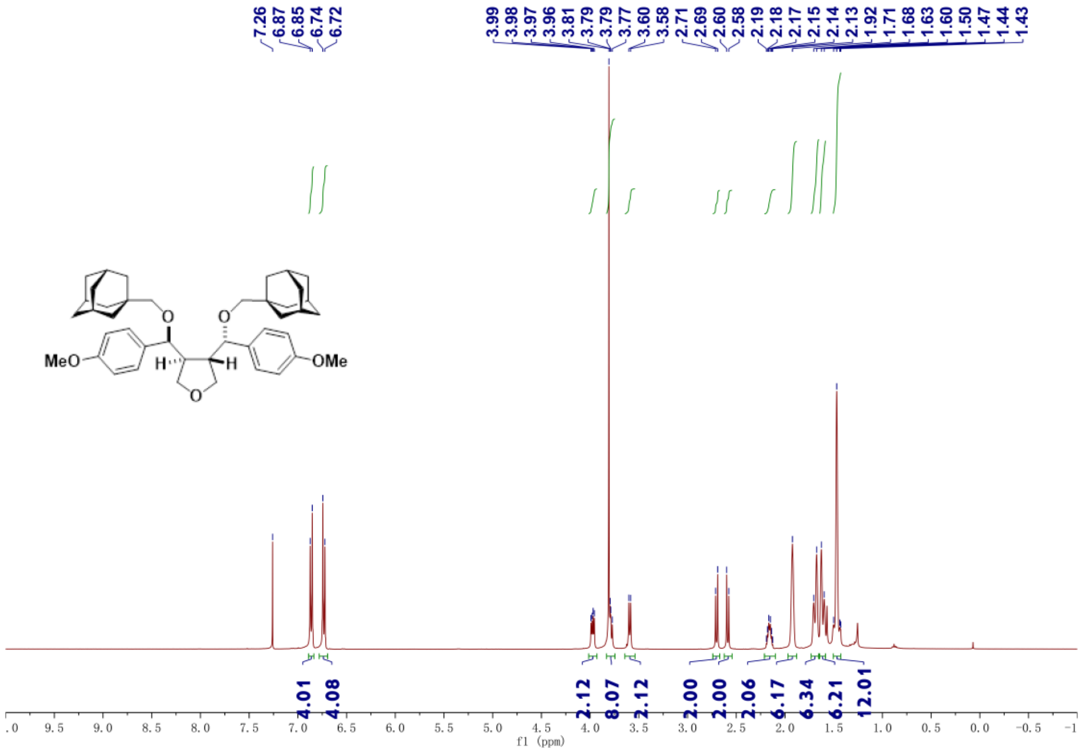

Supplementary Figure 105. ^{13}C NMR spectrum of compound **22i-A** (101 MHz, $CDCl_3$)

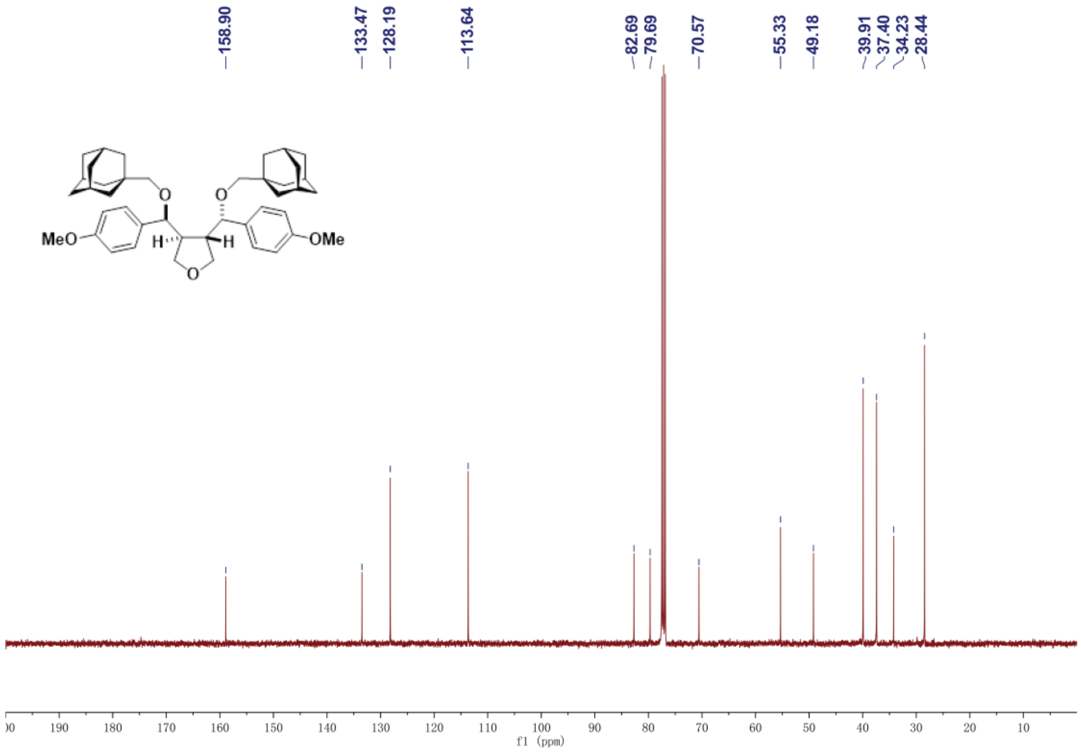

Supplementary Figure 106. ^{1}H NMR spectrum of compound **22j-A** (400 MHz, $CDCl_3$)

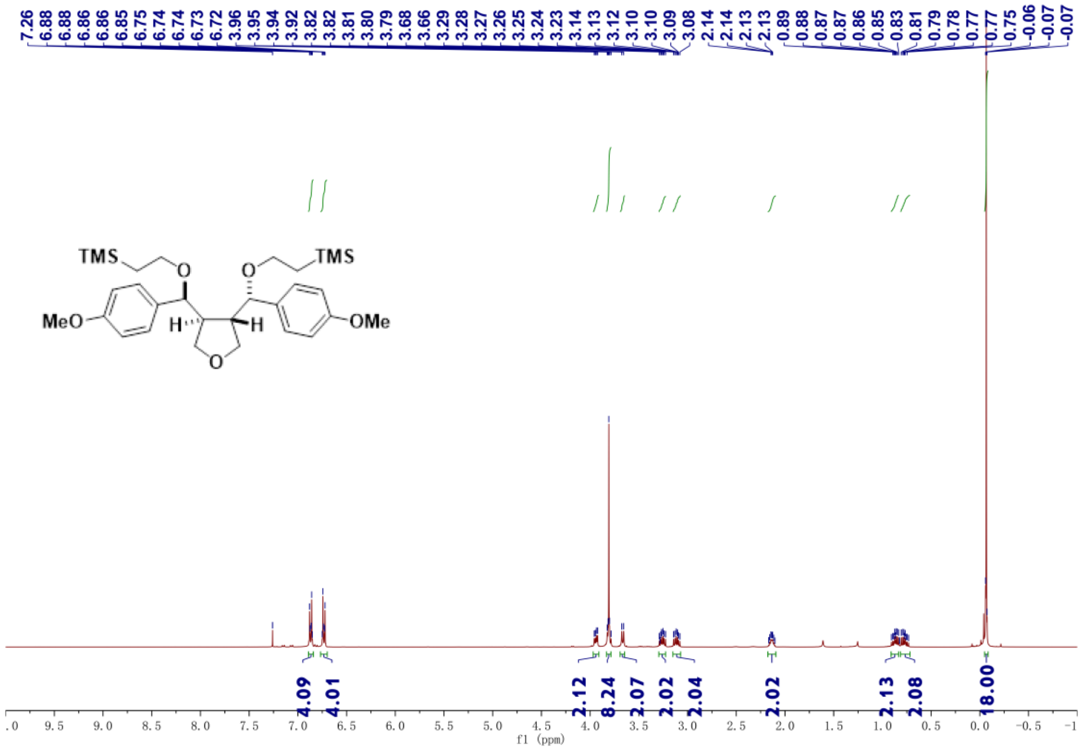

Supplementary Figure 107. ^{13}C NMR spectrum of compound **22j-A** (101 MHz, $CDCl_3$)

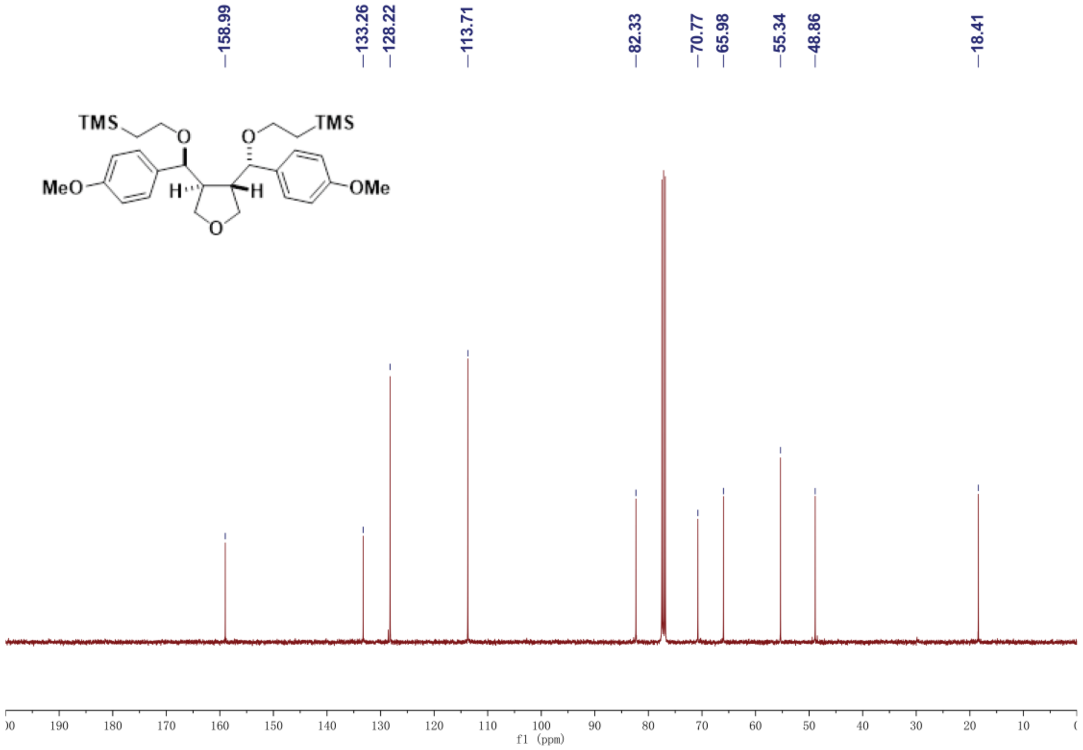

Supplementary Figure 108. ^{1}H NMR spectrum of compound **22k-A** (400 MHz, $CDCl_3$)

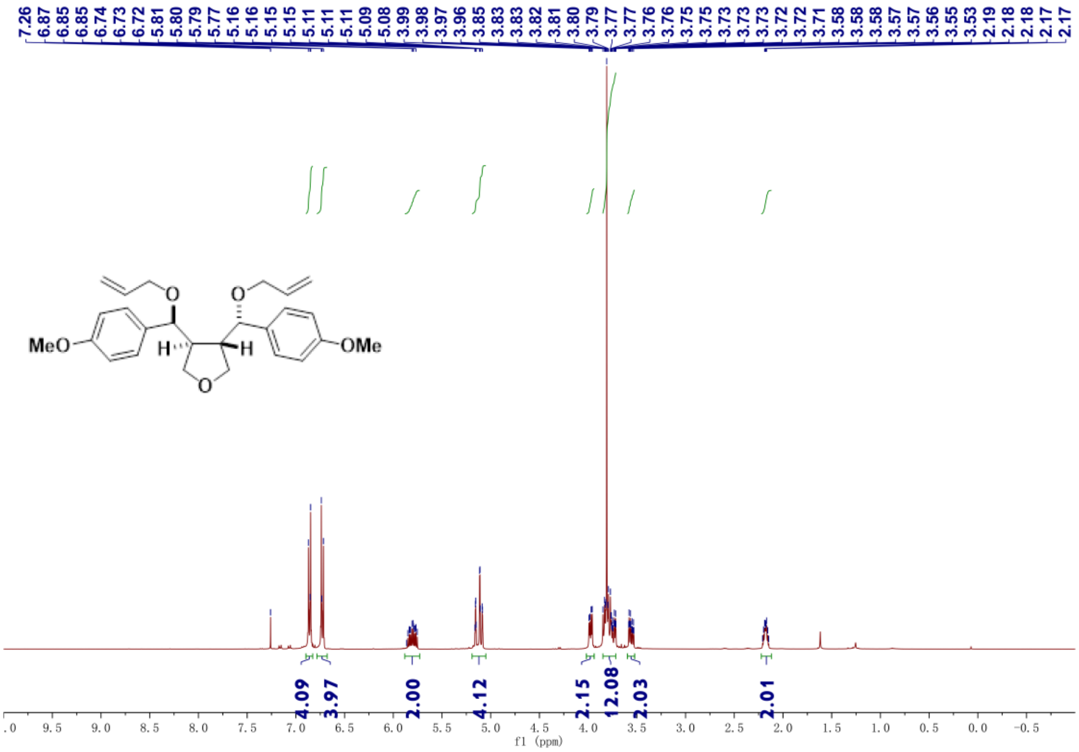

Supplementary Figure 109. ^{13}C NMR spectrum of compound **22k-A** (101 MHz, $CDCl_3$)

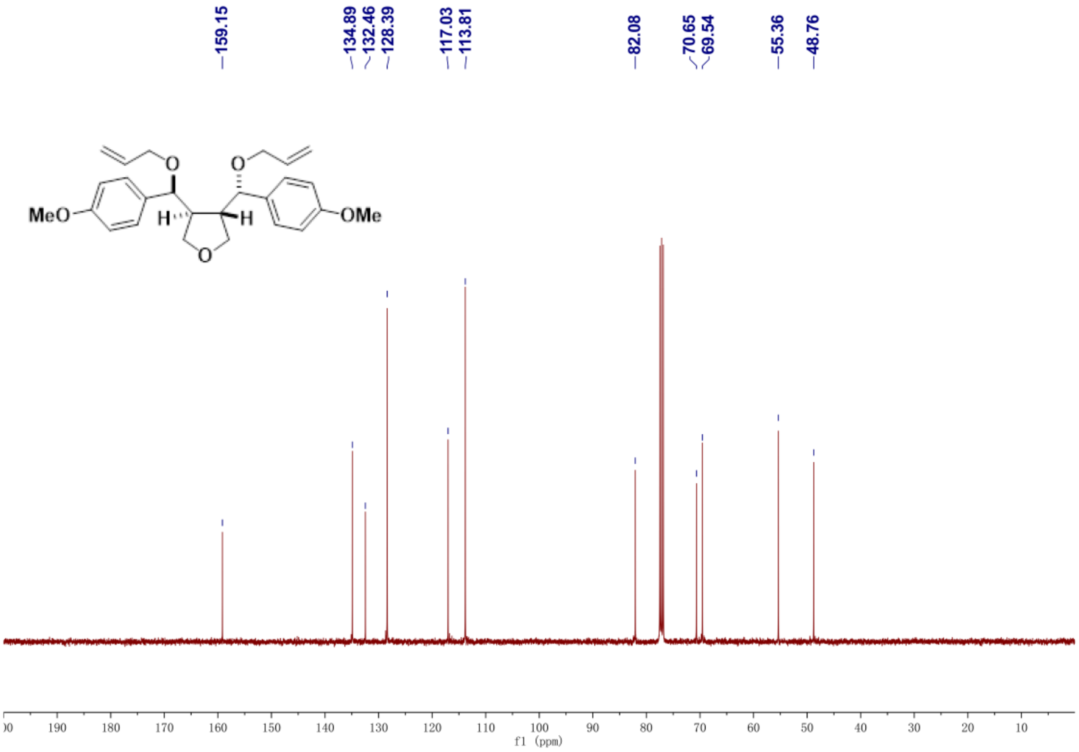

Supplementary Figure 110. ^{1}H NMR spectrum of compound **22l-A** (400 MHz, $CDCl_3$)

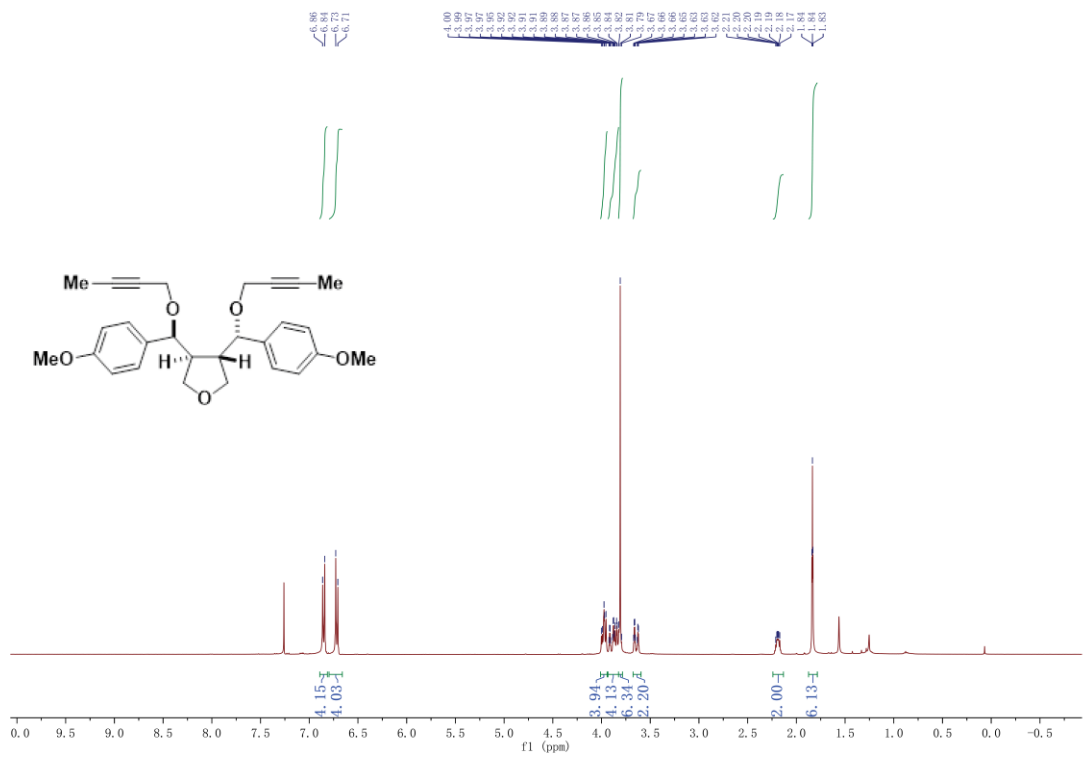

Supplementary Figure 111. ^{13}C NMR spectrum of compound **22l-A** (101 MHz, $CDCl_3$)

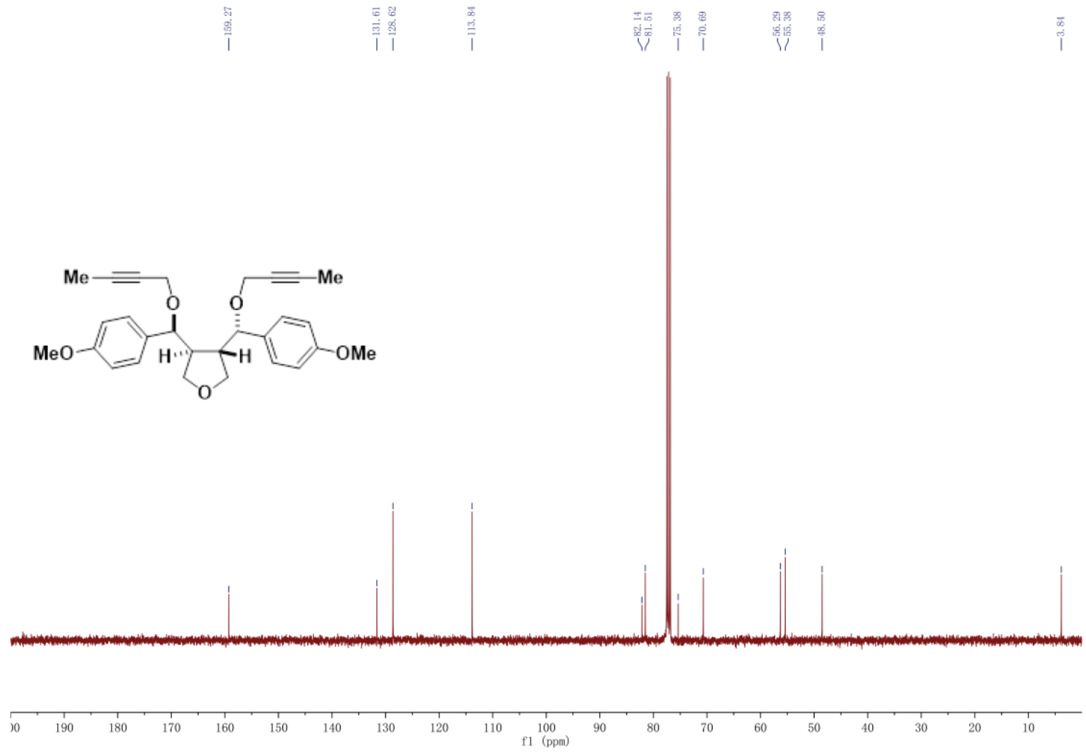

Supplementary Figure 112. ^{1}H NMR spectrum of compound **22m** (400 MHz, $CDCl_3$)

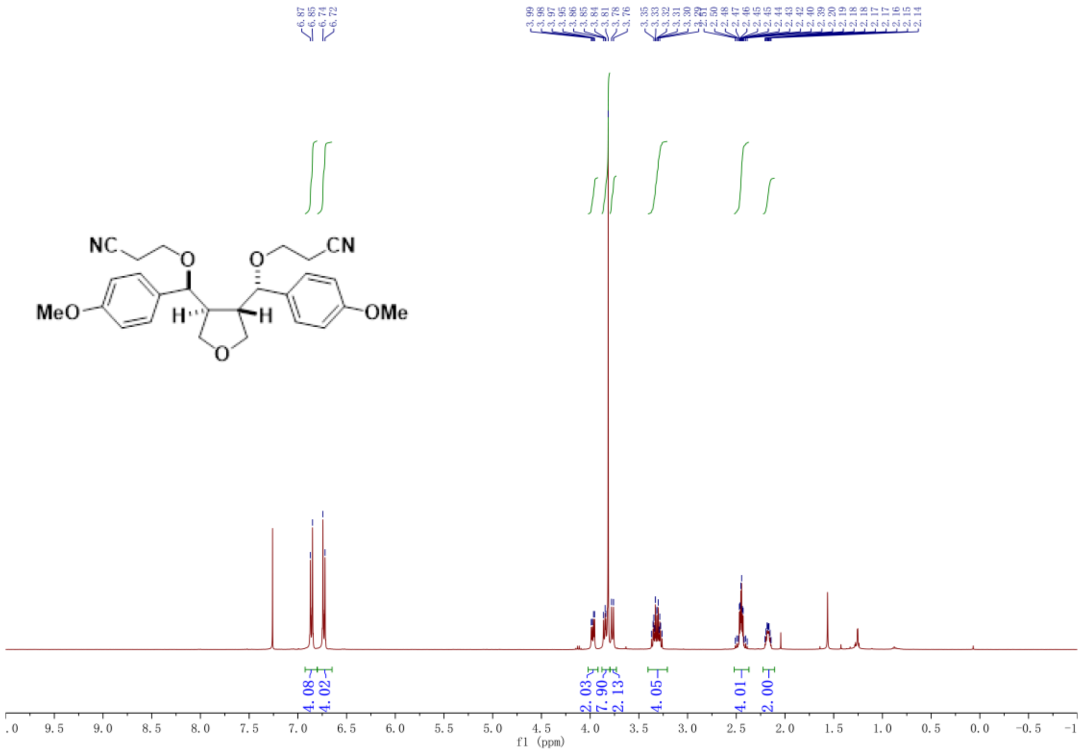

Supplementary Figure 113. ^{13}C NMR spectrum of compound **22m** (101 MHz, $CDCl_3$)

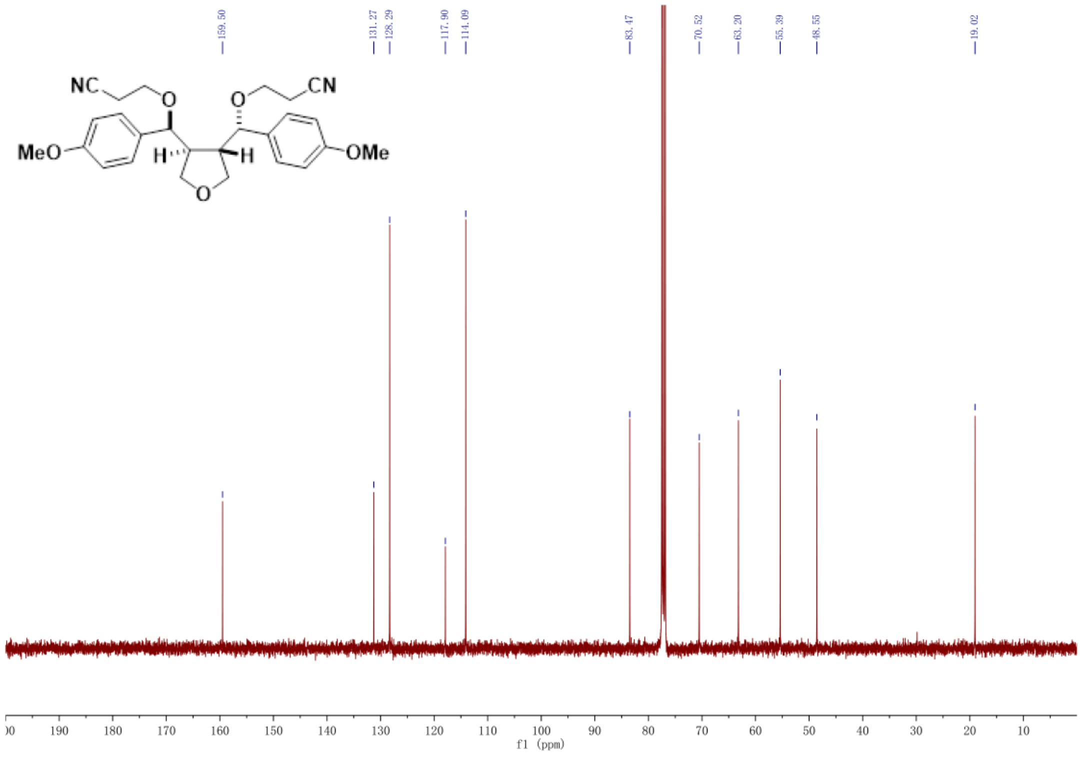

Supplementary Figure 114. ^{1}H NMR spectrum of compound **22n** (400 MHz, $CDCl_3$)

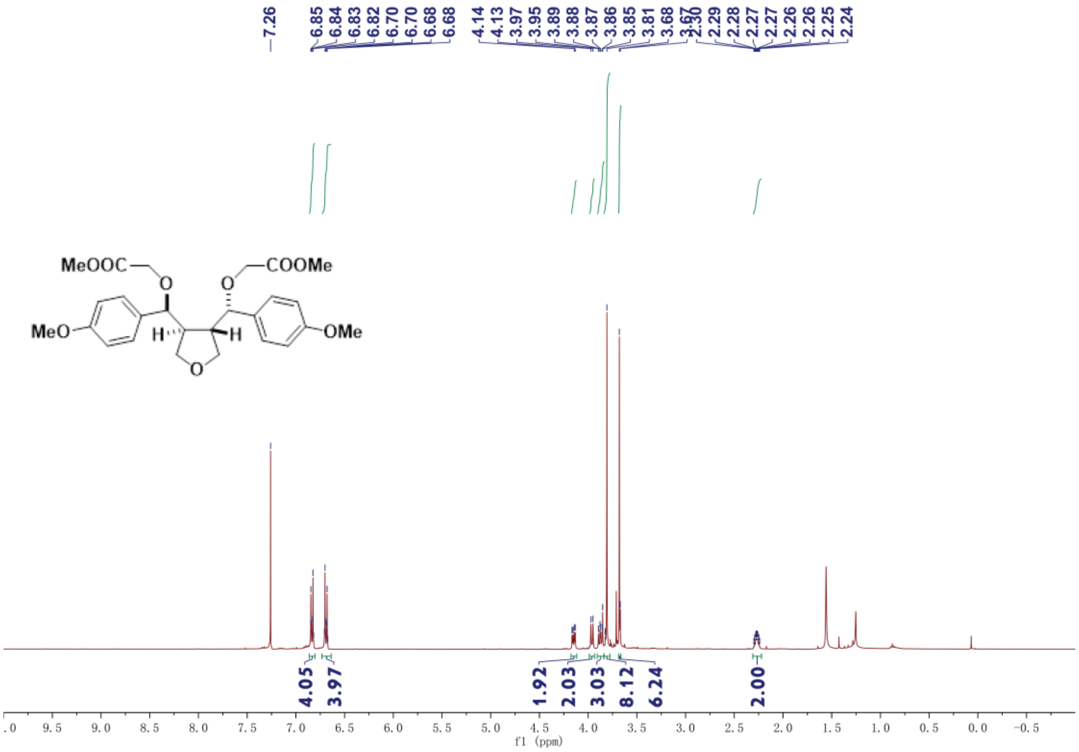

Supplementary Figure 115. ^{13}C NMR spectrum of compound **22n** (101 MHz, $CDCl_3$)

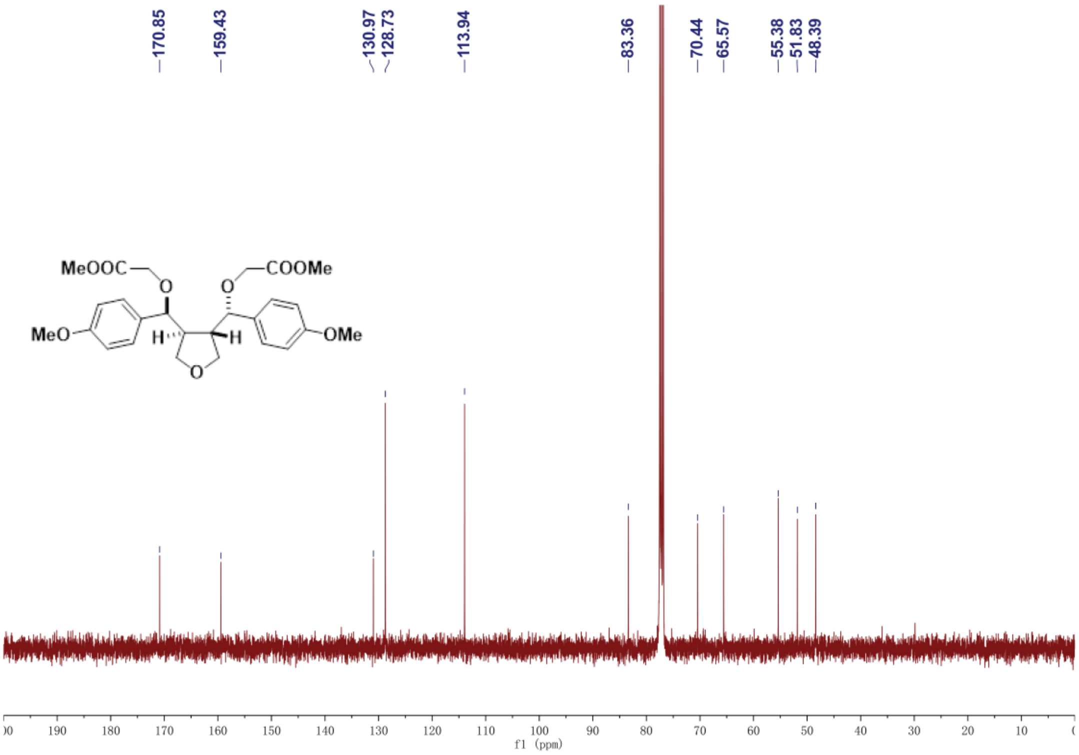

Supplementary Figure 116. ^{1}H NMR spectrum of compound **22o-A** (400 MHz, $CDCl_3$)

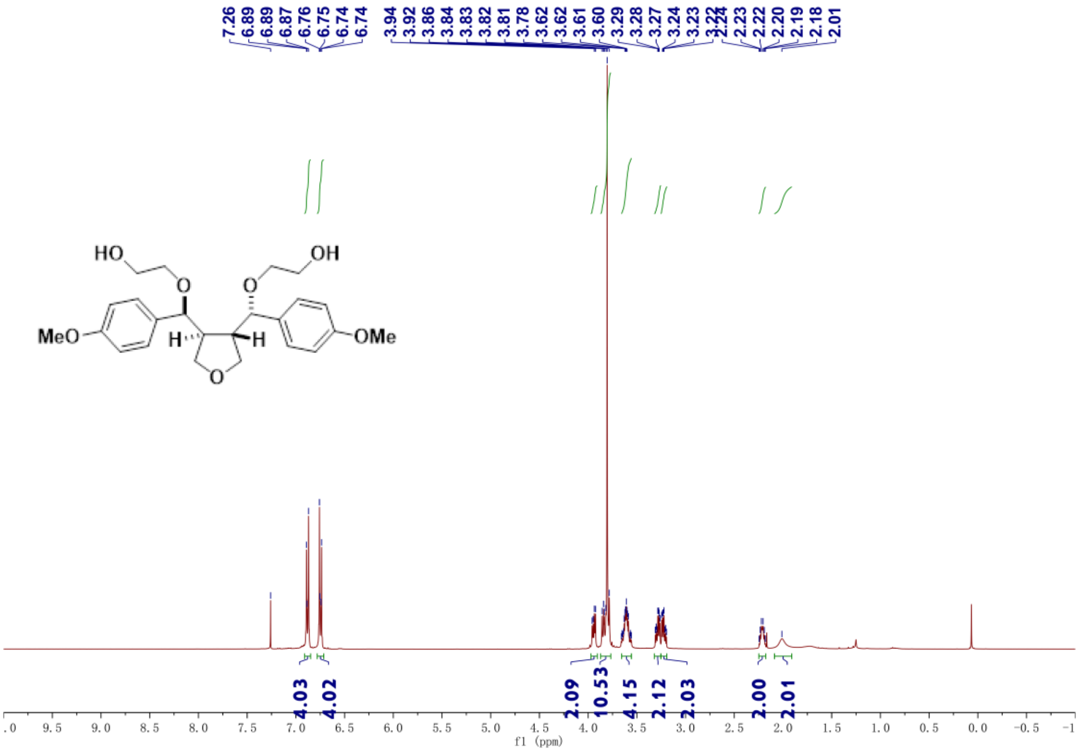

Supplementary Figure 117. ^{13}C NMR spectrum of compound **22o-A** (101 MHz, $CDCl_3$)

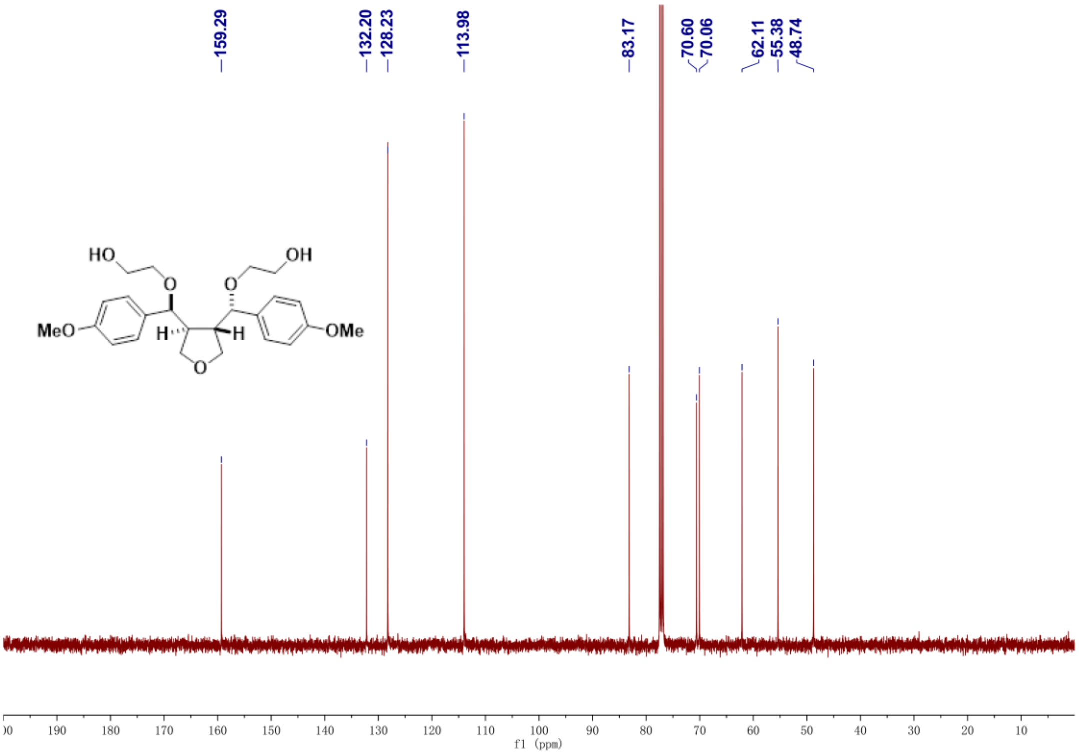

Supplementary Figure 118. ^{1}H NMR spectrum of compound **22p-A** (400 MHz, $CDCl_3$)

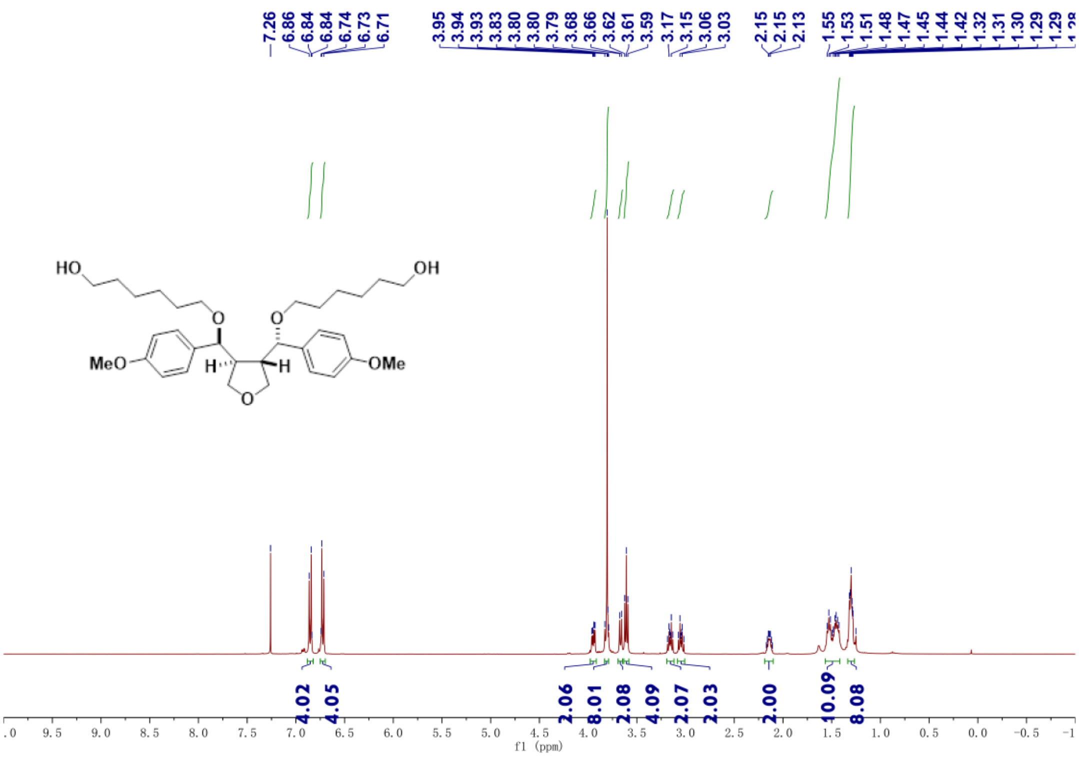

Supplementary Figure 119. ^{13}C NMR spectrum of compound **22p-A** (101 MHz, $CDCl_3$)

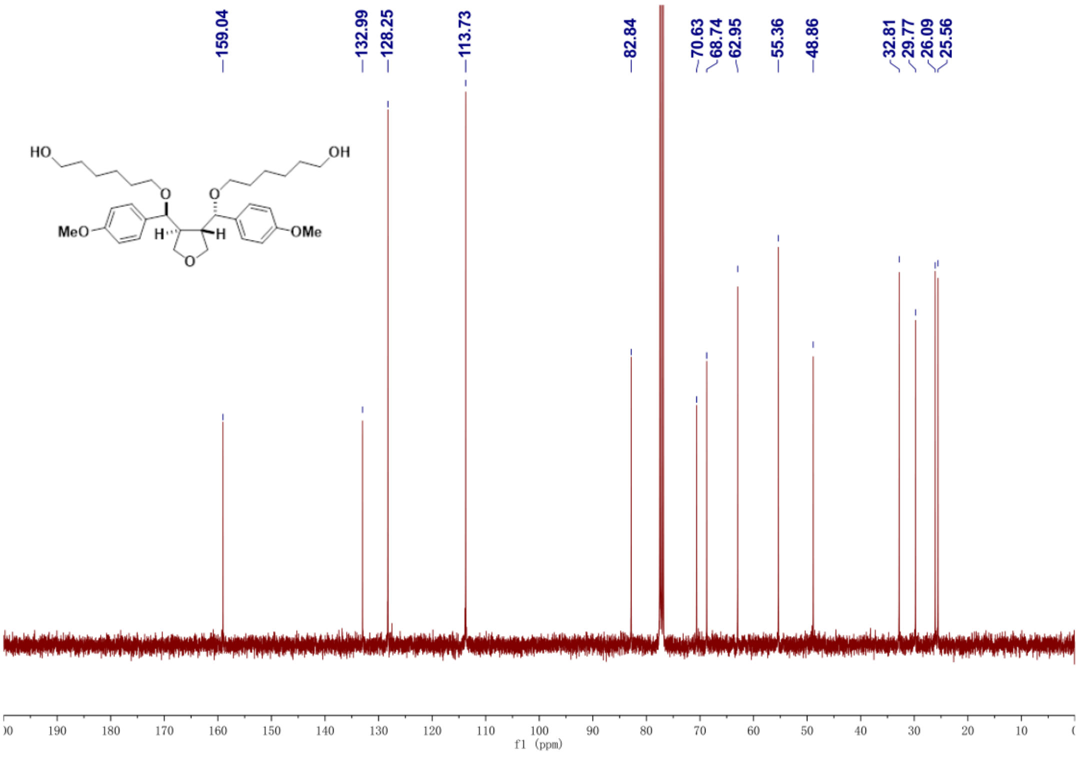

Supplementary Figure 120. ^{1}H NMR spectrum of compound **22q (3 diastereomers)** (400 MHz, $CDCl_3$)

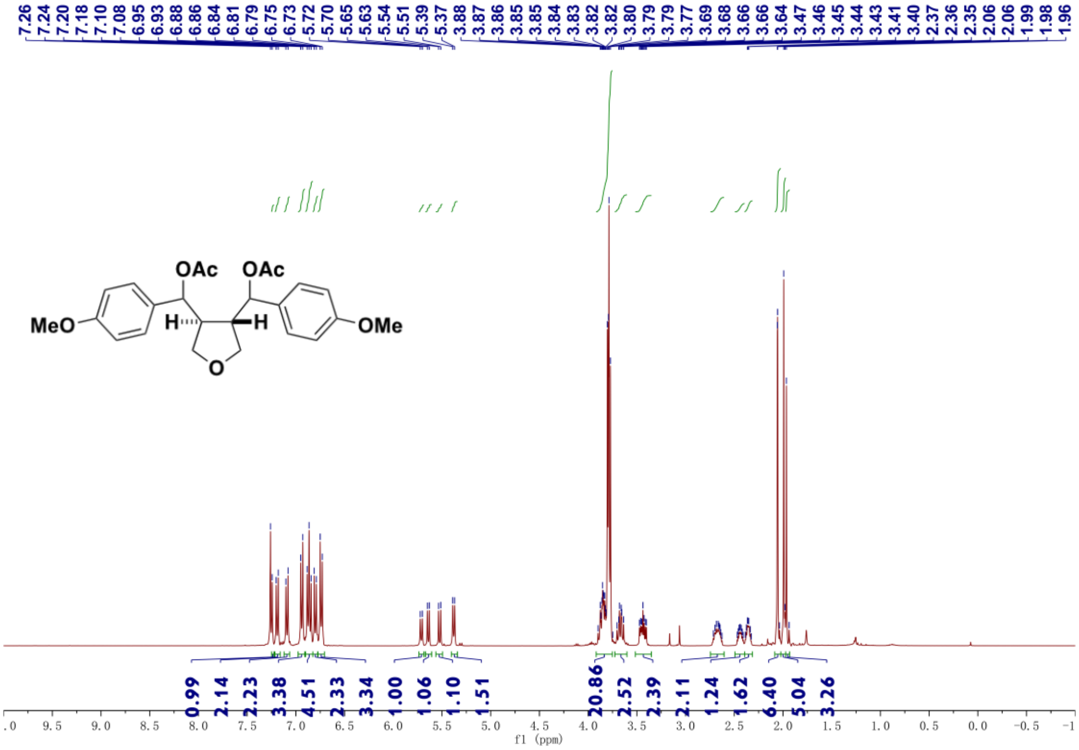

Supplementary Figure 121. ^{13}C NMR spectrum of compound **22q (3 diastereomers)** (101 MHz, $CDCl_3$)

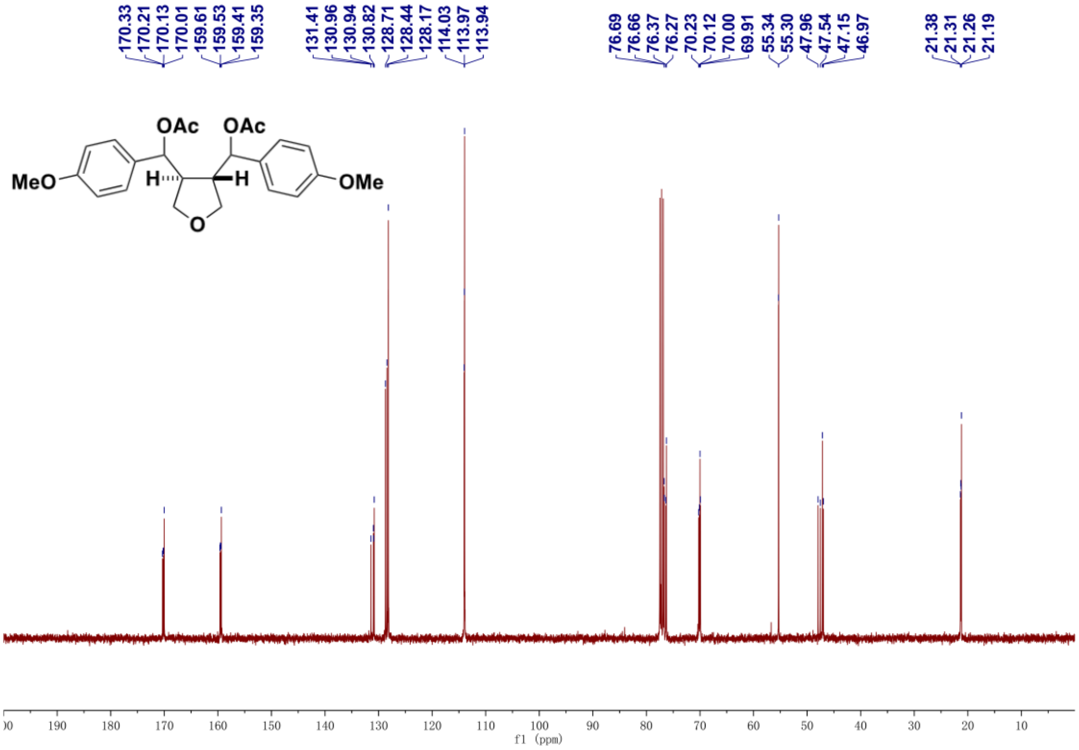

Supplementary Figure 122. ^{1}H NMR spectra of compound **22q (3 diastereomers)** (400 MHz, $CDCl_3$)
Top: 22q-A: **22q-B** = 4:1 dr mixture, isolated from photoredox reaction.
Bottom: **22q-A**: **22q-B** : **22q-C** = 1.5 : 2 : 1, prepared from **S4.1**.

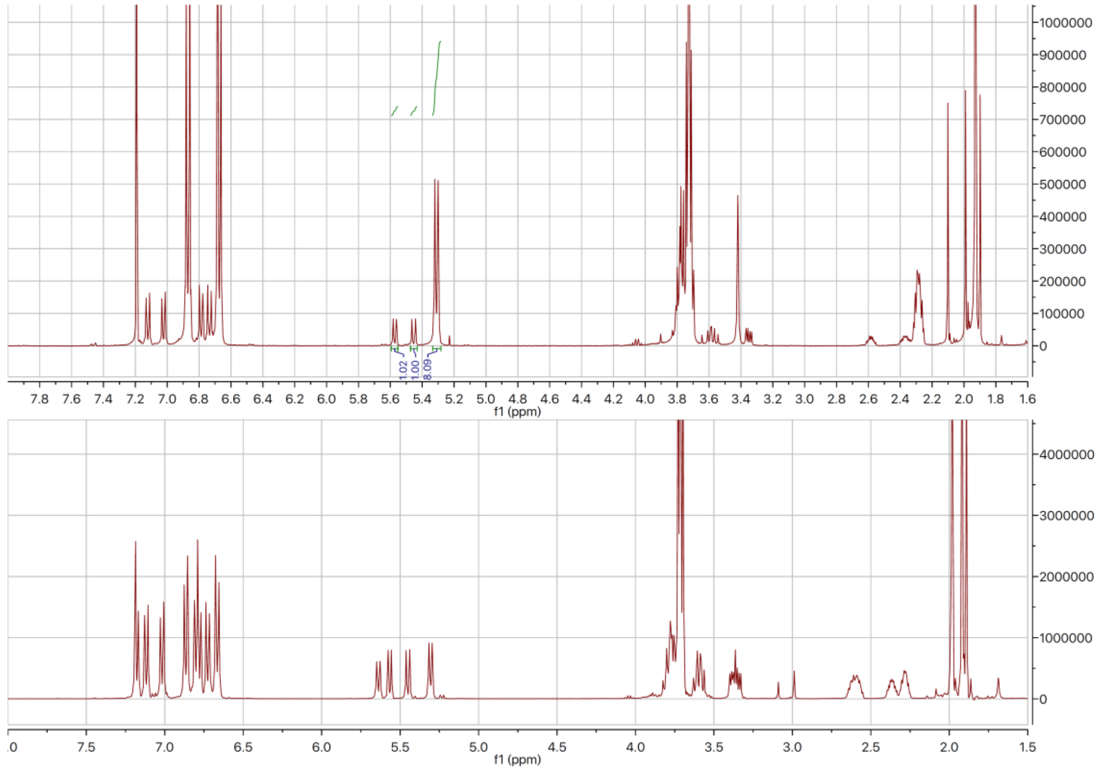

Supplementary Figure 123. ^{1}H NMR spectrum of compound **22r-A** (400 MHz, $CDCl_3$)

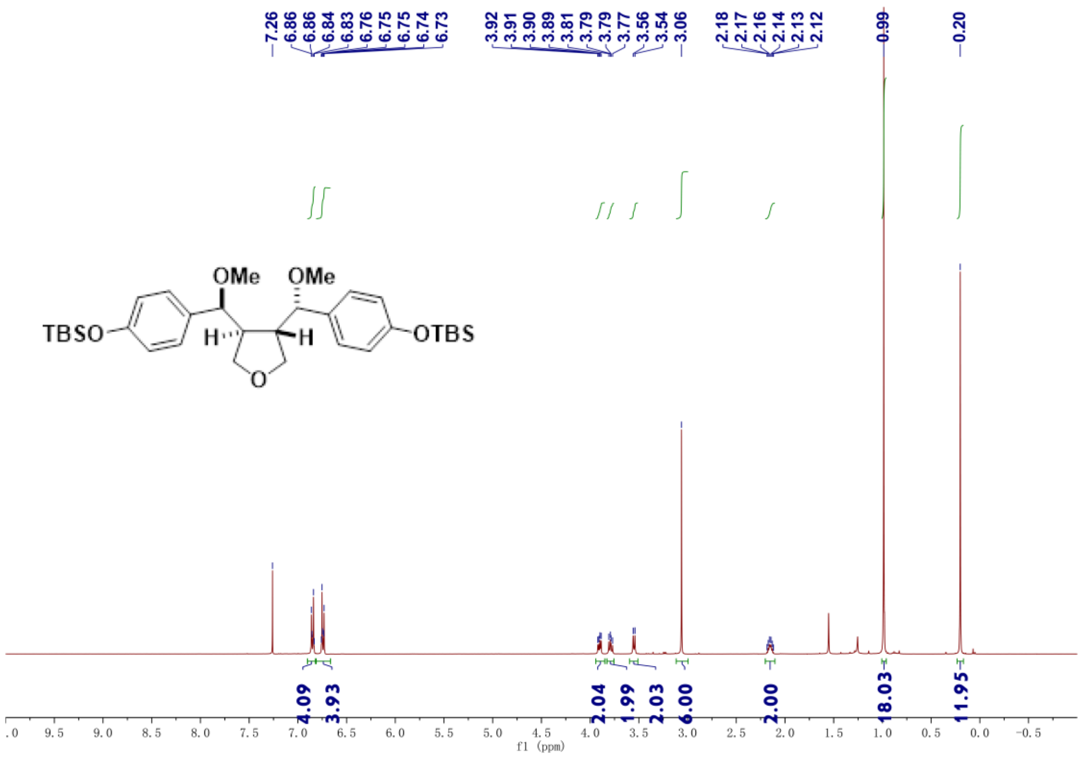

Supplementary Figure 124. ^{13}C NMR spectrum of compound **22r-A** (101 MHz, $CDCl_3$)

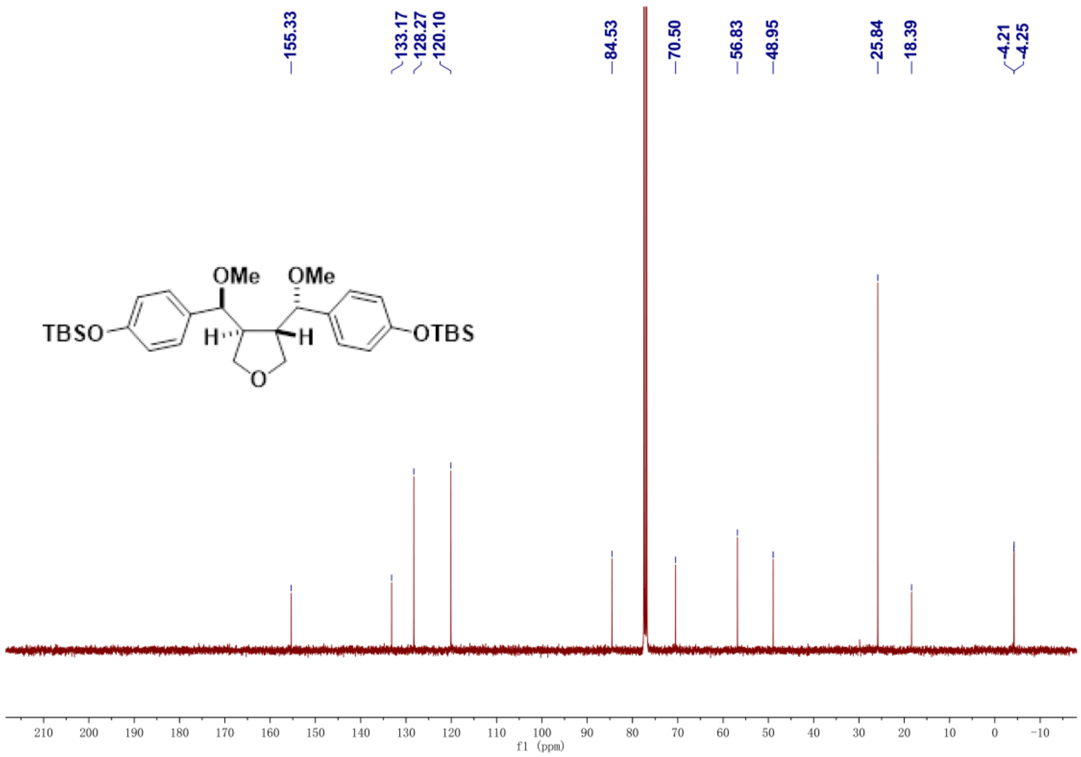

Supplementary Figure 125. ^{1}H NMR spectrum of compound **22s-A** (400 MHz, $CDCl_3$)

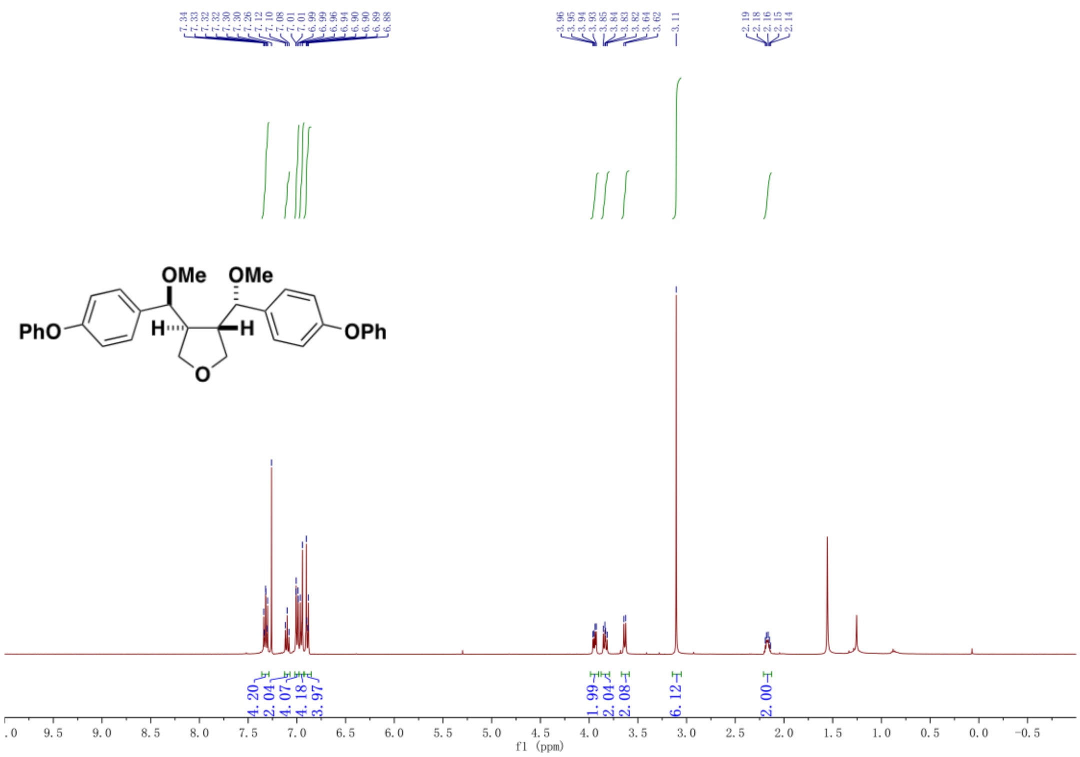

Supplementary Figure 126. ^{13}C NMR spectrum of compound **22s-A** (101 MHz, $CDCl_3$)

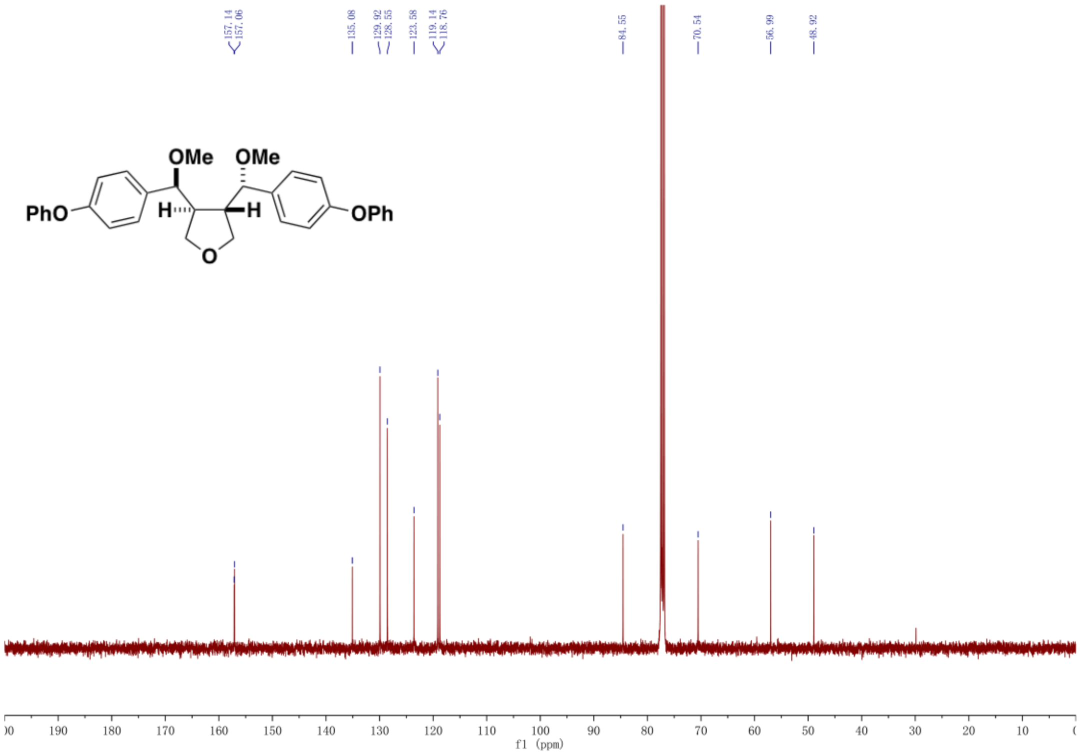

Supplementary Figure 127. ^{1}H NMR spectrum of compound **22t-A** (400 MHz, $CDCl_3$)

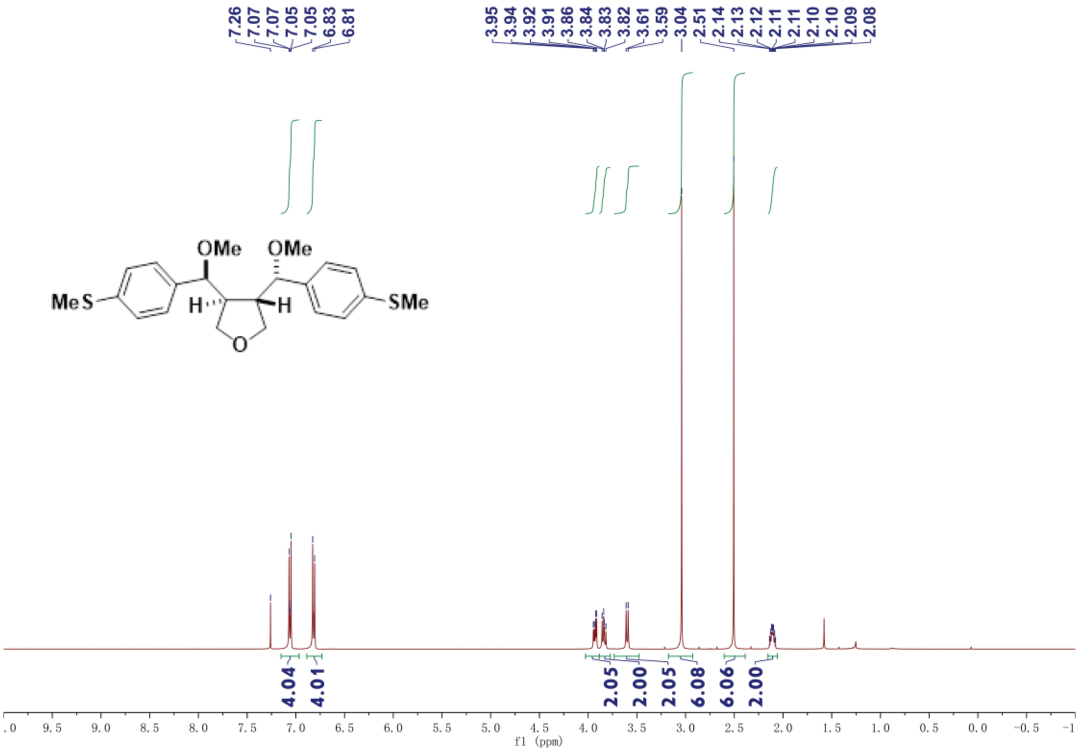

Supplementary Figure 128. ^{13}C NMR spectrum of compound **22t-A** (101 MHz, $CDCl_3$)

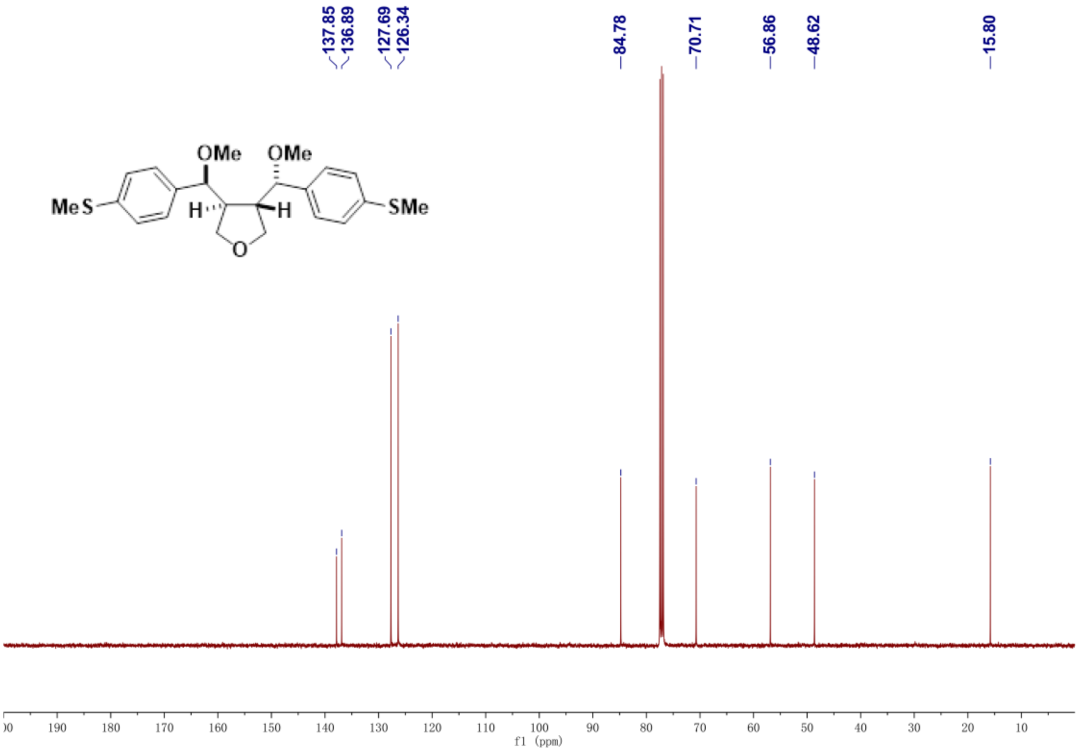

Supplementary Figure 129. ^{1}H NMR spectrum of compound **22u-A** (400 MHz, $CDCl_3$)

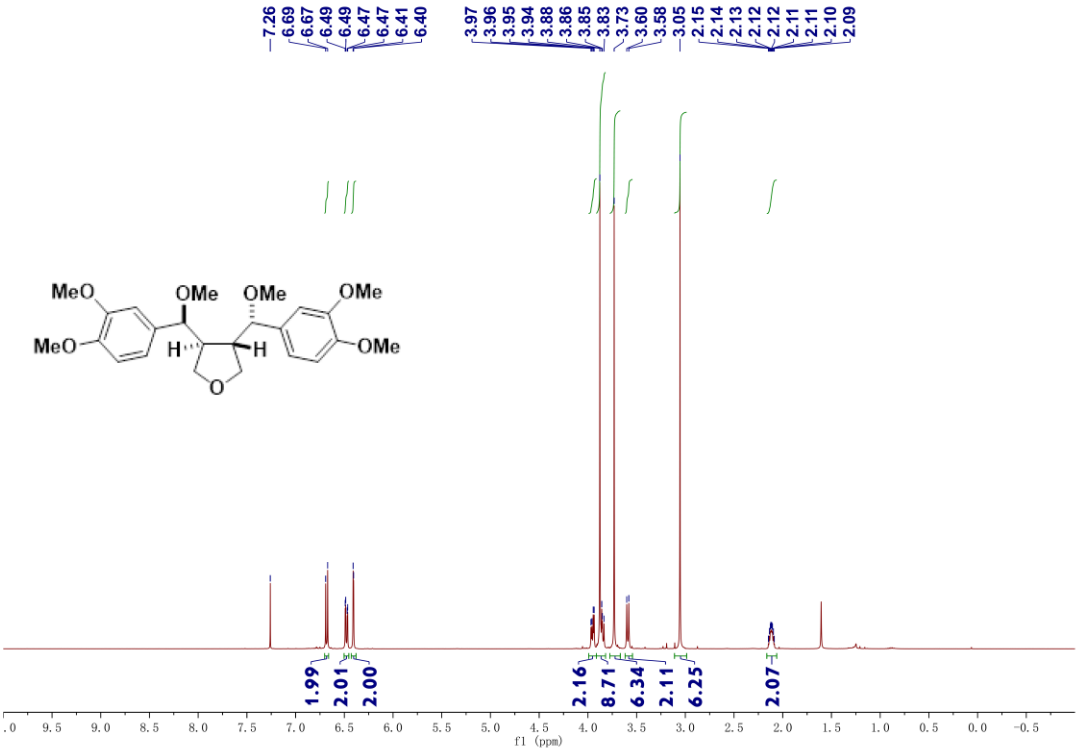

Supplementary Figure 130. ^{13}C NMR spectrum of compound **22u-A** (101 MHz, $CDCl_3$)

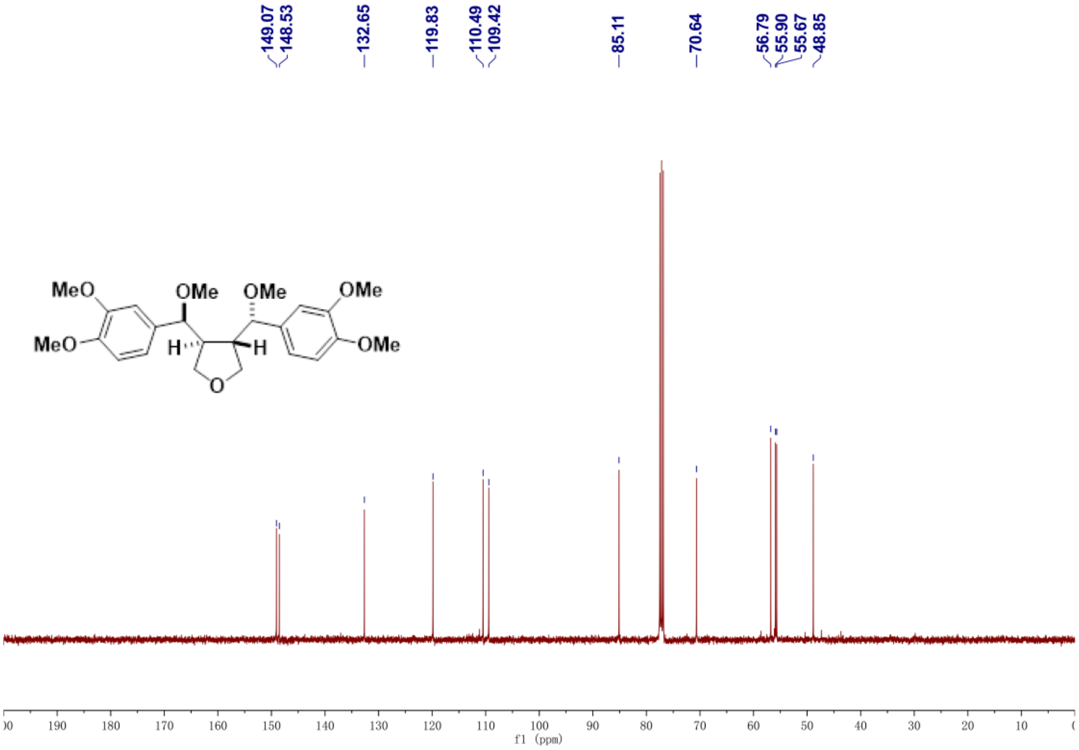

Supplementary Figure 131. ^{1}H NMR spectrum of compound **22v-A** (400 MHz, $CDCl_3$)

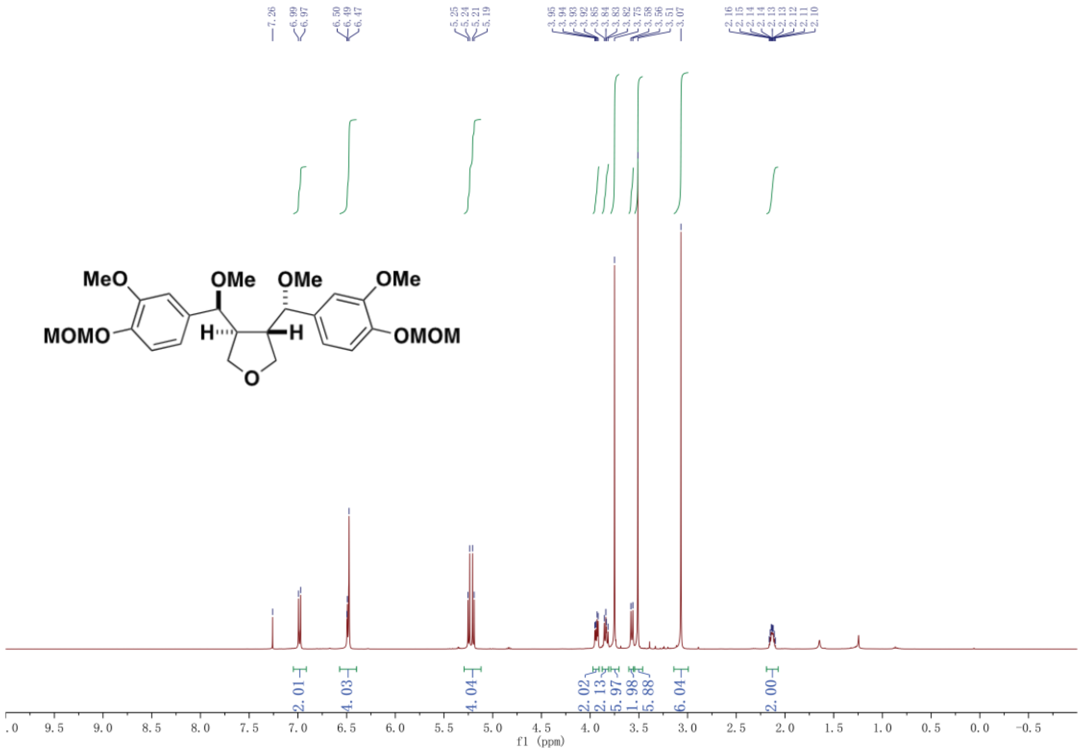

Supplementary Figure 132. ^{13}C NMR spectrum of compound **22v-A** (101 MHz, $CDCl_3$)

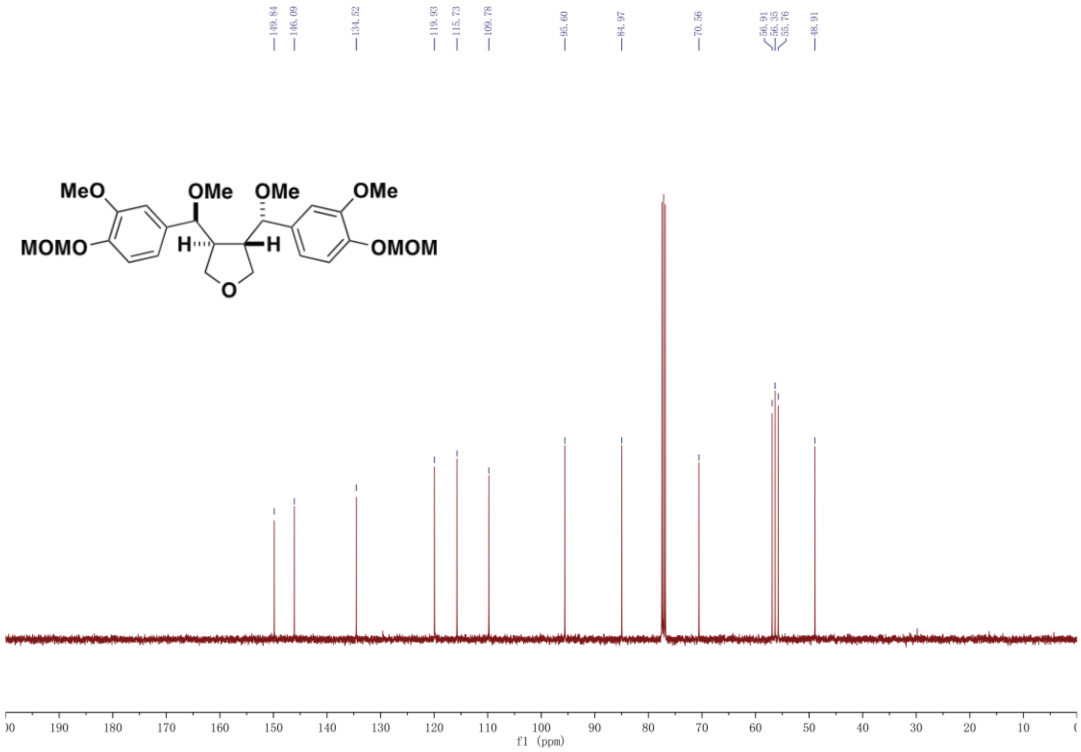

Supplementary Figure 133. ^{1}H NMR spectrum of compound **22w-A** (400 MHz, $CDCl_3$)

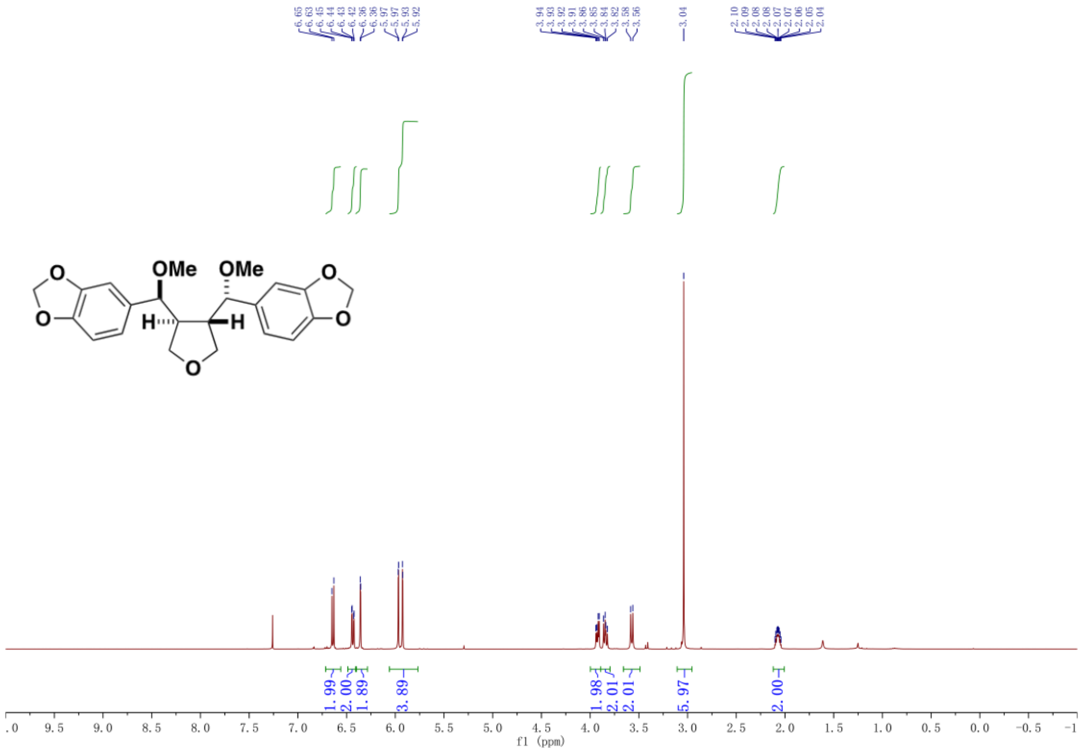

Supplementary Figure 134. ^{13}C NMR spectrum of compound **22w-A** (101 MHz, $CDCl_3$)

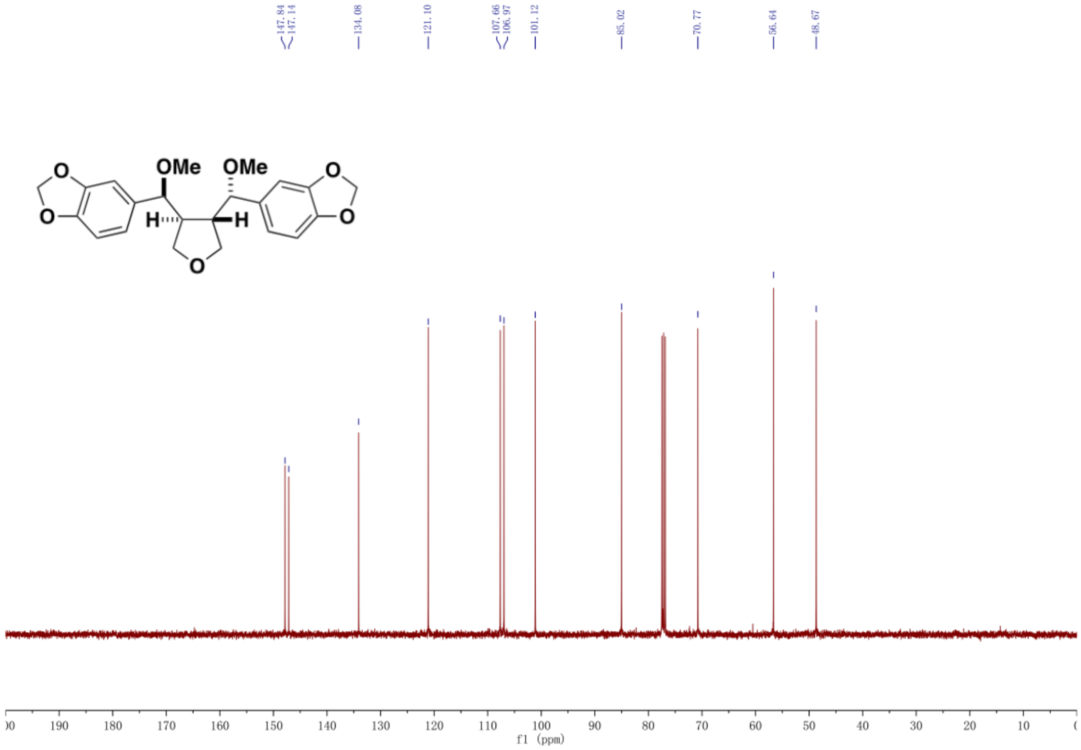

Supplementary Figure 135. ^{1}H NMR spectrum of compound **22x-A** (400 MHz, $CDCl_3$)

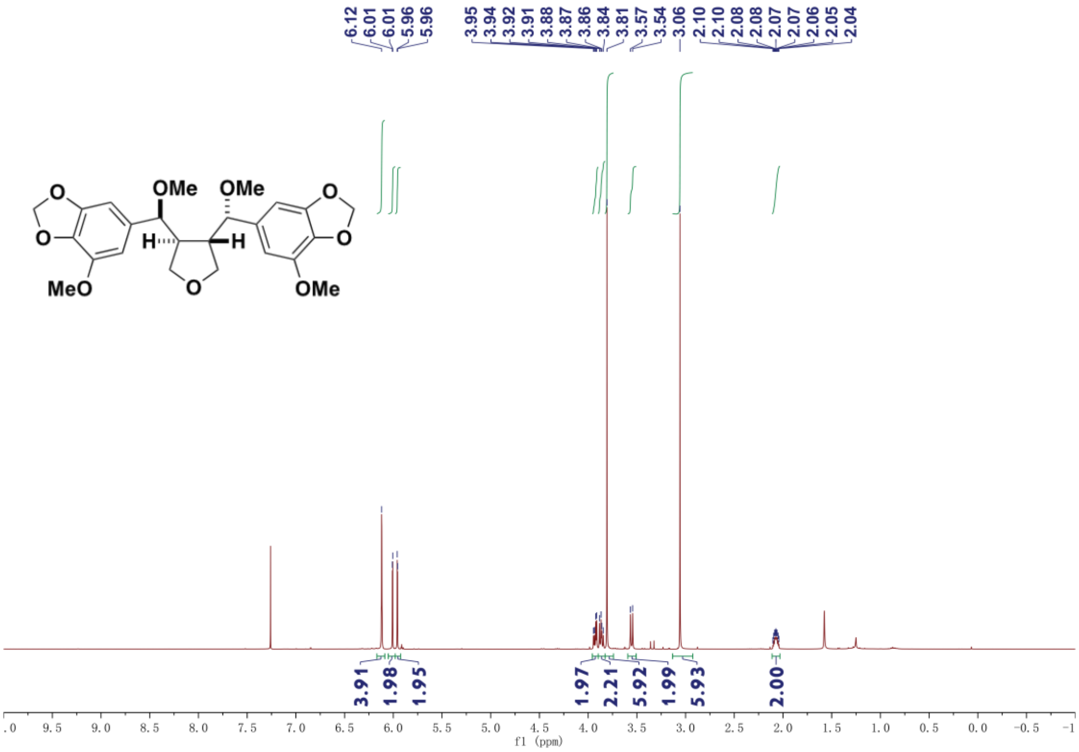

Supplementary Figure 136. ^{13}C NMR spectrum of compound **22x-A** (101 MHz, $CDCl_3$)

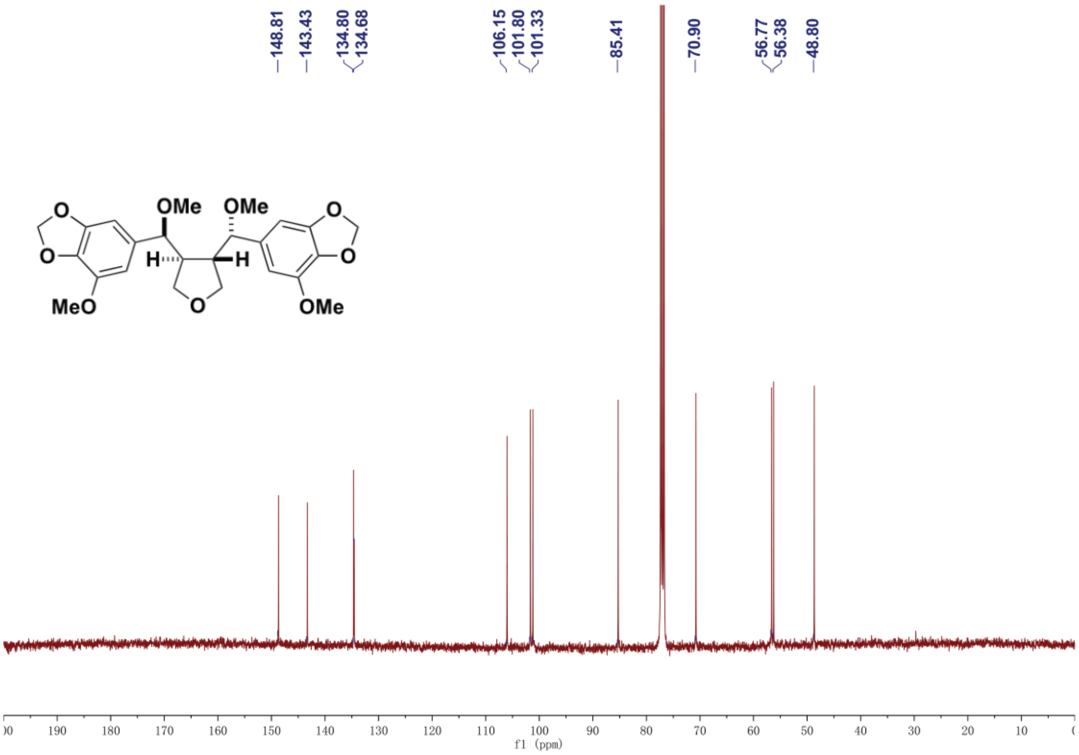

Supplementary Figure 137. ^{1}H NMR spectrum of compound **22y-A** (400 MHz, $CDCl_3$)

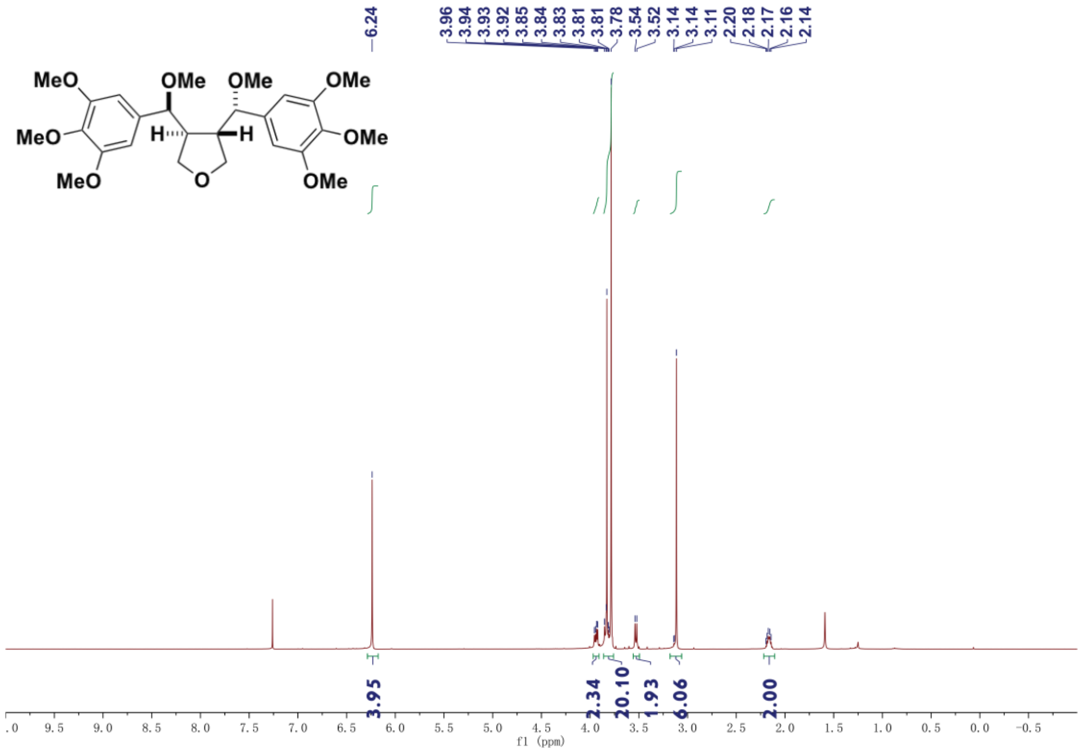

Supplementary Figure 138. ^{13}C NMR spectrum of compound **22y-A** (101 MHz, $CDCl_3$)

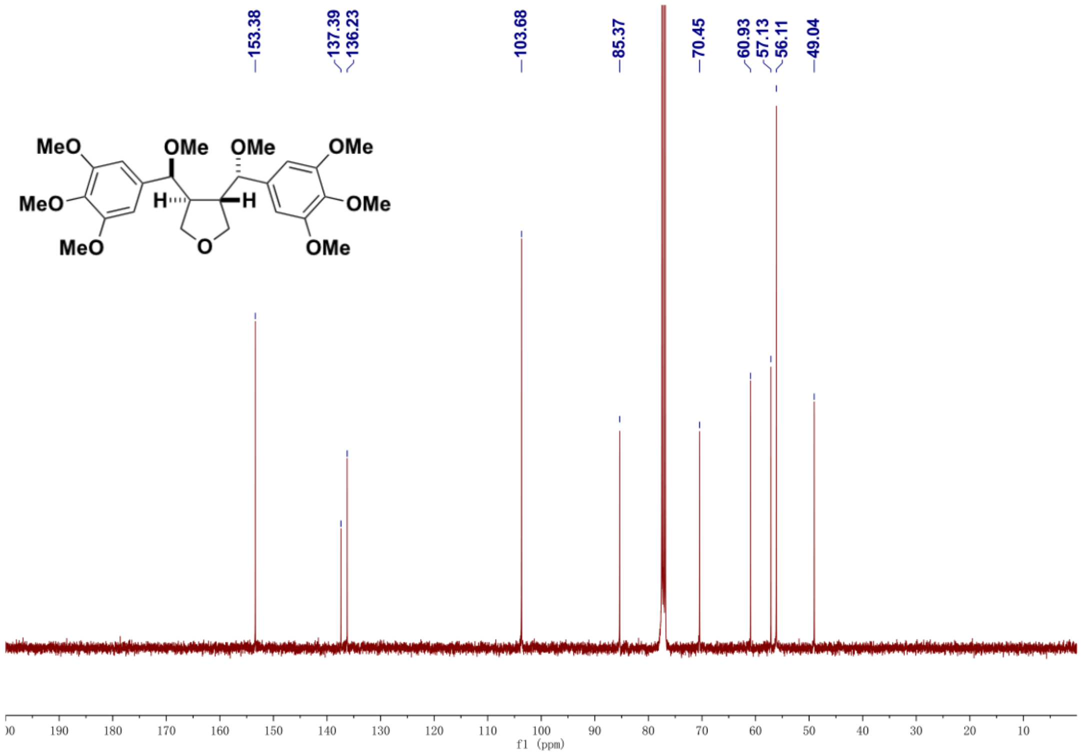

Supplementary Figure 139. ^{1}H NMR spectrum of compound **22z-A** (400 MHz, $CDCl_3$)

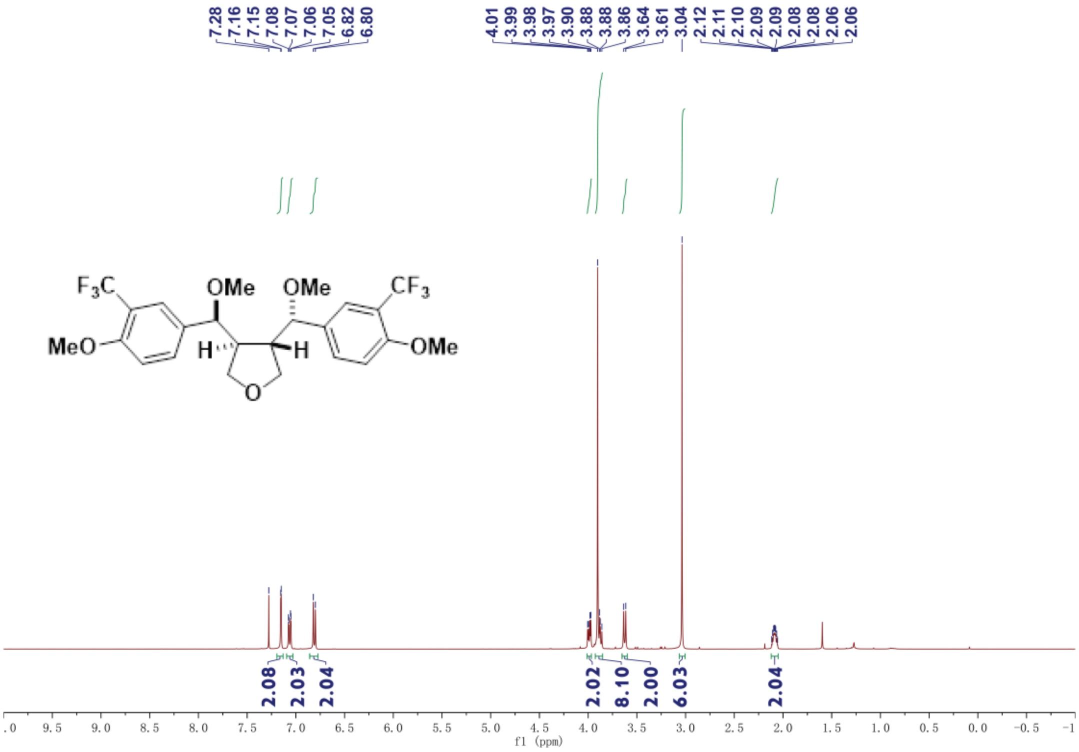

Supplementary Figure 140. ^{13}C NMR spectrum of compound **22z-A** (101 MHz, $CDCl_3$)

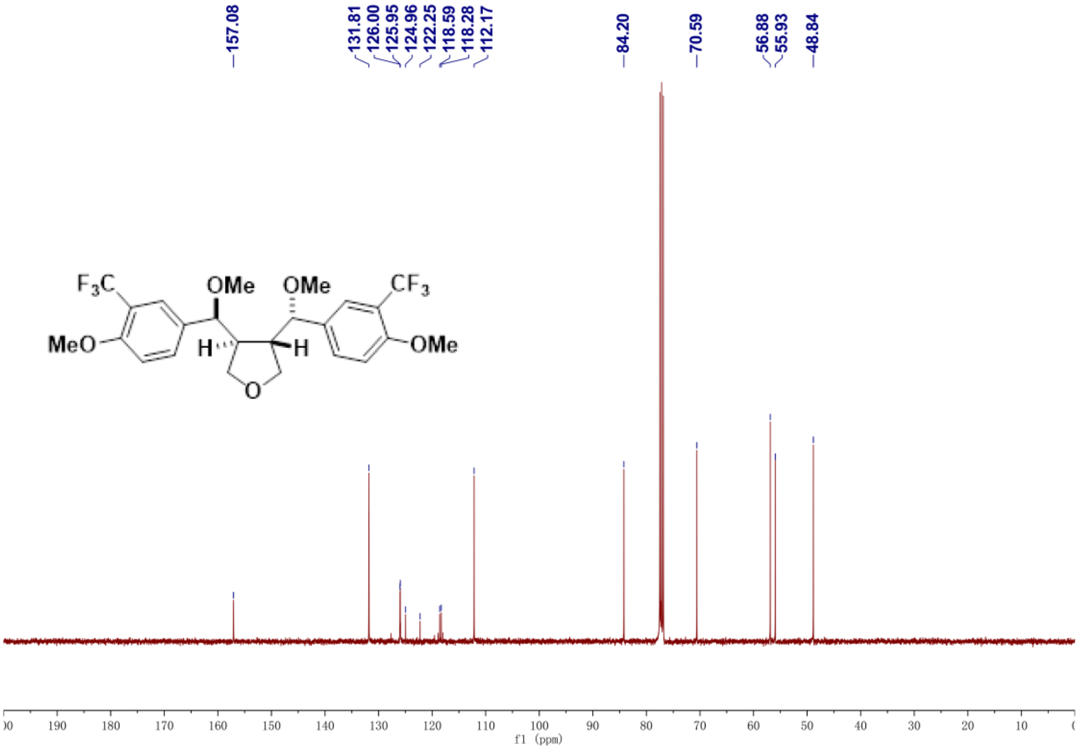

Supplementary Figure 141. ^{19}F NMR spectrum of compound **22z-A** (377 MHz, $CDCl_3$)

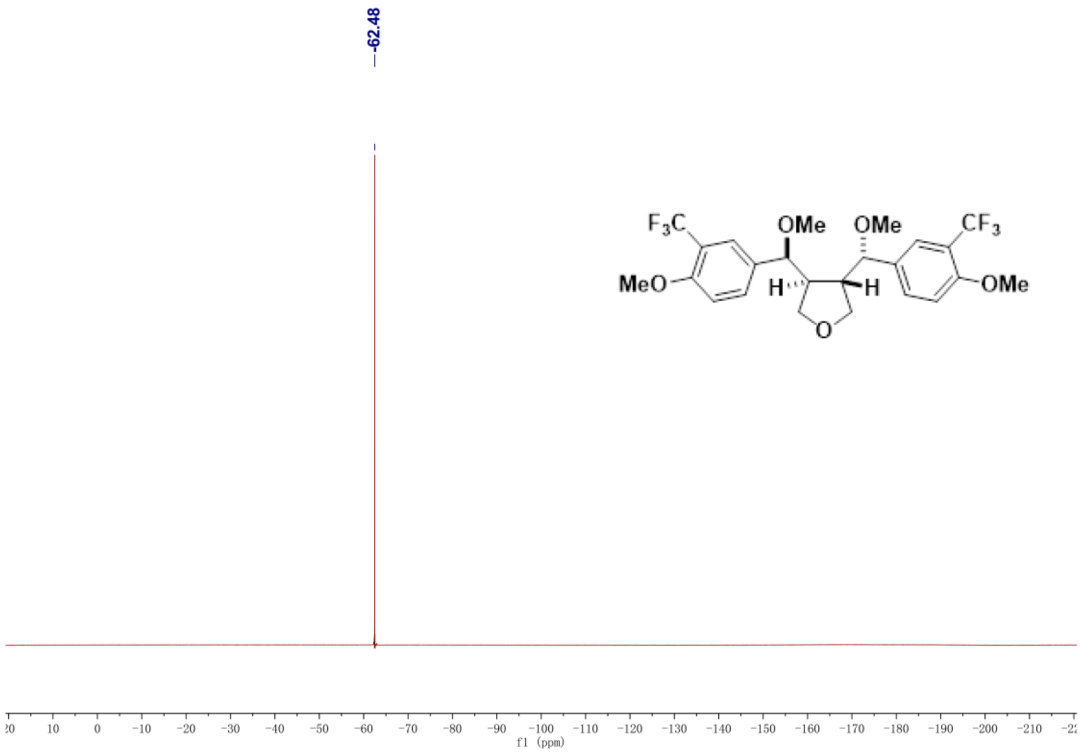

Supplementary Figure 142. ^{1}H NMR spectrum of compound **22aa-A** (400 MHz, $CDCl_3$)

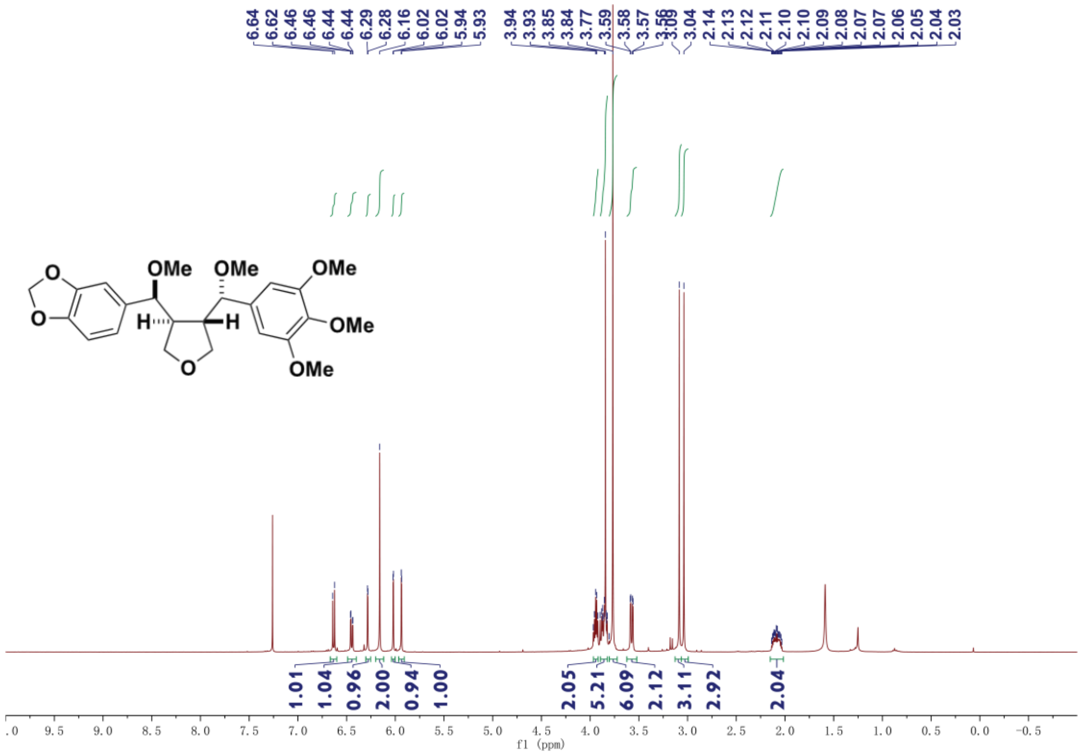

Supplementary Figure 143. ^{13}C NMR spectrum of compound **22aa-A** (101 MHz, $CDCl_3$)

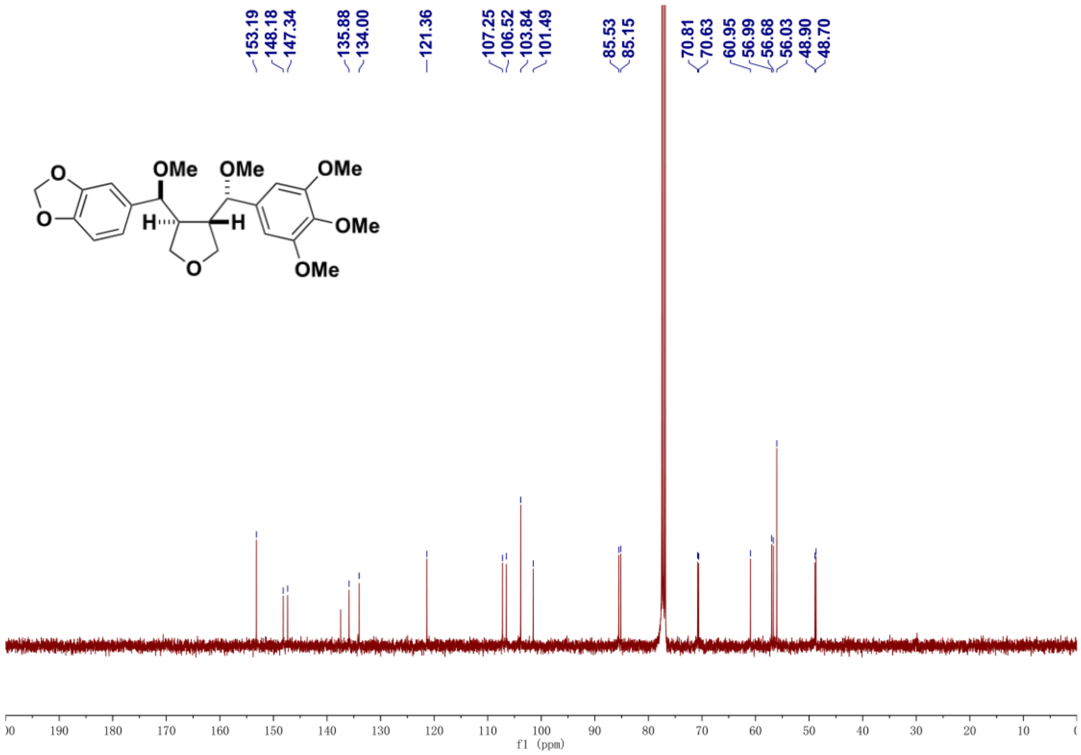

Supplementary Figure 144. ^{1}H NMR spectrum of compound **22ab-A** (400 MHz, $CDCl_3$)

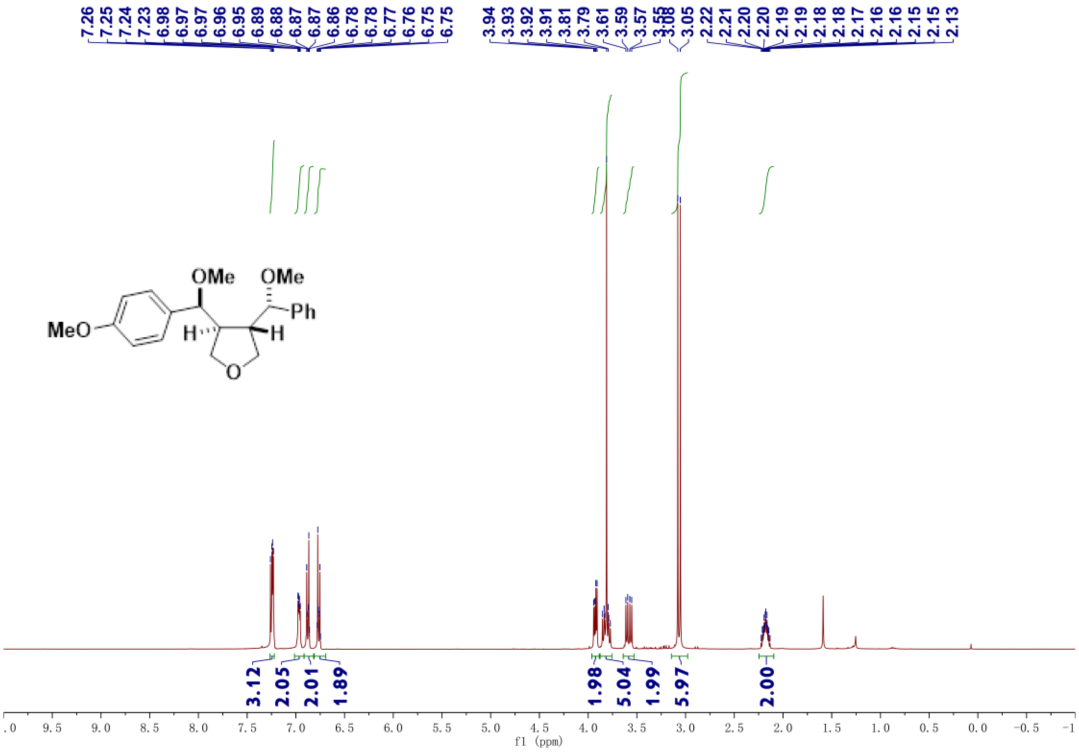

Supplementary Figure 145. ^{13}C NMR spectrum of compound **22ab-A** (101 MHz, $CDCl_3$)

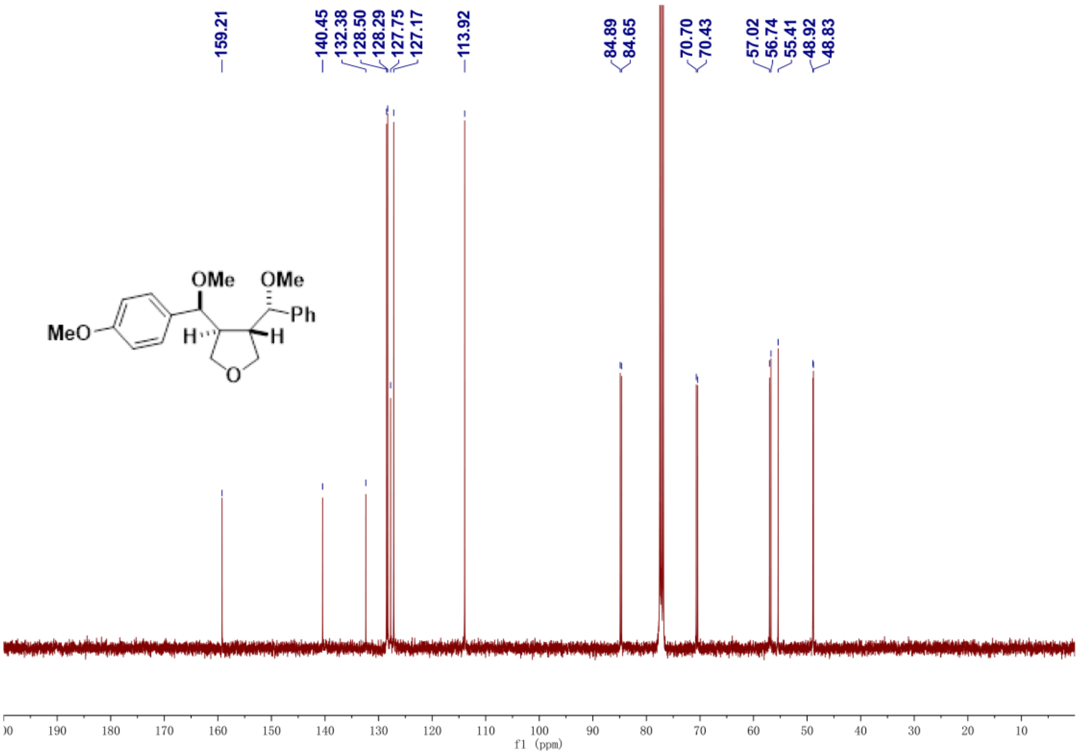

Supplementary Figure 146. ^{1}H NMR spectrum of compound **22ac-A** (400 MHz, $CDCl_3$)

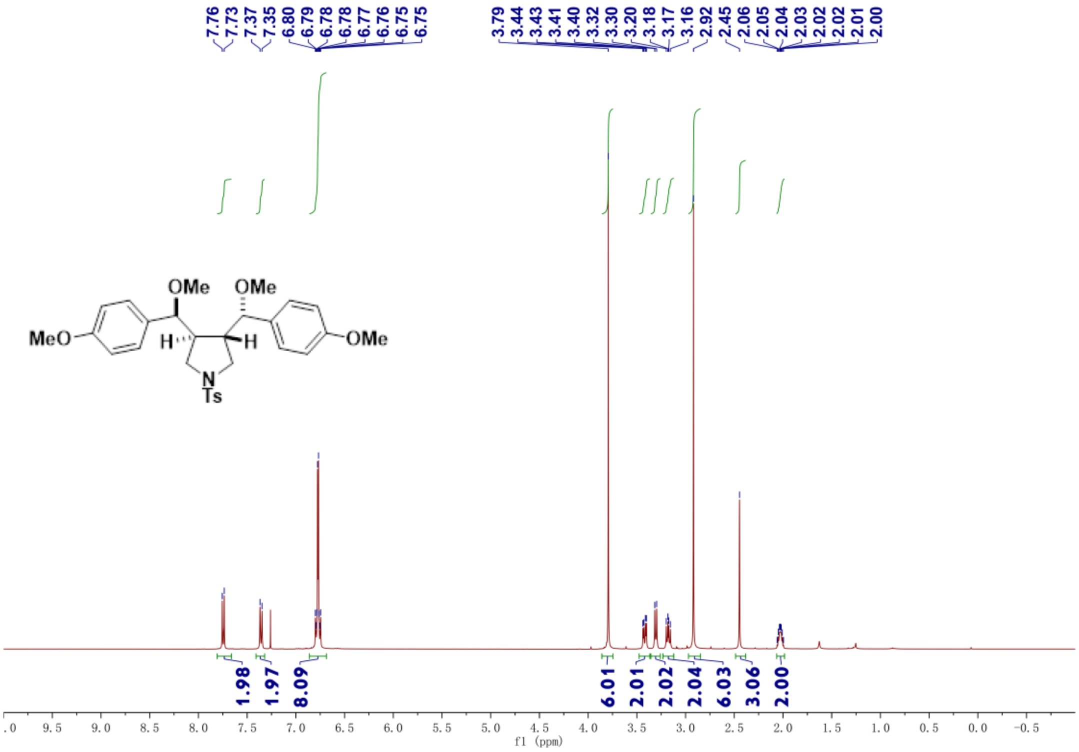

Supplementary Figure 147. ^{13}C NMR spectrum of compound **22ac-A** (101 MHz, $CDCl_3$)

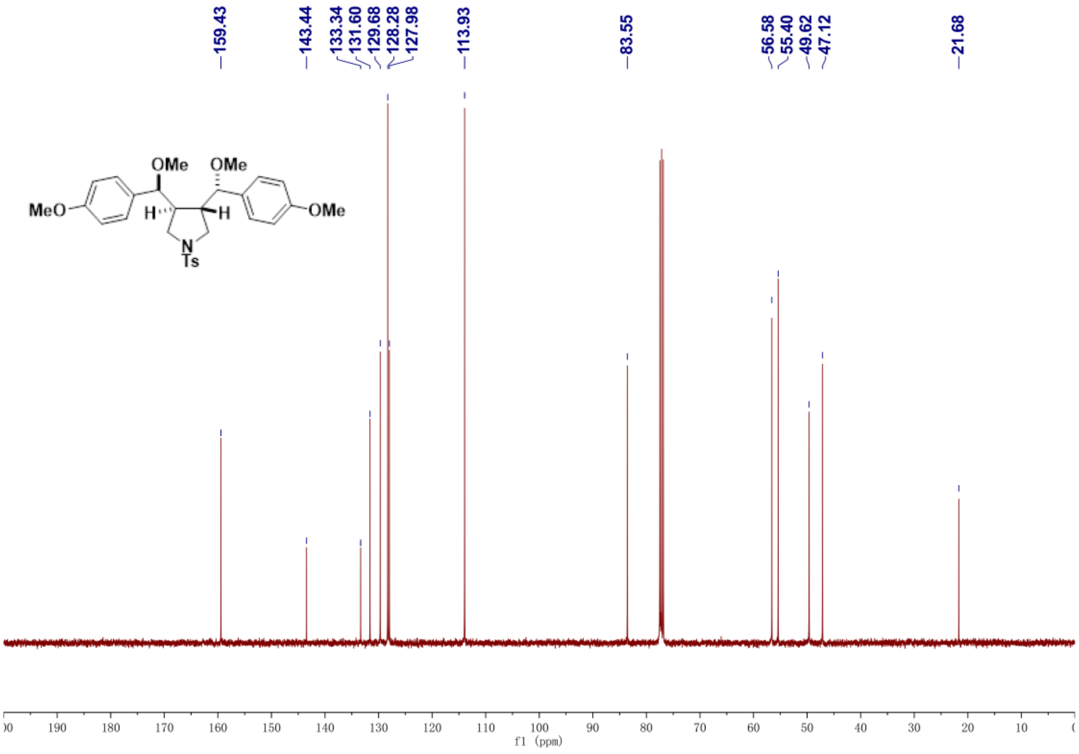

Supplementary Figure 148. ^{1}H NMR spectrum of compound **22ad-A** (400 MHz, $CDCl_3$)

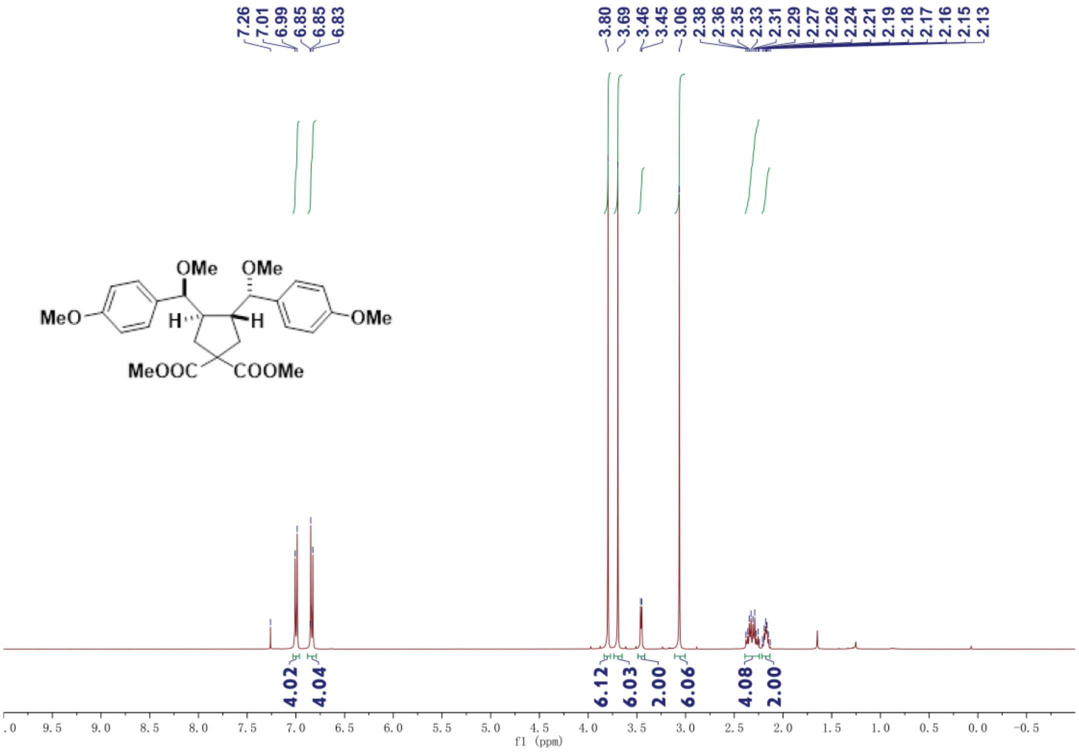

Supplementary Figure 149. ^{13}C NMR spectrum of compound **22ad-A** (101 MHz, $CDCl_3$)

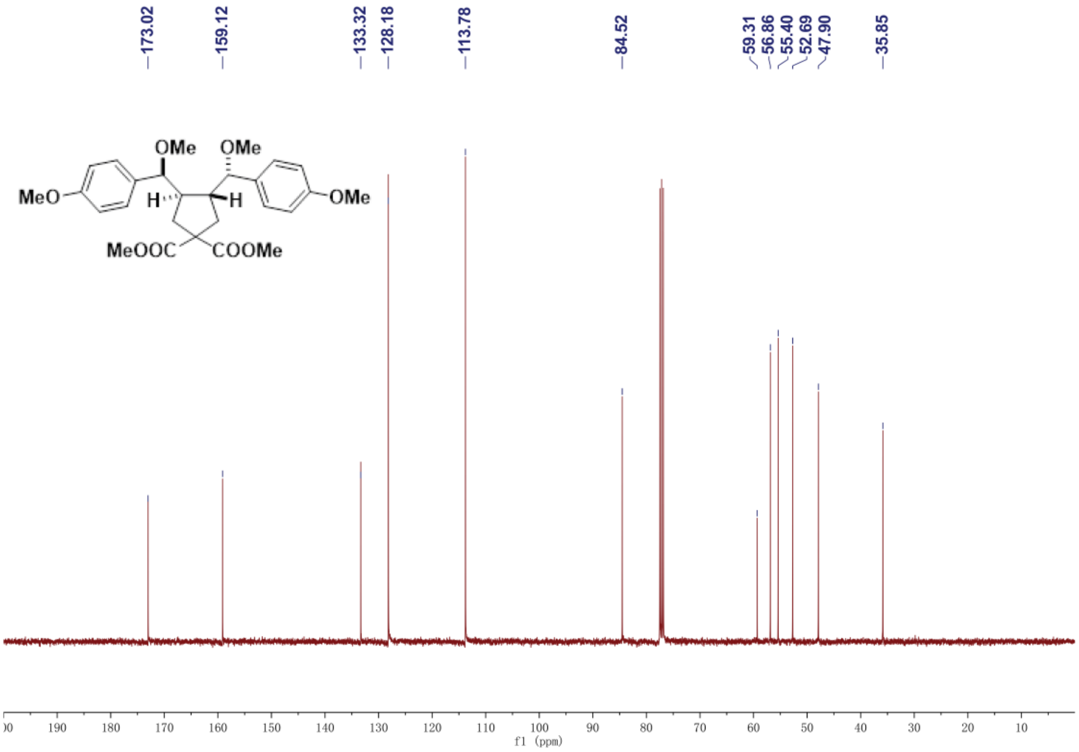

Supplementary Figure 150. ^{1}H NMR spectrum of compound **19a** (400 MHz, $CDCl_3$)

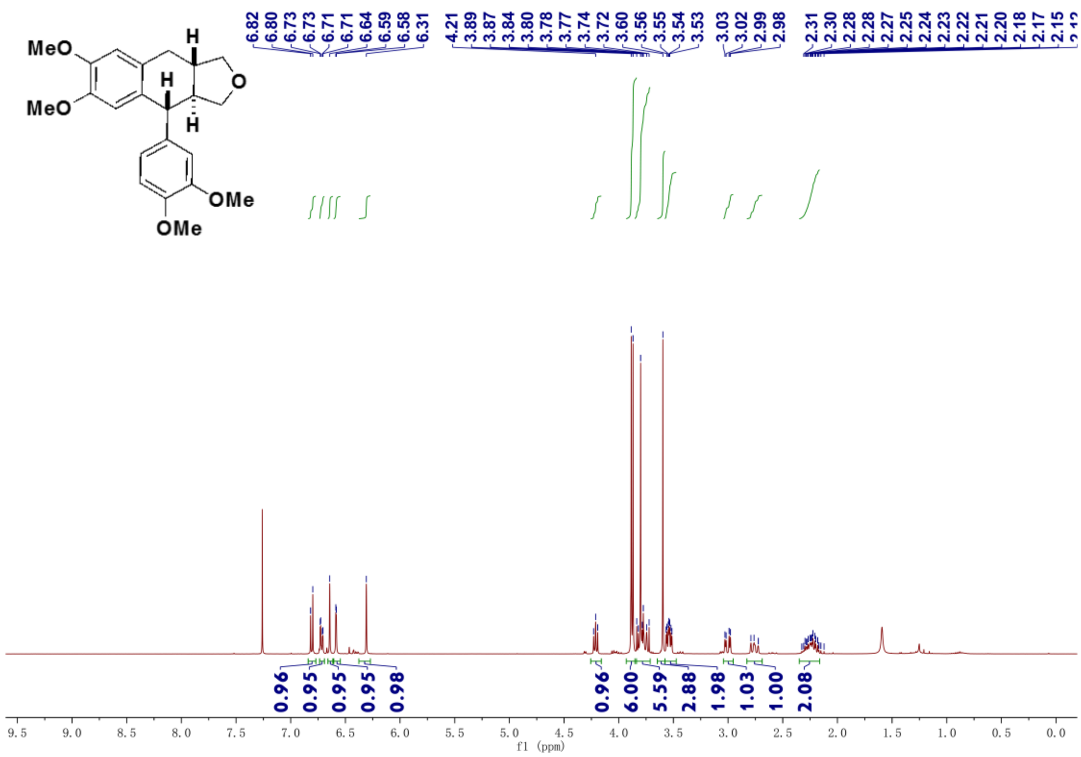

Supplementary Figure 151. ^{13}C NMR spectrum of compound **19a** (101 MHz, $CDCl_3$)

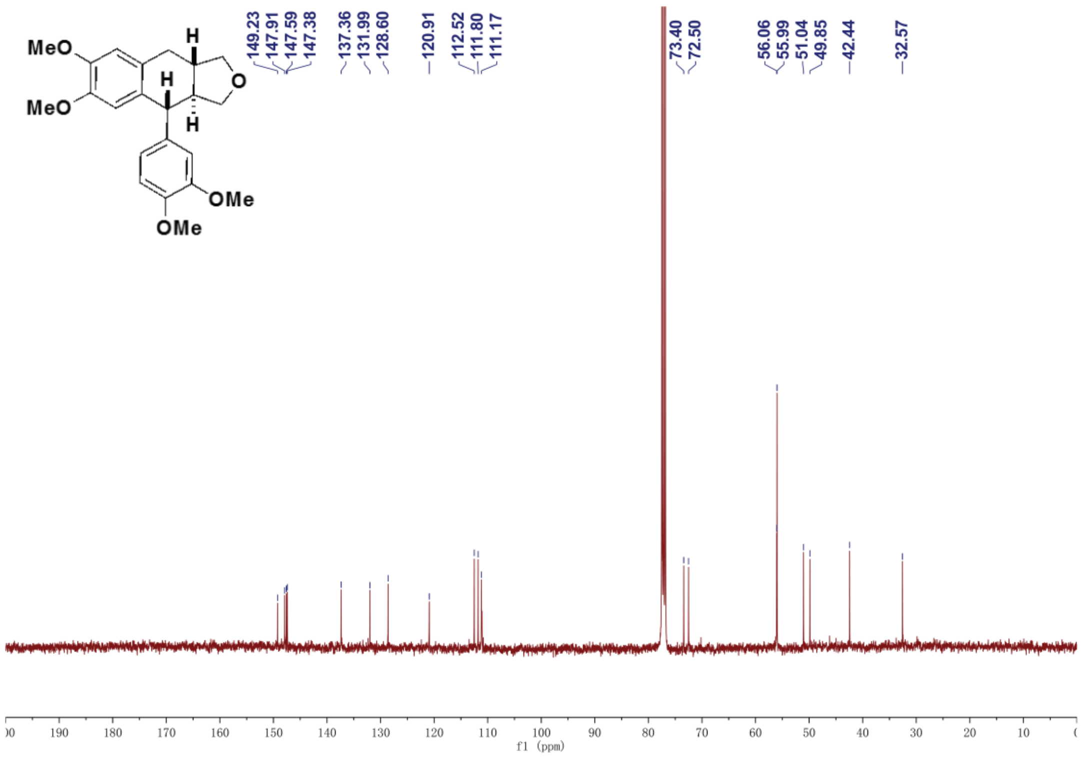

Supplementary Figure 152. ^{1}H NMR spectrum of compound **11 (brassilignan)** (400 MHz, $CDCl_3$)

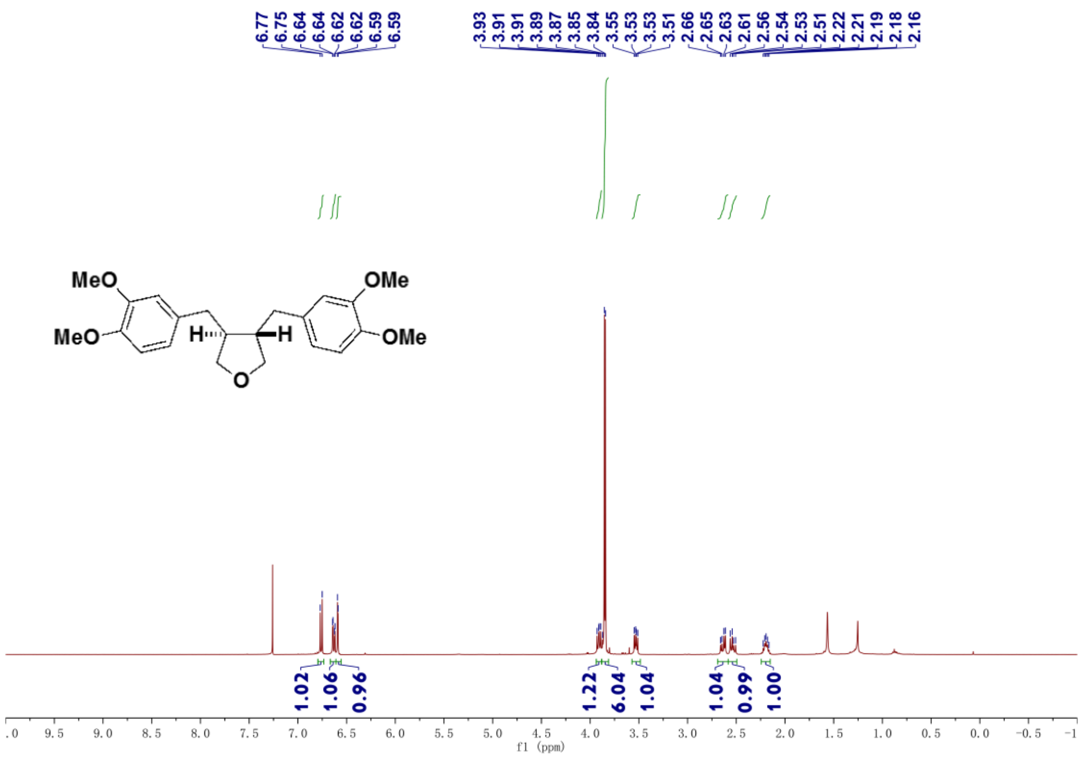

Supplementary Figure 153. ^{13}C NMR spectrum of compound **11 (brassilignan)** (101 MHz, $CDCl_3$)

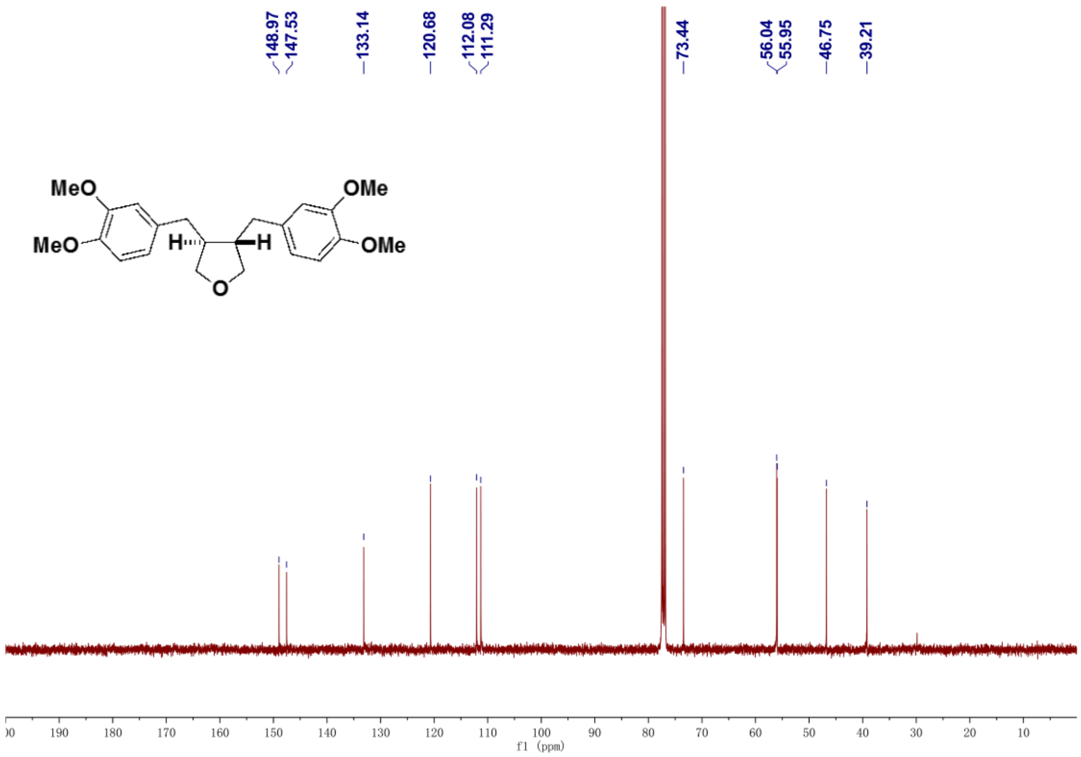

Supplementary Figure 154. ^{1}H NMR spectrum of compound **24a-A** (400 MHz, $CDCl_3$)

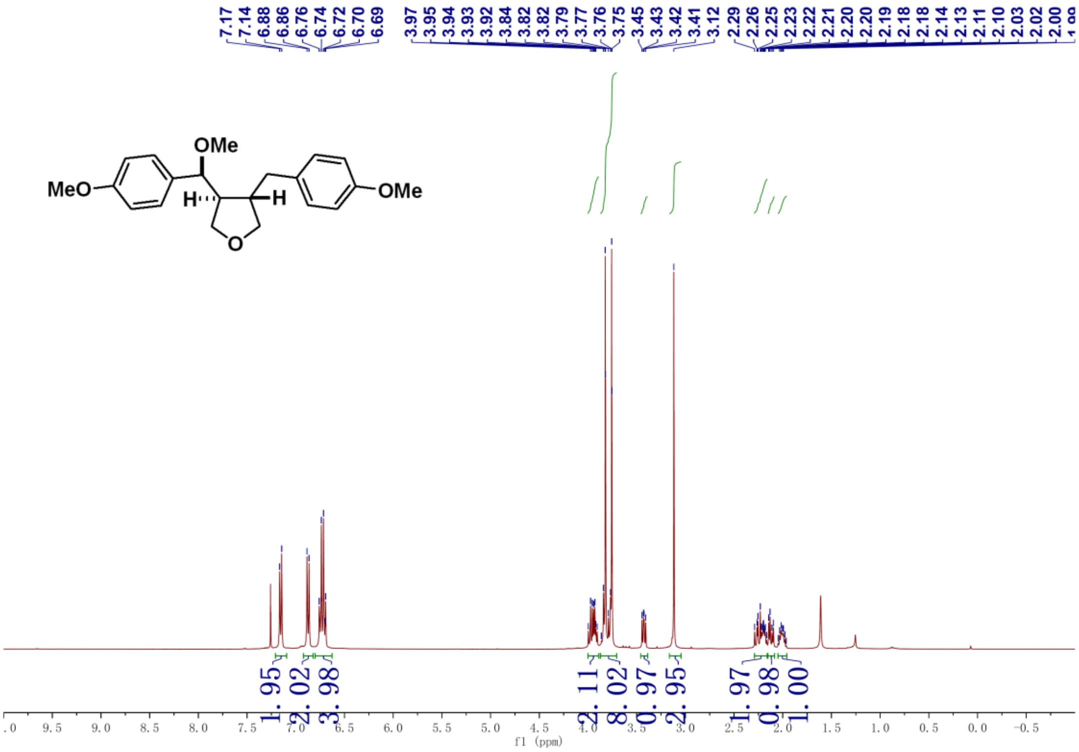

Supplementary Figure 155. ^{13}C NMR spectrum of compound **24a-A** (101 MHz, $CDCl_3$)

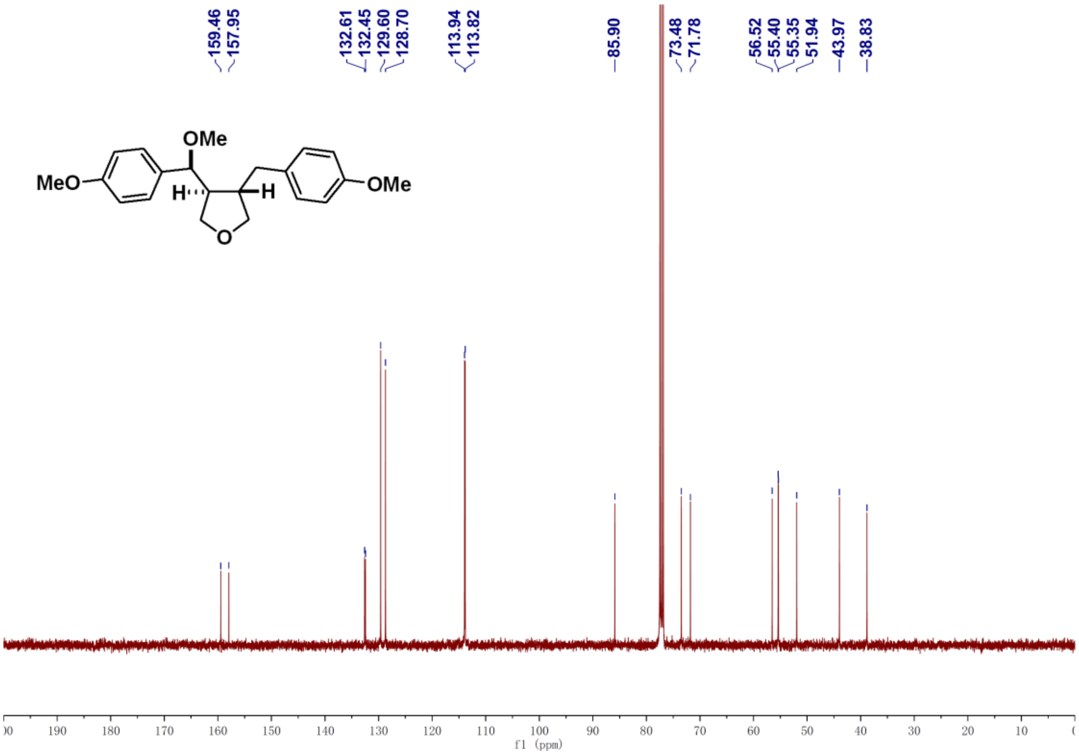

Supplementary Figure 156. 2D NMR spectra of compound **24a-A** ($CDCl_3$)

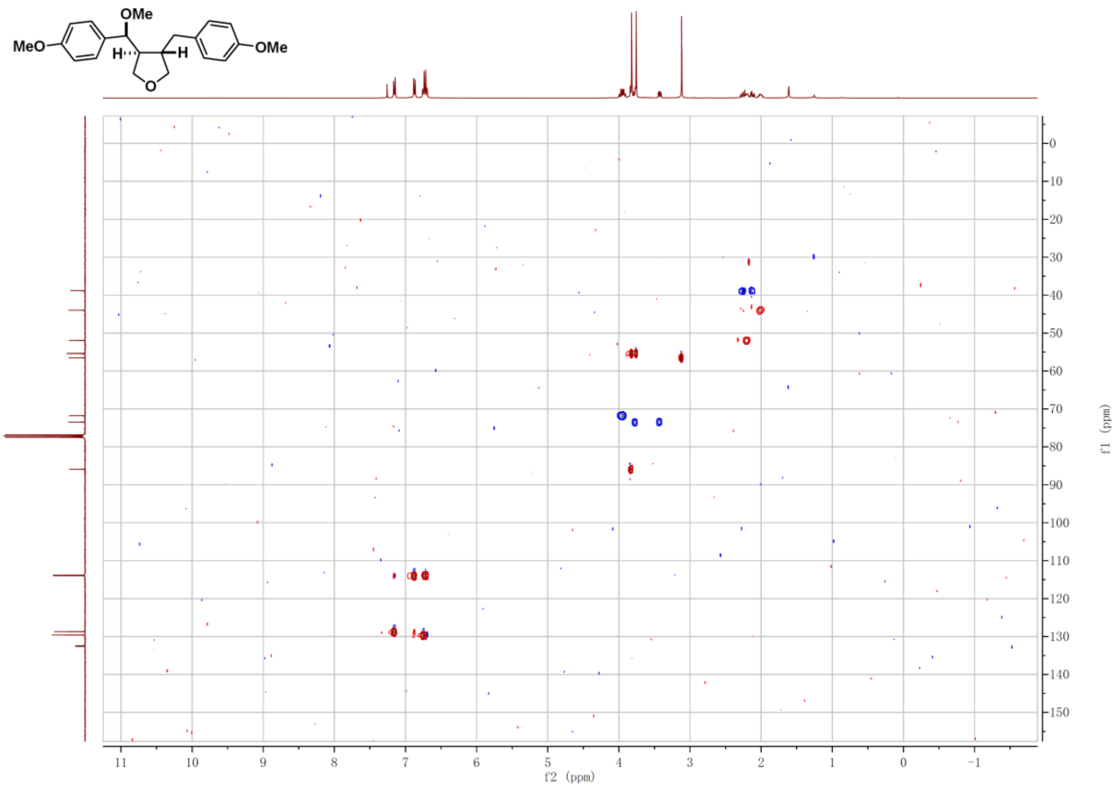

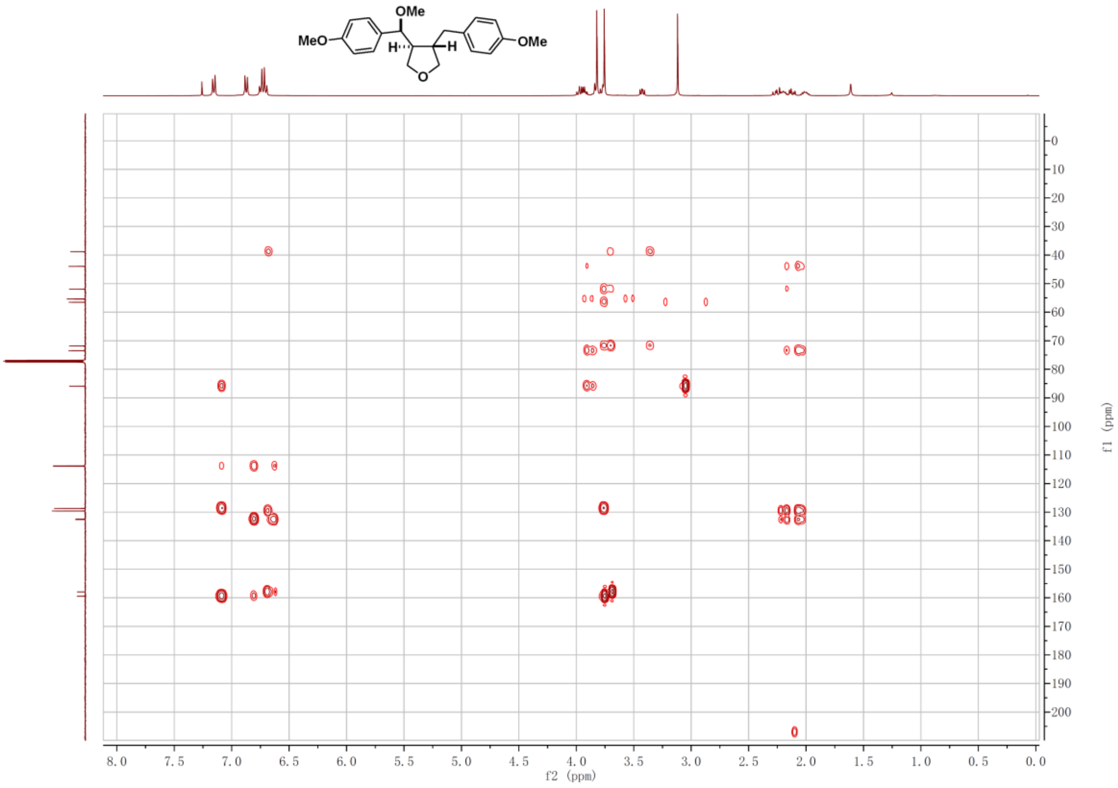

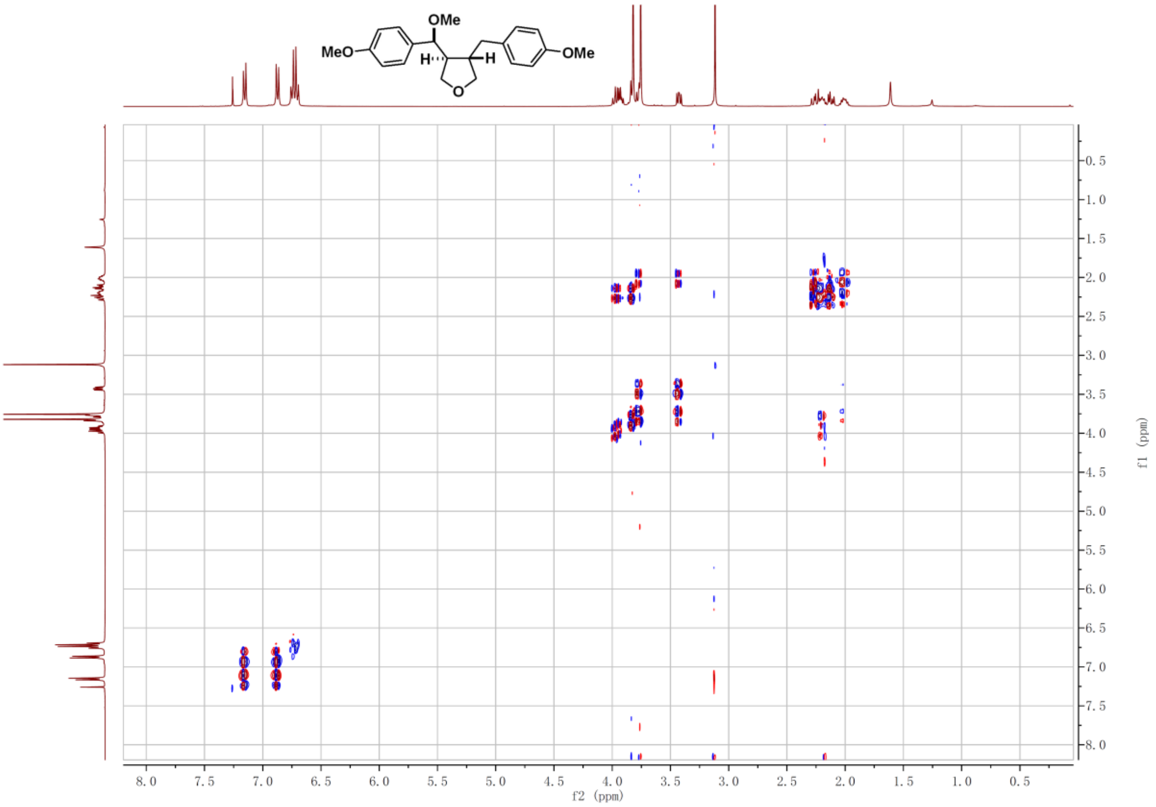
OMe
MeO
OMe
H
H
O
f2 (ppm)
f1 (ppm)

Supplementary Figure 157. ^{1}H NMR spectrum of compound **24a-B (isomer of 24a)** (400 MHz, $CDCl_3$)

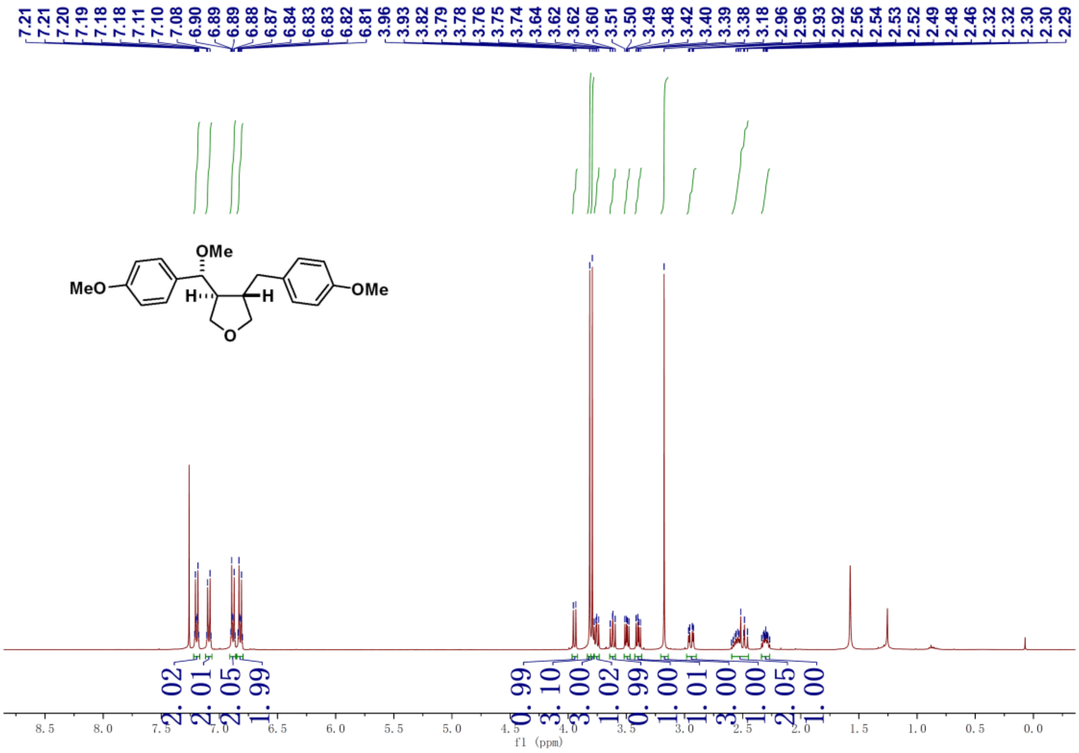

Supplementary Figure 158. ^{13}C NMR spectrum of compound **24a-B (isomer of 24a)** (101 MHz, $CDCl_3$)

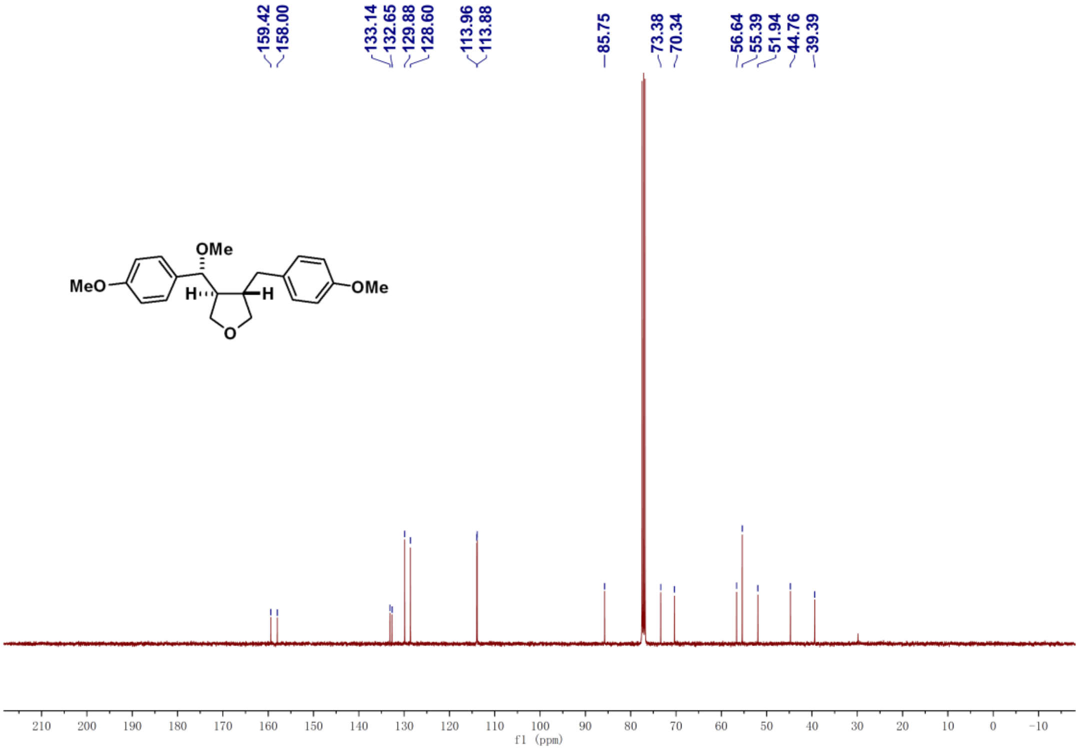

Supplementary Figure 159. 2D NMR spectrum of compound **24a-B (isomer of 24a)** ($CDCl_3$)

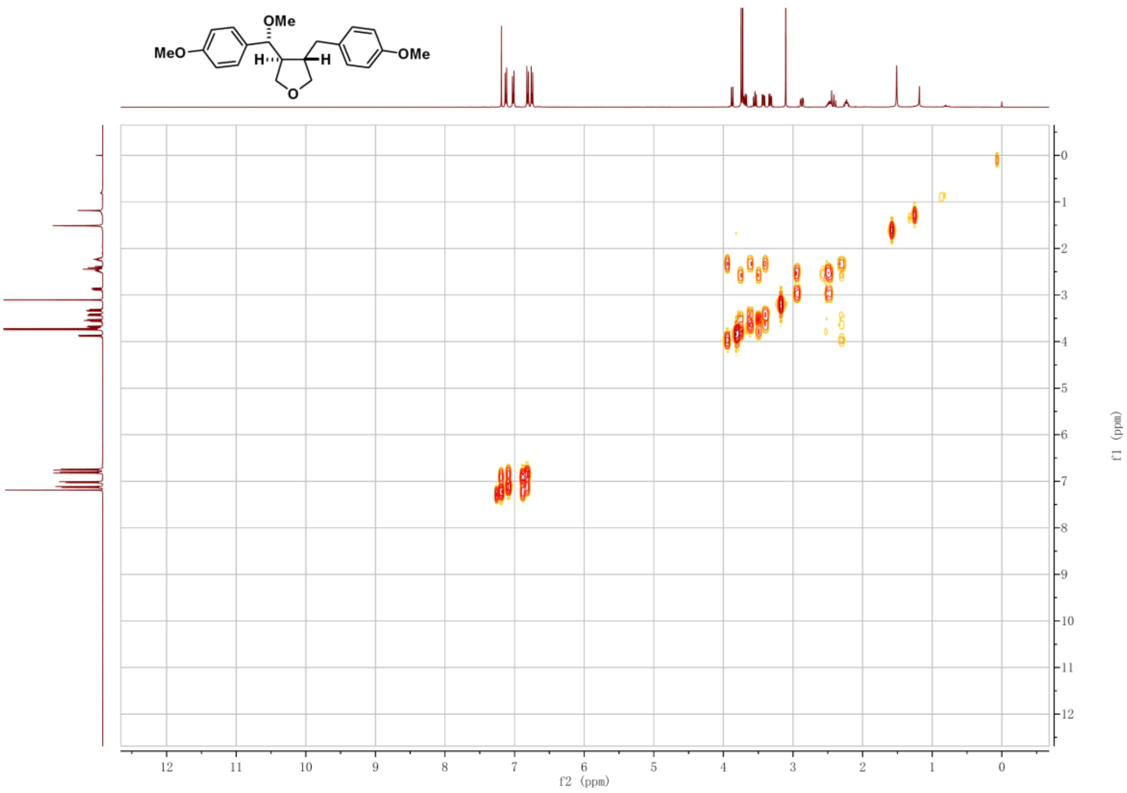

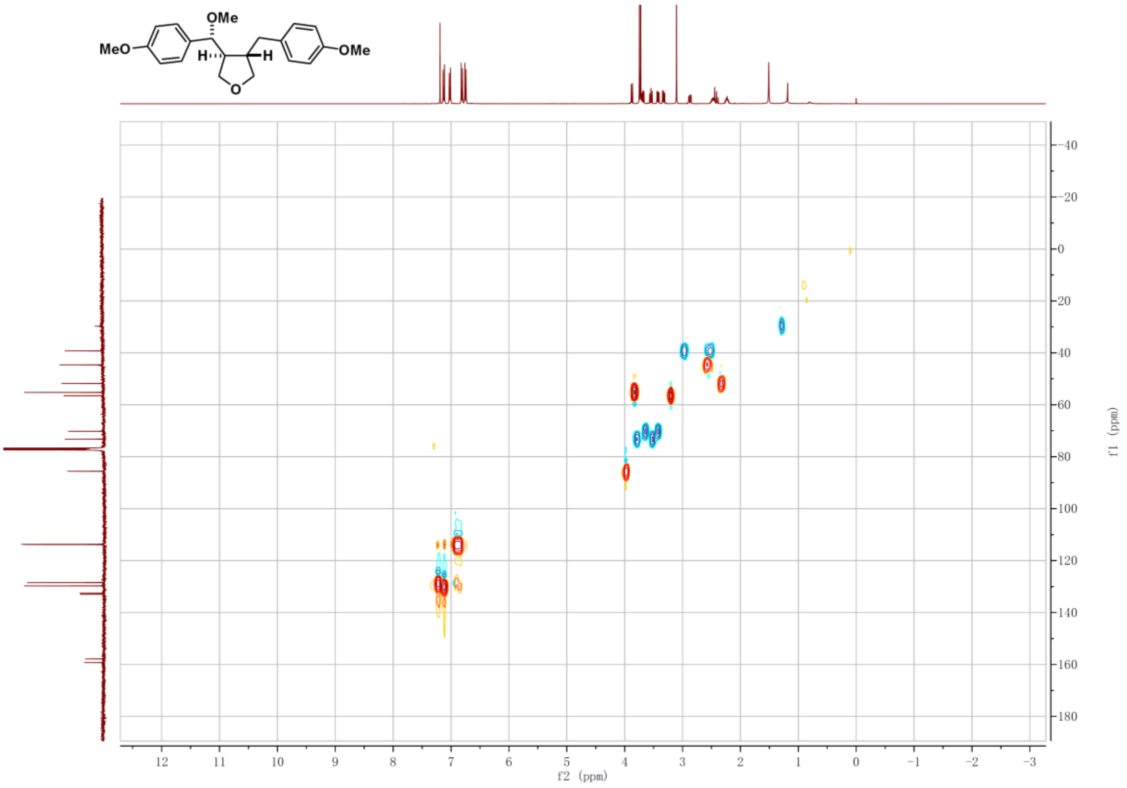

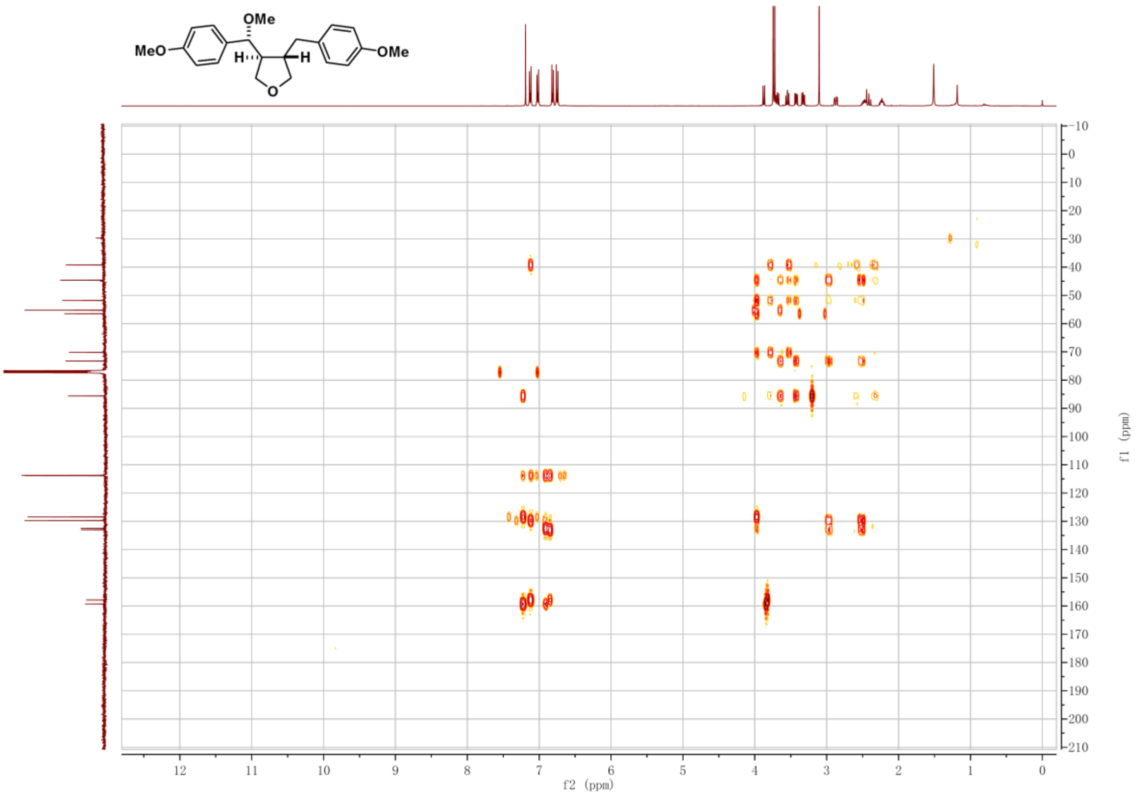
OMe
MeO
H
H
O
OMe
f2 (ppm)
f1 (ppm)

Supplementary Figure 160. ^{1}H NMR spectrum of compound **24b-A** (400 MHz, $CDCl_3$)

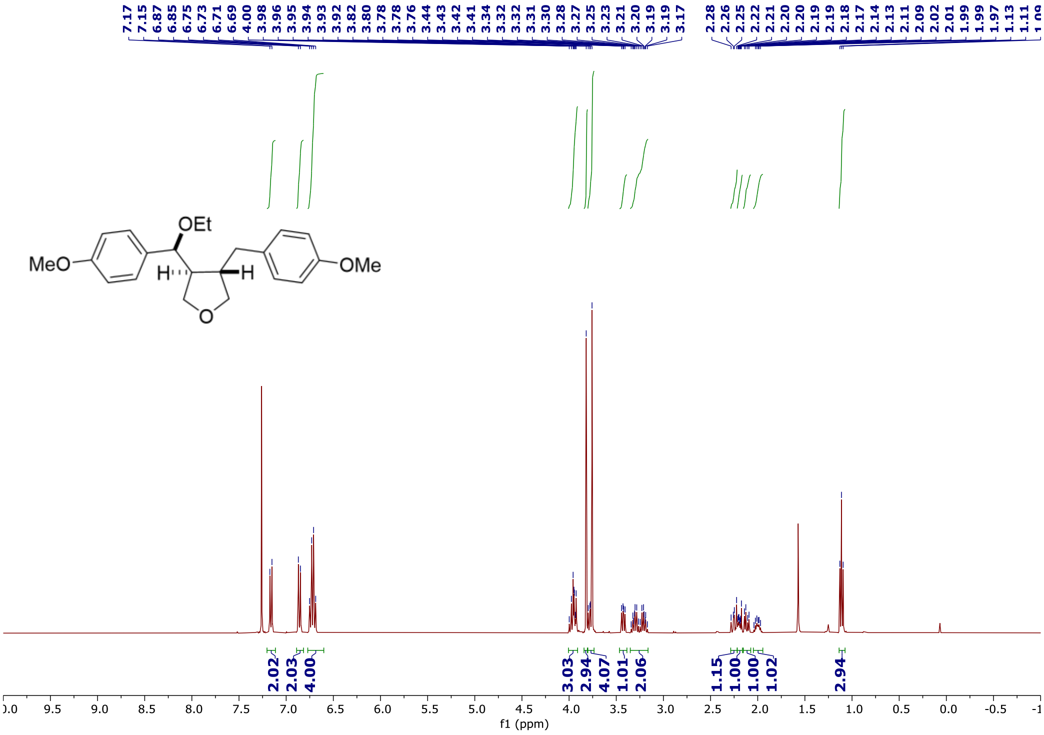

Supplementary Figure 161. ^{13}C NMR spectrum of compound **24b-A** (101 MHz, $CDCl_3$)

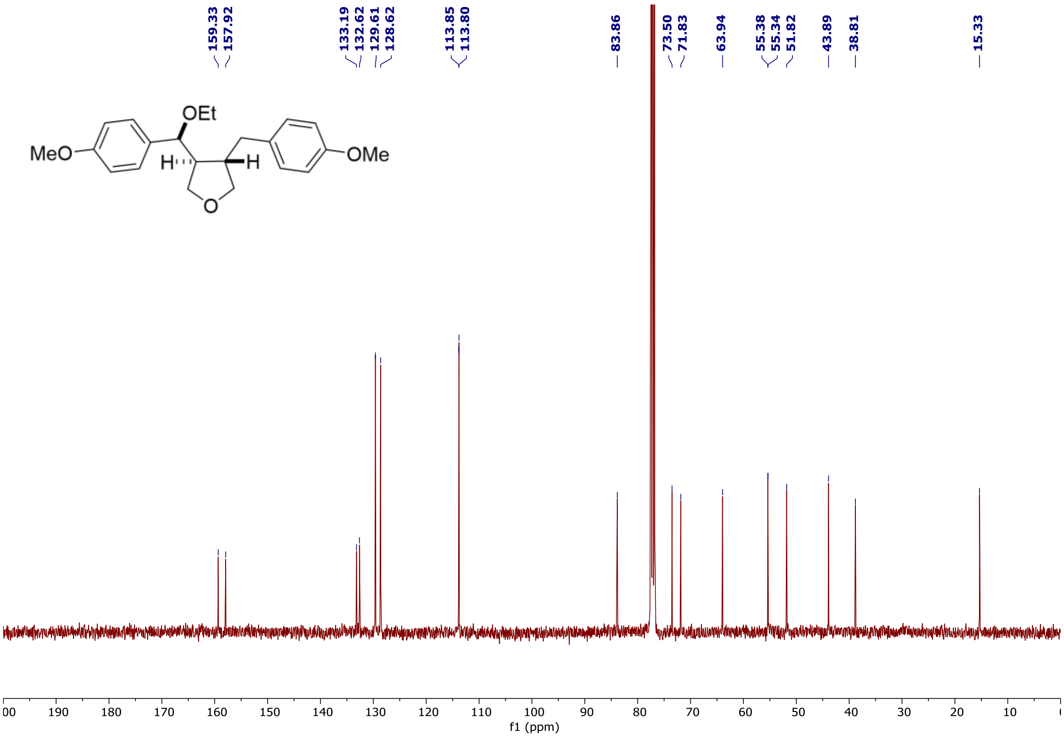

Supplementary Figure 162. ^{1}H NMR spectrum of compound **24c-A** (400 MHz, $CDCl_3$)

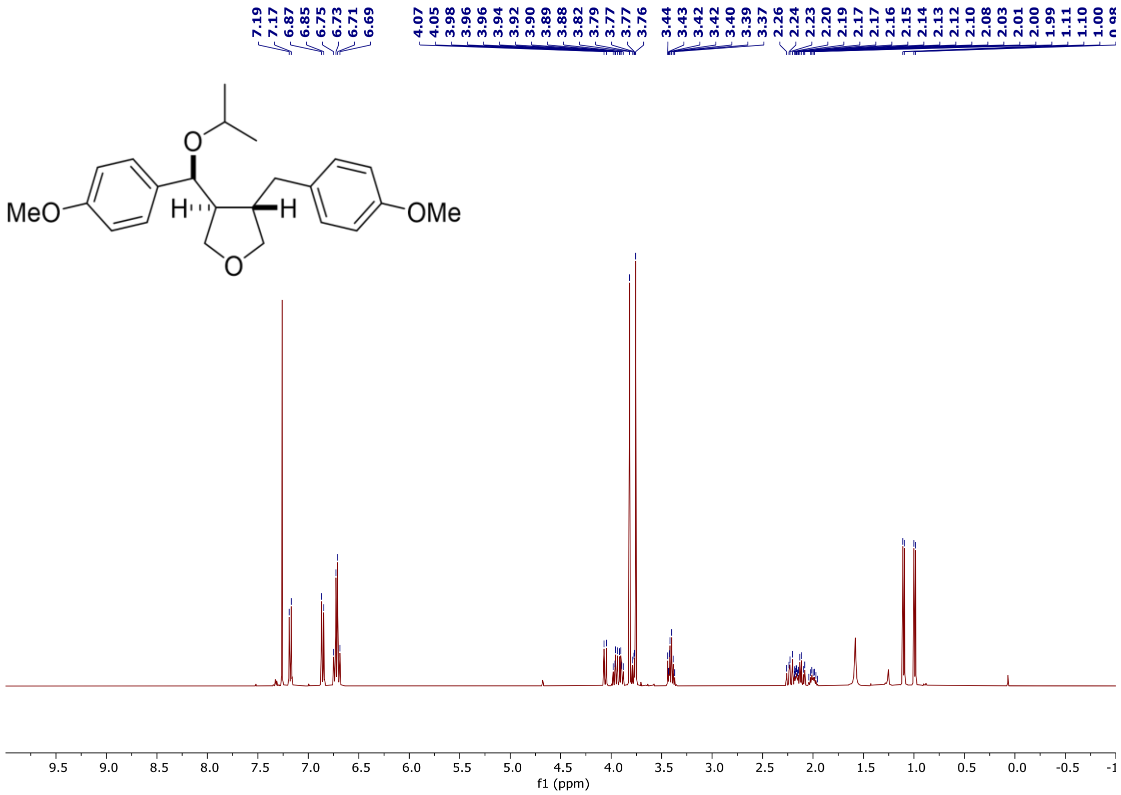

Supplementary Figure 163. ^{13}C NMR spectrum of compound **24c-A** (101 MHz, $CDCl_3$)

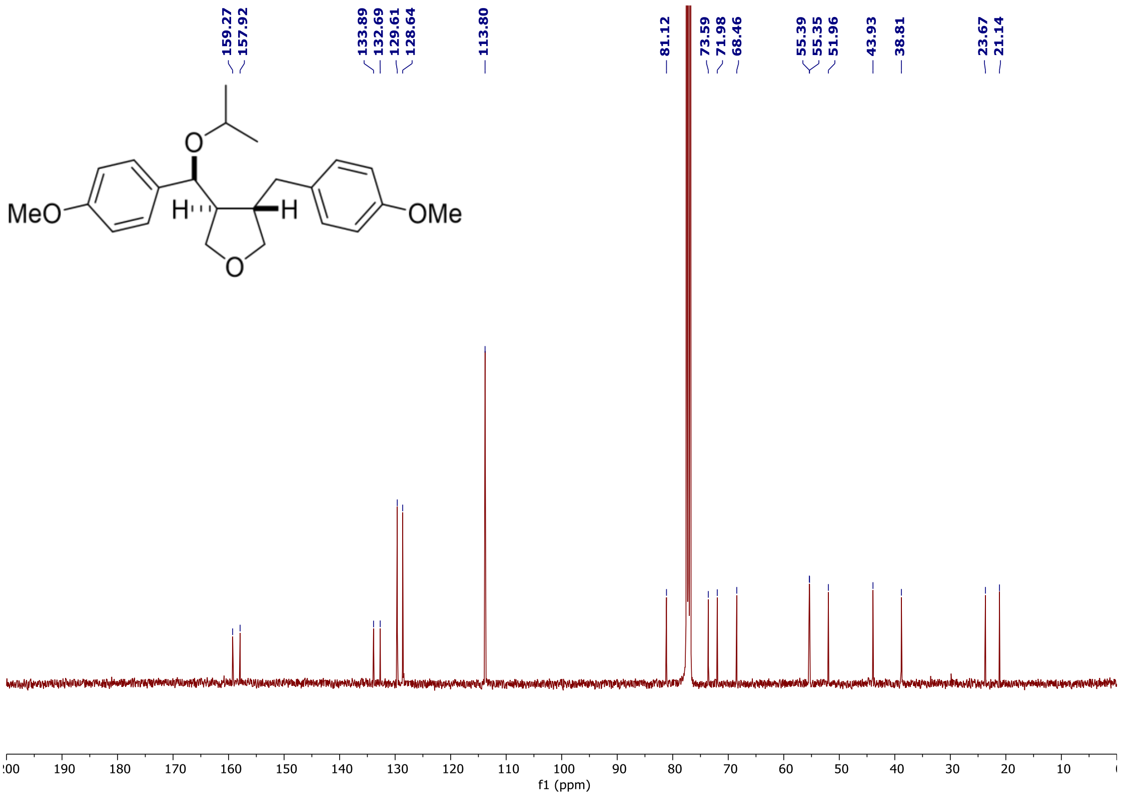

Supplementary Figure 164. ^{1}H NMR spectrum of compound **24d-A** (400 MHz, $CDCl_3$)

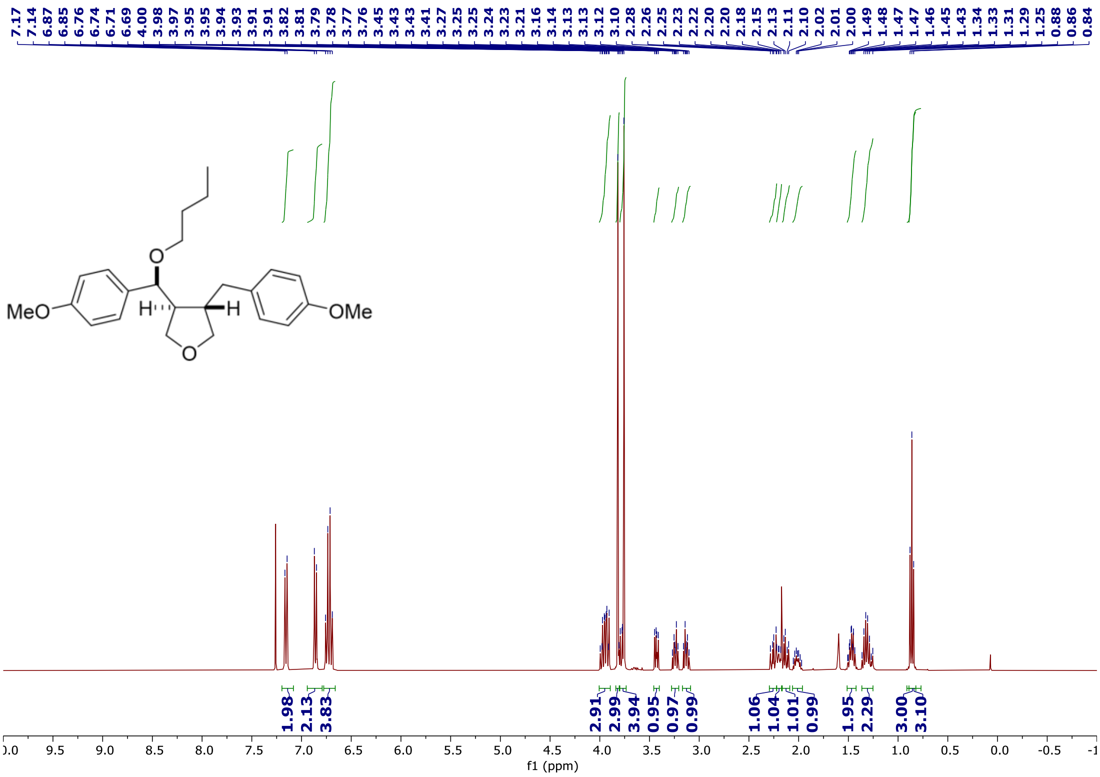

Supplementary Figure 165. ^{13}C NMR spectrum of compound **24d-A** (101 MHz, $CDCl_3$)

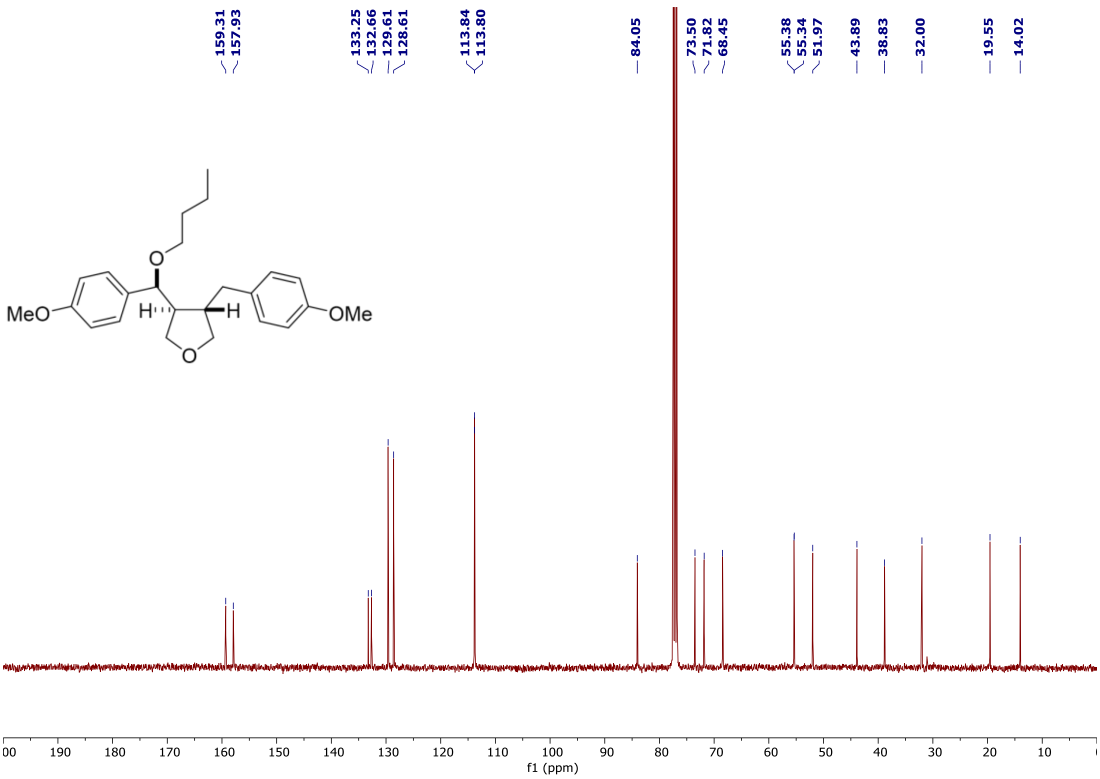

Supplementary Figure 166. ^{1}H NMR spectrum of compound **24e-A** (400 MHz, $CDCl_3$)

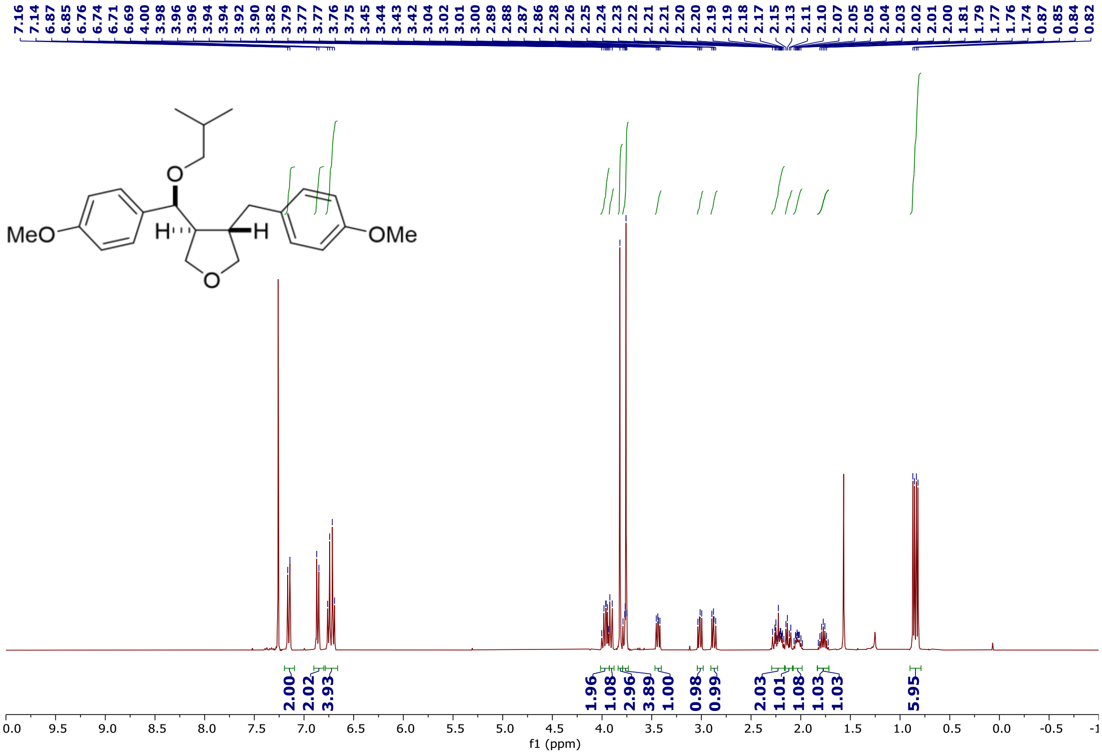

Supplementary Figure 167. ^{13}C NMR spectrum of compound **24e-A** (101 MHz, $CDCl_3$)

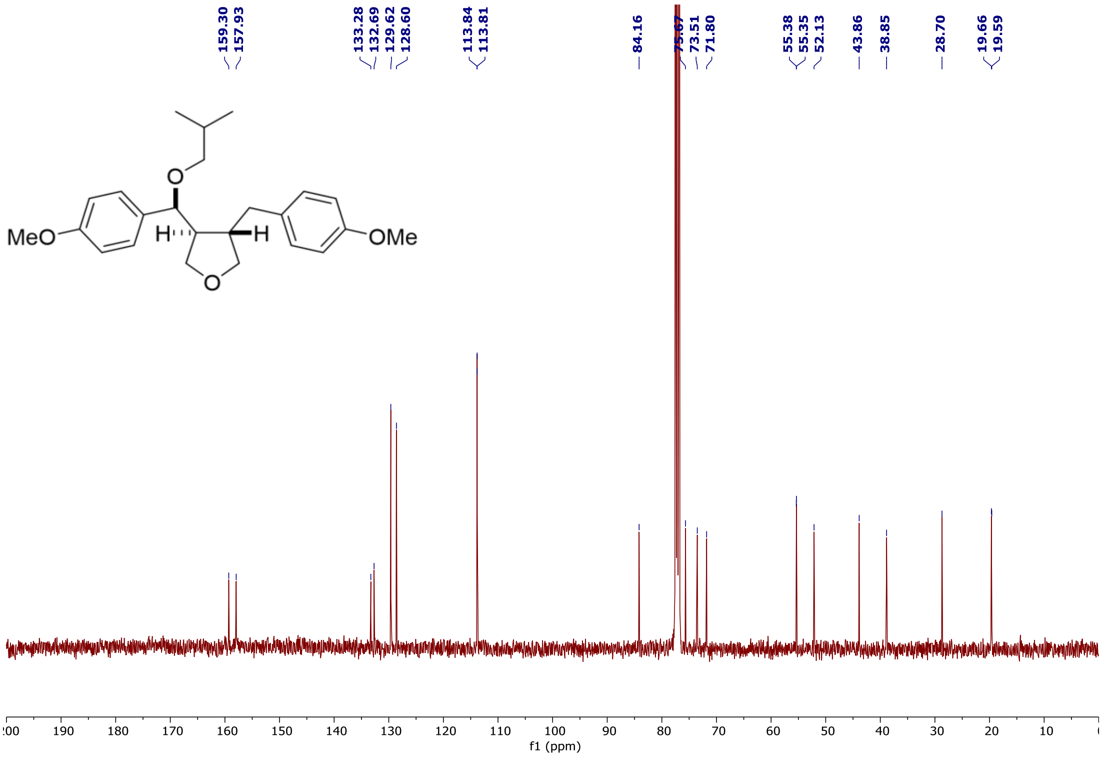

Supplementary Figure 168. ^{1}H NMR spectrum of compound **24f-A** (400 MHz, $CDCl_3$)

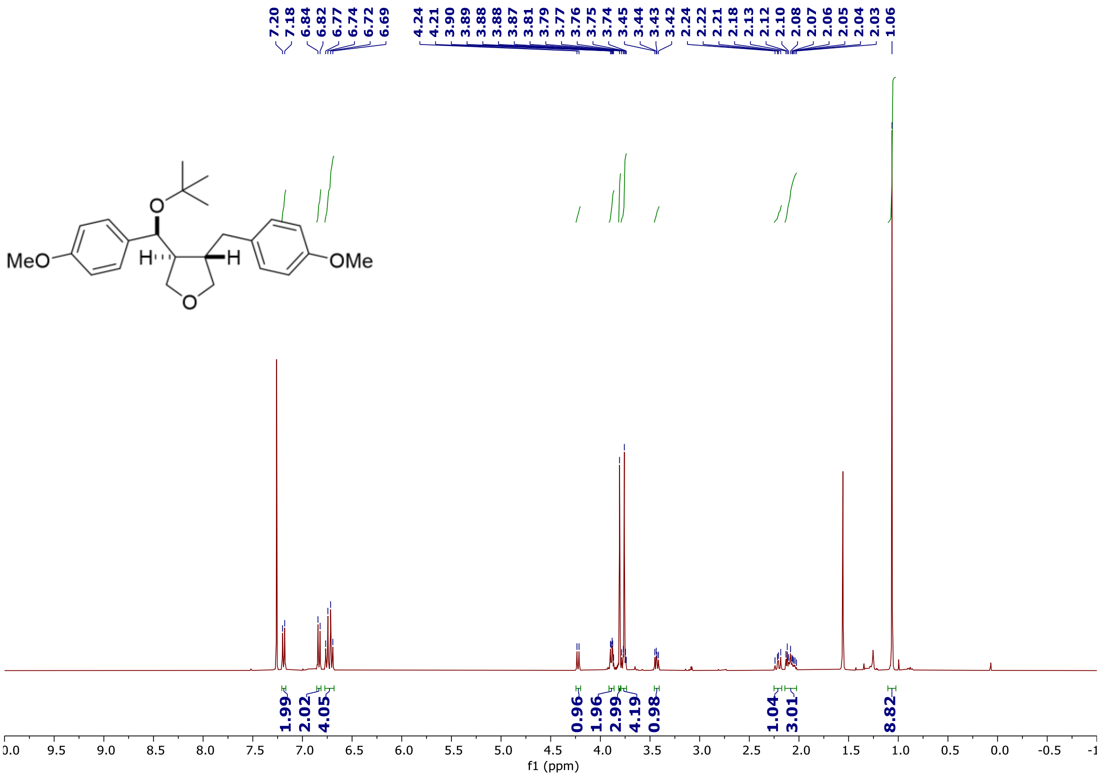

Supplementary Figure 169. ^{13}C NMR spectrum of compound **24f-A** (101 MHz, $CDCl_3$)

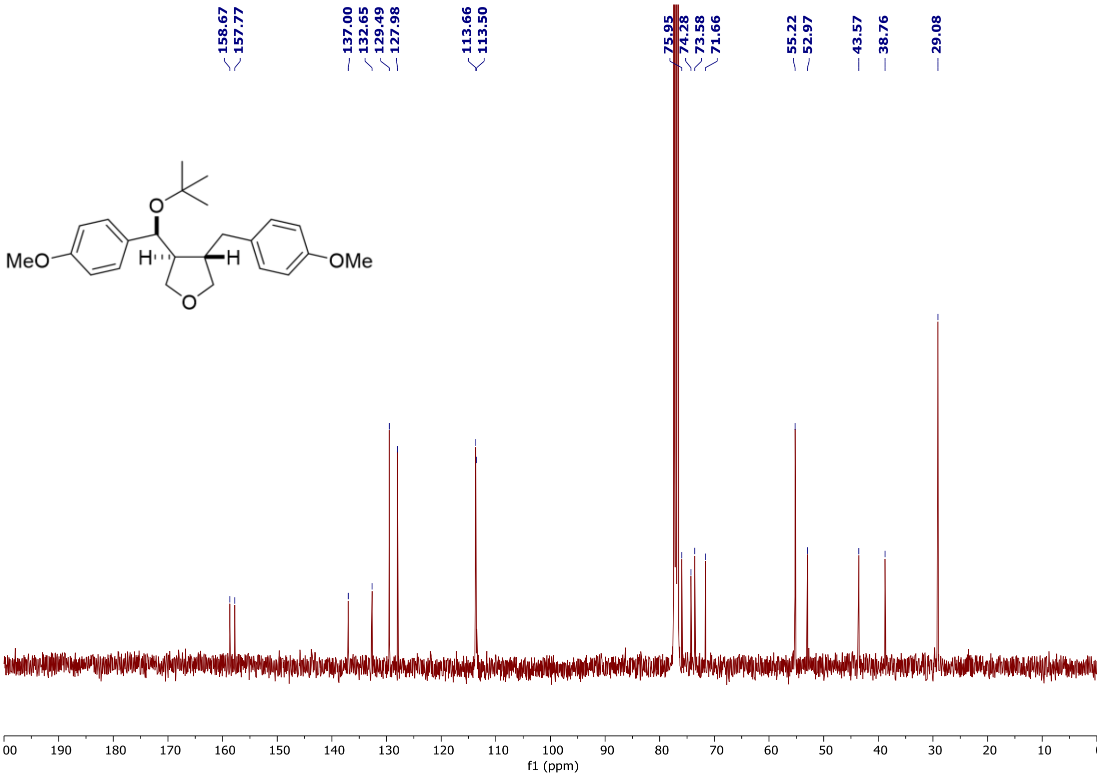

Supplementary Figure 170. ^{1}H NMR spectrum of compound **24g-A** (400 MHz, $CDCl_3$)

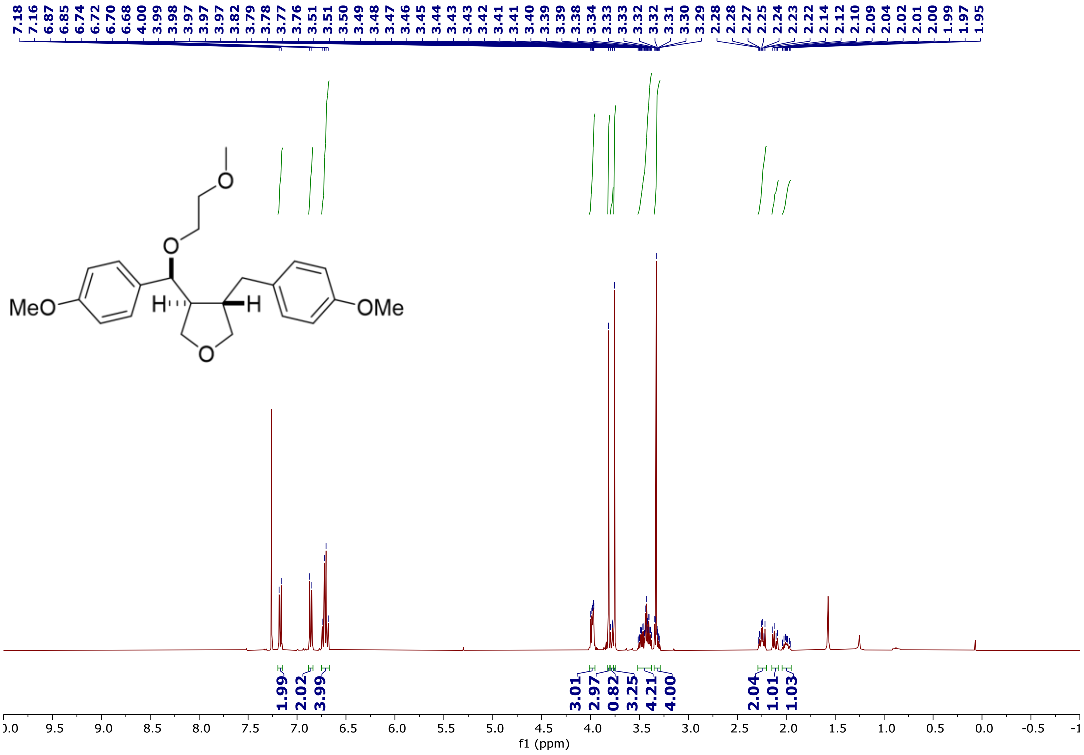

Supplementary Figure 171. ^{13}C NMR spectrum of compound **24g-A** (101 MHz, $CDCl_3$)

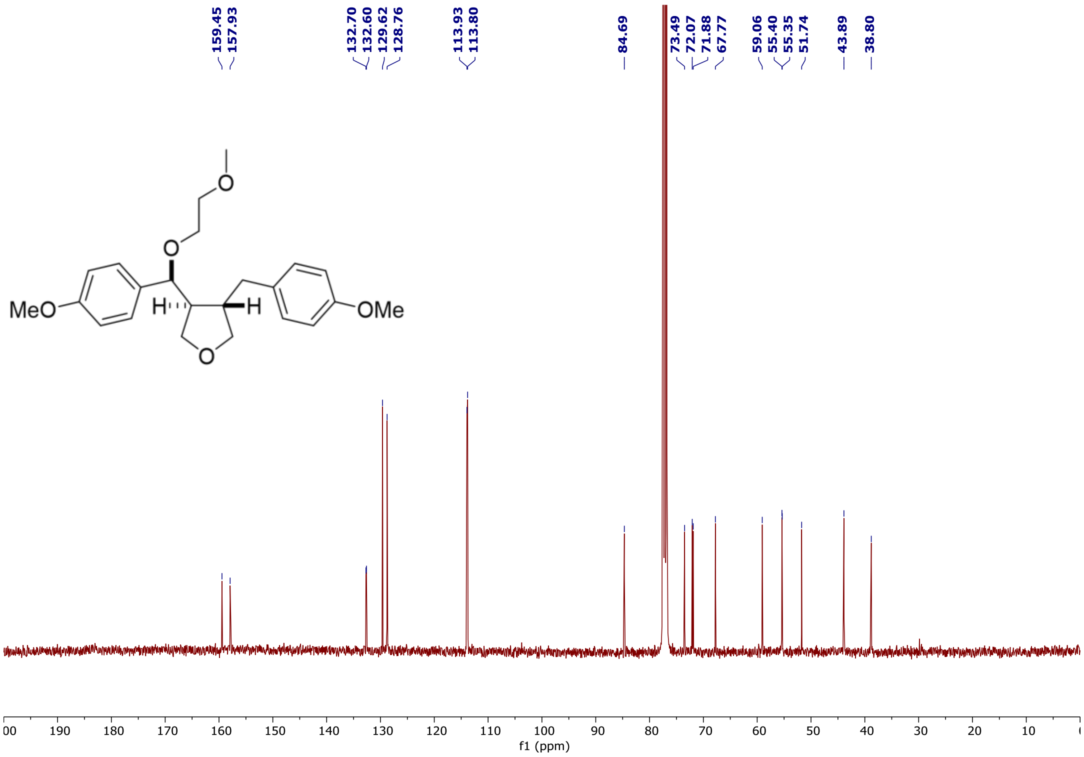

Supplementary Figure 172. ^{1}H NMR spectrum of compound **24h-A** (400 MHz, $CDCl_3$)

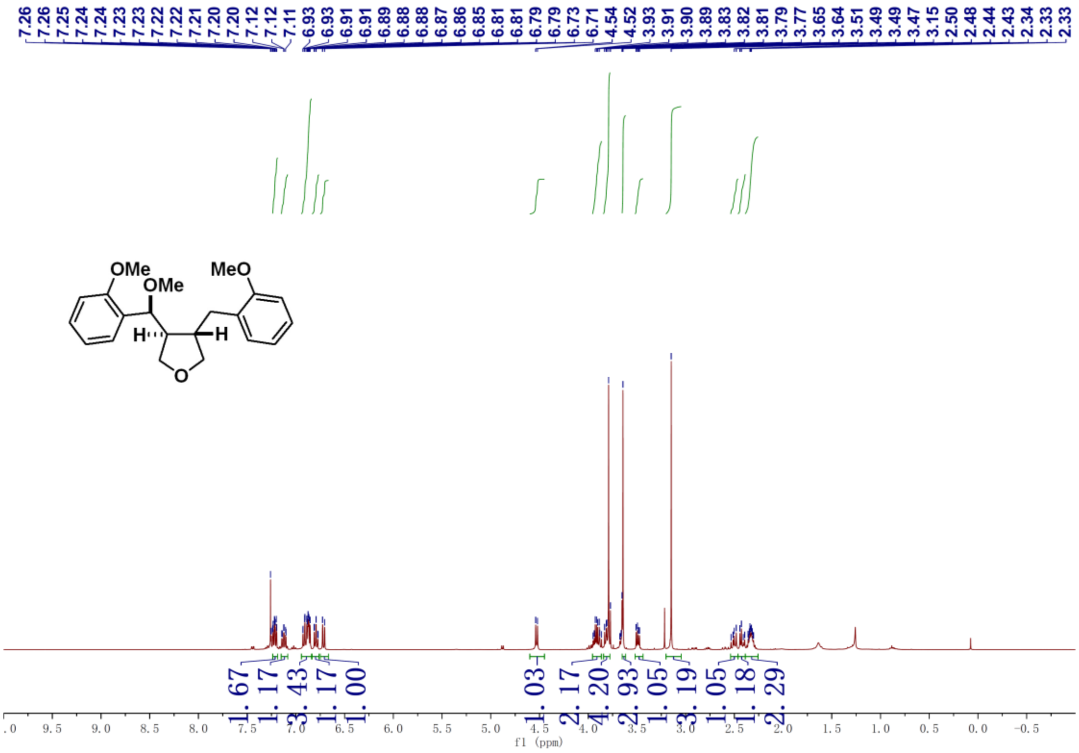

Supplementary Figure 173. ^{13}C NMR spectrum of compound **24h-A** (101 MHz, $CDCl_3$)

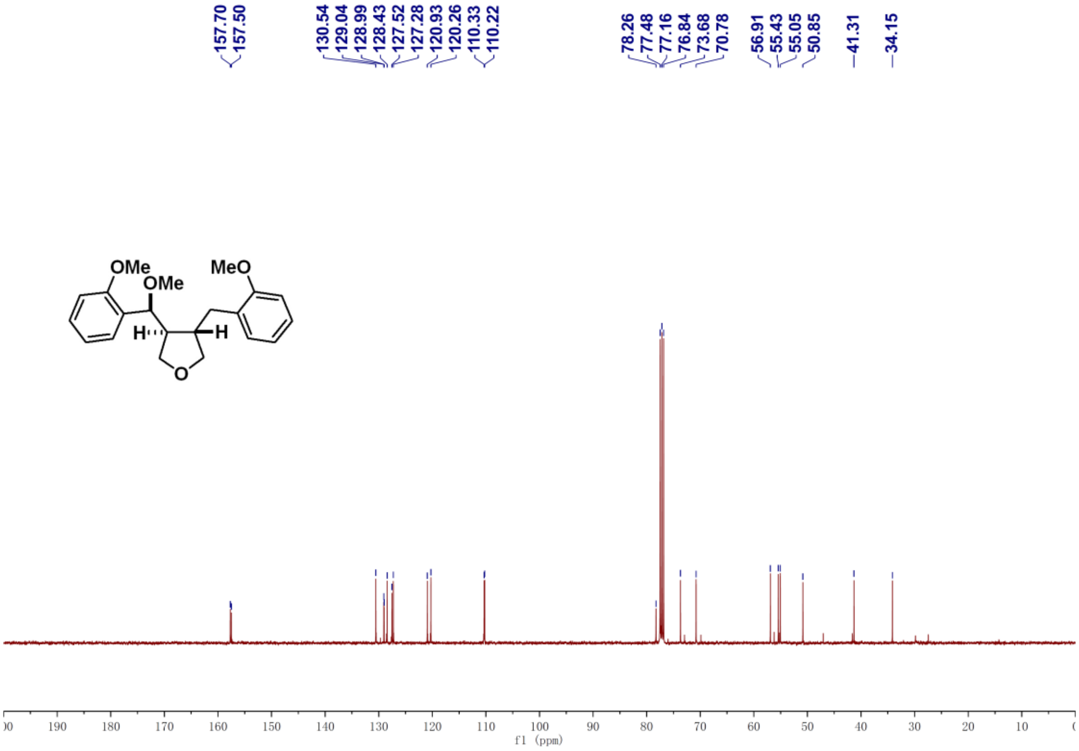

Supplementary Figure 174. ^{1}H NMR spectrum of compound **24i-A** (400 MHz, $CDCl_3$)

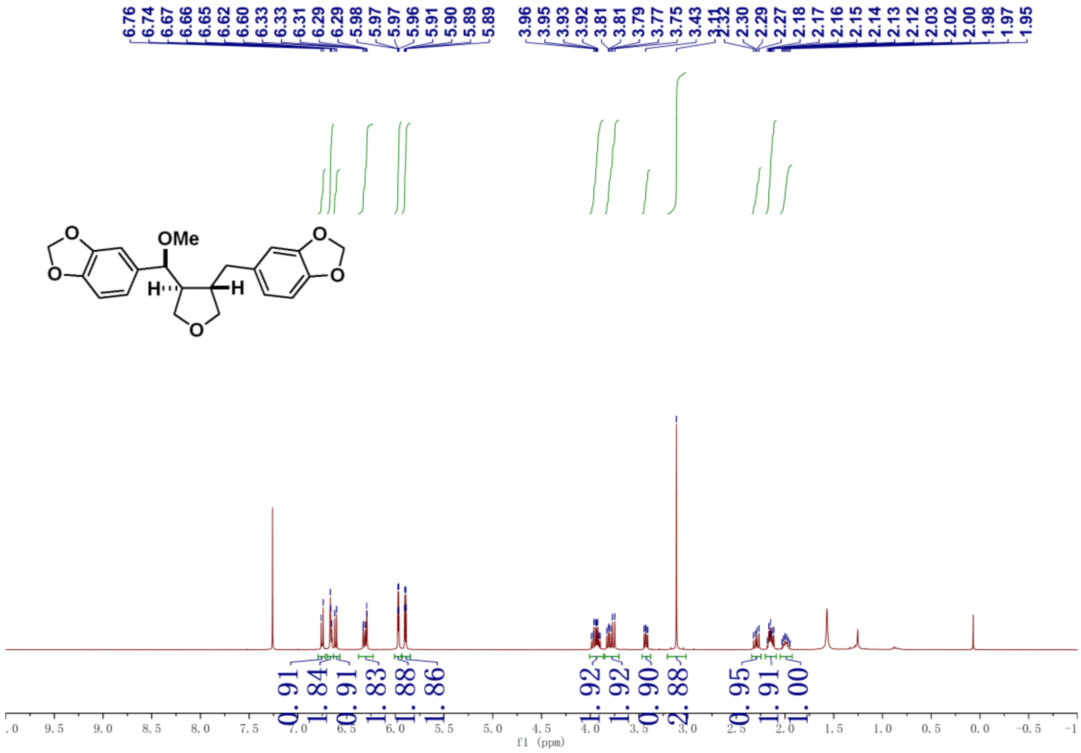

Supplementary Figure 175. ^{13}C NMR spectrum of compound **24i-A** (101 MHz, $CDCl_3$)

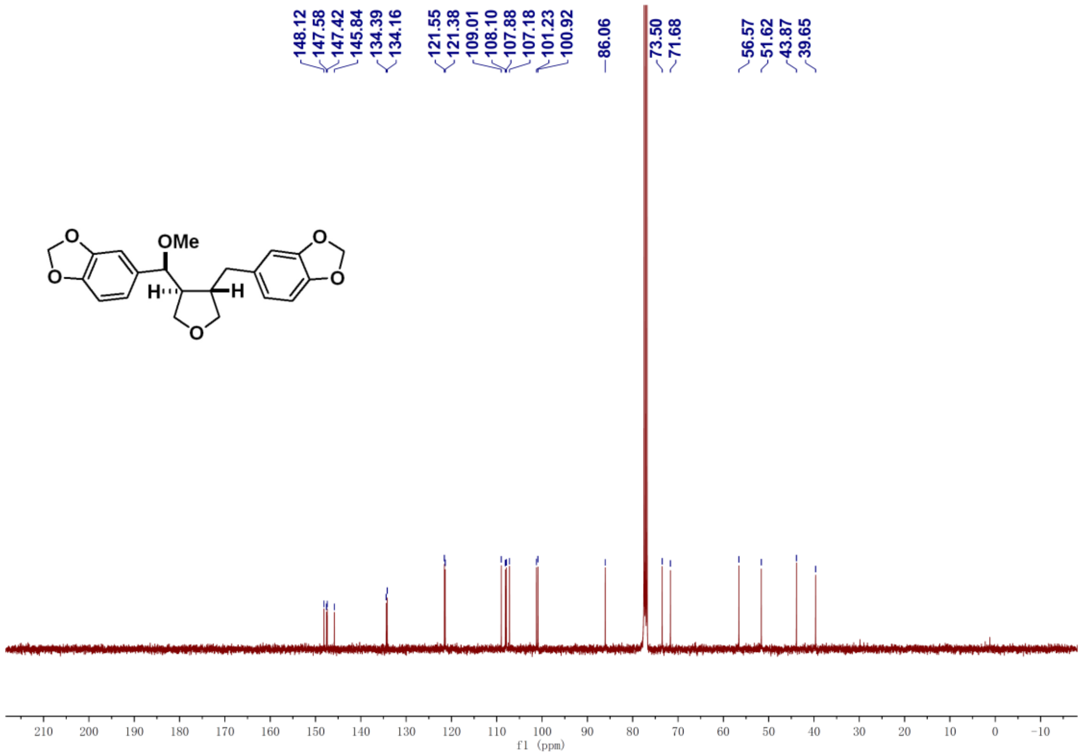

Supplementary Figure 176. ^{1}H NMR spectrum of compound **24j-A** (400 MHz, $CDCl_3$)

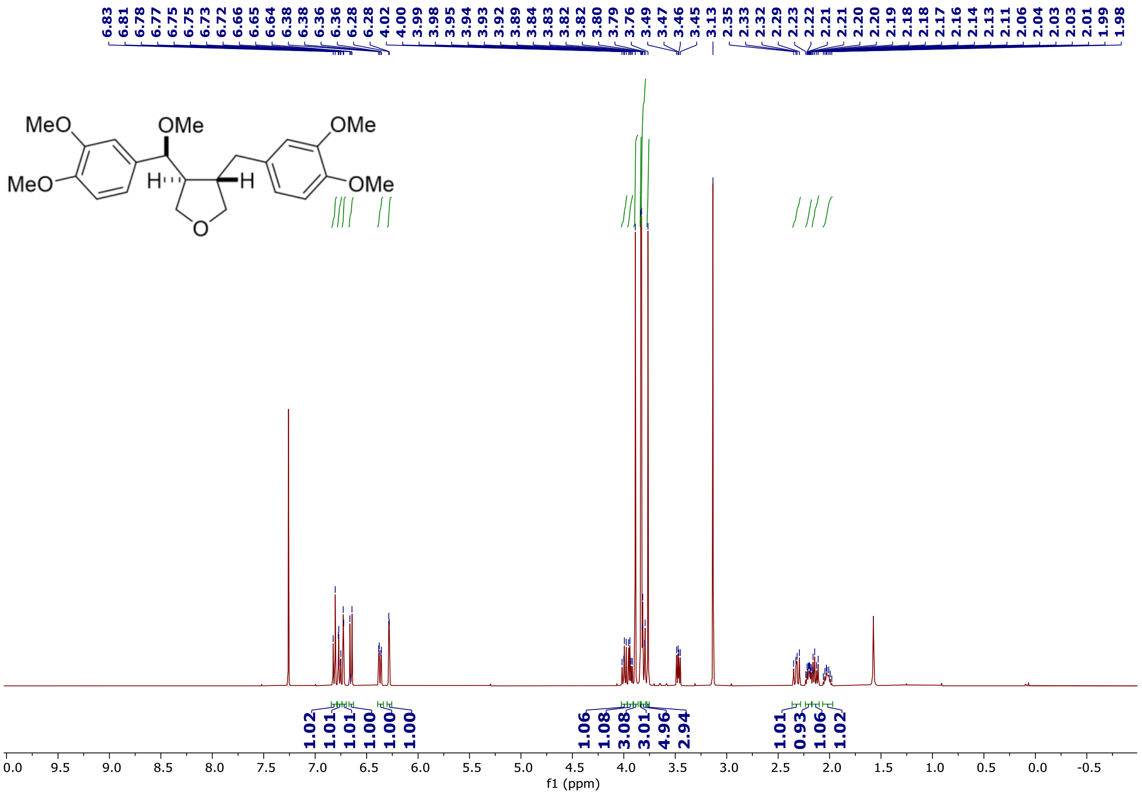

Supplementary Figure 177. ^{13}C NMR spectrum of compound **24j-A** (101 MHz, $CDCl_3$)

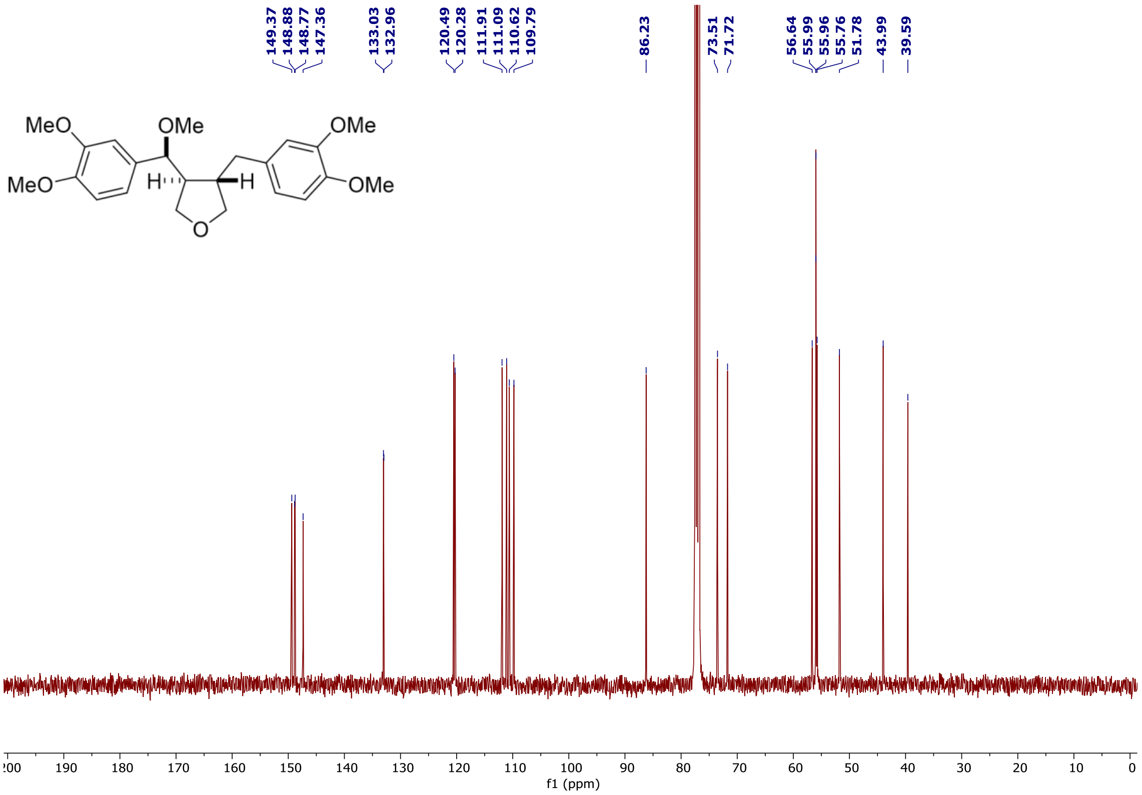

Supplementary Figure 178. 2D NMR spectra of compound **24j-A** ($CDCl_3$)

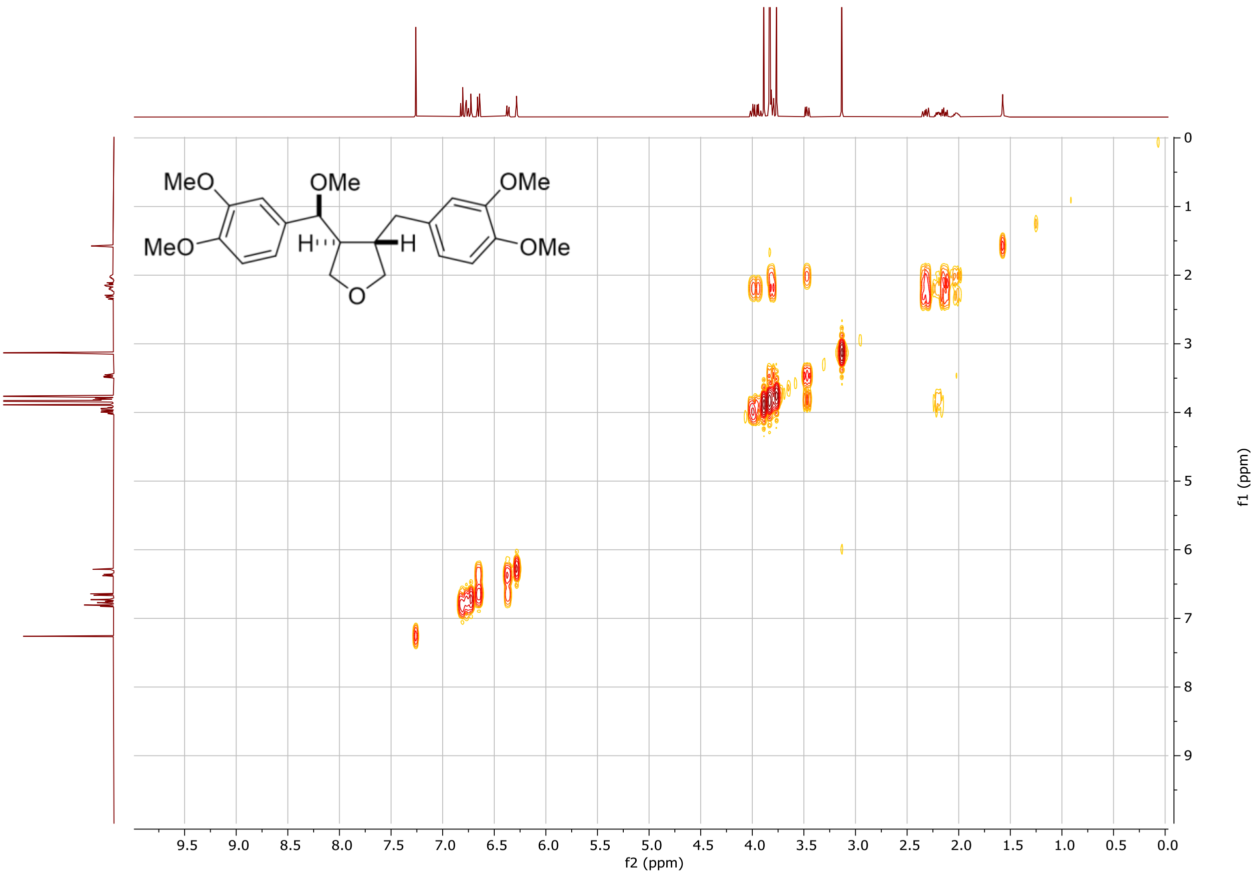

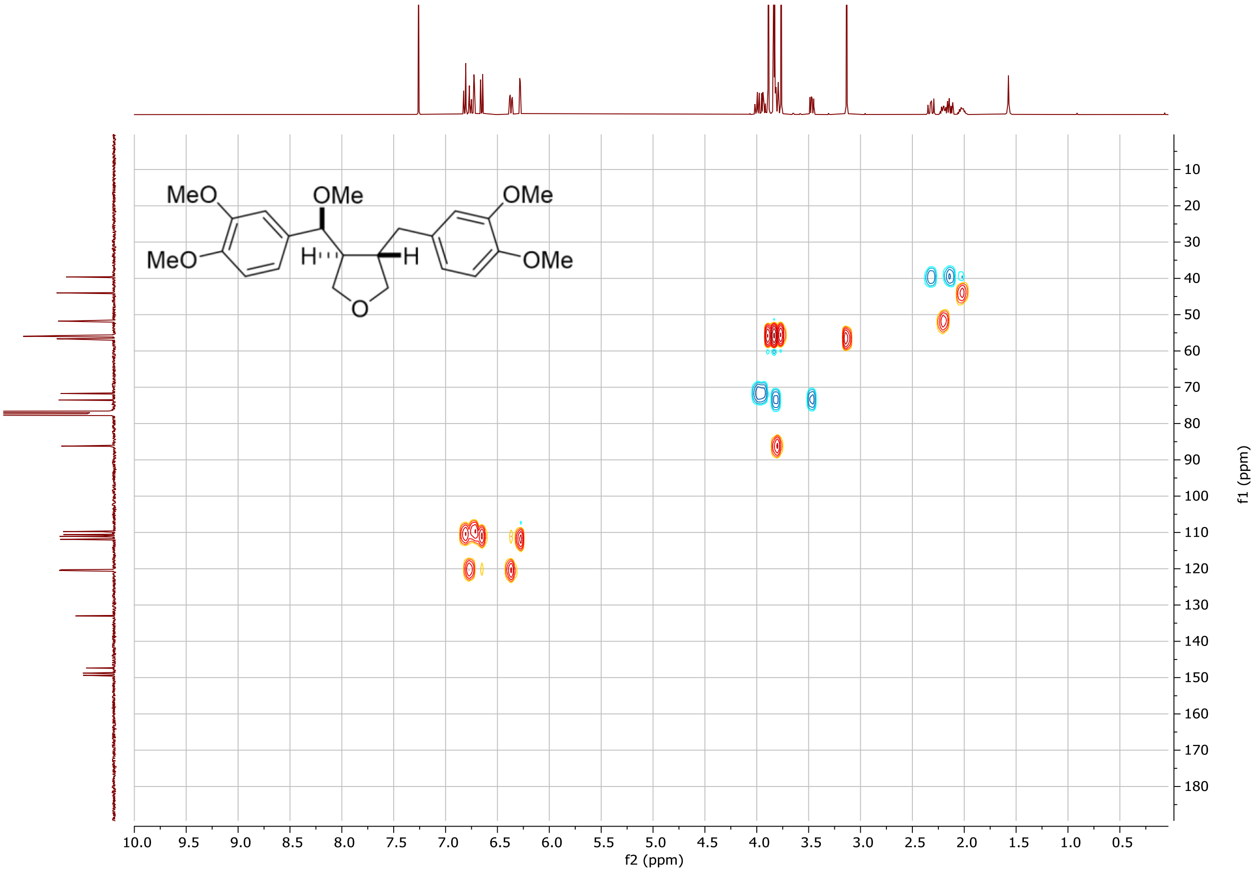

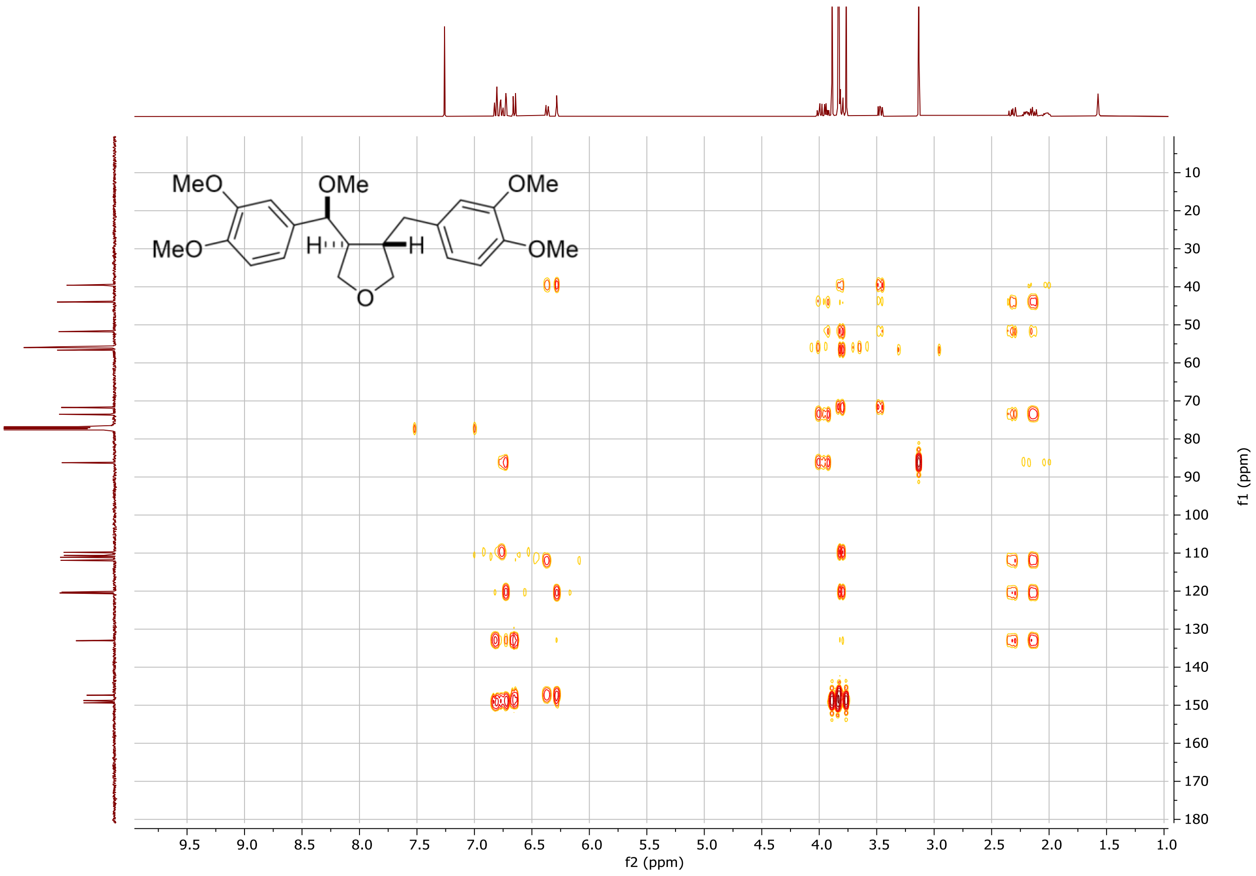
MeO
OMe
OMe
MeO
H
H
O
OMe
f1 (ppm)
f2 (ppm)

Supplementary Figure 179. ^{1}H NMR spectrum of compound **24k-A** (400 MHz, $CDCl_3$)

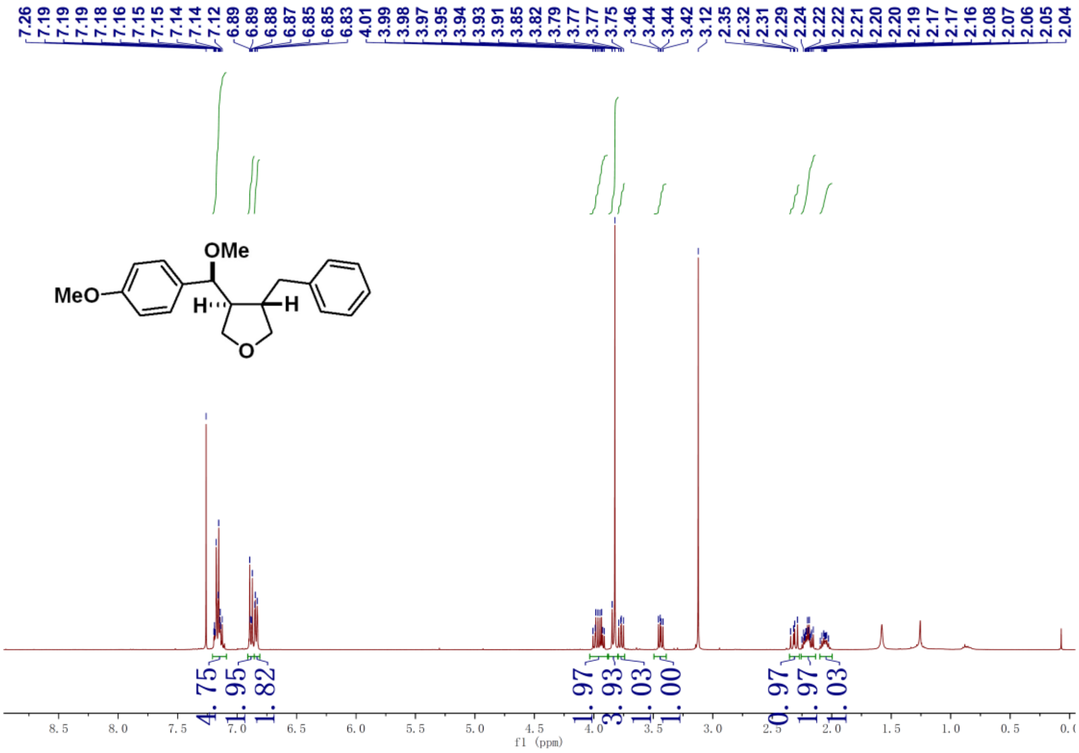

Supplementary Figure 180. ^{13}C NMR spectrum of compound **24k-A** (101 MHz, $CDCl_3$)

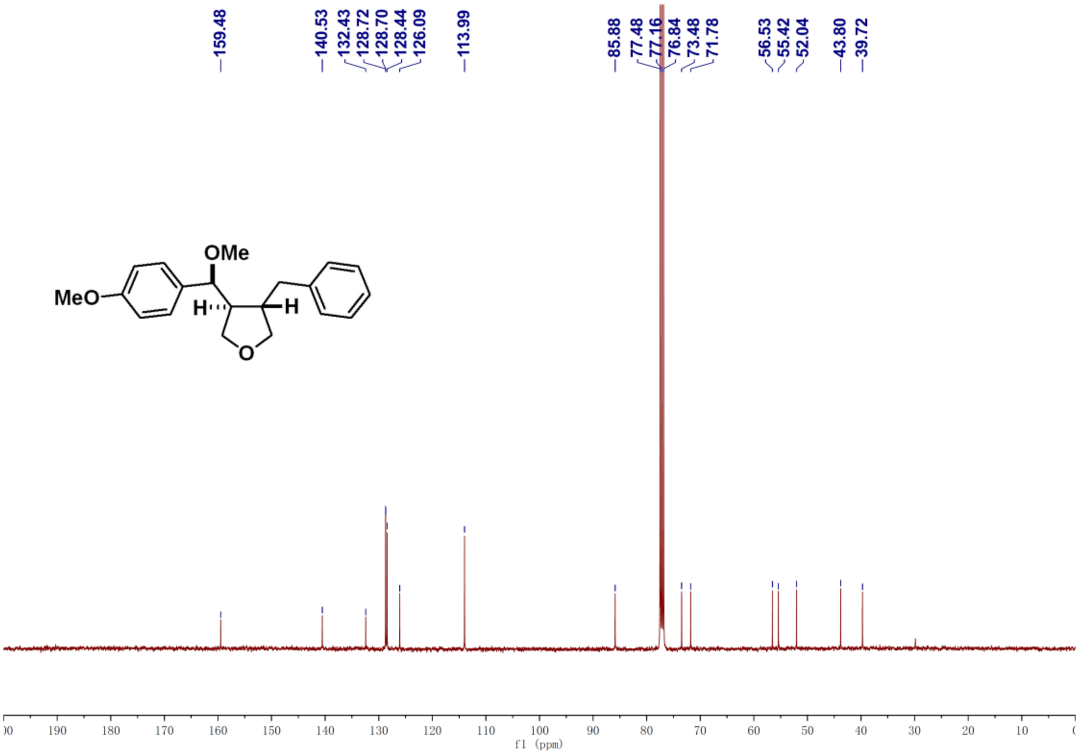

Supplementary Figure 181. 2D NMR spectrum of compound **24k-A** ($CDCl_3$)

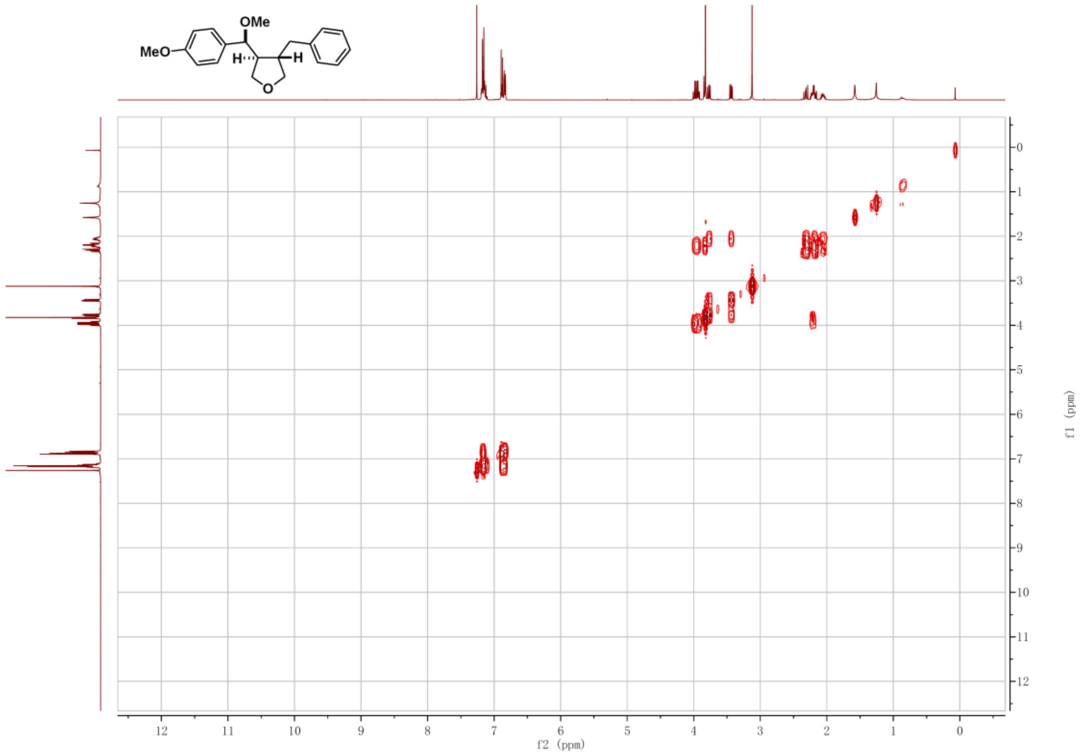

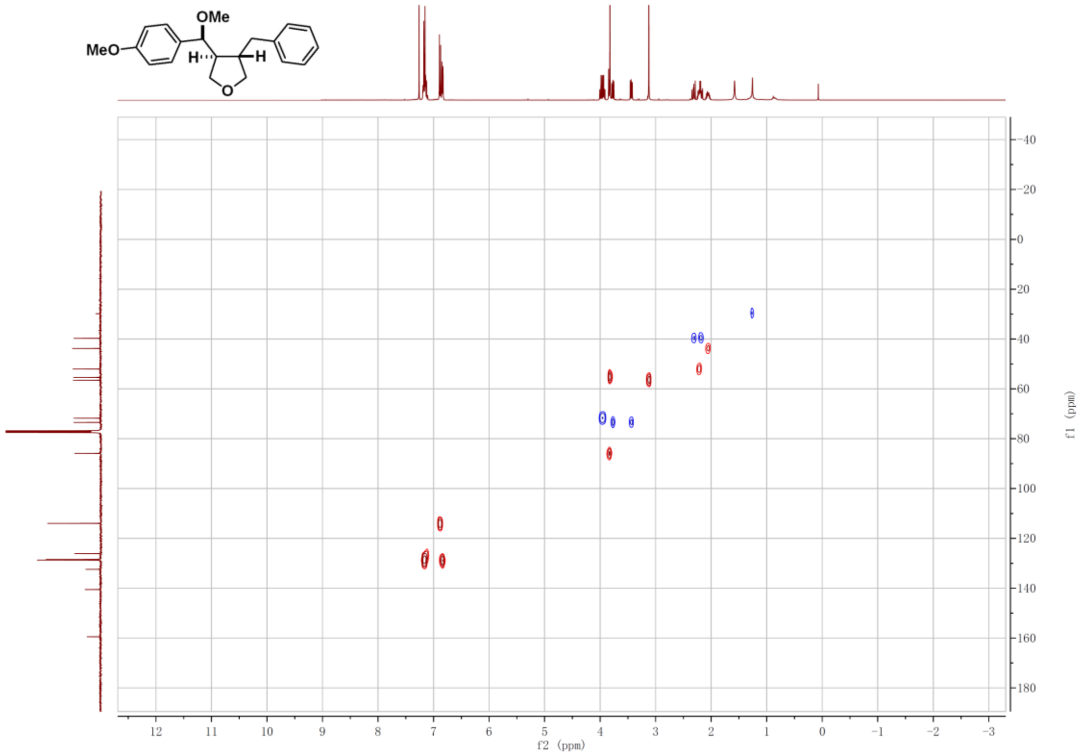

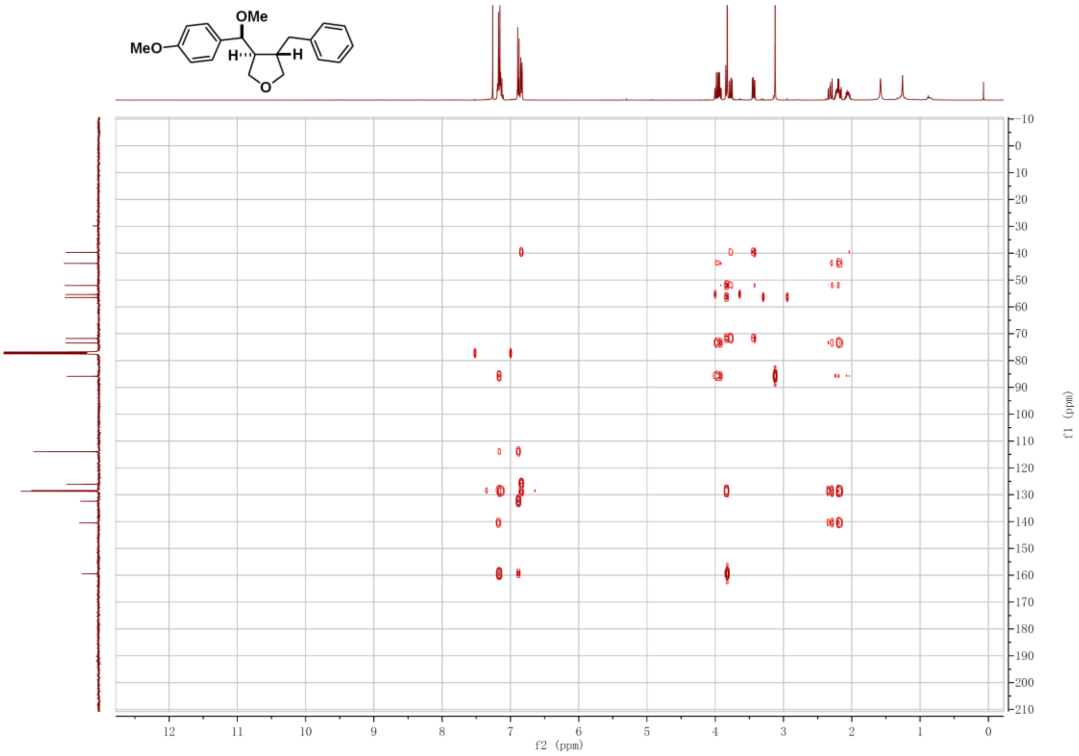

OMe
MeO
H
H
O
f2 (ppm)
f1 (ppm)

Supplementary Figure 182. ^{1}H NMR spectrum of compound **24l-A** (400 MHz, $CDCl_3$)

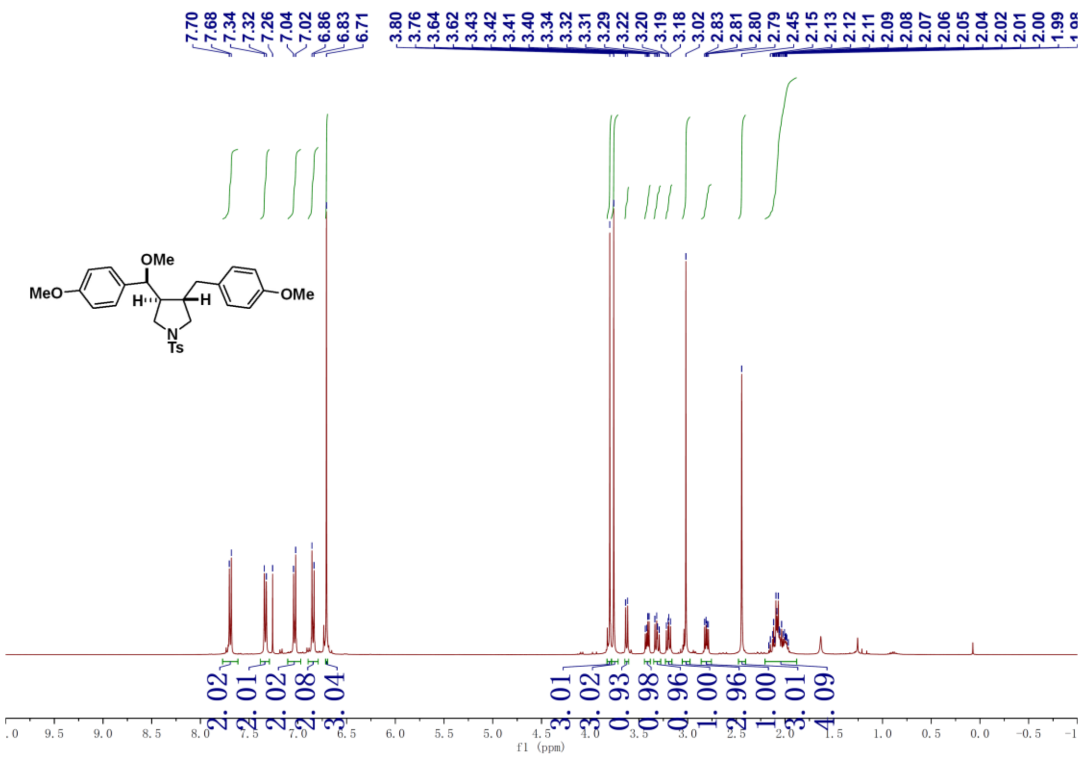

Supplementary Figure 183. ^{13}C NMR spectrum of compound **24l-A** (101 MHz, $CDCl_3$)

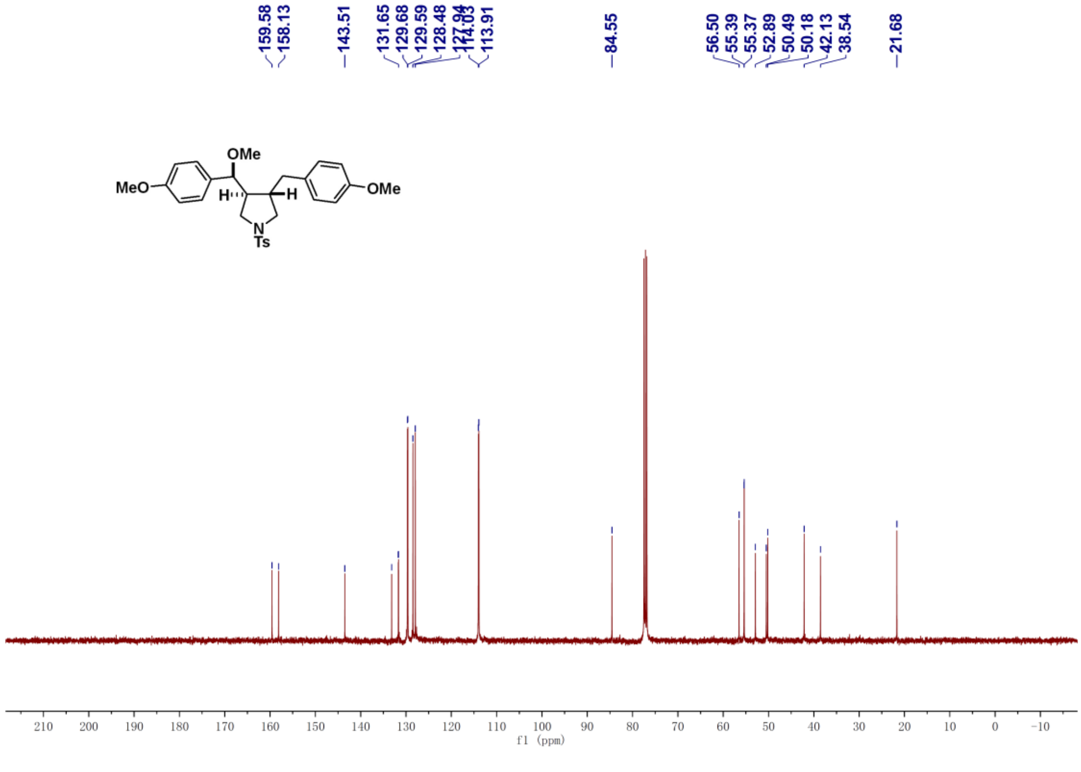

Supplementary Figure 184. 2D NMR spectra of compound **24l-A** ($CDCl_3$)

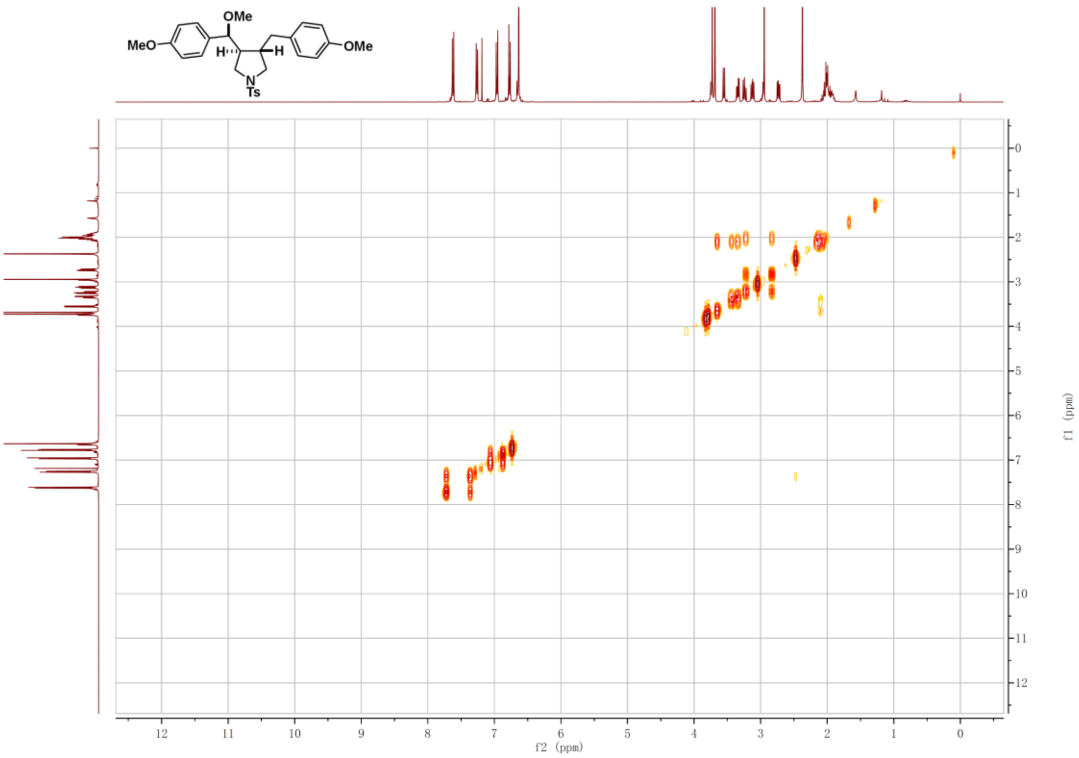

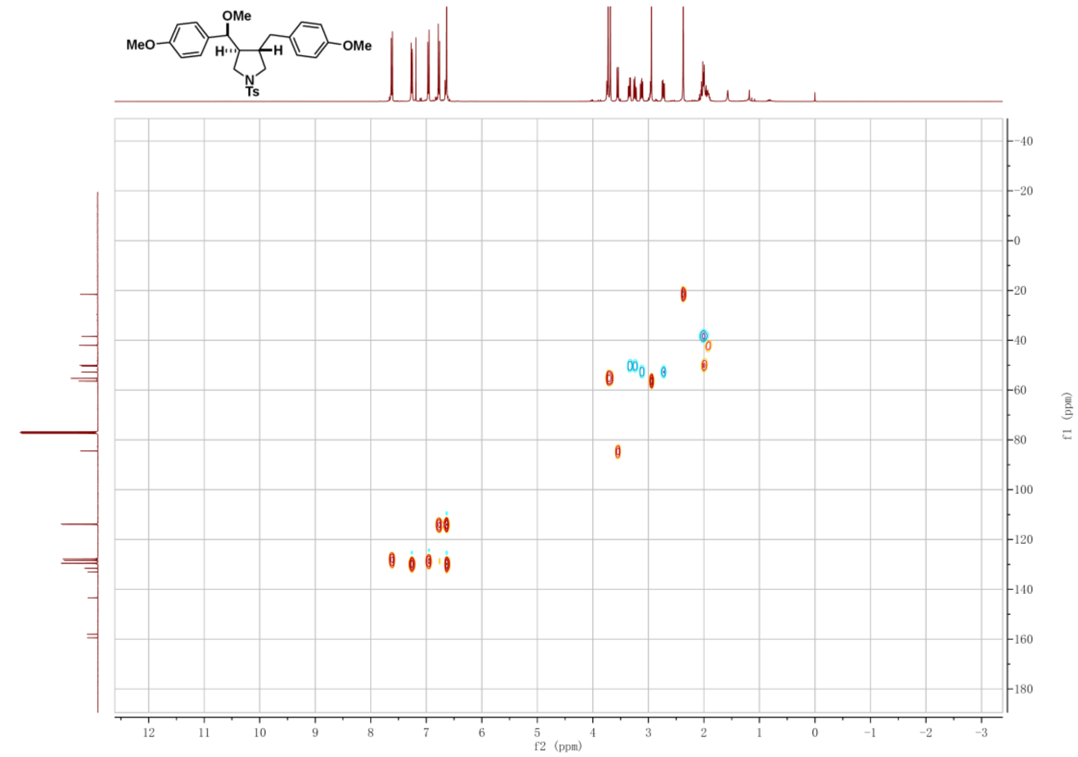

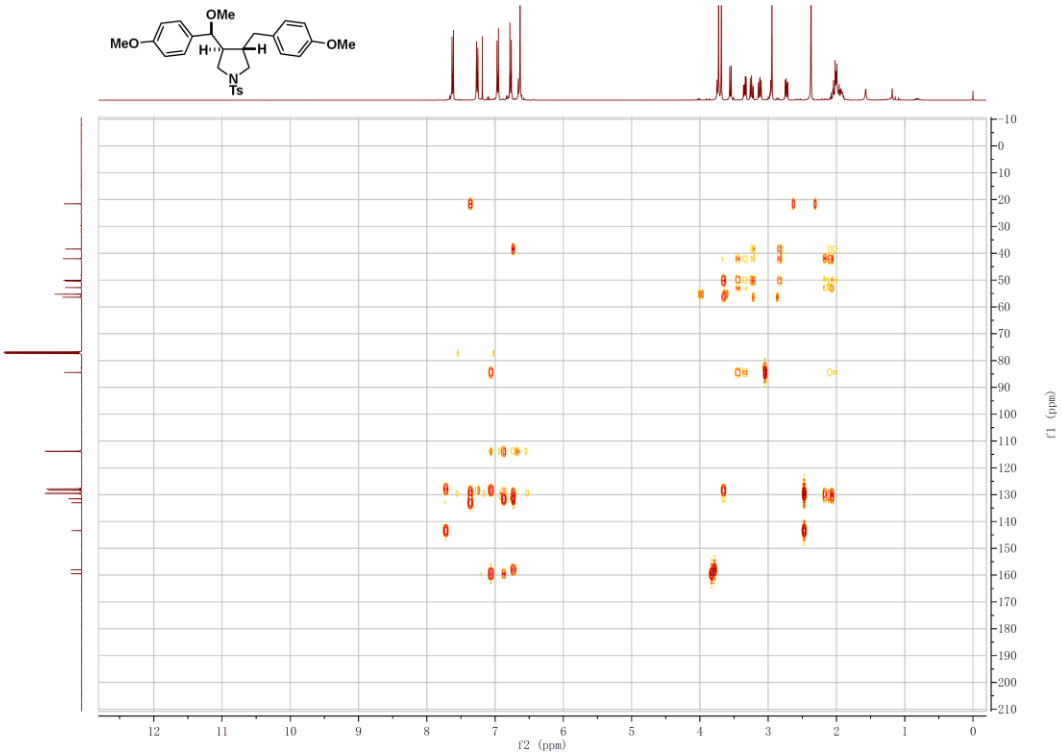
OMe
MeO
OMe
H
H
N
Ts
f2 (ppm)
f1 (ppm)

Supplementary Figure 185. ^{1}H NMR spectrum of compound **24m-A** (400 MHz, $CDCl_3$)

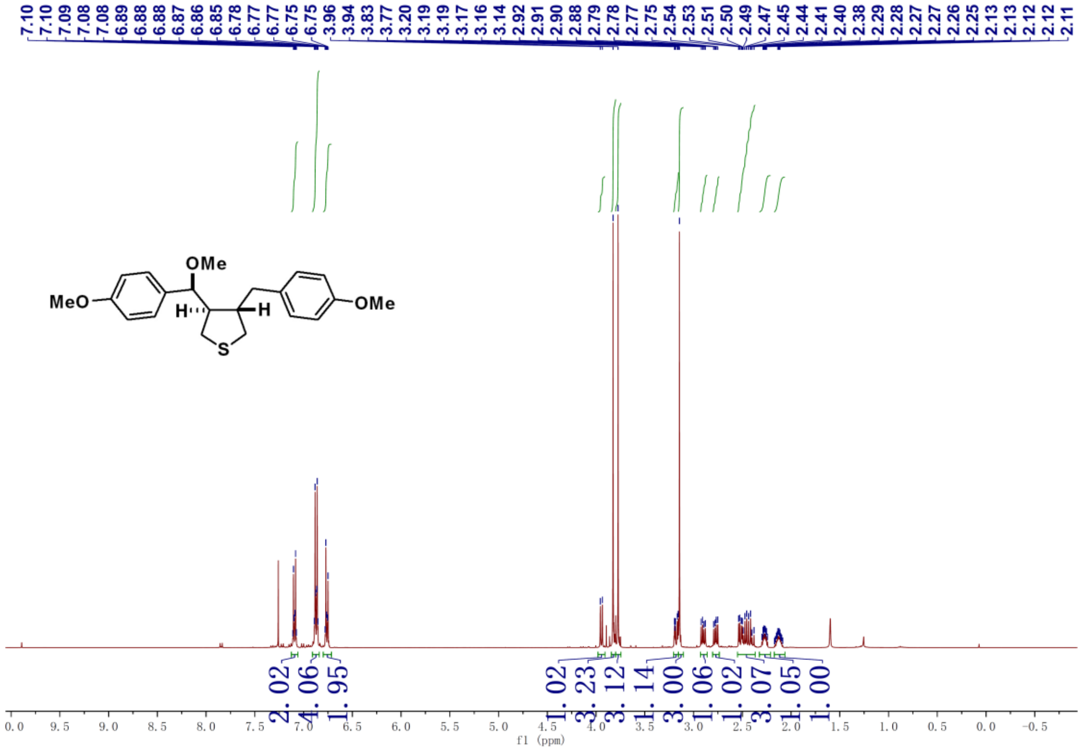

Supplementary Figure 186. ^{13}C NMR spectrum of compound **24m-A** (101 MHz, $CDCl_3$)

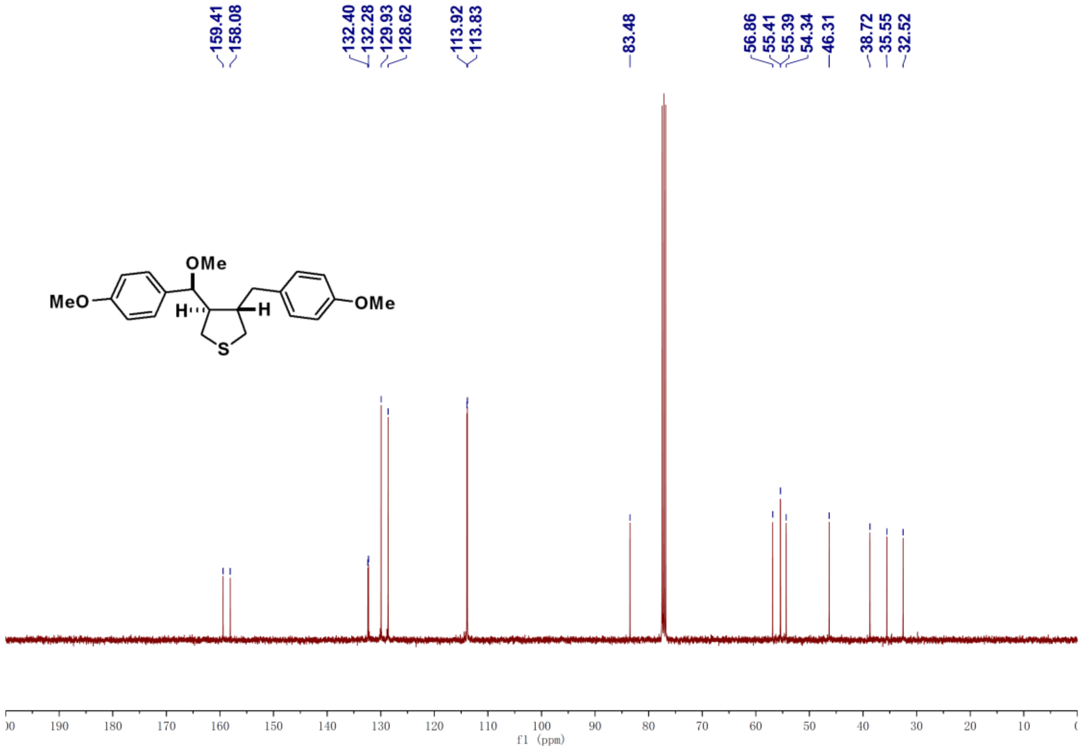

Supplementary Figure 187. ^{1}H NMR spectrum of compound **24n-A** (400 MHz, $CDCl_3$)

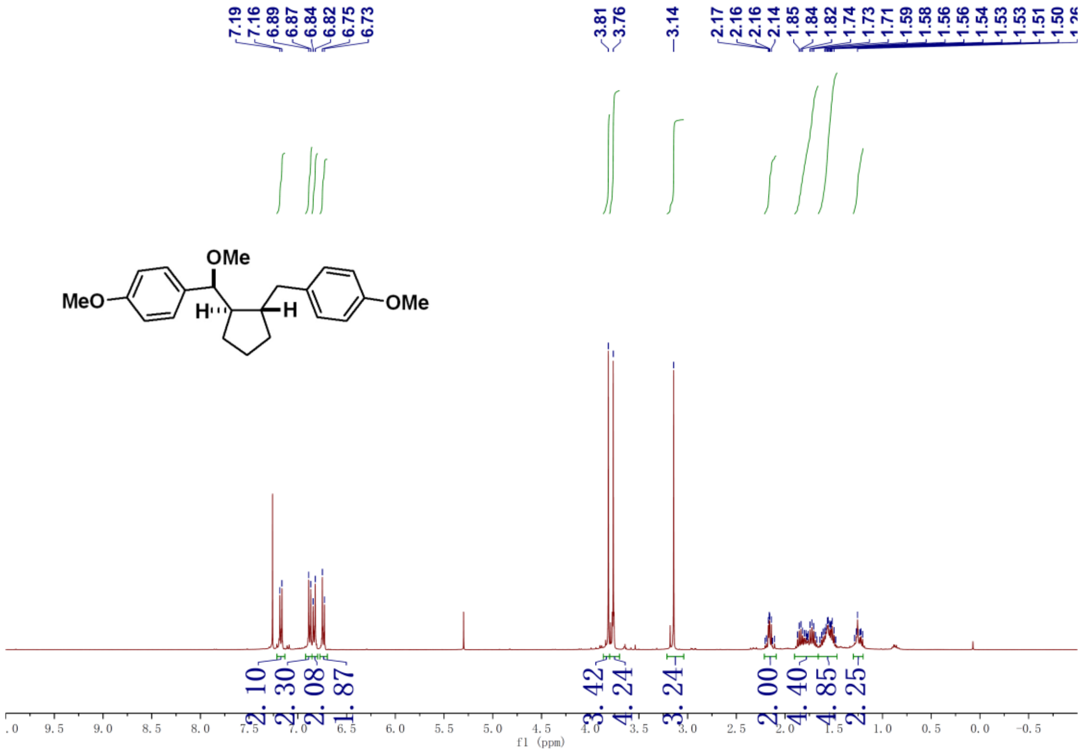

Supplementary Figure 188. ^{13}C NMR spectrum of compound **24n-A** (101 MHz, $CDCl_3$)

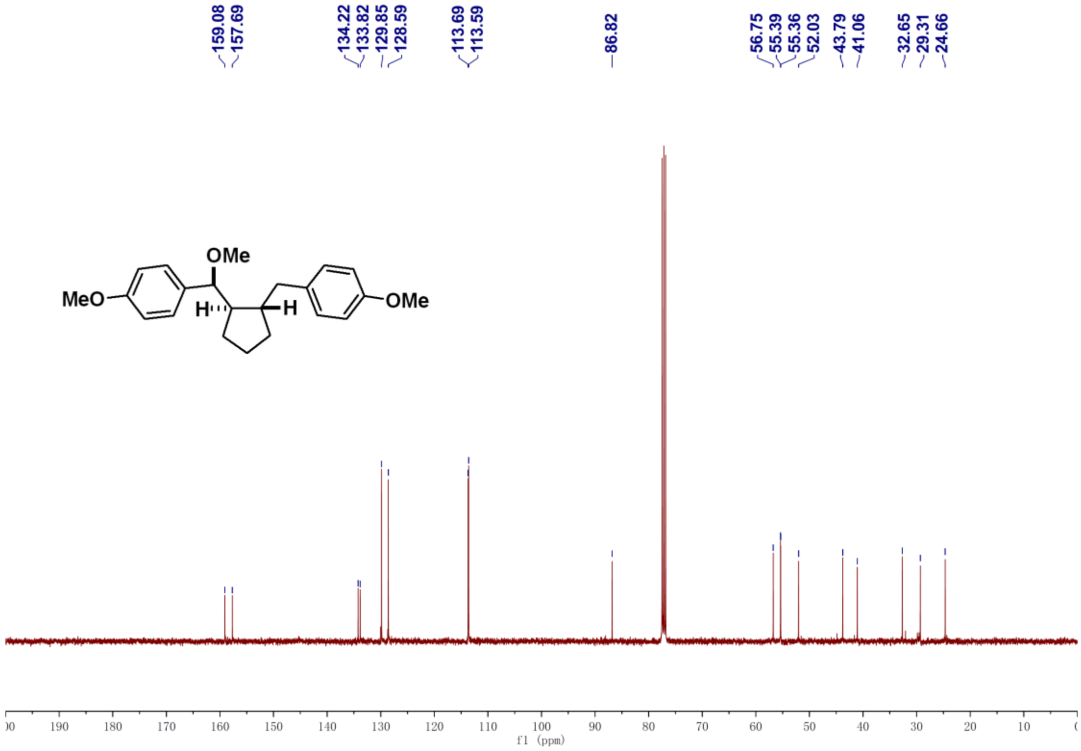

Supplementary Figure 189. ^{1}H NMR spectrum of compound **24o-A** (400 MHz, $CDCl_3$)

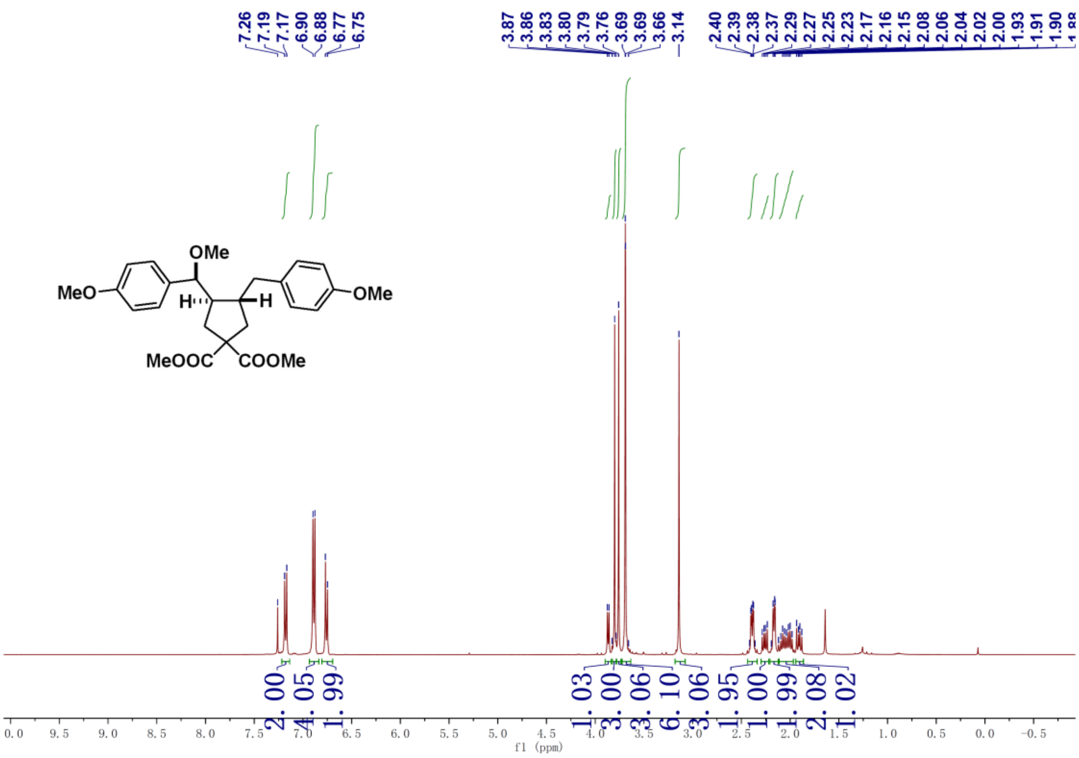

Supplementary Figure 190. ^{13}C NMR spectrum of compound **24o-A** (101 MHz, $CDCl_3$)

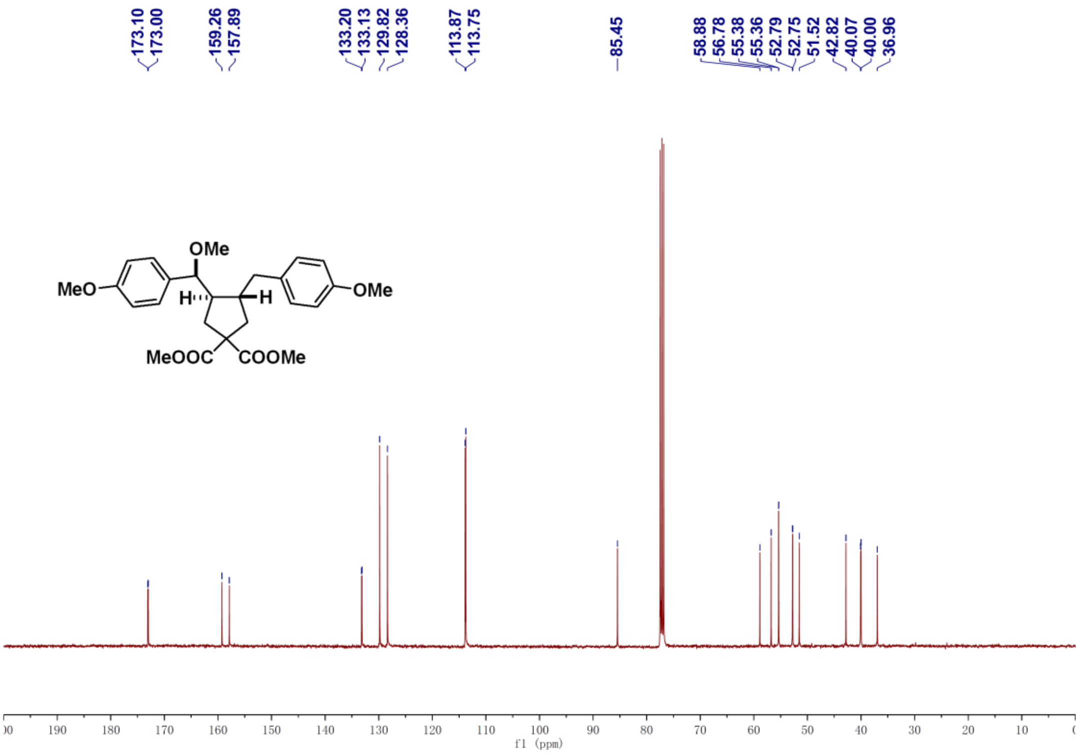

Supplementary Figure 191. ^{1}H NMR spectrum of compound **24p-A** (400 MHz, $CDCl_3$)

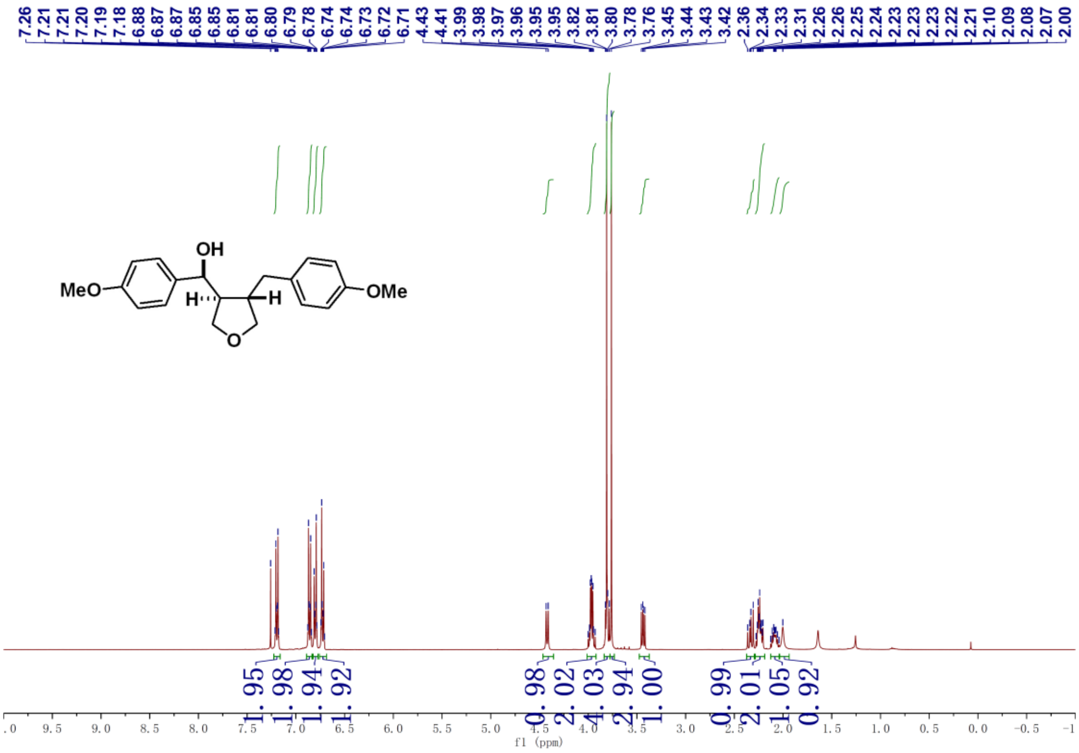

Supplementary Figure 192. ^{13}C NMR spectrum of compound **24p-A** (101 MHz, $CDCl_3$)

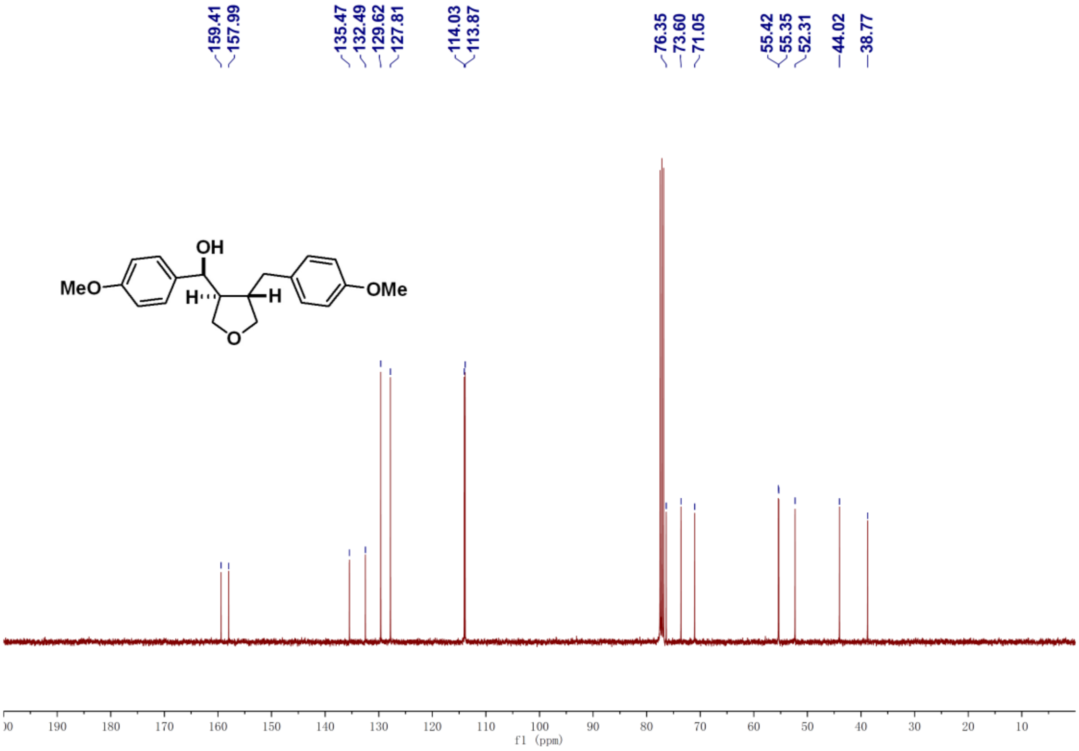

Supplementary Figure 193. ^{1}H NMR spectrum of compound **19b** (400 MHz, $CDCl_3$)

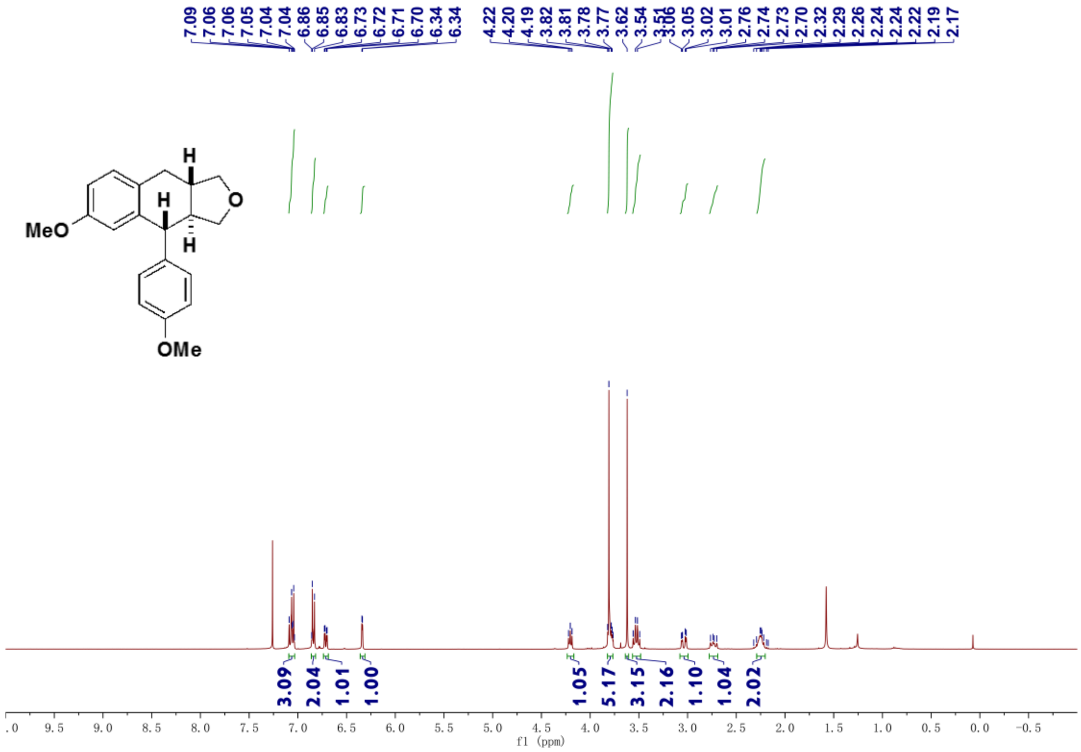

Supplementary Figure 194. ^{13}C NMR spectrum of compound **19b** (101 MHz, $CDCl_3$)

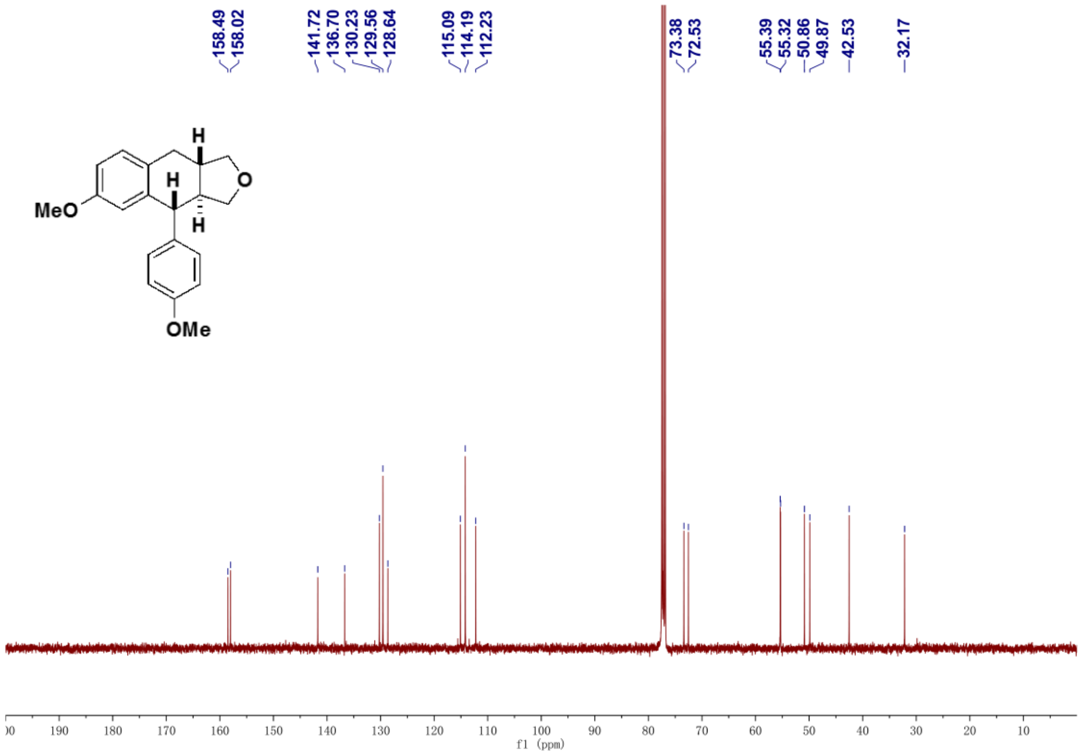

Supplementary Figure 195. 2D NMR spectrum of compound **19b** ($CDCl_3$)

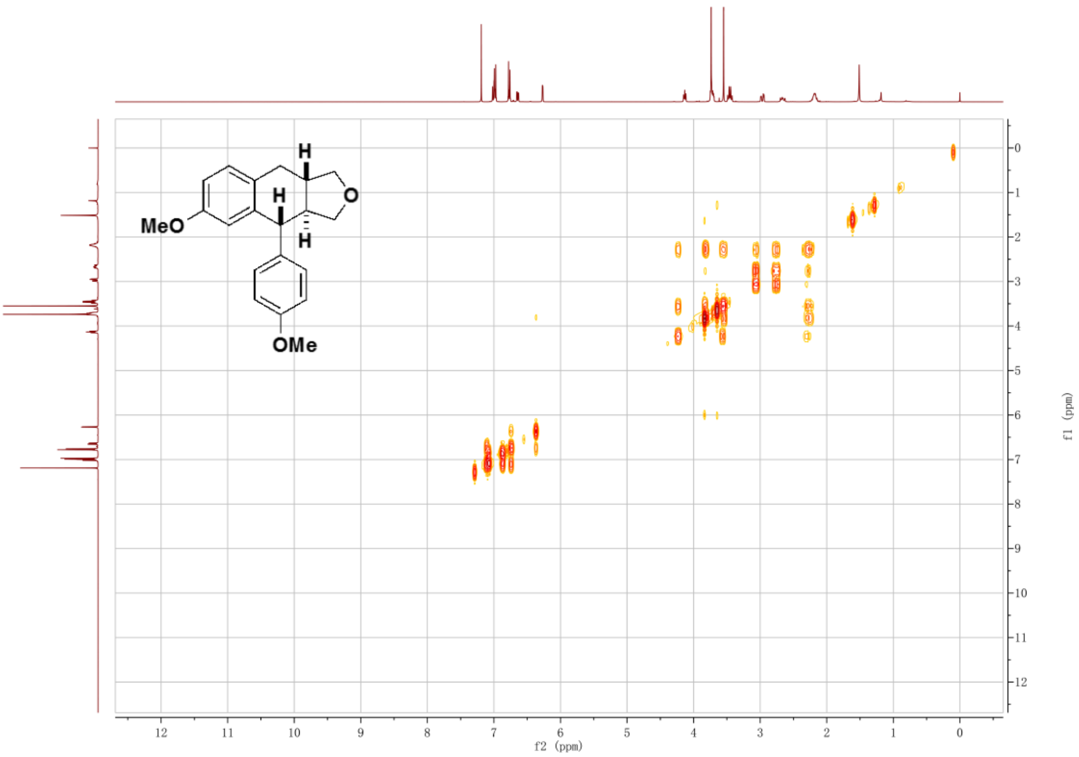

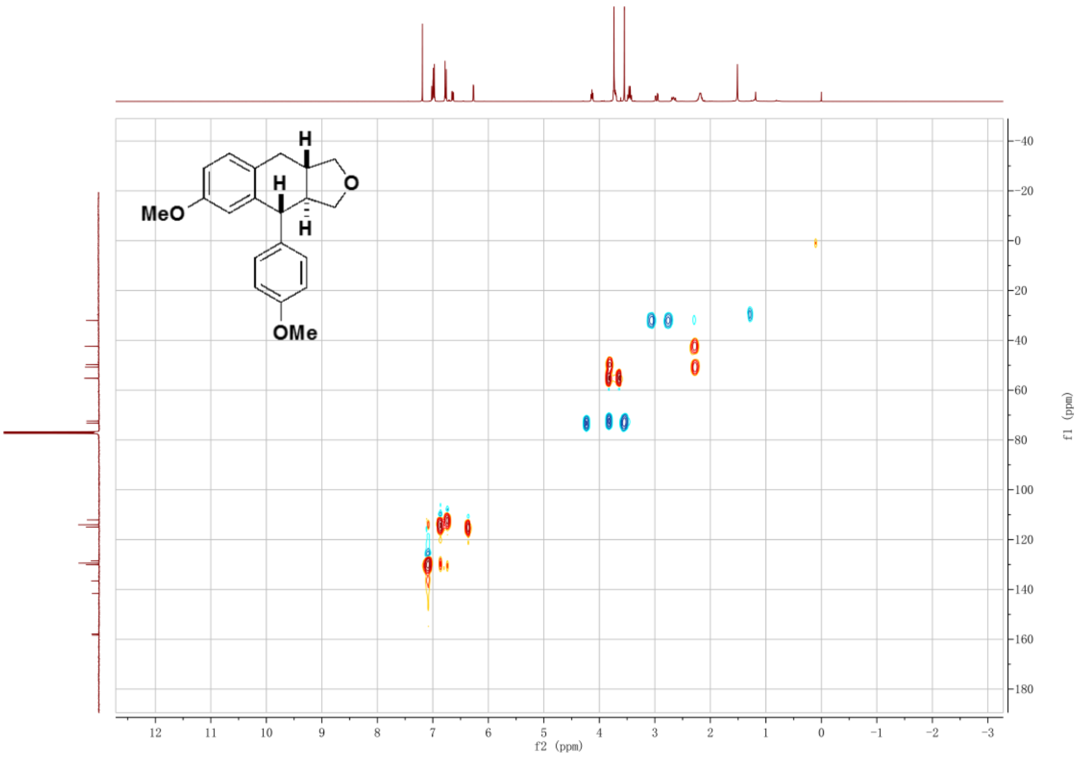

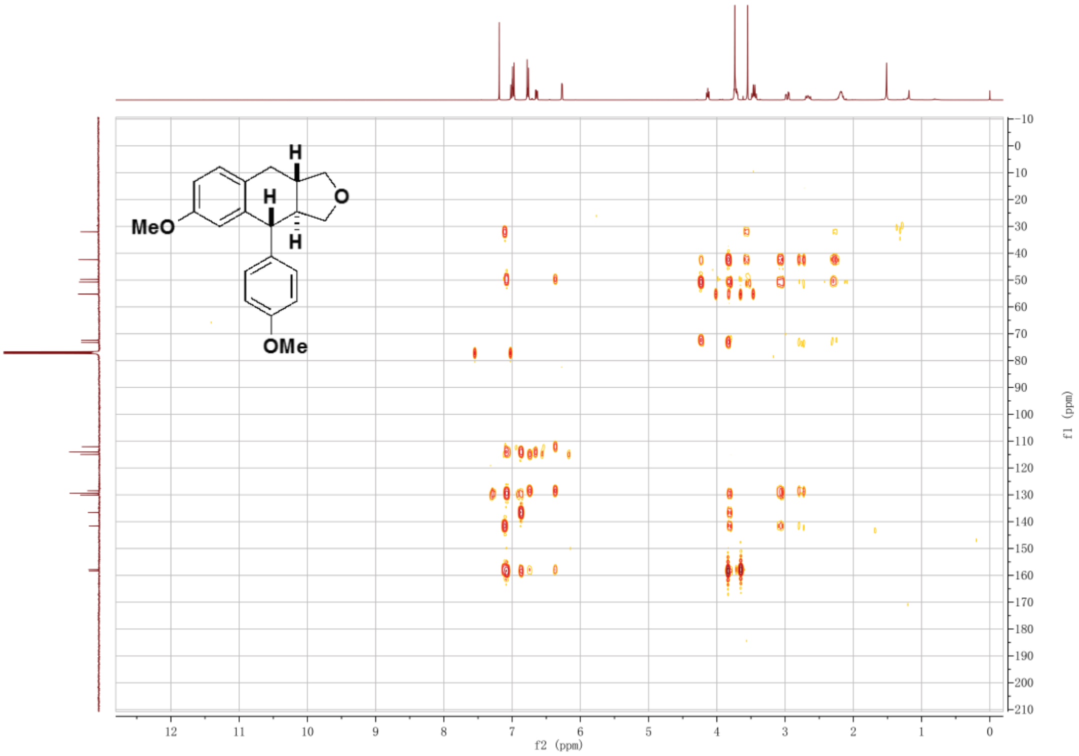
MeO
OMe
H
H
H
O
f2 (ppm)
f1 (ppm)

Supplementary Figure 196. ^{1}H NMR spectrum of compound **19c** (400 MHz, $CDCl_3$)

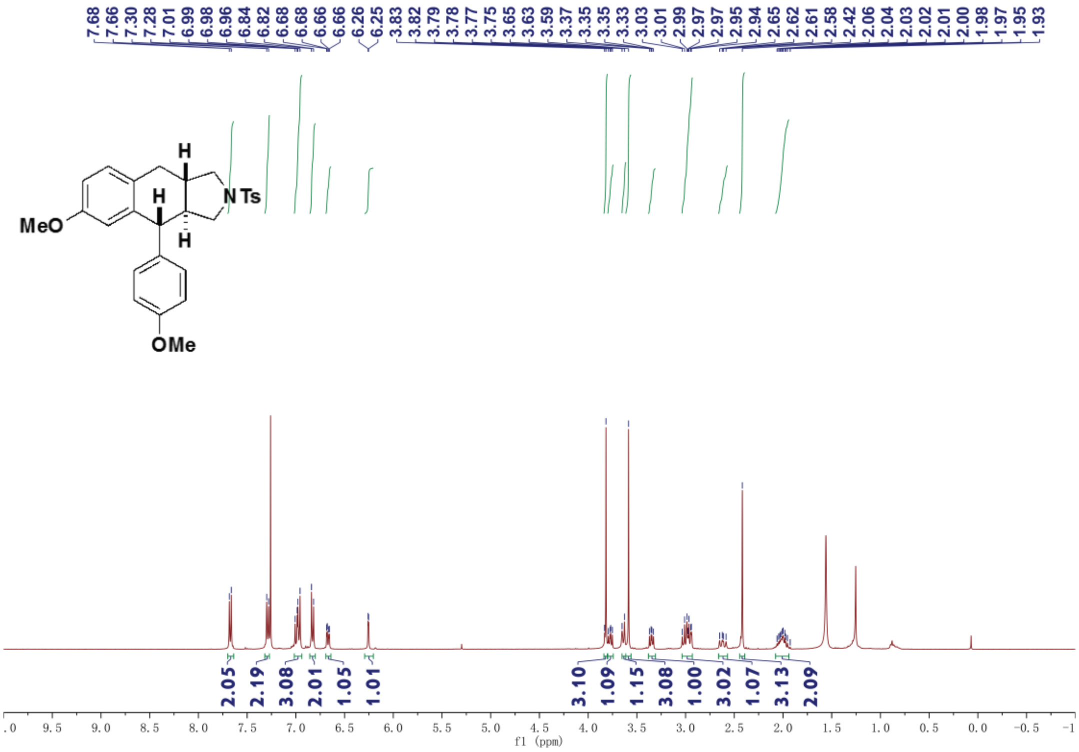

Supplementary Figure 197. ^{13}C NMR spectrum of compound **19c** (101 MHz, $CDCl_3$)

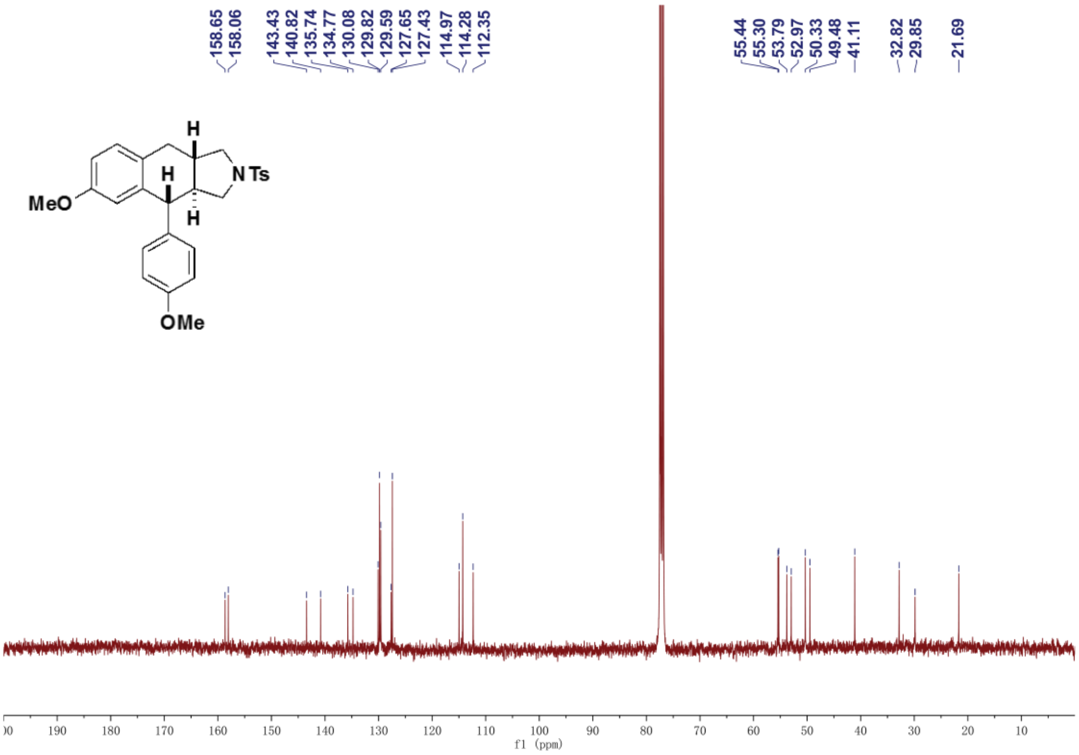

Supplementary Figure 198. ^{1}H NMR spectrum of compound **19d** (400 MHz, $CDCl_3$)

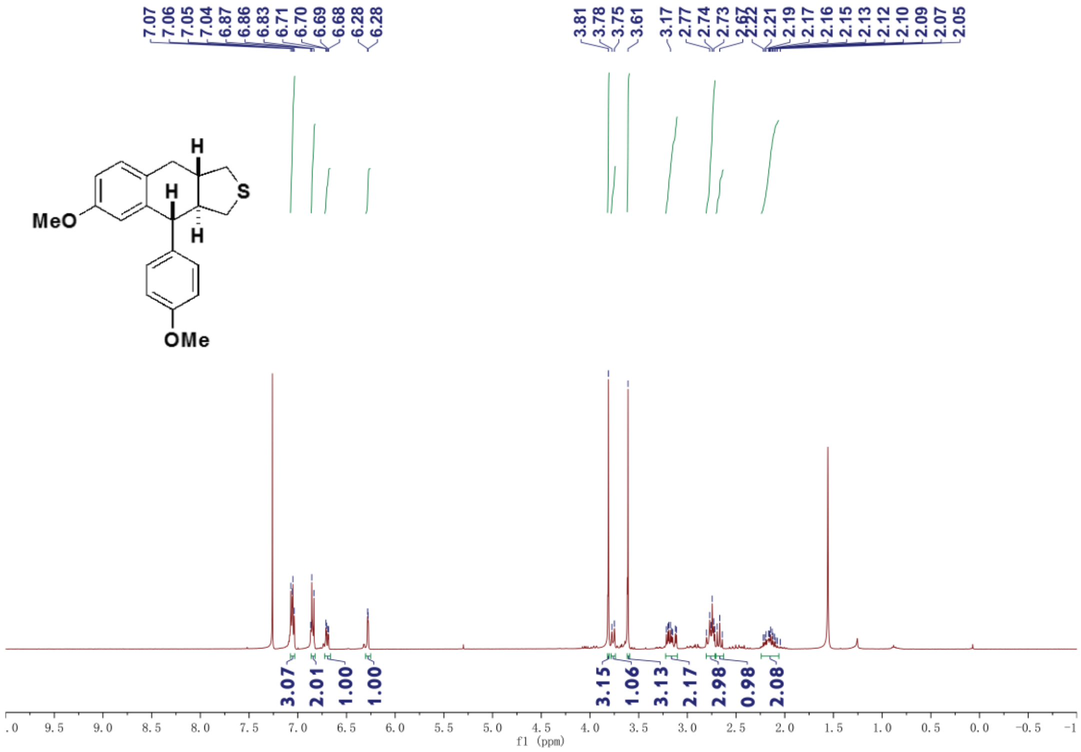

Supplementary Figure 199. ^{13}C NMR spectrum of compound **19d** (101 MHz, $CDCl_3$)

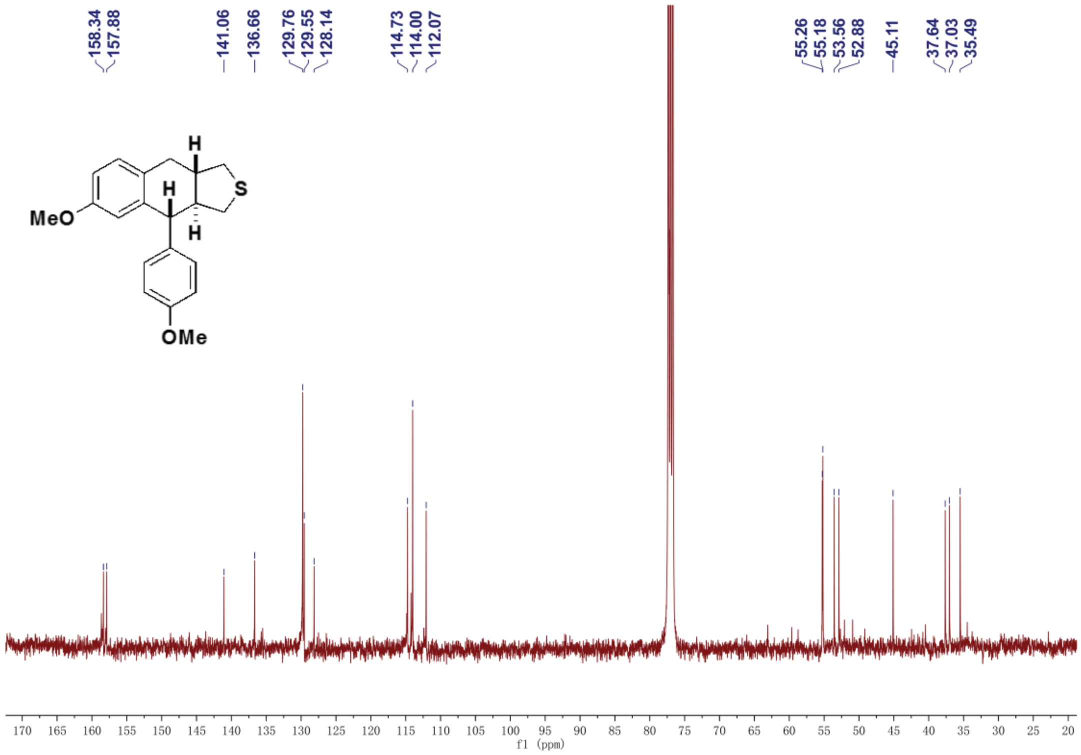

Supplementary Figure 200. ^{1}H NMR spectrum of compound **19e** (400 MHz, $CDCl_3$)

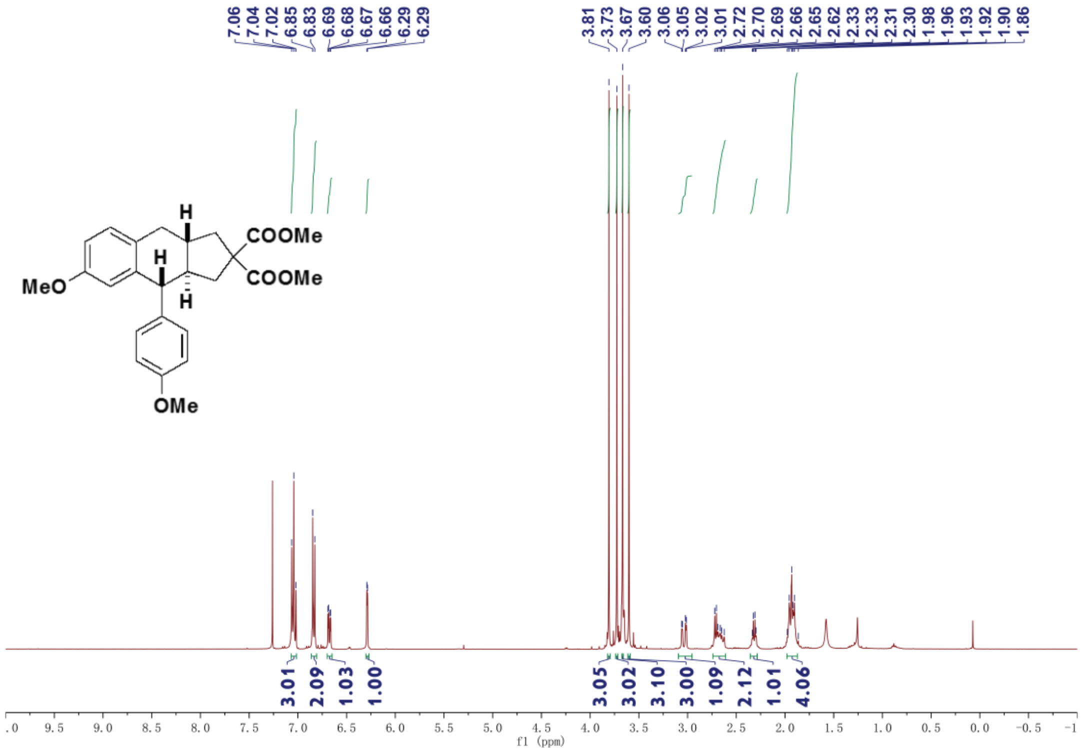

Supplementary Figure 201. ^{13}C NMR spectrum of compound **19e** (101 MHz, $CDCl_3$)

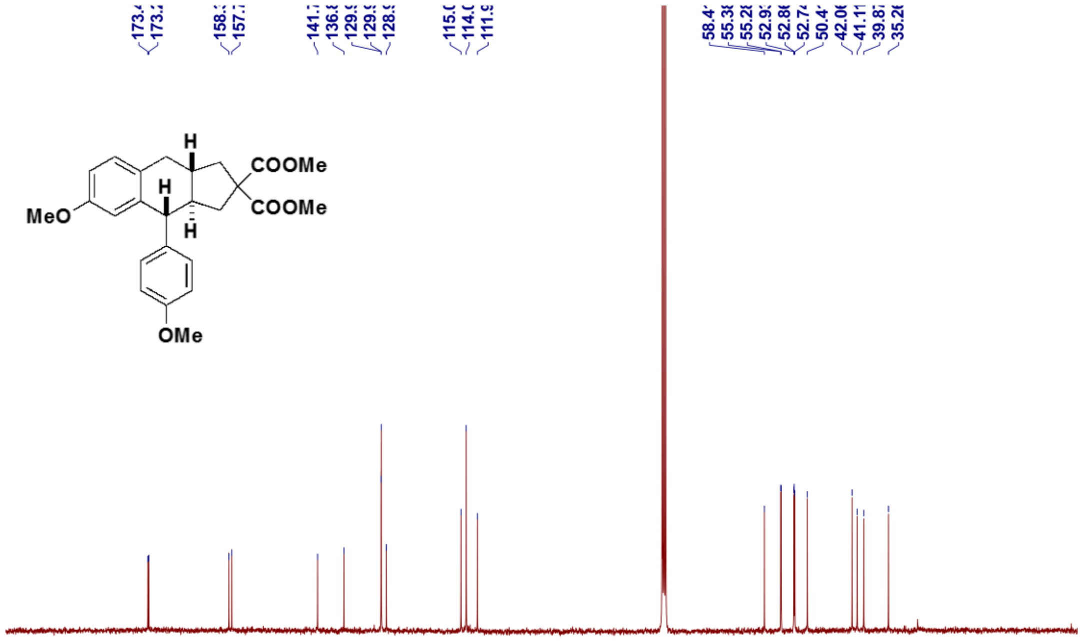

2. Supplementary references

1. Xiang, J. C., Wang, Q. & Zhu, J. Radical-cation cascade to aryltetralin cyclic ether lignans under visible-light photoredox catalysis. *Angew. Chem. Int. Ed.* **59**, 21195-21202 (2020).

2. Ischay, M. A., Lu, Z & Yoon, T. P. [2+2] Cycloadditions by Oxidative Visible Light Photocatalysis. *J. Am. Chem. Soc.* **132**, 8572-8574 (2010).

3. Lu, Z. & Yoon, T. P. Visible Light Photocatalysis of [2+2] Styrene Cycloadditions by Energy Transfer. *Angew. Chem. Int. Ed.* **51**, 10329-10332 (2012).

4. Wu, J., Dou, Y., Guillot, R., Kouklovsky, C. & Vincent, G. Electrochemical Dearomative 2,3-Difunctionalization of Indoles. *J. Am. Chem. Soc.* **141**, 2832–2837 (2019).

5. Lee, B. J., DeGlopper, K. S. & Yoon, T. P. Site-Selective Alkoxylation of Benzylic C−H Bonds by Photoredox Catalysis. *Angew. Chem. Int. Ed.* **59**, 197-202 (2020).

6. Gottlieb, H., Kotlyar, V. & Nudelman, A. NMR Chemical Shifts of Common Laboratory Solvents as Trace Impurities. *J. Org. Chem.* **62**, 7512-7515 (1997).

7. Wang, B. G., Ebel, R., Wang, C. Y., Wray, V. & Proksch, P. New methoxylated aryltetrahydronaphthalene lignans and a norlignan from Aglaia cordata. *Tetrahedron Lett.* **43**, 5783-5787 (2002).

8. Wang, B. G., Ebel, R., Nugroho, B. W., Prijono, D., Frank, W., Steube, K. G., Jao, X. J. & Proksch, P. Aglacins A−D, First Representatives of a New Class of Aryltetralin Cyclic Ether Lignans from Aglaia cordata. *J. Nat. Prod*. **64**, 1521-1526 (2001).
